# Immune cells transcriptome-based drug repositioning for multiple sclerosis

**DOI:** 10.3389/fimmu.2022.1020721

**Published:** 2022-10-20

**Authors:** Xinyue Yin, Xinming Rang, Xiangxiang Hong, Yinglian Zhou, Chaohan Xu, Jin Fu

**Affiliations:** ^1^ Department of Neurology, the Second Affiliated Hospital of Harbin Medical University, Harbin, China; ^2^ College of Bioinformatics Science and Technology, Harbin Medical University, Harbin, China

**Keywords:** multiple sclerosis, transcriptome, drug repositioning, differentially expressed gene, candidate drug

## Abstract

**Objective:**

Finding target genes and target pathways of existing drugs for drug repositioning in multiple sclerosis (MS) based on transcriptomic changes in MS immune cells.

**Materials and Methods:**

Based on transcriptome data from Gene Expression Omnibus (GEO) database, differentially expressed genes (DEGs) in MS patients without treatment were identified by bioinformatics analysis according to the type of immune cells, as well as DEGs in MS patients before and after drug administration. Hub target genes of the drug for MS were analyzed by constructing the protein-protein interaction network, and candidate drugs targeting 2 or more hub target genes were obtained through the connectivity map (CMap) database and Drugbank database. Then, the enriched pathways of MS patients without treatment and the enriched pathways of MS patients before and after drug administration were intersected to obtain the target pathways of the drug for MS, and the candidate drugs targeting 2 or more target pathways were obtained through Kyoto Encyclopedia of Genes and Genomes (KEGG) database.

**Results:**

We obtained 50 hub target genes for CD4^+^ T cells in Fingolimod for MS, 15 hub target genes for Plasmacytoid dendritic cells (pDCs) and 7 hub target genes for Peripheral blood mononuclear cells (PBMC) in interferon-β (IFN-β) for MS. 6 candidate drugs targeting two or more hub targets (Fostamatinib, Copper, Artenimol, Phenethyl isothiocyanate, Aspirin and Zinc) were obtained. In addition, we obtained 4 target pathways for CD19^+^ B cells and 15 target pathways for CD4^+^ T cells in Fingolimod for MS, 7 target pathways for pDCs and 6 target pathways for PBMC in IFN-β for MS, most of which belong to the immune system and viral infectious disease pathways. We obtained 69 candidate drugs targeting two target pathways.

**Conclusion:**

We found that applying candidate drugs that target both the “PI3K-Akt signaling pathway” and “Chemokine signaling pathway” (e.g., Nemiralisib and Umbralisib) or applying tyrosine kinase inhibitors (e.g., Fostamatinib) may be potential therapies for the treatment of MS.

## Introduction

Multiple sclerosis (MS) is one of the most common idiopathic inflammatory demyelinating diseases, involving the white matter of the central nervous system (CNS), with widely distributed lesions, high recurrence rate, and disability rate (1). The number of MS patients worldwide has increased to 2.8 million in 2020, the global prevalence rate was 35.9 per 100,000 people [95%CI: 35.87, 35.95], and the incidence rate was 2.1 per 100,000 persons/year [95%CI: 2.09, 2.12] (2). However, the treatment effect of MS patients is not ideal. Its effective prevention and treatment are urgent problems that need to be solved clinically.

For patients with poor prognosis and recurrent MS, the use of disease-modifying therapies (DMTs) should be considered early (3). There are currently more than a dozen DMTs approved for the treatment of MS, such as subcutaneous injection of Interferon-β (IFN-β), Fingolimod, Teriflunomide, Ocrelizumab, Natalizumab, etc. However, the medicines for the treatment of MS are expensive, have different degrees of side effects, and the control of the recurrence rate of MS is not ideal (4). Therefore, it is very important to find effective MS drugs.

Traditional drug development has the characteristics of high cost, low success rate, lengthy development cycles, and heavy financial burden on patients. Drug repositioning is inspired by Sildenafil and Azidothymidine, which refers to the discovery of new indications of existing drugs in addition to the original indications. It is an increasingly attractive treatment discovery model, which not only saves time and money, but also has the advantage of already having been tested for safety, dosage, and toxicity (5, 6). There are no previous reports in the literature regarding the application of bioinformatic analysis to MS drug repositioning. Therefore, we aim to apply this method to determine candidate drugs for the treatment of MS.

The etiology and pathogenesis of MS are not fully understood, but scholars generally believe that MS is an autoimmune disease mediated by myelin-specific CD4^+^ T cell attacks on CNS myelin sheaths triggered by environmental and infectious factors based on genetic susceptibility (7). CD4^+^ T cells can be divided into 4 subpopulations, namely Th1, Th2, Th17 and Treg, according to their different functions. Th1 cells trigger neuroinflammatory responses in MS pathogenesis, while Th2 cells may have a protective role in suppressing neuroinflammation in MS pathogenesis. Th17 cells promote blood-brain barrier (BBB) injury and enter the CNS to trigger neuroinflammation, while Treg cells have immunosuppressive functions that downregulate the immune response. The tilt of the Th1/Th2 axis toward Th1 and the tilt of the Th17/Treg axis toward Th17 are both strongly associated with the development of MS (8). In recent years, the effective application of anti-CD20 therapy has also revealed an important role of B cells in the pathogenesis of MS (9). B cells play an important role in MS pathogenesis through antibody-dependent and antibody-independent mechanisms (10). Antibody-dependent mechanisms promote MS pathogenesis by producing autoantibodies against specific CNS tissues, while antibody-independent mechanisms promote MS pathogenesis by inducing B-cell receptor (BCR) internalization of autoantigens and presentation to specific CNS pathogenic T cells to promote T-cell activation, by producing cytokines and by forming ectopic lymphoid tissue (11). In addition, plasmacytoid dendritic cells (pDCs) in MS patients may also be involved in MS pathogenesis due to their pro-inflammatory state, their migratory phenotype, and the influence of genetic risk factors (12). Therefore, our study analyzed the transcriptomic data of the above-mentioned immune cells from MS patients. In terms of treatment, DMTs are mainly used for MS remission, including IFN-β, Glatiramer acetate, Teriflunomide, Dimethyl fumarate, Fingolimod, Ocrelizumab, Alemtuzumab, Natalizumab, and Mitoxantrone, etc. Among them, IFN-β and Fingolimod are widely used in early clinical practice with significant efficacy. IFN-β has antiviral and immunomodulatory effects and inhibits T cell migration by disrupting the balance of anti-inflammatory Th2 cells and by blocking metalloproteinases and adhesion molecules (13). Fingolimod is an inhibitor of the sphingosine-1 phosphate (S1P) receptor and inhibits S1P-mediated T-lymphocyte migration, thus favoring the retention of T-lymphocytes in lymph nodes and triggering a “homing response” of peripheral lymphocytes and thus immunosuppression (14). Patients with MS treated with Fingolimod or IFN-β have a significantly lower annualized relapse rate (ARR) (15, 16), so our analysis is reliable using the currently reported transcriptomic dataset of MS patients treated with these two drugs. We obtained the original data from Gene Expression Omnibus (GEO) database, analyzed the target genes and pathways in drug treatment of MS, and obtained potential MS candidate drugs targeting these target genes/pathways based on the connectivity map (CMap) database, Drugbank database and Kyoto Encyclopedia of Genes and Genomes (KEGG) database. Our study may help provide clues to potential MS therapeutic strategies.

## Materials and methods

### Identification of MS transcriptome data

A total of 295 sets of MS transcriptomic data were obtained in the GEO database (https://www.ncbi.nlm.nih.gov/geo/), and transcriptomic datasets of MS patients without treatment, as well as transcriptomic datasets of MS patients before and after treatment, were screened according to the following criteria (17). Inclusion criteria: ① The study samples were immune cells of patients with relapsing-remitting multiple sclerosis (RRMS); ② The number of samples in the experimental group and the control group should not be less than 3 (First, the transcriptome datasets of MS patients without treatment were screened, in which MS patients were the experimental group and healthy people were the control group; second, the transcriptome datasets of MS patients before and after medication were screened, in which the experimental group was the MS patients after the treatment, and the control group was the MS patients before the treatment); MS patients without treatment and MS patients before the treatment have not received DMTs or have not received DMTs for at least one month before the start of the experiment; There is an additional inclusion criteria for the transcriptome datasets of MS patients before and after medication: ④ The drugs used for MS patients before and after the treatment were widely used clinically and have significant effects.

### Transcriptome data preprocessing

The mRNA raw data* *(CEL files) and the annotation file of the sequencing platform were downloaded from the GEO database and processed using the R language. Due to the large difference between the data, the data were* *log2* t*ransformed and normalized using quantile normalization.

### Identification of the differentially expressed genes

The R package “Limma” was used for linear fitting and difference analysis on each group of data to calculate the difference in gene expression between MS patients without treatment and the difference in gene expression of MS patients before and after medication (18). Differentially expressed genes (DEGs) were screened by *P* < 0.05 and fold-change (FC) > 1.2 or 0 < FC < 1/1.2. When FC > 1.2, they were regarded as up-regulated DEGs and when 0 < FC < 1/1.2, they were regarded as down-regulated DEGs.

### Identification of target genes for drug treatment of MS

According to the type of immune cells, the up-regulated DEGs of MS patients without treatment and the down-regulated DEGs of MS patients before and after medication were intersected to obtain the up-regulated target genes for MS drug therapy, the down-regulated DEGs of MS patients without treatment and the up-regulated DEGs of MS patients before and after medication were intersected to obtain the down-regulated target genes for MS drug therapy.

### Construction of protein-protein interaction network and identification of hub target genes for drug treatment of MS

According to the type of immune cells, the protein-protein interaction (PPI) network of target genes was constructed using the STRING* *database (https://cn.string-db.org/, version 11.5) (combined* *confidence* *score* *> 0.400), visualized by Cytoscape (https://cytoscape.org/, version 3.9.1) and the degree of nodes was calculated (19). Using the CytoHubba plugin in Cytoscape, the top 10% of nodes with the highest degree of PPI network connectivity were identified as hub target genes.

### Identification of candidate drugs through CMap database and Drugbank database

Candidate drugs targeting hub target genes were identified through the CMap database (https://clue.io/) and Drugbank database (https://go.drugbank.com/) according to the different types of immune cells (Inclusion criteria: Approved/Investigational drugs) (20, 21). The drug-hub target gene network diagram was visualized through Cytoscape.

### Pathway enrichment analysis of DEGs

The pathway enrichment analysis for the DEGs of MS patients without treatment and the DEGs of MS patients before and after the medication was performed using Rstudio (Ver.3.6.1) according to the different types of immune cells (22), and only the pathways with *P* < 0.05 were included. Then, the pathways of MS patients without treatment and the pathways of MS patients before and after medication were intersected to obtain the target pathways of MS treatment. According to the type of immune cells, calculate the types of DEGs of MS patients without treatment included in each target pathway, the types of DEGs of MS patients before and after medication included in each target pathway, and the types of common DEGs included in each target pathway, and identify the target pathways that were mainly targeted when these drugs were used to treat MS.

### Identification of candidate drugs through the KEGG database

According to the type of immune cells, the candidate drugs targeting target pathways were obtained through the KEGG database (www.genome.jp/kegg/pathway.html) and drug-pathway interaction networks were conducted by Cytoscape (23).

### Proteomic data validation

We extensively screened proteomic data of MS patients without treatment and MS patients before and after drug administration that applying liquid chromatography-tandem mass spectrometry (LC-MS/MS) for further validation through the Proteomics Identification Database (PRIDE, http://www.ebi.ac.uk/pride) (24), the GEO database and Google Scholar (https://scholar.google.com) (25). We only found PXD011785 and PXD028702 data from the PRIDE that could be used to validate the transcriptomic data of MS patients without treatment, both of which were protein-level data on CD4^+^ T cells in MS patients without treatment (26, 27). Unfortunately, we did not find the proteomic dataset of MS patients before and after drug administration, so only the transcriptomic data of MS patients without treatment on CD4^+^ T cells were validated in this article.

**Figure 1:**
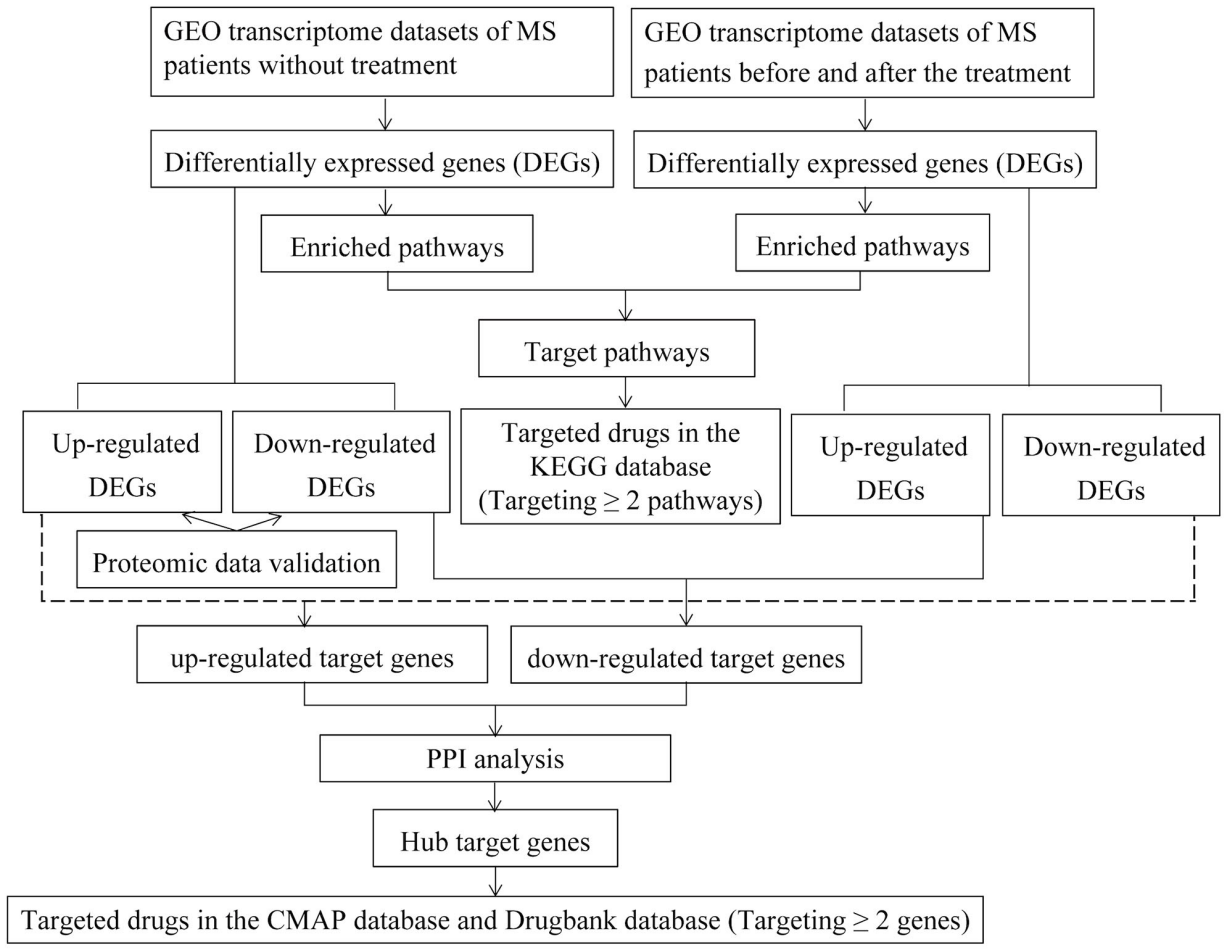
The workflow of the study.

The workflow of the study was shown in **Figure 1**.

## Results

### Identification of DEGs of MS patients without treatment and DEGs before and after IFN-β or Fingolimod treatment

4 sets of transcriptome datasets of CD19^+^ B cells, CD4^+^ T cells, pDCs, and Peripheral blood mononuclear cells (PBMC) from MS patients without treatment were obtained (GSE117935 (28), GSE172009 (29), GSE37750 (30), GSE41890 (31). Then, according to the type of CD19^+^ B cells, CD4^+^ T cells, pDCs and PBMC, 5142 DEGs of MS patients without treatment were obtained according to the standards of *P* < 0.05 and FC > 1.2 or 0 < FC < 1/1.2 (CD19^+^ B cells: 293 DEGs; CD4^+^ T cells: 2032 DEGs; pDCs: 1712 DEGs; PBMC: 1561 DEGs) (**Table 1**; **Supplementary Table S1**). Furthermore, we could only obtain the transcriptome datasets of MS patients before and after the application of IFN-β or Fingolimod. There were 2 sets of transcriptome datasets of CD19^+^ B cells and CD4^+^ T cells from MS patients before and after the application of Fingolimod and 2 sets of transcriptome datasets of pDCs and PBMC from MS patients before and after the application of IFN-β (GSE81604 (32, 33), GSE73079 (32–34), GSE37750 (30), GSE33464 (35, 36)). According to the type of CD19^+^ B cells, CD4^+^ T cells, pDCs and PBMC, 14300 DEGs in MS patients before and after the application of Fingolimod and IFN-β were obtained according to the standards of *P* < 0.05 and FC > 1.2 or 0 < FC < 1/1.2 (CD19^+^ B cells: 1164 DEGs; CD4^+^ T cells: 13201 DEGs; pDCs: 668 DEGs; PBMC: 593 DEGs) (**Table 2**, **Supplementary Table S2**).

**Table 1:** Transcriptome datasets of MS patients without treatment.

ID	Disease	Platform ID	Case/control	Sample	Publish time	DEG	
						Up-regulated DEG	Down-regulated DEG
GSE117935	RRMS	GPL5175	10/10	CD19^+^ B cells	2018	216	77
GSE172009	RRMS	GPL20301 GPL24676	4/4	CD4^+^ T cells	2021	163	1869
GSE37750	RRMS	GPL570	9/8	pDCs	2015	792	920
GSE41890	RRMS	GPL6244	8/4	Peripheral blood leukocytes	2013	883	678

**Table 2:** Transcriptome datasets of MS patients before and after medication.

ID	Disease	Platform ID	Drug	Case/control	Sample	Publish time	DEG	
							Up-regulated DEG	Down-regulated DEG
GSE81604	RRMS	GPL17586	Fingolimod	5/5	CD19^+^ B cells	2016	723	441
GSE73079	RRMS	GPL17586	Fingolimod	5/5	CD4^+^ T cells	2015	4162	9039
GSE37750	RRMS	GPL570	IFN-β	9/9	pDCs	2015	263	405
GSE33464	RRMS	GPL14837	IFN-β	12/12	PBMC	2011	296	297

### Identification of hub target genes in MS patients before and after the application of Fingolimod or IFN-β

**Figure 2:**
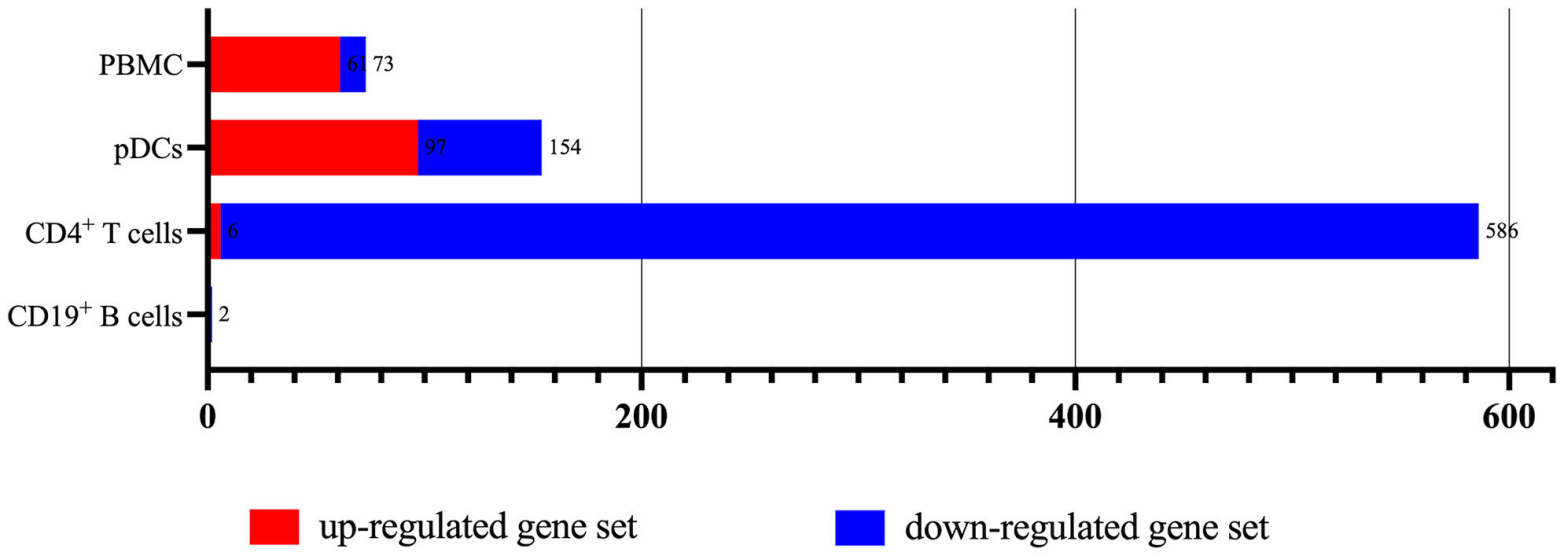
The types of up-regulated and down-regulated target genes for CD19^+^ B cells, CD4^+^ T cells, pDCs, and PBMC in MS patients treated with Fingolimod or IFN-β.

**Figure 3:**
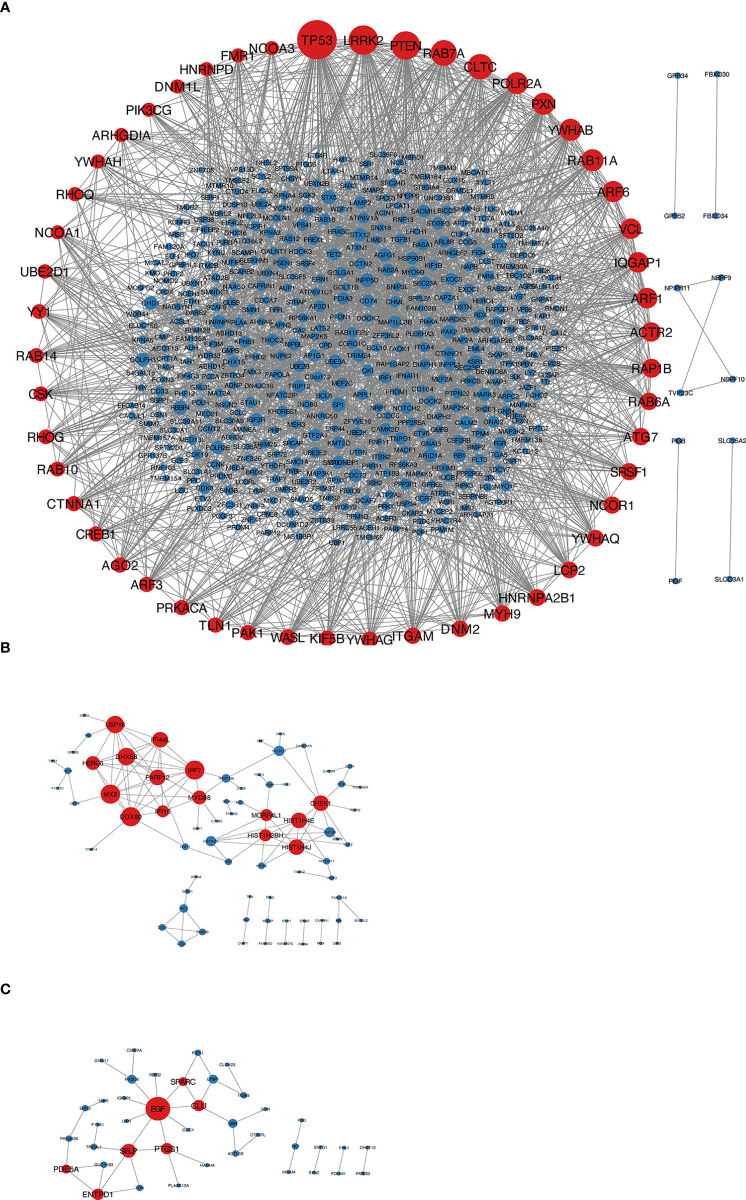
**(A)** Target genes of CD4^+^ T cells in MS patients treated with Fingolimod, red nodes were hub target genes; **(B)** target genes of pDCs in MS patients treated with IFN-β, red nodes were hub target genes; **(C)** target genes of PBMC in MS patients treated with IFN-β, red nodes were hub target genes.

According to the type of CD19^+^ B cells, CD4^+^ T cells, pDCs, and PBMC, a total of 164 up-regulated target genes and 649 down-regulated target genes were obtained (**Figure 2**; **Supplementary Table S3**). The types of target genes targeting CD4^+^ T cells were the most and mainly focused on down-regulated target genes. A PPI network of 560 nodes and 2386 edges was constructed using target genes of CD4^+^ T cells from MS patients after the application of Fingolimod, and 50 hub target genes (top 10%: degree ≥ 21) were identified by PPI analysis and visualization with Cytoscape. In addition, a PPI network with 138 nodes and 115 edges was constructed using target genes targeting pDCs from MS patients after the application of IFN-β, and 15 hub target genes were identified (top 10%: degree ≥ 6). A PPI network with 73 nodes and 40 edges was constructed using target genes targeting PBMC from MS patients after the application of IFN-β, and 7 hub target genes (top 10%: degree ≥ 3) were identified (**Figure 3**).

### Identification of potential candidate drugs for the treatment of MS through the CMap database and Drugbank database

**Figure 4:**
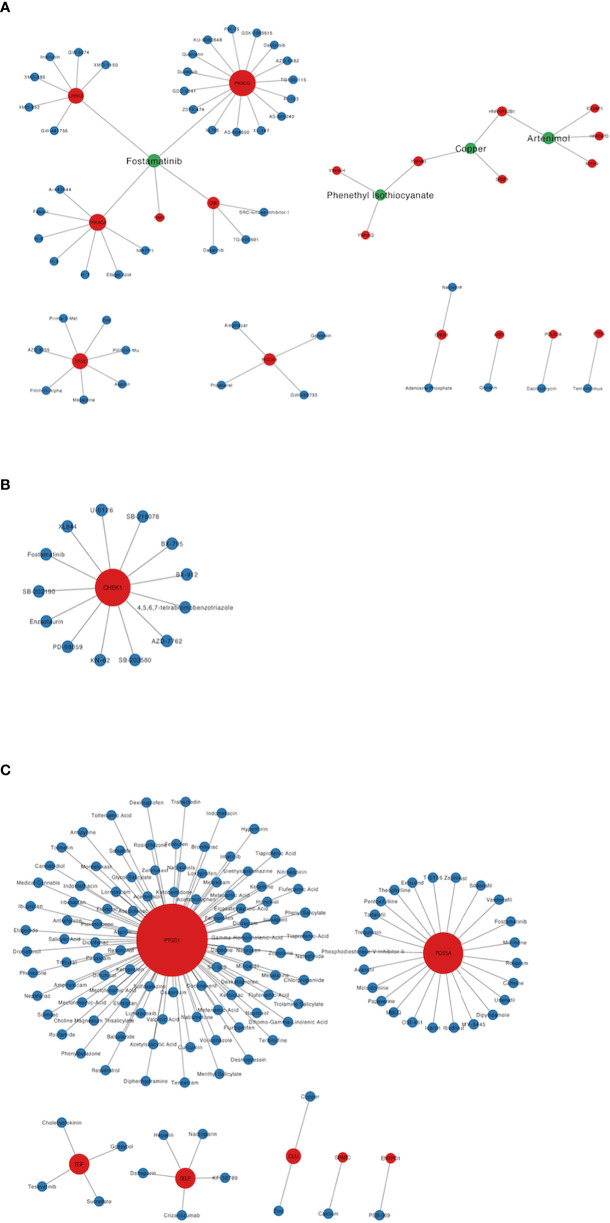
Candidate drugs targeting hub target genes were identified based on the CMap database and Drugbank database. Red nodes represent hub target genes, blue nodes represent candidate drugs targeting hub target genes, and green nodes represent candidate drugs targeting 2 or more than 2 hub target genes. **(A)** The candidate drugs targeting hub target genes of CD4+ T cells in MS patients treated with Fingolimod. **(B)** The candidate drugs targeting hub target genes of pDCs in MS patients treated with IFN-β. **(C)** The candidate drugs targeting hub target genes of PBMC in MS patients treated with IFN-β.

A total of 193 candidate drugs (CD4^+^ T cells: 51; pDCs: 13; PBMC: 134) targeting hub target genes were obtained through the CMap database and Drugbank database according to the types of CD4^+^ T cells, pDCs, and PBMC (**Figure 4**), most of which belonged to antineoplastic drugs and 6 candidates targeted more than 2 hub target genes (**Table 3**).

**Table 3:** The candidate drugs that target 2 or more than 2 hub target genes.

Drug	Target Gene	Effect	Sample
Fostamatinib	LRRK2, PAK1, PRKACA, CSK, PIK3CG, CHEK1, PDE5A	Antineoplastic, Immunomodulator, Spleen tyrosine kinase inhibitor	CD4+ T cells, pDCs, PBMC
Copper	YWHAB, SRSF1, HNRNPA2B1, CLU	Supplement	CD4+ T cells, PBMC
Artenimol	IQGAP1, HNRNPA2B1, MYH9, HNRNPD	Antimalarial	CD4+ T cells
Phenethyl Isothiocyanate	YWHAB, YWHAQ, YWHAH	Antineoplastic	CD4+ T cells
Aspirin	TP53, PTGS1	Analgesic, Anti-inflammatory, Antipyretic, Antirheumatic, Antiplatelet, Cyclooxygenase inhibitor	CD4+ T cells, PBMC
Zinc	TP53, CLU	Supplement	CD4+ T cells, PBMC

### Identification of target pathways for Fingolimod and IFN-β in the treatment of MS

**Figure 5:**
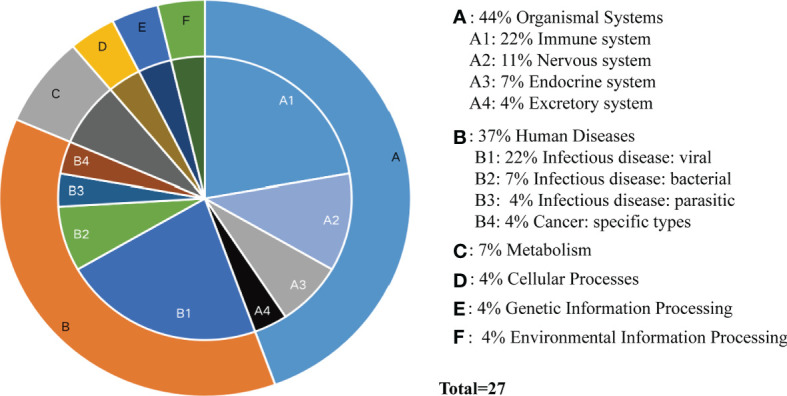
Classification of target pathways in MS patients treated with Fingolimod or IFN-β. **(A)** Organismal Systems; **(B)** Human Diseases; **(C)** Metabolism; **(D)** Cellular Processes; **(E)** Genetic Information Processing; **(F)** Environmental Information Processing. “Fatty acid metabolism” is not classified yet.

**Figure 6:**
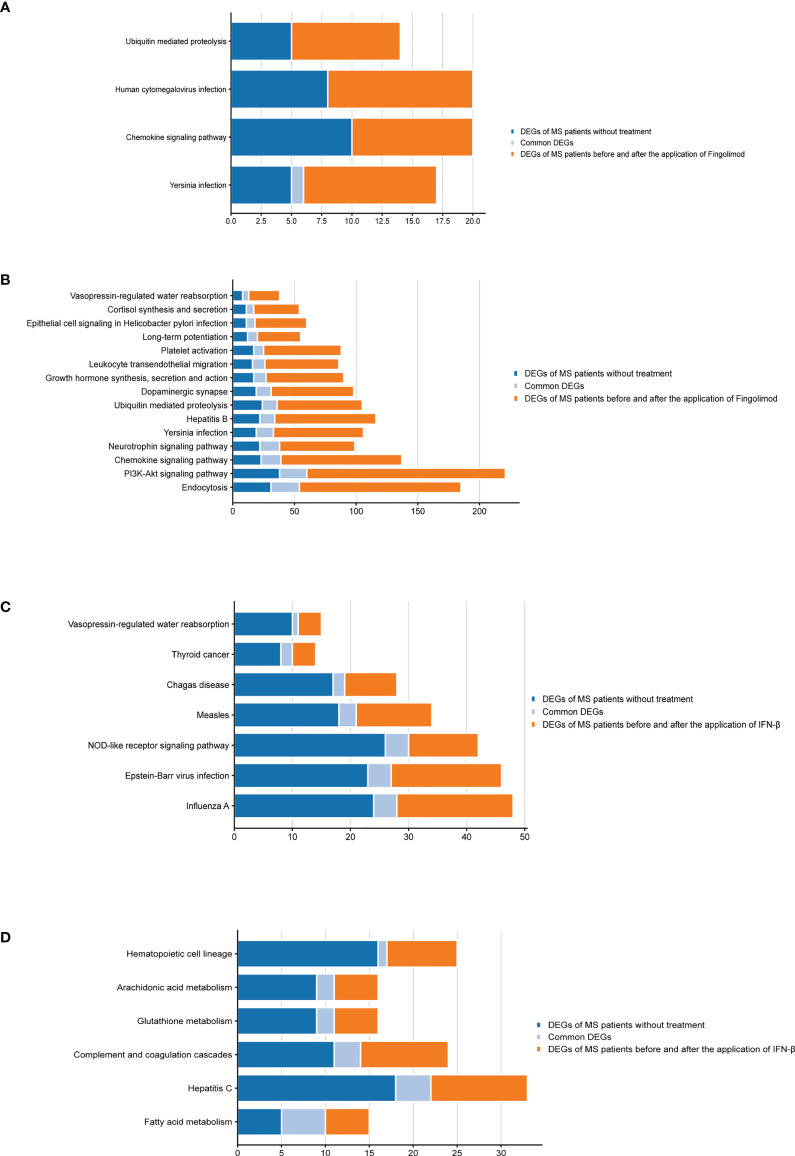
Bar graphs of the target pathways in MS patients treated with Fingolimod or IFN-β. **(A)** DEGs contained in the target pathways of CD19+ B cells in MS patients treated with Fingolimod; **(B)** DEGs contained in the target pathways of CD4+ T cells in MS patients treated with Fingolimod; **(C)** DEGs contained in the target pathways of pDCs in MS patients treated with IFN-β; **(D)** DEGs contained in the target pathways of PBMC in MS patients treated with IFN-β.

According to the type of CD19^+^ B cells, CD4^+^ T cells, pDCs, and PBMC, a total of 139 pathways were enriched at *P* < 0.05 for DEGs in MS patients without treatment (**Supplementary Table S4**), and a total of 112 pathways were enriched at *P* < 0.05 for DEGs in MS patients before and after Fingolimod or IFN-β application (**Supplementary Table S5**). The 28 target pathways of Fingolimod and IFN-β for MS treatment were obtained by taking the intersection processing (**Tables 4**, **5**). The target pathways were classified according to the classification of pathways in the KEGG database into immune system pathways (22%), nervous system pathways (11%), endocrine system pathways (7%), excretory system pathways (4%), viral infectious disease pathways (22%), bacterial infectious disease pathways (7%), parasitic infectious disease pathways (4%), specific types of cancer pathways (4%), metabolism pathways (7%), cellular process pathway (4%), genetic information processing pathway (4%) and environmental information processing pathway (4%) (**Figure 5**). Among them, the immune system pathways and viral infectious disease pathways accounted for the largest proportion. The main target pathways of Fingolimod and IFN-β for MS treatment were identified according to the type of CD19^+^ B cells, CD4^+^ T cells, pDCs, and PBMC (**Figure 6**), where the main target pathway of Fingolimod for MS treatment against CD19^+^ B cells was “Yersinia infection”, the main target pathways for CD4^+^ T cells in Fingolimod therapy for MS were “Endocytosis”, “PI3K-Akt signaling pathway”, “Chemokine signaling pathway” and “Neurotrophin signaling pathway”, the main target pathways for pDCs in MS treatment with IFN-β were “Influenza A”, “Epstein-Barr virus infection” and “NOD-like receptor signaling pathway”, the main target pathways for PBMC in MS treatment with IFN-β were “Fatty acid metabolism” and “Hepatitis C”.

**Table 4:** The number of enriched pathways of DEGs in MS patients without treatment, the number of enriched pathways of DEGs before and after application of Fingolimod or IFN-β, and the number of target pathways.

ID	Sample	Drug	Pathway	Target pathway
GSE117935	CD19^+^ B cells	/	50	4
GSE81604		Fingolimod	24	
GSE172009	CD4^+^ T cells	/	40	15
GSE73079		Fingolimod	75	
GSE37750	pDCs	/	54	7
GSE37750		IFN-β	20	
GSE41890	PBMC	/	58	6
GSE33464		IFN-β	19	

**Table 5:** Target pathways of Fingolimod and IFN-β for MS.

Sample	Pathway ID	Pathway Name	Pathway Class
CD19^+^ B cells	hsa04062	Chemokine signaling pathway	Organismal Systems (Immune system)
	hsa05135	Yersinia infection	Human Diseases (Infectious disease: bacterial)
	hsa05163	Human cytomegalovirus infection	Human Diseases (Infectious disease: viral)
	hsa04120	Ubiquitin mediated proteolysis	Genetic Information Processing (Folding, sorting and degradation)
CD4^+^ T Cells	hsa04670	Leukocyte transendothelial migration	Organismal Systems (Immune system)
	hsa04611	Platelet activation	Organismal Systems (Immune system)
	hsa04062	Chemokine signaling pathway	Organismal Systems (Immune system)
	hsa04722	Neurotrophin signaling pathway	Organismal Systems (Nervous system)
	hsa04720	Long-term potentiation	Organismal Systems (Nervous system)
	hsa04728	Dopaminergic synapse	Organismal Systems (Nervous system)
	hsa04927	Cortisol synthesis and secretion	Organismal Systems (Endocrine system)
	hsa04935	Growth hormone synthesis, secretion and action	Organismal Systems (Endocrine system)
	hsa04962	Vasopressin-regulated water reabsorption	Organismal Systems (Excretory system)
	hsa05161	Hepatitis B	Human Diseases (Infectious disease: viral)
	hsa05135	Yersinia infection	Human Diseases (Infectious disease: bacterial)
	hsa05120	Epithelial cell signaling in Helicobacter pylori infection	Human Diseases (Infectious disease: bacterial)
	hsa04120	Ubiquitin mediated proteolysis	Genetic Information Processing (Folding, sorting and degradation)
	hsa04144	Endocytosis	Cellular Processes (catabolism)
	hsa04151	PI3K-Akt signaling pathway	Environmental Information Processing (Signal transduction)
pDCs	hsa04621	NOD-like receptor signaling pathway	Organismal Systems (Immune system)
	hsa04962	Vasopressin-regulated water reabsorption	Organismal Systems (Excretory system)
	hsa05164	Influenza A	Human Diseases (Infectious disease: viral)
	hsa05162	Measles	Human Diseases (Infectious disease: viral)
	hsa05169	Epstein-Barr virus infection	Human Diseases (Infectious disease: viral)
	hsa05142	Chagas disease	Human Diseases (Infectious disease: parasitic)
	hsa05216	Thyroid cancer	Human Diseases (Cancer: specific types)
PBMC	hsa04610	Complement and coagulation cascades	Organismal Systems (Immune system)
	hsa04640	Hematopoietic cell lineage	Organismal Systems (Immune system)
	hsa05160	Hepatitis C	Human Diseases (Infectious disease: viral)
	hsa00590	Arachidonic acid metabolism	Metabolism (Lipid metabolism)
	hsa00480	Glutathione metabolism	Metabolism (Metabolism of other amino acids)
	hsa01212	Fatty acid metabolism	

### Identification of potential candidate drugs for the treatment of MS based on the KEGG database

Our approach is based on two biological assumptions: first, proteins always have cascading effects in pathways, not just acting alone; second, drugs exert therapeutic effects by modulating pathways involved in disease pathology, rather than directly targeting disease-related proteins (37), so we considered drug candidates that target pathways as potential therapeutic agents for MS. A total of 1062 candidate drugs targeting target pathways were obtained through the KEGG database (CD19^+^ B cells: 33; CD4^+^ T cells: 495; pDCs: 26; PBMC: 304) (**Supplementary Table S6**), most of which were antineoplastic and immunosuppressive drugs. There were 58 candidate drugs targeting 2 target pathways (**Supplementary Table S7**).

### Proteomic data validation

From a biological point of view, the transcriptome represents the intermediate state of gene expression, while proteins are the direct functional performers of the organism, and therefore the study of protein expression levels has an irreplaceable advantage. In the PXD011785 data, a total of 228 proteins were differentially expressed between MS patients and healthy individuals (*P* < 0.05), of which 24 proteins encoded genes consistent with the DEGs we obtained (**Supplementary Table S8**). Among them, 12 were regulated with the same trend at transcriptome level and protein level (TES, GABPA, ARF6, VCL, TYMP, LIMS1, YWHAG, ATP6V1A, PDIA3, ATP2A2, TPM4, CLTC). Among these 24 overlapping coding genes, YWHAG was involved in the “PI3K-Akt signaling pathway”. A total of 10 pathways were enriched for the 24 overlapping coding genes at *P* < 0.05 (**Supplementary Table S9**), in which the ‘‘Endocytosis’’ pathway was enriched in both transcriptome and proteome, further validating the confidence of the transcriptome data. In the PXD028702 data, a total of 18 proteins were differentially expressed (adjusted p ≤ 0.05) between MS patients and healthy individuals (DPH6, GNPDA2, ACAD8, CORO2A, PHF20L1, SRA1, EPC1, PTPN13, DENND10, LAMTOR5, NRDE2, PSMD5, GOPC, ASPH, TCEA3, RHOC, TYK2, BORCS6). With a more stringent threshold, we still obtained 2 protein-coding genes (SRA1 and DENND1) that were consistent with the DEGs we obtained.

## Discussion

MS is considered to be a chronic inflammatory and demyelinating disease of the CNS, and various immune cells play a crucial role in the development of MS (38). In this study, we obtained the target genes and target pathways of Fingolimod and IFN-β for MS treatment based on immune cell transcriptomic datasets of MS patients without treatment and immune cell transcriptomic datasets of MS patients before and after application of Fingolimod or IFN-β, and identified MS candidate drugs targeting hub target genes and target pathways.

### Target genes and target pathways for CD4^+^ T cells in the treatment of MS with Fingolimod

15 target pathways and 586 target genes (6 up-regulated target genes and 580 down-regulated target genes) were obtained by the analysis of CD4^+^ T cell transcriptomic data from MS patients without treatment and CD4^+^ T cell transcriptomic data before and after Fingolimod treatment, suggesting that Fingolimod treatment for MS mainly targeted CD4^+^ T cells, which was consistent with the mechanism that Fingolimod inhibited the migration of T lymphocytes to the CNS and thus relieved MS. Recent clinical trial data suggested that increased Th1/Th17 cells (CD4^+^ T cell subpopulation) in CNS tissue, cerebrospinal fluid, and blood predominate in the pathogenesis of MS (8), so we focused on target genes and target pathways that target CD4^+^ T cells.

Among these 15 target pathways, “Endocytosis”, “PI3K-Akt signaling pathway”, “Chemokine signaling pathway” and “Neurotrophin signaling pathway” are more important. Many studies have shown that the “PI3K-Akt signaling pathway” is associated with autoimmune diseases, inflammation and hematological malignancies and plays an important role in the activation and migration of leukocytes (39). PI3K can be classified into type I, II and III according to the structure and substrate. And according to its type I catalytic subunit, type I PI3K can be further subdivided into 4 subtypes (α, β, γ, δ). The distribution of PI3K expression differs among different catalytic subunits, with PI3Kα and PI3Kβ being expressed in a variety of cells, while PI3Kδ and PI3Kγ are only expressed in the immune system. Among them, PI3Kδ is highly expressed in all leukocyte types, conferring it an important position in immunotherapy. PI3Kδ protects CD4^+^ T cells from apoptosis during autoimmune responses (40). In PI3Kδ-inactivated mice, T cell activation and function were significantly reduced in experimental* *allergic* *encephalomyelitis (EAE) and fewer T cells were observed in CNS (40). AKT is a serine/threonine kinase, also known as protein kinase B. Inhibition of Akt phosphorylation in the CNS of EAE reduced the worsening of clinical symptoms (41). Regarding the effect of the PI3K-AKT pathway on MS patients, PI3K is a key signaling mediator of CD28. CD28 can promote the increase of c-myc and Glucose transporter type 1 (Glut1) in CD4^+^ T cells of MS patients by activating the PI3K-AKT pathway, upregulating glycolysis and increasing Th17 cell-associated inflammatory cytokine expression (42). Therefore, inhibition of PI3K-Akt activity plays an important role in the treatment of MS. In addition, the “Chemokine signaling pathway” is an important immune system-related pathway also associated with leukocyte migration (43), and changes in chemokine expression and distribution are closely associated with the pathological process of MS demyelination (44). There is evidence that CCR2 on human Th17 cells (CCR2(+) CCR5(-) memory CD4^+^ T cells) may serve as a therapeutic target for MS (45). Blockade of the “Chemokine signaling pathway” is expected to be a new therapeutic approach (45). The “Neurotrophin signaling pathway” is a neurologically relevant pathway. Oligodendrocyte precursor cells (OPCs) are differentiated into myelin-forming oligodendrocytes (OLs) under the influence of various factors (including trophic factors, growth factors, and inhibitory factors in the microenvironment), and on this basis, OLs cross-link with each other to form myelin sheaths outside the axons of the central nervous system, a process called myelin regeneration (46). Therefore, regulation of neurotrophic factors is crucial for myelin regeneration in MS patients. Although no studies clearly show the relevance of “Endocytosis” to the treatment of MS, based on our analysis, we believe that relevant studies are necessary.

The top 3 node degrees among the 50 hub target genes obtained by constructing PPI networks were TP53 (down-regulated in MS), LRRK2 (down-regulated in MS), and PTEN (down-regulated in MS). TP53 and PTEN are both tumor suppressor genes associated with PI3K-Akt signaling. Among them, TP53 is one of the most frequently inactivated tumor suppressor genes in human cancer (47). It is a downstream target of the PI3K-Akt signaling pathway, and activation of PI3K-Akt signaling decreases TP53 expression (48). Production of IL-6, granulocyte-macrophage colony-stimulating factor and IL-10 is significantly higher in TP53-deficient EAE mice than in wild-type EAE mice and CNS-infiltrating cells are less apoptotic, suggesting that TP53 may be involved in the regulatory process of EAE by controlling cytokine production and/or inhibiting apoptosis of inflammatory cells (49). PTEN is a phosphatidylinositol-3,4,5- trisphosphate (PIP3)-phosphatase that is required to antagonize PI3K-AKT signaling by dephosphorylating PIP3 on the cell membrane to generate PIP2, which in turn antagonizes PI3K-mediated cell growth, metabolism, proliferation and survival signaling (50). PTEN also dephosphorylates Akt and reduces Akt activation while blocking all downstream signaling events regulated by Akt (50). Reduced PTEN expression indirectly stimulates PI3K-AKT activity. In the last two decades, our understanding of PI3K has evolved from recognition of growth factors, G protein-coupled receptors (GPCR) and enzymatic activities associated with certain oncogene products to targets in cancer and inflammatory diseases (50). Among these, PTEN plays a key role in Th17 cell differentiation by blocking IL-2 expression, and PTEN deficiency increases IL-2, promotes phosphorylation of STAT5 and inhibits phosphorylation of STAT3, thereby inhibiting Th17 cell differentiation (51). LRRK2 is a protein kinase, a gene highly associated with Parkinson’s disease (52), and has not been studied in MS and EAE.

### Target genes and target pathways for CD19^+^ B cells in the treatment of MS with Fingolimod

4 target pathways were obtained by analyzing CD19^+^ B cell transcriptome data from MS patients without treatment and CD19^+^ B cell transcriptome data before and after Fingolimod treatment, and no target genes were obtained, suggesting that Fingolimod treatment in MS has less effect on B cells. Most of these 4 target pathways are associated with immune and infectious diseases, with “Yersinia infection” being the main target pathway. Yersinia is an intestinal bacterium and intestinal bacteria have an important regulatory role in CNS disorders. The concept of the “brain-gut axis” has been proposed early (53). The gut is rich in nerve cells and immune cells, which reach the brain *via* the vagus system or the immune system, thus affecting brain function (54). Intestinal microorganisms can be in direct contact with intestinal cells, and the metabolic by-products of intestinal microorganisms can also activate the intestinal nervous system, interfere with the intestinal neurometabolic secretion system, and regulate the intestinal immune system, and this mode of action is called the “intestinal microbial-gut-brain axis” (55). Intestinal bacteria have been shown to regulate the differentiation, maturation and activation of B cells (56). Yersinia infection can induce polyclonal B-cell activation leading to increased autoantibodies resulting in autoimmune rheumatic diseases such as reactive arthritis and Lyme disease (57), and the relationship between Yersinia infection and MS has not been confirmed.

### Target genes and target pathways for pDCs in the treatment of MS with IFN- β

7 target pathways and 154 target genes (97 up-regulated target genes and 57 down-regulated target genes) were obtained by analyzing the transcriptomic data of pDCs from MS patients without treatment and before and after the application of IFN-β treatment. Most of these 7 target pathways are associated with viral infectious diseases. We know that pDCs sense and process viral DNA through Toll-like receptor 9 (TLR9) and that IFN-β treatment of MS leads to a reduction in the activation of pDCs by viral pathogens and a decrease in the frequency of MS progression by inhibiting TLR9 processing (58). Therefore, it can explain the results obtained from our analysis that the hub target genes with the top 3 node degrees and most of the target pathways were associated with viral infectious diseases. Among them, “Epstein-Barr virus infection” is more important. EBV, one of the most studied viruses regarding MS, is a persistent and frequently reactivated virus with close to 100% epidemiological relevance to MS, triggering local inflammation near or within the CNS and thought to play a dominant role in MS pathogenesis (59).

By constructing a PPI network, 15 hub target genes were obtained, and the top 3 node degrees were MX2 (down-regulated in MS), DDX60 (down-regulated in MS) and IRF7 (down-regulated in MS), all of which were associated with the viral infection. IRF7 (Interferon regulatory factor 7) is a key regulator of the host antiviral defense response and is one of the most important members of the interferon regulatory factor family, playing an essential role in the induction of type I interferon synthesis and in the cellular innate immune response (60). IRF7-deficient mice have a higher degree of CNS leukocyte infiltration, and IRF7 is essential for regulating the inflammatory response in the CNS of MS patients (61). MX2 and DDX60 have not been studied about MS and EAE.

### Target genes and target pathways for PBMC in the treatment of MS with IFN- β

6 target pathways and 73 target genes (61 up-regulated target genes and 12 down-regulated target genes) were obtained by analyzing PBMC transcriptomic data from MS patients without treatment and before and after the application of IFN-β treatment. “Fatty acid metabolism” is more important, as fatty acids are key regulators in the gut, altering the balance between Th1 and Th17 and Treg cells in autoimmune neuroinflammation (62). Long-chain fatty acids (LCFAs) exacerbate EAE by increasing pathogenic Th1 and Th17 cell populations, and short-chain fatty acids (SCFAs) improve EAE and reduce axonal damage by promoting differentiation and proliferation of Treg cells (62), suggesting that regulation of fatty acid metabolism may have an impact on the autoimmune response of MS patients by regulating the intestinal immune microenvironment.

By constructing the PPI network, 7 hub target genes were obtained. Among them, the highest node degree target gene is EGF (upregulated in MS), which is an epidermal growth factor that binds to receptors on the cell membrane and activates the PI3K-Akt signaling pathway (63), and as mentioned earlier, inhibition of the “PI3K-Akt signaling pathway” is essential for MS treatment.

### Potential candidate drugs for MS treatment

Among the 6 candidates obtained for 2 or more hub target genes, Fostamatinib is particularly important, targeting 7 hub target genes (LRRK2, PAK1, PRKACA, CSK, PIK3CG, CHEK1, PDE5A), which are targets of CD4^+^ T cells in Fingolimod for MS and targets of pDCs and PBMCs in IFN-β for MS. Fostamatinib is a spleen tyrosine kinase (SYK) inhibitor. A phase 2 clinical trial of a tyrosine kinase inhibitor (TKI) (Evobrutinib) has been completed in MS with promising results (64), and data from a *post-hoc* analysis of a phase 2 trial of Evobrutinib were presented at the 37th Congress of the European Committee for Treatment and Research in Multiple Sclerosis (ECTRIMS) in 2021, confirming that oral Bruton’s tyrosine kinase inhibitor (BTKi) Evobrutinib affects brain injury associated with chronic inflammation in the CNS, making it the first BTKi shown to significantly reduce slowly expanding lesion (SEL) (65). Among these, SELs are chronic, active, demyelinating MS lesions that are considered to be early indicators of MS disease progression. Fostamatinib is currently used to treat chronic adult idiopathic thrombocytopenic purpura (ITP), which has been poorly treated with previous therapy, by blocking platelet destruction and is the only approved SYK inhibitor on the market (66). Clinical trials have also been conducted on Fostamatinib for the treatment of RA, but the results have not been satisfactory (67). Whether it is effective in treating MS remains unclear.

Based on the target pathways of CD19^+^ B cells and CD4^+^ T cells in Fingolimod for MS and pDCs and PBMCs in IFN-β for MS, we can know that the “PI3K-Akt signaling pathway” and the “Chemokine signaling pathway” are more important, so we focus on drugs that target these pathways simultaneously. Among the 58 candidate drugs obtained that target 2 target pathways, Nemiralisib and Umbralisib target both the “PI3K-Akt signaling pathway” and the “Chemokine signaling pathway”. Nemiralisib, a PI3Kδ inhibitor, is an anti-asthmatic and anti-inflammatory agent (68), while Umbralisib, a dual PI3Kδ/CK1ϵ inhibitor, is an anti-tumor agent currently used in the treatment of lymphoma (69). Neither of them has been studied in MS, and they deserve focused attention.

Our study has the following limitations. First, we could only obtain the transcriptomic dataset of MS patients treated with IFN-β and Fingolimod in the GEO database. If the transcriptome datasets of MS patients treated with other effective drugs are reported in the future, this method can continue to be used to find potential candidate drugs for MS. Second, in our study, the critical values of DEG were relatively low (*P* < 0.05, FC > 1.2 or 0 < FC < 1/1.2). When we increased the FC used for common cases to 2 and performed subsequent analyses, the DEGs for MS patients without treatment were shown in **Supplementary Table S10**, and the DEGs for MS patients before and after the application of IFN-β or Fingolimod were shown in **Supplementary Table S11**. Target genes for CD19^+^ B cells and PBMC were reduced to 0. The upregulated target genes for CD4^+^ T cells were reduced to 0 and the downregulated target genes were reduced to 55. The upregulated target genes of pDCs were reduced to 0 and the downregulated target genes were reduced to 2. In terms of pathway enrichment, CD19^+^ B cells and PBMC did not obtain target pathways, CD4^+^ T cells obtained 7 target pathways, and pDCs obtained 11 target pathways (**Supplementary Table S12**). Although the cutoff for DEGs was relatively low, the results based on transcriptome data were meaningful to a certain extent. Third, limited by the lack of compliant proteomic datasets, we were only able to validate a small portion of our transcriptomic data – the transcriptomic data on CD4^+^ T cells from MS patients without treatment, and protein validation may continue in the future. Fourth, no basic trials or clinical trials have been conducted with Nermiralisib, Umbralisib or Fostamatinib. More work needs to be done in this area to fully realize the practical value of this study.

## Conclusion

In this study, we applied bioinformatics analysis of MS transcriptome data to reposition drugs that may treat MS, which can help identify target genes and target pathways for the treatment of MS, redirect the use of approved drugs, and find new effective drugs that may treat MS. According to our analysis, MS treatment is a complex process involving multiple systemic pathways, including immunity, infection, and signal transduction, etc. We should focus on candidate drugs that target both the “PI3K-Akt signaling pathway” and the “Chemokine signaling pathway” (e.g., Nemiralisib and Umbralisib) and TKI (e.g., Fostamatinib).

## Data availability statement

The datasets presented in this study can be found in online repositories. The names of the repository/repositories and accession number(s) can be found in the article/**Supplementary Material**.

## Author contributions

XY, XR, XH, CX, and JF: conceptualization. XY, XR, and JF: data curation. XY, XH, YZ, CX, and JF: formal analysis. XY, XR, YZ, and JF: funding acquisition. XY: writing—original draft. XY, XR, CX, and JF: writing—review and editing. All authors contributed to the article and approved the submitted version.

## Funding

This work was supported by the project of Nature Scientific Foundation of Heilongjiang Province (ZD2020H004).

## Conflict of interest

The authors declare that the research was conducted in the absence of any commercial or financial relationships that could be construed as a potential conflict of interest.

## Publisher’s note

All claims expressed in this article are solely those of the authors and do not necessarily represent those of their affiliated organizations, or those of the publisher, the editors and the reviewers. Any product that may be evaluated in this article, or claim that may be made by its manufacturer, is not guaranteed or endorsed by the publisher.

## Supplementary material

Supplementary Table 1 Detailed DEGs obtained from MS patients without treatment according to the type of CD19^+^ B cells, CD4^+^ T cells, pDCs and PBMC.

Supplementary Table 2 Detailed DEGs obtained from MS patients before and after the application of Fingolimod and IFN-β according to the type of CD19^+^ B cells, CD4^+^ T cells, pDCs and PBMC.

Supplementary Table 3 Detailed target genes obtained from MS patients after the application of Fingolimod or IFN-β according to the type of CD19^+^ B cells, CD4^+^ T cells, pDCs and PBMC.

Supplementary Table 4 Detailed pathways obtained from MS patients without treatment according to the type of CD19^+^ B cells, CD4^+^ T cells, pDCs and PBMC.

Supplementary Table 5 Detailed pathways obtained from MS patients before and after the application of Fingolimod and IFN-β according to the type of CD19^+^ B cells, CD4^+^ T cells, pDCs and PBMC.

Supplementary Table 6 Detailed candidate drugs for targeting target pathways obtained from the KEGG database according to the types of CD19^+^ B cells, CD4^+^ T cells, pDCs and PBMC.

Supplementary Table 7 Detailed candidate drugs for targeting 2 target pathways obtained from the KEGG database according to the types of CD19^+^ B cells, CD4^+^ T cells, pDCs and PBMC.

Supplementary Table 8 The genes differentially expressed in CD4^+^ T cells of MS patients without treatment at both mRNA and protein levels.

Supplementary Table 9 \The pathways that were enriched by genes differentially expressed in CD4^+^ T cells of MS patients without treatment at both mRNA and protein levels.

Supplementary Table 10 The DEGs of MS patients and healthy individuals (FC > 2).

Supplementary Table 11 The DEGs of MS patients before and after the application of IFN-β or Fingolimod (FC > 2).

Supplementary Table 12 The target pathways of Fingolimod and IFN-β for MS treatment (FC > 2).

## Abbreviations

MS multiple sclerosis; GEO Gene Expression Omnibus; DEGs differentially expressed genes; CMap connectivity map; KEGG Kyoto Encyclopedia of Genes and Genomes; pDCs plasmacytoid dendritic cells; PBMC peripheral blood mononuclear cells; IFN-β interferon-β; CNS central nervous system; DMTs disease-modifying therapies; BBB blood-brain barrier  BCR B-cell receptor; S1P sphingosine-1 phosphate; ARR annualized relapse rate; RRMS relapsing-remitting multiple sclerosis; FC fold-change; PPI protein-protein interaction; PRIDE; Proteomics Identifications database; EAE experimental allergic encephalomyelitis; Glut1  glucose transporter type 1; OPCs oligodendrocyte precursor cells; OLs oligodendrocytes; PIP3 phosphatidylinositol-3,4,5- trisphosphate; GPCR G protein-coupled receptors; TLR9 toll-like receptor 9; LCFAs long-chain fatty acids; SCFAs short-chain fatty acids; SYK spleen tyrosine kinase; TKI tyrosine kinase inhibitor; ECTRIMS European Committee for Treatment and Research in Multiple Sclerosis; BTKi Bruton’s tyrosine kinase inhibitor; SEL slowly expanding lesion; ITP idiopathic thrombocytopenic purpura.

## References

[1] Yamasaki R Kira J . Multiple sclerosis. Adv Exp Med Biol (2019) 1190:217–47. doi: 10.1007/978-981-32-9636-7_14 31760647

[2] Walton C King R Rechtman L Kaye W Leray E Marrie RA . Rising prevalence of multiple sclerosis worldwide: Insights from the atlas of MS, third edition. Mult Scler (2020) 26:1816–21. doi: 10.1177/1352458520970841 PMC7720355 33174475

[3] Polman CH . Regular review: Drug treatment of multiple sclerosis. BMJ (2000) 321:490–4. doi: 10.1136/bmj.321.7259.490 PMC1118391 10948033

[4] Taskapilioglu O . Recent advances in the treatment for multiple sclerosis; current new drugs specific for multiple sclerosis. Arch Neuropsychiatry (2018) 55(Suppl 1):S15–S20. doi: 10.29399/npa.23402 PMC6278629 30692849

[5] Jahchan NS Dudley JT Mazur PK Flores N Yang D Palmerton A . A drug repositioning approach identifies tricyclic antidepressants as inhibitors of small cell lung cancer and other neuroendocrine tumors. Cancer Discov (2013) 3:1364–77. doi: 10.1158/2159-8290.CD-13-0183 PMC3864571 24078773

[6] Nosengo N . Can you teach old drugs new tricks? Nature (2016) 534:314–6. doi: 10.1038/534314a 27306171

[7] Stinissen P Raus J Zhang J . Autoimmune pathogenesis of multiple sclerosis: Role of autoreactive T lymphocytes and new immunotherapeutic strategies. Crit Rev Immunol (1997) 17:33–75. doi: 10.1615/CritRevImmunol.v17.i1.20 9034723

[8] Kaskow BJ Baecher-Allan C . Effector T cells in multiple sclerosis. Cold Spring Harb Perspect Med (2018) 8:a029025. doi: 10.1101/cshperspect.a029025 29358315 PMC5880159

[9] Hauser SL Waubant E Arnold DL Vollmer T Antel J Fox RJ . B-cell depletion with rituximab in relapsing-remitting multiple sclerosis. N Engl J Med (2008) 358:676–88. doi: 10.1056/NEJMoa0706383 18272891

[10] Wilson HL . B cells contribute to MS pathogenesis through antibody-dependent and antibody-independent mechanisms. Biologics (2012) 6:117–23. doi: 10.2147/BTT.S24734 PMC3363029 22690126

[11] Wanleenuwat P Iwanowski P . Role of b cells and antibodies in multiple sclerosis. Multiple Sclerosis Related Disord (2019) 36:101416. doi: 10.1016/j.msard.2019.101416 31577986

[12] Thewissen K Nuyts AH Deckx N Wijmeersch BV Nagels G D’hooghe M . Circulating dendritic cells of multiple sclerosis patients are proinflammatory and their frequency is correlated with MS-associated genetic risk factors. Mult Scler (2014) 20:548–57. doi: 10.1177/1352458513505352 24057429

[13] Jakimovski D Kolb C Ramanathan M Zivadinov R Weinstock-Guttman B . Interferon β for multiple sclerosis. Cold Spring Harb Perspect Med (2018) 8:a032003. doi: 10.1101/cshperspect.a032003 29311124 PMC6211378

[14] Brinkmann V Davis MD Heise CE Albert R Cottens S Hof R . The immune modulator FTY720 targets sphingosine 1-phosphate receptors. J Biol Chem (2002) 277:21453–7. doi: 10.1074/jbc.C200176200 11967257

[15] Freedman MS Brod S Singer BA Cohen BA Hayward B Dangond F . Clinical and MRI efficacy of Sc IFN β-1a tiw in patients with relapsing MS appearing to transition to secondary progressive MS: *Post hoc* analyses of PRISMS and SPECTRIMS. J Neurol (2020) 267:64–75. doi: 10.1007/s00415-019-09532-5 31559532 PMC6954891

[16] Fox E Vieira MC Johnson K Peeples M Bensimon AG Signorovitch J . Real-world durability of relapse rate reduction in patients with multiple sclerosis receiving fingolimod for up to 3 years: A retrospective US claims database analysis. J Neurological Sci (2019) 398:163–70. doi: 10.1016/j.jns.2019.01.036 30731303

[17] Barrett T Wilhite SE Ledoux P Evangelista C Kim IF Tomashevsky M . NCBI GEO: Archive for functional genomics data sets–update. Nucleic Acids Res (2012) 41:D991–5. doi: 10.1093/nar/gks1193 PMC3531084 23193258

[18] Ritchie ME Phipson B Wu D Hu Y Law CW Shi W . Et al. limma powers differential expression analyses for RNA-sequencing and microarray studies. Nucleic Acids Res (2015) 43:e47–7. doi: 10.1093/nar/gkv007 PMC4402510 25605792

[19] Shannon P Markiel A Ozier O Baliga NS Wang JT Ramage D . Cytoscape: A software environment for integrated models of biomolecular interaction networks. Genome Res (2003) 13:2498–504. doi: 10.1101/gr.1239303 PMC403769 14597658

[20] Lamb J Crawford ED Peck D Modell JW Blat IC Wrobel MJ . The connectivity map: Using gene-expression signatures to connect small molecules, genes, and disease. Science (2006) 313:1929–35. doi: 10.1126/science.1132939 17008526

[21] Wishart DS . DrugBank: A comprehensive resource for in silico drug discovery and exploration. Nucleic Acids Res (2006) 34:D668–72. doi: 10.1093/nar/gkj067 PMC1347430 16381955

[22] Yu G Wang L-G Han Y He Q-Y . ClusterProfiler: An r package for comparing biological themes among gene clusters. OMICS: A J Integr Biol (2012) 16:284–7. doi: 10.1089/omi.2011.0118 PMC3339379 22455463

[23] Kanehisa M Goto S Furumichi M Tanabe M Hirakawa M . KEGG for representation and analysis of molecular networks involving diseases and drugs. Nucleic Acids Res (2010) 38:D355–60. doi: 10.1093/nar/gkp896 PMC2808910 19880382

[24] Martens L Hermjakob H Jones P Adamski M Taylor C States D . PRIDE: The proteomics identifications database. Proteomics (2005) 5:3537–45. doi: 10.1002/pmic.200401303 16041671

[25] Henderson J . Google Scholar: A source for clinicians? CMAJ (2005) 172:1549–50. doi: 10.1503/cmaj.050404 PMC558163 15939908

[26] Berge T Eriksson A Brorson IS Høgestøl EA Berg-Hansen P Døskeland A . Quantitative proteomic analyses of CD4+ and CD8+ T cells reveal differentially expressed proteins in multiple sclerosis patients and healthy controls. Clin Proteomics (2019) 16:19. doi: 10.1186/s12014-019-9241-5 31080378 PMC6505067

[27] Cappelletti C Eriksson A Brorson IS Leikfoss IS Kråbøl O Høgestøl EA . Quantitative proteomics reveals protein dysregulation during T cell activation in multiple sclerosis patients compared to healthy controls. Clin Proteomics (2022) 19:23. doi: 10.1186/s12014-022-09361-1 35790914 PMC9254507

[28] Annibali V Umeton R Palermo A Severa M Etna MP Giglio S . Analysis of coding and non-coding transcriptome of peripheral b cells reveals an altered interferon response factor (IRF)-1 pathway in multiple sclerosis patients. J Neuroimmunology (2018) 324:165–71. doi: 10.1016/j.jneuroim.2018.09.005 30270021

[29] Salehi Z Talebi S Maleknia S Palizban F Naser Moghadasi A Kavousi K . RNA Sequencing of CD4+ T cells in relapsing–remitting multiple sclerosis patients at relapse: Deciphering the involvement of novel genes and pathways. J Mol Neurosci (2021) 71:2628–45. doi: 10.1007/s12031-021-01878-8 34286457

[30] Aung LL Brooks A Greenberg SA Rosenberg ML Dhib-Jalbut S Balashov KE . Multiple sclerosis-linked and interferon-Beta-Regulated gene expression in plasmacytoid dendritic cells. J Neuroimmunology (2012) 250:99–105. doi: 10.1016/j.jneuroim.2012.05.013 22688425 PMC3418446

[31] Irizar H Muñoz-Culla M Sepúlveda L Sáenz-Cuesta M Prada Á. Castillo-Triviño T . Transcriptomic profile reveals gender-specific molecular mechanisms driving multiple sclerosis progression. PloS One (2014) 9:e90482. doi: 10.1371/journal.pone.0090482 24587374 PMC3938749

[32] Angerer IC Hecker M Koczan D Roch L Friess J Rüge A . Transcriptome profiling of peripheral blood immune cell populations in multiple sclerosis patients before and during treatment with a sphingosine-1-Phosphate receptor modulator. CNS Neurosci Ther (2018) 24:193–201. doi: 10.1111/cns.12793 29314605 PMC6490155

[33] Koczan D Fitzner B Zettl UK Hecker M . Microarray data of transcriptome shifts in blood cell subsets during S1P receptor modulator therapy. Sci Data (2018) 5:180145. doi: 10.1038/sdata.2018.145 30040082 PMC6057441

[34] Friess J Hecker M Roch L Koczan D Fitzner B Angerer IC . Fingolimod alters the transcriptome profile of circulating CD4+ cells in multiple sclerosis. Sci Rep (2017) 7:42087. doi: 10.1038/srep42087 28155899 PMC5290459

[35] Hecker M Hartmann C Kandulski O Paap BK Koczan D Thiesen H-J . Interferon-beta therapy in multiple sclerosis: The short-term and long-term effects on the patients’ individual gene expression in peripheral blood. Mol Neurobiol (2013) 48:737–56. doi: 10.1007/s12035-013-8463-1 23636981

[36] Hundeshagen A Hecker M Paap BK Angerstein C Kandulski O Fatum C . Elevated type I interferon-like activity in a subset of multiple sclerosis patients: Molecular basis and clinical relevance. J Neuroinflamm (2012) 9:574. doi: 10.1186/1742-2094-9-140 PMC3464734 22727118

[37] Ye H Yang L Cao Z Tang K Li YA . Pathway profile-based method for drug repositioning. Chin Sci Bull (2012) 57:2106–12. doi: 10.1007/s11434-012-4982-9

[38] Constantinescu CS Gran B . Multiple sclerosis: Autoimmune associations in multiple sclerosis. Nat Rev Neurol (2010) 6:591–2. doi: 10.1038/nrneurol.2010.147 21048800

[39] Puri KD Doggett TA Douangpanya J Hou Y Tino WT Wilson T . Mechanisms and implications of phosphoinositide 3-kinase δ in promoting neutrophil trafficking into inflamed tissue. Blood (2004) 103:3448–56. doi: 10.1182/blood-2003-05-1667 14751923

[40] Haylock-Jacobs S Comerford I Bunting M Kara E Townley S Klingler-Hoffmann M . PI3Kδ drives the pathogenesis of experimental autoimmune encephalomyelitis by inhibiting effector T cell apoptosis and promoting Th17 differentiation. J Autoimmun (2011) 36:278–87. doi: 10.1016/j.jaut.2011.02.006 21396797

[41] da Silva LC Lima IVdeA da Silva MCM Corrêa TA de Souza VP de Almeida MV . A new lipophilic amino alcohol, chemically similar to compound FTY720, attenuates the pathogenesis of experimental autoimmune encephalomyelitis by PI3K/Akt pathway inhibition. Int Immunopharmacol (2020) 88:106919. doi: 10.1016/j.intimp.2020.106919 32871475

[42] Kunkl M Sambucci M Ruggieri S Amormino C Tortorella C Gasperini C . CD28 autonomous signaling up-regulates c-myc expression and promotes glycolysis enabling inflammatory T cell responses in multiple sclerosis. Cells (2019) 8:E575. doi: 10.3390/cells8060575 31212712 PMC6628233

[43] Laudanna C Alon R . Right on the spot. chemokine triggering of integrin-mediated arrest of rolling leukocytes. Thromb Haemost (2006) 95:5–11. doi: 10.1160/TH05-07-0482 16543955

[44] Chu T Shields LBE Zhang YP Feng S-Q Shields CB Cai J . CXCL12/CXCR4/CXCR7 chemokine axis in the central nervous system: Therapeutic targets for remyelination in demyelinating diseases. Neuroscientist (2017) 23:627–48. doi: 10.1177/1073858416685690 29283028

[45] Aranami T Yamamura T . Th17 cells and autoimmune encephalomyelitis (EAE/MS). Allergology Int (2008) 57:115–20. doi: 10.2332/allergolint.R-07-159 18427164

[46] Sun J Zhou H Bai F Zhang Z Ren Q . Remyelination: A potential therapeutic strategy for alzheimer’s disease? JAD (2017) 58:597–612. doi: 10.3233/JAD-170036 28453483

[47] Martinez LA . Mutant P53 and ETS2, a tale of reciprocity. Front Oncol (2016) 6:35. doi: 10.3389/fonc.2016.00035 26925389 PMC4757666

[48] Schaefer T Steiner R Lengerke C . SOX2 and P53 expression control converges in PI3K/AKT signaling with versatile implications for stemness and cancer. IJMS (2020) 21:4902. doi: 10.3390/ijms21144902 PMC7402325 32664542

[49] Okuda Y Okuda M Bernard CCA . Regulatory role of P53 in experimental autoimmune encephalomyelitis. J Neuroimmunology (2003) 135:29–37. doi: 10.1016/S0165-5728(02)00428-9 12576221

[50] Vanhaesebroeck B Stephens L Hawkins P . PI3K signalling: The path to discovery and understanding. Nat Rev Mol Cell Biol (2012) 13:195–203. doi: 10.1038/nrm3290 22358332

[51] Kim HS Jang SW Lee W Kim K Sohn H Hwang SS . PTEN drives Th17 cell differentiation by preventing IL-2 production. J Exp Med (2017) 214:3381–98. doi: 10.1084/jem.20170523 PMC5679178 29018045

[52] Bandres-Ciga S Diez-Fairen M Kim JJ Singleton AB . Genetics of parkinson’s disease: An introspection of its journey towards precision medicine. Neurobiol Dis (2020) 137:104782. doi: 10.1016/j.nbd.2020.104782 31991247 PMC7064061

[53] Walsh JH . The brain-gut axis: A new frontier. Proceedings of an international symposium held in Florence Italy, June 29-July 1, 1981. Peptides (1981) 2 Suppl 2:1–299. doi: 10.1016/0196-9781(82)90151-6 7343957

[54] Shen X Sun Z . Microbe-gut-brain axis and neurological disorders: A review. Sheng Wu Gong Cheng Xue Bao (2021) 37:3781–8. doi: 10.13345/j.cjb.200773 34841783

[55] Montiel-Castro AJ González-Cervantes RM Bravo-Ruiseco G Pacheco-López G . The microbiota-Gut-Brain axis: Neurobehavioral correlates, health and sociality. Front Integr Neurosci (2013) 7:70. doi: 10.3389/fnint.2013.00070 24109440 PMC3791857

[56] Lundell A-C Björnsson V Ljung A Ceder M Johansen S Lindhagen G . Infant b cell memory differentiation and early gut bacterial colonization. J.I. (2012) 188:4315–22. doi: 10.4049/jimmunol.1103223 22490441

[57] Crespo A . De M.C.; falcão, D.P.; Ferreira de araújo, P.M.; machado de medeiros, B.M. effects of yersinia enterocolitica O:3 derivatives on b lymphocyte activation in vivo. Microbiol Immunol (2002) 46:95–100. doi: 10.1111/j.1348-0421.2002.tb02664.x 11939584

[58] Balashov KE Aung LL Vaknin-Dembinsky A Dhib-Jalbut S Weiner HL . Interferon-β inhibits toll-like receptor 9 processing in multiple sclerosis. Ann Neurol (2010) 68:899–906. doi: 10.1002/ana.22136 21061396 PMC3058378

[59] Meier U-C Cipian RC Karimi A Ramasamy R Middeldorp JM . Cumulative roles for Epstein-Barr virus, human endogenous retroviruses, and human herpes virus-6 in driving an inflammatory cascade underlying MS pathogenesis. Front Immunol (2021) 12:757302. doi: 10.3389/fimmu.2021.757302 34790199 PMC8592026

[60] Sgarbanti M Marsili G Remoli AL Orsatti R Battistini A . IRF-7: New role in the regulation of genes involved in adaptive immunity. Ann N Y Acad Sci (2007) 1095:325–33. doi: 10.1196/annals.1397.036 17404045

[61] Salem M Mony JT Løbner M Khorooshi R Owens T . Interferon regulatory factor-7 modulates experimental autoimmune encephalomyelitis in mice. J Neuroinflamm (2011) 8:181. doi: 10.1186/1742-2094-8-181 PMC3260126 22196084

[62] Haghikia A Jörg S Duscha A Berg J Manzel A Waschbisch A . Dietary fatty acids directly impact central nervous system autoimmunity. via Small Intestine. Immun (2015) 43:817–29. doi: 10.1016/j.immuni.2015.09.007 26488817

[63] Díaz ME González L Miquet JG Martínez CS Sotelo AI Bartke A . Growth hormone modulation of EGF-induced PI3K-akt pathway in mice liver. Cell Signal (2012) 24:514–23. doi: 10.1016/j.cellsig.2011.10.001 PMC3616332 22019461

[64] Montalban X Arnold DL Weber MS Staikov I Piasecka-Stryczynska K Willmer J . Placebo-controlled trial of an oral BTK inhibitor in multiple sclerosis. N Engl J Med (2019) 380:2406–17. doi: 10.1056/NEJMoa1901981 31075187

[65] Pochon S . 37th congress of the European committee for treatment and research in multiple sclerosis (ECTRIMS 2021): 13–15 October, 2021. Pharm Med (2021) 35:367–70. doi: 10.1007/s40290-021-00411-x 34855157

[66] Markham A . Fostamatinib: First global approval. Drugs (2018) 78:959–63. doi: 10.1007/s40265-018-0927-1 29869203

[67] Scott IC Scott DL . Spleen tyrosine kinase inhibitors for rheumatoid arthritis: Where are we now? Drugs (2014) 74:415–22. doi: 10.1007/s40265-014-0193-9 24610702

[68] Khindri S Cahn A Begg M Montembault M Leemereise C Cui Y . A multicentre, randomized, double-blind, placebo-controlled, crossover study to investigate the efficacy, safety, tolerability, and pharmacokinetics of repeat doses of inhaled nemiralisib in adults with persistent, uncontrolled asthma. J Pharmacol Exp Ther (2018) 367:405–13. doi: 10.1124/jpet.118.249516 30217958

[69] Dhillon S Keam SJ . Umbralisib: First approval. Drugs (2021) 81:857–66. doi: 10.1007/s40265-021-01504-2 33797740

