# Supplementary material for: Immune cells transcriptome-based drug repositioning for multiple sclerosis
Source: Front Immunol. 2022 Oct 20;13:1020721. doi: 10.3389/fimmu.2022.1020721 (PMC9630342; doi:10.3389/fimmu.2022.1020721)
Supplement: Supplementary Table 1 — Detailed DEGs obtained from MS patients without treatment according to the type of CD19+ B cells, CD4+ T cells, pDCs and PBMC. [file Table_1.docx]

| Sample | DEG | Up/Down |
| --- | --- | --- |
| CD19^+^ B cells | SLC38A11 | up |
|  | GK3P |  |
|  | EAF2 |  |
|  | SUMO1P3 |  |
|  | NDUFA4 |  |
|  | SLC15A2 |  |
|  | P2RY14 |  |
|  | FCRL1 |  |
|  | FARS2 |  |
|  | KRTAP4-12 |  |
|  | PHOSPHO2 |  |
|  | VAV3 |  |
|  | FAM133B |  |
|  | KMO |  |
|  | COL19A1 |  |
|  | HIST1H3C |  |
|  | LOC100128252 |  |
|  | STAP1 |  |
|  | NCRNA00265 |  |
|  | NCRNA00189 |  |
|  | CAMK2D |  |
|  | SCN3A |  |
|  | MACF1 |  |
|  | GAPT |  |
|  | FCHSD2 |  |
|  | CT45A5 |  |
|  | IL4R |  |
|  | MAK |  |
|  | NT5E |  |
|  | IGJ |  |
|  | PPAPDC1B |  |
|  | KPNA2 |  |
|  | FCRL2 |  |
|  | NSUN7 |  |
|  | C4orf34 |  |
|  | SDHC |  |
|  | TPST1 |  |
|  | LYST |  |
|  | SSBP3 |  |
|  | TP53INP1 |  |
|  | AKD1 |  |
|  | ATP10D |  |
|  | MME |  |
|  | CD38 |  |
|  | RPSAP58 |  |
|  | TBC1D5 |  |
|  | SESN1 |  |
|  | 3-Mar |  |
|  | KCNH8 |  |
|  | GCA |  |
|  | YEATS4 |  |
|  | PEG10 |  |
|  | TRA2A |  |
|  | ADK |  |
|  | OCR1 |  |
|  | SKP1 |  |
|  | TAF9 |  |
|  | IL6 |  |
|  | TXNDC5 |  |
|  | ALS2CR12 |  |
|  | ARHGAP32 |  |
|  | TAPT1 |  |
|  | CR2 |  |
|  | AFF3 |  |
|  | ADARB1 |  |
|  | POM121 |  |
|  | TMEM156 |  |
|  | CR1 |  |
|  | BMP2K |  |
|  | C6orf115 |  |
|  | AHSA2 |  |
|  | EEF1E1 |  |
|  | ATG4C |  |
|  | RUNDC2A |  |
|  | CEP135 |  |
|  | PIGH |  |
|  | SSBP2 |  |
|  | ZNF318 |  |
|  | SAMD9L |  |
|  | ITPR1 |  |
|  | RASGRP3 |  |
|  | SPRY1 |  |
|  | FCER2 |  |
|  | SKAP2 |  |
|  | GAB1 |  |
|  | IL1RAP |  |
|  | KIAA1407 |  |
|  | LOC100130428 |  |
|  | SATB1 |  |
|  | DZIP3 |  |
|  | TBC1D19 |  |
|  | THAP5 |  |
|  | C11orf80 |  |
|  | CLK1 |  |
|  | RNASE6 |  |
|  | MGC39372 |  |
|  | PNOC |  |
|  | BRP44 |  |
|  | EIF1B |  |
|  | EIF2AK3 |  |
|  | COMMD10 |  |
|  | CHML |  |
|  | PIGF |  |
|  | SESTD1 |  |
|  | N4BP2 |  |
|  | EZH2 |  |
|  | WDR19 |  |
|  | SLC9A9 |  |
|  | PIK3CA |  |
|  | RABGAP1L |  |
|  | KBTBD2 |  |
|  | ZNF382 |  |
|  | MMADHC |  |
|  | SH3BP5 |  |
|  | LRBA |  |
|  | ABCB4 |  |
|  | EFCAB2 |  |
|  | C18orf45 |  |
|  | AP4B1 |  |
|  | SASS6 |  |
|  | DYNC1I2 |  |
|  | TAGAP |  |
|  | AFF1 |  |
|  | RPL5 |  |
|  | C2orf67 |  |
|  | ACBD7 |  |
|  | LRRC37A3 |  |
|  | IFT80 |  |
|  | CLDN12 |  |
|  | KHDRBS2 |  |
|  | CHRM3 |  |
|  | TIGD1 |  |
|  | TSGA10 |  |
|  | C1orf26 |  |
|  | EPB41L2 |  |
|  | C1orf27 |  |
|  | WDR35 |  |
|  | RPL31 |  |
|  | ARL15 |  |
|  | NDC80 |  |
|  | EVI5 |  |
|  | IFRD1 |  |
|  | NFYA |  |
|  | PPIL4 |  |
|  | PTK2B |  |
|  | KLHL24 |  |
|  | THADA |  |
|  | CCDC146 |  |
|  | NBEAL1 |  |
|  | AKIRIN2 |  |
|  | RNFT1 |  |
|  | TRNT1 |  |
|  | POTEM |  |
|  | KLHL2 |  |
|  | C6orf72 |  |
|  | SNX9 |  |
|  | MRPS18C |  |
|  | ARID4B |  |
|  | C6orf125 |  |
|  | THEM4 |  |
|  | CYB5R4 |  |
|  | EFCAB7 |  |
|  | SLC14A1 |  |
|  | EHBP1 |  |
|  | NIPSNAP3B |  |
|  | IFNGR1 |  |
|  | SENP2 |  |
|  | C5orf44 |  |
|  | TEC |  |
|  | RBM8A |  |
|  | GALNT3 |  |
|  | SYF2 |  |
|  | ZNF512 |  |
|  | MLH1 |  |
|  | RIOK1 |  |
|  | UNC119B |  |
|  | ACAT2 |  |
|  | CUL3 |  |
|  | MYO1B |  |
|  | ATL2 |  |
|  | TMF1 |  |
|  | ELL2 |  |
|  | MGC29506 |  |
|  | FLJ14107 |  |
|  | ANO10 |  |
|  | MDM4 |  |
|  | ZBTB11 |  |
|  | BNIP1 |  |
|  | GABBR1 |  |
|  | DLD |  |
|  | BTN2A2 |  |
|  | USP40 |  |
|  | ZRSR2 |  |
|  | U2AF1 |  |
|  | SP100 |  |
|  | PFKFB2 |  |
|  | PTBP2 |  |
|  | DOCK8 |  |
|  | CCDC28A |  |
|  | PAQR3 |  |
|  | C12orf51 |  |
|  | ZNF326 |  |
|  | KCNIP2 |  |
|  | DCAF16 |  |
|  | C2orf43 |  |
|  | CASD1 |  |
|  | FGGY |  |
|  | HACE1 |  |
|  | WASF1 |  |
|  | T1560 |  |
|  | OGFRL1 |  |
|  | PPID |  |
|  | SFRS18 |  |
|  | SNUPN |  |
|  | UGT8 |  |
|  | RNF7 |  |
|  | C20orf79 | down |
|  | GNG8 |  |
|  | MYF6 |  |
|  | SOCS3 |  |
|  | RAB34 |  |
|  | SCG2 |  |
|  | UBE2L6 |  |
|  | C2CD4A |  |
|  | OR1L1 |  |
|  | STAT3 |  |
|  | TBC1D3P2 |  |
|  | GNGT2 |  |
|  | RPS19 |  |
|  | LACTB |  |
|  | DSCR10 |  |
|  | IVL |  |
|  | GATA3 |  |
|  | ATP5H |  |
|  | GAGE12G |  |
|  | TSHZ3 |  |
|  | SPANXB1 |  |
|  | GABARAPL3 |  |
|  | RCSD1 |  |
|  | CAMK2A |  |
|  | CCL7 |  |
|  | IL10RA |  |
|  | PGA4 |  |
|  | C20orf144 |  |
|  | MPZL2 |  |
|  | FAM18B1 |  |
|  | TFDP3 |  |
|  | DEFB118 |  |
|  | DDX53 |  |
|  | SPINT4 |  |
|  | RAB33A |  |
|  | ECEL1P2 |  |
|  | PUS3 |  |
|  | ATF5 |  |
|  | ZBTB34 |  |
|  | LAGE3 |  |
|  | LOC286359 |  |
|  | PRR4 |  |
|  | NLRC5 |  |
|  | CYP1B1 |  |
|  | MRGPRX1 |  |
|  | TOX |  |
|  | OR8S1 |  |
|  | MTFP1 |  |
|  | SUGT1P1 |  |
|  | KRTAP5-3 |  |
|  | OR1L3 |  |
|  | POLR3G |  |
|  | OR2V2 |  |
|  | HSPE1 |  |
|  | AKAP6 |  |
|  | PIPSL |  |
|  | ZBTB6 |  |
|  | PACS2 |  |
|  | CD247 |  |
|  | APOC1P1 |  |
|  | KHK |  |
|  | KRTAP21-1 |  |
|  | OR2K2 |  |
|  | LOC100128751 |  |
|  | TAS2R31 |  |
|  | IRF1 |  |
|  | S100A7 |  |
|  | FAM182A |  |
|  | LCE1D |  |
|  | KIR2DL2 |  |
|  | CXCL9 |  |
|  | ANP32D |  |
|  | WARS |  |
|  | RPSA |  |
|  | AKR1C1 |  |
|  | STAT1 |  |
|  | CXCL10 |  |
| CD4^+^ T cells | HBG1 | up |
|  | HEATR6 |  |
|  | WDR18 |  |
|  | PPARGC1B |  |
|  | WHAMMP2 |  |
|  | GPATCH1 |  |
|  | TMEM45B |  |
|  | TMEM176A |  |
|  | ZNF816 |  |
|  | TAPBP |  |
|  | DHX35 |  |
|  | RIT1 |  |
|  | TRPC1 |  |
|  | CCDC17 |  |
|  | FCRLA |  |
|  | ZNF263 |  |
|  | ZNF829 |  |
|  | CCNI2 |  |
|  | LRFN1 |  |
|  | LOC105379521 |  |
|  | MBIP |  |
|  | TAF8 |  |
|  | MED14OS |  |
|  | MBLAC1 |  |
|  | LOC105369441 |  |
|  | URGCP |  |
|  | TES |  |
|  | LOC105373085 |  |
|  | SUSD4 |  |
|  | LOC105373488 |  |
|  | APBA2 |  |
|  | RNF6 |  |
|  | TAF4B |  |
|  | LOC105375674 |  |
|  | OSTF1 |  |
|  | GTPBP6 |  |
|  | LOC105375511 |  |
|  | IL17RC |  |
|  | PICK1 |  |
|  | MTCH2 |  |
|  | LOC100505915 |  |
|  | TRIM16L |  |
|  | VIPAS39 |  |
|  | SLC6A6 |  |
|  | EIF2B5 |  |
|  | ACER1 |  |
|  | CDKL2 |  |
|  | NAE1 |  |
|  | GABPA |  |
|  | H3C11 |  |
|  | LOC107985364 |  |
|  | RPS16 |  |
|  | NECAP2 |  |
|  | H3C3 |  |
|  | MIR6728 |  |
|  | MHENCR |  |
|  | CARNMT1 |  |
|  | H2AC7 |  |
|  | ALG8 |  |
|  | CD79B |  |
|  | H2AC16 |  |
|  | TSHZ2 |  |
|  | UTP18 |  |
|  | H2AC4 |  |
|  | GIHCG |  |
|  | MT2A |  |
|  | H2AC11 |  |
|  | ISCU |  |
|  | RPL15 |  |
|  | H2BC7 |  |
|  | HDGFL3 |  |
|  | MLH1 |  |
|  | H2BC10 |  |
|  | OSBPL8 |  |
|  | H2AC13 |  |
|  | USP18 |  |
|  | BEX2 |  |
|  | CHD4 |  |
|  | H3C15 |  |
|  | H2AC12 |  |
|  | H3C14 |  |
|  | RPL17 |  |
|  | RPL41 |  |
|  | H3C7 |  |
|  | H4C13 |  |
|  | H2BC11 |  |
|  | EEF1D |  |
|  | H3C13 |  |
|  | RPSAP9 |  |
|  | H2AC8 |  |
|  | H4C4 |  |
|  | H3C10 |  |
|  | H2BC6 |  |
|  | SCARNA2 |  |
|  | GADD45GIP1 |  |
|  | RPL35 |  |
|  | SLA |  |
|  | H4C2 |  |
|  | H4C6 |  |
|  | H2AC21 |  |
|  | H4C3 |  |
|  | RPS5 |  |
|  | H4C14 |  |
|  | H4C15 |  |
|  | CCR7 |  |
|  | RPS7 |  |
|  | CIAO2A |  |
|  | H2AC6 |  |
|  | RPL9 |  |
|  | RPL27 |  |
|  | RPS27 |  |
|  | RPL21 |  |
|  | RPS29 |  |
|  | RPS25 |  |
|  | RPL36 |  |
|  | RPS15A |  |
|  | RPL35A |  |
|  | RPL13A |  |
|  | RPL13 |  |
|  | RPL30 |  |
|  | RPS14 |  |
|  | RPL18 |  |
|  | RN7SK |  |
|  | RPL23 |  |
|  | RPS21 |  |
|  | RPSA |  |
|  | RPS4X |  |
|  | RPL18A |  |
|  | H4C5 |  |
|  | RPS3A |  |
|  | RPL29 |  |
|  | RPL24 |  |
|  | SCARNA21 |  |
|  | RPS15 |  |
|  | RPL37 |  |
|  | RPLP0 |  |
|  | RPS20 |  |
|  | FAU |  |
|  | RPS10 |  |
|  | RPL37A |  |
|  | RPS18 |  |
|  | RPS6 |  |
|  | RPL11 |  |
|  | RPL10A |  |
|  | RPS11 |  |
|  | RPS2 |  |
|  | RPL23A |  |
|  | RPL36A |  |
|  | RPL39 |  |
|  | RPS27A |  |
|  | RPS8 |  |
|  | RPL14 |  |
|  | RPL19 |  |
|  | RPS12 |  |
|  | RPL5 |  |
|  | RPL4 |  |
|  | GAS5 |  |
|  | RPL27A |  |
|  | COX7C |  |
|  | RPS23 |  |
|  | RPL7 |  |
|  | SCARNA7 |  |
|  | TMSB10 |  |
|  | SERINC1 | down |
|  | GNAI3 |  |
|  | PPP1CC |  |
|  | PJA2 |  |
|  | IPO7 |  |
|  | TMED10 |  |
|  | SMC1A |  |
|  | UBL3 |  |
|  | SEC23A |  |
|  | IER3IP1 |  |
|  | CAPN2 |  |
|  | LRP10 |  |
|  | NORAD |  |
|  | DNAJB9 |  |
|  | RAB10 |  |
|  | BMI1 |  |
|  | SRP9 |  |
|  | KCNA3 |  |
|  | ADSS2 |  |
|  | SERP1 |  |
|  | HINT3 |  |
|  | ITM2B |  |
|  | HEXIM1 |  |
|  | IQGAP1 |  |
|  | ARFGEF2 |  |
|  | H3P6 |  |
|  | TMEM43 |  |
|  | GALNT1 |  |
|  | MYH9 |  |
|  | BMT2 |  |
|  | NIBAN1 |  |
|  | GMPS |  |
|  | NIBAN2 |  |
|  | ZMPSTE24 |  |
|  | YWHAQ |  |
|  | UBXN4 |  |
|  | PPP2R5A |  |
|  | CMPK1 |  |
|  | TMX4 |  |
|  | ITGA5 |  |
|  | ITSN2 |  |
|  | AUP1 |  |
|  | SCYL2 |  |
|  | LOC401261 |  |
|  | SLC25A36 |  |
|  | STXBP3 |  |
|  | PRKX |  |
|  | NUS1 |  |
|  | APOL6 |  |
|  | ARF6 |  |
|  | RNF139 |  |
|  | FEM1B |  |
|  | CHMP4B |  |
|  | AFTPH |  |
|  | SGPP1 |  |
|  | ABHD13 |  |
|  | RAP1B |  |
|  | SRP72 |  |
|  | RAB2A |  |
|  | UHMK1 |  |
|  | WDFY1 |  |
|  | ZBTB33 |  |
|  | SLC25A46 |  |
|  | LOC100289230 |  |
|  | EIF1AX |  |
|  | GALNT4 |  |
|  | ACOT13 |  |
|  | TFAM |  |
|  | LCP2 |  |
|  | UBP1 |  |
|  | AP5Z1 |  |
|  | ZFC3H1 |  |
|  | SLC7A5P2 |  |
|  | DNMT1 |  |
|  | USP34 |  |
|  | KIF5B |  |
|  | FUCA2 |  |
|  | TIMM10B |  |
|  | PDP1 |  |
|  | NADSYN1 |  |
|  | NFE2L3 |  |
|  | SRSF4 |  |
|  | SPTY2D1 |  |
|  | GPAT4 |  |
|  | HELZ2 |  |
|  | GNPAT |  |
|  | CIAO1 |  |
|  | TXNDC15 |  |
|  | SPEN |  |
|  | RAB11FIP4 |  |
|  | LMBRD1 |  |
|  | SLAIN2 |  |
|  | PSEN1 |  |
|  | PUM1 |  |
|  | NPEPPS |  |
|  | HNRNPUL2 |  |
|  | RAB18 |  |
|  | DHX36 |  |
|  | TLN1 |  |
|  | KDM7A |  |
|  | PIP4P2 |  |
|  | LYSMD3 |  |
|  | MAP1LC3B |  |
|  | ARG2 |  |
|  | MAT2A |  |
|  | ZDHHC18 |  |
|  | APPL1 |  |
|  | ITGA4 |  |
|  | DCTN2 |  |
|  | SRPK1 |  |
|  | YIPF6 |  |
|  | ATP1B3 |  |
|  | DNM2 |  |
|  | VCL |  |
|  | CNIH1 |  |
|  | CPD |  |
|  | AMY2B |  |
|  | CCDC6 |  |
|  | GOLM1 |  |
|  | KPNA4 |  |
|  | SPCS3 |  |
|  | PDCD6 |  |
|  | TMEM106B |  |
|  | ACTR2 |  |
|  | EFCAB14 |  |
|  | SGK3 |  |
|  | SPPL2A |  |
|  | ZNF148 |  |
|  | PRDM4 |  |
|  | HS2ST1 |  |
|  | PPM1D |  |
|  | GOLPH3 |  |
|  | TRIM25 |  |
|  | GTF2IP4 |  |
|  | TMEM64 |  |
|  | FAM91A1 |  |
|  | DHX15 |  |
|  | HSP90B1 |  |
|  | PDK3 |  |
|  | EXOC5 |  |
|  | SELENOI |  |
|  | SLC25A29 |  |
|  | MAP3K2 |  |
|  | DIAPH2 |  |
|  | EPM2AIP1 |  |
|  | RNMT |  |
|  | NUDT4P2 |  |
|  | LCOR |  |
|  | KIF20B |  |
|  | PPTC7 |  |
|  | GNL3L |  |
|  | PTEN |  |
|  | CCSAP |  |
|  | ZNRF2 |  |
|  | PHIP |  |
|  | VPS13D |  |
|  | ACO2 |  |
|  | STX12 |  |
|  | FAM98B |  |
|  | NUFIP2 |  |
|  | RSBN1 |  |
|  | ATP6V1C1 |  |
|  | CD47 |  |
|  | IGF2R |  |
|  | NUDT21 |  |
|  | EBLN3P |  |
|  | EEA1 |  |
|  | SLK |  |
|  | CDC73 |  |
|  | NOTCH2NLA |  |
|  | RAB22A |  |
|  | KIF27 |  |
|  | ZNF37BP |  |
|  | STRN |  |
|  | PDCD7 |  |
|  | HECA |  |
|  | RRN3P2 |  |
|  | RAB6A |  |
|  | ERBIN |  |
|  | MED21 |  |
|  | STK10 |  |
|  | SRA1 |  |
|  | SLC31A1 |  |
|  | SGTB |  |
|  | KPNA6 |  |
|  | IFNAR1 |  |
|  | RNF103 |  |
|  | TMEM30A |  |
|  | TYMP |  |
|  | B4GALT5 |  |
|  | SF3A1 |  |
|  | NDFIP1 |  |
|  | LIMS1 |  |
|  | COG5 |  |
|  | USF3 |  |
|  | PHLPP2 |  |
|  | GNB2 |  |
|  | PABPC1 |  |
|  | LOC728392 |  |
|  | SRPRA |  |
|  | EIF3J |  |
|  | PRKCB |  |
|  | TRIQK |  |
|  | RYBP |  |
|  | GCLC |  |
|  | TRIM44 |  |
|  | SMIM14 |  |
|  | RAD50 |  |
|  | TMEM167A |  |
|  | SSR1 |  |
|  | GOLM2 |  |
|  | KRCC1 |  |
|  | ZNF354B |  |
|  | C16orf72 |  |
|  | SFT2D2 |  |
|  | RAB11A |  |
|  | AP1G1 |  |
|  | YY1 |  |
|  | OTUD1 |  |
|  | SNX3 |  |
|  | DENND10P1 |  |
|  | ZBED4 |  |
|  | YWHAG |  |
|  | STIMATE |  |
|  | RAB37 |  |
|  | EP300 |  |
|  | KPNA3 |  |
|  | CD164 |  |
|  | STX5 |  |
|  | PURB |  |
|  | EIF2AK1 |  |
|  | VCPIP1 |  |
|  | RAP2A |  |
|  | NR1H2 |  |
|  | KIF1B |  |
|  | AGTPBP1 |  |
|  | KHDC4 |  |
|  | HOOK3 |  |
|  | CXXC1 |  |
|  | LATS2 |  |
|  | CHSY1 |  |
|  | CACUL1 |  |
|  | ITPR3 |  |
|  | RPL23AP53 |  |
|  | RNPC3 |  |
|  | DDI2 |  |
|  | SNX18 |  |
|  | TTL |  |
|  | PCNX4 |  |
|  | NAA30 |  |
|  | RAD21 |  |
|  | ABHD2 |  |
|  | ERP44 |  |
|  | BACH1 |  |
|  | LOC107986160 |  |
|  | BNIP2 |  |
|  | LRIF1 |  |
|  | ASTL |  |
|  | OSBPL9 |  |
|  | LYST |  |
|  | ERN1 |  |
|  | LAMP2 |  |
|  | TMED7 |  |
|  | KPNB1 |  |
|  | SRCAP |  |
|  | RBAK |  |
|  | EDC4 |  |
|  | KLF13 |  |
|  | SCAF8 |  |
|  | MANEA |  |
|  | SCARB2 |  |
|  | SP100 |  |
|  | MIR3064 |  |
|  | LRRC58 |  |
|  | DLST |  |
|  | UBE2K |  |
|  | NCEH1 |  |
|  | ADAM8 |  |
|  | SMG1P1 |  |
|  | RRAGC |  |
|  | PREX1 |  |
|  | DNAAF9 |  |
|  | SP1 |  |
|  | RAB12 |  |
|  | NHLRC3 |  |
|  | STRAP |  |
|  | TTPAL |  |
|  | BMS1P1 |  |
|  | PER1 |  |
|  | PLEKHA3 |  |
|  | B3GALT2 |  |
|  | PGRMC2 |  |
|  | DSTN |  |
|  | RAB11FIP2 |  |
|  | PAPOLA |  |
|  | DOCK7 |  |
|  | MOSPD2 |  |
|  | BPNT2 |  |
|  | CLK3 |  |
|  | FAM8A1 |  |
|  | IRF1 |  |
|  | CNN2 |  |
|  | RBBP4 |  |
|  | MBD4 |  |
|  | TBC1D25 |  |
|  | FAM102B |  |
|  | MYO9B |  |
|  | USP13 |  |
|  | DUSP10 |  |
|  | RNF125 |  |
|  | MED12 |  |
|  | KLHL18 |  |
|  | TAOK1 |  |
|  | NIPBL |  |
|  | NCOA1 |  |
|  | PRDM1 |  |
|  | SNTB1 |  |
|  | SESN2 |  |
|  | LOC107984658 |  |
|  | SMCHD1 |  |
|  | ERO1B |  |
|  | ATXN7 |  |
|  | SLC25A40 |  |
|  | SLC30A1 |  |
|  | GOLT1B |  |
|  | ATP6V1A |  |
|  | ECI2 |  |
|  | NT5DC1 |  |
|  | ITGAM |  |
|  | CA2 |  |
|  | QSOX1 |  |
|  | CCNT2 |  |
|  | SPN |  |
|  | FOXK2 |  |
|  | RAVER2 |  |
|  | NEDD4 |  |
|  | MAP3K1 |  |
|  | TRIM56 |  |
|  | RALGAPA1P1 |  |
|  | TMX3 |  |
|  | MAML1 |  |
|  | KCTD10 |  |
|  | PDIA3 |  |
|  | ABHD15 |  |
|  | PLBD2 |  |
|  | SCAMP1 |  |
|  | FGL2 |  |
|  | DIPK2A |  |
|  | TET2 |  |
|  | YTHDF1 |  |
|  | CHML |  |
|  | SMAD4 |  |
|  | ATP2A2 |  |
|  | FBXO30 |  |
|  | SDF4 |  |
|  | TRIM59 |  |
|  | GPRIN3 |  |
|  | NDUFA10 |  |
|  | TM9SF3 |  |
|  | LYZ |  |
|  | SPTSSA |  |
|  | LOC105377945 |  |
|  | REEP3 |  |
|  | LOC107986515 |  |
|  | SLC25A26 |  |
|  | OGA |  |
|  | DOP1B |  |
|  | ARIH1 |  |
|  | RASA1 |  |
|  | LOC107984546 |  |
|  | EIF4EBP2 |  |
|  | FAR1 |  |
|  | EP400 |  |
|  | LOC100288123 |  |
|  | LOC399900 |  |
|  | NCOA3 |  |
|  | NFKB2 |  |
|  | LOC101928429 |  |
|  | ERMN |  |
|  | ATL3 |  |
|  | OGDH |  |
|  | DKFZP586I1420 |  |
|  | DDB2 |  |
|  | MTG2 |  |
|  | DLEU2 |  |
|  | DYNC2H1 |  |
|  | NSUN2 |  |
|  | MON1B |  |
|  | CCNI |  |
|  | PURA |  |
|  | PRRC2B |  |
|  | LINC00963 |  |
|  | ASB6 |  |
|  | ZNF326 |  |
|  | CRACR2A |  |
|  | SLC5A3 |  |
|  | NHLRC2 |  |
|  | ALG10B |  |
|  | LOC101928361 |  |
|  | FOXK1 |  |
|  | ATP2B4 |  |
|  | HERPUD2 |  |
|  | MED13L |  |
|  | DOCK2 |  |
|  | NUP62 |  |
|  | ANAPC2 |  |
|  | KLF16 |  |
|  | SBF1 |  |
|  | RPS6KA1 |  |
|  | ZSWIM6 |  |
|  | HSD17B12 |  |
|  | MSH3 |  |
|  | TRAF7 |  |
|  | XYLT1 |  |
|  | PPID |  |
|  | PAQR4 |  |
|  | STAG3L2 |  |
|  | USP11 |  |
|  | IRS2 |  |
|  | TUG1 |  |
|  | NKAPD1 |  |
|  | MCOLN1 |  |
|  | RICTOR |  |
|  | PROK2 |  |
|  | MMGT1 |  |
|  | PLCB3 |  |
|  | AHNAK |  |
|  | ZNF276 |  |
|  | GALNT10 |  |
|  | GPBP1L1 |  |
|  | SBNO2 |  |
|  | LOC107985392 |  |
|  | INPPL1 |  |
|  | NCLN |  |
|  | OPA3 |  |
|  | POLR2A |  |
|  | BNIP3L |  |
|  | SMG1P6 |  |
|  | NBPF10 |  |
|  | CORO7 |  |
|  | WDR44 |  |
|  | RDX |  |
|  | PLEKHM1P1 |  |
|  | SDHA |  |
|  | C17orf107 |  |
|  | CPPED1 |  |
|  | MARCHF5 |  |
|  | ZBED5 |  |
|  | SNX20 |  |
|  | NFATC2IP |  |
|  | VPS8 |  |
|  | LOC653653 |  |
|  | WASH2P |  |
|  | LOC102724615 |  |
|  | TMEM65 |  |
|  | FAM199X |  |
|  | STMP1 |  |
|  | RIPK3 |  |
|  | LOC105369313 |  |
|  | TRIM28 |  |
|  | ALOX12 |  |
|  | POLH |  |
|  | UBXN11 |  |
|  | LOC107986649 |  |
|  | SLC30A9 |  |
|  | LOC105370567 |  |
|  | HM13 |  |
|  | SLC35E1 |  |
|  | RAB14 |  |
|  | FBXL20 |  |
|  | PLXDC2 |  |
|  | CNOT11 |  |
|  | LINC00528 |  |
|  | DIAPH1 |  |
|  | MTMR9 |  |
|  | LTB4R |  |
|  | SGO1 |  |
|  | SLC4A8 |  |
|  | LINC01004 |  |
|  | PRMT9 |  |
|  | AP5B1 |  |
|  | UBXN2B |  |
|  | ZGRF1 |  |
|  | SNX29P2 |  |
|  | DCUN1D2 |  |
|  | LOC202181 |  |
|  | DHX40 |  |
|  | PPP1R3F |  |
|  | LUZP6 |  |
|  | FTH1 |  |
|  | LOC107984485 |  |
|  | RTL6 |  |
|  | ZHX2 |  |
|  | IAH1 |  |
|  | AGO2 |  |
|  | SF3B4 |  |
|  | ARL8B |  |
|  | LOC105372105 |  |
|  | IQCN |  |
|  | ZNHIT6 |  |
|  | VPS41 |  |
|  | TACC1 |  |
|  | FOXN3 |  |
|  | VWA8 |  |
|  | CSNK1G2 |  |
|  | CHST2 |  |
|  | PLXNA2 |  |
|  | FURIN |  |
|  | IDUA |  |
|  | ELK3 |  |
|  | INPP5D |  |
|  | LOC101927950 |  |
|  | LOC105379235 |  |
|  | ZNF274 |  |
|  | LRSAM1 |  |
|  | CSF2RB |  |
|  | SPIN1 |  |
|  | PPP6R1 |  |
|  | LIMD1 |  |
|  | MFSD10 |  |
|  | AGFG1 |  |
|  | TESC |  |
|  | WWC3 |  |
|  | FAM133DP |  |
|  | STX7 |  |
|  | USP47 |  |
|  | POLR2E |  |
|  | SLC2A13 |  |
|  | SUZ12P1 |  |
|  | MRE11 |  |
|  | BTG2 |  |
|  | DGKQ |  |
|  | SLC26A2 |  |
|  | LRRN1 |  |
|  | NCOR1 |  |
|  | FBXO34 |  |
|  | EIF2D |  |
|  | PNRC1 |  |
|  | RAB21 |  |
|  | MKLN1 |  |
|  | ABCF2 |  |
|  | TYW5 |  |
|  | SLC35A5 |  |
|  | CS |  |
|  | AAK1 |  |
|  | HTATSF1P2 |  |
|  | RFFL |  |
|  | LINC02666 |  |
|  | CALM2 |  |
|  | ZNF582 |  |
|  | E2F6 |  |
|  | HNRNPR |  |
|  | LOC105371531 |  |
|  | EMD |  |
|  | TRIP12 |  |
|  | LOC107984917 |  |
|  | NCOR2 |  |
|  | BICD2 |  |
|  | MTCP1 |  |
|  | DCUN1D1 |  |
|  | LOC107987245 |  |
|  | S1PR2 |  |
|  | TFE3 |  |
|  | CCNT1 |  |
|  | SMAP2 |  |
|  | GPR65 |  |
|  | BMPR2 |  |
|  | MGC16275 |  |
|  | RNASEH1 |  |
|  | LOC102723798 |  |
|  | WASL |  |
|  | SPRN |  |
|  | NAA50 |  |
|  | WDR48 |  |
|  | DDX3X |  |
|  | UHRF1BP1 |  |
|  | RETREG2 |  |
|  | FAM120A |  |
|  | GNB4 |  |
|  | SPIRE1 |  |
|  | C2orf69 |  |
|  | AMIGO2 |  |
|  | ATAD2B |  |
|  | LOC107986513 |  |
|  | TMEM150A |  |
|  | LOC102724584 |  |
|  | TAF9B |  |
|  | SNORA33 |  |
|  | CTDNEP1 |  |
|  | NOTCH1 |  |
|  | LOC374443 |  |
|  | LOC101927723 |  |
|  | FAAP100 |  |
|  | ZNF518B |  |
|  | MBOAT1 |  |
|  | ZFP36L2 |  |
|  | C9orf72 |  |
|  | CABLES2 |  |
|  | RTN1 |  |
|  | DNAJC5 |  |
|  | TPM4 |  |
|  | LOC107986512 |  |
|  | RAB11B |  |
|  | IPMK |  |
|  | FAM172A |  |
|  | HNRNPA2B1 |  |
|  | LINC01619 |  |
|  | UBE2B |  |
|  | SLC46A2 |  |
|  | CACNA2D4 |  |
|  | BCL10 |  |
|  | ELK4 |  |
|  | KLHL26 |  |
|  | QPRT |  |
|  | POLR1D |  |
|  | LOC101927897 |  |
|  | KAT2A |  |
|  | NOTCH2NLB |  |
|  | LOC105372768 |  |
|  | KIAA0754 |  |
|  | PIGF |  |
|  | PMPCB |  |
|  | SLC12A4 |  |
|  | ATXN1 |  |
|  | HK3 |  |
|  | ADCY3 |  |
|  | USB1 |  |
|  | CPT1A |  |
|  | LINC00674 |  |
|  | XPNPEP3 |  |
|  | LOC105370792 |  |
|  | G2E3 |  |
|  | ZNF714 |  |
|  | PCGF3 |  |
|  | ACIN1 |  |
|  | GMFB |  |
|  | CPNE1 |  |
|  | SEC23IP |  |
|  | NPIPB3 |  |
|  | LOC105371271 |  |
|  | LOC107984656 |  |
|  | SMNDC1 |  |
|  | LAMB1 |  |
|  | LINC02757 |  |
|  | UTRN |  |
|  | GYS1 |  |
|  | CRAMP1 |  |
|  | SOS2 |  |
|  | PDE8A |  |
|  | MIS18BP1 |  |
|  | MRPS25 |  |
|  | MPZL1 |  |
|  | UBASH3B |  |
|  | GLCCI1 |  |
|  | NLGN3 |  |
|  | RAB7A |  |
|  | SLED1 |  |
|  | ZNF654 |  |
|  | LOC100127955 |  |
|  | ETV6 |  |
|  | ZNF80 |  |
|  | JAZF1 |  |
|  | ZNFX1 |  |
|  | CEP44 |  |
|  | LOC100507250 |  |
|  | PCDH12 |  |
|  | LINC01000 |  |
|  | PIEZO1 |  |
|  | RPS18P9 |  |
|  | PTP4A2 |  |
|  | FASTKD1 |  |
|  | TMEM138 |  |
|  | PPP1R16B |  |
|  | LOC107984583 |  |
|  | PIK3CG |  |
|  | PMS2P4 |  |
|  | BAG4 |  |
|  | MAMDC4 |  |
|  | BACE1 |  |
|  | SFXN3 |  |
|  | LOC107984317 |  |
|  | MAF |  |
|  | CAPRIN1 |  |
|  | MYLK |  |
|  | EIF4G2 |  |
|  | TRIM37 |  |
|  | F11R |  |
|  | SEC31B |  |
|  | KIF1C |  |
|  | TM9SF2 |  |
|  | TTLL3 |  |
|  | SRSF1 |  |
|  | FHIP2A |  |
|  | RTKN2 |  |
|  | SYVN1 |  |
|  | LMBR1 |  |
|  | BLOC1S6 |  |
|  | QSOX2 |  |
|  | ARID1A |  |
|  | SLC35F6 |  |
|  | KCNJ1 |  |
|  | MICAL1 |  |
|  | CKAP2L |  |
|  | COL18A1 |  |
|  | LOC105374981 |  |
|  | SNORD97 |  |
|  | LOC105369180 |  |
|  | LOC105376875 |  |
|  | APTX |  |
|  | EMC1 |  |
|  | UBE3A |  |
|  | SLC35A3 |  |
|  | CCNK |  |
|  | TRIM45 |  |
|  | LOC105369706 |  |
|  | API5 |  |
|  | CC2D1A |  |
|  | ANKRD50 |  |
|  | ZMYM6 |  |
|  | EFHD2 |  |
|  | LOC107986963 |  |
|  | SERPIND1 |  |
|  | WASH3P |  |
|  | ARHGDIA |  |
|  | NPEPL1 |  |
|  | FKBP11 |  |
|  | LOC105374947 |  |
|  | LOC441155 |  |
|  | SYCP2 |  |
|  | MAB21L2 |  |
|  | KYAT3 |  |
|  | RSPH4A |  |
|  | CFAP36 |  |
|  | GAR1 |  |
|  | WDR33 |  |
|  | CHTF18 |  |
|  | DCAF7 |  |
|  | LOC107986148 |  |
|  | KCNRG |  |
|  | FASN |  |
|  | SIK2 |  |
|  | WBP11 |  |
|  | CCDC144CP |  |
|  | TMEM120B |  |
|  | MEGF9 |  |
|  | ZBTB25 |  |
|  | TRIM24 |  |
|  | UBE2A |  |
|  | WDFY2 |  |
|  | LEMD2 |  |
|  | LOC105371414 |  |
|  | ST8SIA4 |  |
|  | GRIK1 |  |
|  | NPLOC4 |  |
|  | SNORD1C |  |
|  | ST6GAL1 |  |
|  | ASB14 |  |
|  | RNLS |  |
|  | ZFYVE16 |  |
|  | LOC105374010 |  |
|  | EIF3M |  |
|  | SYPL1 |  |
|  | LOC105372857 |  |
|  | SNORD47 |  |
|  | KSR1 |  |
|  | LOC101928762 |  |
|  | MARCHF6 |  |
|  | MRPS6 |  |
|  | LOC107987081 |  |
|  | ZNF595 |  |
|  | LRRC70 |  |
|  | SLC25A34 |  |
|  | LOC105374925 |  |
|  | GOLGA1 |  |
|  | RB1 |  |
|  | MARCKS |  |
|  | CDK8 |  |
|  | LOC101927372 |  |
|  | LOC101929506 |  |
|  | YWHAB |  |
|  | RPS6KA3 |  |
|  | SMIM7 |  |
|  | LOC105378367 |  |
|  | LOC107987080 |  |
|  | LOC105369151 |  |
|  | ZNF865 |  |
|  | SLC18A2 |  |
|  | BTN2A1 |  |
|  | HNRNPD |  |
|  | TMEM87A |  |
|  | LPXN |  |
|  | ZNF43 |  |
|  | MCF2L2 |  |
|  | RRAD |  |
|  | CD28 |  |
|  | WSB1 |  |
|  | LINC02390 |  |
|  | INO80 |  |
|  | EEF1DP3 |  |
|  | THUMPD3 |  |
|  | LOC286437 |  |
|  | LOC107986364 |  |
|  | LOC107986672 |  |
|  | HEXD |  |
|  | DHX33 |  |
|  | LOC100996506 |  |
|  | GNRH1 |  |
|  | MYH10 |  |
|  | SYT2 |  |
|  | LOC105372945 |  |
|  | AGPS |  |
|  | LOC105371637 |  |
|  | AIFM3 |  |
|  | SH3BP1 |  |
|  | BCKDHB |  |
|  | LOC107985780 |  |
|  | PPP3R1 |  |
|  | ERO1A |  |
|  | GPR52 |  |
|  | CD302 |  |
|  | TOP1MT |  |
|  | PRKAG2 |  |
|  | MYCBP2 |  |
|  | CNTNAP1 |  |
|  | LOC107987150 |  |
|  | LOC105369326 |  |
|  | MBNL2 |  |
|  | SLC9A9 |  |
|  | DENND1C |  |
|  | SLC49A4 |  |
|  | RSKR |  |
|  | TAS2R20 |  |
|  | MIEF1 |  |
|  | LOC105372178 |  |
|  | DARS2 |  |
|  | VMA21 |  |
|  | RNF11 |  |
|  | DGCR11 |  |
|  | SPIN4 |  |
|  | PAPOLG |  |
|  | SASS6 |  |
|  | CRIPT |  |
|  | LOC107984617 |  |
|  | SLC9A3R1 |  |
|  | KCTD12 |  |
|  | ORMDL1 |  |
|  | FSTL3 |  |
|  | NSUN5 |  |
|  | LOC105369968 |  |
|  | ARMCX3 |  |
|  | LOC105370877 |  |
|  | GCNT7 |  |
|  | UBE2D1 |  |
|  | ACAD11 |  |
|  | MEF2C |  |
|  | HTR2B |  |
|  | LOC102724517 |  |
|  | NAP1L5 |  |
|  | ZDHHC8 |  |
|  | LOC107986982 |  |
|  | DNAJC16 |  |
|  | MYO5B |  |
|  | LOC105375668 |  |
|  | LOC105376392 |  |
|  | FGR |  |
|  | LOC105376266 |  |
|  | LOC107986290 |  |
|  | ZFX |  |
|  | IPO5P1 |  |
|  | LOC102723604 |  |
|  | NBR2 |  |
|  | GSE1 |  |
|  | LOC107985010 |  |
|  | FLT3 |  |
|  | FLJ42627 |  |
|  | LOC107986707 |  |
|  | ATG16L2 |  |
|  | EXOC1 |  |
|  | CTSK |  |
|  | ABCA5 |  |
|  | NHSL2 |  |
|  | MTMR14 |  |
|  | LOC105373711 |  |
|  | TMPRSS13 |  |
|  | LBHD1 |  |
|  | STON1 |  |
|  | LOC105376220 |  |
|  | CCDC152 |  |
|  | NFYC |  |
|  | CCDC39 |  |
|  | GUSBP15 |  |
|  | LOC105370557 |  |
|  | LINC01176 |  |
|  | TAS2R14 |  |
|  | PFDN1 |  |
|  | PHACTR4 |  |
|  | MXD1 |  |
|  | RYR3 |  |
|  | TAMALIN |  |
|  | LOC105371159 |  |
|  | TENT4A |  |
|  | MAVS |  |
|  | GPR22 |  |
|  | VCAN |  |
|  | LOC105374418 |  |
|  | KYNU |  |
|  | RNF149 |  |
|  | LOC105378726 |  |
|  | LOC107984200 |  |
|  | TNPO1 |  |
|  | ARF1 |  |
|  | LOC101929280 |  |
|  | LOC105370489 |  |
|  | LOC107986981 |  |
|  | PRR11 |  |
|  | HSP90AB4P |  |
|  | CYTB |  |
|  | ARAP1 |  |
|  | OPLAH |  |
|  | LOC107985878 |  |
|  | ZNF154 |  |
|  | PARP12 |  |
|  | CD33 |  |
|  | CHP1 |  |
|  | ZBTB45 |  |
|  | CAMK2D |  |
|  | ETV3 |  |
|  | KCNQ1OT1 |  |
|  | LOC105373826 |  |
|  | LOC652276 |  |
|  | CSK |  |
|  | TMEM168 |  |
|  | LOC105373046 |  |
|  | AGAP5 |  |
|  | MIR23AHG |  |
|  | LOC105378867 |  |
|  | LOC101928081 |  |
|  | LOC105373271 |  |
|  | LOC105372511 |  |
|  | LOC102724428 |  |
|  | RBM12B |  |
|  | LINC00987 |  |
|  | CLDN12 |  |
|  | CUL5 |  |
|  | LOC107986847 |  |
|  | FBXO9 |  |
|  | PLGLB1 |  |
|  | THOC2 |  |
|  | LOC101929240 |  |
|  | ZCCHC18 |  |
|  | ZNF396 |  |
|  | GLYCTK |  |
|  | PDGFB |  |
|  | ALS2 |  |
|  | HERC4 |  |
|  | TSPAN2 |  |
|  | RPRD1B |  |
|  | LOC105376956 |  |
|  | PKD1L1 |  |
|  | LOC105375514 |  |
|  | ARHGEF2 |  |
|  | KHDRBS1 |  |
|  | LINC01376 |  |
|  | TMEM135 |  |
|  | SNX29 |  |
|  | EEF2K |  |
|  | FCHO2 |  |
|  | LZIC |  |
|  | MAU2 |  |
|  | C1QL3 |  |
|  | LOC107984747 |  |
|  | LOC105373077 |  |
|  | MX2 |  |
|  | LOC105369389 |  |
|  | PML |  |
|  | TOR4A |  |
|  | AUH |  |
|  | FGD6 |  |
|  | PAX6 |  |
|  | LOC105374306 |  |
|  | MTPN |  |
|  | PEX14 |  |
|  | PRKACA |  |
|  | LOC107986798 |  |
|  | LOC105375359 |  |
|  | LOC107985977 |  |
|  | BCL11B |  |
|  | PDE1B |  |
|  | ZC2HC1A |  |
|  | MIR4737 |  |
|  | LOC107987057 |  |
|  | KCNH3 |  |
|  | ZNF75D |  |
|  | MIR4712 |  |
|  | QKI |  |
|  | DSN1 |  |
|  | TAS2R19 |  |
|  | STAG3L3 |  |
|  | APC2 |  |
|  | USP38 |  |
|  | MYBPC3 |  |
|  | KANSL1L |  |
|  | LOC105378487 |  |
|  | NR2F6 |  |
|  | LOC107986600 |  |
|  | LOC101929109 |  |
|  | LOC107987192 |  |
|  | TSHZ3 |  |
|  | LOC339059 |  |
|  | LOC105377402 |  |
|  | PKD1L3 |  |
|  | IQCB1 |  |
|  | MTHFR |  |
|  | FAM114A2 |  |
|  | ATP6V1C2 |  |
|  | NHSL1 |  |
|  | ARMC5 |  |
|  | ATRX |  |
|  | FAM157C |  |
|  | ORM1 |  |
|  | LOC107985120 |  |
|  | FGF9 |  |
|  | PHF2 |  |
|  | NIPA1 |  |
|  | LOC105370563 |  |
|  | CD74 |  |
|  | PIK3R3 |  |
|  | LOC100190986 |  |
|  | PRRC2A |  |
|  | TPRN |  |
|  | ZBTB42 |  |
|  | SNORA72 |  |
|  | IL7 |  |
|  | LOC105375061 |  |
|  | LTA4H |  |
|  | CHROMR |  |
|  | ADAMTS5 |  |
|  | PCM1 |  |
|  | VPS13A |  |
|  | FARP1 |  |
|  | LOC105375669 |  |
|  | A2M |  |
|  | LOC107987147 |  |
|  | UBE2R2 |  |
|  | LINC01881 |  |
|  | MAP4K5 |  |
|  | RBM42 |  |
|  | C1orf56 |  |
|  | LOC107986455 |  |
|  | NRP1 |  |
|  | QPCTL |  |
|  | SCART1 |  |
|  | LOC101927730 |  |
|  | LOC107987269 |  |
|  | LOC107985567 |  |
|  | SNORD5 |  |
|  | TENM1 |  |
|  | ZNF117 |  |
|  | GPR137B |  |
|  | C10orf105 |  |
|  | PTPN4 |  |
|  | CAPZA1 |  |
|  | LOC105373652 |  |
|  | LOC107987261 |  |
|  | LOC100335030 |  |
|  | EYS |  |
|  | MTERF4 |  |
|  | LOC105371894 |  |
|  | PTENP1 |  |
|  | BCL6 |  |
|  | LOC105373730 |  |
|  | SLC35F5 |  |
|  | ERCC8 |  |
|  | ELOVL6 |  |
|  | ZBTB40 |  |
|  | ANGPTL2 |  |
|  | ARHGAP31 |  |
|  | LOC107984347 |  |
|  | OTUD4 |  |
|  | HERC2 |  |
|  | LOC107985193 |  |
|  | PHTF2 |  |
|  | LOC105371168 |  |
|  | LOC107984659 |  |
|  | COL10A1 |  |
|  | ANGPT2 |  |
|  | RAB29 |  |
|  | ELF4 |  |
|  | ZNF337 |  |
|  | NPRL3 |  |
|  | LOC107985119 |  |
|  | LOC101928126 |  |
|  | TGFB1 |  |
|  | DMAC2L |  |
|  | FAM20A |  |
|  | RIC8B |  |
|  | LOC107985921 |  |
|  | TVP23C |  |
|  | MIR7152 |  |
|  | LOC107985289 |  |
|  | TMEM170B |  |
|  | FRY |  |
|  | LOC107986477 |  |
|  | MIR133A1HG |  |
|  | PPP2R3A |  |
|  | PIGB |  |
|  | SNORD87 |  |
|  | PA2G4P4 |  |
|  | LINC02019 |  |
|  | LOC105370504 |  |
|  | SOX12 |  |
|  | SAFB |  |
|  | LOC107985154 |  |
|  | EIPR1 |  |
|  | MAK |  |
|  | MIR7854 |  |
|  | LOC105376991 |  |
|  | ZNF706 |  |
|  | TMEM86A |  |
|  | CCDC200 |  |
|  | LOC105370780 |  |
|  | ARRDC1 |  |
|  | ATG7 |  |
|  | CRSP8P |  |
|  | LOC107985497 |  |
|  | SH2B3 |  |
|  | SERINC4 |  |
|  | RHOQ |  |
|  | POLA1 |  |
|  | ETFRF1 |  |
|  | LOC105378000 |  |
|  | LOC105371512 |  |
|  | TARDBPP3 |  |
|  | MAP2K5 |  |
|  | GP1BA |  |
|  | LOC107985367 |  |
|  | ARMC2 |  |
|  | ME3 |  |
|  | PYHIN1 |  |
|  | ITPK1 |  |
|  | AUTS2 |  |
|  | PAK2 |  |
|  | MIR4648 |  |
|  | NRGN |  |
|  | LOC105376707 |  |
|  | LOC105379420 |  |
|  | YWHAH |  |
|  | LPAR6 |  |
|  | LOC101927018 |  |
|  | SLC14A1 |  |
|  | TAS1R3 |  |
|  | LINC02803 |  |
|  | PAPSS2 |  |
|  | LOC105375779 |  |
|  | LOC102724322 |  |
|  | SSH2 |  |
|  | ACSL1 |  |
|  | SEPTIN7P13 |  |
|  | NPIPB5 |  |
|  | LOC105374775 |  |
|  | MIR3173 |  |
|  | KIAA1656 |  |
|  | LOC105369161 |  |
|  | LOC107984718 |  |
|  | AHSA2P |  |
|  | LOC105378410 |  |
|  | LOC101927647 |  |
|  | ADNP |  |
|  | ACTG1P20 |  |
|  | LOC105369663 |  |
|  | LOC105377449 |  |
|  | KLRA1P |  |
|  | GOLGA8A |  |
|  | LOC105375492 |  |
|  | LOC107985147 |  |
|  | LOC105377452 |  |
|  | TNRC18 |  |
|  | LRCH1 |  |
|  | CPNE8 |  |
|  | CTNNA1 |  |
|  | LOC646762 |  |
|  | PPM1M |  |
|  | DENND6A |  |
|  | MAP4K3 |  |
|  | C3orf20 |  |
|  | ZNF827 |  |
|  | PZP |  |
|  | PP2D1 |  |
|  | OMG |  |
|  | PADI2 |  |
|  | LOC107986862 |  |
|  | STON2 |  |
|  | SHLD1 |  |
|  | IGHMBP2 |  |
|  | LOC107984275 |  |
|  | PGM5P2 |  |
|  | SNORD102 |  |
|  | LOC105372877 |  |
|  | LOC105374610 |  |
|  | MDGA1 |  |
|  | LOC105376591 |  |
|  | LOC107985876 |  |
|  | SCNM1 |  |
|  | LOC105377102 |  |
|  | RASA4 |  |
|  | UNC5A |  |
|  | SMIM18 |  |
|  | PLEKHM1 |  |
|  | PRDM2 |  |
|  | FANCM |  |
|  | DEDD |  |
|  | CPOX |  |
|  | LOC105374773 |  |
|  | LOC100506258 |  |
|  | CLIP4 |  |
|  | FMR1 |  |
|  | TAF4 |  |
|  | PID1 |  |
|  | TAS2R13 |  |
|  | DDX60L |  |
|  | MICOS10P1 |  |
|  | UBE2E3 |  |
|  | ARPP19 |  |
|  | LOC107984120 |  |
|  | NFATC2 |  |
|  | SH3D19 |  |
|  | FAM110A |  |
|  | LPEQ6126 |  |
|  | PHF12 |  |
|  | GABRR2 |  |
|  | ZNF264 |  |
|  | LOC105378122 |  |
|  | LOC105379426 |  |
|  | LOC101927227 |  |
|  | LOC105375945 |  |
|  | TSC22D1 |  |
|  | LOC105375260 |  |
|  | MVB12B |  |
|  | DENND10 |  |
|  | BAIAP2L1 |  |
|  | PIAS3 |  |
|  | SPATA5 |  |
|  | LOC105379195 |  |
|  | BRD7P3 |  |
|  | TAB2 |  |
|  | MICAL2 |  |
|  | LOC105369305 |  |
|  | TMEM67 |  |
|  | LOC105378620 |  |
|  | COP1 |  |
|  | LOC107985487 |  |
|  | SACM1L |  |
|  | LOC107986079 |  |
|  | ADGRE3 |  |
|  | ZFPM1 |  |
|  | TENT4B |  |
|  | CEMIP |  |
|  | PHF6 |  |
|  | MTMR10 |  |
|  | PPP1R27 |  |
|  | LOC105374897 |  |
|  | LOC107984871 |  |
|  | LOC105370556 |  |
|  | MIR27A |  |
|  | NDUFA11 |  |
|  | SLC39A11 |  |
|  | LOC105375218 |  |
|  | LOC105377872 |  |
|  | LOC107986015 |  |
|  | CORO1C |  |
|  | MEF2A |  |
|  | LOXL2 |  |
|  | LOC114224 |  |
|  | LOC105377929 |  |
|  | TLR9 |  |
|  | LOC107985481 |  |
|  | ARPC2 |  |
|  | RANBP3 |  |
|  | UCP3 |  |
|  | SNORA80E |  |
|  | LOC105370664 |  |
|  | RNF13 |  |
|  | DNM1L |  |
|  | XXYLT1 |  |
|  | LRRK2 |  |
|  | LOC101928007 |  |
|  | LOC107984214 |  |
|  | CHP1P2 |  |
|  | RAPGEF1 |  |
|  | MTF2 |  |
|  | HOXB2 |  |
|  | VSIG10 |  |
|  | LOC107984034 |  |
|  | PLEKHA5 |  |
|  | MIR6755 |  |
|  | ZFYVE28 |  |
|  | DISC2 |  |
|  | TIAM1 |  |
|  | MRTFA |  |
|  | CYCSP52 |  |
|  | ZNF397 |  |
|  | LOC105371651 |  |
|  | LETM1 |  |
|  | TSNARE1 |  |
|  | MIDEAS |  |
|  | UBE2E2 |  |
|  | TMEM164 |  |
|  | NPIPB11 |  |
|  | SHTN1 |  |
|  | PEAK3 |  |
|  | LOC107986457 |  |
|  | WDR90 |  |
|  | AKAP5 |  |
|  | MIR3140 |  |
|  | LOC105371942 |  |
|  | CLASP1 |  |
|  | MIR6895 |  |
|  | LOC105375208 |  |
|  | MIR558 |  |
|  | LOC107987182 |  |
|  | LOC107984992 |  |
|  | LOC107987399 |  |
|  | MIR597 |  |
|  | LINC02102 |  |
|  | LOC107986069 |  |
|  | UMPS |  |
|  | CLTC |  |
|  | CA4 |  |
|  | LOC107984837 |  |
|  | MIR6867 |  |
|  | TNKS2 |  |
|  | FIG4 |  |
|  | LOC105370777 |  |
|  | MIR3605 |  |
|  | KCTD9 |  |
|  | COX15 |  |
|  | JAML |  |
|  | CMIP |  |
|  | LOC105372521 |  |
|  | KMO |  |
|  | SKAP2 |  |
|  | PARP14 |  |
|  | LOC105379251 |  |
|  | SPDYE5 |  |
|  | LOC105378539 |  |
|  | LOC107984661 |  |
|  | MIR548C |  |
|  | MIR548Z |  |
|  | TRDMT1 |  |
|  | MIR5194 |  |
|  | A2MP1 |  |
|  | LOC101927692 |  |
|  | SERHL2 |  |
|  | RFX2 |  |
|  | SLC16A5 |  |
|  | LOC105373264 |  |
|  | LOC105373204 |  |
|  | BMP8A |  |
|  | SSBP4 |  |
|  | IP6K1 |  |
|  | ARSB |  |
|  | LOC101927066 |  |
|  | TSC22D4 |  |
|  | NECTIN1 |  |
|  | CCDC174 |  |
|  | HPCAL1 |  |
|  | LOC221946 |  |
|  | LOC107984984 |  |
|  | PTK2B |  |
|  | SUMO1P3 |  |
|  | F2RL2 |  |
|  | ADARB1 |  |
|  | LOC105378046 |  |
|  | MIR4440 |  |
|  | ARHGAP26 |  |
|  | MUSTN1 |  |
|  | LOC105375500 |  |
|  | MNAT1 |  |
|  | GLI1 |  |
|  | LOC107986431 |  |
|  | LOC107987258 |  |
|  | DTNB |  |
|  | LOC105372835 |  |
|  | TAOK2 |  |
|  | MIR4742 |  |
|  | LOC105371088 |  |
|  | TRAF5 |  |
|  | ESRP2 |  |
|  | APOA2 |  |
|  | LOC101927911 |  |
|  | ROCK1P1 |  |
|  | SPNS2 |  |
|  | LOC107986435 |  |
|  | SPATA6 |  |
|  | COPG2IT1 |  |
|  | AMPD2 |  |
|  | UICLM |  |
|  | MAP2K3 |  |
|  | MIR875 |  |
|  | LOC105376033 |  |
|  | MIR1238 |  |
|  | MIR8086 |  |
|  | RAD51D |  |
|  | TMEM86B |  |
|  | CHD1 |  |
|  | NPIPB4 |  |
|  | PAPPA |  |
|  | LOC105370047 |  |
|  | STXBP4 |  |
|  | SETD4 |  |
|  | MTFR2 |  |
|  | SNORD11 |  |
|  | LOC101927770 |  |
|  | MIR6075 |  |
|  | ZNF615 |  |
|  | RFPL1S |  |
|  | TAF1C |  |
|  | LOC105370871 |  |
|  | ARHGEF5 |  |
|  | NTRK1 |  |
|  | FDPSP2 |  |
|  | ZNF7 |  |
|  | AQP11 |  |
|  | LOC107986762 |  |
|  | MIR1255A |  |
|  | CSF1 |  |
|  | LOC105372964 |  |
|  | MIR4513 |  |
|  | LOC101929269 |  |
|  | BBIP1 |  |
|  | GNLY |  |
|  | ANKRD36BP1 |  |
|  | CLEC1B |  |
|  | MIR6876 |  |
|  | NRON |  |
|  | CACFD1 |  |
|  | KIF2A |  |
|  | MIR4802 |  |
|  | RBM26 |  |
|  | LOC105375652 |  |
|  | FOXP4 |  |
|  | SCARB1 |  |
|  | GUSB |  |
|  | AHCYL2 |  |
|  | LOC389834 |  |
|  | MIR548D1 |  |
|  | CREB1 |  |
|  | LOC107984635 |  |
|  | LOC105376725 |  |
|  | CCDC73 |  |
|  | LOC105371981 |  |
|  | NCOA6 |  |
|  | PLCL1 |  |
|  | LOC107984202 |  |
|  | INTS1 |  |
|  | BSCL2 |  |
|  | SLC25A52 |  |
|  | AMZ1 |  |
|  | CPSF6 |  |
|  | ABL1 |  |
|  | VSIG2 |  |
|  | MIR624 |  |
|  | NOTCH2 |  |
|  | CCDC18 |  |
|  | SERPINB8 |  |
|  | SRP68 |  |
|  | SPECC1 |  |
|  | SMARCD3 |  |
|  | MIR4720 |  |
|  | MTA3 |  |
|  | PGAP2 |  |
|  | MIR142 |  |
|  | PLAA |  |
|  | PHOSPHO1 |  |
|  | CD101 |  |
|  | LOC105377295 |  |
|  | MIR6742 |  |
|  | IFNLR1 |  |
|  | TP53BP1 |  |
|  | FMNL1 |  |
|  | MIR4477B |  |
|  | GCSHP3 |  |
|  | GOLGA8K |  |
|  | RBM44 |  |
|  | LOC105372816 |  |
|  | MIR6864 |  |
|  | RBPMS |  |
|  | LOC105371816 |  |
|  | SNORA92 |  |
|  | GNRHR |  |
|  | LOC107987251 |  |
|  | NEO1 |  |
|  | FLJ42393 |  |
|  | MIR6866 |  |
|  | GRAMD1A |  |
|  | THBS4 |  |
|  | LACTB |  |
|  | LRRTM2 |  |
|  | LINC02754 |  |
|  | LCORL |  |
|  | SNORA49 |  |
|  | RAP2C |  |
|  | TRPA1 |  |
|  | ARF3 |  |
|  | LOC107985768 |  |
|  | TBCE |  |
|  | LOC105379096 |  |
|  | TBC1D2 |  |
|  | LOC107985551 |  |
|  | MDN1 |  |
|  | SHOC1 |  |
|  | LOC105371131 |  |
|  | LOC107985262 |  |
|  | PPHLN1 |  |
|  | MIR186 |  |
|  | LOC105371507 |  |
|  | SNORD56B |  |
|  | ECM2 |  |
|  | FLYWCH1 |  |
|  | TTC7A |  |
|  | MIR4632 |  |
|  | PXN |  |
|  | MIR548AC |  |
|  | SNORD143 |  |
|  | LOC101927445 |  |
|  | EML4 |  |
|  | OSBPL5 |  |
|  | LOC105371692 |  |
|  | MIR1248 |  |
|  | SCARNA28 |  |
|  | RBM41 |  |
|  | GPR18 |  |
|  | LOC105370461 |  |
|  | RHOG |  |
|  | GALNT2 |  |
|  | GPR25 |  |
|  | LOC441081 |  |
|  | UNC93B1 |  |
|  | CDK1 |  |
|  | LOC101927283 |  |
|  | VIRMA |  |
|  | KCTD15 |  |
|  | SPATC1 |  |
|  | PSPH |  |
|  | GINS3 |  |
|  | ADCY7 |  |
|  | SCARNA27 |  |
|  | LOC107986428 |  |
|  | LOC102724120 |  |
|  | CKAP2 |  |
|  | TP53 |  |
|  | PCDHGB3 |  |
|  | SLC43A2 |  |
|  | WDR62 |  |
|  | SLFN14 |  |
|  | PPP2R5E |  |
|  | DPYSL4 |  |
|  | SLC15A2 |  |
|  | WDTC1 |  |
|  | LINC00294 |  |
|  | ABCC6 |  |
|  | PCCA |  |
|  | PHYH |  |
|  | LOC105375112 |  |
|  | PRSS27 |  |
|  | CC2D2A |  |
|  | LOC107986436 |  |
|  | RNF17 |  |
|  | JPT1 |  |
|  | MIR548J |  |
|  | RELT |  |
|  | DEPDC5 |  |
|  | TREML5P |  |
|  | STIM1 |  |
|  | ADAT3 |  |
|  | LOC105371922 |  |
|  | LINC02705 |  |
|  | BRD1 |  |
|  | CCDC159 |  |
|  | TASOR |  |
|  | LOC105370052 |  |
|  | PAK1 |  |
|  | THAP9 |  |
|  | GNG3 |  |
|  | RFESD |  |
|  | ARHGAP33 |  |
|  | LOC100129617 |  |
|  | ASB9P1 |  |
|  | LOC100506497 |  |
|  | EVI2B |  |
|  | AIRN |  |
|  | LOC105374152 |  |
|  | DEFB131B |  |
|  | CAPN7 |  |
|  | ANGPTL3 |  |
|  | MIR3146 |  |
|  | GPR34 |  |
|  | MARK3 |  |
|  | CCDC51 |  |
|  | CRHR2 |  |
|  | TBL1X |  |
|  | SLCO3A1 |  |
|  | AGAP3 |  |
|  | UST |  |
|  | ZBTB8A |  |
|  | LOC105371870 |  |
|  | LOC642696 |  |
|  | LOC105373233 |  |
|  | MAP2K4 |  |
|  | MIR4269 |  |
|  | PTGDS |  |
|  | LOC102725180 |  |
|  | DNM3OS |  |
|  | LOC107986369 |  |
|  | HSD17B3 |  |
|  | LOC107984459 |  |
|  | FLT1 |  |
|  | LOC105372214 |  |
|  | SIN3B |  |
|  | MIR4420 |  |
|  | LPCAT2 |  |
|  | LOC101928215 |  |
|  | LOC102723331 |  |
|  | LOC100129697 |  |
|  | SLC6A8 |  |
|  | SNORA101B |  |
|  | LOC105370816 |  |
|  | FZD7 |  |
|  | LOC105377932 |  |
|  | LRRC28 |  |
|  | FAIM |  |
|  | LOC107983996 |  |
|  | LOC105371573 |  |
|  | OR13D1 |  |
|  | LOC107984244 |  |
|  | LOC105375386 |  |
|  | LOC101928214 |  |
|  | LPCAT1 |  |
|  | LOC105370677 |  |
|  | KCNA6 |  |
|  | CYB5R3 |  |
|  | PI4KA |  |
|  | TMUB2 |  |
|  | PTPN22 |  |
|  | LOC105373581 |  |
|  | CYB5R2 |  |
|  | TMEM154 |  |
|  | LOC105374166 |  |
|  | LINC01372 |  |
|  | ACER3 |  |
|  | HLCS |  |
|  | RAP1GAP2 |  |
|  | PRAP1 |  |
|  | PRXL2A |  |
|  | PTPN11 |  |
|  | ZNF491 |  |
|  | CLVS1 |  |
|  | STRIP1 |  |
|  | LOC105370502 |  |
|  | MIR4257 |  |
|  | CNOT10 |  |
|  | SYDE1 |  |
|  | ERF |  |
|  | LOC105374555 |  |
|  | TSPAN1 |  |
|  | USP32P1 |  |
|  | LOC107986522 |  |
|  | MYPOP |  |
|  | ZMYND15 |  |
|  | JAM2 |  |
|  | CLDN19 |  |
|  | SYT15 |  |
|  | PCSK1 |  |
|  | LOC107985012 |  |
|  | MAPRE3 |  |
|  | TLK2 |  |
|  | NOMO2 |  |
|  | GDPD5 |  |
|  | LOC107985322 |  |
|  | EIF4G3 |  |
|  | THRB |  |
|  | PPFIBP2 |  |
|  | SUSD2 |  |
|  | APBA1 |  |
|  | LOC105371532 |  |
|  | PSEN2 |  |
|  | C4orf50 |  |
|  | SLC22A15 |  |
|  | LOC105370363 |  |
|  | LOC105369421 |  |
|  | LOC101927151 |  |
|  | CXCL1 |  |
|  | SIGLEC14 |  |
|  | SIDT1 |  |
|  | SYT9 |  |
|  | SNORA19 |  |
|  | LOC105379163 |  |
|  | ZNF578 |  |
|  | LOC105374663 |  |
|  | LOC105371449 |  |
|  | LOC105374943 |  |
|  | UBE2C |  |
|  | ARSG |  |
|  | TREML4 |  |
|  | AJM1 |  |
|  | GTF2A1 |  |
|  | SMN2 |  |
|  | C1orf105 |  |
|  | ALOX12P2 |  |
|  | NEAT1 |  |
|  | IL17RE |  |
|  | AP2A2 |  |
|  | ITIH2 |  |
|  | RMDN1 |  |
|  | FCRL6 |  |
|  | LOC105374953 |  |
|  | MEN1 |  |
|  | DMPK |  |
|  | RINT1 |  |
|  | SYT17 |  |
|  | HRNR |  |
|  | SHLD2 |  |
|  | ATP11C |  |
|  | LOC101929894 |  |
|  | ZNRF2P2 |  |
|  | PRIMPOL |  |
|  | LOC105377685 |  |
|  | LOC105373952 |  |
|  | AGAP12P |  |
|  | PSTPIP1 |  |
|  | LINC01888 |  |
|  | ASPSCR1 |  |
|  | SNORA77 |  |
|  | CPT1B |  |
|  | SUOX |  |
|  | SPAG8 |  |
|  | OXER1 |  |
|  | TIFAB |  |
|  | NR4A1 |  |
|  | LOC105377632 |  |
|  | STAU1 |  |
|  | ALG14 |  |
|  | NAGS |  |
|  | MIER3 |  |
|  | MYO1F |  |
|  | LOC107985874 |  |
|  | LOC107985529 |  |
|  | GJC2 |  |
|  | SEC24D |  |
|  | CTNND1 |  |
|  | KMT2D |  |
|  | LOC105376436 |  |
|  | LOC107986345 |  |
|  | LINC02610 |  |
|  | FAM3C |  |
|  | LOC105378050 |  |
|  | CDK19 |  |
|  | PARM1 |  |
|  | ACVR1 |  |
|  | LOC105373618 |  |
|  | MARF1 |  |
|  | LOC105378576 |  |
|  | PLD4 |  |
|  | MYEF2 |  |
|  | LOC105370542 |  |
|  | NRBP2 |  |
|  | CMC1 |  |
|  | LOC107986190 |  |
|  | DLG3 |  |
|  | AP3D1 |  |
|  | NPIPB9 |  |
|  | RUBCN |  |
|  | CHCHD5 |  |
|  | MAPK10 |  |
|  | IFT88 |  |
|  | LOC107985211 |  |
|  | LOC105377299 |  |
|  | LINC00539 |  |
|  | LOC101929004 |  |
|  | LOC107986253 |  |
|  | LOC105373504 |  |
|  | SPTY2D1OS |  |
|  | MEIOC |  |
|  | CSPP1 |  |
|  | LOC107986660 |  |
|  | ZSCAN9 |  |
|  | SMARCC1 |  |
|  | CEP128 |  |
|  | WDR19 |  |
|  | ZFP69 |  |
|  | SLC25A17 |  |
|  | LBX2 |  |
|  | SMN1 |  |
|  | PRR5 |  |
|  | GEMIN8 |  |
|  | LOC107984088 |  |
|  | LOC105376830 |  |
|  | LOC105373195 |  |
|  | LOC107986514 |  |
|  | LINC02210 |  |
|  | CACHD1 |  |
|  | NRCAM |  |
|  | PLAG1 |  |
|  | LOC101930091 |  |
|  | ABRA |  |
|  | LOC105373673 |  |
|  | GOLGA8R |  |
|  | LOC107984575 |  |
|  | LIN9 |  |
|  | CCDC88B |  |
|  | CCP110 |  |
|  | MMP19 |  |
|  | ZRANB3 |  |
|  | TP53I11 |  |
|  | CSTF2 |  |
|  | DAPK2 |  |
|  | ZNF343 |  |
|  | CAPN15 |  |
|  | KHK |  |
|  | HIC1 |  |
|  | RWDD2B |  |
|  | DPRXP4 |  |
|  | GNAI2 |  |
|  | BRD2 |  |
|  | NAPB |  |
|  | FAM135A |  |
|  | LOC107984669 |  |
|  | LOC105377743 |  |
|  | SOX13 |  |
|  | FANCA |  |
|  | MAGED1 |  |
|  | CDC25B |  |
|  | SMAD5 |  |
|  | CHAD |  |
|  | OMD |  |
|  | FN3KRP |  |
|  | LOC105375361 |  |
|  | CDCA7 |  |
|  | RUSC2 |  |
|  | WASH8P |  |
|  | HHAT |  |
|  | GUCY1A1 |  |
|  | ZIK1 |  |
|  | ARL6 |  |
|  | UHRF1 |  |
|  | FAM193B |  |
|  | EPHB6 |  |
|  | MMS22L |  |
|  | SLC35G2 |  |
|  | PRPF31 |  |
|  | PITPNM1 |  |
|  | STN1 |  |
|  | CEP131 |  |
|  | ERCC4 |  |
|  | LOC105374535 |  |
|  | KIF16B |  |
|  | NME7 |  |
|  | CDK5R1 |  |
|  | CAPN12 |  |
|  | ZNF225 |  |
|  | WDR35 |  |
|  | ZBTB34 |  |
|  | CPAMD8 |  |
|  | AKAP8 |  |
|  | GTF2H2B |  |
|  | NBPF9 |  |
|  | DCAF1 |  |
|  | AFAP1 |  |
|  | NACC2 |  |
| pDCs | CXCL8 | up |
|  | G0S2 |  |
|  | LGALS2 |  |
|  | FCN1 |  |
|  | SGK1 |  |
|  | S100A12 |  |
|  | IL1B |  |
|  | VCAN |  |
|  | HIST1H2AE |  |
|  | ZNF711 |  |
|  | SERPINA1 |  |
|  | FAM53B-AS1 |  |
|  | TREM1 |  |
|  | RPPH1 |  |
|  | CD83 |  |
|  | KLHL11 |  |
|  | S100A8 |  |
|  | IRF4 |  |
|  | CFP |  |
|  | TLR2 |  |
|  | FPR3 |  |
|  | PTX3 |  |
|  | NFKBIA |  |
|  | AX747826 |  |
|  | MAFB |  |
|  | RP11-235E17.4 |  |
|  | FOS |  |
|  | C5AR1 |  |
|  | FNDC9 |  |
|  | CSTA |  |
|  | TRIB1 |  |
|  | GNG11 |  |
|  | TP53INP2 |  |
|  | GLUL |  |
|  | CD163 |  |
|  | PLAUR |  |
|  | ID2 |  |
|  | HIST1H1D |  |
|  | C16orf87 |  |
|  | AGPAT9 |  |
|  | FOSB |  |
|  | BRE-AS1 |  |
|  | BCL2L11 |  |
|  | IER3 |  |
|  | HIST1H4H |  |
|  | PHEX-AS1 |  |
|  | HIST1H4E |  |
|  | FCGR2A |  |
|  | CEBPD |  |
|  | KLHL20 |  |
|  | CXCL2 |  |
|  | HMOX1 |  |
|  | CNTF |  |
|  | CHEK1 |  |
|  | CLEC7A |  |
|  | GNA13 |  |
|  | HYMAI |  |
|  | LSM7 |  |
|  | IRAK3 |  |
|  | CXCL3 |  |
|  | LYZ |  |
|  | THBS1 |  |
|  | MARCKS |  |
|  | PMAIP1 |  |
|  | CHST2 |  |
|  | LOC100129198 |  |
|  | RBM34 |  |
|  | LOC100127886 |  |
|  | AOAH |  |
|  | PDE4B |  |
|  | IREB2 |  |
|  | KLF10 |  |
|  | CDKN1A |  |
|  | GADD45A |  |
|  | SDE2 |  |
|  | GLB1L |  |
|  | TRAF6 |  |
|  | SSBP1 |  |
|  | NAMPT |  |
|  | LOC399900 |  |
|  | R3HDM4 |  |
|  | HIST1H1E |  |
|  | PIK3CA |  |
|  | RP1-68D18.4 |  |
|  | LOC340184 |  |
|  | BCL10 |  |
|  | MOP-1 |  |
|  | TIPARP |  |
|  | LOC100287221 |  |
|  | HSPC081 |  |
|  | HTR2B |  |
|  | QPCT |  |
|  | FAM8A1 |  |
|  | RHOC |  |
|  | NFE2L3 |  |
|  | NDRG2 |  |
|  | SLCO3A1 |  |
|  | SOX4 |  |
|  | IER5 |  |
|  | FCGR2C |  |
|  | MSR1 |  |
|  | TRA2A |  |
|  | RP4-742J24.2 |  |
|  | GP1BA |  |
|  | CSF1R |  |
|  | KLF4 |  |
|  | P2RY2 |  |
|  | TCEA1 |  |
|  | BTG3 |  |
|  | FLJ13773 |  |
|  | KLF11 |  |
|  | ATAD2 |  |
|  | PRDM1 |  |
|  | ADAM9 |  |
|  | NID2 |  |
|  | DUSP1 |  |
|  | IRS2 |  |
|  | PPIF |  |
|  | RNF103 |  |
|  | TCF7L2 |  |
|  | GFI1 |  |
|  | TAGAP |  |
|  | PYGL |  |
|  | CLEC10A |  |
|  | MRPL54 |  |
|  | BACH1 |  |
|  | ZNF503 |  |
|  | ANKLE2 |  |
|  | CXCR4 |  |
|  | BCL6 |  |
|  | HIST1H2BE |  |
|  | MYLIP |  |
|  | RTTN |  |
|  | S100A9 |  |
|  | RP11-231E19.1 |  |
|  | P2RY10 |  |
|  | CCL5 |  |
|  | SLC7A11-AS1 |  |
|  | GPR83 |  |
|  | H3F3A |  |
|  | CELF2-AS1 |  |
|  | CPT1A |  |
|  | POLG |  |
|  | RARS2 |  |
|  | SLC10A1 |  |
|  | ELL2 |  |
|  | SLC7A7 |  |
|  | RP4-781K5.2 |  |
|  | ATF3 |  |
|  | IQCC |  |
|  | APLP2 |  |
|  | RP5-1065J22.8 |  |
|  | TSSK3 |  |
|  | PIM3 |  |
|  | HIST1H2AJ |  |
|  | BTG1 |  |
|  | PIK3R4 |  |
|  | CPSF1 |  |
|  | PRO2852 |  |
|  | INO80 |  |
|  | HMGB3 |  |
|  | FAM198B |  |
|  | PPP1R2 |  |
|  | RAB11FIP1 |  |
|  | LOC101928191 |  |
|  | RIN2 |  |
|  | CYBA |  |
|  | NR2C1 |  |
|  | CPVL |  |
|  | ELF2 |  |
|  | BD495725 |  |
|  | FAM53B |  |
|  | GAFA1 |  |
|  | LST1 |  |
|  | PLBD1 |  |
|  | CHPT1 |  |
|  | BAZ2A |  |
|  | CHMP1B |  |
|  | SLC16A6 |  |
|  | PDE4D |  |
|  | H2BFS |  |
|  | SF1 |  |
|  | PIGA |  |
|  | FOSL2 |  |
|  | BC041363 |  |
|  | SLC46A2 |  |
|  | NFIL3 |  |
|  | MCL1 |  |
|  | ZNF669 |  |
|  | SMAGP |  |
|  | F3 |  |
|  | RAB33B |  |
|  | RXRB |  |
|  | WIPI2 |  |
|  | RGCC |  |
|  | NFKBIZ |  |
|  | PQBP1 |  |
|  | PTMS |  |
|  | OTULIN |  |
|  | ISG20L2 |  |
|  | AHR |  |
|  | FRMD8 |  |
|  | TPM3 |  |
|  | SLC39A8 |  |
|  | CCNB2 |  |
|  | FYTTD1 |  |
|  | RCL1 |  |
|  | WDYHV1 |  |
|  | DUSP10 |  |
|  | HIST1H1C |  |
|  | RGS2 |  |
|  | TUFT1 |  |
|  | LOC102724718 |  |
|  | LOC101927211 |  |
|  | SNAI1 |  |
|  | ANXA1 |  |
|  | SPIDR |  |
|  | LINC00216 |  |
|  | TNFAIP3 |  |
|  | RAB20 |  |
|  | ZNF124 |  |
|  | RLIM |  |
|  | ATXN2L |  |
|  | SPARC |  |
|  | SLC22A15 |  |
|  | RHOBTB3 |  |
|  | CFLAR |  |
|  | CYP1B1 |  |
|  | VDR |  |
|  | LILRA3 |  |
|  | NADK |  |
|  | SRXN1 |  |
|  | AHI1 |  |
|  | SPRED1 |  |
|  | CCNY |  |
|  | IGFBP7 |  |
|  | ZFAND5 |  |
|  | MTDH |  |
|  | UTRN |  |
|  | UBXN2A |  |
|  | CNOT8 |  |
|  | CEMP1 |  |
|  | RNF39 |  |
|  | CACUL1 |  |
|  | NOL12 |  |
|  | TIGD1 |  |
|  | TADA1 |  |
|  | KCTD20 |  |
|  | HIST1H2BH |  |
|  | NXT1 |  |
|  | SUPT7L |  |
|  | RP9 |  |
|  | RPAIN |  |
|  | FAM109B |  |
|  | CHD2 |  |
|  | CD74 |  |
|  | SREK1 |  |
|  | CCNL1 |  |
|  | WDFY3 |  |
|  | NUBPL |  |
|  | GADD45B |  |
|  | TLR4 |  |
|  | SNORA71A |  |
|  | VAMP2 |  |
|  | LGALSL |  |
|  | FURIN |  |
|  | TNF |  |
|  | RAP2C |  |
|  | IL6 |  |
|  | LOC101928371 |  |
|  | ZNF385A |  |
|  | EPB42 |  |
|  | CPAMD8 |  |
|  | ZNF460 |  |
|  | UBALD2 |  |
|  | MEX3C |  |
|  | ALOX12 |  |
|  | ZNF784 |  |
|  | FHL1 |  |
|  | ZNF746 |  |
|  | PSMB4 |  |
|  | CSRNP1 |  |
|  | REL |  |
|  | LINC00565 |  |
|  | SERPIND1 |  |
|  | ACSL1 |  |
|  | C1orf52 |  |
|  | TCF15 |  |
|  | RRP8 |  |
|  | TNFAIP2 |  |
|  | AFF4 |  |
|  | SSBP3 |  |
|  | FMNL1 |  |
|  | FOLR2 |  |
|  | SEPT9 |  |
|  | MXD1 |  |
|  | RBM38 |  |
|  | RNF19B |  |
|  | RNF139 |  |
|  | PRNP |  |
|  | KIAA0922 |  |
|  | PLAU |  |
|  | SERPINB2 |  |
|  | GABARAPL1 |  |
|  | ARID4B |  |
|  | ST3GAL5 |  |
|  | PELI2 |  |
|  | ZNF136 |  |
|  | MPZ |  |
|  | NKTR |  |
|  | BASP1 |  |
|  | SNRPA1 |  |
|  | FAM217A |  |
|  | TLE4 |  |
|  | GAB1 |  |
|  | PRDM2 |  |
|  | ARHGDIB |  |
|  | IER2 |  |
|  | BRD7P3 |  |
|  | RAB35 |  |
|  | RPL18 |  |
|  | VSIG2 |  |
|  | DYRK2 |  |
|  | LOC101927018 |  |
|  | ADCY1 |  |
|  | GINM1 |  |
|  | AC139100.3 |  |
|  | ICAM1 |  |
|  | 6-Mar |  |
|  | MTPAP |  |
|  | HIST1H4J |  |
|  | PHF1 |  |
|  | SEPN1 |  |
|  | C6orf48 |  |
|  | LOC644090 |  |
|  | TCERG1 |  |
|  | RBBP6 |  |
|  | ZNF92 |  |
|  | TCP11L2 |  |
|  | CTSL |  |
|  | UBE2C |  |
|  | MXI1 |  |
|  | PLEKHB2 |  |
|  | BMF |  |
|  | ALPP |  |
|  | RASGEF1B |  |
|  | ARRB2 |  |
|  | IL15 |  |
|  | TOE1 |  |
|  | MEF2D |  |
|  | MAD2L1BP |  |
|  | PNRC1 |  |
|  | KIAA1432 |  |
|  | ROM1 |  |
|  | TCP10 |  |
|  | GRINA |  |
|  | ZNF134 |  |
|  | SENP5 |  |
|  | VAMP1 |  |
|  | TRAF4 |  |
|  | TAPT1 |  |
|  | SRRT |  |
|  | ABAT |  |
|  | SFT2D3 |  |
|  | ANXA5 |  |
|  | EIF1 |  |
|  | HIST1H1T |  |
|  | ARL4A |  |
|  | FBXO7 |  |
|  | PILRA |  |
|  | FBRS |  |
|  | ENY2 |  |
|  | PDE9A |  |
|  | VIM |  |
|  | RP3-336K20__B.2 |  |
|  | TNKS |  |
|  | CCDC59 |  |
|  | INSIG1 |  |
|  | RELL1 |  |
|  | DDX3X |  |
|  | CPSF6 |  |
|  | HIST1H3C |  |
|  | DNAJB8-AS1 |  |
|  | AX746968 |  |
|  | RNPEP |  |
|  | ZBTB43 |  |
|  | ZBTB18 |  |
|  | FEM1B |  |
|  | SLC25A37 |  |
|  | POLR2J |  |
|  | LRRC48 |  |
|  | KLHL18 |  |
|  | ATP2B1 |  |
|  | CAND2 |  |
|  | AX748292 |  |
|  | PROCR |  |
|  | ERO1LB |  |
|  | GATSL2 |  |
|  | MAP4K3 |  |
|  | PPM1E |  |
|  | SPRYD4 |  |
|  | RNASET2 |  |
|  | CCRL2 |  |
|  | BCLAF1 |  |
|  | TBC1D20 |  |
|  | C3AR1 |  |
|  | COX4I1 |  |
|  | DNAJC1 |  |
|  | SOD2 |  |
|  | VMP1 |  |
|  | ZFP36 |  |
|  | RNF24 |  |
|  | HBEGF |  |
|  | TAF8 |  |
|  | LOC100507217 |  |
|  | LIN7A |  |
|  | TMEM2 |  |
|  | SECISBP2L |  |
|  | HIST1H2BB |  |
|  | CXXC5 |  |
|  | GABPB1 |  |
|  | NRIP1 |  |
|  | RP11-16P6.1 |  |
|  | SERHL2 |  |
|  | TLR1 |  |
|  | LAMB1 |  |
|  | SNRPB2 |  |
|  | SOCS3 |  |
|  | DLL1 |  |
|  | BAK1 |  |
|  | FKBP14 |  |
|  | TEAD3 |  |
|  | G6PC3 |  |
|  | VNN1 |  |
|  | NDE1 |  |
|  | UBXN11 |  |
|  | F5 |  |
|  | CFLAR-AS1 |  |
|  | FNIP1 |  |
|  | IL17C |  |
|  | FCAR |  |
|  | FUBP1 |  |
|  | HMGB1 |  |
|  | RCBTB2 |  |
|  | CLEC4D |  |
|  | ZNF496 |  |
|  | DDX59 |  |
|  | FLJ11710 |  |
|  | UNKL |  |
|  | STRN4 |  |
|  | POM121C |  |
|  | DLST |  |
|  | DEPTOR |  |
|  | PPP1R17 |  |
|  | APH1A |  |
|  | FOXO1 |  |
|  | ARHGEF7 |  |
|  | RNPC3 |  |
|  | LINC00620 |  |
|  | IFT57 |  |
|  | RP11-425D10.10 |  |
|  | CLIC4 |  |
|  | ASAH1 |  |
|  | SPTBN1 |  |
|  | BNIP3L |  |
|  | HP |  |
|  | B3GNT5 |  |
|  | SZRD1 |  |
|  | RP11-473I1.9 |  |
|  | PPTC7 |  |
|  | TIMP1 |  |
|  | SMARCD3 |  |
|  | USP30 |  |
|  | DDX6 |  |
|  | CMTM4 |  |
|  | EBLN3 |  |
|  | SH3BP1 |  |
|  | ZFHX3 |  |
|  | B4GALT1 |  |
|  | IL17RA |  |
|  | ARHGAP42 |  |
|  | C1QB |  |
|  | CIB2 |  |
|  | SOX21 |  |
|  | EMR3 |  |
|  | KCNIP2 |  |
|  | TNIK |  |
|  | STK17B |  |
|  | CD34 |  |
|  | AC083843.1 |  |
|  | KMT2E |  |
|  | ZNF814 |  |
|  | RBM25 |  |
|  | RAB5C |  |
|  | TMED2 |  |
|  | SMARCB1 |  |
|  | RP11-508N22.12 |  |
|  | BHLHE40 |  |
|  | SULT1A2 |  |
|  | FNDC8 |  |
|  | PXMP2 |  |
|  | DEFA5 |  |
|  | SIK1 |  |
|  | TMEM198 |  |
|  | UBASH3B |  |
|  | UBXN4 |  |
|  | KLF6 |  |
|  | CABP5 |  |
|  | ACVR1B |  |
|  | INO80B |  |
|  | LOC728445 |  |
|  | PLXNB2 |  |
|  | C10orf11 |  |
|  | DUSP2 |  |
|  | TPT1-AS1 |  |
|  | FBXL5 |  |
|  | AKAP13 |  |
|  | ANKRD10-IT1 |  |
|  | FEZ1 |  |
|  | RICTOR |  |
|  | RC3H1 |  |
|  | CNPY3 |  |
|  | MVD |  |
|  | ADNP2 |  |
|  | SMARCC2 |  |
|  | C5AR2 |  |
|  | GLIPR2 |  |
|  | TBL1X |  |
|  | TNFRSF10D |  |
|  | ABHD8 |  |
|  | MAN2A2 |  |
|  | MSI2 |  |
|  | SERTAD2 |  |
|  | ZNF467 |  |
|  | TRAPPC1 |  |
|  | RAB3D |  |
|  | FKBP8 |  |
|  | RP11-400N9.1 |  |
|  | LOC338667 |  |
|  | FOXP1 |  |
|  | HMX1 |  |
|  | EPN3 |  |
|  | NLN |  |
|  | SIAH1 |  |
|  | VEGFA |  |
|  | DDX24 |  |
|  | TFDP2 |  |
|  | FAM91A1 |  |
|  | PTP4A1 |  |
|  | LOC142937 |  |
|  | PPP1R15A |  |
|  | TTF1 |  |
|  | NID1 |  |
|  | G3BP2 |  |
|  | FOXN2 |  |
|  | NR2F1 |  |
|  | ANXA2P2 |  |
|  | MOS |  |
|  | CST1 |  |
|  | RNF168 |  |
|  | GRIA3 |  |
|  | CTIF |  |
|  | UBE2Q1 |  |
|  | MSLN |  |
|  | HES6 |  |
|  | TXNIP |  |
|  | RASSF5 |  |
|  | UBE2N |  |
|  | PRSS12 |  |
|  | NSFL1C |  |
|  | MED25 |  |
|  | KCNN4 |  |
|  | RP1-263J7.2 |  |
|  | PCYOX1 |  |
|  | RIT1 |  |
|  | SFSWAP |  |
|  | ZC3H15 |  |
|  | FYCO1 |  |
|  | ICOSLG |  |
|  | JOSD1 |  |
|  | F2 |  |
|  | PABPN1 |  |
|  | PI4KB |  |
|  | QTRTD1 |  |
|  | LSM14B |  |
|  | DVL2 |  |
|  | KAT6B |  |
|  | WDPCP |  |
|  | EPOR |  |
|  | SOX12 |  |
|  | NFYA |  |
|  | RAPGEF2 |  |
|  | BAMBI |  |
|  | HIST1H2AK |  |
|  | AKIRIN1 |  |
|  | WASL |  |
|  | CCL20 |  |
|  | GOLIM4 |  |
|  | SIN3B |  |
|  | SLC35D1 |  |
|  | HNRNPL |  |
|  | COG3 |  |
|  | ADH1A |  |
|  | DST |  |
|  | HEY1 |  |
|  | CTB-12A17.3 |  |
|  | IER5L |  |
|  | FAHD2CP |  |
|  | SYDE1 |  |
|  | BLVRA |  |
|  | TRIM62 |  |
|  | CCDC159 |  |
|  | YWHAZ |  |
|  | ARFGEF2 |  |
|  | BCAS2 |  |
|  | SAMD4A |  |
|  | MAPKAPK2 |  |
|  | PCIF1 |  |
|  | MAD2L2 |  |
|  | LRG1 |  |
|  | RAP2A |  |
|  | RPL8 |  |
|  | REG1P |  |
|  | LOC101927292 |  |
|  | WDR26 |  |
|  | ARL4D |  |
|  | TYMS |  |
|  | BRF1 |  |
|  | KCNC3 |  |
|  | IDH3B |  |
|  | C19orf33 |  |
|  | CAMTA1 |  |
|  | TLN1 |  |
|  | NPCDR1 |  |
|  | MAP2K5 |  |
|  | MFGE8 |  |
|  | ITPK1 |  |
|  | SEC14L3 |  |
|  | EVC |  |
|  | AAED1 |  |
|  | MTMR11 |  |
|  | SAR1A |  |
|  | NLRP1 |  |
|  | MIR4313 |  |
|  | GFRA2 |  |
|  | LOC339539 |  |
|  | ABL1 |  |
|  | DIP2C |  |
|  | WDR1 |  |
|  | GABPB1-AS1 |  |
|  | GPS1 |  |
|  | HID1 |  |
|  | LRRC75B |  |
|  | BPIFA1 |  |
|  | NGDN |  |
|  | CRYBB1 |  |
|  | CTNNAP1 |  |
|  | MACF1 |  |
|  | FAM189A1 |  |
|  | SPIRE1 |  |
|  | SCMH1 |  |
|  | PRRG4 |  |
|  | COX5B |  |
|  | MLC1 |  |
|  | CAPRIN1 |  |
|  | CRMP1 |  |
|  | USP38 |  |
|  | LOC102724814 |  |
|  | PLIN1 |  |
|  | NAAA |  |
|  | LOC101929167 |  |
|  | CADM4 |  |
|  | SLC4A5 |  |
|  | EIF5A |  |
|  | MTSS1L |  |
|  | LOC100996286 |  |
|  | KAAG1 |  |
|  | SZT2 |  |
|  | TLR3 |  |
|  | COL18A1-AS1 |  |
|  | VRK3 |  |
|  | AMN |  |
|  | MYEOV |  |
|  | PRPF38A |  |
|  | THBS3 |  |
|  | LOC101927051 |  |
|  | RP11-513N24.1 |  |
|  | ELOVL5 |  |
|  | HTR6 |  |
|  | MGC57346 |  |
|  | DCTN4 |  |
|  | SELT |  |
|  | TM6SF2 |  |
|  | SLC6A6 |  |
|  | KIF18B |  |
|  | EBF3 |  |
|  | PITX3 |  |
|  | OSR2 |  |
|  | SEZ6L2 |  |
|  | LOC102723918 |  |
|  | LOC101928132 |  |
|  | PON3 |  |
|  | HIST1H4D |  |
|  | ZNF736 |  |
|  | GDF2 |  |
|  | ATP11A |  |
|  | PCDHGC5 |  |
|  | DNAJB5 |  |
|  | ZNF292 |  |
|  | BRD2 |  |
|  | TIMM17A |  |
|  | PRR7 |  |
|  | DHX9 |  |
|  | GRPEL2 |  |
|  | RP11-410D17.2 |  |
|  | CCNJ |  |
|  | MISP |  |
|  | GNAI2 |  |
|  | MORF4L1 |  |
|  | STXBP2 |  |
|  | OR1Q1 |  |
|  | LINC01428 |  |
|  | CRNN |  |
|  | PP13 |  |
|  | DKKL1 |  |
|  | MGEA5 |  |
|  | SFXN5 |  |
|  | AVPI1 |  |
|  | SLC6A12 |  |
|  | EAPP |  |
|  | FAM161A |  |
|  | DRAP1 |  |
|  | TAOK2 |  |
|  | APOL4 |  |
|  | COLGALT1 |  |
|  | HIC1 |  |
|  | CRABP2 |  |
|  | TFE3 |  |
|  | CASP2 |  |
|  | LOC101927967 |  |
|  | PXN |  |
|  | SPATA17 |  |
|  | XPNPEP2 |  |
|  | CTAG2 |  |
|  | SH3GLB1 |  |
|  | SLC25A36 |  |
|  | AMD1 |  |
|  | LOC100132005 |  |
|  | LOC101929114 |  |
|  | C1orf200 |  |
|  | EVI5L |  |
|  | TSTA3 |  |
|  | PC |  |
|  | LINC00337 |  |
|  | RSL24D1 |  |
|  | PLXND1 |  |
|  | PHF3 |  |
|  | CCDC71L |  |
|  | WBP4 |  |
|  | ROCK1 |  |
|  | ZNF697 |  |
|  | HOOK1 |  |
|  | TBX6 |  |
|  | VPS13B |  |
|  | CRCP |  |
|  | PLEKHM2 |  |
|  | ZC3H7A |  |
|  | KCNQ1DN |  |
|  | GIPR |  |
|  | IDH3G |  |
|  | EIF5 |  |
|  | HEATR1 |  |
|  | KCNQ1 |  |
|  | RBAK |  |
|  | NONO |  |
|  | GJA4 |  |
|  | ZC3HAV1 |  |
|  | FLJ46026 |  |
|  | FAM64A |  |
|  | RNF26 |  |
|  | GRIK5 |  |
|  | CYP2A13 |  |
|  | ASPSCR1 |  |
|  | SETD8 |  |
|  | SHISA4 |  |
|  | DHRS12 |  |
|  | TMEM190 |  |
|  | CLPS |  |
|  | UVRAG |  |
|  | LINC01398 |  |
|  | UBC |  |
|  | ZRANB2 |  |
|  | CASC17 | down |
|  | OR7E156P |  |
|  | PTPRE |  |
|  | PQLC1 |  |
|  | FHOD1 |  |
|  | UBQLN4 |  |
|  | PTCRA |  |
|  | GRIA4 |  |
|  | ZNF605 |  |
|  | TJAP1 |  |
|  | GSTK1 |  |
|  | MFSD11 |  |
|  | TXNDC15 |  |
|  | LINC00470 |  |
|  | TAF2 |  |
|  | TM4SF4 |  |
|  | TMEM27 |  |
|  | CBR4 |  |
|  | ENSA |  |
|  | JPH1 |  |
|  | CARS |  |
|  | THTPA |  |
|  | SMIM2 |  |
|  | IPO9 |  |
|  | NEXN-AS1 |  |
|  | WDR44 |  |
|  | RMDN3 |  |
|  | DALRD3 |  |
|  | OLIG3 |  |
|  | DDAH2 |  |
|  | RNPS1 |  |
|  | CNTN5 |  |
|  | PPID |  |
|  | CLCA3P |  |
|  | HERC1 |  |
|  | IWS1 |  |
|  | BRAT1 |  |
|  | HARS2 |  |
|  | KRTAP7-1 |  |
|  | NKX2-2 |  |
|  | EIF3A |  |
|  | MRPS27 |  |
|  | TXNL1 |  |
|  | EARS2 |  |
|  | DHX58 |  |
|  | LOC101928335 |  |
|  | HRAS |  |
|  | PUS7L |  |
|  | ENOX1 |  |
|  | IFT172 |  |
|  | ICK |  |
|  | JAKMIP2-AS1 |  |
|  | C12orf65 |  |
|  | PHF20 |  |
|  | GLRB |  |
|  | CASP9 |  |
|  | ARF4 |  |
|  | UTP3 |  |
|  | MTIF3 |  |
|  | PPP1R18 |  |
|  | TMEM26 |  |
|  | KIF1B |  |
|  | NRDE2 |  |
|  | RPS24 |  |
|  | CYP2E1 |  |
|  | CHKB |  |
|  | ZSCAN30 |  |
|  | CEP57L1 |  |
|  | FTSJ3 |  |
|  | KIAA1244 |  |
|  | ALKBH6 |  |
|  | OR7A5 |  |
|  | VPS28 |  |
|  | RBX1 |  |
|  | TBC1D9B |  |
|  | CHKA |  |
|  | SLC25A12 |  |
|  | COMMD6 |  |
|  | POLE |  |
|  | ATP5E |  |
|  | RABEPK |  |
|  | WDR6 |  |
|  | POGZ |  |
|  | MSL3 |  |
|  | EXOSC8 |  |
|  | SLC4A3 |  |
|  | BTN2A1 |  |
|  | UQCC1 |  |
|  | SMC4 |  |
|  | TRIP12 |  |
|  | TAF6 |  |
|  | EPG5 |  |
|  | USP36 |  |
|  | KIAA0513 |  |
|  | PWWP2A |  |
|  | LOC285766 |  |
|  | KRTAP13-1 |  |
|  | LRCH2 |  |
|  | KIAA0040 |  |
|  | IPO13 |  |
|  | LOC148413 |  |
|  | STRN3 |  |
|  | GTF2F1 |  |
|  | SMEK2 |  |
|  | TIRAP |  |
|  | NIF3L1 |  |
|  | MAP9 |  |
|  | CYB561A3 |  |
|  | NANS |  |
|  | UBE2W |  |
|  | SART3 |  |
|  | NUP107 |  |
|  | F11R |  |
|  | ADCK1 |  |
|  | ZNF506 |  |
|  | NDUFB2 |  |
|  | MON1A |  |
|  | NME3 |  |
|  | KANSL1 |  |
|  | MYD88 |  |
|  | STX12 |  |
|  | SAP30BP |  |
|  | FH |  |
|  | TASP1 |  |
|  | MMP8 |  |
|  | SMARCA4 |  |
|  | COX5A |  |
|  | GPBP1 |  |
|  | UBA3 |  |
|  | ABRA |  |
|  | RTCB |  |
|  | SLC30A7 |  |
|  | NMT2 |  |
|  | DHDDS |  |
|  | LOC643733 |  |
|  | RNF6 |  |
|  | PPP6R3 |  |
|  | LINS |  |
|  | POMGNT1 |  |
|  | TBCEL |  |
|  | FGFR1OP |  |
|  | FAM46D |  |
|  | TPRN |  |
|  | OOSP2 |  |
|  | CIPC |  |
|  | RPL7L1 |  |
|  | GRK7 |  |
|  | PDCL |  |
|  | POLR3D |  |
|  | CTSB |  |
|  | EPT1 |  |
|  | TMEM184B |  |
|  | DIAPH2-AS1 |  |
|  | CBFA2T3 |  |
|  | AK021537 |  |
|  | RGS19 |  |
|  | PSMD7 |  |
|  | POLR2I |  |
|  | AK098263 |  |
|  | PPCS |  |
|  | EIF1B |  |
|  | GORASP2 |  |
|  | SLC17A5 |  |
|  | ZKSCAN5 |  |
|  | DCBLD1 |  |
|  | DCUN1D5 |  |
|  | GTF3C3 |  |
|  | AFTPH |  |
|  | RP11-258C19.7 |  |
|  | ZHX2 |  |
|  | COLEC10 |  |
|  | MON1B |  |
|  | C11orf1 |  |
|  | BC053951 |  |
|  | 9-Mar |  |
|  | CD97 |  |
|  | PKIG |  |
|  | VPS33A |  |
|  | MCCC1 |  |
|  | MORC4 |  |
|  | MYCL |  |
|  | LOC101927829 |  |
|  | CRTC3 |  |
|  | VPS36 |  |
|  | TCEAL7 |  |
|  | SACM1L |  |
|  | ATP6AP1 |  |
|  | ZNF826P |  |
|  | PIP5K1C |  |
|  | FBXL15 |  |
|  | N4BP1 |  |
|  | SLC25A16 |  |
|  | HMGCL |  |
|  | SOCS4 |  |
|  | MLLT1 |  |
|  | MGA |  |
|  | GCC2 |  |
|  | SLAIN2 |  |
|  | UBA2 |  |
|  | NUBP2 |  |
|  | MKL2 |  |
|  | MAGOHB |  |
|  | TMEM136 |  |
|  | TXN |  |
|  | SPSB2 |  |
|  | GTF2E2 |  |
|  | BRCC3 |  |
|  | CBR3 |  |
|  | HDGFRP2 |  |
|  | ANO8 |  |
|  | PELP1 |  |
|  | PITPNB |  |
|  | BLMH |  |
|  | EXOSC4 |  |
|  | HHAT |  |
|  | C2CD3 |  |
|  | TGIF2 |  |
|  | RDH11 |  |
|  | FAM192A |  |
|  | CSF2RA |  |
|  | RPARP-AS1 |  |
|  | VPS72 |  |
|  | SSX2B |  |
|  | TPH1 |  |
|  | ACAD9 |  |
|  | TMEM110 |  |
|  | AHCY |  |
|  | MYRIP |  |
|  | XPO5 |  |
|  | LINC00493 |  |
|  | PNPO |  |
|  | ZNF526 |  |
|  | AKR1A1 |  |
|  | CAPN1 |  |
|  | TEX30 |  |
|  | ZNF117 |  |
|  | DCLRE1C |  |
|  | ITPKC |  |
|  | IFFO1 |  |
|  | TOB2 |  |
|  | OR7E37P |  |
|  | LINC01432 |  |
|  | RMDN1 |  |
|  | PIGL |  |
|  | TNFAIP8L1 |  |
|  | ANAPC13 |  |
|  | FADD |  |
|  | TFB1M |  |
|  | CAP1 |  |
|  | GPATCH11 |  |
|  | GTPBP4 |  |
|  | CAPZA1 |  |
|  | CC2D2A |  |
|  | ZNF486 |  |
|  | PSMC3IP |  |
|  | BTN1A1 |  |
|  | PHF11 |  |
|  | TDG |  |
|  | NPRL3 |  |
|  | SETBP1 |  |
|  | CREB3L2 |  |
|  | MRPS16 |  |
|  | FAM206A |  |
|  | ZSCAN31 |  |
|  | PFKFB2 |  |
|  | TAS2R4 |  |
|  | SH3BP5L |  |
|  | ZNF142 |  |
|  | PTPN20B |  |
|  | CHSY1 |  |
|  | ITPR1 |  |
|  | HOMER1 |  |
|  | PRPF8 |  |
|  | LLNLR-246C6.1 |  |
|  | GABPB2 |  |
|  | TUBGCP4 |  |
|  | DNMT1 |  |
|  | MTFP1 |  |
|  | DUSP11 |  |
|  | EIF4ENIF1 |  |
|  | USP8 |  |
|  | TMEM168 |  |
|  | RUFY1 |  |
|  | COQ10A |  |
|  | TMEM187 |  |
|  | SLC5A9 |  |
|  | C19orf24 |  |
|  | PCED1A |  |
|  | E2F3 |  |
|  | MYO1E |  |
|  | DCK |  |
|  | AP5S1 |  |
|  | RNF123 |  |
|  | TMEM186 |  |
|  | TNFSF13 |  |
|  | FAM160A2 |  |
|  | SUMF2 |  |
|  | BAZ1A |  |
|  | MEAF6 |  |
|  | TUBG2 |  |
|  | EFHC1 |  |
|  | C9orf69 |  |
|  | ZNF48 |  |
|  | ATP6V1C1 |  |
|  | MAVS |  |
|  | NIPAL3 |  |
|  | TMEM5 |  |
|  | LEMD3 |  |
|  | MRPL50 |  |
|  | LPIN2 |  |
|  | CEP162 |  |
|  | ZMIZ1-AS1 |  |
|  | ZNF696 |  |
|  | PP12719 |  |
|  | REV3L |  |
|  | PSMC4 |  |
|  | SPG11 |  |
|  | ABCB10 |  |
|  | TMEM165 |  |
|  | CORO2B |  |
|  | NEB |  |
|  | STOX1 |  |
|  | ZBTB4 |  |
|  | IFI16 |  |
|  | CEP104 |  |
|  | FASTKD3 |  |
|  | GAS2 |  |
|  | NR2C2AP |  |
|  | CHD9 |  |
|  | RBM42 |  |
|  | SNX5 |  |
|  | GPM6B |  |
|  | ATP5G1 |  |
|  | LPIN1 |  |
|  | CTB-176F20.3 |  |
|  | LOC100507156 |  |
|  | SLC39A1 |  |
|  | FAT1 |  |
|  | ZNF84 |  |
|  | LILRA4 |  |
|  | METTL15 |  |
|  | SETD3 |  |
|  | COMMD10 |  |
|  | MPLKIP |  |
|  | OARD1 |  |
|  | FMO2 |  |
|  | RAB11A |  |
|  | AGL |  |
|  | RPS27L |  |
|  | HSD17B1 |  |
|  | ALAS1 |  |
|  | GMPPA |  |
|  | SMC1A |  |
|  | SPAG16 |  |
|  | ZNF264 |  |
|  | TDRKH |  |
|  | DDX26B |  |
|  | HIVEP2 |  |
|  | FARSA |  |
|  | RBMX2 |  |
|  | CHST12 |  |
|  | ZNF706 |  |
|  | ZNF180 |  |
|  | LRRN1 |  |
|  | RP11-285F7.2 |  |
|  | RBM10 |  |
|  | TGOLN2 |  |
|  | GNS |  |
|  | DCLRE1A |  |
|  | ZNF721 |  |
|  | RGL2 |  |
|  | ZZZ3 |  |
|  | MORC3 |  |
|  | TRMT12 |  |
|  | AGPAT4-IT1 |  |
|  | WDR81 |  |
|  | CALCRL |  |
|  | PPAT |  |
|  | MLYCD |  |
|  | RNF214 |  |
|  | LOC100506730 |  |
|  | RGP1 |  |
|  | ITSN2 |  |
|  | DROSHA |  |
|  | C17orf85 |  |
|  | UBAC2 |  |
|  | NPTN |  |
|  | DHRS1 |  |
|  | SNX6 |  |
|  | ZDHHC16 |  |
|  | ITGAV |  |
|  | PPM1K |  |
|  | BTBD7 |  |
|  | HIATL1 |  |
|  | COL4A4 |  |
|  | SOS1 |  |
|  | KATNAL1 |  |
|  | MRPS36 |  |
|  | SUCLG1 |  |
|  | SPCS1 |  |
|  | MRPL13 |  |
|  | MRPS35 |  |
|  | ACOT9 |  |
|  | SLC30A9 |  |
|  | FILIP1 |  |
|  | ZNF45 |  |
|  | TMEM38B |  |
|  | TUBG1 |  |
|  | ANKIB1 |  |
|  | TECPR1 |  |
|  | ANKMY2 |  |
|  | CMC4 |  |
|  | VARS2 |  |
|  | C6orf203 |  |
|  | CUEDC2 |  |
|  | TBC1D10B |  |
|  | STK11IP |  |
|  | EXOC6 |  |
|  | PAF1 |  |
|  | ITGB3BP |  |
|  | BC048141 |  |
|  | RP11-480D4.6 |  |
|  | WIBG |  |
|  | OTUD6B-AS1 |  |
|  | DFFB |  |
|  | MAGIX |  |
|  | CSRNP2 |  |
|  | KIF20B |  |
|  | ZNF518B |  |
|  | ARMCX5 |  |
|  | SLC10A3 |  |
|  | DPH5 |  |
|  | DTWD1 |  |
|  | NDUFB4 |  |
|  | C1orf131 |  |
|  | CC2D1B |  |
|  | CYP4V2 |  |
|  | RP11-54K16.2 |  |
|  | KLC2 |  |
|  | ADK |  |
|  | TMOD2 |  |
|  | TOMM5 |  |
|  | SUPT16H |  |
|  | GNL2 |  |
|  | PAICS |  |
|  | COG4 |  |
|  | ZMAT3 |  |
|  | MAD2L1 |  |
|  | LYSMD2 |  |
|  | EP300 |  |
|  | ELL3 |  |
|  | TIA1 |  |
|  | FBXO8 |  |
|  | GINS3 |  |
|  | FAM122A |  |
|  | PWP1 |  |
|  | ZNF678 |  |
|  | HEATR2 |  |
|  | MTERF3 |  |
|  | KLHL34 |  |
|  | RBM12 |  |
|  | UBE2L3 |  |
|  | DHRS13 |  |
|  | PEX5L |  |
|  | TALDO1 |  |
|  | PHF13 |  |
|  | C6orf47 |  |
|  | CWC27 |  |
|  | DCP1B |  |
|  | GPD1L |  |
|  | SCN9A |  |
|  | UBE2E2 |  |
|  | DHRS4-AS1 |  |
|  | CD3EAP |  |
|  | GBAP1 |  |
|  | PIGF |  |
|  | GTF2A2 |  |
|  | RPP40 |  |
|  | GATAD1 |  |
|  | ZFYVE19 |  |
|  | SCAPER |  |
|  | SMARCD2 |  |
|  | HERC6 |  |
|  | B4GALT4 |  |
|  | AHCYL1 |  |
|  | PIH1D1 |  |
|  | RBM23 |  |
|  | GPANK1 |  |
|  | EIF2B4 |  |
|  | CENPJ |  |
|  | SEC23IP |  |
|  | UST |  |
|  | CAMKMT |  |
|  | SPDL1 |  |
|  | TMEM86A |  |
|  | FAM127A |  |
|  | LSM8 |  |
|  | THAP2 |  |
|  | DNLZ |  |
|  | ATE1 |  |
|  | PRKCI |  |
|  | LINC00052 |  |
|  | LINC00957 |  |
|  | THAP6 |  |
|  | FZD6 |  |
|  | ZNHIT6 |  |
|  | SDHAF1 |  |
|  | DNAJC12 |  |
|  | CPNE5 |  |
|  | PKD2 |  |
|  | NDUFAB1 |  |
|  | ITGA10 |  |
|  | DIP2B |  |
|  | CCT6A |  |
|  | HIRIP3 |  |
|  | TBC1D19 |  |
|  | EVI2A |  |
|  | C16orf54 |  |
|  | GUCY2D |  |
|  | SLC25A46 |  |
|  | TXLNB |  |
|  | TDP2 |  |
|  | HADH |  |
|  | MED7 |  |
|  | CRLS1 |  |
|  | FAIM3 |  |
|  | TSHZ1 |  |
|  | PCNT |  |
|  | ZNF575 |  |
|  | ZCCHC8 |  |
|  | MYOM2 |  |
|  | MSTO1 |  |
|  | YBEY |  |
|  | CERK |  |
|  | CCDC92 |  |
|  | POP5 |  |
|  | PARP1 |  |
|  | CD38 |  |
|  | ZMYM1 |  |
|  | RALA |  |
|  | PDSS2 |  |
|  | CCT6B |  |
|  | LYSMD3 |  |
|  | ZNF521 |  |
|  | CDC73 |  |
|  | BC038205 |  |
|  | LINC00996 |  |
|  | TM9SF1 |  |
|  | LOC101927164 |  |
|  | NOSIP |  |
|  | PGM1 |  |
|  | IRF7 |  |
|  | ZNF473 |  |
|  | SETD6 |  |
|  | CERS2 |  |
|  | UNC119B |  |
|  | PQLC2 |  |
|  | PAN2 |  |
|  | DYRK4 |  |
|  | PPCDC |  |
|  | CEP192 |  |
|  | TBL2 |  |
|  | NT5DC1 |  |
|  | P2RY6 |  |
|  | COPS7A |  |
|  | SLC38A6 |  |
|  | HEATR5A |  |
|  | STAU2 |  |
|  | DAPK2 |  |
|  | TRAF3IP3 |  |
|  | ICE1 |  |
|  | KRTCAP2 |  |
|  | TGDS |  |
|  | CALCOCO1 |  |
|  | TMEM14C |  |
|  | TNFSF13B |  |
|  | TINF2 |  |
|  | RNF135 |  |
|  | LOC101928504 |  |
|  | RAB29 |  |
|  | CXCR1 |  |
|  | IDH1 |  |
|  | TMEM218 |  |
|  | PLRG1 |  |
|  | ARHGAP18 |  |
|  | HMGCR |  |
|  | XPC |  |
|  | TMEM156 |  |
|  | TBC1D13 |  |
|  | ZNF257 |  |
|  | RNASEH2B |  |
|  | VPS37A |  |
|  | FKBP15 |  |
|  | GOT2 |  |
|  | HGH1 |  |
|  | TLR7 |  |
|  | LOC100132891 |  |
|  | MNAT1 |  |
|  | CCDC127 |  |
|  | METTL23 |  |
|  | ERCC1 |  |
|  | FBXO28 |  |
|  | TMEM170A |  |
|  | NDUFB5 |  |
|  | LIPT1 |  |
|  | JRKL |  |
|  | NOP14 |  |
|  | ZSCAN21 |  |
|  | NSL1 |  |
|  | MFSD5 |  |
|  | HPS6 |  |
|  | C1orf112 |  |
|  | ZFP62 |  |
|  | OPN3 |  |
|  | LINC00847 |  |
|  | LOC100507535 |  |
|  | TREML2 |  |
|  | ATR |  |
|  | MRPS9 |  |
|  | C15orf41 |  |
|  | TMEM98 |  |
|  | CPSF3 |  |
|  | WBP5 |  |
|  | TRIM32 |  |
|  | PARS2 |  |
|  | TIAM2 |  |
|  | OIP5 |  |
|  | FAM3C |  |
|  | SCOC |  |
|  | CTC1 |  |
|  | TRIM68 |  |
|  | HSPH1 |  |
|  | ARPC1B |  |
|  | DET1 |  |
|  | ASUN |  |
|  | KLHDC10 |  |
|  | SLFN11 |  |
|  | DRAM2 |  |
|  | ZNF222 |  |
|  | FLJ38773 |  |
|  | POT1 |  |
|  | TUSC1 |  |
|  | C11orf49 |  |
|  | TIMM21 |  |
|  | HSPBAP1 |  |
|  | EPRS |  |
|  | LAMP2 |  |
|  | TRIP4 |  |
|  | COMMD7 |  |
|  | ENDOG |  |
|  | USP37 |  |
|  | LINC00909 |  |
|  | CCDC86 |  |
|  | NCOA5 |  |
|  | TVP23B |  |
|  | AX748339 |  |
|  | EXOC4 |  |
|  | ZNF717 |  |
|  | UNG |  |
|  | MRPL48 |  |
|  | MOB3C |  |
|  | GRAMD3 |  |
|  | RNF34 |  |
|  | TMEM60 |  |
|  | TDRD7 |  |
|  | MREG |  |
|  | GNG10 |  |
|  | RAB1B |  |
|  | CTC-444N24.11 |  |
|  | DCP2 |  |
|  | RGS7 |  |
|  | NUDCD1 |  |
|  | ERGIC2 |  |
|  | PSMB10 |  |
|  | TTC4 |  |
|  | TOR1B |  |
|  | JAGN1 |  |
|  | CBLB |  |
|  | TMEM53 |  |
|  | BNIP3 |  |
|  | LOC100129461 |  |
|  | SF3B4 |  |
|  | SRP54 |  |
|  | SCAMP5 |  |
|  | ZNF75A |  |
|  | CD180 |  |
|  | DYNC2LI1 |  |
|  | CTC-471J1.2 |  |
|  | C10orf32 |  |
|  | GPR65 |  |
|  | MEF2C |  |
|  | TRAPPC11 |  |
|  | TSPYL5 |  |
|  | TTC13 |  |
|  | GRB14 |  |
|  | IPO11 |  |
|  | RPP25L |  |
|  | C19orf12 |  |
|  | TSEN2 |  |
|  | CHMP5 |  |
|  | TTC27 |  |
|  | TXNDC9 |  |
|  | GDPD3 |  |
|  | MMS19 |  |
|  | LOC283745 |  |
|  | SIT1 |  |
|  | NHLRC2 |  |
|  | NVL |  |
|  | TNFAIP8L2 |  |
|  | ARCN1 |  |
|  | SMARCAL1 |  |
|  | PRKXP1 |  |
|  | PRR5L |  |
|  | SECTM1 |  |
|  | PTPLAD1 |  |
|  | MGAT2 |  |
|  | AVEN |  |
|  | UPF3B |  |
|  | NLK |  |
|  | C7orf55 |  |
|  | RCCD1 |  |
|  | HINFP |  |
|  | NUP37 |  |
|  | MGC16275 |  |
|  | UBTD2 |  |
|  | LOC158960 |  |
|  | C5orf15 |  |
|  | CENPQ |  |
|  | NDUFAF4 |  |
|  | TRMT13 |  |
|  | FAM134C |  |
|  | TRIM14 |  |
|  | STAMBPL1 |  |
|  | LOC157562 |  |
|  | PPP3CB |  |
|  | AL133493.2 |  |
|  | MSMO1 |  |
|  | XK |  |
|  | ELMO1 |  |
|  | BBS10 |  |
|  | POP7 |  |
|  | GUCY1A3 |  |
|  | MTX2 |  |
|  | LOC101929774 |  |
|  | ZFYVE21 |  |
|  | ZSCAN29 |  |
|  | LMBRD2 |  |
|  | CETN3 |  |
|  | DOCK8 |  |
|  | TBCA |  |
|  | PYROXD2 |  |
|  | CREB3L4 |  |
|  | TAF15 |  |
|  | GLT8D1 |  |
|  | FBXW2 |  |
|  | ACP6 |  |
|  | IGIP |  |
|  | MSRB1 |  |
|  | PREB |  |
|  | DNAJC13 |  |
|  | NDC80 |  |
|  | CSK |  |
|  | MINPP1 |  |
|  | NUP43 |  |
|  | CRYM |  |
|  | TP53RK |  |
|  | FAM50B |  |
|  | UBLCP1 |  |
|  | ECT2 |  |
|  | AK097453 |  |
|  | RNF111 |  |
|  | ZNF688 |  |
|  | ERI2 |  |
|  | MPHOSPH8 |  |
|  | LSM10 |  |
|  | KIAA1024 |  |
|  | CXCR3 |  |
|  | GSKIP |  |
|  | WDR36 |  |
|  | WBP1L |  |
|  | YEATS4 |  |
|  | FTCDNL1 |  |
|  | KLHDC1 |  |
|  | PARG |  |
|  | FITM2 |  |
|  | MRFAP1L1 |  |
|  | AKR1C3 |  |
|  | DNAH7 |  |
|  | NCOA7 |  |
|  | TBX19 |  |
|  | RP11-350F4.2 |  |
|  | VBP1 |  |
|  | ACTL6A |  |
|  | TRABD2A |  |
|  | LEPREL1 |  |
|  | C2orf44 |  |
|  | RPA3OS |  |
|  | PAQR8 |  |
|  | PRIMPOL |  |
|  | PIGV |  |
|  | OXR1 |  |
|  | ARHGAP30 |  |
|  | ARL6IP6 |  |
|  | ELP3 |  |
|  | CTSO |  |
|  | ACTR6 |  |
|  | AK6 |  |
|  | AMMECR1 |  |
|  | GPN3 |  |
|  | ZBTB34 |  |
|  | RAB3GAP1 |  |
|  | CMC1 |  |
|  | BAZ2B |  |
|  | ZW10 |  |
|  | DCTPP1 |  |
|  | RNF20 |  |
|  | LY96 |  |
|  | SCYL3 |  |
|  | MRPS17 |  |
|  | LINC01355 |  |
|  | ZNF175 |  |
|  | ZNF468 |  |
|  | LACTB2 |  |
|  | RACGAP1 |  |
|  | ATG4C |  |
|  | CMTR2 |  |
|  | CCDC176 |  |
|  | LAGE3 |  |
|  | ADRB2 |  |
|  | TBCK |  |
|  | DNAJC5 |  |
|  | SS18L1 |  |
|  | P2RY14 |  |
|  | SNX27 |  |
|  | C6orf211 |  |
|  | HECTD3 |  |
|  | LINC01393 |  |
|  | PMEPA1 |  |
|  | FTO |  |
|  | HCCS |  |
|  | CCR5 |  |
|  | SUV420H1 |  |
|  | IL6R |  |
|  | GPR160 |  |
|  | KMO |  |
|  | NRTN |  |
|  | ANKRD46 |  |
|  | TMEM69 |  |
|  | RMI1 |  |
|  | ZNF775 |  |
|  | ZNF559 |  |
|  | METTL18 |  |
|  | MX2 |  |
|  | MCCC2 |  |
|  | AL833181 |  |
|  | SPIB |  |
|  | CTD-2124B8.2 |  |
|  | NICN1 |  |
|  | CYB5B |  |
|  | MINOS1P1 |  |
|  | GNPAT |  |
|  | PFAS |  |
|  | FAM179B |  |
|  | SLC35F1 |  |
|  | ATG4A |  |
|  | PARP12 |  |
|  | PTPRO |  |
|  | RAB38 |  |
|  | LEAP2 |  |
|  | MFAP1 |  |
|  | LINC00998 |  |
|  | TEFM |  |
|  | METTL13 |  |
|  | TRUB1 |  |
|  | CHM |  |
|  | PSPH |  |
|  | UBE2V2 |  |
|  | USP18 |  |
|  | EXO5 |  |
|  | RNF219 |  |
|  | FIG4 |  |
|  | GTF2E1 |  |
|  | DNER |  |
|  | DYNC1I2 |  |
|  | OSTM1 |  |
|  | WRB |  |
|  | GTF2H1 |  |
|  | TRIM69 |  |
|  | RGS18 |  |
|  | CHD6 |  |
|  | TRMT1L |  |
|  | KANK1 |  |
|  | SYNRG |  |
|  | TNFRSF17 |  |
|  | CCDC102B |  |
|  | C17orf62 |  |
|  | MSRB2 |  |
|  | IRF2 |  |
|  | BTN3A3 |  |
|  | HPS3 |  |
|  | ZNF234 |  |
|  | TMA16 |  |
|  | GMNN |  |
|  | NDUFAF1 |  |
|  | CUZD1 |  |
|  | DDX60 |  |
|  | ZNF189 |  |
|  | CYYR1 |  |
|  | EHHADH |  |
|  | ZKSCAN3 |  |
|  | ZKSCAN4 |  |
|  | B3GALT6 |  |
|  | GOLPH3L |  |
|  | DYNLL1 |  |
|  | DNAJB6 |  |
|  | IFI44L |  |
|  | DNAJA3 |  |
|  | CPOX |  |
|  | CKS2 |  |
|  | HLA-DQA1 |  |
| PBMC | OLFM4 | up |
|  | ARG1 |  |
|  | DEFA1B |  |
|  | CEACAM8 |  |
|  | LCN2 |  |
|  | BPI |  |
|  | HP |  |
|  | ANXA3 |  |
|  | OLR1 |  |
|  | DAAM2 |  |
|  | ITGB3 |  |
|  | CYBRD1 |  |
|  | SH3BGRL2 |  |
|  | MMRN1 |  |
|  | CLEC4E |  |
|  | CTDSPL |  |
|  | PGLYRP1 |  |
|  | ANKRD22 |  |
|  | TPST1 |  |
|  | NAIP |  |
|  | TREML1 |  |
|  | FKBP5 |  |
|  | IL18RAP |  |
|  | SELP |  |
|  | WRB |  |
|  | KIAA1324 |  |
|  | F13A1 |  |
|  | PRKAR2B |  |
|  | PTGS1 |  |
|  | PF4V1 |  |
|  | MMP9 |  |
|  | BMX |  |
|  | ERLIN1 |  |
|  | ITGA2B |  |
|  | GNG11 |  |
|  | GUCY1B3 |  |
|  | LTBP1 |  |
|  | HIST1H1B |  |
|  | ZNF542P |  |
|  | ALOX12 |  |
|  | NLRC4 |  |
|  | BEND2 |  |
|  | SLC37A3 |  |
|  | CDA |  |
|  | TUBB1 |  |
|  | SPARC |  |
|  | GUCY1A3 |  |
|  | CD163 |  |
|  | CYP1B1 |  |
|  | MARC1 |  |
|  | ZNF641 |  |
|  | CRISPLD2 |  |
|  | PF4 |  |
|  | C6orf25 |  |
|  | CLU |  |
|  | FHL1 |  |
|  | MGAM |  |
|  | F5 |  |
|  | IL18 |  |
|  | CMTM5 |  |
|  | ABCA1 |  |
|  | SIPA1L2 |  |
|  | SULT1B1 |  |
|  | PDE5A |  |
|  | PGD |  |
|  | CCDC144A |  |
|  | REPS2 |  |
|  | PADI4 |  |
|  | CEACAM1 |  |
|  | PLSCR1 |  |
|  | SUCNR1 |  |
|  | ROPN1L |  |
|  | LIPH |  |
|  | GPR160 |  |
|  | CASP5 |  |
|  | GMPR |  |
|  | KCNE3 |  |
|  | PAPSS2 |  |
|  | VCL |  |
|  | C1orf198 |  |
|  | GP1BA |  |
|  | SLC40A1 |  |
|  | EHD3 |  |
|  | TLR2 |  |
|  | PGRMC1 |  |
|  | IRAK3 |  |
|  | LGALSL |  |
|  | ZNF486 |  |
|  | EGF |  |
|  | NDUFB3 |  |
|  | SORT1 |  |
|  | MPP1 |  |
|  | SERPINB1 |  |
|  | CTTN |  |
|  | MFAP3L |  |
|  | ATP8B4 |  |
|  | PKHD1L1 |  |
|  | GPR141 |  |
|  | CDK2AP1 |  |
|  | NUDT5 |  |
|  | XK |  |
|  | VEPH1 |  |
|  | MPZL1 |  |
|  | HSPA1A |  |
|  | CD226 |  |
|  | NEXN |  |
|  | CARD6 |  |
|  | RAB27B |  |
|  | TMEM40 |  |
|  | SMOX |  |
|  | TNFSF4 |  |
|  | ELOVL7 |  |
|  | CLEC4D |  |
|  | HAL |  |
|  | PPBP |  |
|  | SLC8A1 |  |
|  | B4GALT5 |  |
|  | FLT3 |  |
|  | MTHFS |  |
|  | MBOAT2 |  |
|  | C3orf62 |  |
|  | PLA2G12A |  |
|  | ENDOD1 |  |
|  | RRAGD |  |
|  | BECN1 |  |
|  | SLC22A4 |  |
|  | ARHGEF12 |  |
|  | STOM |  |
|  | C1orf85 |  |
|  | NFIB |  |
|  | LIN7A |  |
|  | GP6 |  |
|  | JAM3 |  |
|  | PSMB6 |  |
|  | CCDC144CP |  |
|  | NQO2 |  |
|  | CCDC159 |  |
|  | NFE4 |  |
|  | ZNF429 |  |
|  | YWHAH |  |
|  | ANO6 |  |
|  | NFIL3 |  |
|  | CCNJL |  |
|  | TLR4 |  |
|  | ITGB5 |  |
|  | MAPK14 |  |
|  | HTATIP2 |  |
|  | RHOBTB1 |  |
|  | ZFAND1 |  |
|  | RNU6-475P |  |
|  | AQP10 |  |
|  | ARHGAP19 |  |
|  | ASAP2 |  |
|  | LRRC6 |  |
|  | GPD2 |  |
|  | TMEM185A |  |
|  | TSPAN33 |  |
|  | SRPK1 |  |
|  | RSBN1L |  |
|  | NDUFAF1 |  |
|  | MSRB2 |  |
|  | FAXDC2 |  |
|  | DDX11L2 |  |
|  | LRPAP1 |  |
|  | TST |  |
|  | TDP2 |  |
|  | MTFMT |  |
|  | IGF2BP3 |  |
|  | SKA2 |  |
|  | CDKL5 |  |
|  | CPNE2 |  |
|  | ZNF438 |  |
|  | LY6G6F |  |
|  | USP32P3 |  |
|  | MCTP1 |  |
|  | FGD4 |  |
|  | GLB1 |  |
|  | RP2 |  |
|  | TMEM91 |  |
|  | ZNF487 |  |
|  | NLRP12 |  |
|  | MSL3P1 |  |
|  | ANO10 |  |
|  | SULT1A2 |  |
|  | BIN2 |  |
|  | PACSIN2 |  |
|  | HAUS4 |  |
|  | KRCC1 |  |
|  | MLLT4 |  |
|  | FCAR |  |
|  | TMEM55A |  |
|  | ARPC1B |  |
|  | RNASEL |  |
|  | ZNF814 |  |
|  | CDC14B |  |
|  | RSU1 |  |
|  | RALGAPA2 |  |
|  | PPP2R3C |  |
|  | TMOD3 |  |
|  | SNX19P1 |  |
|  | ASF1B |  |
|  | CLEC6A |  |
|  | DEGS1 |  |
|  | PRDX6 |  |
|  | GDE1 |  |
|  | PARVB |  |
|  | RILPL2 |  |
|  | HSPA1B |  |
|  | TM6SF1 |  |
|  | LHFPL2 |  |
|  | RAB31 |  |
|  | APMAP |  |
|  | TLR6 |  |
|  | SUPT20HL2 |  |
|  | ANKRD28 |  |
|  | SFT2D1 |  |
|  | 2-Mar |  |
|  | ELOF1 |  |
|  | DPM1 |  |
|  | DNAJC8 |  |
|  | TFPI |  |
|  | CD59 |  |
|  | IKBKG |  |
|  | GRAP2 |  |
|  | MLKL |  |
|  | TRIM21 |  |
|  | CNIH4 |  |
|  | TTC7B |  |
|  | SLC4A1AP |  |
|  | SQRDL |  |
|  | OASL |  |
|  | PIK3CB |  |
|  | FSTL1 |  |
|  | PARP9 |  |
|  | TRAPPC6B |  |
|  | RNF146 |  |
|  | HK2 |  |
|  | KIAA0391 |  |
|  | APP |  |
|  | CLCN3 |  |
|  | SDPR |  |
|  | RPH3A |  |
|  | KCNE1 |  |
|  | ABLIM3 |  |
|  | PTGES3P1 |  |
|  | LIMS1 |  |
|  | CSGALNACT2 |  |
|  | GBA |  |
|  | GPSM2 |  |
|  | MGLL |  |
|  | FAH |  |
|  | ST20 |  |
|  | NRD1 |  |
|  | FRMD4B |  |
|  | E2F3 |  |
|  | MEIS1 |  |
|  | MTHFD2P7 |  |
|  | NRGN |  |
|  | BCL2A1 |  |
|  | EIF2AK2 |  |
|  | SLC22A15 |  |
|  | HIST2H2AA4 |  |
|  | CLEC1B |  |
|  | BEST1 |  |
|  | RN7SKP292 |  |
|  | NCBP2L |  |
|  | ZNF75D |  |
|  | LCLAT1 |  |
|  | SRR |  |
|  | PDE3A |  |
|  | ARHGAP18 |  |
|  | NBR1 |  |
|  | UIMC1 |  |
|  | LMAN2 |  |
|  | DNAJC3 |  |
|  | SLC31A2 |  |
|  | MSL3 |  |
|  | SULT1A1 |  |
|  | FAM177A1 |  |
|  | HRH2 |  |
|  | ACSL4 |  |
|  | TRPC2 |  |
|  | C15orf38-AP3S2 |  |
|  | RNU6-938P |  |
|  | DAB2 |  |
|  | CYP4F3 |  |
|  | PSMA6 |  |
|  | WLS |  |
|  | CHURC1-FNTB |  |
|  | NCKAP1 |  |
|  | FBXO38 |  |
|  | MX2 |  |
|  | SLC16A6 |  |
|  | TGFB2 |  |
|  | RNU6-1235P |  |
|  | CLEC1A |  |
|  | LYRM1 |  |
|  | PCSK6 |  |
|  | OSBPL11 |  |
|  | SH3GLB1 |  |
|  | NETO2 |  |
|  | MYLIP |  |
|  | PCMT1 |  |
|  | TAX1BP3 |  |
|  | SLC44A1 |  |
|  | PRKG1 |  |
|  | GPR97 |  |
|  | PISD |  |
|  | EXOC6 |  |
|  | MXD1 |  |
|  | PDK3 |  |
|  | MTURN |  |
|  | SIRPD |  |
|  | CCDC125 |  |
|  | P2RY12 |  |
|  | PCTP |  |
|  | NME8 |  |
|  | EMB |  |
|  | NXT2 |  |
|  | CFLAR |  |
|  | POLE2 |  |
|  | ENTPD1 |  |
|  | KPNA4 |  |
|  | RAB33B |  |
|  | EFHC2 |  |
|  | PPIP5K2 |  |
|  | HSDL2 |  |
|  | C6orf211 |  |
|  | PDLIM1 |  |
|  | DHPS |  |
|  | NPL |  |
|  | PPP4R1 |  |
|  | MAB21L3 |  |
|  | LDHA |  |
|  | WIPI1 |  |
|  | PPP2R2D |  |
|  | CTBS |  |
|  | RNF144B |  |
|  | LAIR1 |  |
|  | FAM105A |  |
|  | TNFRSF9 |  |
|  | GSKIP |  |
|  | RMI1 |  |
|  | FOXRED1 |  |
|  | MARK2 |  |
|  | GIPC3 |  |
|  | SCFD2 |  |
|  | ILK |  |
|  | IPO8 |  |
|  | RBPMS2 |  |
|  | CLEC7A |  |
|  | GALNT3 |  |
|  | MPZL3 |  |
|  | N4BP1 |  |
|  | UGP2 |  |
|  | PJA2 |  |
|  | CA2 |  |
|  | HSD17B12 |  |
|  | NUDT16P1 |  |
|  | CEBPD |  |
|  | PUS10 |  |
|  | DAPK2 |  |
|  | ODC1 |  |
|  | TRIM27 |  |
|  | TREML2 |  |
|  | CPQ |  |
|  | PSTPIP2 |  |
|  | SLFN12 |  |
|  | GBE1 |  |
|  | MERTK |  |
|  | FAM65B |  |
|  | DSCR3 |  |
|  | SLC36A1 |  |
|  | FAM151B |  |
|  | LEPR |  |
|  | NUP50 |  |
|  | GTF2H2C_2 |  |
|  | PLB1 |  |
|  | ACTR10 |  |
|  | TRAPPC1 |  |
|  | SNN |  |
|  | RAB32 |  |
|  | HSD17B11 |  |
|  | LAMTOR1 |  |
|  | SLA2 |  |
|  | LIMS3L |  |
|  | SOS2 |  |
|  | HN1 |  |
|  | STXBP5 |  |
|  | SNX27 |  |
|  | ZMAT5 |  |
|  | GTF3C6 |  |
|  | CABP5 |  |
|  | HGD |  |
|  | TIMP2 |  |
|  | TCN2 |  |
|  | HPCAL1 |  |
|  | GSK3B |  |
|  | ETFA |  |
|  | PDCL |  |
|  | MYLK |  |
|  | CD9 |  |
|  | ZNF33A |  |
|  | ISY1 |  |
|  | AGTRAP |  |
|  | TSNAX |  |
|  | RAB28 |  |
|  | HIST1H2AG |  |
|  | TBC1D2 |  |
|  | IREB2 |  |
|  | TJP2 |  |
|  | LHFP |  |
|  | LAT2 |  |
|  | AKTIP |  |
|  | PTTG1IP |  |
|  | PCYOX1 |  |
|  | BTBD10 |  |
|  | CORO1C |  |
|  | SEMA4A |  |
|  | AVPR1A |  |
|  | TRIP4 |  |
|  | TANGO2 |  |
|  | SSH1 |  |
|  | MYL12A |  |
|  | TSC22D3 |  |
|  | PCYT1B |  |
|  | KIF3C |  |
|  | HARS |  |
|  | ADAM9 |  |
|  | ASB7 |  |
|  | SERPINA1 |  |
|  | SUOX |  |
|  | CCPG1 |  |
|  | FCER1G |  |
|  | RNF24 |  |
|  | RGS6 |  |
|  | HIGD1A |  |
|  | PGM1 |  |
|  | TIMMDC1 |  |
|  | DUSP18 |  |
|  | KDM7A |  |
|  | PPM1D |  |
|  | BCL6 |  |
|  | RMND5A |  |
|  | ORAI2 |  |
|  | YIPF3 |  |
|  | CHMP3 |  |
|  | ARMC3 |  |
|  | SNAP23 |  |
|  | SPTLC2 |  |
|  | SCPEP1 |  |
|  | MED8 |  |
|  | IL4R |  |
|  | RIPK3 |  |
|  | ABCC3 |  |
|  | CCDC144NL |  |
|  | C7orf49 |  |
|  | STON2 |  |
|  | SLC35A5 |  |
|  | AVIL |  |
|  | C1orf123 |  |
|  | RALBP1 |  |
|  | DYNLRB1 |  |
|  | MFSD1 |  |
|  | SIAE |  |
|  | GBAP1 |  |
|  | RNU6-189P |  |
|  | VAV1 |  |
|  | VNN3 |  |
|  | MANBAL |  |
|  | BAZ1A |  |
|  | GSN |  |
|  | OAZ1 |  |
|  | CTSA |  |
|  | NUCB1 |  |
|  | WDR47 |  |
|  | CSF2RA |  |
|  | CERS5 |  |
|  | NT5DC3 |  |
|  | KIAA0513 |  |
|  | CCDC30 |  |
|  | SNX13 |  |
|  | B4GALT6 |  |
|  | SSX2IP |  |
|  | GPX4 |  |
|  | RFC2 |  |
|  | NAGK |  |
|  | TRAPPC5 |  |
|  | PINK1 |  |
|  | ZNF106 |  |
|  | GALM |  |
|  | VIPAS39 |  |
|  | C14orf119 |  |
|  | ZNF552 |  |
|  | SSFA2 |  |
|  | ATE1 |  |
|  | PLD1 |  |
|  | MYL8P |  |
|  | APOBEC3A |  |
|  | ERGIC1 |  |
|  | NPEPPS |  |
|  | TRAFD1 |  |
|  | NSFL1C |  |
|  | HEBP1 |  |
|  | ARHGAP21 |  |
|  | TKT |  |
|  | CLTCL1 |  |
|  | TIPIN |  |
|  | ZNF284 |  |
|  | NDUFS2 |  |
|  | UBR7 |  |
|  | TMCO6 |  |
|  | PTPN18 |  |
|  | GCLM |  |
|  | HEATR5A |  |
|  | JPX |  |
|  | RDH10 |  |
|  | SLC6A4 |  |
|  | HMGN2P6 |  |
|  | PARP16 |  |
|  | IRF2BPL |  |
|  | MILR1 |  |
|  | POLD3 |  |
|  | PANX1 |  |
|  | PXK |  |
|  | TTI2 |  |
|  | RNA5SP198 |  |
|  | TERF1P2 |  |
|  | STK3 |  |
|  | BTNL8 |  |
|  | DSEL |  |
|  | DNAJC13 |  |
|  | FAM45A |  |
|  | HK3 |  |
|  | XKR3 |  |
|  | MSRA |  |
|  | LAPTM4B |  |
|  | ANTXR2 |  |
|  | SMIM8 |  |
|  | SNORA37 |  |
|  | LOC101060747 |  |
|  | ACVR1 |  |
|  | ZMAT3 |  |
|  | ALDOC |  |
|  | COG4 |  |
|  | NADK |  |
|  | DEDD |  |
|  | C1GALT1C1 |  |
|  | MTM1 |  |
|  | PDK1 |  |
|  | RNU6-643P |  |
|  | EDEM2 |  |
|  | SCYL2 |  |
|  | GLA |  |
|  | TMEM150B |  |
|  | MAPK1 |  |
|  | ERVH-6 |  |
|  | H2AFV |  |
|  | OSBPL5 |  |
|  | ABCG1 |  |
|  | TBL1X |  |
|  | RPS6KA1 |  |
|  | LYPLA1 |  |
|  | PXYLP1 |  |
|  | TNFAIP8L2-SCNM1 |  |
|  | SDCBP |  |
|  | RAC1 |  |
|  | STARD10 |  |
|  | COPE |  |
|  | CDK19 |  |
|  | MMD |  |
|  | ARF4 |  |
|  | WSB1 |  |
|  | RUFY1 |  |
|  | CINP |  |
|  | ITPK1 |  |
|  | KIFAP3 |  |
|  | IL1RN |  |
|  | ZNF749 |  |
|  | IL10RB |  |
|  | M1AP |  |
|  | PSMA1 |  |
|  | CHURC1 |  |
|  | TEX2 |  |
|  | C2orf88 |  |
|  | DNAJC14 |  |
|  | CMTM6 |  |
|  | ITGAM |  |
|  | C9orf78 |  |
|  | INPP5A |  |
|  | ALDH2 |  |
|  | SAP30L |  |
|  | KAT6A |  |
|  | KDM5A |  |
|  | CASC3 |  |
|  | KIT |  |
|  | RHOA |  |
|  | ARHGAP15 |  |
|  | MB21D1 |  |
|  | CNTLN |  |
|  | QSOX1 |  |
|  | RILPL1 |  |
|  | STAT3 |  |
|  | DPY19L3 |  |
|  | UBE2Q1 |  |
|  | TMEM167A |  |
|  | RNU6-759P |  |
|  | TBC1D30 |  |
|  | KIAA0319L |  |
|  | TM7SF3 |  |
|  | SLC35B3 |  |
|  | SPP1 |  |
|  | FERMT3 |  |
|  | SEL1L |  |
|  | FLOT1 |  |
|  | PTPRJ |  |
|  | PAK2 |  |
|  | CERS2 |  |
|  | RNA5SP307 |  |
|  | CLIP1 |  |
|  | ATP6AP1 |  |
|  | DOK6 |  |
|  | ENY2 |  |
|  | PPP4R2 |  |
|  | TOR1A |  |
|  | H3F3C |  |
|  | ATP6V1D |  |
|  | MAP2K6 |  |
|  | LBR |  |
|  | TMUB2 |  |
|  | HMBS |  |
|  | MEFV |  |
|  | MLX |  |
|  | LPGAT1 |  |
|  | RBM23 |  |
|  | FEZ2 |  |
|  | SAV1 |  |
|  | DIRC2 |  |
|  | SEC62 |  |
|  | ARF3 |  |
|  | SLC35E3 |  |
|  | GLTSCR1L |  |
|  | ESAM |  |
|  | HADHA |  |
|  | RAB11A |  |
|  | FAM204A |  |
|  | GDI2 |  |
|  | SIRT5 |  |
|  | GABARAPL2 |  |
|  | NCF2 |  |
|  | NFYC |  |
|  | SMIM7 |  |
|  | ADSS |  |
|  | SLC35D2 |  |
|  | KIAA0430 |  |
|  | MSANTD3 |  |
|  | TMEM43 |  |
|  | CRIPT |  |
|  | STRIP2 |  |
|  | ASH2L |  |
|  | STX11 |  |
|  | FANCI |  |
|  | LZIC |  |
|  | TRIM22 |  |
|  | SRBD1 |  |
|  | ZNF148 |  |
|  | ATP6V1A |  |
|  | FAM192A |  |
|  | PSTPIP1 |  |
|  | SYTL3 |  |
|  | CCT5 |  |
|  | AIG1 |  |
|  | ST7 |  |
|  | KCND3 |  |
|  | FBXL5 |  |
|  | TAGAP |  |
|  | CASS4 |  |
|  | NCAPD2 |  |
|  | TADA2A |  |
|  | KLHL12 |  |
|  | CSNK1A1 |  |
|  | CRLF3 |  |
|  | FGR |  |
|  | TMEM71 |  |
|  | SCLT1 |  |
|  | OSBPL1A |  |
|  | GPANK1 |  |
|  | CEP97 |  |
|  | SPATA6 |  |
|  | MPP7 |  |
|  | TMEM120A |  |
|  | CNN2 |  |
|  | C11orf54 |  |
|  | FLI1 |  |
|  | LAMTOR5P1 |  |
|  | GINM1 |  |
|  | BICD2 |  |
|  | PGK1 |  |
|  | TRAPPC8 |  |
|  | EPS15 |  |
|  | ZNF346 |  |
|  | HNRNPLL |  |
|  | CAV2 |  |
|  | KAT2B |  |
|  | GTPBP2 |  |
|  | CCM2 |  |
|  | RACGAP1 |  |
|  | TMX4 |  |
|  | ALOX5 |  |
|  | HIF1AN |  |
|  | MAEA |  |
|  | TYROBP |  |
|  | METTL9 |  |
|  | MAML3 |  |
|  | GMNN |  |
|  | PLGLB1 |  |
|  | RUNDC1 |  |
|  | SLC14A1 |  |
|  | ATF6 |  |
|  | MICAL2 |  |
|  | HEXIM1 |  |
|  | TBC1D14 |  |
|  | KDELR2 |  |
|  | GSR |  |
|  | TMEM106C |  |
|  | GRN |  |
|  | RAB24 |  |
|  | ATG13 |  |
|  | ELP3 |  |
|  | CCND3 |  |
|  | RNF130 |  |
|  | CADM1 |  |
|  | CTSB |  |
|  | SH3BGRL |  |
|  | SCP2 |  |
|  | PTEN |  |
|  | FBXW2 |  |
|  | TAF7 |  |
|  | EIF4G3 |  |
|  | NFIA |  |
|  | CARD8 |  |
|  | TCEANC2 |  |
|  | SH3BP5 |  |
|  | TCAIM |  |
|  | LOC100996517 |  |
|  | COQ7 |  |
|  | NPTN |  |
|  | LCOR |  |
|  | OSTF1 |  |
|  | PDXK |  |
|  | LRRC8D |  |
|  | FAM188A |  |
|  | VPS39 |  |
|  | OXSR1 |  |
|  | PPP2R2A |  |
|  | GLB1L |  |
|  | SDHB |  |
|  | GOLM1 |  |
|  | STK24 |  |
|  | STX6 |  |
|  | CHPT1 |  |
|  | ZNF281 |  |
|  | EBLN2 |  |
|  | COL4A3BP |  |
|  | AGPS |  |
|  | STAG2 |  |
|  | STRN |  |
|  | ARFGEF1 |  |
|  | SMIM11 |  |
|  | PHKA2 |  |
|  | P4HB |  |
|  | NATD1 |  |
|  | CKAP2 |  |
|  | TMEM185B |  |
|  | AMPD3 |  |
|  | SDHAF2 |  |
|  | PIGC |  |
|  | THAP6 |  |
|  | DPEP2 |  |
|  | WWC2-AS2 |  |
|  | FAM13A |  |
|  | LUZP6 |  |
|  | TWF2 |  |
|  | CAST |  |
|  | MAP2K4 |  |
|  | USP10 |  |
|  | CHRNA2 |  |
|  | ZYG11B |  |
|  | EMC3 |  |
|  | LAMTOR5 |  |
|  | NDUFB9 |  |
|  | RNU6-788P |  |
|  | PECR |  |
|  | FCN1 |  |
|  | GMPR2 |  |
|  | SPTLC1 |  |
|  | PLOD1 |  |
|  | PRKCZ |  |
|  | SEPT4 |  |
|  | ME2 |  |
|  | CD55 |  |
|  | PSMD1 |  |
|  | ARSG |  |
|  | NXPE3 |  |
|  | TRIP11 |  |
|  | CRADD |  |
|  | AMD1 |  |
|  | SP1 |  |
|  | TACC3 |  |
|  | INTS8 |  |
|  | ERMAP |  |
|  | THRAP3 |  |
|  | SNX10 |  |
|  | KIDINS220 |  |
|  | AIFM1 |  |
|  | RAB8B |  |
|  | VCPIP1 |  |
|  | CDC42P6 |  |
|  | APPBP2 |  |
|  | ACAP2 |  |
|  | FBXO48 |  |
|  | NKIRAS2 |  |
|  | TBL1XR1 |  |
|  | CWC25 |  |
|  | POLI |  |
|  | SERINC3 |  |
|  | MCFD2 |  |
|  | PLAGL1 |  |
|  | HK1 |  |
|  | TTLL4 |  |
|  | FMO5 |  |
|  | ANAPC7 |  |
|  | LOC646358 |  |
|  | CALCOCO1 |  |
|  | FAR1 |  |
|  | PPA2 |  |
|  | TRIM9 |  |
|  | RNU6-708P |  |
|  | FOCAD |  |
|  | ARCN1 |  |
|  | DNASE1L1 |  |
|  | TLK1 |  |
|  | ST3GAL3 |  |
|  | ZP3 |  |
|  | NAA50 |  |
|  | ANXA11 |  |
|  | GXYLT1 |  |
|  | NEU1 |  |
|  | TDRP |  |
|  | IQGAP1 |  |
|  | H2AFY |  |
|  | ARMC8 |  |
|  | CLASP1 |  |
|  | RPIA |  |
|  | ATL3 |  |
|  | DENND2C |  |
|  | TMTC2 |  |
|  | VAV3 |  |
|  | ELMOD3 |  |
|  | ARNT |  |
|  | FBXO18 |  |
|  | CHSY1 |  |
|  | STAM |  |
|  | HADHB |  |
|  | CEP41 |  |
|  | RASA1 |  |
|  | PRPF38A |  |
|  | TOLLIP |  |
|  | AGBL5 |  |
|  | DPYD |  |
|  | AP3B1 |  |
|  | SPPL3 |  |
|  | FBP2 |  |
|  | CDC27 |  |
|  | PDGFRB |  |
|  | SRP54 |  |
|  | RSPRY1 |  |
|  | SPCS3 |  |
|  | STAMBP |  |
|  | RNU6-879P |  |
|  | SLC36A4 |  |
|  | PDS5B |  |
|  | POLR2KP2 |  |
|  | ZNF469 | down |
|  | LOC100128653 |  |
|  | BSX |  |
|  | HNRNPA3P1 |  |
|  | SEMA3G |  |
|  | RN7SKP122 |  |
|  | TEX19 |  |
|  | CDK15 |  |
|  | SPINK4 |  |
|  | OR5A1 |  |
|  | TBX18 |  |
|  | KCNJ6 |  |
|  | ATP8B5P |  |
|  | KCNH8 |  |
|  | ST6GALNAC4 |  |
|  | KIAA0125 |  |
|  | CDH22 |  |
|  | GJB1 |  |
|  | CALML6 |  |
|  | LEPREL1 |  |
|  | ADIG |  |
|  | C10orf91 |  |
|  | KCNA6 |  |
|  | LCE3B |  |
|  | FGFR2 |  |
|  | OLIG2 |  |
|  | ASB12 |  |
|  | GATA4 |  |
|  | CHPF |  |
|  | CD276 |  |
|  | AQP5 |  |
|  | CHST10 |  |
|  | SCD5 |  |
|  | WDR93 |  |
|  | CYP17A1 |  |
|  | MDK |  |
|  | NFKBIE |  |
|  | ANKS6 |  |
|  | STMN4 |  |
|  | PAX9 |  |
|  | MIR455 |  |
|  | HRCT1 |  |
|  | TDRD12 |  |
|  | NCCRP1 |  |
|  | FLJ11292 |  |
|  | REXO1 |  |
|  | RN7SL846P |  |
|  | CEACAM20 |  |
|  | C20orf181 |  |
|  | CCDC121 |  |
|  | CAPN7 |  |
|  | NIPAL4 |  |
|  | DIXDC1 |  |
|  | FAM162B |  |
|  | RN7SL570P |  |
|  | EFCC1 |  |
|  | RN7SL468P |  |
|  | PLEKHA7 |  |
|  | TNNT2 |  |
|  | GPR173 |  |
|  | DAO |  |
|  | PRDM12 |  |
|  | CNGA2 |  |
|  | ABLIM2 |  |
|  | RN7SL792P |  |
|  | TAP1 |  |
|  | RN7SL756P |  |
|  | FOXC2 |  |
|  | KIF5A |  |
|  | LOC389199 |  |
|  | ACTL7B |  |
|  | C7orf72 |  |
|  | GNA14 |  |
|  | ACOT11 |  |
|  | FAM71D |  |
|  | ALOX12B |  |
|  | RNA5SP469 |  |
|  | KSR2 |  |
|  | TRHDE-AS1 |  |
|  | LRRN4 |  |
|  | CD70 |  |
|  | NACAD |  |
|  | PNLIPRP1 |  |
|  | LAMP3 |  |
|  | C22orf15 |  |
|  | NLRP10 |  |
|  | C4orf6 |  |
|  | CLLU1OS |  |
|  | RESP18 |  |
|  | TRIM29 |  |
|  | TM4SF5 |  |
|  | C16orf89 |  |
|  | MIR141 |  |
|  | NAPSA |  |
|  | RN7SL150P |  |
|  | PRSS33 |  |
|  | SCG3 |  |
|  | MORN5 |  |
|  | ISLR |  |
|  | LOXL2 |  |
|  | IGSF9 |  |
|  | MPPED1 |  |
|  | SCN3B |  |
|  | MUC4 |  |
|  | RPL39L |  |
|  | CELSR1 |  |
|  | EFNA1 |  |
|  | CREB3L3 |  |
|  | CDH15 |  |
|  | SYT9 |  |
|  | NT5C1A |  |
|  | HIC2 |  |
|  | RNU6-321P |  |
|  | RNF151 |  |
|  | GALR2 |  |
|  | SYN2 |  |
|  | RNU6-1075P |  |
|  | RN7SL578P |  |
|  | SLC1A6 |  |
|  | PAX8 |  |
|  | NR1I2 |  |
|  | ZNF667 |  |
|  | ARL5C |  |
|  | KCNIP2 |  |
|  | TH |  |
|  | ADCYAP1R1 |  |
|  | HHIPL1 |  |
|  | DNER |  |
|  | IL17RD |  |
|  | CYLC2 |  |
|  | SPATA9 |  |
|  | GALP |  |
|  | DENND2A |  |
|  | ARHGEF26 |  |
|  | RN7SL392P |  |
|  | WT1-AS |  |
|  | SOX3 |  |
|  | GOLGA2P9 |  |
|  | OR5AQ1P |  |
|  | HMGCS1 |  |
|  | SLC45A3 |  |
|  | HSD17B2 |  |
|  | CCDC81 |  |
|  | GPX5 |  |
|  | SIX4 |  |
|  | NKX2-1-AS1 |  |
|  | OR7G3 |  |
|  | RNU6ATAC38P |  |
|  | DHDH |  |
|  | GPAM |  |
|  | RPL31P7 |  |
|  | ZCCHC5 |  |
|  | DLGAP3 |  |
|  | IL20RA |  |
|  | ZSCAN4 |  |
|  | TEKT2 |  |
|  | PRSS54 |  |
|  | ABHD15 |  |
|  | ACSBG2 |  |
|  | BRINP1 |  |
|  | LTK |  |
|  | RNU6-1044P |  |
|  | RNU6-284P |  |
|  | RNA5SP78 |  |
|  | MAZ |  |
|  | IGFBP4 |  |
|  | RN7SL664P |  |
|  | CTGF |  |
|  | NKX6-3 |  |
|  | AXL |  |
|  | DEGS2 |  |
|  | RND2 |  |
|  | KLHDC8A |  |
|  | KRT5 |  |
|  | SLC45A1 |  |
|  | TVP23A |  |
|  | FBXO46 |  |
|  | KLK10 |  |
|  | DNM1P46 |  |
|  | NACA3P |  |
|  | CLDN23 |  |
|  | RNU4-8P |  |
|  | HEY1 |  |
|  | KIAA1024L |  |
|  | RNA5SP209 |  |
|  | RN7SL42P |  |
|  | MIR323B |  |
|  | CLCNKB |  |
|  | TCTN2 |  |
|  | TTC23L |  |
|  | RNU7-174P |  |
|  | TBC1D3P5 |  |
|  | GPR3 |  |
|  | SPHKAP |  |
|  | FSHR |  |
|  | EPHX4 |  |
|  | HOXB5 |  |
|  | OR8S1 |  |
|  | RNU6-131P |  |
|  | MIR196A2 |  |
|  | DKFZP434K028 |  |
|  | RBPMS |  |
|  | SLC22A18AS |  |
|  | FABP3 |  |
|  | GSTA1 |  |
|  | CD207 |  |
|  | ZNF713 |  |
|  | RNU1-132P |  |
|  | TMEM171 |  |
|  | RGS20 |  |
|  | SDR9C7 |  |
|  | TCEB1P3 |  |
|  | RPL10L |  |
|  | SMIM23 |  |
|  | ZBTB42 |  |
|  | RN7SKP214 |  |
|  | METTL20 |  |
|  | TTLL9 |  |
|  | KIAA0895L |  |
|  | CPO |  |
|  | RN7SL434P |  |
|  | ASIP |  |
|  | ADO |  |
|  | MIR34C |  |
|  | ZSCAN10 |  |
|  | HSBP1P2 |  |
|  | ENDOG |  |
|  | PLEKHG1 |  |
|  | RNU6-716P |  |
|  | TNFRSF13B |  |
|  | EBF3 |  |
|  | GPR39 |  |
|  | ADRB3 |  |
|  | RNA5SP53 |  |
|  | HOXB4 |  |
|  | SKOR2 |  |
|  | FAM83C |  |
|  | SNTG1 |  |
|  | NLGN2 |  |
|  | RNU6-556P |  |
|  | LINC00951 |  |
|  | CACNG6 |  |
|  | IRX6 |  |
|  | PCAT18 |  |
|  | C14orf23 |  |
|  | SYNC |  |
|  | LOC100506571 |  |
|  | STX19 |  |
|  | PINK1-AS |  |
|  | RNU6-1324P |  |
|  | RNA5SP258 |  |
|  | ENHO |  |
|  | GMCL1P1 |  |
|  | RN7SL418P |  |
|  | FLJ46365 |  |
|  | GPR110 |  |
|  | NEU2 |  |
|  | MRPL36 |  |
|  | RN7SL208P |  |
|  | RN7SKP113 |  |
|  | OR51D1 |  |
|  | CLDN22 |  |
|  | GUCY2D |  |
|  | EYS |  |
|  | ADAMTS12 |  |
|  | RN7SL504P |  |
|  | MXRA8 |  |
|  | KRT39 |  |
|  | GCRG224 |  |
|  | C4orf19 |  |
|  | SLC10A6 |  |
|  | RNU6-501P |  |
|  | MS4A10 |  |
|  | UBD |  |
|  | IGFBP1 |  |
|  | MUC3A |  |
|  | MKRN3 |  |
|  | CNBD2 |  |
|  | LOC100130264 |  |
|  | MRO |  |
|  | RN7SL375P |  |
|  | RNU6-500P |  |
|  | SLITRK1 |  |
|  | SCARA5 |  |
|  | TEX13B |  |
|  | SLC22A6 |  |
|  | OLFML1 |  |
|  | RNU6-726P |  |
|  | SRY |  |
|  | SDR42E1 |  |
|  | RNA5SP458 |  |
|  | SLC51B |  |
|  | TCHH |  |
|  | LPHN3 |  |
|  | CCL13 |  |
|  | RPL29P30 |  |
|  | SPAG11A |  |
|  | SPERT |  |
|  | CACNA1F |  |
|  | ARSI |  |
|  | FOXL1 |  |
|  | CEACAM7 |  |
|  | SLC19A3 |  |
|  | RNU6-999P |  |
|  | CASP14 |  |
|  | LRRC66 |  |
|  | ATP1B2 |  |
|  | GRK1 |  |
|  | OLIG3 |  |
|  | SELE |  |
|  | OPRK1 |  |
|  | RN7SL266P |  |
|  | CCDC168 |  |
|  | FOXA2 |  |
|  | FBXO24 |  |
|  | ARGFX |  |
|  | LRRC36 |  |
|  | TMEM136 |  |
|  | LOC101060524 |  |
|  | CD19 |  |
|  | BLK |  |
|  | DBX1 |  |
|  | PIGH |  |
|  | PPIF |  |
|  | PCDHB1 |  |
|  | C16orf87 |  |
|  | RNU6-616P |  |
|  | RN7SL282P |  |
|  | CAP2 |  |
|  | SERPINA5 |  |
|  | XKR4 |  |
|  | PLG |  |
|  | MUC1 |  |
|  | POU5F2 |  |
|  | SPATA31C1 |  |
|  | RN7SKP8 |  |
|  | RNA5SP238 |  |
|  | SCIN |  |
|  | HSD17B1P1 |  |
|  | LOC101928751 |  |
|  | CHRNA9 |  |
|  | TCHHL1 |  |
|  | TMIGD1 |  |
|  | PLEKHH1 |  |
|  | CYP4Z2P |  |
|  | RN7SL515P |  |
|  | RN7SL766P |  |
|  | ERVFC1-1 |  |
|  | FAM169B |  |
|  | RN7SL168P |  |
|  | KIAA1217 |  |
|  | PPIAP11 |  |
|  | TRIM17 |  |
|  | RNU6-1184P |  |
|  | HIST1H2AA |  |
|  | RN7SL700P |  |
|  | RN7SL408P |  |
|  | RNA5SP54 |  |
|  | JUND |  |
|  | NPHS2 |  |
|  | CCL3 |  |
|  | MIR130B |  |
|  | IL36A |  |
|  | RNU6-636P |  |
|  | C8orf31 |  |
|  | DUSP9 |  |
|  | TNFAIP8L3 |  |
|  | RSPH10B2 |  |
|  | FDCSP |  |
|  | PCDH20 |  |
|  | LOC100996619 |  |
|  | NFKB2 |  |
|  | FAM181A |  |
|  | CORO2B |  |
|  | OR2Z1 |  |
|  | RNA5SP31 |  |
|  | PDLIM3 |  |
|  | CEND1 |  |
|  | MYF6 |  |
|  | ERICH6 |  |
|  | RN7SL645P |  |
|  | LINC01356 |  |
|  | ZC2HC1C |  |
|  | DPY19L2P4 |  |
|  | TDO2 |  |
|  | REL |  |
|  | RNU4-27P |  |
|  | MIR23B |  |
|  | RNA5SP519 |  |
|  | RN7SL38P |  |
|  | AKNAD1 |  |
|  | RN7SL842P |  |
|  | RNA5SP491 |  |
|  | C1orf168 |  |
|  | MIR138-1 |  |
|  | RNA5SP64 |  |
|  | CTU1 |  |
|  | OR2V1 |  |
|  | C14orf177 |  |
|  | PTGDR2 |  |
|  | RNU6-877P |  |
|  | RN7SL541P |  |
|  | RN7SL757P |  |
|  | UCA1 |  |
|  | RNU4ATAC5P |  |
|  | CLCA3P |  |
|  | GLI1 |  |
|  | PLEKHG7 |  |
|  | RN7SKP186 |  |
|  | ZNF503-AS2 |  |
|  | RNU1-84P |  |
|  | RN7SKP53 |  |
|  | TRIM71 |  |
|  | ZNF300P1 |  |
|  | GDF2 |  |
|  | TMEM14E |  |
|  | RNA5SP118 |  |
|  | OR4D1 |  |
|  | EFCAB3 |  |
|  | ENAH |  |
|  | PCDHB11 |  |
|  | RN7SKP6 |  |
|  | RN7SL7P |  |
|  | ACTN2 |  |
|  | GSTA2 |  |
|  | C3orf43 |  |
|  | WDR63 |  |
|  | MIR34A |  |
|  | RNU6-660P |  |
|  | JPH1 |  |
|  | RNU6-363P |  |
|  | RN7SL864P |  |
|  | LOC613206 |  |
|  | RNU6-541P |  |
|  | RNA5SP110 |  |
|  | WDR88 |  |
|  | SPINT5P |  |
|  | RNA5SP319 |  |
|  | AQP7 |  |
|  | PRSS41 |  |
|  | HLA-DQA2 |  |
|  | PM20D1 |  |
|  | ENO4 |  |
|  | RNU1-31P |  |
|  | RNASE10 |  |
|  | LINC00527 |  |
|  | TRIM45 |  |
|  | CCDC148-AS1 |  |
|  | CLK1 |  |
|  | RGS8 |  |
|  | ITIH2 |  |
|  | RNU1-40P |  |
|  | C1orf87 |  |
|  | RNU6ATAC5P |  |
|  | FBXL2 |  |
|  | RNU6-1226P |  |
|  | RNA5SP504 |  |
|  | RNU4ATAC13P |  |
|  | FAM205CP |  |
|  | RTN4IP1 |  |
|  | RNA5SP404 |  |
|  | MIR320A |  |
|  | RNA5SP356 |  |
|  | RNU6-831P |  |
|  | PEG10 |  |
|  | VASH2 |  |
|  | C6orf123 |  |
|  | MEP1AP4 |  |
|  | EYA4 |  |
|  | GNG12 |  |
|  | RNA5SP408 |  |
|  | TMEM134 |  |
|  | OR2B7P |  |
|  | AIFM2 |  |
|  | DENND5B |  |
|  | SPANXA2-OT1 |  |
|  | MAGEB6P1 |  |
|  | ACTL6B |  |
|  | HSD17B7P2 |  |
|  | BTF3P11 |  |
|  | DNAJB7 |  |
|  | MIR196A1 |  |
|  | RNA5SP384 |  |
|  | RNA5SP455 |  |
|  | FAM47DP |  |
|  | RNA5SP462 |  |
|  | RN7SL685P |  |
|  | HNRNPCL1 |  |
|  | RNU6ATAC33P |  |
|  | AMBN |  |
|  | RNA5SP480 |  |
|  | SSBP3-AS1 |  |
|  | RNU4ATAC6P |  |
|  | TTTY13 |  |
|  | CLEC19A |  |
|  | RNU5F-6P |  |
|  | RN7SL516P |  |
|  | SCN2B |  |
|  | IFNL3 |  |
|  | RNU6-973P |  |
|  | LCE2B |  |
|  | CSTL1 |  |
|  | APOA2 |  |
|  | MS4A12 |  |
|  | MIR132 |  |
|  | C1QL2 |  |
|  | LINC01165 |  |
|  | RNU4ATAC2P |  |
|  | RNA5SP116 |  |
|  | PRSS55 |  |
|  | LOC645188 |  |
|  | FAM227A |  |
|  | RNA5SP204 |  |
|  | FLJ41327 |  |
|  | CACNG2 |  |
|  | WFDC10A |  |
|  | OR2A2 |  |
|  | RNA5SP360 |  |
|  | UPF3A |  |
|  | RN7SL44P |  |
|  | CTHRC1 |  |
|  | MIR218-1 |  |
|  | CDR1 |  |
|  | RN7SL465P |  |
|  | RN7SL231P |  |
|  | LOC441268 |  |
|  | KRT8 |  |
|  | TFAP2D |  |
|  | RN7SL411P |  |
|  | RNA5SP32 |  |
|  | SLC4A11 |  |
|  | RNA5SP231 |  |
|  | DKK2 |  |
|  | RNA5SP142 |  |
|  | FAM129C |  |
|  | RNA5SP375 |  |
|  | POU2AF1 |  |
|  | EYA2 |  |
|  | MIR9-1 |  |
|  | RNA5SP505 |  |
|  | RNA5SP282 |  |
|  | LCE1B |  |
|  | FCRL1 |  |
|  | NPVF |  |
|  | OR52P2P |  |
|  | RNA5SP396 |  |
|  | FGG |  |
|  | INSL5 |  |
|  | SERPINB11 |  |
|  | ANKRD20A19P |  |
|  | ZBTB10 |  |
|  | RPL22P11 |  |
|  | LOC102724314 |  |
|  | GSTM5P1 |  |
|  | TRAF4 |  |
|  | RN7SL508P |  |
|  | LOC442132 |  |
|  | RNA5SP487 |  |
|  | SPIB |  |
|  | TTTY6 |  |
|  | CXXC5 |  |
|  | WDR72 |  |
|  | RNU6-1340P |  |
|  | RN7SL326P |  |
|  | RNU6-981P |  |
|  | RNU6-336P |  |
|  | RNU4ATAC7P |  |
|  | RNY3P7 |  |
|  | OR7A17 |  |
|  | SLC35G5 |  |
|  | RNU6-218P |  |
|  | NR4A3 |  |
|  | RNA5SP35 |  |
|  | RNA5SP29 |  |
|  | CNR2 |  |
|  | DDX18P1 |  |
|  | RPSA |  |
|  | RN7SL11P |  |
|  | OR1F1 |  |
|  | OR10T1P |  |
|  | RN7SL219P |  |
|  | RN7SL771P |  |
|  | MYCNOS |  |
|  | RNU6-893P |  |
|  | FAM25C |  |
|  | RNA5SP269 |  |
|  | RNA5SP176 |  |
|  | RN7SL712P |  |
|  | SLCO1B3 |  |
|  | SNORA70E |  |
|  | LOC100133106 |  |
|  | KCNE2 |  |
|  | MIR34B |  |
|  | RNA5SP62 |  |
|  | RNF185-AS1 |  |
|  | SOX5 |  |
|  | RPS15AP25 |  |
|  | RNU6-602P |  |
|  | FOXN3P1 |  |
|  | MIR331 |  |
|  | RNU6-1029P |  |
|  | RNA5SP372 |  |
|  | RNU6-561P |  |
|  | RN7SL14P |  |
|  | RN7SL164P |  |
|  | RNA5SP305 |  |
|  | OR4L1 |  |
|  | LINC00238 |  |
|  | LOC101927628 |  |
|  | SNORD51 |  |
|  | RNU6-302P |  |
|  | RNA5SP278 |  |
|  | ARL14EP |  |
|  | OR4K3 |  |
|  | RNU6-1043P |  |
|  | HBEGF |  |
|  | OR4C5 |  |
|  | EPPIN |  |
|  | JUP |  |
|  | FLJ34521 |  |
|  | RNA5SP190 |  |
|  | RNA5SP125 |  |
|  | RNU6-425P |  |
|  | LINC00326 |  |
|  | OR10A6 |  |
|  | RNU1-46P |  |
|  | VN1R4 |  |
|  | NR4A1 |  |
|  | NLRP7 |  |
|  | RN7SL197P |  |
|  | CYP4F22 |  |
|  | RPSAP56 |  |
|  | RNA5SP76 |  |
|  | RN7SL634P |  |
|  | RNA5SP510 |  |
|  | FCRL5 |  |
|  | RNA5SP479 |  |
|  | RNU6-1188P |  |
|  | TNF |  |
|  | RNU6-528P |  |
|  | RNA5SP114 |  |
|  | PAX5 |  |
|  | RN7SKP255 |  |
|  | ZNF860 |  |
|  | RN7SL688P |  |
|  | RN7SL144P |  |
|  | RN7SL607P |  |
|  | HLA-DOA |  |
|  | RN7SL438P |  |
|  | HBG2 |  |
|  | LOC389834 |  |
|  | ADAM28 |  |
|  | RBMY2AP |  |
|  | RNU6-392P |  |
|  | RNU6-1190P |  |
|  | COBLL1 |  |
|  | SNORD35A |  |
|  | OR6B2 |  |
|  | CYP2B6 |  |
|  | RNU6-190P |  |
|  | SNORD52 |  |
|  | RNA5SP427 |  |
|  | RNU6-864P |  |
|  | RNU1-85P |  |
|  | CXCL8 |  |
|  | RNA5SP234 |  |
|  | AFF3 |  |
|  | RNU6-629P |  |
|  | RNA5SP276 |  |
|  | RNA5SP87 |  |
|  | LOC100233156 |  |
|  | LINC00268 |  |
|  | MS4A1 |  |
|  | SNORD3B-1 |  |
|  | RNA5SP366 |  |
|  | RNA5SP132 |  |
|  | ERAP2 |  |
|  | RNA5SP164 |  |
