# Supplementary material for: Immune cells transcriptome-based drug repositioning for multiple sclerosis
Source: Front Immunol. 2022 Oct 20;13:1020721. doi: 10.3389/fimmu.2022.1020721 (PMC9630342; doi:10.3389/fimmu.2022.1020721)
Supplement: Supplementary Table 2 — Detailed DEGs obtained from MS patients before and after the application of Fingolimod and IFN-β according to the type of CD19+ B cells, CD4+ T cells, pDCs and PBMC. [file Table_2.docx]

| Sample | DEG | Up/Down |
| --- | --- | --- |
| CD19^+^ B cells | RGS18 | up |
|  | HIST1H3H |  |
|  | RAB27B |  |
|  | PPBP |  |
|  | DNM3 |  |
|  | ENKUR |  |
|  | SLCO4C1 |  |
|  | MMD |  |
|  | RNA5SP348 |  |
|  | HIST1H4H |  |
|  | MIR3140 |  |
|  | RNY4 |  |
|  | OTTHUMG00000163389 |  |
|  | MOB1B |  |
|  | SRGN |  |
|  | RNA5SP243 |  |
|  | KIF2A |  |
|  | RAB11A |  |
|  | MPP6 |  |
|  | CLEC1B |  |
|  | CD200R1 |  |
|  | OTTHUMG00000161551 |  |
|  | RAP1B |  |
|  | DAPP1 |  |
|  | RSU1P1 |  |
|  | RNU4-1 |  |
|  | TMSB4XP4 |  |
|  | RNU4-2 |  |
|  | ARHGAP18 |  |
|  | CA2 |  |
|  | MIR4295 |  |
|  | ITGAX |  |
|  | IQGAP2 |  |
|  | FCRL5 |  |
|  | RNY4P1 |  |
|  | PTPN12 |  |
|  | MS4A14 |  |
|  | VTRNA1-1 |  |
|  | AGTPBP1 |  |
|  | GPR137B |  |
|  | RNU6-31P |  |
|  | RNU6-47 |  |
|  | RNA5SP199 |  |
|  | OTTHUMG00000162862 |  |
|  | MAP4K3 |  |
|  | VIM-AS1 |  |
|  | RPS15AP10 |  |
|  | RNA5SP307 |  |
|  | FGD4 |  |
|  | MIR4668 |  |
|  | RIOK3 |  |
|  | MS4A7 |  |
|  | HIST1H2BH |  |
|  | SOX5 |  |
|  | PLXNC1 |  |
|  | ZEB2 |  |
|  | MYCT1 |  |
|  | RNY4P2 |  |
|  | TOX |  |
|  | LOC400958 |  |
|  | OTTHUMG00000161550 |  |
|  | MIR644A |  |
|  | TMEM50A |  |
|  | RNA5SP450 |  |
|  | RNU6-30P |  |
|  | RNA5SP217 |  |
|  | LOC100505573 |  |
|  | OTTHUMG00000019395 |  |
|  | TTC33 |  |
|  | RNA5SP202 |  |
|  | TMSB4XP8 |  |
|  | RNY4P5 |  |
|  | SNORA2A |  |
|  | ARHGEF12 |  |
|  | HIST2H4B |  |
|  | RNU6-14P |  |
|  | PLEK |  |
|  | OTTHUMG00000163254 |  |
|  | GABARAPL2 |  |
|  | RNA5S17 |  |
|  | RNA5SP325 |  |
|  | RNA5S9 |  |
|  | ATP8A1 |  |
|  | NT5C3A |  |
|  | HEMGN |  |
|  | OTTHUMG00000035703 |  |
|  | RNA5SP355 |  |
|  | RNA5SP150 |  |
|  | OTTHUMG00000151104 |  |
|  | OTTHUMG00000019401 |  |
|  | ACAP2-IT1 |  |
|  | RNA5SP33 |  |
|  | RNU6-39P |  |
|  | RTN3 |  |
|  | RNA5SP435 |  |
|  | RNU6-27 |  |
|  | RNA5SP149 |  |
|  | PIP4K2A |  |
|  | ASAH1 |  |
|  | OTTHUMG00000018686 |  |
|  | RNU6-82P |  |
|  | BROX |  |
|  | RNU1-18P |  |
|  | RNU1-14P |  |
|  | TAOK1 |  |
|  | CD38 |  |
|  | RNU6-4 |  |
|  | DLEU2L |  |
|  | MIR15A |  |
|  | GTSF1L |  |
|  | RASGRP1 |  |
|  | WDR44 |  |
|  | SUSD1 |  |
|  | ITM2B |  |
|  | RNU6-50 |  |
|  | TBX21 |  |
|  | VAMP7 |  |
|  | CORO1C |  |
|  | OTTHUMG00000037053 |  |
|  | RNA5SP62 |  |
|  | SERPINB1 |  |
|  | RNA5SP19 |  |
|  | N4BP2L2-IT2 |  |
|  | RNU6-59P |  |
|  | OTTHUMG00000160538 |  |
|  | MIRLET7F1 |  |
|  | AKAP11-IT1 |  |
|  | HCK |  |
|  | RNY4P18 |  |
|  | CD36 |  |
|  | OTTHUMG00000161590 |  |
|  | PPP3R1 |  |
|  | RNA5SP74 |  |
|  | RANBP9 |  |
|  | ARRB1 |  |
|  | RNU6-29 |  |
|  | RNU6-7 |  |
|  | PIP5K1B |  |
|  | HIST1H4C |  |
|  | HOOK3 |  |
|  | RNY4P7 |  |
|  | RNU6-16P |  |
|  | LINC00657 |  |
|  | LUZP6 |  |
|  | HIST1H2BF |  |
|  | RNA5SP85 |  |
|  | MAP4K5 |  |
|  | UBE2K |  |
|  | SNORA59B |  |
|  | EAF2 |  |
|  | OTTHUMG00000011643 |  |
|  | NEB |  |
|  | RNA5SP399 |  |
|  | MIR566 |  |
|  | MYL12A |  |
|  | ANKRD28 |  |
|  | EYA3-IT1 |  |
|  | YPEL5 |  |
|  | BLZF1 |  |
|  | LCP2 |  |
|  | RNU6-23P |  |
|  | JAK2 |  |
|  | CASC5 |  |
|  | ANP32A-IT1 |  |
|  | OTTHUMG00000164171 |  |
|  | LRMP |  |
|  | RNY3P2 |  |
|  | DYNLL1 |  |
|  | RNA5SP242 |  |
|  | IRAK3 |  |
|  | PICALM |  |
|  | YWHAE |  |
|  | FMR1-IT1 |  |
|  | H3F3A |  |
|  | RBL1 |  |
|  | RNA5SP122 |  |
|  | SCLT1 |  |
|  | SCARNA3 |  |
|  | RNY4P19 |  |
|  | CAMK2D |  |
|  | RNU6-8 |  |
|  | RNU6-34P |  |
|  | ASAP1 |  |
|  | RNF11 |  |
|  | SKAP2 |  |
|  | RNU6-15P |  |
|  | NPTN |  |
|  | RNU6-80 |  |
|  | OTTHUMG00000073673 |  |
|  | CNST |  |
|  | SH3BGRL |  |
|  | STK39 |  |
|  | KLF3 |  |
|  | ETFA |  |
|  | MIR1273A |  |
|  | EFCAB13 |  |
|  | OST4 |  |
|  | TBC1D15 |  |
|  | HIST1H2AE |  |
|  | RNU6-37 |  |
|  | RNU6-1 |  |
|  | RNU6-11 |  |
|  | RNU1-11P |  |
|  | ROCK2 |  |
|  | CAPZA2 |  |
|  | TEFM |  |
|  | RNU6-33P |  |
|  | TMEM14E |  |
|  | RNU6-36P |  |
|  | RIT1 |  |
|  | CHML |  |
|  | SNORA11 |  |
|  | VDAC3 |  |
|  | DNAJC3 |  |
|  | SUZ12P1 |  |
|  | CLCN3 |  |
|  | RNU1-17P |  |
|  | TAS2R30 |  |
|  | OTTHUMG00000041460 |  |
|  | DENND4A |  |
|  | OTTHUMG00000160797 |  |
|  | RNU6-20 |  |
|  | IFRD1 |  |
|  | CPEB4 |  |
|  | ZNF124 |  |
|  | PRKACB |  |
|  | LOC100505794 |  |
|  | FAM3C |  |
|  | SNX18 |  |
|  | HIST1H2BC |  |
|  | RNA5SP403 |  |
|  | USP12 |  |
|  | RNU6-21P |  |
|  | MIR3613 |  |
|  | RNA5SP298 |  |
|  | KIFAP3 |  |
|  | CMIP |  |
|  | RNU6-18 |  |
|  | RNU6-17 |  |
|  | LUST |  |
|  | WWTR1-IT1 |  |
|  | RNU4-10P |  |
|  | MTRNR2L10 |  |
|  | OTTHUMG00000015592 |  |
|  | SLAIN2 |  |
|  | GPCPD1 |  |
|  | PTP4A2 |  |
|  | WHAMMP1 |  |
|  | LINC00685 |  |
|  | MBTD1 |  |
|  | ODC1 |  |
|  | RNU6-32 |  |
|  | RNU6-13 |  |
|  | RNU6-12 |  |
|  | RSF1-IT1 |  |
|  | NLK |  |
|  | RNA5SP226 |  |
|  | RNY3P9 |  |
|  | SEPT7 |  |
|  | RNY4P24 |  |
|  | MTMR12 |  |
|  | RNU6-10P |  |
|  | RNU1-10P |  |
|  | PAIP2 |  |
|  | RNA5SP335 |  |
|  | RNY4P15 |  |
|  | SCFD1 |  |
|  | RNA5SP145 |  |
|  | RNU6-43 |  |
|  | RNA5SP493 |  |
|  | RNU6-60 |  |
|  | DENND4C |  |
|  | RNA5SP141 |  |
|  | RNA5SP429 |  |
|  | OTTHUMG00000018179 |  |
|  | LOC100506060 |  |
|  | CMTM6 |  |
|  | RNU7-40P |  |
|  | RNY4P25 |  |
|  | DNAJB14 |  |
|  | HSBP1 |  |
|  | OTTHUMG00000140109 |  |
|  | IVNS1ABP |  |
|  | LYPLAL1 |  |
|  | SCARNA15 |  |
|  | ANGPTL1 |  |
|  | RC3H2 |  |
|  | SLC9A9 |  |
|  | RNA5SP290 |  |
|  | RNU6-49 |  |
|  | CHD9 |  |
|  | LINC00597 |  |
|  | RNU6-42P |  |
|  | EIF4G3 |  |
|  | AKIRIN2 |  |
|  | RNA5SP263 |  |
|  | OTTHUMG00000158562 |  |
|  | RNA5SP481 |  |
|  | MTRNR2L8 |  |
|  | RNA5SP134 |  |
|  | PHF20L1 |  |
|  | SEPT11 |  |
|  | UBE2W |  |
|  | MAP3K8 |  |
|  | ARL15 |  |
|  | RNA5SP99 |  |
|  | RABGAP1L-IT1 |  |
|  | OTTHUMG00000133759 |  |
|  | TRAM1 |  |
|  | SHOC2 |  |
|  | FBXO11 |  |
|  | PKN2 |  |
|  | OTTHUMG00000164254 |  |
|  | RNA5SP273 |  |
|  | CCDC7 |  |
|  | OTTHUMG00000151134 |  |
|  | MIR505 |  |
|  | SDCBP |  |
|  | CCDC18 |  |
|  | RNA5SP37 |  |
|  | DIAPH1 |  |
|  | OTTHUMG00000019444 |  |
|  | RNY3 |  |
|  | RSF1-IT2 |  |
|  | ARRDC3 |  |
|  | CERS6 |  |
|  | BNIP3L |  |
|  | HIST1H2BN |  |
|  | HIST1H4J |  |
|  | UBE2Q2 |  |
|  | DCAF13P3 |  |
|  | RNU2-6P |  |
|  | WHAMMP3 |  |
|  | WHAMMP2 |  |
|  | ATAD2B |  |
|  | DNMBP |  |
|  | CLIC4 |  |
|  | REPS2 |  |
|  | ARL8B |  |
|  | MAP3K5 |  |
|  | CALM2 |  |
|  | SLMAP |  |
|  | RNY4P8 |  |
|  | PDCD10 |  |
|  | RNA5SP68 |  |
|  | ZBTB1 |  |
|  | BRK1 |  |
|  | RNY3P1 |  |
|  | WASF1 |  |
|  | BAZ2B |  |
|  | OTTHUMG00000037842 |  |
|  | MPHOSPH9 |  |
|  | OTTHUMG00000152880 |  |
|  | ATG12 |  |
|  | OTTHUMG00000164037 |  |
|  | MAP1LC3B |  |
|  | MIR643 |  |
|  | RPS6KA5 |  |
|  | GLA |  |
|  | RNY4P23 |  |
|  | SUMO4 |  |
|  | OTTHUMG00000078326 |  |
|  | HM13-IT1 |  |
|  | AP1S2 |  |
|  | DNAJC1 |  |
|  | HELQ |  |
|  | OTTHUMG00000154698 |  |
|  | THUMPD2 |  |
|  | ACSL4 |  |
|  | MIR4677 |  |
|  | MPC1 |  |
|  | SMIM14 |  |
|  | LOC100506007 |  |
|  | COQ2 |  |
|  | OTTHUMG00000152506 |  |
|  | NDUFB2 |  |
|  | RNY4P17 |  |
|  | RAB4A |  |
|  | RNA5SP344 |  |
|  | STRN3 |  |
|  | TMSB4X |  |
|  | RNU6-70 |  |
|  | LOC100506985 |  |
|  | MEF2A |  |
|  | ITFG1 |  |
|  | RNVU1-9 |  |
|  | SUGT1 |  |
|  | OTTHUMG00000017742 |  |
|  | RNU6ATAC3P |  |
|  | OTTHUMG00000150514 |  |
|  | LARP4 |  |
|  | UBE2H |  |
|  | TEX21P |  |
|  | RNA5SP86 |  |
|  | RNA5SP382 |  |
|  | RNA5SP440 |  |
|  | CLEC4A |  |
|  | ATF2 |  |
|  | JHDM1D |  |
|  | EVI5 |  |
|  | TLK1 |  |
|  | KIN |  |
|  | SNRPG |  |
|  | ENDOD1 |  |
|  | RNU1-19P |  |
|  | SLC35D1 |  |
|  | RNU6-24 |  |
|  | SMAD2 |  |
|  | OTTHUMG00000017845 |  |
|  | OTTHUMG00000066284 |  |
|  | ZCCHC2 |  |
|  | ZNF518A |  |
|  | HIST1H2AI |  |
|  | OTTHUMG00000152672 |  |
|  | HMBOX1-IT1 |  |
|  | NDUFA6 |  |
|  | RNU1-1 |  |
|  | RABGAP1L |  |
|  | RNA5SP352 |  |
|  | GPD2 |  |
|  | LOC100216546 |  |
|  | CD72 |  |
|  | SNX5 |  |
|  | BPTF |  |
|  | PDS5A |  |
|  | NFATC3 |  |
|  | PPM1A |  |
|  | GK-IT1 |  |
|  | MBD5 |  |
|  | FAM188A |  |
|  | ITCH-AS1 |  |
|  | CLIP1 |  |
|  | OTTHUMG00000046310 |  |
|  | MIR4653 |  |
|  | TAB3-AS2 |  |
|  | CASP3 |  |
|  | RPAP2 |  |
|  | RHOA-IT1 |  |
|  | OTTHUMG00000040465 |  |
|  | TET2 |  |
|  | RNA5SP267 |  |
|  | GRIK1-AS2 |  |
|  | SUPT4H1 |  |
|  | DAAM1 |  |
|  | ERBB2IP |  |
|  | CCDC82 |  |
|  | MIR4659A |  |
|  | MIR628 |  |
|  | RNU6-28P |  |
|  | ACTR10 |  |
|  | WDR67 |  |
|  | RNA5SP81 |  |
|  | RAPGEF6 |  |
|  | PTBP2 |  |
|  | LOC100507254 |  |
|  | LRBA |  |
|  | RNA5SP383 |  |
|  | OTTHUMG00000162535 |  |
|  | RNA5SP50 |  |
|  | OTTHUMG00000163785 |  |
|  | RNA5SP183 |  |
|  | RNA5SP506 |  |
|  | DPRXP4 |  |
|  | ZNF791 |  |
|  | RNA5SP215 |  |
|  | DKFZP686I15217 |  |
|  | LINC00449 |  |
|  | CLYBL-IT1 |  |
|  | MACF1 |  |
|  | ERV3-1 |  |
|  | SRGAP2B |  |
|  | MIR454 |  |
|  | KMT2C |  |
|  | EMC3 |  |
|  | GLUL |  |
|  | YES1 |  |
|  | RNA5SP191 |  |
|  | RNA5SP77 |  |
|  | OTTHUMG00000055979 |  |
|  | LRRC37A |  |
|  | RNU6-35P |  |
|  | LOC100505828 |  |
|  | LOC727896 |  |
|  | FGFR1OP2 |  |
|  | CRBN |  |
|  | RHEB |  |
|  | CNIH |  |
|  | PIGN |  |
|  | RUFY1 |  |
|  | RCN1P2 |  |
|  | TRMT61B |  |
|  | OTTHUMG00000019506 |  |
|  | GSK3B |  |
|  | TBL1XR1 |  |
|  | CHIC2 |  |
|  | OTTHUMG00000164295 |  |
|  | RP2 |  |
|  | TUBB6 |  |
|  | CPD |  |
|  | UGCG |  |
|  | UBASH3B |  |
|  | TMEM165 |  |
|  | SOX4 |  |
|  | RNU1-20P |  |
|  | KIAA1468 |  |
|  | CEP57L1P1 |  |
|  | N4BP2L2 |  |
|  | VPS37A |  |
|  | OTTHUMG00000164836 |  |
|  | RASA1 |  |
|  | FAM63B |  |
|  | ZNF594 |  |
|  | KIF3B |  |
|  | OTTHUMG00000016629 |  |
|  | OTTHUMG00000003552 |  |
|  | RNA5SP389 |  |
|  | RUFY2 |  |
|  | ELF2 |  |
|  | GPR52 |  |
|  | ZNF542 |  |
|  | OTTHUMG00000153210 |  |
|  | CEP135 |  |
|  | BRWD1 |  |
|  | CD47 |  |
|  | RNU6-46P |  |
|  | RNA5SP374 |  |
|  | OTTHUMG00000057501 |  |
|  | OTTHUMG00000163720 |  |
|  | MFAP1 |  |
|  | RNY3P7 |  |
|  | RNU1-16P |  |
|  | RNY4P6 |  |
|  | BRE-AS1 |  |
|  | RNY1P5 |  |
|  | OTTHUMG00000019429 |  |
|  | HEATR5A |  |
|  | RNY4P11 |  |
|  | RNA5SP368 |  |
|  | IPMK |  |
|  | ITCH-IT1 |  |
|  | RAP2B |  |
|  | CDC27 |  |
|  | OTTHUMG00000162774 |  |
|  | RNA5SP152 |  |
|  | RNU4-8P |  |
|  | PDK1 |  |
|  | C5orf42 |  |
|  | WASL |  |
|  | DCTN4 |  |
|  | PIGF |  |
|  | SLC30A1 |  |
|  | RAB6A |  |
|  | CNEP1R1 |  |
|  | RNA5SP143 |  |
|  | GOSR1 |  |
|  | RNU6-19P |  |
|  | OTTHUMG00000160386 |  |
|  | CHMP5 |  |
|  | ZNF25 |  |
|  | DCP2 |  |
|  | RNU6-44 |  |
|  | FBXO48 |  |
|  | OTTHUMG00000057562 |  |
|  | TNFSF13B |  |
|  | KLHL5 |  |
|  | SNORA76 |  |
|  | OTTHUMG00000157398 |  |
|  | VRK1 |  |
|  | MXD1 |  |
|  | MIR4659B |  |
|  | MTMR10 |  |
|  | MIR1268A |  |
|  | POC1B |  |
|  | RNU7-23P |  |
|  | FAM73A |  |
|  | UBE2B |  |
|  | CXorf21 |  |
|  | TSC22D1 |  |
|  | OTTHUMG00000152699 |  |
|  | RNA5SP473 |  |
|  | OTTHUMG00000162817 |  |
|  | TNIK |  |
|  | B2M |  |
|  | UBE2F |  |
|  | RNU1-21P |  |
|  | KIAA1841 |  |
|  | RAB1A |  |
|  | IL24 |  |
|  | OTTHUMG00000162476 |  |
|  | RNA5SP67 |  |
|  | LOC100506190 |  |
|  | MIR320E |  |
|  | FER |  |
|  | RNA5SP173 |  |
|  | TMEM2 |  |
|  | PCMTD1 |  |
|  | BIRC6-AS1 |  |
|  | MTSS1 |  |
|  | ABI1 |  |
|  | LOC283710 |  |
|  | C7orf73 |  |
|  | OTTHUMG00000015823 |  |
|  | MIR1254-1 |  |
|  | RNA5SP225 |  |
|  | TXNDC15 |  |
|  | RNU2-4P |  |
|  | ATP2C1 |  |
|  | RNA5SP259 |  |
|  | MST4 |  |
|  | OGFRL1 |  |
|  | DCLRE1A |  |
|  | DCAF6 |  |
|  | HECTD4 |  |
|  | RNA5SP338 |  |
|  | FOXO3B |  |
|  | IQSEC1 |  |
|  | OTTHUMG00000034494 |  |
|  | RNA5SP395 |  |
|  | RNA5SP260 |  |
|  | OTTHUMG00000159252 |  |
|  | LITAF |  |
|  | ZFAND1 |  |
|  | NAPG |  |
|  | OTTHUMG00000164842 |  |
|  | IKZF2 |  |
|  | MIR320D1 |  |
|  | CDKL1 |  |
|  | MIR548O |  |
|  | RNA5SP370 |  |
|  | MIR3115 |  |
|  | KLHDC1 |  |
|  | RNA5SP463 |  |
|  | N4BP2L1 |  |
|  | GALNT3 |  |
|  | OTTHUMG00000152764 |  |
|  | DTX1 |  |
|  | C6orf163 |  |
|  | ZNF271 |  |
|  | PPP2R5A |  |
|  | RNA5SP245 |  |
|  | MIR181A1HG |  |
|  | SETD5-AS1 |  |
|  | CPEB2 |  |
|  | TCEAL3-AS1 |  |
|  | RNY3P8 |  |
|  | STAM |  |
|  | OTTHUMG00000086635 |  |
|  | EIF4E |  |
|  | EYA3 |  |
|  | REV3L-IT1 |  |
|  | OTTHUMG00000152004 |  |
|  | FAM179B |  |
|  | CAAP1 |  |
|  | PMS2P1 |  |
|  | SSFA2 |  |
|  | SPAG9 |  |
|  | CDC42 |  |
|  | OTTHUMG00000162573 |  |
|  | JKAMP |  |
|  | HEXIM1 |  |
|  | UHRF2 |  |
|  | ATF7IP2 |  |
|  | OTTHUMG00000162284 |  |
|  | C18orf54 |  |
|  | PTK2 |  |
|  | CKLF |  |
|  | LRCH3 |  |
|  | PAG1 |  |
|  | OTTHUMG00000150939 |  |
|  | CMC2 |  |
|  | FAM46A |  |
|  | TMEM167A |  |
|  | SDCCAG8 |  |
|  | RNY4P10 |  |
|  | MIR597 |  |
|  | YWHAZ |  |
|  | LOC729603 |  |
|  | UCHL3 |  |
|  | HERC1 |  |
|  | RNA5SP494 |  |
|  | LOC100507117 |  |
|  | LOC100506051 |  |
|  | GDPD1 |  |
|  | ATP5L2 |  |
|  | ARRDC5 |  |
|  | STX7 |  |
|  | RNF115 |  |
|  | ZNF763 |  |
|  | RCHY1 |  |
|  | TAF11 |  |
|  | PCDH9 |  |
|  | POLR3G |  |
|  | PPM1B |  |
|  | DNAJB6 |  |
|  | RNA5SP250 |  |
|  | OTTHUMG00000155934 |  |
|  | RAD23B |  |
|  | RNU6-45P |  |
|  | ATP5L |  |
|  | RNU7-57P |  |
|  | SLC12A2 |  |
|  | OTTHUMG00000022274 |  |
|  | KLHL2 |  |
|  | RNY4P14 |  |
|  | RNA5SP185 |  |
|  | MIR1285-2 |  |
|  | VPS41 |  |
|  | ATG5 |  |
|  | RAB14 |  |
|  | ZZEF1 |  |
|  | YBX3P1 |  |
|  | RFTN1 |  |
|  | HES1 |  |
|  | SCRN3 |  |
|  | OTTHUMG00000161597 |  |
|  | LPP |  |
|  | BARD1 |  |
|  | TBC1D1 |  |
|  | TMEM41B |  |
|  | OTTHUMG00000155268 |  |
|  | RNA5SP365 |  |
|  | FNIP1 |  |
|  | RNA5SP132 |  |
|  | EGFLAM-AS4 | down |
|  | OTTHUMG00000132778 |  |
|  | OTTHUMG00000037849 |  |
|  | KRTAP21-3 |  |
|  | OTTHUMG00000152489 |  |
|  | OTTHUMG00000017314 |  |
|  | OTTHUMG00000037619 |  |
|  | OTTHUMG00000164050 |  |
|  | OTTHUMG00000163875 |  |
|  | OTTHUMG00000161775 |  |
|  | OTTHUMG00000150950 |  |
|  | ZPLD1 |  |
|  | IGHJ1 |  |
|  | OTTHUMG00000153044 |  |
|  | APOOP5 |  |
|  | FLJ42842 |  |
|  | LOC100131289 |  |
|  | OTTHUMG00000015980 |  |
|  | OTTHUMG00000163287 |  |
|  | OTTHUMG00000154907 |  |
|  | OTTHUMG00000037078 |  |
|  | OTTHUMG00000163446 |  |
|  | OTTHUMG00000163830 |  |
|  | OR2G6 |  |
|  | GIPC2 |  |
|  | LINC00445 |  |
|  | MIR4446 |  |
|  | MIR489 |  |
|  | KRT25 |  |
|  | KRTAP20-3 |  |
|  | OTTHUMG00000163757 |  |
|  | MIR3177 |  |
|  | CDC20P1 |  |
|  | MIR511-1 |  |
|  | MIR511-2 |  |
|  | OTTHUMG00000161058 |  |
|  | OTTHUMG00000162998 |  |
|  | OTTHUMG00000155483 |  |
|  | XIRP2-AS1 |  |
|  | FER1L6-AS1 |  |
|  | MIR1294 |  |
|  | OTTHUMG00000154132 |  |
|  | MIR3158-2 |  |
|  | OTTHUMG00000040691 |  |
|  | LINC00502 |  |
|  | OTTHUMG00000154842 |  |
|  | OTTHUMG00000155418 |  |
|  | OTTHUMG00000155340 |  |
|  | MT4 |  |
|  | ZNF165 |  |
|  | DEFB121 |  |
|  | CNR1 |  |
|  | LOC100507030 |  |
|  | OTTHUMG00000017942 |  |
|  | LINC00361 |  |
|  | C14orf132 |  |
|  | OTTHUMG00000154871 |  |
|  | RNU105C |  |
|  | OTTHUMG00000160372 |  |
|  | OTTHUMG00000078483 |  |
|  | C7orf69 |  |
|  | HIST1H2AA |  |
|  | OTTHUMG00000014659 |  |
|  | C3orf70 |  |
|  | OTTHUMG00000158541 |  |
|  | OTTHUMG00000144166 |  |
|  | OTTHUMG00000037758 |  |
|  | OTTHUMG00000161802 |  |
|  | OTTHUMG00000153043 |  |
|  | OTTHUMG00000160203 |  |
|  | MIR4486 |  |
|  | LRRC52 |  |
|  | MYF5 |  |
|  | LINC00411 |  |
|  | OTTHUMG00000161777 |  |
|  | OR5K3 |  |
|  | OTTHUMG00000158446 |  |
|  | MIR575 |  |
|  | OTTHUMG00000020240 |  |
|  | OTTHUMG00000164708 |  |
|  | ZNF705E |  |
|  | OTTHUMG00000160828 |  |
|  | MIR4289 |  |
|  | OTTHUMG00000156394 |  |
|  | OTTHUMG00000162779 |  |
|  | C16orf47 |  |
|  | ST6GAL2-IT1 |  |
|  | OTTHUMG00000163868 |  |
|  | EML6 |  |
|  | SRRM5 |  |
|  | OTTHUMG00000031835 |  |
|  | OTTHUMG00000020435 |  |
|  | OTTHUMG00000009400 |  |
|  | OTTHUMG00000164044 |  |
|  | OTTHUMG00000015708 |  |
|  | MT1P2 |  |
|  | FAM24A |  |
|  | OTTHUMG00000162457 |  |
|  | OTTHUMG00000151415 |  |
|  | CHL1-AS1 |  |
|  | MIR146A |  |
|  | OTTHUMG00000037001 |  |
|  | MYF6 |  |
|  | RNA5SP272 |  |
|  | OR5P3 |  |
|  | OTTHUMG00000154248 |  |
|  | OTTHUMG00000015384 |  |
|  | OTTHUMG00000019075 |  |
|  | LOC100506851 |  |
|  | OTTHUMG00000161627 |  |
|  | OTTHUMG00000164486 |  |
|  | OTTHUMG00000162204 |  |
|  | TPI1P3 |  |
|  | OTTHUMG00000161633 |  |
|  | SPRR3 |  |
|  | REG1A |  |
|  | KRTAP4-3 |  |
|  | PLCH1-AS2 |  |
|  | GUSBP5 |  |
|  | HMGN5 |  |
|  | OTTHUMG00000164291 |  |
|  | LOC100506895 |  |
|  | OTTHUMG00000161063 |  |
|  | OTTHUMG00000037423 |  |
|  | OTTHUMG00000014652 |  |
|  | SCEL-AS1 |  |
|  | MPRIP-AS1 |  |
|  | OTTHUMG00000000451 |  |
|  | OTTHUMG00000003875 |  |
|  | OTTHUMG00000160990 |  |
|  | LOC643770 |  |
|  | FABP3P2 |  |
|  | SLC9A9-AS2 |  |
|  | SPERT |  |
|  | DNAH10OS |  |
|  | OTTHUMG00000155014 |  |
|  | OTTHUMG00000008128 |  |
|  | ERC2-IT1 |  |
|  | OTTHUMG00000163148 |  |
|  | OR51I1 |  |
|  | OTTHUMG00000035804 |  |
|  | OTTHUMG00000019009 |  |
|  | CMA1 |  |
|  | OR2T11 |  |
|  | LINC00626 |  |
|  | OTTHUMG00000153891 |  |
|  | OTTHUMG00000162302 |  |
|  | IFITM5 |  |
|  | OTTHUMG00000066356 |  |
|  | OTTHUMG00000008421 |  |
|  | KRTAP4-6 |  |
|  | OOEP-AS1 |  |
|  | OTTHUMG00000162592 |  |
|  | OTTHUMG00000144163 |  |
|  | OTTHUMG00000161032 |  |
|  | LOC643733 |  |
|  | OTTHUMG00000164860 |  |
|  | MLIP-IT1 |  |
|  | MIR1260A |  |
|  | OTTHUMG00000152017 |  |
|  | OTTHUMG00000151461 |  |
|  | OTTHUMG00000162820 |  |
|  | OTTHUMG00000159355 |  |
|  | OTTHUMG00000041351 |  |
|  | MIR433 |  |
|  | LOC100129636 |  |
|  | OTTHUMG00000163152 |  |
|  | OTTHUMG00000078332 |  |
|  | OTTHUMG00000011637 |  |
|  | LOC100287225 |  |
|  | OTTHUMG00000165067 |  |
|  | CAPNS2 |  |
|  | AKR1B1P6 |  |
|  | LOC283856 |  |
|  | OTTHUMG00000154316 |  |
|  | OR10A6 |  |
|  | OTTHUMG00000163475 |  |
|  | TECRP1 |  |
|  | KRTAP9-9 |  |
|  | OTTHUMG00000058802 |  |
|  | OTTHUMG00000155064 |  |
|  | MIR874 |  |
|  | OTTHUMG00000017366 |  |
|  | MIR4640 |  |
|  | OTTHUMG00000162456 |  |
|  | MIR4727 |  |
|  | OTTHUMG00000015106 |  |
|  | OTTHUMG00000015645 |  |
|  | OTTHUMG00000153737 |  |
|  | OTTHUMG00000017546 |  |
|  | OR1C1 |  |
|  | OR1N2 |  |
|  | OTTHUMG00000161561 |  |
|  | OTTHUMG00000000481 |  |
|  | OTTHUMG00000164494 |  |
|  | OTTHUMG00000015667 |  |
|  | OTTHUMG00000163069 |  |
|  | LINC00670 |  |
|  | CYP2B7P1 |  |
|  | CARTPT |  |
|  | OTTHUMG00000153667 |  |
|  | LOC285000 |  |
|  | OR5AS1 |  |
|  | MIR4739 |  |
|  | OTTHUMG00000152744 |  |
|  | OTTHUMG00000158073 |  |
|  | CSTF3-AS1 |  |
|  | OTTHUMG00000017554 |  |
|  | OTTHUMG00000163511 |  |
|  | KRTAP25-1 |  |
|  | OTTHUMG00000164278 |  |
|  | OTTHUMG00000074543 |  |
|  | LOC153910 |  |
|  | DEFB116 |  |
|  | MIR504 |  |
|  | OR8G1 |  |
|  | PARM1 |  |
|  | PRHOXNB |  |
|  | MAGI1-AS1 |  |
|  | OTTHUMG00000153231 |  |
|  | LOC728084 |  |
|  | OTTHUMG00000034556 |  |
|  | OTTHUMG00000159952 |  |
|  | OTTHUMG00000015399 |  |
|  | OTTHUMG00000153504 |  |
|  | OTTHUMG00000155424 |  |
|  | OTTHUMG00000152019 |  |
|  | LOC158434 |  |
|  | MIR4661 |  |
|  | OTTHUMG00000037400 |  |
|  | MIR605 |  |
|  | MIR4290 |  |
|  | ECRP |  |
|  | OTTHUMG00000152589 |  |
|  | OTTHUMG00000153390 |  |
|  | OTTHUMG00000151319 |  |
|  | OTTHUMG00000160243 |  |
|  | OR2T3 |  |
|  | HPYR1 |  |
|  | OTTHUMG00000162202 |  |
|  | OR10G4 |  |
|  | OTTHUMG00000021313 |  |
|  | PCA3 |  |
|  | KU-MEL-3 |  |
|  | GJA8 |  |
|  | OTTHUMG00000017662 |  |
|  | OR10G8 |  |
|  | DEFB125 |  |
|  | OTTHUMG00000164862 |  |
|  | TPI1P2 |  |
|  | OTTHUMG00000161691 |  |
|  | PCAT4 |  |
|  | KRTAP3-1 |  |
|  | OR4C15 |  |
|  | OTTHUMG00000150945 |  |
|  | TNR-IT1 |  |
|  | KPRP |  |
|  | LOC100506937 |  |
|  | PRAMEF17 |  |
|  | OTTHUMG00000163190 |  |
|  | OTTHUMG00000163524 |  |
|  | OTTHUMG00000160355 |  |
|  | OR5H6 |  |
|  | OTTHUMG00000018267 |  |
|  | LOC100505799 |  |
|  | MIR218-1 |  |
|  | OTTHUMG00000035527 |  |
|  | MGC24103 |  |
|  | OTTHUMG00000161739 |  |
|  | OTTHUMG00000066035 |  |
|  | OTTHUMG00000017902 |  |
|  | MIR340 |  |
|  | HCG4 |  |
|  | TRBV6-7 |  |
|  | OTTHUMG00000017628 |  |
|  | OTTHUMG00000163927 |  |
|  | PHEX-AS1 |  |
|  | EGFLAM-AS1 |  |
|  | USP17L4 |  |
|  | MRGPRX2 |  |
|  | OTTHUMG00000153695 |  |
|  | OR9A2 |  |
|  | TRAV14DV4 |  |
|  | OTTHUMG00000157228 |  |
|  | OTTHUMG00000163335 |  |
|  | OTTHUMG00000014196 |  |
|  | OTTHUMG00000153160 |  |
|  | OTTHUMG00000014664 |  |
|  | SPATA31C2 |  |
|  | OTTHUMG00000155475 |  |
|  | OTTHUMG00000164017 |  |
|  | OTTHUMG00000163632 |  |
|  | OTTHUMG00000155049 |  |
|  | MIR325 |  |
|  | PRAMEF16 |  |
|  | BACH1-AS1 |  |
|  | OTTHUMG00000016533 |  |
|  | OTTHUMG00000164831 |  |
|  | OTTHUMG00000162519 |  |
|  | IFNG-AS1 |  |
|  | OTTHUMG00000156378 |  |
|  | OTTHUMG00000035542 |  |
|  | DEFA1B |  |
|  | C15orf32 |  |
|  | OTTHUMG00000158223 |  |
|  | OTTHUMG00000013934 |  |
|  | OR4F21 |  |
|  | OTTHUMG00000164266 |  |
|  | OTTHUMG00000151774 |  |
|  | OTTHUMG00000158362 |  |
|  | MIR3976 |  |
|  | LINC00700 |  |
|  | OTTHUMG00000002706 |  |
|  | RNA5SP31 |  |
|  | OTTHUMG00000031780 |  |
|  | OTTHUMG00000017936 |  |
|  | TXNIP |  |
|  | OTTHUMG00000078370 |  |
|  | OR4C13 |  |
|  | OTTHUMG00000155735 |  |
|  | OTTHUMG00000154730 |  |
|  | OTTHUMG00000132850 |  |
|  | LOC100996455 |  |
|  | OTTHUMG00000150135 |  |
|  | PBOV1 |  |
|  | OTTHUMG00000160485 |  |
|  | KRTAP20-1 |  |
|  | ZSCAN12P1 |  |
|  | OTTHUMG00000163795 |  |
|  | OTTHUMG00000015798 |  |
|  | OR5A1 |  |
|  | OTTHUMG00000153616 |  |
|  | OTTHUMG00000164156 |  |
|  | OTTHUMG00000090613 |  |
|  | OTTHUMG00000153411 |  |
|  | OTTHUMG00000165097 |  |
|  | OR2A5 |  |
|  | OTTHUMG00000156392 |  |
|  | KIRREL3-AS2 |  |
|  | OTTHUMG00000155731 |  |
|  | OR13J1 |  |
|  | OTTHUMG00000019205 |  |
|  | MIR138-1 |  |
|  | OTTHUMG00000150954 |  |
|  | OTTHUMG00000039875 |  |
|  | LOC100288814 |  |
|  | OTTHUMG00000161004 |  |
|  | OR2M2 |  |
|  | OTTHUMG00000037874 |  |
|  | OTTHUMG00000040080 |  |
|  | MIR4711 |  |
|  | OTTHUMG00000031374 |  |
|  | NDP-AS1 |  |
|  | OTTHUMG00000008097 |  |
|  | OTTHUMG00000001219 |  |
|  | OTTHUMG00000016983 |  |
|  | OTTHUMG00000015256 |  |
|  | OTTHUMG00000032494 |  |
|  | MRGPRX4 |  |
|  | LOC100996616 |  |
|  | MIR4474 |  |
|  | FLJ45831 |  |
|  | OTTHUMG00000151328 |  |
|  | OR5B3 |  |
|  | OR4Q3 |  |
|  | OTTHUMG00000078275 |  |
|  | MIR2116 |  |
|  | OTTHUMG00000163814 |  |
|  | OR2T6 |  |
|  | OR4M2 |  |
|  | TTC3-AS1 |  |
|  | VSIG1 |  |
|  | OTTHUMG00000016695 |  |
|  | OR5M10 |  |
|  | ABCC6P2 |  |
|  | CT45A6 |  |
|  | OTTHUMG00000153603 |  |
|  | OTTHUMG00000155708 |  |
|  | OR4D11 |  |
|  | KRTAP11-1 |  |
|  | OTTHUMG00000152476 |  |
|  | OTTHUMG00000155472 |  |
|  | MIR802 |  |
|  | OTTHUMG00000160893 |  |
|  | VTRNA2-1 |  |
|  | MYO16-AS1 |  |
|  | OTTHUMG00000161773 |  |
|  | OTTHUMG00000043432 |  |
|  | OR5E1P |  |
|  | OTTHUMG00000161729 |  |
|  | OTTHUMG00000015988 |  |
|  | HAO2-IT1 |  |
|  | OR8H1 |  |
|  | OTTHUMG00000161340 |  |
|  | SCGB1D1 |  |
|  | RPTN |  |
|  | CT45A2 |  |
|  | OTTHUMG00000086700 |  |
|  | OTTHUMG00000163670 |  |
|  | OR51A4 |  |
|  | MIR216A |  |
|  | OR2M3 |  |
|  | USP17L2 |  |
|  | OR2T27 |  |
|  | TRAV8-7 |  |
|  | KRTAP4-4 |  |
|  | OTTHUMG00000151708 |  |
|  | RNA5SP222 |  |
|  | LOC340074 |  |
|  | OTTHUMG00000163984 |  |
|  | POU5F1B |  |
|  | OTTHUMG00000019201 |  |
|  | OR51F1 |  |
|  | TRAJ14 |  |
|  | OTTHUMG00000018018 |  |
|  | OR5A2 |  |
|  | OTTHUMG00000013474 |  |
|  | OR1J4 |  |
|  | FAM71C |  |
|  | OR4F17 |  |
|  | OR1D5 |  |
|  | OR51C1P |  |
|  | OTTHUMG00000150319 |  |
|  | OR6C3 |  |
|  | OR4X2 |  |
|  | OTTHUMG00000163339 |  |
|  | OR4A15 |  |
|  | OR4F6 |  |
|  | OR4N2 |  |
|  | OTTHUMG00000014787 |  |
|  | RNA5SP104 |  |
|  | OR5D18 |  |
|  | OR8A1 |  |
|  | OTTHUMG00000037063 |  |
|  | ATP2B2-IT2 |  |
|  | MIR4320 |  |
|  | TAAR3 |  |
|  | OTTHUMG00000163460 |  |
|  | OR4F4 |  |
|  | OR9A1P |  |
|  | OR4F5 |  |
| CD4^+^ T cells | FGL2 | up |
|  | CYBB |  |
|  | CLEC12A |  |
|  | RGS18 |  |
|  | MNDA |  |
|  | IRAK3 |  |
|  | IFI30 |  |
|  | TYROBP |  |
|  | MPEG1 |  |
|  | IGSF6 |  |
|  | CLEC7A |  |
|  | HLA-DRA |  |
|  | LYN |  |
|  | LRRK2 |  |
|  | MS4A7 |  |
|  | MIR21 |  |
|  | HLA-DRB4 |  |
|  | DUSP6 |  |
|  | FCER1G |  |
|  | PLEK |  |
|  | PLXDC2 |  |
|  | VCAN |  |
|  | FCGR2C |  |
|  | DMXL2 |  |
|  | IL13RA1 |  |
|  | SCIMP |  |
|  | P2RY13 |  |
|  | RNASE6 |  |
|  | AOAH |  |
|  | FCGR3A |  |
|  | HLA-DPA1 |  |
|  | GAPT |  |
|  | LPCAT2 |  |
|  | MS4A6A |  |
|  | KCTD12 |  |
|  | CCDC88A |  |
|  | RAB31 |  |
|  | CD68 |  |
|  | CPPED1 |  |
|  | NCF2 |  |
|  | CD36 |  |
|  | CCDC50 |  |
|  | OGFRL1 |  |
|  | CD86 |  |
|  | ZEB2 |  |
|  | BTK |  |
|  | HLA-DRB5 |  |
|  | CD14 |  |
|  | CD74 |  |
|  | TLR2 |  |
|  | LY86 |  |
|  | HLA-DRB1 |  |
|  | MS4A14 |  |
|  | STX11 |  |
|  | OTTHUMG00000164295 |  |
|  | PLBD1 |  |
|  | CTSS |  |
|  | PSAP |  |
|  | IGJ |  |
|  | FCGR2A |  |
|  | EMR3 |  |
|  | FCER1A |  |
|  | PECAM1 |  |
|  | S100A12 |  |
|  | ADRBK2 |  |
|  | HLA-DMA |  |
|  | JAK2 |  |
|  | LYST |  |
|  | PDK4 |  |
|  | FAM49A |  |
|  | CLEC4A |  |
|  | SULF2 |  |
|  | CD180 |  |
|  | RGS2 |  |
|  | TLR6 |  |
|  | HLA-DMB |  |
|  | HCK |  |
|  | TLR7 |  |
|  | LYZ |  |
|  | S100A9 |  |
|  | TGFBI |  |
|  | TLR8 |  |
|  | MIR644A |  |
|  | SYK |  |
|  | SLAMF7 |  |
|  | SAT1 |  |
|  | CX3CR1 |  |
|  | CD93 |  |
|  | FCN1 |  |
|  | IFNGR1 |  |
|  | IRF8 |  |
|  | NCF1B |  |
|  | NCF1C |  |
|  | NLRC4 |  |
|  | CCR1 |  |
|  | CLEC4E |  |
|  | ITGAX |  |
|  | RNF130 |  |
|  | WARS |  |
|  | ACAP2-IT1 |  |
|  | TBC1D9 |  |
|  | LOC100507639 |  |
|  | PIK3AP1 |  |
|  | PLXNC1 |  |
|  | PAK1 |  |
|  | F13A1 |  |
|  | MCTP1 |  |
|  | DOCK5 |  |
|  | HIST1H3H |  |
|  | LILRA1 |  |
|  | LACTB |  |
|  | APLP2 |  |
|  | CSTA |  |
|  | PYGL |  |
|  | CORO1C |  |
|  | MEFV |  |
|  | P2RY14 |  |
|  | CSF1R |  |
|  | MYOF |  |
|  | TLR4 |  |
|  | MIR223 |  |
|  | ASAH1 |  |
|  | CD1C |  |
|  | C19orf38 |  |
|  | SLC7A7 |  |
|  | MS4A4E |  |
|  | KYNU |  |
|  | LRRC25 |  |
|  | TNFSF13B |  |
|  | GZMB |  |
|  | CXCR2P1 |  |
|  | FCGR3B |  |
|  | GNB4 |  |
|  | SORT1 |  |
|  | ATP6V1B2 |  |
|  | MIR4434 |  |
|  | DAPP1 |  |
|  | STX7 |  |
|  | FPR3 |  |
|  | C9orf72 |  |
|  | ANXA5 |  |
|  | SPOPL |  |
|  | CXorf21 |  |
|  | MARCKS |  |
|  | ITGAM |  |
|  | GZMA |  |
|  | CSF2RA |  |
|  | CHST15 |  |
|  | MIR15A |  |
|  | MIS18BP1 |  |
|  | CLEC4C |  |
|  | GRN |  |
|  | CIITA |  |
|  | GNS |  |
|  | CTSC |  |
|  | FGR |  |
|  | DKFZP586I1420 |  |
|  | KIAA1598 |  |
|  | AP1S2 |  |
|  | PLA2G7 |  |
|  | METTL7A |  |
|  | PTPN12 |  |
|  | PSTPIP2 |  |
|  | CASP1 |  |
|  | LRP1 |  |
|  | AMICA1 |  |
|  | OTTHUMG00000161550 |  |
|  | MYO1F |  |
|  | IL3RA |  |
|  | HERPUD1 |  |
|  | CAT |  |
|  | FGD4 |  |
|  | OSBPL11 |  |
|  | WSB1 |  |
|  | P2RX1 |  |
|  | ANPEP |  |
|  | QKI |  |
|  | EVI5 |  |
|  | ENTPD1 |  |
|  | ANKRD10-IT1 |  |
|  | LILRB2 |  |
|  | GRIK1-AS2 |  |
|  | FCGR2B |  |
|  | SKAP2 |  |
|  | HLA-DRB6 |  |
|  | ALOX5 |  |
|  | SCARB2 |  |
|  | DAPK1 |  |
|  | SCPEP1 |  |
|  | MIR548C |  |
|  | NOTCH2 |  |
|  | TCF4 |  |
|  | COTL1 |  |
|  | SCARNA18 |  |
|  | DIAPH2 |  |
|  | NAIP |  |
|  | C3AR1 |  |
|  | ARRB1 |  |
|  | CPVL |  |
|  | PILRA |  |
|  | ANP32A-IT1 |  |
|  | PICALM |  |
|  | SNORD89 |  |
|  | SRGN |  |
|  | NAGA |  |
|  | ANXA2P2 |  |
|  | SLCO4C1 |  |
|  | RHOQ |  |
|  | NPC2 |  |
|  | VMP1 |  |
|  | PTPRE |  |
|  | FAM198B |  |
|  | FAR1 |  |
|  | YBX3 |  |
|  | PLSCR1 |  |
|  | APAF1 |  |
|  | MIR142 |  |
|  | BAZ2B |  |
|  | NAPSB |  |
|  | HNMT |  |
|  | VTRNA1-1 |  |
|  | OTTHUMG00000149581 |  |
|  | ARHGAP18 |  |
|  | KMO |  |
|  | RP2 |  |
|  | SCARNA4 |  |
|  | RNA5SP363 |  |
|  | KDM1B |  |
|  | CCR2 |  |
|  | C5AR1 |  |
|  | LTA4H |  |
|  | PEA15 |  |
|  | PARP14 |  |
|  | CTSH |  |
|  | ANXA2 |  |
|  | LIPN |  |
|  | CST3 |  |
|  | MAN1A1 |  |
|  | AQP9 |  |
|  | RTN3 |  |
|  | FTL |  |
|  | OTTHUMG00000041327 |  |
|  | HLA-DQA1 |  |
|  | MEGF9 |  |
|  | SIGLEC9 |  |
|  | HLA-DPB1 |  |
|  | ATP8B4 |  |
|  | TPMT |  |
|  | BMP2K |  |
|  | MEF2C |  |
|  | ANKRD50 |  |
|  | SUSD1 |  |
|  | FTLP3 |  |
|  | PTPRJ |  |
|  | PLCG2 |  |
|  | ALDH2 |  |
|  | MOB1B |  |
|  | SLC20A1 |  |
|  | AGTPBP1 |  |
|  | ACSL4 |  |
|  | UGCG |  |
|  | OTTHUMG00000018686 |  |
|  | FOXP1-IT1 |  |
|  | TCF7L2 |  |
|  | MANBA |  |
|  | HLA-DQB1 |  |
|  | ATP6V1A |  |
|  | CHD9 |  |
|  | EMR1 |  |
|  | SIDT2 |  |
|  | LUZP6 |  |
|  | B4GALT5 |  |
|  | SNX10 |  |
|  | CD300E |  |
|  | SLFN11 |  |
|  | CMTM6 |  |
|  | TNFAIP2 |  |
|  | RIOK3 |  |
|  | WDFY3 |  |
|  | OTTHUMG00000066283 |  |
|  | SLC15A4 |  |
|  | MIR612 |  |
|  | EFHD2 |  |
|  | CPNE8 |  |
|  | MAP3K5 |  |
|  | UHRF1BP1L |  |
|  | SCLT1 |  |
|  | ST8SIA4 |  |
|  | PIK3CG |  |
|  | OTTHUMG00000164171 |  |
|  | MIR3140 |  |
|  | PPT1 |  |
|  | GZMH |  |
|  | SNX18 |  |
|  | OTTHUMG00000161551 |  |
|  | CD163 |  |
|  | OTTHUMG00000011643 |  |
|  | GCA |  |
|  | OAZ1 |  |
|  | OTTHUMG00000151134 |  |
|  | UBE2D1 |  |
|  | P2RX7 |  |
|  | ZSWIM6 |  |
|  | LILRB1 |  |
|  | REL |  |
|  | DPRXP4 |  |
|  | PTPLAD2 |  |
|  | FLT3 |  |
|  | GPR34 |  |
|  | COX8A |  |
|  | RPS15AP10 |  |
|  | NAGK |  |
|  | EMR2 |  |
|  | PRKCD |  |
|  | HLA-DQA2 |  |
|  | ARPC3 |  |
|  | CD58 |  |
|  | ZNF106 |  |
|  | TET2 |  |
|  | GALNT3 |  |
|  | CYB5R4 |  |
|  | ARHGAP26 |  |
|  | CARD16 |  |
|  | RAP2A |  |
|  | TNFRSF1B |  |
|  | STK38L |  |
|  | NAAA |  |
|  | CLIC4 |  |
|  | BCL6 |  |
|  | KPNA2 |  |
|  | FCHO2 |  |
|  | LILRA2 |  |
|  | IFNGR2 |  |
|  | AGO4 |  |
|  | AHR |  |
|  | PGD |  |
|  | SLC31A1 |  |
|  | GOLIM4 |  |
|  | DRAM2 |  |
|  | ZCCHC6 |  |
|  | TXN |  |
|  | SERPINA1 |  |
|  | ATP6V0A1 |  |
|  | MBOAT1 |  |
|  | HNRNPU-AS1 |  |
|  | SRGAP2B |  |
|  | RNA5SP348 |  |
|  | TLR1 |  |
|  | SPPL2A |  |
|  | ME2 |  |
|  | TFEC |  |
|  | ACPP |  |
|  | FAM105A |  |
|  | PSEN1 |  |
|  | CTBS |  |
|  | ACER3 |  |
|  | USP32 |  |
|  | CLEC10A |  |
|  | RASSF2 |  |
|  | ARRDC4 |  |
|  | EAF2 |  |
|  | CLTC |  |
|  | GAB3 |  |
|  | LST1 |  |
|  | SKIL |  |
|  | PRF1 |  |
|  | CYP1B1 |  |
|  | HIST2H2BE |  |
|  | LAMP2 |  |
|  | TNFSF10 |  |
|  | VCL |  |
|  | RNA5SP307 |  |
|  | SLC16A6 |  |
|  | ZFAND5 |  |
|  | LOC100129034 |  |
|  | RNF13 |  |
|  | APOBEC3A |  |
|  | UBE2W |  |
|  | LY96 |  |
|  | CTSZ |  |
|  | PRCP |  |
|  | SNAP23 |  |
|  | CD300A |  |
|  | SWAP70 |  |
|  | MIR4295 |  |
|  | HLA-DPB2 |  |
|  | SSFA2 |  |
|  | VAV3 |  |
|  | AIF1 |  |
|  | IL18 |  |
|  | CREG1 |  |
|  | PLA2G4A |  |
|  | PAK2 |  |
|  | SNAPC3 |  |
|  | FCGR1C |  |
|  | LPGAT1 |  |
|  | OTTHUMG00000034984 |  |
|  | OTTHUMG00000041460 |  |
|  | HAVCR2 |  |
|  | BLNK |  |
|  | RNA5SP243 |  |
|  | ATP10D |  |
|  | GABARAP |  |
|  | SLC30A1 |  |
|  | CALM2 |  |
|  | HEXB |  |
|  | CPD |  |
|  | FCGR1A |  |
|  | TBK1 |  |
|  | SLC9A7P1 |  |
|  | TMEM165 |  |
|  | NPC1 |  |
|  | AKIRIN2 |  |
|  | CRYBG3 |  |
|  | MTHFD2 |  |
|  | OTTHUMG00000162862 |  |
|  | SDCBP |  |
|  | FAM129A |  |
|  | ZFYVE16 |  |
|  | RNU7-40P |  |
|  | GCH1 |  |
|  | COQ2 |  |
|  | COX5A |  |
|  | RHOB |  |
|  | SNORA31 |  |
|  | MYO5A |  |
|  | RB1 |  |
|  | NUMB |  |
|  | FKBP15 |  |
|  | FPR1 |  |
|  | USP15 |  |
|  | PHKB |  |
|  | SPG11 |  |
|  | FPR2 |  |
|  | SPTLC2 |  |
|  | CCL5 |  |
|  | HOOK3 |  |
|  | SH3BGRL |  |
|  | CAPZA2 |  |
|  | OTTHUMG00000163389 |  |
|  | RNF144B |  |
|  | CXCL16 |  |
|  | GPR137B |  |
|  | ATG3 |  |
|  | TUBA1B |  |
|  | DNAJC3 |  |
|  | ERP44 |  |
|  | MB21D1 |  |
|  | OTTHUMG00000160797 |  |
|  | DLEU2L |  |
|  | TM9SF2 |  |
|  | FGFBP2 |  |
|  | HIST1H2AC |  |
|  | ETV6 |  |
|  | HIATL1 |  |
|  | FCGR1B |  |
|  | RCOR1 |  |
|  | TARP |  |
|  | CNEP1R1 |  |
|  | SEC11A |  |
|  | OTTHUMG00000021796 |  |
|  | RCBTB2 |  |
|  | LILRA4 |  |
|  | RAB10 |  |
|  | PSMA2 |  |
|  | HSPA1B |  |
|  | ARAP2 |  |
|  | TM6SF1 |  |
|  | RAB11FIP1 |  |
|  | SESTD1 |  |
|  | PGM2 |  |
|  | FGD2 |  |
|  | CLEC4D |  |
|  | N4BP2L2-IT2 |  |
|  | RAC1P2 |  |
|  | ATP2B1 |  |
|  | C1orf162 |  |
|  | KIAA1033 |  |
|  | DYNLT1 |  |
|  | DPYD |  |
|  | ETS2 |  |
|  | HIST2H4B |  |
|  | SAMHD1 |  |
|  | OTTHUMG00000162912 |  |
|  | TALDO1 |  |
|  | TNFSF13 |  |
|  | VNN1 |  |
|  | DNM1L |  |
|  | MIR590 |  |
|  | GIT2 |  |
|  | RTN3P1 |  |
|  | UBE2A |  |
|  | MLKL |  |
|  | BID |  |
|  | CSF3R |  |
|  | ADAP2 |  |
|  | RNU6-51 |  |
|  | SH2B3 |  |
|  | OTTHUMG00000018491 |  |
|  | SNX27 |  |
|  | TANK |  |
|  | UBE2J1 |  |
|  | PRR5L |  |
|  | SRGAP2 |  |
|  | MIR4802 |  |
|  | IDH3A |  |
|  | PELI1 |  |
|  | AGPS |  |
|  | HLA-DRB3 |  |
|  | IPMK |  |
|  | MIR4668 |  |
|  | MIR1281 |  |
|  | TMEM170B |  |
|  | CST7 |  |
|  | RNF141 |  |
|  | DRAM1 |  |
|  | TMEM33 |  |
|  | RNU4-9P |  |
|  | LGALS1 |  |
|  | UBE2R2 |  |
|  | CECR1 |  |
|  | SIRPB1 |  |
|  | EIF4G3 |  |
|  | ADAM17 |  |
|  | RNA5SP217 |  |
|  | DPYSL2 |  |
|  | IL18RAP |  |
|  | SOD2 |  |
|  | RAB3D |  |
|  | CNTLN |  |
|  | LAP3 |  |
|  | CMKLR1 |  |
|  | TMEM62 |  |
|  | OTTHUMG00000150514 |  |
|  | H3F3A |  |
|  | NCKAP1L |  |
|  | AKAP11-IT1 |  |
|  | CTNNA1 |  |
|  | GLIPR1 |  |
|  | CHP1 |  |
|  | TMBIM4 |  |
|  | LOC100506115 |  |
|  | PPP4R1 |  |
|  | IQGAP2 |  |
|  | RBM47 |  |
|  | MOB1A |  |
|  | HSPA1A |  |
|  | BST1 |  |
|  | TEFM |  |
|  | NCF1 |  |
|  | OTTHUMG00000162817 |  |
|  | PLXNB2 |  |
|  | CLIC2 |  |
|  | HEATR5A |  |
|  | TMEM154 |  |
|  | GNAI3 |  |
|  | STAM2 |  |
|  | TFRC |  |
|  | EPB41L3 |  |
|  | GSAP |  |
|  | RAB32 |  |
|  | C20orf194 |  |
|  | ETFA |  |
|  | GINM1 |  |
|  | WDR26 |  |
|  | STAT2 |  |
|  | RYBP |  |
|  | MIR628 |  |
|  | NMI |  |
|  | FAM21D |  |
|  | PSMA4 |  |
|  | NABP1 |  |
|  | MAP3K8 |  |
|  | BCL11A |  |
|  | SPAST |  |
|  | ATP6V0D1 |  |
|  | DCP2 |  |
|  | ARRB2 |  |
|  | NKG7 |  |
|  | TMEM55A |  |
|  | FIG4 |  |
|  | USP28 |  |
|  | STOM |  |
|  | RNFT1 |  |
|  | MIR186 |  |
|  | RAP1GDS1 |  |
|  | MTCH2 |  |
|  | CRTAP |  |
|  | SLC25A24 |  |
|  | CTSB |  |
|  | DICER1 |  |
|  | ACOT9 |  |
|  | SLFN12 |  |
|  | HIST1H2BK |  |
|  | HSPA7 |  |
|  | RAB27A |  |
|  | CPT1A |  |
|  | RNF19B |  |
|  | NHLRC2 |  |
|  | ATP6V0B |  |
|  | LOC100505573 |  |
|  | CDC42-IT1 |  |
|  | NAMPT |  |
|  | RAP1B |  |
|  | UTRN |  |
|  | SCFD1 |  |
|  | GLUL |  |
|  | VAMP7 |  |
|  | PRDX3 |  |
|  | IL17RA |  |
|  | CFP |  |
|  | LOC100216546 |  |
|  | UBR2 |  |
|  | MOSPD2 |  |
|  | ZDHHC7 |  |
|  | ZAK |  |
|  | DNAJC13 |  |
|  | AP3B1 |  |
|  | SFT2D2 |  |
|  | RNA5SP149 |  |
|  | SNX5 |  |
|  | STX12 |  |
|  | HIST2H2AA4 |  |
|  | FRAT2 |  |
|  | ADRB2 |  |
|  | OTTHUMG00000161654 |  |
|  | WIPI1 |  |
|  | TRIM25 |  |
|  | PIGB |  |
|  | MTMR10 |  |
|  | GPCPD1 |  |
|  | PIK3C2A |  |
|  | TATDN3 |  |
|  | DNAJC10 |  |
|  | MTSS1 |  |
|  | CPED1 |  |
|  | SBF2 |  |
|  | YWHAG |  |
|  | OTTHUMG00000149333 |  |
|  | FCHSD2 |  |
|  | HIST1H4H |  |
|  | UNC93B1 |  |
|  | GNG5 |  |
|  | RAB1A |  |
|  | JUNB |  |
|  | PP12708 |  |
|  | SPG21 |  |
|  | C12orf5 |  |
|  | YWHAE |  |
|  | MAN2B1 |  |
|  | STRAP |  |
|  | WDR11 |  |
|  | STEAP4 |  |
|  | ATP1B3 |  |
|  | CFD |  |
|  | KCNE3 |  |
|  | SLC8A1 |  |
|  | ASAP1 |  |
|  | COL4A3BP |  |
|  | MYD88 |  |
|  | RNA5S17 |  |
|  | AKAP10 |  |
|  | RAB12 |  |
|  | AOAH-IT1 |  |
|  | SNX2 |  |
|  | UVRAG |  |
|  | TAOK1 |  |
|  | UBE2K |  |
|  | SAMD9L |  |
|  | NHLRC3 |  |
|  | CPEB4 |  |
|  | FTH1 |  |
|  | NLK |  |
|  | CEPT1 |  |
|  | IL10RB |  |
|  | GPX1 |  |
|  | ARHGAP17 |  |
|  | ITCH |  |
|  | KLRG1 |  |
|  | ATP6AP2 |  |
|  | GBP5 |  |
|  | LINC00641 |  |
|  | RNA5SP202 |  |
|  | PPIP5K2 |  |
|  | ARL5A |  |
|  | TLN1 |  |
|  | CASP4 |  |
|  | FEZ2 |  |
|  | RFWD2 |  |
|  | RRN3P2 |  |
|  | SNORA28 |  |
|  | NCOA4 |  |
|  | BLZF1 |  |
|  | ARL8B |  |
|  | MBD2 |  |
|  | SNX14 |  |
|  | PSENEN |  |
|  | OTTHUMG00000132571 |  |
|  | ARHGAP21 |  |
|  | HIPK3 |  |
|  | KIAA1468 |  |
|  | CD300LF |  |
|  | FLJ44635 |  |
|  | RAB11A |  |
|  | RBBP8 |  |
|  | TBC1D8 |  |
|  | HIST1H4J |  |
|  | TGFBR1 |  |
|  | CTNND1 |  |
|  | WNK1 |  |
|  | IFIH1 |  |
|  | RIN2 |  |
|  | MGST2 |  |
|  | PTPN22 |  |
|  | HSBP1 |  |
|  | CTSD |  |
|  | ROCK2 |  |
|  | CHML |  |
|  | CHD1 |  |
|  | SLC43A3 |  |
|  | DOPEY2 |  |
|  | IRF1 |  |
|  | FAM126A |  |
|  | PRELID1 |  |
|  | KIF5B |  |
|  | KIAA0513 |  |
|  | PRKAR1A |  |
|  | GDAP2 |  |
|  | ABHD5 |  |
|  | OTTHUMG00000162841 |  |
|  | IQGAP1 |  |
|  | C10orf76 |  |
|  | EMP3 |  |
|  | TGFB1 |  |
|  | ABI3 |  |
|  | OTTHUMG00000153694 |  |
|  | MIR3690 |  |
|  | TPP1 |  |
|  | OS9 |  |
|  | GLA |  |
|  | LOC284837 |  |
|  | ACTR1A |  |
|  | OSTF1 |  |
|  | RNA5SP199 |  |
|  | UBA6 |  |
|  | RNU4ATAC |  |
|  | TRIM38 |  |
|  | AMD1 |  |
|  | CLIC1 |  |
|  | RNF213 |  |
|  | SNORA71A |  |
|  | C18orf8 |  |
|  | PRKCB |  |
|  | ID2B |  |
|  | ADPGK |  |
|  | ATXN1 |  |
|  | ACTR2 |  |
|  | OTTHUMG00000160994 |  |
|  | CAST |  |
|  | BROX |  |
|  | ATP6V1C1 |  |
|  | DENND1A |  |
|  | PKN2 |  |
|  | ZNF267 |  |
|  | FAM49B |  |
|  | MPP1 |  |
|  | FLJ10038 |  |
|  | SERPINB8 |  |
|  | SASH1 |  |
|  | WDFY4 |  |
|  | ID2 |  |
|  | PGGT1B |  |
|  | OTTHUMG00000040465 |  |
|  | NRD1 |  |
|  | GAB2 |  |
|  | FTH1P4 |  |
|  | TKT |  |
|  | ATAD2B |  |
|  | FAM45A |  |
|  | RAP2B |  |
|  | SLC36A4 |  |
|  | GDI2 |  |
|  | FBXO11 |  |
|  | LINC00152 |  |
|  | VIM-AS1 |  |
|  | NT5C3A |  |
|  | HIST1H2AE |  |
|  | RAC1 |  |
|  | MIR3136 |  |
|  | SFPQ |  |
|  | RIT1 |  |
|  | PLCB2 |  |
|  | XRN2 |  |
|  | EMILIN2 |  |
|  | LAPTM4A |  |
|  | CYSLTR2 |  |
|  | SAT2 |  |
|  | CFLAR |  |
|  | OTTHUMG00000132657 |  |
|  | FAM91A1 |  |
|  | ANXA2P1 |  |
|  | VAMP3 |  |
|  | OTTHUMG00000014906 |  |
|  | ZMPSTE24 |  |
|  | DMXL1 |  |
|  | IFNAR2 |  |
|  | ITPR2 |  |
|  | DNAJA1 |  |
|  | TOP1 |  |
|  | RNA5SP33 |  |
|  | JHDM1D |  |
|  | CSGALNACT2 |  |
|  | ZNRF2 |  |
|  | GALNT1 |  |
|  | THEMIS2 |  |
|  | DOCK2 |  |
|  | BAZ1A |  |
|  | CD97 |  |
|  | NIPSNAP3A |  |
|  | TMEM65 |  |
|  | PPP3R1 |  |
|  | FAM120A |  |
|  | SCARNA8 |  |
|  | GPR65 |  |
|  | CAPNS1 |  |
|  | MYO1E |  |
|  | KCTD5 |  |
|  | HADHB |  |
|  | AGFG1 |  |
|  | RASGRP4 |  |
|  | SAMSN1 |  |
|  | FNDC3B |  |
|  | EOMES |  |
|  | NHSL2 |  |
|  | TMEM50A |  |
|  | RNU6-82P |  |
|  | PHTF2 |  |
|  | VPS36 |  |
|  | IL10RA |  |
|  | NFE2L3 |  |
|  | WDR44 |  |
|  | PIK3CB |  |
|  | SERPINF1 |  |
|  | VPS13C |  |
|  | MICAL2 |  |
|  | LOC285972 |  |
|  | CCDC47 |  |
|  | PPP3CB |  |
|  | PTGER4 |  |
|  | ACSL1 |  |
|  | ARHGAP31 |  |
|  | NEDD9 |  |
|  | PELI2 |  |
|  | IFI6 |  |
|  | CAPZA1 |  |
|  | ITFG1 |  |
|  | PRR11 |  |
|  | INTS7 |  |
|  | NPTN |  |
|  | LRMP |  |
|  | NOP10 |  |
|  | STXBP3 |  |
|  | EFR3A |  |
|  | CYTH4 |  |
|  | VCAN-AS1 |  |
|  | CYFIP1 |  |
|  | PAPD4 |  |
|  | LINC00341 |  |
|  | ACAA2 |  |
|  | CYBA |  |
|  | ATP5C1 |  |
|  | SGK1 |  |
|  | PSMA3 |  |
|  | STX8 |  |
|  | PGK1 |  |
|  | WDFY2 |  |
|  | MERTK |  |
|  | PREX1 |  |
|  | GANC |  |
|  | ALDH1A1 |  |
|  | KAT2B |  |
|  | RPN1 |  |
|  | FRY |  |
|  | CTSA |  |
|  | SUPT4H1 |  |
|  | RCN1P2 |  |
|  | TRAPPC11 |  |
|  | GLB1 |  |
|  | EPS15 |  |
|  | SERPINB1 |  |
|  | SNORA2A |  |
|  | PPM1D |  |
|  | SHOC2 |  |
|  | OTTHUMG00000066284 |  |
|  | SLC35F5 |  |
|  | TUBA1A |  |
|  | KIAA1432 |  |
|  | ULK2 |  |
|  | CD44 |  |
|  | AFTPH |  |
|  | BMPR2 |  |
|  | SNORA53 |  |
|  | HIST1H3B |  |
|  | SNX30 |  |
|  | MEMO1P1 |  |
|  | HIST1H1B |  |
|  | PSME4 |  |
|  | PXK |  |
|  | DNASE1L3 |  |
|  | ELF4 |  |
|  | PTPN9 |  |
|  | OTTHUMG00000155528 |  |
|  | FEM1C |  |
|  | CD244 |  |
|  | SIGLEC10 |  |
|  | UBXN2B |  |
|  | TRIP12 |  |
|  | NLRP3 |  |
|  | MRPS35 |  |
|  | TMEM179B |  |
|  | SIRPB2 |  |
|  | ARPC2 |  |
|  | LOC100190986 |  |
|  | ATP2A2 |  |
|  | SETX |  |
|  | KIN |  |
|  | POLK |  |
|  | NSF |  |
|  | VCPIP1 |  |
|  | DCAF13P3 |  |
|  | ZNF124 |  |
|  | RANBP9 |  |
|  | CRK |  |
|  | BLOC1S2 |  |
|  | SLC9A9 |  |
|  | ACOX1 |  |
|  | PTGDR |  |
|  | KLHL5 |  |
|  | KIAA0368 |  |
|  | MIR2110 |  |
|  | FGD6 |  |
|  | MED13L |  |
|  | MIR573 |  |
|  | FAM160B1 |  |
|  | CD164 |  |
|  | H2AFY |  |
|  | FUCA2 |  |
|  | YPEL5 |  |
|  | NFIL3 |  |
|  | HMGCR |  |
|  | PDP1 |  |
|  | NPL |  |
|  | SEC24B |  |
|  | KIAA0226L |  |
|  | RNA5S9 |  |
|  | ATP11A |  |
|  | TP53INP1 |  |
|  | HELZ |  |
|  | USP8 |  |
|  | DIAPH1 |  |
|  | KDELR2 |  |
|  | RNY4 |  |
|  | CSNK1A1 |  |
|  | TRIB1 |  |
|  | SPI1 |  |
|  | TRGV2 |  |
|  | KIAA0100 |  |
|  | ATP5E |  |
|  | LOC729603 |  |
|  | HSPA5 |  |
|  | ADAM9 |  |
|  | SNX4 |  |
|  | HIST1H2BH |  |
|  | DUSP22 |  |
|  | SLC35A1 |  |
|  | KIF13B |  |
|  | MAPK1 |  |
|  | IREB2 |  |
|  | TAOK3 |  |
|  | QSER1 |  |
|  | GLT1D1 |  |
|  | DCLRE1C |  |
|  | SH3GLB1 |  |
|  | HGF |  |
|  | PPP1R12A |  |
|  | MAP3K3 |  |
|  | GIMAP8 |  |
|  | MON2 |  |
|  | ANTXR2 |  |
|  | ACTN4 |  |
|  | FLNA |  |
|  | DIRC2 |  |
|  | TSPAN13 |  |
|  | FAM102B |  |
|  | DNAJC1 |  |
|  | SNX3 |  |
|  | SLC9A7 |  |
|  | GNLY |  |
|  | FLVCR2 |  |
|  | ANKRD44-IT1 |  |
|  | CBL |  |
|  | TMX1 |  |
|  | PLEKHO1 |  |
|  | NT5C2 |  |
|  | LPCAT3 |  |
|  | HNRNPLL |  |
|  | S100Z |  |
|  | PAN3 |  |
|  | CANX |  |
|  | SRP54 |  |
|  | GM2A |  |
|  | CR1 |  |
|  | HLA-DOA |  |
|  | RHOU |  |
|  | ANXA1 |  |
|  | SSH2 |  |
|  | SELT |  |
|  | DECR1 |  |
|  | NFKBIZ |  |
|  | PPP2R3C |  |
|  | KLHL8 |  |
|  | PDIA3 |  |
|  | TAS2R19 |  |
|  | SNAP29 |  |
|  | SMIM14 |  |
|  | HSPBAP1 |  |
|  | COPA |  |
|  | BIRC6 |  |
|  | IBTK |  |
|  | APP |  |
|  | CHPT1 |  |
|  | ALG2 |  |
|  | LILRA5 |  |
|  | PANK3 |  |
|  | ATR |  |
|  | IFI16 |  |
|  | PPP3CA |  |
|  | USP12 |  |
|  | MAP4K5 |  |
|  | FAM63B |  |
|  | VPS54 |  |
|  | FUCA1 |  |
|  | TLR5 |  |
|  | IGF2R |  |
|  | VIM |  |
|  | EIF2AK4 |  |
|  | RAB8A |  |
|  | SNORD78 |  |
|  | CAP1 |  |
|  | GLIPR2 |  |
|  | LRRFIP1 |  |
|  | LOC653653 |  |
|  | AKAP9 |  |
|  | CHMP2A |  |
|  | UEVLD |  |
|  | ITGAL |  |
|  | XPR1 |  |
|  | SMC6 |  |
|  | GTF2A2 |  |
|  | LRRFIP2 |  |
|  | HECTD1 |  |
|  | FAM45B |  |
|  | LRRC40 |  |
|  | P4HA1 |  |
|  | TNF |  |
|  | CALU |  |
|  | PSMA6 |  |
|  | EVI2A |  |
|  | ETFDH |  |
|  | USP3 |  |
|  | MIR3975 |  |
|  | SLC4A7 |  |
|  | HSPA4 |  |
|  | KPNB1 |  |
|  | DDX60L |  |
|  | ATG4C |  |
|  | OTTHUMG00000160538 |  |
|  | BICD2 |  |
|  | POMP |  |
|  | CAPN2 |  |
|  | ZUFSP |  |
|  | PPID |  |
|  | MARK3 |  |
|  | CTSO |  |
|  | CNPY3 |  |
|  | ALCAM |  |
|  | MAPK14 |  |
|  | LOC595101 |  |
|  | PTPN6 |  |
|  | OAS1 |  |
|  | MTF1 |  |
|  | EIF2AK2 |  |
|  | HPSE |  |
|  | POLB |  |
|  | HEATR3 |  |
|  | RDX |  |
|  | RAB2A |  |
|  | COMMD9 |  |
|  | CLMN |  |
|  | USP24 |  |
|  | HIST1H3F |  |
|  | ACAP2 |  |
|  | RNU11 |  |
|  | VPS35 |  |
|  | PARP9 |  |
|  | SLC43A2 |  |
|  | RABGAP1L |  |
|  | EIF4A1 |  |
|  | RRAGC |  |
|  | RAB8B |  |
|  | CCDC18 |  |
|  | MILR1 |  |
|  | PTP4A2 |  |
|  | CKLF |  |
|  | FKBP1A |  |
|  | FNIP2 |  |
|  | LMNB1 |  |
|  | SUZ12P1 |  |
|  | NF1P5 |  |
|  | CDC42EP3 |  |
|  | STAT1 |  |
|  | MT2A |  |
|  | DOCK8 |  |
|  | RNA5SP450 |  |
|  | PRKAR2A |  |
|  | ROCK1 |  |
|  | SMPDL3A |  |
|  | UGGT1 |  |
|  | SMG1P1 |  |
|  | UTP6 |  |
|  | GK |  |
|  | ZMIZ1 |  |
|  | CACUL1 |  |
|  | SLC25A40 |  |
|  | NBN |  |
|  | MEF2A |  |
|  | KPNA4 |  |
|  | PRRG4 |  |
|  | SNORA71C |  |
|  | GALC |  |
|  | PCNXL4 |  |
|  | NUPL2 |  |
|  | PIKFYVE |  |
|  | AP4E1 |  |
|  | RASGEF1B |  |
|  | GSK3B |  |
|  | PPP2R5A |  |
|  | FBXL20 |  |
|  | SLC25A6 |  |
|  | C1orf63 |  |
|  | ZDHHC17 |  |
|  | NETO2 |  |
|  | FMNL2 |  |
|  | NSL1 |  |
|  | CCNH |  |
|  | SSR3 |  |
|  | BNIP2 |  |
|  | SGPP1 |  |
|  | RNF169 |  |
|  | CD46 |  |
|  | FAM178A |  |
|  | ERV3-1 |  |
|  | KLF7-IT1 |  |
|  | RUFY3 |  |
|  | ZMYM4 |  |
|  | PSMB3 |  |
|  | PSMD1 |  |
|  | ILK |  |
|  | PLAA |  |
|  | RHOT1 |  |
|  | JKAMP |  |
|  | DUSP10 |  |
|  | MCOLN2 |  |
|  | MDM2 |  |
|  | C6orf62 |  |
|  | NCOA2 |  |
|  | PARP4 |  |
|  | RIPK2 |  |
|  | DUSP5 |  |
|  | NUPL1 |  |
|  | SGK3 |  |
|  | KIAA0232 |  |
|  | PTEN |  |
|  | DNAJC7 |  |
|  | USP48 |  |
|  | PTTG1IP |  |
|  | TRAPPC8 |  |
|  | CCT8 |  |
|  | NFAT5 |  |
|  | SNORA16B |  |
|  | TIMM23 |  |
|  | ADAM10 |  |
|  | TMEM131 |  |
|  | EVI2B |  |
|  | HPS3 |  |
|  | HSD17B11 |  |
|  | SCARNA23 |  |
|  | DLD |  |
|  | HNRNPR |  |
|  | HIST1H1E |  |
|  | PHF20L1 |  |
|  | RASSF4 |  |
|  | NUDT3 |  |
|  | C20orf24 |  |
|  | NIPBL |  |
|  | TBXAS1 |  |
|  | TIMM17A |  |
|  | C16orf70 |  |
|  | ANO6 |  |
|  | RSF1-IT2 |  |
|  | SEC24A |  |
|  | RHOA |  |
|  | GBP1 |  |
|  | C1orf27 |  |
|  | FAR2 |  |
|  | SOS1 |  |
|  | RBPJ |  |
|  | FBXL5 |  |
|  | COLGALT1 |  |
|  | SLC7A5P2 |  |
|  | CCNT2 |  |
|  | PAIP2 |  |
|  | GSR |  |
|  | TNFRSF1A |  |
|  | CMIP |  |
|  | TIMMDC1 |  |
|  | TBC1D1 |  |
|  | TMEM127 |  |
|  | ZC2HC1A |  |
|  | STRN3 |  |
|  | SLC25A16 |  |
|  | RAB4A |  |
|  | SIRPD |  |
|  | SLC41A2 |  |
|  | ITGB2 |  |
|  | MLX |  |
|  | CCZ1 |  |
|  | MBTD1 |  |
|  | ATP13A3 |  |
|  | RC3H2 |  |
|  | CASP3 |  |
|  | CHUK |  |
|  | GBP3 |  |
|  | FTH1P3 |  |
|  | RRN3P3 |  |
|  | EMC7 |  |
|  | SPIB |  |
|  | UGGT2 |  |
|  | CXCR6 |  |
|  | EXOC1 |  |
|  | DDX21 |  |
|  | DDHD1 |  |
|  | MED13 |  |
|  | HIST1H2AI |  |
|  | LMBRD1 |  |
|  | RSU1 |  |
|  | TMEM181 |  |
|  | HOTAIRM1 |  |
|  | ANP32A |  |
|  | LOC100506060 |  |
|  | RAB5A |  |
|  | PPIB |  |
|  | NDUFA6 |  |
|  | TRGV8 |  |
|  | DCAF12 |  |
|  | SPCS3 |  |
|  | NBEAL2 |  |
|  | GPR155 |  |
|  | SNORA21 |  |
|  | TOR1AIP1 |  |
|  | STT3A |  |
|  | NPEPPS |  |
|  | GATAD1 |  |
|  | ZNF326 |  |
|  | PSMG2 |  |
|  | GNPAT |  |
|  | GNAQ |  |
|  | RAP1GAP2 |  |
|  | NCSTN |  |
|  | EDEM2 |  |
|  | SLC39A6 |  |
|  | ECHDC1 |  |
|  | SEC24D |  |
|  | MXD1 |  |
|  | EPSTI1 |  |
|  | NAA50 |  |
|  | LCP1 |  |
|  | HCLS1 |  |
|  | PPP2R5E |  |
|  | GDE1 |  |
|  | CYCS |  |
|  | SMG1 |  |
|  | TRA2B |  |
|  | C12orf75 |  |
|  | RAB5C |  |
|  | FAM188A |  |
|  | PFKFB2 |  |
|  | RNA5SP150 |  |
|  | RNU4-1 |  |
|  | TRPM7 |  |
|  | YIPF4 |  |
|  | AKAP13 |  |
|  | SNORD83A |  |
|  | LACC1 |  |
|  | RNA5SP68 |  |
|  | LGALS9 |  |
|  | ZFAND6 |  |
|  | NCOA3 |  |
|  | PID1 |  |
|  | ARRDC3 |  |
|  | FANCL |  |
|  | BRWD3 |  |
|  | TBL1XR1 |  |
|  | ASCC3 |  |
|  | PSMC6 |  |
|  | TBC1D23 |  |
|  | ZNF562 |  |
|  | TRIP4 |  |
|  | GOLGA4 |  |
|  | CPSF3 |  |
|  | ARFIP1 |  |
|  | LCORL |  |
|  | NIPAL2 |  |
|  | SIPA1L1 |  |
|  | MBNL3 |  |
|  | YWHAB |  |
|  | CREBBP |  |
|  | RC3H1 |  |
|  | RPE |  |
|  | LOC440354 |  |
|  | PLEKHM1P |  |
|  | SAMD8 |  |
|  | MR1 |  |
|  | LPP |  |
|  | RAB7A |  |
|  | HIST2H2BC |  |
|  | PDLIM5 |  |
|  | GNAI2 |  |
|  | HERC4 |  |
|  | DHX29 |  |
|  | TM7SF3 |  |
|  | RNF111 |  |
|  | RMDN1 |  |
|  | ADIPOR1 |  |
|  | SCYL2 |  |
|  | C12orf4 |  |
|  | TRPS1 |  |
|  | GAB1 |  |
|  | IL18R1 |  |
|  | CDK19 |  |
|  | ARHGEF12 |  |
|  | DNAJB14 |  |
|  | ANGEL2 |  |
|  | MCUR1 |  |
|  | ARPC4 |  |
|  | ATP2C1 |  |
|  | SENP6 |  |
|  | IDH1 |  |
|  | ALOX5AP |  |
|  | SNORD56B |  |
|  | TBC1D5 |  |
|  | OTUD6B |  |
|  | FLJ31306 |  |
|  | MSN |  |
|  | SCAF11 |  |
|  | OTTHUMG00000015263 |  |
|  | ANXA4 |  |
|  | CHMP4B |  |
|  | HPRT1 |  |
|  | VPS41 |  |
|  | PPP2CA |  |
|  | RSU1P1 |  |
|  | ADCY7 |  |
|  | DYNC1LI1 |  |
|  | SS18 |  |
|  | RNU7-38P |  |
|  | VASP |  |
|  | SNORD12 |  |
|  | ATP6V0E1 |  |
|  | ARHGDIB |  |
|  | GNPTAB |  |
|  | GBP2 |  |
|  | CD33 |  |
|  | OTTHUMG00000017742 |  |
|  | DAB2 |  |
|  | DCTN4 |  |
|  | TMF1 |  |
|  | HIATL2 |  |
|  | REREP3 |  |
|  | RELL1 |  |
|  | IFNAR1 |  |
|  | MIRLET7G |  |
|  | PLCB1 |  |
|  | NOTCH2NL |  |
|  | YTHDC2 |  |
|  | SNX13 |  |
|  | CPEB2 |  |
|  | CPSF2 |  |
|  | SOAT1 |  |
|  | CSF2RB |  |
|  | IRAK4 |  |
|  | RNA5SP429 |  |
|  | LYPLAL1 |  |
|  | DENND3 |  |
|  | ATG12 |  |
|  | BTBD1 |  |
|  | SNORD98 |  |
|  | METTL21D |  |
|  | C17orf85 |  |
|  | ITGA4 |  |
|  | SEL1L |  |
|  | TRAPPC6B |  |
|  | MIR1284 |  |
|  | TIPRL |  |
|  | DCTN2 |  |
|  | TRIT1 |  |
|  | ITCH-IT1 |  |
|  | ANAPC5 |  |
|  | LOC100129550 |  |
|  | CHMP5 |  |
|  | GAS7 |  |
|  | UCP2 |  |
|  | ARPC5 |  |
|  | N4BP2L1 |  |
|  | EGLN1 |  |
|  | FAM135A |  |
|  | FRAT1 |  |
|  | MASTL |  |
|  | CUL3 |  |
|  | PLEKHO2 |  |
|  | RAB24 |  |
|  | RNA5SP481 |  |
|  | ZMYM6NB |  |
|  | YTHDF3 |  |
|  | FMNL1 |  |
|  | PLAUR |  |
|  | CALR |  |
|  | MGEA5 |  |
|  | KRIT1 |  |
|  | AZI2 |  |
|  | VPS29 |  |
|  | TMEM167A |  |
|  | SMS |  |
|  | GLUD1 |  |
|  | ACTR3 |  |
|  | COPB1 |  |
|  | AGO1 |  |
|  | GPBP1L1 |  |
|  | TRIO |  |
|  | MAP3K1 |  |
|  | SRD5A3 |  |
|  | ENO1 |  |
|  | TAX1BP1 |  |
|  | XPO7 |  |
|  | TACC1 |  |
|  | VPS25 |  |
|  | NPIPB11 |  |
|  | SOS2 |  |
|  | NFE2L2 |  |
|  | SCARNA3 |  |
|  | NLN |  |
|  | NEMF |  |
|  | MS4A4A |  |
|  | PMAIP1 |  |
|  | ADSS |  |
|  | MIR454 |  |
|  | SLC25A5 |  |
|  | IFI44 |  |
|  | BRI3BP |  |
|  | CDC123 |  |
|  | TXNDC12 |  |
|  | HIGD1A |  |
|  | SSR1 |  |
|  | OTTHUMG00000155228 |  |
|  | OTTHUMG00000155862 |  |
|  | POLR3C |  |
|  | DHX15 |  |
|  | ZNF217 |  |
|  | GTF2F2 |  |
|  | RB1CC1 |  |
|  | FAM32A |  |
|  | ETF1 |  |
|  | APOBEC3C |  |
|  | PSMD12 |  |
|  | PSME2 |  |
|  | RPS6KC1 |  |
|  | RPN2 |  |
|  | TAGLN2 |  |
|  | LOC646329 |  |
|  | SCRN1 |  |
|  | LARP4 |  |
|  | KIAA1109 |  |
|  | TTC33 |  |
|  | CLIP1 |  |
|  | ARF4 |  |
|  | MPC1 |  |
|  | PRPF40A |  |
|  | SEC61A1 |  |
|  | MIR3662 |  |
|  | PAXBP1 |  |
|  | OXSR1 |  |
|  | DMTF1 |  |
|  | VPS45 |  |
|  | TEP1 |  |
|  | ANKRD32 |  |
|  | RAPGEF2 |  |
|  | PFN1 |  |
|  | PANK2 |  |
|  | COX6B1 |  |
|  | FLJ37453 |  |
|  | KIF13A |  |
|  | ZMYM2 |  |
|  | CSNK2A2 |  |
|  | OTTHUMG00000152880 |  |
|  | IFRD1 |  |
|  | GPD2 |  |
|  | BST2 |  |
|  | ITGAV |  |
|  | XPO1 |  |
|  | SNORA2B |  |
|  | CDC42 |  |
|  | ZFP36L2 |  |
|  | APC |  |
|  | SUGT1 |  |
|  | DPP8 |  |
|  | PITPNA |  |
|  | SLC31A2 |  |
|  | SLC30A7 |  |
|  | VSIG4 |  |
|  | CLINT1 |  |
|  | CCT5 |  |
|  | RNF115 |  |
|  | SLC24A4 |  |
|  | S100A11 |  |
|  | PRKX |  |
|  | TBC1D12 |  |
|  | GNB1 |  |
|  | RNASET2 |  |
|  | REEP3 |  |
|  | ARHGAP11B |  |
|  | ADNP |  |
|  | KLHL2 |  |
|  | MIR632 |  |
|  | RHEB |  |
|  | USP33 |  |
|  | NUSAP1 |  |
|  | CLCN5 |  |
|  | ELF2 |  |
|  | KIAA0825 |  |
|  | IMMT |  |
|  | KIDINS220 |  |
|  | PPP1R21 |  |
|  | RAD23B |  |
|  | RNU7-45P |  |
|  | LRRC58 |  |
|  | GBAS |  |
|  | ITSN2 |  |
|  | ZNF791 |  |
|  | TMEM168 |  |
|  | EXOC8 |  |
|  | RAP1A |  |
|  | MYSM1 |  |
|  | NUP54 |  |
|  | BOD1L1 |  |
|  | RABEP1 |  |
|  | SLC25A30 |  |
|  | CXorf23 |  |
|  | GTF2IP1 |  |
|  | RECQL |  |
|  | C6orf211 |  |
|  | PAPSS1 |  |
|  | XRN1 |  |
|  | PCCA |  |
|  | TAF1B |  |
|  | EIF3A |  |
|  | CEP152 |  |
|  | ADIPOR2 |  |
|  | MAP3K2 |  |
|  | DYNC1H1 |  |
|  | KIAA0196 |  |
|  | SDCCAG8 |  |
|  | EXT1 |  |
|  | SETD7 |  |
|  | RNA5SP325 |  |
|  | SPAG9 |  |
|  | LOC100505794 |  |
|  | SMAD5 |  |
|  | STARD7 |  |
|  | SLC33A1 |  |
|  | ATL3 |  |
|  | C15orf39 |  |
|  | HPS5 |  |
|  | ANKRD44 |  |
|  | WDR47 |  |
|  | MTMR14 |  |
|  | VPS26A |  |
|  | OTTHUMG00000037473 |  |
|  | FOPNL |  |
|  | PRDX1 |  |
|  | HSD17B4 |  |
|  | ANAPC4 |  |
|  | ARFGEF1 |  |
|  | WASF2 |  |
|  | SMNDC1 |  |
|  | ATP5B |  |
|  | DDX52 |  |
|  | FNIP1 |  |
|  | RASA1 |  |
|  | USO1 |  |
|  | TTC13 |  |
|  | ZNF655 |  |
|  | EXOC6 |  |
|  | SNORA70B |  |
|  | CLEC12B |  |
|  | SEPT11 |  |
|  | PCMT1 |  |
|  | YIPF1 |  |
|  | USF1 |  |
|  | ERN1 |  |
|  | SQRDL |  |
|  | YWHAQ |  |
|  | FAM111A |  |
|  | TMEM106A |  |
|  | NTAN1 |  |
|  | PHACTR2 |  |
|  | ARNT |  |
|  | SLC11A1 |  |
|  | TRAFD1 |  |
|  | SMC4 |  |
|  | EIF1B |  |
|  | GABARAPL2 |  |
|  | NRAS |  |
|  | CD300LB |  |
|  | ATP5F1 |  |
|  | MFSD1 |  |
|  | MKNK1 |  |
|  | CERS5 |  |
|  | TSG101 |  |
|  | S100A10 |  |
|  | PRKAG1 |  |
|  | RSPH3 |  |
|  | MAPKAPK3 |  |
|  | SMAD2 |  |
|  | PIGF |  |
|  | ETNK1 |  |
|  | TMED7-TICAM2 |  |
|  | RNY3P2 |  |
|  | NAPG |  |
|  | IER5 |  |
|  | EYA3 |  |
|  | ZFR |  |
|  | ACBD5 |  |
|  | LAPTM5 |  |
|  | LYSMD3 |  |
|  | MAPKAPK2 |  |
|  | NCOR1 |  |
|  | CCNJ |  |
|  | RRN3P1 |  |
|  | RAF1 |  |
|  | ABHD3 |  |
|  | GTF2H1 |  |
|  | IQSEC1 |  |
|  | UTP3 |  |
|  | C10orf54 |  |
|  | FTX |  |
|  | LINC00260 |  |
|  | CEP192 |  |
|  | FAM133B |  |
|  | PLAGL2 |  |
|  | NEK3 |  |
|  | TRA2A |  |
|  | LOC440434 |  |
|  | HSPB11 |  |
|  | PTPN2 |  |
|  | TMED5 |  |
|  | TMOD2 |  |
|  | ZFC3H1 |  |
|  | EAPP |  |
|  | ZZEF1 |  |
|  | NID1 |  |
|  | MIR505 |  |
|  | SP110 |  |
|  | DHX57 |  |
|  | LYPLA1 |  |
|  | MYO1G |  |
|  | SYNE1 |  |
|  | PURB |  |
|  | DCAF6 |  |
|  | INPP5D |  |
|  | BRCC3 |  |
|  | SLC38A9 |  |
|  | TRPC4AP |  |
|  | TMEM184C |  |
|  | NDUFAB1 |  |
|  | BHLHE40 |  |
|  | ATG7 |  |
|  | OAZ2 |  |
|  | NCBP1 |  |
|  | CKAP5 |  |
|  | PSMD13 |  |
|  | ABCD3 |  |
|  | MYBL1 |  |
|  | HCCS |  |
|  | ACTB |  |
|  | HIST1H2BM |  |
|  | OSTC |  |
|  | SLC25A3 |  |
|  | SNRPB2 |  |
|  | IDE |  |
|  | BRK1 |  |
|  | KRCC1 |  |
|  | HEATR5B |  |
|  | ACTG1 |  |
|  | CD53 |  |
|  | MYO9B |  |
|  | PSMA1 |  |
|  | KLC1 |  |
|  | C3orf38 |  |
|  | ZDHHC20 |  |
|  | TDRD7 |  |
|  | OTTHUMG00000157039 |  |
|  | FAM105B |  |
|  | SCARNA16 |  |
|  | VAPA |  |
|  | KIAA0247 |  |
|  | SLC37A2 |  |
|  | COPB2 |  |
|  | HADHA |  |
|  | SETDB1 |  |
|  | PIK3C3 |  |
|  | USP46 |  |
|  | ARAP1 |  |
|  | KDM4A |  |
|  | AHNAK |  |
|  | LSM12 |  |
|  | ARID1A |  |
|  | SCAMP1 |  |
|  | LOC606724 |  |
|  | TIAL1 |  |
|  | MORC3 |  |
|  | PARL |  |
|  | RPS6KA1 |  |
|  | LRRC57 |  |
|  | KCTD20 |  |
|  | PNPLA8 |  |
|  | GOLGA5 |  |
|  | REPS2 |  |
|  | RNF114 |  |
|  | NEDD1 |  |
|  | TTC21B |  |
|  | NCF4 |  |
|  | KCMF1 |  |
|  | CMAS |  |
|  | MAFB |  |
|  | CERS6 |  |
|  | WHAMMP1 |  |
|  | ZMYM5 |  |
|  | ATMIN |  |
|  | LILRB3 |  |
|  | TPM3 |  |
|  | YWHAH |  |
|  | PPIL4 |  |
|  | ZNF652 |  |
|  | MIR1307 |  |
|  | ACBD3 |  |
|  | SRD5A1 |  |
|  | COX6A1 |  |
|  | VPS8 |  |
|  | GADD45B |  |
|  | PAFAH1B1 |  |
|  | CIR1 |  |
|  | SEC23IP |  |
|  | PPFIA1 |  |
|  | RNF10 |  |
|  | DOCK11 |  |
|  | NPIPL3 |  |
|  | CCT6A |  |
|  | SLMAP |  |
|  | MIR7-1 |  |
|  | RUFY2 |  |
|  | OSBPL1A |  |
|  | PHIP |  |
|  | SLC35A5 |  |
|  | BPTF |  |
|  | EED |  |
|  | UBA3 |  |
|  | NARS |  |
|  | IGFBP7 |  |
|  | STAT6 |  |
|  | C2CD5 |  |
|  | LINC00843 |  |
|  | HIST1H3A |  |
|  | FOSL2 |  |
|  | EFTUD2 |  |
|  | TM9SF4 |  |
|  | PBRM1 |  |
|  | GBP4 |  |
|  | CITED2 |  |
|  | GK5 |  |
|  | RNF146 |  |
|  | FAM118B |  |
|  | CHCHD1 |  |
|  | RNA5SP298 |  |
|  | NIPA2 |  |
|  | VEZF1 |  |
|  | TOR1AIP2 |  |
|  | PTBP2 |  |
|  | RNA5SP226 |  |
|  | PKM |  |
|  | SGPL1 |  |
|  | CHSY1 |  |
|  | NOMO2 |  |
|  | MGME1 |  |
|  | ABI1 |  |
|  | NOMO3 |  |
|  | PHF21A |  |
|  | PREP |  |
|  | MIR15B |  |
|  | EIF2B2 |  |
|  | AP3M1 |  |
|  | SLC1A5 |  |
|  | MIOS |  |
|  | BLOC1S6 |  |
|  | NDUFAF1 |  |
|  | GPR82 |  |
|  | AP1G1 |  |
|  | NIN |  |
|  | TTC17 |  |
|  | MIR601 |  |
|  | CKAP2 |  |
|  | DCAF7 |  |
|  | MTERFD1 |  |
|  | IQCB1 |  |
|  | SFT2D1 |  |
|  | YBX1 |  |
|  | SLC25A13 |  |
|  | GTF2A1 |  |
|  | BAG4 |  |
|  | DDX46 |  |
|  | RNA5SP399 |  |
|  | ZNF330 |  |
|  | SRSF9 |  |
|  | TNKS2 |  |
|  | GRB2 |  |
|  | GAPDH |  |
|  | XYLT1 |  |
|  | JMJD1C |  |
|  | RPF1 |  |
|  | RNA5SP19 |  |
|  | TRIM33 |  |
|  | PAIP1 |  |
|  | PQLC3 |  |
|  | RNU1-1 |  |
|  | PSMD8 |  |
|  | CMC1 |  |
|  | SON |  |
|  | FOXN2 |  |
|  | SUMO4 |  |
|  | SNRPG |  |
|  | KBTBD8 |  |
|  | LTN1 |  |
|  | SCARNA22 |  |
|  | PPTC7 |  |
|  | SERINC3 |  |
|  | DNM2 |  |
|  | OTTHUMG00000017937 |  |
|  | SREK1 |  |
|  | SHKBP1 |  |
|  | PEPD |  |
|  | SLC6A6 |  |
|  | STAG3L4 |  |
|  | ARMC8 |  |
|  | CUX1 |  |
|  | STAM |  |
|  | SNX1 |  |
|  | SLC2A3 |  |
|  | ATP2B4 |  |
|  | MIR4677 |  |
|  | FAM53B |  |
|  | PRR13 |  |
|  | ATP6V1F |  |
|  | PIP4K2A |  |
|  | MSH3 |  |
|  | ATG5 |  |
|  | C21orf91 |  |
|  | C1orf43 |  |
|  | U2AF1 |  |
|  | CDC40 |  |
|  | CXorf38 |  |
|  | HTT |  |
|  | POLR2A |  |
|  | MDM1 |  |
|  | STX3 |  |
|  | STX6 |  |
|  | MED14 |  |
|  | OTTHUMG00000154698 |  |
|  | CLASP2 |  |
|  | TMEM59 |  |
|  | DENND4C |  |
|  | WSB2 |  |
|  | GLRX3 |  |
|  | NFKBIA |  |
|  | UBE2E2 |  |
|  | CUL5 |  |
|  | MCMBP |  |
|  | SLTM |  |
|  | ANKRD10 |  |
|  | BECN1 |  |
|  | MIR103A2 |  |
|  | NOMO1 |  |
|  | USP25 |  |
|  | DYNC1I2 |  |
|  | CEP78 |  |
|  | ZC3H7A |  |
|  | LOC613037 |  |
|  | DPM1 |  |
|  | FAM214A |  |
|  | FUT11 |  |
|  | RNU4-2 |  |
|  | PPP1R10 |  |
|  | TUBGCP3 |  |
|  | SEC31A |  |
|  | TMEM30A |  |
|  | FBP1 |  |
|  | RARS |  |
|  | DTX3L |  |
|  | SLC39A9 |  |
|  | RRAS |  |
|  | SOCS4 |  |
|  | HIST2H3D |  |
|  | RNA5SP74 |  |
|  | HEATR6 |  |
|  | SEC23B |  |
|  | ESD |  |
|  | UCHL3 |  |
|  | PCYT1A |  |
|  | PRKACA |  |
|  | KIAA1841 |  |
|  | PLEKHF2 |  |
|  | SPATA6 |  |
|  | ZC3H15 |  |
|  | COQ10B |  |
|  | ATXN10 |  |
|  | HHEX |  |
|  | UBE4B |  |
|  | HMBOX1 |  |
|  | MIB1 |  |
|  | PSMB1 |  |
|  | ALG13 |  |
|  | CCDC82 |  |
|  | EMC3 |  |
|  | ANKRD17 |  |
|  | NGLY1 |  |
|  | OPA1 |  |
|  | LOC146880 |  |
|  | MTMR12 |  |
|  | IDI1 |  |
|  | PARP12 |  |
|  | SYNJ1 |  |
|  | ALDH3A2 |  |
|  | DOPEY1 |  |
|  | SRBD1 |  |
|  | SCAF8 |  |
|  | ITM2B |  |
|  | LIMD1 |  |
|  | SNORA23 |  |
|  | EIF4EBP2 |  |
|  | MLEC |  |
|  | HIST1H4C |  |
|  | UQCRC2 |  |
|  | TTC9C |  |
|  | RNU7-29P |  |
|  | STK10 |  |
|  | IRF2BP2 |  |
|  | FGFR1OP2 |  |
|  | RNVU1-9 |  |
|  | HGSNAT |  |
|  | HIP1 |  |
|  | NPIPB3 |  |
|  | ST3GAL5 |  |
|  | RBM45 |  |
|  | DYNLL1 |  |
|  | TRAPPC1 |  |
|  | PPP1R15B |  |
|  | ERH |  |
|  | CARD8 |  |
|  | ABHD2 |  |
|  | UBE2D3 |  |
|  | NCOA1 |  |
|  | SLC12A6 |  |
|  | MIR4742 |  |
|  | 15-Sep |  |
|  | DHX36 |  |
|  | TXNL1 |  |
|  | PPWD1 |  |
|  | RDH11 |  |
|  | SLC35D2 |  |
|  | ADRBK1 |  |
|  | VPS13B |  |
|  | MOB3A |  |
|  | CDC16 |  |
|  | FAM35A |  |
|  | HIST1H3J |  |
|  | XAF1 |  |
|  | ATF6 |  |
|  | CEP135 |  |
|  | RAB6A |  |
|  | H2AFZ |  |
|  | BNIP3L |  |
|  | IMPAD1 |  |
|  | PRDX6 |  |
|  | CD84 |  |
|  | KPNA3 |  |
|  | PGAM1 |  |
|  | EIF4EBP3 |  |
|  | FAM13B |  |
|  | ZHX1 |  |
|  | OTTHUMG00000159435 |  |
|  | BCL2A1 |  |
|  | USP4 |  |
|  | KIAA0141 |  |
|  | PSMC4 |  |
|  | TMED2 |  |
|  | CHST11 |  |
|  | AGPAT4 |  |
|  | GNL2 |  |
|  | CHMP1B |  |
|  | MRPS10 |  |
|  | MAST3 |  |
|  | PLEKHA2 |  |
|  | SETD2 |  |
|  | OSTM1 |  |
|  | ARL15 |  |
|  | JARID2 |  |
|  | AGPAT9 |  |
|  | ERBB2IP |  |
|  | GAPVD1 |  |
|  | VTI1B |  |
|  | ATP6V1E1 |  |
|  | PTPRS |  |
|  | KIF1B |  |
|  | MAP3K7 |  |
|  | MFN1 |  |
|  | ARPC1B |  |
|  | HIF1A |  |
|  | TARS |  |
|  | NLRP12 |  |
|  | RILPL2 |  |
|  | SDHB |  |
|  | LMAN1 |  |
|  | MIDN |  |
|  | MIR5047 |  |
|  | C4orf29 |  |
|  | RBM18 |  |
|  | BTAF1 |  |
|  | HDAC2 |  |
|  | DR1 |  |
|  | NPIPB5 |  |
|  | TXNDC16 |  |
|  | ZBTB33 |  |
|  | TXNDC5 |  |
|  | AATF |  |
|  | GFM2 |  |
|  | DYM |  |
|  | CEP350 |  |
|  | RAB14 |  |
|  | CREB1 |  |
|  | CNDP2 |  |
|  | SPEN |  |
|  | ERP29 |  |
|  | AVL9 |  |
|  | SLC25A20 |  |
|  | CHKA |  |
|  | ODC1 |  |
|  | GNA13 |  |
|  | RREB1 |  |
|  | PANX1 |  |
|  | TRIM27 |  |
|  | RRM2B |  |
|  | KMT2C |  |
|  | HIST1H2BF |  |
|  | EIF5 |  |
|  | SPRED1 |  |
|  | TMEM2 |  |
|  | CA2 |  |
|  | ATP8A1 |  |
|  | TMEM87A |  |
|  | S100PBP |  |
|  | RCHY1 |  |
|  | TRIQK |  |
|  | TPM4 |  |
|  | RRN3 |  |
|  | ASNA1 |  |
|  | MTMR6 |  |
|  | SYNCRIP |  |
|  | UBE3A |  |
|  | SLIRP |  |
|  | EFCAB14 |  |
|  | MRE11A |  |
|  | HMGCS1 |  |
|  | LOC283922 |  |
|  | CLCC1 |  |
|  | TRAPPC5 |  |
|  | CAB39 |  |
|  | RNPEP |  |
|  | C8orf44 |  |
|  | TMSB4XP8 |  |
|  | CAMKK2 |  |
|  | SRP72 |  |
|  | UFC1 |  |
|  | SNORA11B |  |
|  | GRSF1 |  |
|  | TRAF3 |  |
|  | CNOT6 |  |
|  | LRRC8B |  |
|  | DIP2A-IT1 |  |
|  | DENND5A |  |
|  | TRAF6 |  |
|  | NNT |  |
|  | SNORD10 |  |
|  | THUMPD2 |  |
|  | COMMD3 |  |
|  | GTDC1 |  |
|  | DDX39B |  |
|  | LOC100507577 |  |
|  | TMSB10 |  |
|  | PPA2 |  |
|  | INSIG1 |  |
|  | MIR1278 |  |
|  | THOC2 |  |
|  | RNA5SP155 |  |
|  | NOL10 |  |
|  | ZBTB1 |  |
|  | HERC1 |  |
|  | HSP90B1 |  |
|  | ZNF438 |  |
|  | PPP1R9B |  |
|  | FASTKD1 |  |
|  | SLC35A3 |  |
|  | INTS3 |  |
|  | PSME2P2 |  |
|  | AFF4 |  |
|  | LOC541471 |  |
|  | POLD3 |  |
|  | RMRP |  |
|  | PLP2 |  |
|  | HIST1H2BD |  |
|  | MIR4420 |  |
|  | LOC100506459 |  |
|  | GPR21 |  |
|  | CCSAP |  |
|  | NDUFV2 |  |
|  | SLK |  |
|  | CAPN3 |  |
|  | SEC11C |  |
|  | N4BP2L2 |  |
|  | SLC18B1 |  |
|  | NECAP1 |  |
|  | UPF2 |  |
|  | ITPK1 |  |
|  | UBN2 |  |
|  | UBR5 |  |
|  | YPEL2 |  |
|  | IFIT3 |  |
|  | DENND4A |  |
|  | EDEM3 |  |
|  | MED28 |  |
|  | CBWD2 |  |
|  | PSMD11 |  |
|  | HBP1 |  |
|  | PHF11 |  |
|  | TMX3 |  |
|  | WIPF1 |  |
|  | TMEM39A |  |
|  | MIER1 |  |
|  | GLYR1 |  |
|  | CHM |  |
|  | MPZL2 |  |
|  | IDS |  |
|  | LCP2 |  |
|  | VIPAS39 |  |
|  | RTN4 |  |
|  | PIGN |  |
|  | FNDC3A |  |
|  | SNORA47 |  |
|  | HNRNPA2B1 |  |
|  | REEP5 |  |
|  | RSPRY1 |  |
|  | FBXO7 |  |
|  | LAMP5 |  |
|  | MANEA |  |
|  | GHITM |  |
|  | PNP |  |
|  | SETD5 |  |
|  | APMAP |  |
|  | HMOX1 |  |
|  | GABPB1 |  |
|  | GEN1 |  |
|  | TBRG1 |  |
|  | ROCK1P1 |  |
|  | ADAM19 |  |
|  | DNAJC21 |  |
|  | ARPC1A |  |
|  | EIF4H |  |
|  | OXR1 |  |
|  | EML4 |  |
|  | CHD1L |  |
|  | CHIC2 |  |
|  | PSME1 |  |
|  | PIK3CA |  |
|  | RPS6KA3 |  |
|  | ZCCHC8 |  |
|  | ABR |  |
|  | SNW1 |  |
|  | ZDHHC6 |  |
|  | DNAJA2 |  |
|  | NDUFB2 |  |
|  | CDK13 |  |
|  | RNA5SP242 |  |
|  | SUZ12 |  |
|  | PDZD8 |  |
|  | RNU1-16P |  |
|  | RNASEH2B |  |
|  | ACADM |  |
|  | RTCA |  |
|  | MAP1LC3B |  |
|  | CYBRD1 |  |
|  | TCERG1 |  |
|  | MIR421 |  |
|  | EZH2 |  |
|  | STK38 |  |
|  | NDUFAF7 |  |
|  | PRKD3 |  |
|  | MIR3142 |  |
|  | CNOT1 |  |
|  | PDIA3P |  |
|  | MAPRE1 |  |
|  | LOC100996472 |  |
|  | SMEK1 |  |
|  | RBM5 |  |
|  | SPTSSA |  |
|  | NCK1 |  |
|  | VPS37A |  |
|  | NSMAF |  |
|  | FRYL |  |
|  | ATP7A |  |
|  | OTTHUMG00000018660 |  |
|  | PDCD10 |  |
|  | FAM96A |  |
|  | PTBP3 |  |
|  | RSBN1L |  |
|  | FNTA |  |
|  | PCNA |  |
|  | SMAP2 |  |
|  | POLR2K |  |
|  | RNA5SP352 |  |
|  | SELPLG |  |
|  | PDE8A |  |
|  | ARHGAP1 |  |
|  | KIAA1731 |  |
|  | NBEAL1 |  |
|  | VPS4B |  |
|  | MAP2K1 |  |
|  | ZNF143 |  |
|  | SLCO3A1 |  |
|  | TAB3 |  |
|  | ZNF644 |  |
|  | B4GALT1 |  |
|  | CLNS1A |  |
|  | ABRACL |  |
|  | ARF6 |  |
|  | 7-Mar |  |
|  | HUWE1 |  |
|  | CTR9 |  |
|  | SCFD2 |  |
|  | MAN2A1 |  |
|  | ANAPC7 |  |
|  | CARD6 |  |
|  | IWS1 |  |
|  | SNORA29 |  |
|  | POC1B |  |
|  | TLK2 |  |
|  | ANKRD13A |  |
|  | ACTR10 |  |
|  | SLC30A9 |  |
|  | PSMD10 |  |
|  | RABGGTB |  |
|  | HORMAD1 |  |
|  | SRPK2 |  |
|  | CYB5B |  |
|  | SECTM1 |  |
|  | ANKIB1 |  |
|  | APBB1IP |  |
|  | THAP9-AS1 |  |
|  | WDR75 |  |
|  | ATP6V1D |  |
|  | CCNY |  |
|  | RNU1-11P |  |
|  | C3orf62 |  |
|  | STYXL1 |  |
|  | SAR1B |  |
|  | MGA |  |
|  | TUBA1C |  |
|  | PATL1 |  |
|  | LASP1 |  |
|  | FMR1 |  |
|  | RMND5A |  |
|  | CCDC90B |  |
|  | TMEM167B |  |
|  | CBWD1 |  |
|  | EXOC5 |  |
|  | DDX58 |  |
|  | BZW1 |  |
|  | WDR41 |  |
|  | RALGAPB |  |
|  | DIS3 |  |
|  | OTTHUMG00000037662 |  |
|  | ZNF638 |  |
|  | BIN2 |  |
|  | RNA5SP383 |  |
|  | PRPF4B |  |
|  | SIK3 |  |
|  | YARS |  |
|  | AZIN1 |  |
|  | HEXA |  |
|  | PDPR |  |
|  | TMEM260 |  |
|  | ERO1LB |  |
|  | EIF4G2 |  |
|  | PRPF38B |  |
|  | SRSF7 |  |
|  | SKIV2L2 |  |
|  | CCZ1B |  |
|  | LY75-CD302 |  |
|  | ZCCHC2 |  |
|  | SIRPA |  |
|  | SIGLEC14 |  |
|  | MYL12A |  |
|  | USP7 |  |
|  | OTTHUMG00000152789 |  |
|  | CEP170P1 |  |
|  | AAED1 |  |
|  | ITGA5 |  |
|  | CCDC112 |  |
|  | OGT |  |
|  | VTA1 |  |
|  | STRN |  |
|  | SLAIN2 |  |
|  | VAV1 |  |
|  | RNU1-10P |  |
|  | FBXW2 |  |
|  | KIAA1009 |  |
|  | ARRDC5 |  |
|  | AGPAT5 |  |
|  | EPM2AIP1 |  |
|  | HAUS4 |  |
|  | DPY19L1 |  |
|  | MORF4L1 |  |
|  | DNAJB6 |  |
|  | LZIC |  |
|  | TIA1 |  |
|  | HNRNPAB |  |
|  | AP5M1 |  |
|  | MSANTD4 |  |
|  | AP4B1 |  |
|  | ILF2 |  |
|  | NSD1 |  |
|  | WAC |  |
|  | NEK7 |  |
|  | MICU1 |  |
|  | INO80 |  |
|  | CELF1 |  |
|  | VIMP |  |
|  | GPR107 |  |
|  | PPM1A |  |
|  | RNA5SP77 |  |
|  | RNF7 |  |
|  | PHTF1 |  |
|  | IL6R |  |
|  | CPEB3 |  |
|  | SARNP |  |
|  | NF1 |  |
|  | KIAA0753 |  |
|  | TTC1 |  |
|  | CASP10 |  |
|  | MRC1 |  |
|  | RASSF1 |  |
|  | NCEH1 |  |
|  | ACSL5 |  |
|  | SIK3-IT1 |  |
|  | EBLN2 |  |
|  | CREB3L2 |  |
|  | DCAF10 |  |
|  | COPE |  |
|  | C1orf52 |  |
|  | RBBP6 |  |
|  | OCLM |  |
|  | NSFL1C |  |
|  | BTBD10 |  |
|  | SENP5 |  |
|  | MRPL16 |  |
|  | RHOA-IT1 |  |
|  | MIR624 |  |
|  | GTF2I |  |
|  | UBR3 |  |
|  | RNU1-19P |  |
|  | IPO9 |  |
|  | RHOQP2 |  |
|  | RAB18 |  |
|  | KDM5A |  |
|  | PSMB7 |  |
|  | GTF2E2 |  |
|  | UTP18 |  |
|  | NAMPTL |  |
|  | PPM1B |  |
|  | TAF15 |  |
|  | SYF2 |  |
|  | AHCYL1 |  |
|  | MIR3115 |  |
|  | DENND1B |  |
|  | SEC24C |  |
|  | DCAF13 |  |
|  | IRF2 |  |
|  | NAA25 |  |
|  | FLVCR1 |  |
|  | TAF5 |  |
|  | NEK4 |  |
|  | CAPRIN1 |  |
|  | TTL |  |
|  | SREK1IP1 |  |
|  | AP3S2 |  |
|  | LPXN |  |
|  | FRMD4B |  |
|  | SCAF4 |  |
|  | SPCS2 |  |
|  | KIAA0754 |  |
|  | APOBEC3G |  |
|  | SMG7 |  |
|  | BLVRA |  |
|  | DHTKD1 |  |
|  | OTTHUMG00000018457 |  |
|  | SRSF3 |  |
|  | NLRP1 |  |
|  | DNAJC15 |  |
|  | SLC30A5 |  |
|  | BTBD7 |  |
|  | BAZ2A |  |
|  | SLC15A2 |  |
|  | SETD3 |  |
|  | CLIP4 |  |
|  | PNPT1 |  |
|  | ATP6V1H |  |
|  | HMGB2 |  |
|  | SEC63 |  |
|  | GUF1 |  |
|  | CCNDBP1 |  |
|  | MAN1A2 |  |
|  | NME8 |  |
|  | LILRB4 |  |
|  | RALB |  |
|  | PON2 |  |
|  | AP3D1 |  |
|  | EID1 |  |
|  | ATRN |  |
|  | MIR643 |  |
|  | OR56B1 |  |
|  | ZCRB1 |  |
|  | CNOT8 |  |
|  | KDM6A |  |
|  | NUP214 |  |
|  | SUCLG1 |  |
|  | BCL10 |  |
|  | SPTLC1 |  |
|  | HIST1H3D |  |
|  | SLC46A3 |  |
|  | TLK1 |  |
|  | SYT11 |  |
|  | LOC101060578 |  |
|  | OTTHUMG00000020030 |  |
|  | AP2A1 |  |
|  | CSTF3 |  |
|  | CWC25 |  |
|  | ICAM1 |  |
|  | CNIH |  |
|  | CDC73 |  |
|  | KMT2D |  |
|  | C16orf62 |  |
|  | ATP6V0C |  |
|  | CIDEB |  |
|  | CEP104 |  |
|  | NAPB |  |
|  | TLR10 |  |
|  | CSRP1 |  |
|  | CD99P1 |  |
|  | SACM1L |  |
|  | RNA5SP85 |  |
|  | SNORA46 |  |
|  | GLTP |  |
|  | YY1 |  |
|  | ZNF280C |  |
|  | SNX6 |  |
|  | MYL6 |  |
|  | GNG2 |  |
|  | UBE2F |  |
|  | C8orf76 |  |
|  | SLC17A5 |  |
|  | PSPC1 |  |
|  | LRIG2 |  |
|  | HIST2H2AB |  |
|  | KDM2A |  |
|  | LOC100507217 |  |
|  | PRKDC |  |
|  | SLU7 |  |
|  | GUCY1A3 |  |
|  | ZNF451 |  |
|  | CEP170 |  |
|  | TNFAIP3 |  |
|  | QPCT |  |
|  | SEPT2 |  |
|  | GMIP |  |
|  | CRKL |  |
|  | LINC00657 |  |
|  | SASH3 |  |
|  | YEATS2 |  |
|  | G2E3 |  |
|  | PSMB9 |  |
|  | EIF3C |  |
|  | QTRTD1 |  |
|  | CASC4 |  |
|  | ATF2 |  |
|  | SMURF2 |  |
|  | CENPE |  |
|  | ALG6 |  |
|  | ARHGEF2 |  |
|  | CBWD3 |  |
|  | RFX7 |  |
|  | 1-Mar |  |
|  | PSMA7 |  |
|  | FAM21C |  |
|  | PCMTD1 |  |
|  | VCP |  |
|  | FAM21B |  |
|  | ZFYVE26 |  |
|  | NUDT16 |  |
|  | TSN |  |
|  | PSMD2 |  |
|  | PFDN1 |  |
|  | CLEC6A |  |
|  | FBXW7 |  |
|  | BARD1 |  |
|  | ARID2 |  |
|  | PRR14L |  |
|  | CCDC6 |  |
|  | STAU1 |  |
|  | HECTD4 |  |
|  | INPPL1 |  |
|  | VRK2 |  |
|  | RNA5SP82 |  |
|  | SNX17 |  |
|  | MSL1 |  |
|  | DERL1 |  |
|  | APPL1 |  |
|  | TMEM87B |  |
|  | PCF11 |  |
|  | GALNT7 |  |
|  | SKA2 |  |
|  | SETD5-AS1 |  |
|  | RNA5SP335 |  |
|  | RSRC2 |  |
|  | OTTHUMG00000161726 |  |
|  | SRI |  |
|  | GPATCH2L |  |
|  | UTP11L |  |
|  | SBNO1 |  |
|  | AKAP11 |  |
|  | RCSD1 |  |
|  | UBE2B |  |
|  | PI4K2A |  |
|  | AFF1 |  |
|  | CLPX |  |
|  | FAM192A |  |
|  | ZNF385A |  |
|  | YAF2 |  |
|  | CCBL2 |  |
|  | MCU |  |
|  | SETDB2 |  |
|  | ATP5A1 |  |
|  | HM13 |  |
|  | FERMT3 |  |
|  | STIP1 |  |
|  | MAP4K3 |  |
|  | FCGRT |  |
|  | NT5C |  |
|  | CNIH4 |  |
|  | GOSR1 |  |
|  | TMSB4X |  |
|  | UBE2Z |  |
|  | CALHM2 |  |
|  | PPP1R14BP3 |  |
|  | MTDH |  |
|  | SP1 |  |
|  | GPX4 |  |
|  | PDSS1 |  |
|  | SECISBP2L |  |
|  | QRSL1 |  |
|  | STK24 |  |
|  | TM2D1 |  |
|  | DEK |  |
|  | STAT3 |  |
|  | DUSP3 |  |
|  | LINC00294 |  |
|  | CUL4A |  |
|  | POLH |  |
|  | PIAS2 |  |
|  | EIF6 |  |
|  | TAP1 |  |
|  | GOLGB1 |  |
|  | KPNA1 |  |
|  | NAPA |  |
|  | LPCAT1 |  |
|  | HEXIM1 |  |
|  | KDM3B |  |
|  | VPS13D |  |
|  | STK39 |  |
|  | TMEM230 |  |
|  | RFXAP |  |
|  | CHTOP |  |
|  | FBXO9 |  |
|  | LTV1 |  |
|  | CALCOCO2 |  |
|  | WDFY1 |  |
|  | USP38 |  |
|  | LDHA |  |
|  | MAT2A |  |
|  | ZBTB11 |  |
|  | ANXA7 |  |
|  | CMC2 |  |
|  | TAF2 |  |
|  | HCFC2 |  |
|  | OGDH |  |
|  | LRRC37A2 |  |
|  | TNPO1 |  |
|  | RRP36 |  |
|  | KSR1 |  |
|  | ADAM1A |  |
|  | FLJ45340 |  |
|  | MCCC2 |  |
|  | TRMT1L |  |
|  | SCN9A |  |
|  | UQCR10 |  |
|  | MGRN1 |  |
|  | TGDS |  |
|  | RRAGA |  |
|  | BLVRB |  |
|  | ELP4 |  |
|  | PLEKHM1 |  |
|  | OXA1L |  |
|  | ARHGAP11A |  |
|  | MIR3916 |  |
|  | LOC100506190 |  |
|  | RNF122 |  |
|  | FKSG29 |  |
|  | SCCPDH |  |
|  | C11orf54 |  |
|  | DYNLRB1 |  |
|  | RNU1-14P |  |
|  | C14orf166 |  |
|  | RCC2 |  |
|  | SRSF10 |  |
|  | RABGAP1L-IT1 |  |
|  | WWP2 |  |
|  | AGO3 |  |
|  | RBBP4 |  |
|  | GBF1 |  |
|  | MICU2 |  |
|  | USP34 |  |
|  | IFITM3 |  |
|  | SUPT6H |  |
|  | M6PR |  |
|  | NBR1 |  |
|  | IL15 |  |
|  | CNTRL |  |
|  | SIGLEC7 |  |
|  | ZNF26 |  |
|  | HIPK1 |  |
|  | PGLS |  |
|  | NDUFS4 |  |
|  | ABCB10 |  |
|  | TOPBP1 |  |
|  | OAS2 |  |
|  | ZNF394 |  |
|  | CLTA |  |
|  | TBC1D2 |  |
|  | GPR108 |  |
|  | OTTHUMG00000162284 |  |
|  | PSMC2 |  |
|  | TOR1A |  |
|  | ATAD2 |  |
|  | HIST1H2BC |  |
|  | TCEB3 |  |
|  | TXNDC15 |  |
|  | MYCBP2 |  |
|  | SMIM7 |  |
|  | NBAS |  |
|  | MAPK1IP1L |  |
|  | AIMP2 |  |
|  | HSPA14 |  |
|  | GGPS1 |  |
|  | GLCE |  |
|  | MRPL47 |  |
|  | CCDC132 |  |
|  | SH2D1B |  |
|  | MMADHC |  |
|  | MTIF2 |  |
|  | MAN2A2 |  |
|  | PDS5A |  |
|  | AUH |  |
|  | PSMD7 |  |
|  | PAG1 |  |
|  | ATG2B |  |
|  | MIR579 |  |
|  | XRCC4 |  |
|  | SND1 |  |
|  | LRRC37B |  |
|  | PDSS2 |  |
|  | RNA5SP263 |  |
|  | SNORA79 |  |
|  | PAK1IP1 |  |
|  | ACAT1 |  |
|  | LINC00672 |  |
|  | CHD8 |  |
|  | VTI1A |  |
|  | AK2 |  |
|  | TES |  |
|  | EIF1 |  |
|  | YLPM1 |  |
|  | MAGT1 |  |
|  | OTTHUMG00000017761 |  |
|  | DHX8 |  |
|  | AGA |  |
|  | VEZT |  |
|  | OTTHUMG00000153914 |  |
|  | RLF |  |
|  | PAPOLA |  |
|  | LOC401320 |  |
|  | FAM179B |  |
|  | NOA1 |  |
|  | REV3L |  |
|  | EIF3CL |  |
|  | H3F3B |  |
|  | MRPS5 |  |
|  | SP100 |  |
|  | CCNYL1 |  |
|  | ISG20L2 |  |
|  | VPS53 |  |
|  | PI4KA |  |
|  | BDP1 |  |
|  | DGKE |  |
|  | ASB8 |  |
|  | TRAPPC13 |  |
|  | PSME3 |  |
|  | CD2AP |  |
|  | KIAA0907 |  |
|  | PTS |  |
|  | PPP1CB |  |
|  | JUND |  |
|  | HIAT1 |  |
|  | TGOLN2 |  |
|  | BRWD1 |  |
|  | POLR2E |  |
|  | ATRAID |  |
|  | WBP4 |  |
|  | EIF4E3 |  |
|  | GTF2B |  |
|  | TMEM19 |  |
|  | SRSF6 |  |
|  | MED17 |  |
|  | MYNN |  |
|  | EAF1 |  |
|  | HNRNPD |  |
|  | CSK |  |
|  | SNX29P1 |  |
|  | ELAVL1 |  |
|  | NDUFS1 |  |
|  | GLO1 |  |
|  | NUP93 |  |
|  | CTDNEP1 |  |
|  | DENND6A |  |
|  | LATS1 |  |
|  | LRRC8D |  |
|  | MBNL2 |  |
|  | MFAP1 |  |
|  | CERS2 |  |
|  | WDR83OS |  |
|  | HIST2H2AC |  |
|  | NBPF10 |  |
|  | ABCC1 |  |
|  | LRRC37A |  |
|  | NOD2 |  |
|  | PPP2R2A |  |
|  | MTRF1L |  |
|  | SCO1 |  |
|  | KHDRBS1 |  |
|  | RALGAPA2 |  |
|  | GPR141 |  |
|  | MRPS25 |  |
|  | XPO5 |  |
|  | DBT |  |
|  | ING3 |  |
|  | CASP8 |  |
|  | NDUFB10 |  |
|  | P4HB |  |
|  | ERLIN1 |  |
|  | RSRC1 |  |
|  | APOL6 |  |
|  | ASXL2 |  |
|  | WASL |  |
|  | RIPK3 |  |
|  | OTTHUMG00000034463 |  |
|  | EMC4 |  |
|  | RNASEK |  |
|  | SRFBP1 |  |
|  | LMO4 |  |
|  | KDELR1 |  |
|  | USP9X |  |
|  | SPATA5L1 |  |
|  | RTP4 |  |
|  | LRRC37BP1 |  |
|  | N4BP2 |  |
|  | MIR922 |  |
|  | PDHB |  |
|  | RBM12 |  |
|  | SHPRH |  |
|  | TRAM1 |  |
|  | FBXO38 |  |
|  | PBX2 |  |
|  | EIF3M |  |
|  | EHBP1L1 |  |
|  | CCDC84 |  |
|  | RARS2 |  |
|  | NUS1 |  |
|  | STAC3 |  |
|  | POLG2 |  |
|  | YWHAZP3 |  |
|  | NSUN6 |  |
|  | PIK3R5 |  |
|  | ATP5H |  |
|  | SUGP2 |  |
|  | RAB3GAP2 |  |
|  | TMBIM1 |  |
|  | PDCD6IP |  |
|  | CCT6P1 |  |
|  | NAB1 |  |
|  | DPY19L3 |  |
|  | RNY4P8 |  |
|  | ZXDC |  |
|  | GXYLT1 |  |
|  | GBA2 |  |
|  | OSBPL3 |  |
|  | PMM2 |  |
|  | FLJ45445 |  |
|  | SH3BGRL3 |  |
|  | CLOCK |  |
|  | MED23 |  |
|  | RIOK1 |  |
|  | DNAJB11 |  |
|  | TAF10 |  |
|  | SMG8 |  |
|  | RAPGEF1 |  |
|  | NUP62 |  |
|  | HNRNPH3 |  |
|  | SEC61B |  |
|  | SEC23A |  |
|  | PSMB8 |  |
|  | LATS2 |  |
|  | TMEM138 |  |
|  | GARS |  |
|  | PTP4A1 |  |
|  | DYNC1LI2 |  |
|  | MITD1 |  |
|  | RNA5SP370 |  |
|  | DOCK7 |  |
|  | GSTK1 |  |
|  | RNU7-59P |  |
|  | RBM39 |  |
|  | ZBTB44 |  |
|  | TRIM24 |  |
|  | MKI67IP |  |
|  | RNA5SP389 |  |
|  | TMEM140 |  |
|  | MRPS15 |  |
|  | FAM76B |  |
|  | HAT1 |  |
|  | DCTN6 |  |
|  | ATXN3 |  |
|  | SF3B14 |  |
|  | OTUD4 |  |
|  | MIRLET7F1 |  |
|  | UBE2L3 |  |
|  | TMEM14C |  |
|  | PRPF18 |  |
|  | STK40 |  |
|  | SNRNP48 |  |
|  | NDUFB5 |  |
|  | SOX4 |  |
|  | HIST1H2AM |  |
|  | OPN3 |  |
|  | FNBP1 |  |
|  | AMFR |  |
|  | NBPF9 |  |
|  | SKP1 |  |
|  | ARL6IP5 |  |
|  | C5orf24 |  |
|  | RNF166 |  |
|  | UBE2E1 |  |
|  | LARP4B |  |
|  | UBQLN1 |  |
|  | RNA5SP493 |  |
|  | SLC23A2 |  |
|  | SECISBP2 |  |
|  | KRAS |  |
|  | TSC22D2 |  |
|  | HPGD |  |
|  | FH |  |
|  | RBM3 |  |
|  | ANKFY1 |  |
|  | NUDT5 |  |
|  | WWTR1-IT1 |  |
|  | MKLN1 |  |
|  | SAR1A |  |
|  | LOC100505815 |  |
|  | RIN3 |  |
|  | ZCCHC10 |  |
|  | MGAT2 |  |
|  | GBE1 |  |
|  | ERVK13-1 |  |
|  | RPS6KA5 |  |
|  | HIPK2 |  |
|  | RBBP7 |  |
|  | PRRC2C |  |
|  | LOC100132913 |  |
|  | BUD13 |  |
|  | DPF2 |  |
|  | CLCN3 |  |
|  | UBE3C |  |
|  | FAM73A |  |
|  | HIST1H1C |  |
|  | LINC00849 |  |
|  | NUP155 |  |
|  | C7orf55-LUC7L2 |  |
|  | ELMOD2 |  |
|  | ARMC10 |  |
|  | RNY4P7 |  |
|  | MIR3684 |  |
|  | TPTEP1 |  |
|  | RUFY1 |  |
|  | SLA |  |
|  | PROSC |  |
|  | HEG1 |  |
|  | MFF |  |
|  | C5orf20 |  |
|  | UBR4 |  |
|  | ADAR |  |
|  | PA2G4P4 |  |
|  | CSNK2A1P |  |
|  | AP1B1 |  |
|  | ARNTL |  |
|  | TLE4 |  |
|  | RNF214 |  |
|  | CCDC111 |  |
|  | EPT1 |  |
|  | C10orf137 |  |
|  | SMN1 |  |
|  | PIP5K1A |  |
|  | ARL8A |  |
|  | NDRG1 |  |
|  | PPP2R1B |  |
|  | STAMBP |  |
|  | KIAA1919 |  |
|  | MIR553 |  |
|  | TCP11L1 |  |
|  | NELFCD |  |
|  | KLHDC10 |  |
|  | PROSER1 |  |
|  | HNRNPC |  |
|  | CCT2 |  |
|  | BRAP |  |
|  | OTTHUMG00000022515 |  |
|  | UBA1 |  |
|  | ARID4A |  |
|  | TM2D2 |  |
|  | KCNJ2 |  |
|  | COG3 |  |
|  | RBM7 |  |
|  | CDK8 |  |
|  | L3MBTL3 |  |
|  | EIF2S2 |  |
|  | RER1 |  |
|  | PITHD1 |  |
|  | C4orf33 |  |
|  | LRCH3 |  |
|  | NDUFB6 |  |
|  | SYNGR2 |  |
|  | NPHP3 |  |
|  | GK-IT1 |  |
|  | LOC285074 |  |
|  | ELMO2 |  |
|  | ATXN7L3 |  |
|  | GOLT1B |  |
|  | PARK7 |  |
|  | LAMTOR1 |  |
|  | PHEX |  |
|  | BCCIP |  |
|  | CBWD5 |  |
|  | UBE4A |  |
|  | MTMR3 |  |
|  | UFSP2 |  |
|  | SMEK2 |  |
|  | OTTHUMG00000162476 |  |
|  | WDR70 |  |
|  | TP53BP2 |  |
|  | RCOR3 |  |
|  | ARL11 |  |
|  | CLK4 |  |
|  | PDHA1 |  |
|  | GGNBP2 |  |
|  | PSMD6 |  |
|  | DUS2L |  |
|  | OTTHUMG00000042553 |  |
|  | IFIT5 |  |
|  | RNA5SP355 |  |
|  | HTATIP2 |  |
|  | PSMD14 |  |
|  | NPIPB6 |  |
|  | GTF2H2B |  |
|  | NKTR |  |
|  | ABHD6 |  |
|  | CCNL1 |  |
|  | CD63 |  |
|  | BTF3L4 |  |
|  | MED21 |  |
|  | RNF34 |  |
|  | MYO9A |  |
|  | IDH2 |  |
|  | PTPN18 |  |
|  | SNRPD1 |  |
|  | UBE2N |  |
|  | SERPINB9 |  |
|  | HSP90AA1 |  |
|  | GOLPH3 |  |
|  | ARL6IP1 |  |
|  | NEK6 |  |
|  | TRIM21 |  |
|  | ANKRD40 |  |
|  | TNPO3 |  |
|  | ATP5EP2 |  |
|  | MAP4K4 |  |
|  | HK1 |  |
|  | SMARCC1 |  |
|  | MARS |  |
|  | COPG1 |  |
|  | TBC1D14 |  |
|  | DAZAP2 |  |
|  | NFATC2IP |  |
|  | TMCO1 |  |
|  | MIR1273A |  |
|  | MRFAP1 |  |
|  | SLX1A |  |
|  | TMEM185B |  |
|  | SNX16 |  |
|  | SLC10A7 |  |
|  | RNF145 |  |
|  | ACLY |  |
|  | PKD2 |  |
|  | MTOR |  |
|  | MACF1 |  |
|  | CNTF |  |
|  | S100A4 |  |
|  | EIF4A3 |  |
|  | TNS3 |  |
|  | RSC1A1 |  |
|  | COX20 |  |
|  | LONRF1 |  |
|  | RNF170 |  |
|  | NUP98 |  |
|  | HDLBP |  |
|  | PIGT |  |
|  | DOCK10 |  |
|  | MCM9 |  |
|  | ATP6AP1 |  |
|  | CMAHP |  |
|  | MFSD8 |  |
|  | RNF2 |  |
|  | YTHDF2 |  |
|  | B2M |  |
|  | SGMS1 |  |
|  | ADK |  |
|  | RTFDC1 |  |
|  | PUS3 |  |
|  | AKIRIN1 |  |
|  | PDPK1 |  |
|  | N4BP1 |  |
|  | QARS |  |
|  | ZDHHC13 |  |
|  | ZNF518A |  |
|  | TCIRG1 |  |
|  | MEMO1 |  |
|  | MFSD11 |  |
|  | GPN1 |  |
|  | HAUS1 |  |
|  | PLA2G16 |  |
|  | TUG1 |  |
|  | COX19 |  |
|  | GSK3A |  |
|  | KLF10 |  |
|  | COX6A1P2 |  |
|  | PPP4C |  |
|  | KMT2E |  |
|  | ZNF25 |  |
|  | CDCA7 |  |
|  | SH3BP5-AS1 |  |
|  | OTTHUMG00000159786 |  |
|  | EMC2 |  |
|  | FAM43A |  |
|  | NXF1 |  |
|  | CD38 |  |
|  | ATF7 |  |
|  | ACADVL |  |
|  | CUL1 |  |
|  | OSGIN2 |  |
|  | LRRC33 |  |
|  | TOP3A |  |
|  | RBM25 |  |
|  | CYP51A1 |  |
|  | CBFB |  |
|  | DNTTIP1 |  |
|  | HIGD2A |  |
|  | PYCARD |  |
|  | DAAM1 |  |
|  | LSMD1 |  |
|  | SRSF4 |  |
|  | BRD2 |  |
|  | TMA16 |  |
|  | ATG14 |  |
|  | KNSTRN |  |
|  | ZNF706 |  |
|  | SSH1 |  |
|  | GATC |  |
|  | OTTHUMG00000164254 |  |
|  | WAS |  |
|  | NOL8 |  |
|  | LOC100506282 |  |
|  | ECT2 |  |
|  | XPNPEP1 |  |
|  | PRMT10 |  |
|  | FBXO28 |  |
|  | VPS28 |  |
|  | DHRS7 |  |
|  | ABHD10 |  |
|  | DEGS1 |  |
|  | TDRD3 |  |
|  | TIMM8B |  |
|  | SMC1A |  |
|  | HSPA9 |  |
|  | TOR1B |  |
|  | OTTHUMG00000018585 |  |
|  | C2orf49 |  |
|  | ZNFX1 |  |
|  | SDHAF2 |  |
|  | PHF12 |  |
|  | C6orf106 |  |
|  | MIR581 |  |
|  | MIR32 |  |
|  | RBM22 |  |
|  | POGZ |  |
|  | PTAR1 |  |
|  | EIF2B1 |  |
|  | WTAP |  |
|  | FAM35DP |  |
|  | KARS |  |
|  | METTL9 |  |
|  | E2F3-IT1 |  |
|  | TPP2 |  |
|  | MLH1 |  |
|  | LOC100505812 |  |
|  | E2F4 |  |
|  | COPZ1 |  |
|  | PTPRC |  |
|  | RNA5SP141 |  |
|  | FAM13A-AS1 |  |
|  | KANSL1L |  |
|  | KLHL18 |  |
|  | CRNKL1 |  |
|  | GMFB |  |
|  | TRRAP |  |
|  | OTTHUMG00000154423 |  |
|  | MEF2D |  |
|  | RAB11FIP2 |  |
|  | ENSA |  |
|  | NIP7 |  |
|  | WDR45B |  |
|  | WDR33 |  |
|  | PRDM1 |  |
|  | SLC39A11 |  |
|  | TAF1 |  |
|  | YWHAZ |  |
|  | HIST4H4 |  |
|  | HNRNPU |  |
|  | SNORA48 |  |
|  | PWP1 |  |
|  | RAB1B |  |
|  | RBM15 |  |
|  | KIAA0020 |  |
|  | MAP2K4 |  |
|  | UBN1 |  |
|  | OTTHUMG00000016846 |  |
|  | POLA2 |  |
|  | RPRD1A |  |
|  | ZNF592 |  |
|  | CD99 |  |
|  | RNF24 |  |
|  | KIF3B |  |
|  | AMMECR1L |  |
|  | PPP1CA |  |
|  | GRAMD1B |  |
|  | GPR89C |  |
|  | APOL3 |  |
|  | NUFIP2 |  |
|  | PTGDS |  |
|  | OTTHUMG00000164068 |  |
|  | ARFGAP3 |  |
|  | CTNNB1 |  |
|  | GID8 |  |
|  | PLEKHB2 |  |
|  | SENP2 |  |
|  | GMPS |  |
|  | AUP1 |  |
|  | DARS2 |  |
|  | TCF25 |  |
|  | CDC42BPB |  |
|  | ARHGAP19 |  |
|  | TBC1D2B |  |
|  | DOK2 |  |
|  | RSF1 |  |
|  | GTPBP4 |  |
|  | GMFG |  |
|  | PMS2P1 |  |
|  | ATP5L |  |
|  | KIAA0930 |  |
|  | CD1D |  |
|  | RBMS1 |  |
|  | SMAP1 |  |
|  | PARVG |  |
|  | MCOLN1 |  |
|  | FASTKD2 |  |
|  | RNA5-8S5 |  |
|  | TMEM183B |  |
|  | ABHD15 |  |
|  | C8orf59 |  |
|  | REXO2 |  |
|  | TANGO6 |  |
|  | TCEA1 |  |
|  | ZYX |  |
|  | GYG1 |  |
|  | 5-Mar |  |
|  | GLT8D1 |  |
|  | PAPD5 |  |
|  | TMEM60 |  |
|  | LOC100505828 |  |
|  | LOC642799 |  |
|  | SMA4 |  |
|  | DLG1 |  |
|  | ORMDL1 |  |
|  | DCUN1D5 |  |
|  | CCDC117 |  |
|  | NFATC3 |  |
|  | AGAP6 |  |
|  | DIS3L |  |
|  | TRMT61B |  |
|  | H2AFV |  |
|  | COX15 |  |
|  | FAM204A |  |
|  | ARFGEF2 |  |
|  | METAP1 |  |
|  | ARHGAP25 |  |
|  | ARHGAP35 |  |
|  | RTCB |  |
|  | SLC12A2 |  |
|  | PMS1 |  |
|  | OTTHUMG00000133759 |  |
|  | WAPAL |  |
|  | DST |  |
|  | CWC15 |  |
|  | DDOST |  |
|  | SRRM2 |  |
|  | TAF13 |  |
|  | RASSF3 |  |
|  | RHOG |  |
|  | KATNBL1 |  |
|  | MED8 |  |
|  | POLDIP3 |  |
|  | C6orf120 |  |
|  | SMARCE1 |  |
|  | KANSL2 |  |
|  | C10orf128 |  |
|  | CEBPG |  |
|  | C10orf12 |  |
|  | CAMK2D |  |
|  | STAU2 |  |
|  | PI4KB |  |
|  | KLHL24 |  |
|  | CAPZB |  |
|  | EXOSC1 |  |
|  | SLC15A3 |  |
|  | RIPK1 |  |
|  | LOC100287497 |  |
|  | SUDS3 |  |
|  | THOC7 |  |
|  | UBE2L6 |  |
|  | RNF167 |  |
|  | NSUN2 |  |
|  | CARD17 |  |
|  | NUDT19 |  |
|  | CCNC |  |
|  | NR2C2 |  |
|  | ATG16L2 |  |
|  | PIGY |  |
|  | SNX29 |  |
|  | TM2D3 |  |
|  | ANKRD27 |  |
|  | LOC728734 |  |
|  | OTTHUMG00000059818 |  |
|  | ELP2 |  |
|  | C9orf64 |  |
|  | RNA5SP122 |  |
|  | MAT2B |  |
|  | CRYZL1 |  |
|  | ZDHHC21 |  |
|  | TMEM126B |  |
|  | TMOD3 |  |
|  | FAM206A |  |
|  | MSH5 |  |
|  | COG5 |  |
|  | SUCO |  |
|  | EIF4E2 |  |
|  | XRCC5 |  |
|  | CDADC1 |  |
|  | BSG |  |
|  | DDB1 |  |
|  | TMEM259 |  |
|  | VAMP8 |  |
|  | KTI12 |  |
|  | HEBP2 |  |
|  | FAM35BP |  |
|  | RNA5SP145 |  |
|  | COX5B |  |
|  | SDHC |  |
|  | SLC7A5 |  |
|  | TAF12 |  |
|  | NUP133 |  |
|  | EPG5 |  |
|  | ZNF410 |  |
|  | KIAA0430 |  |
|  | NANS |  |
|  | ARCN1 |  |
|  | BAZ1B |  |
|  | OTTHUMG00000073673 |  |
|  | DNAAF2 |  |
|  | KCTD6 |  |
|  | PHACTR4 |  |
|  | SRP14 |  |
|  | ELOVL5 |  |
|  | NADK |  |
|  | INSR |  |
|  | ULK4P1 |  |
|  | GTF2H2D |  |
|  | PHF14 |  |
|  | CUL2 |  |
|  | MAGOH |  |
|  | TARBP1 |  |
|  | EPB41L2 |  |
|  | DROSHA |  |
|  | RAB22A |  |
|  | LOC100507463 |  |
|  | RNY4P25 |  |
|  | UBE2G2 |  |
|  | ARHGDIA |  |
|  | PIAS1 |  |
|  | GSTP1 |  |
|  | PSMB6 |  |
|  | LOC145783 |  |
|  | POU2F2 |  |
|  | MKL1 |  |
|  | PMS2P5 |  |
|  | BIVM |  |
|  | ANP32AP1 |  |
|  | PPIF |  |
|  | SNORA32 |  |
|  | AREL1 |  |
|  | DNMBP |  |
|  | FAM157B |  |
|  | DPH3 |  |
|  | CXCL10 |  |
|  | GFM1 |  |
|  | PDIA6 |  |
|  | TIGD2 |  |
|  | PWWP2A |  |
|  | RAB7L1 |  |
|  | ATXN2 |  |
|  | TMLHE |  |
|  | IPO7 |  |
|  | TRPV2 |  |
|  | OTTHUMG00000152618 |  |
|  | WDR67 |  |
|  | PSMA5 |  |
|  | HACL1 |  |
|  | G6PC3 |  |
|  | ARF3 |  |
|  | TRAPPC10 |  |
|  | PARN |  |
|  | ALKBH1 |  |
|  | SNORD51 |  |
|  | SPTY2D1 |  |
|  | ACSL3 |  |
|  | AAGAB |  |
|  | TFCP2 |  |
|  | RNU2-7P |  |
|  | ITM2C |  |
|  | SEMA4A |  |
|  | WDR5B |  |
|  | ALYREF |  |
|  | CDC37 |  |
|  | PPM1L |  |
|  | R3HDM2 |  |
|  | SERP1 |  |
|  | HDAC8 |  |
|  | MDH1 |  |
|  | OLA1 |  |
|  | TRIM37 |  |
|  | CDC27 |  |
|  | JTB |  |
|  | DERA |  |
|  | FAM120AOS |  |
|  | GPR114 |  |
|  | INTS10 |  |
|  | TP53RK |  |
|  | CSNK2A1 |  |
|  | CIAO1 |  |
|  | HIBCH |  |
|  | RUNDC1 |  |
|  | WHSC1L1 |  |
|  | HDHD2 |  |
|  | COX7B |  |
|  | HNRNPA3 |  |
|  | UBC |  |
|  | SLBP |  |
|  | FBXW11 |  |
|  | LOC286437 |  |
|  | CEP95 |  |
|  | NECAP2 |  |
|  | RNA5SP134 |  |
|  | PCID2 |  |
|  | RBM12B |  |
|  | C5orf42 |  |
|  | SUB1 |  |
|  | DDX19B |  |
|  | TAF11 |  |
|  | ADI1 |  |
|  | FBXO30 |  |
|  | PNRC2 |  |
|  | ALG5 |  |
|  | UIMC1 |  |
|  | PDZD11 |  |
|  | CAPN1 |  |
|  | DRG1 |  |
|  | LSM6 |  |
|  | TOX4 |  |
|  | ELP3 |  |
|  | BRE |  |
|  | MRPS36 |  |
|  | SNAPIN |  |
|  | IKBKB |  |
|  | SAP130 |  |
|  | OTTHUMG00000031766 |  |
|  | IPO8 |  |
|  | LSM14B |  |
|  | DEDD |  |
|  | SPPL3 |  |
|  | ATF6B |  |
|  | SRCAP |  |
|  | MCCC1 |  |
|  | PARP6 |  |
|  | PAPD7 |  |
|  | ZFAT |  |
|  | FGFR1OP |  |
|  | ZKSCAN1 |  |
|  | NPIPA3 |  |
|  | ACOT13 |  |
|  | DLST |  |
|  | LGMN |  |
|  | NRBP1 |  |
|  | HM13-IT1 |  |
|  | DIMT1 |  |
|  | LRCH1 |  |
|  | KLF3 |  |
|  | CLCN7 |  |
|  | SP3 |  |
|  | RAB35 |  |
|  | ZMAT2 |  |
|  | R3HCC1L |  |
|  | DNAJC2 |  |
|  | SNORD1A |  |
|  | YIPF3 |  |
|  | C7orf73 |  |
|  | PLIN3 |  |
|  | HLA-DQB2 |  |
|  | USP45 |  |
|  | EHD4 |  |
|  | MLH3 |  |
|  | RNF19A |  |
|  | PLEKHM2 |  |
|  | FASTKD3 |  |
|  | MTIF3 |  |
|  | SLC25A19 |  |
|  | RBM17 |  |
|  | DTX2P1-UPK3BP1-PMS2P11 |  |
|  | C11orf58 |  |
|  | GRAMD4 |  |
|  | ITGB1BP1 |  |
|  | PABPN1 |  |
|  | GBP1P1 |  |
|  | TBCE |  |
|  | SPRTN |  |
|  | GNG5P2 |  |
|  | UHRF2 |  |
|  | ARHGAP30 |  |
|  | MAPK8 |  |
|  | LIMS1 |  |
|  | SART3 |  |
|  | LBR |  |
|  | AHSA2 |  |
|  | LOC399753 |  |
|  | RARA |  |
|  | LOC100506007 |  |
|  | GCNT1 |  |
|  | VDAC3 |  |
|  | GABBR1 |  |
|  | RLIM |  |
|  | CHST7 |  |
|  | RNA5SP368 |  |
|  | ZBTB8OS |  |
|  | ERCC1 |  |
|  | CCR5 |  |
|  | RNY4P19 |  |
|  | ASH2L |  |
|  | GUSBP9 |  |
|  | CTNS |  |
|  | FAM122B |  |
|  | GNL3L |  |
|  | CRLS1 |  |
|  | UBXN4 |  |
|  | ETV3 |  |
|  | TNFRSF21 |  |
|  | UBE2H |  |
|  | MBNL1 |  |
|  | KIAA0319L |  |
|  | CSPP1 |  |
|  | MRPL3 |  |
|  | TBCEL |  |
|  | PRDM4 |  |
|  | MRPL53 |  |
|  | RNF11 |  |
|  | ITCH-AS1 |  |
|  | COG6 |  |
|  | TMEM109 |  |
|  | FRRS1 |  |
|  | KIAA0146 |  |
|  | FLII |  |
|  | TXNL4A |  |
|  | CPNE2 |  |
|  | KIAA2026 |  |
|  | IDH3G |  |
|  | TVP23B |  |
|  | DCPS |  |
|  | HLA-B |  |
|  | TTLL4 |  |
|  | SKP2 |  |
|  | NDUFB9 |  |
|  | PTPMT1 |  |
|  | TUFM |  |
|  | CORO1B |  |
|  | ZSCAN9 |  |
|  | VHL |  |
|  | FKBP14 |  |
|  | IGFLR1 |  |
|  | ORMDL2 |  |
|  | TMCC1 |  |
|  | WIPF2 |  |
|  | CPOX |  |
|  | MTMR9 |  |
|  | HDX |  |
|  | CTSL1 |  |
|  | PLXND1 |  |
|  | CBWD6 |  |
|  | EIF3K |  |
|  | KATNAL1 |  |
|  | DPP9 |  |
|  | RNF38 |  |
|  | CMTM1 |  |
|  | C14orf159 |  |
|  | HSCB |  |
|  | PRNP |  |
|  | CBR4 |  |
|  | NSMCE4A |  |
|  | FLI1 |  |
|  | ILKAP |  |
|  | PMS2L2 |  |
|  | CCDC69 |  |
|  | C5orf15 |  |
|  | KIAA1524 |  |
|  | UBQLN2 |  |
|  | ATF7IP |  |
|  | GART |  |
|  | CCDC43 |  |
|  | HLA-C |  |
|  | DSN1 |  |
|  | PPP2CB |  |
|  | LTB4R |  |
|  | IMPACT |  |
|  | LIPA |  |
|  | MRPL39 |  |
|  | BRD7 |  |
|  | CPQ |  |
|  | SNX11 |  |
|  | KDM4C |  |
|  | CEBPD |  |
|  | UBL5 |  |
|  | HIST1H2BN |  |
|  | ZBTB34 |  |
|  | NDUFA12 |  |
|  | TRAPPC3 |  |
|  | CNOT4 |  |
|  | C5orf51 |  |
|  | UBP1 |  |
|  | NBPF24 |  |
|  | NR3C1 |  |
|  | TMUB2 |  |
|  | TTC7A |  |
|  | RNASEL |  |
|  | PTPRA |  |
|  | E2F3 |  |
|  | HNRNPL |  |
|  | SLC35B3 |  |
|  | LOC644656 |  |
|  | HDAC9 |  |
|  | SUMO2 |  |
|  | RNU6-28P |  |
|  | RMND1 |  |
|  | PLCL2 |  |
|  | C14orf119 |  |
|  | SUCLA2 |  |
|  | C11orf57 |  |
|  | CAMK1 |  |
|  | ZNF800 |  |
|  | DESI1 |  |
|  | BCAS3 |  |
|  | BCAP29 |  |
|  | LAMP1 |  |
|  | SNRPA1 |  |
|  | MRPL33 |  |
|  | LMAN2 |  |
|  | MIF4GD |  |
|  | TSEN15 |  |
|  | SLC7A6OS |  |
|  | FBXL4 |  |
|  | RBM34 |  |
|  | LAMTOR3 |  |
|  | TMEM208 |  |
|  | ZNF169 |  |
|  | CHCHD2 |  |
|  | CTAGE5 |  |
|  | MDM4 |  |
|  | MIRLET7I |  |
|  | C18orf54 |  |
|  | GPATCH2 |  |
|  | TPPP3 |  |
|  | WDR1 |  |
|  | RALGAPA1 |  |
|  | PPIA |  |
|  | OTTHUMG00000152788 |  |
|  | ENTPD7 |  |
|  | RPGR |  |
|  | ICAM2 |  |
|  | GPI |  |
|  | C15orf38 |  |
|  | KATNA1 |  |
|  | TP53 |  |
|  | ZNF516 |  |
|  | PDXDC2P |  |
|  | POU4F3 |  |
|  | TBC1D22A |  |
|  | PCBD2 |  |
|  | KLHL12 |  |
|  | ZNF789 |  |
|  | ZFAND3 |  |
|  | ARSB |  |
|  | COX7A2L |  |
|  | MAP2K5 |  |
|  | AP2S1 |  |
|  | IAH1 |  |
|  | HIST1H4E |  |
|  | MAX |  |
|  | TMEM199 |  |
|  | RBM33 |  |
|  | C11orf30 |  |
|  | ALDH9A1 |  |
|  | UBXN7 |  |
|  | TYMP |  |
|  | CTNNBL1 |  |
|  | WDR37 |  |
|  | ZNF641 |  |
|  | FLJ43663 |  |
|  | TRIM14 |  |
|  | TMEM43 |  |
|  | RNY3 |  |
|  | TTF2 |  |
|  | TPI1 |  |
|  | OTTHUMG00000003552 |  |
|  | RNF103 |  |
|  | GCLC |  |
|  | MAP4K1 |  |
|  | SYMPK |  |
|  | YTHDF1 |  |
|  | DEPDC5 |  |
|  | PSMB2 |  |
|  | MED10 |  |
|  | FAM200B |  |
|  | MBD4 |  |
|  | MRPL37 |  |
|  | YKT6 |  |
|  | C3orf58 |  |
|  | RNF41 |  |
|  | C14orf2 |  |
|  | EWSR1 |  |
|  | MRPS11 |  |
|  | AKR1A1 |  |
|  | CREBL2 |  |
|  | IFIT2 |  |
|  | ARL5B |  |
|  | NAA38 |  |
|  | VPS26B |  |
|  | FOXO3 |  |
|  | LUC7L |  |
|  | EMC6 |  |
|  | EFTUD1 |  |
|  | HARS2 |  |
|  | SLC25A32 |  |
|  | PTPN1 |  |
|  | ATF4 |  |
|  | SRSF1 |  |
|  | SLC35F6 |  |
|  | NDUFS2 |  |
|  | TSPAN3 |  |
|  | AFG3L2 |  |
|  | KLF11 |  |
|  | ALAS1 |  |
|  | TMEM192 |  |
|  | DSTN |  |
|  | HAUS2 |  |
|  | C17orf62 |  |
|  | UBB |  |
|  | EXT2 |  |
|  | DAGLB |  |
|  | OAS3 |  |
|  | SYAP1 |  |
|  | TXNDC17 |  |
|  | MPPE1 |  |
|  | EXOSC10 |  |
|  | IVNS1ABP |  |
|  | NPIPA5 |  |
|  | BAG5 |  |
|  | BBS10 |  |
|  | RNY5 |  |
|  | CRYGS |  |
|  | KCNRG |  |
|  | HN1 |  |
|  | TMEM164 |  |
|  | POGLUT1 |  |
|  | ZNF622 |  |
|  | VPS52 |  |
|  | DESI2 |  |
|  | MLLT10 |  |
|  | MTMR1 |  |
|  | KPNA6 |  |
|  | JAGN1 |  |
|  | C6orf70 |  |
|  | ZNF318 |  |
|  | PLIN2 |  |
|  | MRRF |  |
|  | SLC35B1 |  |
|  | CFDP1 |  |
|  | TAPBPL |  |
|  | CDV3 |  |
|  | PNPLA6 |  |
|  | TNKS |  |
|  | HECTD3 |  |
|  | WRNIP1 |  |
|  | LOC100130557 |  |
|  | PPP1R7 |  |
|  | HIF1AN |  |
|  | RCN1 |  |
|  | STARD3NL |  |
|  | MDFIC |  |
|  | RPS7 |  |
|  | NUTF2 |  |
|  | FDX1 |  |
|  | ZBTB7A |  |
|  | TAPBP |  |
|  | PLEKHA3 |  |
|  | HMGN3 |  |
|  | FOXN3 |  |
|  | NDST2 |  |
|  | SURF4 |  |
|  | MKRN1 |  |
|  | MRPS18C |  |
|  | GOSR2 |  |
|  | CKS2 |  |
|  | FAM78A |  |
|  | CDK12 |  |
|  | ZBTB21 |  |
|  | SEPSECS |  |
|  | KANSL1 |  |
|  | RMI1 |  |
|  | AGAP7 |  |
|  | STX18 |  |
|  | MRPL51 |  |
|  | FAM46A |  |
|  | TIMM22 |  |
|  | GLE1 |  |
|  | SLC11A2 |  |
|  | RAB28 |  |
|  | DCK |  |
|  | PPM1M |  |
|  | ACIN1 |  |
|  | BCOR |  |
|  | C16orf72 |  |
|  | CWF19L1 |  |
|  | OTTHUMG00000002407 |  |
|  | PRKRIR |  |
|  | TMEM189 |  |
|  | BTN2A1 |  |
|  | AP1M1 |  |
|  | CXCL9 |  |
|  | TRIM34 |  |
|  | ATP6V0A2 |  |
|  | BTN2A2 |  |
|  | VKORC1L1 |  |
|  | FAM219B |  |
|  | OTTHUMG00000037053 |  |
|  | NDUFAF5 |  |
|  | ZRSR2 |  |
|  | C9orf91 |  |
|  | ZCCHC7 |  |
|  | MCM5 |  |
|  | WDR61 |  |
|  | DHDDS |  |
|  | IRF4 |  |
|  | LITAF |  |
|  | ZNF37A |  |
|  | NDUFA2 |  |
|  | COIL |  |
|  | LSM1 |  |
|  | PPM1G |  |
|  | THADA |  |
|  | GTF2H2 |  |
|  | MYH9 |  |
|  | CCDC93 |  |
|  | PALB2 |  |
|  | TXNDC9 |  |
|  | PPCS |  |
|  | USP39 |  |
|  | HMGN2 |  |
|  | SIN3B |  |
|  | MAPK9 |  |
|  | SCNM1 |  |
|  | MRPL19 |  |
|  | SAMM50 |  |
|  | ADHFE1 |  |
|  | H1F0 |  |
|  | ZBTB37 |  |
|  | COPS3 |  |
|  | LOC101060684 |  |
|  | MCM3AP |  |
|  | GGCT |  |
|  | CRYM-AS1 |  |
|  | TAMM41 |  |
|  | RNA5SP195 |  |
|  | NUBP1 |  |
|  | PIGX |  |
|  | SCAMP5 |  |
|  | OARD1 |  |
|  | TMEM206 |  |
|  | ARF1 |  |
|  | CDS2 |  |
|  | TMEM69 |  |
|  | RNY4P11 |  |
|  | HNRNPA1 |  |
|  | GLRX |  |
|  | COG4 |  |
|  | GIGYF2 |  |
|  | ATP9B |  |
|  | SFSWAP |  |
|  | CCNK |  |
|  | SNX19 |  |
|  | RNF181 |  |
|  | PDE6D |  |
|  | LAIR1 |  |
|  | DNAJB12 |  |
|  | DRAP1 |  |
|  | AP3S1 |  |
|  | MTHFD1 |  |
|  | KIAA1715 |  |
|  | C2orf68 |  |
|  | PLEKHM3 |  |
|  | CCDC14 |  |
|  | NPIPL1 |  |
|  | TAP2 |  |
|  | NSMCE2 |  |
|  | TMX2 |  |
|  | CEBPA |  |
|  | JAZF1 |  |
|  | TEX2 |  |
|  | METTL25 |  |
|  | TARDBP |  |
|  | TRNT1 |  |
|  | LOC101060449 |  |
|  | NDUFV3 |  |
|  | FZD1 |  |
|  | IST1 |  |
|  | PCGF3 |  |
|  | GUSB |  |
|  | ERCC3 |  |
|  | DAZAP1 |  |
|  | FBXO34 |  |
|  | SLX4IP |  |
|  | TRIM8 |  |
|  | RPS11 |  |
|  | MED30 |  |
|  | SNORD125 |  |
|  | TIPARP |  |
|  | LARP1 |  |
|  | SF1 |  |
|  | TPK1 |  |
|  | MAP4 |  |
|  | EDF1 |  |
|  | TBCCD1 |  |
|  | MKNK2 |  |
|  | ANKMY2 |  |
|  | COMMD7 |  |
|  | COX4I1 |  |
|  | LACTB2 |  |
|  | TAF5L |  |
|  | RFTN1 |  |
|  | MCPH1 |  |
|  | OSER1 |  |
|  | RN7SL1 |  |
|  | ECD |  |
|  | PHYKPL |  |
|  | ME1 |  |
|  | IGBP1 |  |
|  | EIF4ENIF1 |  |
|  | ZNF236 |  |
|  | CTDSP2 |  |
|  | NDUFA4 |  |
|  | OTTHUMG00000164324 |  |
|  | RBM28 |  |
|  | BZW2 |  |
|  | EPS15L1 |  |
|  | TBC1D20 |  |
|  | STARD4 |  |
|  | OTTHUMG00000152764 |  |
|  | PACRGL |  |
|  | PRKRA |  |
|  | OTTHUMG00000018482 |  |
|  | TCAIM |  |
|  | SCAND2P |  |
|  | TTI1 |  |
|  | IMP3 |  |
|  | SAP30 |  |
|  | LFNG |  |
|  | ABHD17B |  |
|  | PSMD4 |  |
|  | HMBOX1-IT1 |  |
|  | HSH2D |  |
|  | APH1B |  |
|  | RAB4B |  |
|  | LOC652276 |  |
|  | CERK |  |
|  | CCDC77 |  |
|  | CHTF8 |  |
|  | POP4 |  |
|  | CDK5 |  |
|  | TYW5 |  |
|  | MSL2 |  |
|  | ZCCHC9 |  |
|  | CHFR |  |
|  | ZDHHC5 |  |
|  | CAMTA2 |  |
|  | PEX2 |  |
|  | NEDD8 |  |
|  | COX7A2 |  |
|  | UFD1L |  |
|  | APOBEC3B |  |
|  | FAM103A1 |  |
|  | DDX59 |  |
|  | LRPAP1 |  |
|  | ATP5J2 |  |
|  | TERF1 |  |
|  | MRPS7 |  |
|  | PTMA |  |
|  | RABIF |  |
|  | TYK2 |  |
|  | OTTHUMG00000157258 |  |
|  | RBBP9 |  |
|  | AKNA |  |
|  | USP40 |  |
|  | TDG |  |
|  | NRP1 |  |
|  | DNASE2 |  |
|  | ARHGAP27 |  |
|  | MSRB1 |  |
|  | MAPKAP1 |  |
|  | MRPL44 |  |
|  | POLDIP2 |  |
|  | COPS5 |  |
|  | FAM149B1 |  |
|  | ARID3A |  |
|  | SH2D3C |  |
|  | PIGS |  |
|  | AGAP5 |  |
|  | VDAC1 |  |
|  | SMURF1 |  |
|  | GOLGA1 |  |
|  | HYOU1 |  |
|  | ZFX |  |
|  | TMEM104 |  |
|  | UNC119 |  |
|  | LOC202181 |  |
|  | CSNK1D |  |
|  | SPATS2L |  |
|  | TUBB4B |  |
|  | TRAM2 |  |
|  | POM121 |  |
|  | INPP4A |  |
|  | SMARCC2 |  |
|  | HDAC3 |  |
|  | AGO2 |  |
|  | HIST3H2A |  |
|  | HCG11 |  |
|  | HERC2P9 |  |
|  | FBXO22 |  |
|  | LOC100506392 |  |
|  | TSPAN14 |  |
|  | CCNG2 |  |
|  | WASH3P |  |
|  | MIR636 |  |
|  | ADD1 |  |
|  | ARHGAP10 |  |
|  | TLE3 |  |
|  | ZNF638-IT1 |  |
|  | PYGB |  |
|  | ZADH2 |  |
|  | GPR52 |  |
|  | STIL |  |
|  | SNORA30 |  |
|  | P2RX4 |  |
|  | DBNL |  |
|  | RAB5B |  |
|  | NADSYN1 |  |
|  | SETD9 |  |
|  | GNA15 |  |
|  | OTTHUMG00000057501 |  |
|  | CNPY2 |  |
|  | GALNT10 |  |
|  | WAC-AS1 |  |
|  | WDR12 |  |
|  | TSC1 |  |
|  | C12orf45 |  |
|  | INPP5F |  |
|  | RFFL |  |
|  | DCUN1D2 |  |
|  | VAC14 |  |
|  | OTTHUMG00000040105 |  |
|  | PAFAH1B2 |  |
|  | CNPPD1 |  |
|  | FAM129C |  |
|  | PRDM2 |  |
|  | SEC61G |  |
|  | TVP23C |  |
|  | COX17 |  |
|  | ABHD13 |  |
|  | NKRF |  |
|  | DHX35 |  |
|  | TINF2 |  |
|  | CSNK1G1 |  |
|  | DNAJC25 |  |
|  | KIAA1586 |  |
|  | TOMM22 |  |
|  | ERI1 |  |
|  | MYL12B |  |
|  | NUDT4 |  |
|  | PIEZO1 |  |
|  | TRAF7 |  |
|  | EIF3I |  |
|  | CACYBP |  |
|  | SYNE3 |  |
|  | SIAH2 |  |
|  | THOC5 |  |
|  | UBXN11 |  |
|  | MRPL24 |  |
|  | SLC38A10 |  |
|  | LENG8 |  |
|  | NPIPL2 |  |
|  | LOC100294145 |  |
|  | SFT2D3 |  |
|  | DGUOK |  |
|  | INPP5K |  |
|  | KDM2B |  |
|  | NQO2 |  |
|  | UBASH3B |  |
|  | DDX17 |  |
|  | SEC16A |  |
|  | RAB9A |  |
|  | GOLGA8Q |  |
|  | C16orf87 |  |
|  | COX18 |  |
|  | PRCC |  |
|  | PMS2P3 |  |
|  | DSE |  |
|  | GGA3 |  |
|  | MINPP1 |  |
|  | TTLL3 |  |
|  | TXNRD1 |  |
|  | STX5 |  |
|  | LOC100132077 |  |
|  | TPT1 |  |
|  | C1GALT1C1 |  |
|  | HIST1H2AK |  |
|  | PLD2 |  |
|  | FBXL17 |  |
|  | SAP30L |  |
|  | DNAJC16 |  |
|  | ICMT |  |
|  | WDYHV1 |  |
|  | FSD1L |  |
|  | DUSP7 |  |
|  | GTF2E1 |  |
|  | OTTHUMG00000154081 |  |
|  | RMDN2 |  |
|  | SNN |  |
|  | DDX56 |  |
|  | PXN |  |
|  | EVX2 | down |
|  | DZIP1 |  |
|  | CRADD |  |
|  | LOC100133669 |  |
|  | FRK |  |
|  | PREX2 |  |
|  | TSPAN15 |  |
|  | RNA5SP144 |  |
|  | OTTHUMG00000154493 |  |
|  | LOC101059957 |  |
|  | OTTHUMG00000152729 |  |
|  | OTTHUMG00000152615 |  |
|  | C1QTNF7 |  |
|  | ATP6V0D2 |  |
|  | TPD52L1 |  |
|  | OTTHUMG00000155382 |  |
|  | LOC100506011 |  |
|  | PTH1R |  |
|  | OTTHUMG00000156985 |  |
|  | OTTHUMG00000164447 |  |
|  | MIR3162 |  |
|  | OTTHUMG00000016997 |  |
|  | OTTHUMG00000160946 |  |
|  | LOC100505636 |  |
|  | VANGL2 |  |
|  | PPFIA3 |  |
|  | PLCL1 |  |
|  | SYT13 |  |
|  | KIAA1257 |  |
|  | NLRP13 |  |
|  | OTTHUMG00000013953 |  |
|  | MIR548Z |  |
|  | OTTHUMG00000163496 |  |
|  | GYS2 |  |
|  | ZNF783 |  |
|  | ATP8B5P |  |
|  | UBE2U |  |
|  | ZNF575 |  |
|  | SSPN |  |
|  | OTTHUMG00000017520 |  |
|  | FAM135B |  |
|  | NR2F1-AS1 |  |
|  | RHPN1 |  |
|  | INTU |  |
|  | BNC2 |  |
|  | FAM115C |  |
|  | SLC25A21 |  |
|  | SAMD11 |  |
|  | SPAG11B |  |
|  | SLC24A5 |  |
|  | OTTHUMG00000162814 |  |
|  | OTTHUMG00000161775 |  |
|  | LYPD6B |  |
|  | NTHL1 |  |
|  | PITPNM2 |  |
|  | OTTHUMG00000155403 |  |
|  | OTTHUMG00000163741 |  |
|  | EVA1A |  |
|  | C11orf35 |  |
|  | LINC00910 |  |
|  | CREB3L4 |  |
|  | RLBP1 |  |
|  | LOC100506497 |  |
|  | C9orf135 |  |
|  | OTTHUMG00000016526 |  |
|  | LTBP3 |  |
|  | OTTHUMG00000037132 |  |
|  | PROX1 |  |
|  | RSU1P2 |  |
|  | OTTHUMG00000035180 |  |
|  | ANKRD20A4 |  |
|  | OTTHUMG00000015781 |  |
|  | DNAH7 |  |
|  | SRPK3 |  |
|  | EN2 |  |
|  | CRB1 |  |
|  | SLC27A6 |  |
|  | SLC39A12 |  |
|  | APOL4 |  |
|  | NREP-AS1 |  |
|  | PAH |  |
|  | OTTHUMG00000020080 |  |
|  | ATP8A2 |  |
|  | HDC |  |
|  | OTTHUMG00000164544 |  |
|  | HSD11B2 |  |
|  | REEP6 |  |
|  | OTTHUMG00000008994 |  |
|  | OTTHUMG00000078698 |  |
|  | OTTHUMG00000161346 |  |
|  | HSPA12A |  |
|  | COBL |  |
|  | PPP4R4 |  |
|  | SOGA2 |  |
|  | B4GALT4-AS1 |  |
|  | LOC257358 |  |
|  | TCEAL6 |  |
|  | SNTG1 |  |
|  | RGSL1 |  |
|  | ZNF479 |  |
|  | LRRC66 |  |
|  | HS3ST5 |  |
|  | PLIN1 |  |
|  | OTTHUMG00000154962 |  |
|  | FRMPD1 |  |
|  | RNF180 |  |
|  | DNM3-IT1 |  |
|  | FAM19A4 |  |
|  | MRAP2 |  |
|  | STXBP6 |  |
|  | BMP5 |  |
|  | OTTHUMG00000163006 |  |
|  | OTTHUMG00000159469 |  |
|  | NWD1 |  |
|  | SPARCL1 |  |
|  | PLAC1L |  |
|  | TRAIP |  |
|  | OTTHUMG00000164543 |  |
|  | LRRC56 |  |
|  | SYT10 |  |
|  | ADAM7 |  |
|  | RECQL4 |  |
|  | ZNF90 |  |
|  | RHBDL1 |  |
|  | OTTHUMG00000164202 |  |
|  | OTTHUMG00000162796 |  |
|  | PHOX2A |  |
|  | OTTHUMG00000019035 |  |
|  | CCDC42B |  |
|  | HKDC1 |  |
|  | GABRG1 |  |
|  | BCL2L14 |  |
|  | ZNF683 |  |
|  | RNA5SP305 |  |
|  | C12orf71 |  |
|  | TTC40 |  |
|  | LOC340017 |  |
|  | FREM3 |  |
|  | ZBTB22 |  |
|  | CA3 |  |
|  | EPHB6 |  |
|  | ITGA10 |  |
|  | GPR161 |  |
|  | OTTHUMG00000074032 |  |
|  | RNF215 |  |
|  | TMPRSS11A |  |
|  | OTTHUMG00000160241 |  |
|  | LOC100144602 |  |
|  | C17orf77 |  |
|  | OTTHUMG00000163340 |  |
|  | OTTHUMG00000160611 |  |
|  | OVCH2 |  |
|  | MIR181A1 |  |
|  | OTTHUMG00000008404 |  |
|  | AFM |  |
|  | MAGEL2 |  |
|  | CDC42EP1 |  |
|  | C15orf43 |  |
|  | OTTHUMG00000152926 |  |
|  | OTTHUMG00000160709 |  |
|  | OTTHUMG00000018331 |  |
|  | C15orf27 |  |
|  | INHBA |  |
|  | KANK1 |  |
|  | OR51B2 |  |
|  | PTGER3 |  |
|  | OTTHUMG00000164506 |  |
|  | LOC100507435 |  |
|  | LOC151121 |  |
|  | OTTHUMG00000153449 |  |
|  | SYT12 |  |
|  | OTTHUMG00000155412 |  |
|  | EPHA7 |  |
|  | BMP10 |  |
|  | MMP26 |  |
|  | SBF2-AS1 |  |
|  | GPR39 |  |
|  | OTTHUMG00000018286 |  |
|  | OTTHUMG00000163689 |  |
|  | OTTHUMG00000164258 |  |
|  | CETN1 |  |
|  | C14orf182 |  |
|  | C19orf80 |  |
|  | ARMCX2 |  |
|  | LMO3 |  |
|  | B4GALNT2 |  |
|  | NDST4 |  |
|  | ANKRD20A1 |  |
|  | OTTHUMG00000058519 |  |
|  | DZIP1L |  |
|  | OTTHUMG00000000769 |  |
|  | ROBO3 |  |
|  | OTTHUMG00000153820 |  |
|  | LRIG3 |  |
|  | NXPH4 |  |
|  | FRMD4A |  |
|  | SPEF1 |  |
|  | PMCHL1 |  |
|  | SLC26A4-AS1 |  |
|  | OTTHUMG00000153045 |  |
|  | GALNTL5 |  |
|  | GPC2 |  |
|  | SDR42E1 |  |
|  | CXCL1 |  |
|  | LINC00443 |  |
|  | DCD |  |
|  | LINC00474 |  |
|  | OTTHUMG00000152520 |  |
|  | AXDND1 |  |
|  | OTTHUMG00000153300 |  |
|  | ROS1 |  |
|  | LINC00240 |  |
|  | ZNF311 |  |
|  | ABRA |  |
|  | SLC22A9 |  |
|  | EXOC3L4 |  |
|  | WARS2-IT1 |  |
|  | TEX36 |  |
|  | ADORA2A |  |
|  | NPR3 |  |
|  | CFL1P1 |  |
|  | MIR4276 |  |
|  | CCDC80 |  |
|  | OTTHUMG00000160615 |  |
|  | C5orf17 |  |
|  | OTTHUMG00000018307 |  |
|  | OTTHUMG00000160407 |  |
|  | GPR116 |  |
|  | OTTHUMG00000020547 |  |
|  | LARS2-AS1 |  |
|  | HTN1 |  |
|  | OTTHUMG00000162825 |  |
|  | OTTHUMG00000012284 |  |
|  | ANO4 |  |
|  | DLL1 |  |
|  | C8orf34 |  |
|  | OTTHUMG00000021246 |  |
|  | CBX2 |  |
|  | OTTHUMG00000016536 |  |
|  | OTTHUMG00000162104 |  |
|  | SCGN |  |
|  | NKAIN2 |  |
|  | TMEM220 |  |
|  | SLC28A3 |  |
|  | ZNF608 |  |
|  | SHF |  |
|  | IGF1 |  |
|  | LOC100507205 |  |
|  | AEBP1 |  |
|  | ASTN2 |  |
|  | FBXO39 |  |
|  | FRMD6-AS2 |  |
|  | OTTHUMG00000152939 |  |
|  | EYS |  |
|  | OTTHUMG00000153912 |  |
|  | SPATA9 |  |
|  | PRPS1L1 |  |
|  | IQCC |  |
|  | CABS1 |  |
|  | TMPRSS15 |  |
|  | GTSF1L |  |
|  | LOC100506551 |  |
|  | DCUN1D2-AS1 |  |
|  | HBG2 |  |
|  | GNRHR |  |
|  | C3orf67 |  |
|  | OTTHUMG00000158300 |  |
|  | COLGALT2 |  |
|  | PLK5 |  |
|  | OTTHUMG00000151730 |  |
|  | OTTHUMG00000159007 |  |
|  | PEX5L |  |
|  | PTGIR |  |
|  | C3orf55 |  |
|  | OTTHUMG00000159058 |  |
|  | OTTHUMG00000162040 |  |
|  | TRIM49D2P |  |
|  | HMGA1P1 |  |
|  | OTOF |  |
|  | CNTN3 |  |
|  | OTTHUMG00000019542 |  |
|  | CDC20 |  |
|  | GNG4 |  |
|  | OTTHUMG00000158567 |  |
|  | LINC00332 |  |
|  | DNAH17 |  |
|  | CALB2 |  |
|  | FAT3 |  |
|  | OTTHUMG00000162871 |  |
|  | OTTHUMG00000140137 |  |
|  | OTTHUMG00000159106 |  |
|  | UNC5C |  |
|  | THBS4 |  |
|  | KLB |  |
|  | OTTHUMG00000014826 |  |
|  | SLC25A41 |  |
|  | OTTHUMG00000160643 |  |
|  | OTTHUMG00000015244 |  |
|  | OTTHUMG00000165057 |  |
|  | RNA5SP287 |  |
|  | OTTHUMG00000043582 |  |
|  | LIMCH1 |  |
|  | UTS2B |  |
|  | ACE |  |
|  | OGDHL |  |
|  | KIAA1462 |  |
|  | MIR132 |  |
|  | STOML3 |  |
|  | MOK |  |
|  | MISP |  |
|  | GPR128 |  |
|  | HYAL4 |  |
|  | LOC283332 |  |
|  | CD163L1 |  |
|  | CRISP3 |  |
|  | CTSF |  |
|  | OTTHUMG00000001907 |  |
|  | PCDH9 |  |
|  | OTTHUMG00000022220 |  |
|  | TJP1 |  |
|  | CTAGE7P |  |
|  | OTTHUMG00000157310 |  |
|  | OSM |  |
|  | RNA5SP425 |  |
|  | SPA17 |  |
|  | RNA5SP443 |  |
|  | DNAH11 |  |
|  | ANKRD20A3 |  |
|  | CCDC158 |  |
|  | NPY1R |  |
|  | OTTHUMG00000160185 |  |
|  | HSD17B8 |  |
|  | OTTHUMG00000159738 |  |
|  | TMEM59L |  |
|  | SKP1P2 |  |
|  | CHRM3-AS1 |  |
|  | TMUB1 |  |
|  | OTTHUMG00000156483 |  |
|  | LOC200726 |  |
|  | ME3 |  |
|  | OTTHUMG00000162807 |  |
|  | RHPN2 |  |
|  | LOC647323 |  |
|  | LINC00272 |  |
|  | SLC9A2 |  |
|  | MIR3928 |  |
|  | LEFTY1 |  |
|  | CASC2 |  |
|  | CNTN4 |  |
|  | TMC1 |  |
|  | OTTHUMG00000152350 |  |
|  | TULP1 |  |
|  | KCND2 |  |
|  | GPR98 |  |
|  | GNRHR2 |  |
|  | OTTHUMG00000159221 |  |
|  | FAM83H |  |
|  | ABCA9-AS1 |  |
|  | OSTN |  |
|  | ACOT12 |  |
|  | OTTHUMG00000163520 |  |
|  | DGAT2L6 |  |
|  | FAM196B |  |
|  | C6orf201 |  |
|  | OTTHUMG00000009216 |  |
|  | RNU6-56P |  |
|  | KSR2 |  |
|  | LOC338817 |  |
|  | TTC12 |  |
|  | OTTHUMG00000162203 |  |
|  | ICAM5 |  |
|  | CDH2 |  |
|  | SPTBN4 |  |
|  | SDC2 |  |
|  | RNA5SP140 |  |
|  | ZWINT |  |
|  | KCNS3 |  |
|  | OTTHUMG00000155112 |  |
|  | LOC100507330 |  |
|  | C9 |  |
|  | FSD1 |  |
|  | MDK |  |
|  | POM121L12 |  |
|  | AMTN |  |
|  | SPATA16 |  |
|  | AGXT2 |  |
|  | LOC100133207 |  |
|  | OTTHUMG00000014297 |  |
|  | OTTHUMG00000162740 |  |
|  | UNC13A |  |
|  | MPP4 |  |
|  | CAV2 |  |
|  | PLA2R1 |  |
|  | SLC45A2 |  |
|  | OR6A2 |  |
|  | ZNF662 |  |
|  | OSMR |  |
|  | SH2D3A |  |
|  | LRRC17 |  |
|  | CPA3 |  |
|  | FAM5C |  |
|  | OTTHUMG00000155122 |  |
|  | RNA5SP204 |  |
|  | PPP1R17 |  |
|  | TPSAB1 |  |
|  | CPN1 |  |
|  | MIR320B1 |  |
|  | KDR |  |
|  | FLJ44006 |  |
|  | APOA4 |  |
|  | KIF1A |  |
|  | KIAA1161 |  |
|  | SCN11A |  |
|  | CEP170B |  |
|  | OTTHUMG00000162214 |  |
|  | OTTHUMG00000151480 |  |
|  | TECTB |  |
|  | LOC100128787 |  |
|  | OTTHUMG00000155409 |  |
|  | FNDC1 |  |
|  | PACSIN3 |  |
|  | TMEM108 |  |
|  | PCSK5 |  |
|  | WDR62 |  |
|  | GNG13 |  |
|  | KCNE4 |  |
|  | OSBP2 |  |
|  | YAP1 |  |
|  | MIR3659 |  |
|  | TRIM54 |  |
|  | OTTHUMG00000015548 |  |
|  | BOK |  |
|  | AIM1L |  |
|  | MYRF |  |
|  | MIR1182 |  |
|  | PSAT1P4 |  |
|  | PDE2A |  |
|  | PAX9 |  |
|  | OTTHUMG00000002836 |  |
|  | CACNA1G |  |
|  | RND3 |  |
|  | OTTHUMG00000154852 |  |
|  | OTTHUMG00000014960 |  |
|  | LUM |  |
|  | PMEPA1 |  |
|  | HEPH |  |
|  | TPH2 |  |
|  | ESRRG |  |
|  | FLNC |  |
|  | NR2E1 |  |
|  | NKX2-6 |  |
|  | LINC00867 |  |
|  | RGL3 |  |
|  | PFN3 |  |
|  | CCDC85A |  |
|  | OTUD7A |  |
|  | G0S2 |  |
|  | TUSC7 |  |
|  | TNNT1 |  |
|  | CGN |  |
|  | LINC00364 |  |
|  | BATF2 |  |
|  | PVRIG |  |
|  | MAPK10 |  |
|  | RNA5SP394 |  |
|  | OTTHUMG00000150989 |  |
|  | CNTD2 |  |
|  | OTTHUMG00000153712 |  |
|  | ADAM5 |  |
|  | NINL |  |
|  | SRRM3 |  |
|  | ENTPD2 |  |
|  | C8orf48 |  |
|  | DDX39B-AS1 |  |
|  | OTTHUMG00000137359 |  |
|  | MYO1A |  |
|  | GPR115 |  |
|  | LOC100129046 |  |
|  | KGFLP1 |  |
|  | OTTHUMG00000157235 |  |
|  | OR2B3 |  |
|  | KRT19 |  |
|  | TDRD12 |  |
|  | PPP1R14D |  |
|  | FNDC5 |  |
|  | DPYSL4 |  |
|  | HCG9 |  |
|  | DLL3 |  |
|  | LINC00491 |  |
|  | FAM66C |  |
|  | LINC00359 |  |
|  | HS3ST6 |  |
|  | FAM198A |  |
|  | ROBO1 |  |
|  | LOC100996583 |  |
|  | RNU5F-4P |  |
|  | C14orf105 |  |
|  | LZTS2 |  |
|  | BHLHB9 |  |
|  | OTTHUMG00000067435 |  |
|  | OTTHUMG00000010912 |  |
|  | OTTHUMG00000152656 |  |
|  | OTTHUMG00000162354 |  |
|  | OTTHUMG00000165097 |  |
|  | TAS2R1 |  |
|  | RLN1 |  |
|  | OTTHUMG00000151766 |  |
|  | OTTHUMG00000159877 |  |
|  | CDH19 |  |
|  | RNU6-71P |  |
|  | DSG1 |  |
|  | OR4K1 |  |
|  | OTTHUMG00000153097 |  |
|  | OTTHUMG00000003039 |  |
|  | RNA5SP456 |  |
|  | MYO1H |  |
|  | ARMC4 |  |
|  | LOC728755 |  |
|  | SLC25A23 |  |
|  | OTTHUMG00000078687 |  |
|  | SPINK5 |  |
|  | CCDC136 |  |
|  | CCDC11 |  |
|  | TUFT1 |  |
|  | PAK3 |  |
|  | OTTHUMG00000020560 |  |
|  | OTTHUMG00000043574 |  |
|  | KIAA1210 |  |
|  | NR1I2 |  |
|  | OTTHUMG00000161728 |  |
|  | ZNRF4 |  |
|  | LOC100130502 |  |
|  | CXXC1P1 |  |
|  | LOC154761 |  |
|  | LOC100506274 |  |
|  | AMPH |  |
|  | OTTHUMG00000151625 |  |
|  | CALN1 |  |
|  | PLEKHG6 |  |
|  | OTTHUMG00000159017 |  |
|  | TMEM132B |  |
|  | ZIC1 |  |
|  | DDC |  |
|  | PROCR |  |
|  | NT5C1B |  |
|  | LINC00971 |  |
|  | SEMA3D |  |
|  | IFNA4 |  |
|  | FGF3 |  |
|  | OTTHUMG00000155267 |  |
|  | LOC100507108 |  |
|  | EID3 |  |
|  | LSAMP-AS1 |  |
|  | OTTHUMG00000161483 |  |
|  | PRPH2 |  |
|  | PCP2 |  |
|  | MIR1276 |  |
|  | NGF |  |
|  | RXFP2 |  |
|  | SH3RF2 |  |
|  | LOC100996444 |  |
|  | ETV1 |  |
|  | LURAP1L |  |
|  | LOC441009 |  |
|  | OTTHUMG00000158739 |  |
|  | OTTHUMG00000153857 |  |
|  | TTC28 |  |
|  | CCDC154 |  |
|  | C22orf23 |  |
|  | SMIM2-IT1 |  |
|  | C8orf22 |  |
|  | TSG1 |  |
|  | CERS3 |  |
|  | LINC00457 |  |
|  | OTTHUMG00000158224 |  |
|  | AKAP12 |  |
|  | TAS2R40 |  |
|  | C19orf71 |  |
|  | IL36RN |  |
|  | WWTR1 |  |
|  | OTTHUMG00000035258 |  |
|  | CCDC147-AS1 |  |
|  | DCST2 |  |
|  | CASKIN2 |  |
|  | OTTHUMG00000163316 |  |
|  | LOC340107 |  |
|  | GPAT2 |  |
|  | C15orf52 |  |
|  | LINC00498 |  |
|  | OTTHUMG00000017571 |  |
|  | LOC100506993 |  |
|  | CHRM5 |  |
|  | DCLK3 |  |
|  | RPLP0P2 |  |
|  | OTTHUMG00000153714 |  |
|  | OTTHUMG00000162452 |  |
|  | OTTHUMG00000161787 |  |
|  | PDZD7 |  |
|  | RAPSN |  |
|  | ZNF804B |  |
|  | LOC440981 |  |
|  | OTTHUMG00000161776 |  |
|  | LOC221122 |  |
|  | RNA5SP510 |  |
|  | SNX31 |  |
|  | OTTHUMG00000163453 |  |
|  | LOC100506241 |  |
|  | RN7SKP10 |  |
|  | PCDH17 |  |
|  | LOC100506389 |  |
|  | OTTHUMG00000154544 |  |
|  | OTTHUMG00000020972 |  |
|  | GLYATL2 |  |
|  | C17orf47 |  |
|  | OTTHUMG00000014883 |  |
|  | OTTHUMG00000013198 |  |
|  | AASS |  |
|  | CPNE7 |  |
|  | C6orf58 |  |
|  | OTTHUMG00000019202 |  |
|  | C10orf107 |  |
|  | TRBV27 |  |
|  | MMP7 |  |
|  | OTTHUMG00000164063 |  |
|  | EFCAB8 |  |
|  | OTTHUMG00000153084 |  |
|  | MAPT |  |
|  | FAM227A |  |
|  | OTTHUMG00000160217 |  |
|  | OTTHUMG00000017144 |  |
|  | ATHL1 |  |
|  | OTTHUMG00000059449 |  |
|  | SGK223 |  |
|  | IL17D |  |
|  | LINC00290 |  |
|  | SYNPR |  |
|  | WT1 |  |
|  | ITLN2 |  |
|  | OTTHUMG00000160683 |  |
|  | OR5T3 |  |
|  | CLDN20 |  |
|  | RNF183 |  |
|  | ENPP3 |  |
|  | LUZP2 |  |
|  | OTTHUMG00000161871 |  |
|  | OTTHUMG00000018252 |  |
|  | APOA2 |  |
|  | OTTHUMG00000164414 |  |
|  | LOC100507487 |  |
|  | MIR548D1 |  |
|  | CLCA2 |  |
|  | ONECUT2 |  |
|  | LOXL4 |  |
|  | OTTHUMG00000164513 |  |
|  | OTTHUMG00000153781 |  |
|  | KRTAP21-1 |  |
|  | DIAPH2-AS1 |  |
|  | ZSCAN10 |  |
|  | OTTHUMG00000035769 |  |
|  | LOC100996456 |  |
|  | MYH10 |  |
|  | PHLDA3 |  |
|  | OTTHUMG00000153426 |  |
|  | CDCP1 |  |
|  | DMP1 |  |
|  | OTTHUMG00000163341 |  |
|  | MGP |  |
|  | C20orf26 |  |
|  | LINC00470 |  |
|  | TMEM78 |  |
|  | GTF2IRD1 |  |
|  | EPHA3 |  |
|  | OTTHUMG00000132778 |  |
|  | PNLIPRP1 |  |
|  | MTTP |  |
|  | EFEMP1 |  |
|  | PABPC1P2 |  |
|  | ZNF215 |  |
|  | FERMT1 |  |
|  | ADCY2 |  |
|  | LINC00282 |  |
|  | CNKSR3 |  |
|  | B3GNT3 |  |
|  | F12 |  |
|  | ZNF454 |  |
|  | C4orf22 |  |
|  | RNA5SP164 |  |
|  | OTTHUMG00000018278 |  |
|  | DLG2 |  |
|  | LOC101060091 |  |
|  | VENTXP1 |  |
|  | PLA1A |  |
|  | PTGES2-AS1 |  |
|  | C2orf40 |  |
|  | PINLYP |  |
|  | LGI1 |  |
|  | GAD1 |  |
|  | LOC100134259 |  |
|  | TTTY10 |  |
|  | SPAG4 |  |
|  | OTTHUMG00000160297 |  |
|  | APOBEC4 |  |
|  | KIAA1211 |  |
|  | OTTHUMG00000017963 |  |
|  | HORMAD2 |  |
|  | LEPREL2 |  |
|  | TUB |  |
|  | LINC00202-1 |  |
|  | IFNA8 |  |
|  | OTTHUMG00000041127 |  |
|  | N6AMT1 |  |
|  | MEPE |  |
|  | LOC149086 |  |
|  | PDIA2 |  |
|  | RSPO2 |  |
|  | OTTHUMG00000152593 |  |
|  | LINC00340 |  |
|  | SVOPL |  |
|  | OTTHUMG00000020459 |  |
|  | SLC22A31 |  |
|  | ABCA13 |  |
|  | COL6A3 |  |
|  | OTTHUMG00000018458 |  |
|  | PTPRU |  |
|  | OTTHUMG00000161603 |  |
|  | BASP1P1 |  |
|  | OTTHUMG00000161810 |  |
|  | FAM69C |  |
|  | C20orf144 |  |
|  | OTTHUMG00000163449 |  |
|  | OR2F1 |  |
|  | OTTHUMG00000161344 |  |
|  | OTTHUMG00000020281 |  |
|  | TTTY12 |  |
|  | C17orf112 |  |
|  | OTTHUMG00000017608 |  |
|  | SPINK8 |  |
|  | OTTHUMG00000163486 |  |
|  | LMTK3 |  |
|  | LOC440335 |  |
|  | FLJ46010 |  |
|  | LINC00477 |  |
|  | CYP19A1 |  |
|  | RBPMS |  |
|  | PDLIM4 |  |
|  | DTX1 |  |
|  | OTTHUMG00000160909 |  |
|  | C1orf61 |  |
|  | PCDH15 |  |
|  | ARHGAP40 |  |
|  | EFHD1 |  |
|  | MOBP |  |
|  | KCNAB1-AS1 |  |
|  | LOC407835 |  |
|  | EPHA5 |  |
|  | SLC13A3 |  |
|  | MMP10 |  |
|  | FAM183CP |  |
|  | HAO2 |  |
|  | FLJ31662 |  |
|  | WFDC8 |  |
|  | LOC440040 |  |
|  | HRG |  |
|  | DEFA6 |  |
|  | OTTHUMG00000161916 |  |
|  | MIR548F3 |  |
|  | RNF148 |  |
|  | PAX8 |  |
|  | PHACTR3 |  |
|  | OTTHUMG00000163879 |  |
|  | SLC35F4 |  |
|  | PTPRB |  |
|  | KAL1 |  |
|  | FREM1 |  |
|  | OTTHUMG00000162462 |  |
|  | LOC100507513 |  |
|  | ZNF503-AS1 |  |
|  | OTTHUMG00000161177 |  |
|  | TPST1 |  |
|  | TSPAN16 |  |
|  | LOC100996465 |  |
|  | ZNF620 |  |
|  | OTTHUMG00000037303 |  |
|  | OTTHUMG00000161212 |  |
|  | CACNB2 |  |
|  | AQP4-AS1 |  |
|  | PNPLA3 |  |
|  | AKAP6 |  |
|  | OTTHUMG00000154207 |  |
|  | NEURL1B |  |
|  | PRSS12 |  |
|  | OTTHUMG00000154769 |  |
|  | OTTHUMG00000157169 |  |
|  | OTTHUMG00000161531 |  |
|  | OTTHUMG00000160350 |  |
|  | SHANK1 |  |
|  | OTTHUMG00000163633 |  |
|  | OTTHUMG00000021210 |  |
|  | CCHCR1 |  |
|  | GHR |  |
|  | ZIM3 |  |
|  | OTTHUMG00000159392 |  |
|  | PP2D1 |  |
|  | STAP2 |  |
|  | IL1RL2 |  |
|  | MAS1 |  |
|  | OTTHUMG00000161118 |  |
|  | OTTHUMG00000150676 |  |
|  | OTTHUMG00000014350 |  |
|  | NELL1 |  |
|  | LOC284395 |  |
|  | LOC100129297 |  |
|  | CCDC179 |  |
|  | CHST9 |  |
|  | OTTHUMG00000161178 |  |
|  | OTTHUMG00000156953 |  |
|  | OTTHUMG00000034564 |  |
|  | MIR490 |  |
|  | LRRC7 |  |
|  | PHF2P2 |  |
|  | PCDH7 |  |
|  | LOC731779 |  |
|  | MIR3124 |  |
|  | ZNF320 |  |
|  | AOX1 |  |
|  | FOXP2 |  |
|  | OTTHUMG00000024178 |  |
|  | OTTHUMG00000159742 |  |
|  | APOH |  |
|  | TOB1-AS1 |  |
|  | LOC100128593 |  |
|  | OTTHUMG00000134301 |  |
|  | CA10 |  |
|  | FTLP10 |  |
|  | EPHA6 |  |
|  | CCDC147 |  |
|  | ETV2 |  |
|  | PATE1 |  |
|  | OTTHUMG00000161394 |  |
|  | OTTHUMG00000015760 |  |
|  | FLJ30838 |  |
|  | TG |  |
|  | LOC100131691 |  |
|  | LIFR-AS1 |  |
|  | LOXL1 |  |
|  | SLCO5A1 |  |
|  | LOC100506134 |  |
|  | OTTHUMG00000164885 |  |
|  | KLHL29 |  |
|  | MYH7 |  |
|  | LOC100505918 |  |
|  | ANKRD29 |  |
|  | IL9R |  |
|  | GDF2 |  |
|  | OTTHUMG00000035451 |  |
|  | OTTHUMG00000016711 |  |
|  | STAC |  |
|  | HRK |  |
|  | PTCHD1 |  |
|  | MPPED1 |  |
|  | HCRTR2 |  |
|  | H2BFXP |  |
|  | HTRA3 |  |
|  | SLC16A14 |  |
|  | GALNT14 |  |
|  | KRTAP19-6 |  |
|  | C2orf83 |  |
|  | MIR492 |  |
|  | LRP1B |  |
|  | JPH2 |  |
|  | OTTHUMG00000152498 |  |
|  | GSG1L |  |
|  | XKR9 |  |
|  | PHLDB3 |  |
|  | OTTHUMG00000162533 |  |
|  | LOC100288911 |  |
|  | P4HA3 |  |
|  | OTTHUMG00000164832 |  |
|  | OTTHUMG00000037935 |  |
|  | PGAM1P5 |  |
|  | SUMO1P1 |  |
|  | SRRM4 |  |
|  | OTTHUMG00000015092 |  |
|  | LINC00238 |  |
|  | PLEK2 |  |
|  | MORF4L2-AS1 |  |
|  | KRT71 |  |
|  | MIR548AI |  |
|  | STAR |  |
|  | LOC100506085 |  |
|  | SOX6 |  |
|  | CCDC36 |  |
|  | TRPM8 |  |
|  | DHRS11 |  |
|  | OTTHUMG00000074396 |  |
|  | AMZ1 |  |
|  | OR7G1 |  |
|  | CELA1 |  |
|  | AGRN |  |
|  | CHRNA9 |  |
|  | GC |  |
|  | LOC100507291 |  |
|  | OTTHUMG00000019705 |  |
|  | SNAR-E |  |
|  | SRD5A3-AS1 |  |
|  | UGGT2-IT1 |  |
|  | FAM220BP |  |
|  | OTTHUMG00000022289 |  |
|  | SLC7A9 |  |
|  | HMCN1 |  |
|  | DRC1 |  |
|  | CYP27B1 |  |
|  | RAB3B |  |
|  | TMEM229A |  |
|  | OTTHUMG00000015498 |  |
|  | OTTHUMG00000158360 |  |
|  | C11orf88 |  |
|  | LINC00588 |  |
|  | FRMPD4 |  |
|  | AP1B1P1 |  |
|  | C2orf82 |  |
|  | PTCH2 |  |
|  | FMO9P |  |
|  | CTNND2 |  |
|  | LOC100286925 |  |
|  | OTTHUMG00000017273 |  |
|  | FAM186B |  |
|  | PCGEM1 |  |
|  | PDZRN4 |  |
|  | KRTAP19-5 |  |
|  | ASAH2B |  |
|  | WDR72 |  |
|  | KHK |  |
|  | KANK3 |  |
|  | EPPIN |  |
|  | ANKRD2 |  |
|  | GRIFIN |  |
|  | MIR4791 |  |
|  | OTTHUMG00000019462 |  |
|  | AP3B2 |  |
|  | UPK1A-AS1 |  |
|  | RAB3IL1 |  |
|  | LINC00114 |  |
|  | OTTHUMG00000150105 |  |
|  | APOF |  |
|  | OTTHUMG00000163729 |  |
|  | MIR4305 |  |
|  | PRSS1 |  |
|  | OTTHUMG00000019427 |  |
|  | C3orf27 |  |
|  | LOC100288123 |  |
|  | SLC30A10 |  |
|  | C19orf45 |  |
|  | OTTHUMG00000019016 |  |
|  | OTTHUMG00000010865 |  |
|  | C1R |  |
|  | RETNLB |  |
|  | XAGE2B |  |
|  | PPT2 |  |
|  | ASB11 |  |
|  | OTTHUMG00000086644 |  |
|  | OTTHUMG00000020194 |  |
|  | OTTHUMG00000155475 |  |
|  | PIPSL |  |
|  | LINC00880 |  |
|  | OTTHUMG00000153807 |  |
|  | CDK20 |  |
|  | MYO15A |  |
|  | A1BG |  |
|  | TCF7L1 |  |
|  | GPC6 |  |
|  | MAGI2 |  |
|  | HAS2 |  |
|  | RUFY4 |  |
|  | PSD |  |
|  | OTTHUMG00000037449 |  |
|  | FIGLA |  |
|  | OTTHUMG00000160780 |  |
|  | OTTHUMG00000162877 |  |
|  | NEIL2 |  |
|  | DSP |  |
|  | OTTHUMG00000155428 |  |
|  | SLCO1C1 |  |
|  | EFCAB4A |  |
|  | MPP2 |  |
|  | F11 |  |
|  | SLC17A8 |  |
|  | OTTHUMG00000019116 |  |
|  | CDO1 |  |
|  | OR8J1 |  |
|  | NAV2-AS4 |  |
|  | C22orf26 |  |
|  | LINC00293 |  |
|  | KRT80 |  |
|  | OTTHUMG00000152138 |  |
|  | CD200 |  |
|  | NPSR1-AS1 |  |
|  | SLC39A4 |  |
|  | FAM194B |  |
|  | OTTHUMG00000164031 |  |
|  | OTTHUMG00000039869 |  |
|  | OTTHUMG00000019970 |  |
|  | LOC100506271 |  |
|  | OTTHUMG00000162145 |  |
|  | OTTHUMG00000040853 |  |
|  | ADAMTS9 |  |
|  | FBXO16 |  |
|  | SH2D5 |  |
|  | GK2 |  |
|  | LOC253573 |  |
|  | SFRP4 |  |
|  | TRBV23OR9-2 |  |
|  | NRSN1 |  |
|  | RNA5SP476 |  |
|  | GGT8P |  |
|  | SLC26A3 |  |
|  | ATP13A5 |  |
|  | LINC00644 |  |
|  | ARG1 |  |
|  | GGN |  |
|  | OTTHUMG00000133693 |  |
|  | LOC653501 |  |
|  | OTTHUMG00000162296 |  |
|  | OTTHUMG00000164616 |  |
|  | TEX41 |  |
|  | TMPRSS9 |  |
|  | LOC400756 |  |
|  | IGHV3-13 |  |
|  | PRCD |  |
|  | LOC401463 |  |
|  | OTTHUMG00000163434 |  |
|  | CCDC116 |  |
|  | TCEAL7 |  |
|  | OTC |  |
|  | DES |  |
|  | ITIH5 |  |
|  | OTTHUMG00000132315 |  |
|  | ADCY6 |  |
|  | AQPEP |  |
|  | ITIH3 |  |
|  | OTTHUMG00000010107 |  |
|  | OTTHUMG00000161417 |  |
|  | TXNRD3NB |  |
|  | KPNA7 |  |
|  | ZNF385B |  |
|  | TMPRSS11D |  |
|  | PRODH |  |
|  | NR1H4 |  |
|  | C1orf228 |  |
|  | ZNF471 |  |
|  | ASB5 |  |
|  | OTTHUMG00000017866 |  |
|  | TLN2 |  |
|  | SPO11 |  |
|  | CPLX2 |  |
|  | HAO1 |  |
|  | OTTHUMG00000017241 |  |
|  | OTTHUMG00000154303 |  |
|  | ERG |  |
|  | ZNF579 |  |
|  | ISYNA1 |  |
|  | PEG3 |  |
|  | CNTNAP4 |  |
|  | TPTE |  |
|  | CARD14 |  |
|  | CCNYL3 |  |
|  | TTC34 |  |
|  | OTTHUMG00000019532 |  |
|  | MEGF6 |  |
|  | C3orf20 |  |
|  | MIR4275 |  |
|  | OTTHUMG00000164339 |  |
|  | ARHGEF28 |  |
|  | TNNT2 |  |
|  | SEC1P |  |
|  | OTTHUMG00000018616 |  |
|  | OTTHUMG00000152890 |  |
|  | PRKX-AS1 |  |
|  | OTTHUMG00000017910 |  |
|  | LINC00536 |  |
|  | MIR4468 |  |
|  | ECEL1 |  |
|  | LOC100506834 |  |
|  | SPATA8-AS1 |  |
|  | OTTHUMG00000152879 |  |
|  | FLJ34503 |  |
|  | LOC100506122 |  |
|  | BEST3 |  |
|  | C6orf99 |  |
|  | OTTHUMG00000163611 |  |
|  | CLDN18 |  |
|  | FBXL2 |  |
|  | TTBK1 |  |
|  | NRG2 |  |
|  | IP6K3 |  |
|  | CAMK2N2 |  |
|  | OTTHUMG00000164338 |  |
|  | OTTHUMG00000162758 |  |
|  | OTTHUMG00000074322 |  |
|  | OTTHUMG00000161225 |  |
|  | LZTS1 |  |
|  | SHROOM4 |  |
|  | PABPN1L |  |
|  | OTTHUMG00000132249 |  |
|  | SMARCA5-AS1 |  |
|  | ETNPPL |  |
|  | SPOCK1 |  |
|  | CASK-AS1 |  |
|  | OTTHUMG00000154923 |  |
|  | USHBP1 |  |
|  | TTTY1B |  |
|  | FGF12 |  |
|  | RNF182 |  |
|  | FER1L4 |  |
|  | IGHV1-69 |  |
|  | OTTHUMG00000035812 |  |
|  | CLDN1 |  |
|  | CCDC85C |  |
|  | LPHN3 |  |
|  | OTTHUMG00000009350 |  |
|  | LOC151760 |  |
|  | SH3BGR |  |
|  | OTTHUMG00000015296 |  |
|  | KIF19 |  |
|  | DSG4 |  |
|  | SPAM1 |  |
|  | TEK |  |
|  | CRTAC1 |  |
|  | OTTHUMG00000153756 |  |
|  | NOS3 |  |
|  | ADAM33 |  |
|  | CDH15 |  |
|  | EPB41L4B |  |
|  | IGFBPL1 |  |
|  | OTTHUMG00000161161 |  |
|  | MGAT5B |  |
|  | SCGB3A2 |  |
|  | LOC100507373 |  |
|  | ADPGK-AS1 |  |
|  | VSIG8 |  |
|  | SCN3B |  |
|  | OTTHUMG00000160100 |  |
|  | LCN12 |  |
|  | KRT121P |  |
|  | OTTHUMG00000161454 |  |
|  | OTTHUMG00000154232 |  |
|  | LIG1 |  |
|  | OTTHUMG00000021126 |  |
|  | WDR65 |  |
|  | RPL3L |  |
|  | KIFC3 |  |
|  | FAM13C |  |
|  | OTTHUMG00000155618 |  |
|  | OTTHUMG00000162349 |  |
|  | OTTHUMG00000009931 |  |
|  | OACYLP |  |
|  | BTBD16 |  |
|  | LINC00654 |  |
|  | LOC440117 |  |
|  | OTTHUMG00000019313 |  |
|  | PRX |  |
|  | OTTHUMG00000010873 |  |
|  | OTTHUMG00000019549 |  |
|  | ABCB9 |  |
|  | GRIN2A |  |
|  | LOC375295 |  |
|  | OTTHUMG00000162429 |  |
|  | ZSCAN18 |  |
|  | LAMB3 |  |
|  | NAV2-IT1 |  |
|  | MUC17 |  |
|  | RTKN |  |
|  | OTTHUMG00000031832 |  |
|  | SLC1A2 |  |
|  | NR2E3 |  |
|  | CCL14 |  |
|  | FAM66D |  |
|  | OTTHUMG00000156059 |  |
|  | LINC00486 |  |
|  | TTTY1 |  |
|  | SPNS2 |  |
|  | CMTM8 |  |
|  | SH3GL2 |  |
|  | MIR2115 |  |
|  | VSTM2L |  |
|  | OTTHUMG00000153863 |  |
|  | OTTHUMG00000041729 |  |
|  | C1orf229 |  |
|  | CCDC3 |  |
|  | GLRA2 |  |
|  | OTTHUMG00000018530 |  |
|  | C14orf177 |  |
|  | HMGA2 |  |
|  | PRB4 |  |
|  | AMOT |  |
|  | OTTHUMG00000154818 |  |
|  | LOC100289361 |  |
|  | TRIML2 |  |
|  | TRIM50 |  |
|  | GSTT2 |  |
|  | OTTHUMG00000162098 |  |
|  | OTTHUMG00000032052 |  |
|  | LOC728012 |  |
|  | LOC100505938 |  |
|  | PIWIL1 |  |
|  | GPSM1 |  |
|  | DIAPH3-AS2 |  |
|  | MIR206 |  |
|  | OTTHUMG00000162883 |  |
|  | RNA5SP116 |  |
|  | OTTHUMG00000016683 |  |
|  | C9orf163 |  |
|  | OTTHUMG00000162998 |  |
|  | BSN-AS2 |  |
|  | NOX4 |  |
|  | OTTHUMG00000132691 |  |
|  | OTTHUMG00000018715 |  |
|  | TMEM177 |  |
|  | OTTHUMG00000163249 |  |
|  | OTTHUMG00000007731 |  |
|  | OTTHUMG00000163858 |  |
|  | XRCC6P5 |  |
|  | ADAMTS2 |  |
|  | OTTHUMG00000164459 |  |
|  | FBXW12 |  |
|  | OTTHUMG00000013065 |  |
|  | PDE11A |  |
|  | TMEM38A |  |
|  | NRG3 |  |
|  | NPM2 |  |
|  | NECAB2 |  |
|  | PRDM16 |  |
|  | OTTHUMG00000164493 |  |
|  | MYH16 |  |
|  | CBLC |  |
|  | NDNF |  |
|  | LBX2 |  |
|  | LDB2 |  |
|  | SORBS1 |  |
|  | PDE1C |  |
|  | ZNF599 |  |
|  | PROX2 |  |
|  | TENM4 |  |
|  | FAM71E2 |  |
|  | OTTHUMG00000140389 |  |
|  | HOTAIR |  |
|  | OTTHUMG00000153105 |  |
|  | FLJ37644 |  |
|  | OTTHUMG00000153335 |  |
|  | OTTHUMG00000161881 |  |
|  | PDZRN3 |  |
|  | LAMC3 |  |
|  | OTTHUMG00000132690 |  |
|  | SHISA2 |  |
|  | BCAR1 |  |
|  | ZNF540 |  |
|  | LOC100128881 |  |
|  | COL15A1 |  |
|  | FAM154B |  |
|  | C12orf74 |  |
|  | OTTHUMG00000151742 |  |
|  | C16orf82 |  |
|  | OTTHUMG00000022349 |  |
|  | OTTHUMG00000153856 |  |
|  | TRIM42 |  |
|  | EDIL3 |  |
|  | NLE1 |  |
|  | INHBE |  |
|  | FAM194A |  |
|  | EPGN |  |
|  | VGLL3 |  |
|  | CDH10 |  |
|  | MROH2A |  |
|  | MIR3197 |  |
|  | CEACAM5 |  |
|  | LHX8 |  |
|  | OTTHUMG00000015729 |  |
|  | OTTHUMG00000036253 |  |
|  | GABRA2 |  |
|  | OTTHUMG00000157659 |  |
|  | SUSD4 |  |
|  | OTTHUMG00000067508 |  |
|  | OTTHUMG00000002707 |  |
|  | OTTHUMG00000152025 |  |
|  | OTTHUMG00000017563 |  |
|  | OTTHUMG00000018345 |  |
|  | ZC3H12B |  |
|  | SLC29A2 |  |
|  | CD300LD |  |
|  | MYOZ1 |  |
|  | OTTHUMG00000162630 |  |
|  | TYR |  |
|  | UNC13C |  |
|  | VSTM5 |  |
|  | RADIL |  |
|  | AJUBA |  |
|  | FAM188B |  |
|  | KRT79 |  |
|  | OTTHUMG00000164967 |  |
|  | JOSD2 |  |
|  | TUBAL3 |  |
|  | LINC00308 |  |
|  | OTTHUMG00000165043 |  |
|  | OTTHUMG00000014286 |  |
|  | SULT1C4 |  |
|  | CYP11B2 |  |
|  | OTTHUMG00000153051 |  |
|  | AQP6 |  |
|  | PTPRF |  |
|  | OTTHUMG00000015889 |  |
|  | OTTHUMG00000162184 |  |
|  | OTTHUMG00000160487 |  |
|  | ANTXR1 |  |
|  | KRT17 |  |
|  | OTTHUMG00000017314 |  |
|  | UGT8 |  |
|  | KCNG2 |  |
|  | DYNC1I1 |  |
|  | VGLL2 |  |
|  | BFSP1 |  |
|  | OTTHUMG00000021529 |  |
|  | ZNF517 |  |
|  | ANKRD20A2 |  |
|  | TRIP6 |  |
|  | CLRN1-AS1 |  |
|  | NPHP3-AS1 |  |
|  | CCDC63 |  |
|  | TEX38 |  |
|  | TANC1 |  |
|  | LOC100129722 |  |
|  | OTTHUMG00000162966 |  |
|  | OTTHUMG00000165056 |  |
|  | OTTHUMG00000160880 |  |
|  | OTTHUMG00000164332 |  |
|  | LEMD1 |  |
|  | EGFL8 |  |
|  | FGFR4 |  |
|  | OTTHUMG00000163870 |  |
|  | NOTCH3 |  |
|  | PCSK2 |  |
|  | MPPED2 |  |
|  | OTTHUMG00000154285 |  |
|  | ITGB4 |  |
|  | MIR3187 |  |
|  | OTTHUMG00000014382 |  |
|  | LOC100507557 |  |
|  | DNMT3B |  |
|  | RNA5SP113 |  |
|  | LOC100132078 |  |
|  | MIR602 |  |
|  | AMOTL2 |  |
|  | ADAMTS16 |  |
|  | CLRN1 |  |
|  | MAGEA4 |  |
|  | OTTHUMG00000161179 |  |
|  | RASSF7 |  |
|  | GLT8D2 |  |
|  | SCARA5 |  |
|  | PLD5 |  |
|  | OTTHUMG00000150549 |  |
|  | P4HA2 |  |
|  | PHLDB1 |  |
|  | DLC1 |  |
|  | C4BPB |  |
|  | LOC100505624 |  |
|  | TTC29 |  |
|  | LEFTY2 |  |
|  | CYP11A1 |  |
|  | OTTHUMG00000163668 |  |
|  | MYOM2 |  |
|  | LOC100506083 |  |
|  | APOBEC1 |  |
|  | LOC100507175 |  |
|  | C1orf106 |  |
|  | MIR135B |  |
|  | AKR1C6P |  |
|  | DCN |  |
|  | HULC |  |
|  | CFHR5 |  |
|  | MIR4302 |  |
|  | SMIM17 |  |
|  | ENO4 |  |
|  | OTTHUMG00000160961 |  |
|  | SPC24 |  |
|  | LAG3 |  |
|  | LOC100505782 |  |
|  | OTTHUMG00000151848 |  |
|  | C1QTNF9B |  |
|  | LOC100506999 |  |
|  | LINC00514 |  |
|  | OTTHUMG00000020399 |  |
|  | OTTHUMG00000152979 |  |
|  | SIM2 |  |
|  | OTTHUMG00000159958 |  |
|  | OTTHUMG00000162333 |  |
|  | RTP1 |  |
|  | OTTHUMG00000157665 |  |
|  | LOC100289187 |  |
|  | FHL2 |  |
|  | NXPH3 |  |
|  | SNORD114-23 |  |
|  | OTTHUMG00000039910 |  |
|  | TRBJ2-2P |  |
|  | GRID1 |  |
|  | UTS2R |  |
|  | ZNF705A |  |
|  | OTTHUMG00000161387 |  |
|  | PPP3R2 |  |
|  | OTTHUMG00000162200 |  |
|  | C1orf127 |  |
|  | OTTHUMG00000163571 |  |
|  | C10orf90 |  |
|  | LIN28B |  |
|  | HHLA1 |  |
|  | CASQ2 |  |
|  | DNAJC5B |  |
|  | IGFBP2 |  |
|  | NFATC4 |  |
|  | CCDC74B |  |
|  | OTTHUMG00000157652 |  |
|  | CCDC42 |  |
|  | LOC100130480 |  |
|  | CFHR4 |  |
|  | OTTHUMG00000018320 |  |
|  | OTTHUMG00000151715 |  |
|  | LOC100287592 |  |
|  | LINC00271 |  |
|  | LOC100506526 |  |
|  | ATP1A2 |  |
|  | DPYS |  |
|  | ANKS1B |  |
|  | SERPINB13 |  |
|  | RNA5SP512 |  |
|  | TBX20 |  |
|  | CACNA1C-IT3 |  |
|  | OTTHUMG00000163705 |  |
|  | HEY2 |  |
|  | RNF43 |  |
|  | BLOC1S4 |  |
|  | C1orf94 |  |
|  | CAPN9 |  |
|  | OTTHUMG00000154142 |  |
|  | OTTHUMG00000010834 |  |
|  | LINC00309 |  |
|  | OTTHUMG00000048109 |  |
|  | DGKI |  |
|  | GRIN3B |  |
|  | TCF24 |  |
|  | ZNF488 |  |
|  | OTTHUMG00000015537 |  |
|  | FAM209B |  |
|  | OTTHUMG00000159625 |  |
|  | OTTHUMG00000162778 |  |
|  | SNORD114-26 |  |
|  | SYNPO2 |  |
|  | C15orf60 |  |
|  | RNA5SP72 |  |
|  | CHRD |  |
|  | DUXA |  |
|  | CRP |  |
|  | LOC100287944 |  |
|  | PTPRT |  |
|  | SLC5A2 |  |
|  | OTTHUMG00000015930 |  |
|  | ECE2 |  |
|  | LOC100996269 |  |
|  | EVPL |  |
|  | FRMD1 |  |
|  | ARHGEF38 |  |
|  | OTTHUMG00000158951 |  |
|  | LOC100507066 |  |
|  | ADAM2 |  |
|  | PPP1R9A |  |
|  | ABLIM2 |  |
|  | OTTHUMG00000162993 |  |
|  | FMO2 |  |
|  | OTTHUMG00000018892 |  |
|  | FZD4 |  |
|  | CACNG6 |  |
|  | CD276 |  |
|  | OR52A4 |  |
|  | SCIN |  |
|  | OTTHUMG00000032757 |  |
|  | SNAP25 |  |
|  | FBN1 |  |
|  | OTTHUMG00000150113 |  |
|  | OTTHUMG00000151891 |  |
|  | OTTHUMG00000024094 |  |
|  | LINC00908 |  |
|  | SLC7A2 |  |
|  | PKDCC |  |
|  | STPG2-AS1 |  |
|  | LOC284798 |  |
|  | OTTHUMG00000152688 |  |
|  | GRM7 |  |
|  | LINC00358 |  |
|  | OTTHUMG00000159418 |  |
|  | OTTHUMG00000157252 |  |
|  | OTTHUMG00000158465 |  |
|  | OTTHUMG00000019702 |  |
|  | INSL3 |  |
|  | COL6A5 |  |
|  | SLC25A18 |  |
|  | LOC100130417 |  |
|  | OTTHUMG00000073723 |  |
|  | OTTHUMG00000152731 |  |
|  | GRIN2B |  |
|  | GTF2IRD1P1 |  |
|  | UROC1 |  |
|  | ST5 |  |
|  | OTTHUMG00000003034 |  |
|  | RNA5SP235 |  |
|  | TINAG |  |
|  | HECW1 |  |
|  | OTTHUMG00000014749 |  |
|  | GGNBP1 |  |
|  | SALL2 |  |
|  | GGT6 |  |
|  | M1AP |  |
|  | FAM86A |  |
|  | C6orf132 |  |
|  | OR5H6 |  |
|  | TBX22 |  |
|  | ZNF442 |  |
|  | PRAP1 |  |
|  | ASAH2 |  |
|  | SNORA80 |  |
|  | OTTHUMG00000163447 |  |
|  | RNA5SP93 |  |
|  | LOC339807 |  |
|  | CPEB1 |  |
|  | OTTHUMG00000020433 |  |
|  | AIPL1 |  |
|  | ADAMTS7 |  |
|  | SPTA1 |  |
|  | PKD1L3 |  |
|  | OTTHUMG00000003931 |  |
|  | ELOVL2 |  |
|  | LMCD1-AS1 |  |
|  | OTTHUMG00000154838 |  |
|  | ITIH2 |  |
|  | OTTHUMG00000015508 |  |
|  | CLEC4GP1 |  |
|  | TRPC7-AS1 |  |
|  | EVPLL |  |
|  | RNA5SP281 |  |
|  | IBSP |  |
|  | OTTHUMG00000154652 |  |
|  | CDH13 |  |
|  | MAP3K9 |  |
|  | LRRIQ1 |  |
|  | ACE2 |  |
|  | LINC00222 |  |
|  | OTTHUMG00000156022 |  |
|  | OTTHUMG00000162555 |  |
|  | OTTHUMG00000161765 |  |
|  | OTTHUMG00000164604 |  |
|  | RNA5SP190 |  |
|  | OTTHUMG00000155012 |  |
|  | KIAA0319 |  |
|  | NKAIN4 |  |
|  | C3orf30 |  |
|  | CFTR |  |
|  | SPTBN2 |  |
|  | OTTHUMG00000152485 |  |
|  | C16orf96 |  |
|  | OTTHUMG00000160312 |  |
|  | RNA5SP393 |  |
|  | SLPI |  |
|  | COL18A1-AS2 |  |
|  | ATP6V0A4 |  |
|  | HHLA3 |  |
|  | MIR655 |  |
|  | RNA5SP455 |  |
|  | OTTHUMG00000150016 |  |
|  | C11orf92 |  |
|  | TMEM202 |  |
|  | OTTHUMG00000014754 |  |
|  | OTTHUMG00000152508 |  |
|  | SERPINA7 |  |
|  | OTTHUMG00000078877 |  |
|  | PLEKHH3 |  |
|  | PLEKHG4 |  |
|  | OTTHUMG00000157196 |  |
|  | GIMD1 |  |
|  | OTTHUMG00000164350 |  |
|  | PASD1 |  |
|  | IGHV1-58 |  |
|  | KRBA1 |  |
|  | MIR9-2 |  |
|  | ENPEP |  |
|  | NEU4 |  |
|  | XIRP2 |  |
|  | LINC00051 |  |
|  | KCND3-IT1 |  |
|  | OTTHUMG00000153384 |  |
|  | OTTHUMG00000159376 |  |
|  | TMEM79 |  |
|  | SLC26A10 |  |
|  | CCDC157 |  |
|  | ZNF610 |  |
|  | SLC7A13 |  |
|  | ZFPM1 |  |
|  | OR8J3 |  |
|  | BEND3P3 |  |
|  | LOC100505875 |  |
|  | OFCC1 |  |
|  | CCL28 |  |
|  | PDE6H |  |
|  | LOC100996557 |  |
|  | OR51E1 |  |
|  | LINC00466 |  |
|  | MAB21L1 |  |
|  | OTTHUMG00000164704 |  |
|  | ANKRD30BP3 |  |
|  | ADD2 |  |
|  | OTTHUMG00000157043 |  |
|  | OTTHUMG00000015742 |  |
|  | TMPRSS6 |  |
|  | OTTHUMG00000162726 |  |
|  | SLC16A9 |  |
|  | RAET1E |  |
|  | CCBE1 |  |
|  | SLC35D3 |  |
|  | OTTHUMG00000159257 |  |
|  | ADAMTS18 |  |
|  | NKAIN3 |  |
|  | ISX |  |
|  | THSD4 |  |
|  | FAM189A2 |  |
|  | AHNAK2 |  |
|  | UCP1 |  |
|  | CNTN1 |  |
|  | SLC9B1 |  |
|  | UNC79 |  |
|  | DAAM2 |  |
|  | FAM169B |  |
|  | 3-Mar |  |
|  | FSTL5 |  |
|  | SPIRE2 |  |
|  | NLGN4X |  |
|  | OTTHUMG00000035719 |  |
|  | OTTHUMG00000163251 |  |
|  | OTTHUMG00000154944 |  |
|  | ILDR2 |  |
|  | OTTHUMG00000152496 |  |
|  | LOC100996634 |  |
|  | OR52W1 |  |
|  | TVP23A |  |
|  | OTTHUMG00000031880 |  |
|  | FAM74A2 |  |
|  | CDCP2 |  |
|  | OTTHUMG00000164999 |  |
|  | MACROD2 |  |
|  | LOC100128076 |  |
|  | RNA5SP209 |  |
|  | OTTHUMG00000163986 |  |
|  | DSCAM-IT1 |  |
|  | ROR2 |  |
|  | SPAG17 |  |
|  | OTTHUMG00000015002 |  |
|  | FGD5 |  |
|  | KCND3-AS1 |  |
|  | RNA5SP407 |  |
|  | TPI1P3 |  |
|  | TRPM6 |  |
|  | AOX2P |  |
|  | TRPM5 |  |
|  | ASB14 |  |
|  | ANXA10 |  |
|  | OTTHUMG00000020554 |  |
|  | CECR3 |  |
|  | OPLAH |  |
|  | OTTHUMG00000159256 |  |
|  | ANKS6 |  |
|  | DPP10 |  |
|  | MGC15885 |  |
|  | B3GALNT1 |  |
|  | IQSEC3 |  |
|  | MIR650 |  |
|  | DNAAF1 |  |
|  | FAM196A |  |
|  | GDF7 |  |
|  | SULF1 |  |
|  | RPL19P20 |  |
|  | CTH |  |
|  | SP7 |  |
|  | OTTHUMG00000004188 |  |
|  | KCNQ5 |  |
|  | CYP4F24P |  |
|  | LOC101060385 |  |
|  | LINC00596 |  |
|  | OTTHUMG00000041155 |  |
|  | CLEC2A |  |
|  | OTTHUMG00000162989 |  |
|  | PPP2R2C |  |
|  | H1FX-AS1 |  |
|  | C9orf96 |  |
|  | OTTHUMG00000078273 |  |
|  | C18orf42 |  |
|  | OTTHUMG00000164271 |  |
|  | PIK3C2G |  |
|  | OTTHUMG00000164915 |  |
|  | ADH1B |  |
|  | NOS1AP |  |
|  | LRTOMT |  |
|  | LOC654780 |  |
|  | LYPD3 |  |
|  | OR5AK2 |  |
|  | KIRREL3 |  |
|  | LOC402160 |  |
|  | BAIAP2L2 |  |
|  | LINC00372 |  |
|  | LANCL3 |  |
|  | OTTHUMG00000153181 |  |
|  | DIO2 |  |
|  | OTTHUMG00000037165 |  |
|  | SYDE2 |  |
|  | ARMC9 |  |
|  | CPNE4 |  |
|  | LARGE-AS1 |  |
|  | OTTHUMG00000066050 |  |
|  | C20orf203 |  |
|  | FCRL4 |  |
|  | LOC100507053 |  |
|  | WBSCR27 |  |
|  | ABCB11 |  |
|  | DNAH6 |  |
|  | OTTHUMG00000154211 |  |
|  | IL1RAPL1 |  |
|  | SYN2 |  |
|  | OTTHUMG00000022178 |  |
|  | GCK |  |
|  | PRSS38 |  |
|  | CFI |  |
|  | OTTHUMG00000155117 |  |
|  | PTPRN |  |
|  | DIAPH3-AS1 |  |
|  | EBF1 |  |
|  | KIAA1217 |  |
|  | GPC1 |  |
|  | FRZB |  |
|  | OTTHUMG00000152301 |  |
|  | FGF14 |  |
|  | CPA4 |  |
|  | POU2F3 |  |
|  | TNNC2 |  |
|  | MAP6 |  |
|  | ERBB2 |  |
|  | SHH |  |
|  | MIR3926-2 |  |
|  | RNA5SP459 |  |
|  | KRT34 |  |
|  | USH2A |  |
|  | LINC00276 |  |
|  | IGHV3-21 |  |
|  | CAND2 |  |
|  | ELMO1-AS1 |  |
|  | SYDE1 |  |
|  | LOC100505973 |  |
|  | RSPH10B2 |  |
|  | LOC100996447 |  |
|  | SLC6A2 |  |
|  | ERC2 |  |
|  | CLPSL1 |  |
|  | OTTHUMG00000059641 |  |
|  | MIR4519 |  |
|  | OTTHUMG00000154964 |  |
|  | OTTHUMG00000150044 |  |
|  | DBH-AS1 |  |
|  | APOB |  |
|  | CXorf64 |  |
|  | DOK7 |  |
|  | C17orf53 |  |
|  | NCAM2 |  |
|  | DEFB122 |  |
|  | SLC27A5 |  |
|  | LOC1720 |  |
|  | OTTHUMG00000163720 |  |
|  | DOC2GP |  |
|  | OTTHUMG00000161378 |  |
|  | KRT12 |  |
|  | OTTHUMG00000161859 |  |
|  | OTTHUMG00000160532 |  |
|  | GABRB2 |  |
|  | GATA1 |  |
|  | ACSS3 |  |
|  | OTTHUMG00000032990 |  |
|  | SAC3D1 |  |
|  | DCHS2 |  |
|  | OR8U1 |  |
|  | GP2 |  |
|  | OTTHUMG00000160989 |  |
|  | OPCML |  |
|  | KCNV1 |  |
|  | ZNF704 |  |
|  | MCM10 |  |
|  | L1CAM |  |
|  | SCRG1 |  |
|  | OTTHUMG00000153891 |  |
|  | OTTHUMG00000164966 |  |
|  | LOC644145 |  |
|  | PRKAA2 |  |
|  | OTTHUMG00000017777 |  |
|  | HSD17B1P1 |  |
|  | OTTHUMG00000164913 |  |
|  | TMEM82 |  |
|  | OTTHUMG00000041347 |  |
|  | TOLLIP-AS1 |  |
|  | DOCK3 |  |
|  | SNAP25-AS1 |  |
|  | SLC17A1 |  |
|  | CDH11 |  |
|  | OTTHUMG00000020094 |  |
|  | RIPPLY2 |  |
|  | LINC00889 |  |
|  | FAT4 |  |
|  | SYTL5 |  |
|  | OTTHUMG00000142920 |  |
|  | ANKRD30B |  |
|  | PGAM2 |  |
|  | C18orf56 |  |
|  | RNY5P3 |  |
|  | LOC100129858 |  |
|  | DBC1 |  |
|  | OTTHUMG00000015298 |  |
|  | ASAH2C |  |
|  | OTX2-AS1 |  |
|  | MYH2 |  |
|  | MDFI |  |
|  | OTTHUMG00000156056 |  |
|  | GRIA4 |  |
|  | ADAM11 |  |
|  | OTTHUMG00000032185 |  |
|  | PGK2 |  |
|  | NRXN3 |  |
|  | CNN1 |  |
|  | OTTHUMG00000164905 |  |
|  | OTTHUMG00000155185 |  |
|  | IFLTD1 |  |
|  | SPTBN5 |  |
|  | LMX1A |  |
|  | COL21A1 |  |
|  | TNNC1 |  |
|  | SVOP |  |
|  | OTTHUMG00000152512 |  |
|  | OTTHUMG00000020933 |  |
|  | SERPINB7 |  |
|  | CCDC37 |  |
|  | LINC00924 |  |
|  | EMILIN1 |  |
|  | LOC100996609 |  |
|  | OTTHUMG00000032120 |  |
|  | PXDNL |  |
|  | MAGEB2 |  |
|  | C3orf52 |  |
|  | PAGE4 |  |
|  | GRIA2 |  |
|  | SAMD14 |  |
|  | OTTHUMG00000015054 |  |
|  | USP43 |  |
|  | C10orf35 |  |
|  | ALPPL2 |  |
|  | ATP6V0CP3 |  |
|  | GRIK1-AS1 |  |
|  | TTC9B |  |
|  | FLJ39080 |  |
|  | HCG20 |  |
|  | UBE2E2-AS1 |  |
|  | OTTHUMG00000161124 |  |
|  | CKMT2 |  |
|  | OTTHUMG00000010848 |  |
|  | IGHV3-64 |  |
|  | OTTHUMG00000133696 |  |
|  | GFRA3 |  |
|  | RNF150 |  |
|  | B3GNT8 |  |
|  | ACTRT3 |  |
|  | MIR210 |  |
|  | H1FOO |  |
|  | OTTHUMG00000003704 |  |
|  | OTTHUMG00000016713 |  |
|  | LNX1 |  |
|  | MLIP |  |
|  | COL9A1 |  |
|  | GPT |  |
|  | GPR113 |  |
|  | OTTHUMG00000153036 |  |
|  | OR2H1 |  |
|  | PLEKHA7 |  |
|  | OSGIN1 |  |
|  | OTTHUMG00000152139 |  |
|  | OTTHUMG00000078747 |  |
|  | C1orf172 |  |
|  | MMEL1 |  |
|  | TIMP4 |  |
|  | KLK12 |  |
|  | OTTHUMG00000163475 |  |
|  | GPHB5 |  |
|  | CHTF18 |  |
|  | OVCH1 |  |
|  | CASP12 |  |
|  | MMP28 |  |
|  | OTTHUMG00000154727 |  |
|  | ETNK2 |  |
|  | OR4A47 |  |
|  | PRTFDC1 |  |
|  | NTM |  |
|  | OR51S1 |  |
|  | RGMA |  |
|  | OTTHUMG00000153149 |  |
|  | MFSD4 |  |
|  | NOX3 |  |
|  | THNSL2 |  |
|  | IQCH |  |
|  | LOC101060360 |  |
|  | KIRREL |  |
|  | GRK7 |  |
|  | ADCY5 |  |
|  | OTTHUMG00000156571 |  |
|  | PLEKHA4 |  |
|  | MGAT3 |  |
|  | RPL29P2 |  |
|  | HAPLN2 |  |
|  | LOC442497 |  |
|  | DPY19L2P1 |  |
|  | ANKS4B |  |
|  | SLC2A4 |  |
|  | TLE2 |  |
|  | RSPH6A |  |
|  | TBXA2R |  |
|  | HMGN5 |  |
|  | LINC00942 |  |
|  | OTTHUMG00000155614 |  |
|  | MYOZ3 |  |
|  | RNA5SP160 |  |
|  | ITIH1 |  |
|  | CNKSR1 |  |
|  | OTTHUMG00000035772 |  |
|  | OR2F2 |  |
|  | SMTNL2 |  |
|  | CALCR |  |
|  | OTTHUMG00000162639 |  |
|  | FTMT |  |
|  | SCAND3 |  |
|  | IGHV3-49 |  |
|  | KL |  |
|  | LIMS2 |  |
|  | TMEM198 |  |
|  | OTTHUMG00000041501 |  |
|  | OTTHUMG00000017360 |  |
|  | OTTHUMG00000163137 |  |
|  | ACTL8 |  |
|  | FAM166A |  |
|  | TSPEAR |  |
|  | PDE6C |  |
|  | OTTHUMG00000162974 |  |
|  | OTTHUMG00000159160 |  |
|  | OTTHUMG00000161393 |  |
|  | OTTHUMG00000151774 |  |
|  | NYAP1 |  |
|  | CLDN11 |  |
|  | OTTHUMG00000019570 |  |
|  | TRIM61 |  |
|  | LOC100132167 |  |
|  | OTTHUMG00000159585 |  |
|  | LINC00609 |  |
|  | OTTHUMG00000163701 |  |
|  | MLPH |  |
|  | AKAP4 |  |
|  | TMCO5B |  |
|  | MIR99A |  |
|  | SV2C |  |
|  | RNA5SP203 |  |
|  | RNFT2 |  |
|  | FAM47E |  |
|  | DUSP27 |  |
|  | OTTHUMG00000155740 |  |
|  | ATP1B4 |  |
|  | OTTHUMG00000157253 |  |
|  | PTPRZ1 |  |
|  | WNT8B |  |
|  | TMPRSS13 |  |
|  | ANO3 |  |
|  | HTR3A |  |
|  | LYZL4 |  |
|  | AGR3 |  |
|  | UPK3B |  |
|  | EGFEM1P |  |
|  | LOC401010 |  |
|  | OTTHUMG00000065034 |  |
|  | PIF1 |  |
|  | OTTHUMG00000164389 |  |
|  | NOS1 |  |
|  | NIPAL4 |  |
|  | RNF175 |  |
|  | FDXR |  |
|  | C5orf64 |  |
|  | OTTHUMG00000151907 |  |
|  | GCM2 |  |
|  | VWF |  |
|  | MIR122 |  |
|  | CARD10 |  |
|  | IL25 |  |
|  | ALPP |  |
|  | OTTHUMG00000018538 |  |
|  | C10orf112 |  |
|  | LOC440518 |  |
|  | SNORA35 |  |
|  | NCR1 |  |
|  | FLJ40194 |  |
|  | TENC1 |  |
|  | LOC154092 |  |
|  | EDNRB |  |
|  | OTTHUMG00000037001 |  |
|  | NTN3 |  |
|  | VWA3B |  |
|  | PADI3 |  |
|  | OTTHUMG00000015507 |  |
|  | OLAH |  |
|  | AADACL3 |  |
|  | RAB19 |  |
|  | FAM171A2 |  |
|  | OTTHUMG00000156043 |  |
|  | LRRC53 |  |
|  | SEC14L2 |  |
|  | RAX2 |  |
|  | SLC15A1 |  |
|  | LMOD2 |  |
|  | PTPRH |  |
|  | GUCA1C |  |
|  | EBF2 |  |
|  | KRT18 |  |
|  | CDCA5 |  |
|  | CDC42EP5 |  |
|  | MIR4713 |  |
|  | SRD5A2 |  |
|  | SPAG11A |  |
|  | LINC00547 |  |
|  | ENOX1-AS2 |  |
|  | DDX53 |  |
|  | OR5G5P |  |
|  | SLC5A10 |  |
|  | LOC101060175 |  |
|  | TINAGL1 |  |
|  | OTTHUMG00000039487 |  |
|  | C1orf226 |  |
|  | OTTHUMG00000158801 |  |
|  | PODXL2 |  |
|  | LOC100996378 |  |
|  | WBSCR17 |  |
|  | OTTHUMG00000152120 |  |
|  | GREB1 |  |
|  | ARL14EPL |  |
|  | THRB |  |
|  | FLJ25715 |  |
|  | LOC151475 |  |
|  | GABBR2 |  |
|  | AZGP1 |  |
|  | CHRNA10 |  |
|  | OTTHUMG00000015125 |  |
|  | GAL |  |
|  | CCDC13 |  |
|  | MORN4 |  |
|  | NME9 |  |
|  | OCM |  |
|  | SYNPO2L |  |
|  | MMP27 |  |
|  | ABCC12 |  |
|  | GJB1 |  |
|  | FLJ35024 |  |
|  | SOAT2 |  |
|  | OTTHUMG00000148655 |  |
|  | ZNF446 |  |
|  | LINC00507 |  |
|  | MIR936 |  |
|  | ADAM30 |  |
|  | MYO3B |  |
|  | OTTHUMG00000153028 |  |
|  | OTOG |  |
|  | ZNF572 |  |
|  | WIPF3 |  |
|  | ARHGAP42 |  |
|  | NEUROD4 |  |
|  | FAM222A |  |
|  | SNORD116-19 |  |
|  | PRR15L |  |
|  | OTTHUMG00000020380 |  |
|  | PRM2 |  |
|  | LOC100506684 |  |
|  | OTTHUMG00000019544 |  |
|  | OTTHUMG00000154056 |  |
|  | CCDC27 |  |
|  | RHBG |  |
|  | MIR137 |  |
|  | PPARGC1A |  |
|  | LOC285626 |  |
|  | LRCOL1 |  |
|  | LCN6 |  |
|  | PRSS27 |  |
|  | OTTHUMG00000018728 |  |
|  | ZMAT4 |  |
|  | JAZF1-AS1 |  |
|  | EPB41L1 |  |
|  | HGD |  |
|  | OTTHUMG00000159896 |  |
|  | IQCF6 |  |
|  | LOC100506801 |  |
|  | OTTHUMG00000150934 |  |
|  | SMTN |  |
|  | OTTHUMG00000157098 |  |
|  | NT5C1A |  |
|  | OTTHUMG00000020975 |  |
|  | CHIAP2 |  |
|  | KCTD16 |  |
|  | OTTHUMG00000164677 |  |
|  | WFDC10B |  |
|  | FCAMR |  |
|  | MLXIPL |  |
|  | KRT27 |  |
|  | TRMT61A |  |
|  | APOA5 |  |
|  | OTTHUMG00000162209 |  |
|  | CSDC2 |  |
|  | ELFN2 |  |
|  | OTTHUMG00000153047 |  |
|  | OTTHUMG00000161187 |  |
|  | OTTHUMG00000153333 |  |
|  | KLHL3 |  |
|  | USP2-AS1 |  |
|  | FHL5 |  |
|  | LAMA4 |  |
|  | COL13A1 |  |
|  | TRIM31 |  |
|  | OTTHUMG00000041417 |  |
|  | OTTHUMG00000164348 |  |
|  | LOC100507377 |  |
|  | MDS2 |  |
|  | IL6 |  |
|  | LOC100131289 |  |
|  | ANKRD30BL |  |
|  | OTTHUMG00000017135 |  |
|  | OTTHUMG00000152416 |  |
|  | BEND5 |  |
|  | CTSL1P8 |  |
|  | MIR3157 |  |
|  | GRID2IP |  |
|  | ASB16 |  |
|  | LRP4 |  |
|  | PRDM6 |  |
|  | ACER1 |  |
|  | LINC00244 |  |
|  | MEP1A |  |
|  | MUC6 |  |
|  | FOSL1 |  |
|  | CYP26C1 |  |
|  | KCNH7 |  |
|  | SNCAIP |  |
|  | NKX6-3 |  |
|  | OTTHUMG00000155766 |  |
|  | TNXB |  |
|  | ENTPD3 |  |
|  | DRP2 |  |
|  | TF |  |
|  | MYO18B |  |
|  | PFN4 |  |
|  | OTTHUMG00000017187 |  |
|  | OTTHUMG00000153489 |  |
|  | COL12A1 |  |
|  | OTTHUMG00000007920 |  |
|  | PRAME |  |
|  | DUSP26 |  |
|  | OTTHUMG00000009930 |  |
|  | SSTR2 |  |
|  | DNAJC9-AS1 |  |
|  | PSPN |  |
|  | OTTHUMG00000163702 |  |
|  | LOC100131138 |  |
|  | MAGEB3 |  |
|  | OTTHUMG00000141277 |  |
|  | ZBED3-AS1 |  |
|  | DNAI1 |  |
|  | CACNA1E |  |
|  | PRSS56 |  |
|  | OTTHUMG00000009147 |  |
|  | RTDR1 |  |
|  | TIE1 |  |
|  | OTTHUMG00000157861 |  |
|  | ABCC11 |  |
|  | CYTL1 |  |
|  | ESPNL |  |
|  | LINC00948 |  |
|  | CORIN |  |
|  | RNU6-49 |  |
|  | LPIN3 |  |
|  | RPH3AL |  |
|  | TBX15 |  |
|  | GPC5 |  |
|  | FLG-AS1 |  |
|  | C14orf164 |  |
|  | KCNU1 |  |
|  | CSN1S2AP |  |
|  | TCHHL1 |  |
|  | ATP2B2 |  |
|  | OTTHUMG00000132645 |  |
|  | KLHL32 |  |
|  | TMEM184A |  |
|  | OTTHUMG00000163488 |  |
|  | CCDC33 |  |
|  | OTTHUMG00000162146 |  |
|  | KHDC1L |  |
|  | CLYBL-AS1 |  |
|  | OTTHUMG00000015142 |  |
|  | CCR9 |  |
|  | PRAMEF13 |  |
|  | CRNN |  |
|  | SV2A |  |
|  | PCDH11X |  |
|  | MGC24103 |  |
|  | C22orf24 |  |
|  | RNA5SP248 |  |
|  | KCNQ5-AS1 |  |
|  | LOC145845 |  |
|  | FRMD5 |  |
|  | PLEKHG4B |  |
|  | TECTA |  |
|  | OTTHUMG00000020075 |  |
|  | VWA3A |  |
|  | EXOC3L1 |  |
|  | FLRT3 |  |
|  | CD79A |  |
|  | ACSM2B |  |
|  | KEL |  |
|  | LOC285768 |  |
|  | SH3GL3 |  |
|  | CBY3 |  |
|  | CHRDL1 |  |
|  | LOC149950 |  |
|  | MAGEA6 |  |
|  | OTTHUMG00000152648 |  |
|  | LOC100506303 |  |
|  | CES5AP1 |  |
|  | MIR520C |  |
|  | LIPK |  |
|  | FRMD8P1 |  |
|  | LRRTM3 |  |
|  | LOC100506422 |  |
|  | LOC100506107 |  |
|  | OTTHUMG00000152710 |  |
|  | EIF4E1B |  |
|  | GDAP1L1 |  |
|  | OTTHUMG00000163713 |  |
|  | OTTHUMG00000007879 |  |
|  | OTTHUMG00000161689 |  |
|  | ZNF503-AS2 |  |
|  | PTPN21 |  |
|  | GPR151 |  |
|  | OTTHUMG00000020029 |  |
|  | TRIM9 |  |
|  | ATP4A |  |
|  | OTTHUMG00000031675 |  |
|  | OTTHUMG00000150470 |  |
|  | OTTHUMG00000032013 |  |
|  | OTTHUMG00000160920 |  |
|  | RNU105C |  |
|  | FGF12-AS3 |  |
|  | LINC00661 |  |
|  | LOC100507560 |  |
|  | CCDC148-AS1 |  |
|  | SCN1A |  |
|  | GAS2L2 |  |
|  | NEURL2 |  |
|  | MIR4500HG |  |
|  | RNA5SP390 |  |
|  | IGSF22 |  |
|  | RBM20 |  |
|  | OTTHUMG00000154822 |  |
|  | HAMP |  |
|  | RNA5SP237 |  |
|  | OTTHUMG00000154350 |  |
|  | ARSI |  |
|  | OTTHUMG00000159705 |  |
|  | LRRC48 |  |
|  | OTTHUMG00000153077 |  |
|  | MYOG |  |
|  | OTTHUMG00000159504 |  |
|  | RHBDL3 |  |
|  | KIF6 |  |
|  | LCN15 |  |
|  | OTTHUMG00000018537 |  |
|  | PAX7 |  |
|  | OTTHUMG00000041597 |  |
|  | SDR9C7 |  |
|  | SLC9A3R2 |  |
|  | CALY |  |
|  | OTTHUMG00000150449 |  |
|  | XDH |  |
|  | SLC30A8 |  |
|  | FAM92B |  |
|  | LOC643770 |  |
|  | FAM201A |  |
|  | MIR214 |  |
|  | BCL6B |  |
|  | KRT17P1 |  |
|  | OTTHUMG00000021406 |  |
|  | NUP210L |  |
|  | LINC00161 |  |
|  | OTTHUMG00000020552 |  |
|  | OTTHUMG00000160691 |  |
|  | RNU6-64P |  |
|  | OTTHUMG00000018644 |  |
|  | LHFPL1 |  |
|  | OTTHUMG00000151373 |  |
|  | MANSC1 |  |
|  | RNF151 |  |
|  | NAALADL2-AS2 |  |
|  | NTF3 |  |
|  | WNT9B |  |
|  | EYA2 |  |
|  | OTTHUMG00000163801 |  |
|  | OTTHUMG00000045138 |  |
|  | TSPAN6 |  |
|  | ITGA8 |  |
|  | OTTHUMG00000156017 |  |
|  | OTTHUMG00000032768 |  |
|  | SDK2 |  |
|  | OTTHUMG00000152414 |  |
|  | LOC392232 |  |
|  | OTTHUMG00000153238 |  |
|  | LINC00307 |  |
|  | NLRP5 |  |
|  | TTC9 |  |
|  | OTTHUMG00000163336 |  |
|  | SPAG6 |  |
|  | GCG |  |
|  | OTTHUMG00000161130 |  |
|  | IZUMO4 |  |
|  | CDC42BPG |  |
|  | JSRP1 |  |
|  | OTTHUMG00000019300 |  |
|  | FCRL1 |  |
|  | TNFRSF4 |  |
|  | C2orf48 |  |
|  | IL20 |  |
|  | OTTHUMG00000018682 |  |
|  | ASPA |  |
|  | RNA5SP95 |  |
|  | PLCE1 |  |
|  | PAMR1 |  |
|  | LOC100505985 |  |
|  | PTPRD |  |
|  | CCDC120 |  |
|  | CDH16 |  |
|  | SEMA5B |  |
|  | OTTHUMG00000164595 |  |
|  | OTTHUMG00000155825 |  |
|  | GCKR |  |
|  | OTTHUMG00000151722 |  |
|  | DGAT2L7P |  |
|  | SPRY3 |  |
|  | SNORD113-5 |  |
|  | CCL24 |  |
|  | ACSBG2 |  |
|  | CA1 |  |
|  | OTTHUMG00000059148 |  |
|  | OTTHUMG00000010623 |  |
|  | OTTHUMG00000019288 |  |
|  | ENHO |  |
|  | MAMLD1 |  |
|  | OTTHUMG00000140105 |  |
|  | OTTHUMG00000160282 |  |
|  | LPPR1 |  |
|  | OTTHUMG00000020176 |  |
|  | LOC285501 |  |
|  | OTTHUMG00000019699 |  |
|  | OTTHUMG00000090408 |  |
|  | GRIN3A |  |
|  | ARHGEF16 |  |
|  | PPFIA4 |  |
|  | TTLL11-IT1 |  |
|  | CLPS |  |
|  | OTTHUMG00000066812 |  |
|  | CNTN2 |  |
|  | ZBTB7C |  |
|  | DKK3 |  |
|  | LIPI |  |
|  | WSCD2 |  |
|  | WWC1 |  |
|  | RAPGEF3 |  |
|  | OTTHUMG00000086192 |  |
|  | BCL2L10 |  |
|  | OTTHUMG00000164613 |  |
|  | AGTR1 |  |
|  | OTTHUMG00000153836 |  |
|  | KRT3 |  |
|  | TMEM225 |  |
|  | OTTHUMG00000154546 |  |
|  | OTTHUMG00000160251 |  |
|  | FAM181A-AS1 |  |
|  | ABCG2 |  |
|  | FAT1 |  |
|  | SLC17A3 |  |
|  | NPAS1 |  |
|  | ADAMTSL2 |  |
|  | KCNB1 |  |
|  | ZDHHC19 |  |
|  | KLHL14 |  |
|  | OTTHUMG00000018047 |  |
|  | OTTHUMG00000159845 |  |
|  | IYD |  |
|  | POPDC3 |  |
|  | IL23A |  |
|  | PTCHD4 |  |
|  | SEMA3A |  |
|  | SPEG |  |
|  | HOXD9 |  |
|  | FER1L6-AS2 |  |
|  | SFTPD |  |
|  | OTTHUMG00000164174 |  |
|  | EMX1 |  |
|  | KAZALD1 |  |
|  | RGS7BP |  |
|  | ZMYND10 |  |
|  | ABCC8 |  |
|  | DCST1 |  |
|  | RPL34-AS1 |  |
|  | MGC34034 |  |
|  | OTTHUMG00000017032 |  |
|  | VCX |  |
|  | LOC285762 |  |
|  | HMGB3P1 |  |
|  | MYOM3 |  |
|  | PAK7 |  |
|  | LCE3C |  |
|  | OTTHUMG00000017052 |  |
|  | SLC35G3 |  |
|  | LOC100505697 |  |
|  | OTTHUMG00000161226 |  |
|  | OTTHUMG00000163337 |  |
|  | OTTHUMG00000010944 |  |
|  | HMGCS2 |  |
|  | MLK7-AS1 |  |
|  | LOC100134868 |  |
|  | OTTHUMG00000000497 |  |
|  | TTLL9 |  |
|  | CSNK1G2-AS1 |  |
|  | OTTHUMG00000162888 |  |
|  | OTTHUMG00000011907 |  |
|  | LINC00421 |  |
|  | EDNRA |  |
|  | AMPD1 |  |
|  | LOC100505547 |  |
|  | PHGDH |  |
|  | PARD3B |  |
|  | OR12D3 |  |
|  | BSND |  |
|  | OTTHUMG00000066052 |  |
|  | OTTHUMG00000155311 |  |
|  | CDHR2 |  |
|  | ARC |  |
|  | OR10AB1P |  |
|  | LINC00550 |  |
|  | C14orf180 |  |
|  | COL28A1 |  |
|  | SH3TC2 |  |
|  | LOC100506289 |  |
|  | GREB1L |  |
|  | OTTHUMG00000163307 |  |
|  | GRHL2 |  |
|  | OTTHUMG00000162816 |  |
|  | CPXM2 |  |
|  | NRXN1 |  |
|  | CALCA |  |
|  | OTTHUMG00000163018 |  |
|  | OTTHUMG00000153147 |  |
|  | ATXN8OS |  |
|  | LOC100129345 |  |
|  | OR6K3 |  |
|  | LOC100506238 |  |
|  | SLFNL1 |  |
|  | UNC80 |  |
|  | DNAI2 |  |
|  | FLJ33534 |  |
|  | DIRAS2 |  |
|  | GLT6D1 |  |
|  | OTTHUMG00000161115 |  |
|  | PCLO |  |
|  | DKFZp434L192 |  |
|  | COL7A1 |  |
|  | LOC145837 |  |
|  | FNDC4 |  |
|  | HIF3A |  |
|  | KCNE2 |  |
|  | SIRT4 |  |
|  | ZYG11A |  |
|  | C21orf37 |  |
|  | TLX2 |  |
|  | TRIM49 |  |
|  | CYP2C18 |  |
|  | OTTHUMG00000162968 |  |
|  | CLEC3A |  |
|  | OTTHUMG00000157225 |  |
|  | MIR920 |  |
|  | TGIF2LX |  |
|  | YIPF7 |  |
|  | FAM74A1 |  |
|  | CCDC135 |  |
|  | RNA5SP142 |  |
|  | LOC645638 |  |
|  | HSPG2 |  |
|  | SLC6A5 |  |
|  | NAV2 |  |
|  | LOC100506465 |  |
|  | OR2D2 |  |
|  | LINC00326 |  |
|  | LRRTM4 |  |
|  | ABCC9 |  |
|  | DMKN |  |
|  | OTTHUMG00000154176 |  |
|  | TUBB3 |  |
|  | PCDP1 |  |
|  | RBMS3 |  |
|  | OTTHUMG00000154250 |  |
|  | B3GALT5 |  |
|  | PPL |  |
|  | GLP1R |  |
|  | MIR4486 |  |
|  | TRBV6-4 |  |
|  | UPP2 |  |
|  | EMID1 |  |
|  | MIR4300 |  |
|  | KIF17 |  |
|  | LOC285819 |  |
|  | CYP4F3 |  |
|  | ZFR2 |  |
|  | FAM172BP |  |
|  | BOLL |  |
|  | FAM83A |  |
|  | TMIGD1 |  |
|  | TMEM182 |  |
|  | OTTHUMG00000161392 |  |
|  | HMCN2 |  |
|  | OTTHUMG00000162463 |  |
|  | ACOXL |  |
|  | COL23A1 |  |
|  | SERPINB3 |  |
|  | BFSP2 |  |
|  | SCNN1D |  |
|  | LOC100128131 |  |
|  | OTTHUMG00000153516 |  |
|  | OTTHUMG00000162499 |  |
|  | OTTHUMG00000018322 |  |
|  | OTTHUMG00000090402 |  |
|  | DBX2 |  |
|  | OTTHUMG00000153381 |  |
|  | IGHV3-11 |  |
|  | ARNT2 |  |
|  | TCN1 |  |
|  | LOC283440 |  |
|  | TM4SF1 |  |
|  | OTTHUMG00000152505 |  |
|  | OTTHUMG00000160132 |  |
|  | GRIK4 |  |
|  | GPRC5C |  |
|  | OR2T33 |  |
|  | NYNRIN |  |
|  | LOC100288336 |  |
|  | CHRDL2 |  |
|  | SPG20OS |  |
|  | OTTHUMG00000153911 |  |
|  | RNA5SP224 |  |
|  | ADIPOQ |  |
|  | AIF1L |  |
|  | PRAMEF19 |  |
|  | ASPG |  |
|  | OTTHUMG00000164285 |  |
|  | MIR4706 |  |
|  | GPR123 |  |
|  | AATK-AS1 |  |
|  | PRNT |  |
|  | LRRC4B |  |
|  | MAGEB1 |  |
|  | CADM3 |  |
|  | MYBPC1 |  |
|  | PLS1 |  |
|  | LOC100506881 |  |
|  | OTTHUMG00000161197 |  |
|  | WFDC3 |  |
|  | D21S2088E |  |
|  | OTTHUMG00000162350 |  |
|  | PKD1L1 |  |
|  | MIR1471 |  |
|  | KAZN |  |
|  | ELOVL2-AS1 |  |
|  | ADAMTS20 |  |
|  | KRT40 |  |
|  | TNFRSF13B |  |
|  | PRAMEF14 |  |
|  | OLFM4 |  |
|  | METTL24 |  |
|  | SHBG |  |
|  | DSCAML1 |  |
|  | SMTNL1 |  |
|  | NPHS1 |  |
|  | HYAL1 |  |
|  | SLC22A14 |  |
|  | NAV3 |  |
|  | OTTHUMG00000074517 |  |
|  | OTTHUMG00000162460 |  |
|  | OTTHUMG00000162053 |  |
|  | OTTHUMG00000151421 |  |
|  | PTPN3 |  |
|  | ITGBL1 |  |
|  | RNA5SP231 |  |
|  | HAPLN1 |  |
|  | OTTHUMG00000164247 |  |
|  | CNTNAP3 |  |
|  | TRPM1 |  |
|  | SHISA7 |  |
|  | OTTHUMG00000163836 |  |
|  | SPZ1 |  |
|  | TRPM3 |  |
|  | OTTHUMG00000019750 |  |
|  | PCNAP1 |  |
|  | FAM83A-AS1 |  |
|  | RNA5SP43 |  |
|  | VWCE |  |
|  | RNF26 |  |
|  | RAB40A |  |
|  | OTTHUMG00000147348 |  |
|  | SERPINA10 |  |
|  | LOC284581 |  |
|  | MIR744 |  |
|  | MID1 |  |
|  | OTTHUMG00000162685 |  |
|  | SPATA21 |  |
|  | LOC100652824 |  |
|  | NKD2 |  |
|  | OR6F1 |  |
|  | LRP2BP |  |
|  | OTTHUMG00000160881 |  |
|  | BGN |  |
|  | CYP2W1 |  |
|  | OTTHUMG00000152909 |  |
|  | KCP |  |
|  | C3orf49 |  |
|  | TRBV5-7 |  |
|  | RUNX1T1 |  |
|  | OTTHUMG00000032717 |  |
|  | C10orf99 |  |
|  | PPP1R2P9 |  |
|  | VSTM4 |  |
|  | PRSS54 |  |
|  | DSG3 |  |
|  | IQCA1 |  |
|  | AKNAD1 |  |
|  | OTTHUMG00000031831 |  |
|  | DEFB103A |  |
|  | OTTHUMG00000032958 |  |
|  | OTTHUMG00000164251 |  |
|  | OR5C1 |  |
|  | UPK1B |  |
|  | OTTHUMG00000032284 |  |
|  | FILIP1 |  |
|  | LOC100130548 |  |
|  | XKR4 |  |
|  | LOC100131047 |  |
|  | FLT1 |  |
|  | AFAP1L1 |  |
|  | TJP3 |  |
|  | OTTHUMG00000160063 |  |
|  | NUTM2F |  |
|  | OTTHUMG00000019203 |  |
|  | SDCBP2 |  |
|  | CTAG2 |  |
|  | LRRC31 |  |
|  | OTTHUMG00000163615 |  |
|  | KRT7 |  |
|  | OTTHUMG00000153018 |  |
|  | CSF3 |  |
|  | LOC284294 |  |
|  | VIT |  |
|  | EPO |  |
|  | LOC100130015 |  |
|  | MFAP5 |  |
|  | OTTHUMG00000152016 |  |
|  | ASIC3 |  |
|  | MYT1L |  |
|  | OTTHUMG00000153380 |  |
|  | OTTHUMG00000017863 |  |
|  | PLEKHA6 |  |
|  | OTTHUMG00000163514 |  |
|  | OTTHUMG00000161633 |  |
|  | OTTHUMG00000013351 |  |
|  | FAM180A |  |
|  | RBBP8NL |  |
|  | OTTHUMG00000033265 |  |
|  | PLEKHH1 |  |
|  | ANKRD6 |  |
|  | OTTHUMG00000018924 |  |
|  | OTTHUMG00000003650 |  |
|  | OTTHUMG00000033178 |  |
|  | HAVCR1 |  |
|  | HLCS-IT1 |  |
|  | CREB3L3 |  |
|  | SMIM1 |  |
|  | OTTHUMG00000161489 |  |
|  | OSBPL10-AS1 |  |
|  | OTTHUMG00000156391 |  |
|  | TMEM150C |  |
|  | OTTHUMG00000016054 |  |
|  | CDON |  |
|  | OTTHUMG00000154317 |  |
|  | OTTHUMG00000163215 |  |
|  | MIR4264 |  |
|  | PPFIA2 |  |
|  | OTTHUMG00000017165 |  |
|  | OTTHUMG00000157208 |  |
|  | OTTHUMG00000151713 |  |
|  | CSRNP3 |  |
|  | GREM1 |  |
|  | PLXNB1 |  |
|  | BTN1A1 |  |
|  | MEOX2 |  |
|  | OTTHUMG00000152594 |  |
|  | KCNV2 |  |
|  | C4orf51 |  |
|  | OTTHUMG00000151392 |  |
|  | KCNH5 |  |
|  | MFI2 |  |
|  | OTTHUMG00000152878 |  |
|  | OTTHUMG00000156423 |  |
|  | NTRK1 |  |
|  | TMEM61 |  |
|  | C21orf15 |  |
|  | FANCD2OS |  |
|  | IGSF11 |  |
|  | IFNA6 |  |
|  | OTTHUMG00000163178 |  |
|  | TEAD2 |  |
|  | TBC1D3P5 |  |
|  | ACAN |  |
|  | NRAP |  |
|  | MCHR1 |  |
|  | ARHGEF4 |  |
|  | RPL23AP32 |  |
|  | FAM222A-AS1 |  |
|  | OTTHUMG00000018145 |  |
|  | NMS |  |
|  | ADARB2 |  |
|  | C9orf169 |  |
|  | OTTHUMG00000148821 |  |
|  | OTTHUMG00000140114 |  |
|  | OTTHUMG00000018294 |  |
|  | OTTHUMG00000152535 |  |
|  | GSG2 |  |
|  | APOM |  |
|  | UMOD |  |
|  | MIR194-1 |  |
|  | TUSC3 |  |
|  | OTTHUMG00000163859 |  |
|  | FGF13 |  |
|  | LOC100507531 |  |
|  | OR2M1P |  |
|  | FER1L5 |  |
|  | MIR3944 |  |
|  | SYNPO |  |
|  | ZNF165 |  |
|  | OPN4 |  |
|  | OTTHUMG00000160733 |  |
|  | OTTHUMG00000164814 |  |
|  | RNA5SP176 |  |
|  | KRT84 |  |
|  | TGM6 |  |
|  | QRFPR |  |
|  | OTTHUMG00000164199 |  |
|  | OTTHUMG00000162564 |  |
|  | LOC100093698 |  |
|  | OTTHUMG00000160745 |  |
|  | PLS1-AS1 |  |
|  | CHRND |  |
|  | OTTHUMG00000165067 |  |
|  | OTTHUMG00000020115 |  |
|  | THSD7A |  |
|  | MIR1202 |  |
|  | ALS2CL |  |
|  | OTTHUMG00000154724 |  |
|  | HTR2A |  |
|  | PNMA5 |  |
|  | OTTHUMG00000155377 |  |
|  | DGKK |  |
|  | CAPN5 |  |
|  | OTTHUMG00000016932 |  |
|  | DUOX2 |  |
|  | KIRREL2 |  |
|  | DAB2IP |  |
|  | OTTHUMG00000150904 |  |
|  | OTTHUMG00000137386 |  |
|  | FBLIM1 |  |
|  | OTTHUMG00000032967 |  |
|  | LAMA1 |  |
|  | OTTHUMG00000151289 |  |
|  | OTTHUMG00000037125 |  |
|  | PSAT1 |  |
|  | OTTHUMG00000151405 |  |
|  | SCTR |  |
|  | KIF18B |  |
|  | BLACE |  |
|  | OTTHUMG00000161879 |  |
|  | OTTHUMG00000004830 |  |
|  | RNA5SP220 |  |
|  | RNF208 |  |
|  | OTTHUMG00000156715 |  |
|  | WDR34 |  |
|  | OTTHUMG00000161396 |  |
|  | RASAL1 |  |
|  | GPR110 |  |
|  | MROH7 |  |
|  | PTPN5 |  |
|  | FLJ37505 |  |
|  | FAM19A1 |  |
|  | CEACAM6 |  |
|  | LOC93432 |  |
|  | SLIT3 |  |
|  | OTTHUMG00000164273 |  |
|  | RNA5SP234 |  |
|  | NDUFA4L2 |  |
|  | MFAP2 |  |
|  | OTTHUMG00000157041 |  |
|  | CNGB3 |  |
|  | PRAMEF18 |  |
|  | SYT9 |  |
|  | PNMA6B |  |
|  | LOC646862 |  |
|  | OTTHUMG00000162373 |  |
|  | OTTHUMG00000164491 |  |
|  | OTTHUMG00000154387 |  |
|  | OPRM1 |  |
|  | OTTHUMG00000154367 |  |
|  | SNCG |  |
|  | OTTHUMG00000164822 |  |
|  | MIR212 |  |
|  | UGT2B17 |  |
|  | ABCA4 |  |
|  | OTTHUMG00000017139 |  |
|  | LINC00346 |  |
|  | OTTHUMG00000017655 |  |
|  | ASIP |  |
|  | TTTY18 |  |
|  | OTTHUMG00000020056 |  |
|  | MYO5B |  |
|  | OTTHUMG00000163752 |  |
|  | CCDC40 |  |
|  | MIR31 |  |
|  | OTTHUMG00000134272 |  |
|  | OTTHUMG00000164521 |  |
|  | PRICKLE2-AS1 |  |
|  | OTTHUMG00000000941 |  |
|  | OTTHUMG00000019594 |  |
|  | CPB2 |  |
|  | OTTHUMG00000158358 |  |
|  | LINC00616 |  |
|  | OTTHUMG00000035890 |  |
|  | NPM3 |  |
|  | PDE1A |  |
|  | ACMSD |  |
|  | SEMA6A |  |
|  | PKHD1 |  |
|  | GPR144 |  |
|  | OTTHUMG00000019749 |  |
|  | CSMD3 |  |
|  | C11orf94 |  |
|  | DACT2 |  |
|  | A2ML1 |  |
|  | TRO |  |
|  | SLC10A1 |  |
|  | RNA5SP505 |  |
|  | OTTHUMG00000032129 |  |
|  | ZDHHC15 |  |
|  | YPEL4 |  |
|  | OTTHUMG00000019262 |  |
|  | SAA3P |  |
|  | RNA5SP503 |  |
|  | LOC100128028 |  |
|  | OTTHUMG00000162534 |  |
|  | LYPD6 |  |
|  | TRIM43 |  |
|  | BAALC |  |
|  | TTTY6 |  |
|  | OTTHUMG00000155483 |  |
|  | OTTHUMG00000046188 |  |
|  | MT1P2 |  |
|  | KALRN |  |
|  | FGF5 |  |
|  | LARGE |  |
|  | LINC00698 |  |
|  | KLF17 |  |
|  | FAM197Y2 |  |
|  | OTTHUMG00000020256 |  |
|  | FAM5B |  |
|  | OTTHUMG00000163257 |  |
|  | OTTHUMG00000162339 |  |
|  | ZNF835 |  |
|  | LOC100507250 |  |
|  | OTTHUMG00000159626 |  |
|  | STOX2 |  |
|  | NKPD1 |  |
|  | OTTHUMG00000160104 |  |
|  | PLA2G12B |  |
|  | GYPE |  |
|  | TRIM55 |  |
|  | LOC645752 |  |
|  | EGFLAM |  |
|  | FTCD |  |
|  | SHANK2-AS3 |  |
|  | SLC4A1 |  |
|  | C10orf105 |  |
|  | C16orf45 |  |
|  | WDR93 |  |
|  | OTTHUMG00000150933 |  |
|  | TRIM10 |  |
|  | DLGAP1 |  |
|  | OTTHUMG00000164598 |  |
|  | DPP6 |  |
|  | CYP2B6 |  |
|  | OTTHUMG00000018330 |  |
|  | IZUMO1 |  |
|  | OTTHUMG00000056480 |  |
|  | HECW2 |  |
|  | IGSF1 |  |
|  | OTTHUMG00000165062 |  |
|  | OTTHUMG00000162964 |  |
|  | LINC00548 |  |
|  | RNA5SP343 |  |
|  | SLC5A9 |  |
|  | ZNF716 |  |
|  | SHROOM3 |  |
|  | C9orf117 |  |
|  | MIR3185 |  |
|  | OTTHUMG00000150135 |  |
|  | ADAMTS4 |  |
|  | TGIF2LY |  |
|  | OTTHUMG00000017555 |  |
|  | KY |  |
|  | OTTHUMG00000009790 |  |
|  | RIMS1 |  |
|  | ADAMTS6 |  |
|  | OTTHUMG00000059482 |  |
|  | OTTHUMG00000014332 |  |
|  | OTTHUMG00000157254 |  |
|  | ADAMTS8 |  |
|  | CCDC103 |  |
|  | OTTHUMG00000018393 |  |
|  | OTTHUMG00000153665 |  |
|  | LOC283683 |  |
|  | RYR2 |  |
|  | LOC100996664 |  |
|  | GPC3 |  |
|  | KRT31 |  |
|  | MYH4 |  |
|  | FAM9C |  |
|  | SH2D6 |  |
|  | ROPN1 |  |
|  | GOLGA2P2Y |  |
|  | SEPT14 |  |
|  | ANKRD1 |  |
|  | CCDC74A |  |
|  | IGLV3-32 |  |
|  | UOX |  |
|  | LOC400794 |  |
|  | OTTHUMG00000153200 |  |
|  | NEFM |  |
|  | OTTHUMG00000163980 |  |
|  | CAMK1G |  |
|  | ANXA13 |  |
|  | NTRK3-AS1 |  |
|  | OTTHUMG00000164850 |  |
|  | RCAN3AS |  |
|  | FXYD4 |  |
|  | C1orf145 |  |
|  | GUCA1A |  |
|  | C1orf222 |  |
|  | OTTHUMG00000159735 |  |
|  | DIO1 |  |
|  | LOC100289473 |  |
|  | LINC00158 |  |
|  | ADCY1 |  |
|  | PRKD1 |  |
|  | LINC00333 |  |
|  | OTTHUMG00000153096 |  |
|  | LINC00347 |  |
|  | OTTHUMG00000002490 |  |
|  | OTTHUMG00000015981 |  |
|  | LOC79015 |  |
|  | SLC7A11-AS1 |  |
|  | OTTHUMG00000150092 |  |
|  | CEMP1 |  |
|  | C16orf86 |  |
|  | PTPN20B |  |
|  | C8A |  |
|  | KLK13 |  |
|  | ASAP3 |  |
|  | CILP |  |
|  | C12orf54 |  |
|  | ALKBH3-AS1 |  |
|  | EIF2B5-IT1 |  |
|  | OTTHUMG00000152794 |  |
|  | IGHV3OR16-7 |  |
|  | OTTHUMG00000157289 |  |
|  | LOC100507140 |  |
|  | FAM47C |  |
|  | OTTHUMG00000153821 |  |
|  | TNN |  |
|  | GALNT8 |  |
|  | COL2A1 |  |
|  | CAMKV |  |
|  | CHADL |  |
|  | CSN1S1 |  |
|  | CELA3A |  |
|  | CAPN14 |  |
|  | CABP1 |  |
|  | C12orf42 |  |
|  | STRA6 |  |
|  | FSD2 |  |
|  | OTTHUMG00000007304 |  |
|  | C1QTNF6 |  |
|  | OTTHUMG00000015439 |  |
|  | OTTHUMG00000009688 |  |
|  | LINC00906 |  |
|  | KCNK7 |  |
|  | TMC2 |  |
|  | OTTHUMG00000156627 |  |
|  | MYH15 |  |
|  | OTTHUMG00000159357 |  |
|  | CSMD2 |  |
|  | OTTHUMG00000164394 |  |
|  | OTTHUMG00000165032 |  |
|  | FNDC7 |  |
|  | SPATA42 |  |
|  | PAEP |  |
|  | PRH2 |  |
|  | ZFHX2 |  |
|  | KCNJ6 |  |
|  | LINC00841 |  |
|  | POU2AF1 |  |
|  | OSTCP1 |  |
|  | SEMA6D |  |
|  | LOC80154 |  |
|  | LINC00929 |  |
|  | OTTHUMG00000155482 |  |
|  | CLDN14 |  |
|  | C7orf65 |  |
|  | OTTHUMG00000018612 |  |
|  | PTPN20C |  |
|  | FOXP1-AS1 |  |
|  | OTTHUMG00000015628 |  |
|  | OTTHUMG00000040861 |  |
|  | SLC10A6 |  |
|  | KIF2B |  |
|  | HSPB7 |  |
|  | LOC643837 |  |
|  | PTGIS |  |
|  | OTTHUMG00000041231 |  |
|  | SAMD10 |  |
|  | LOC100144595 |  |
|  | CHODL-AS1 |  |
|  | MUC15 |  |
|  | ODAM |  |
|  | PAX4 |  |
|  | LINC00652 |  |
|  | CNTNAP3B |  |
|  | HEPACAM2 |  |
|  | PHF2P1 |  |
|  | TH |  |
|  | BCMO1 |  |
|  | OTTHUMG00000024201 |  |
|  | LINC00669 |  |
|  | LOC728606 |  |
|  | OTTHUMG00000039470 |  |
|  | OTTHUMG00000159849 |  |
|  | PLP1 |  |
|  | C3P1 |  |
|  | OTTHUMG00000003875 |  |
|  | OTTHUMG00000164938 |  |
|  | KLRG2 |  |
|  | OTTHUMG00000158297 |  |
|  | LOC100128554 |  |
|  | C6orf10 |  |
|  | LINC00598 |  |
|  | SPATA3 |  |
|  | CACNA1I |  |
|  | OTTHUMG00000163792 |  |
|  | MOCOS |  |
|  | LOC388813 |  |
|  | UNC5D |  |
|  | RYR3 |  |
|  | OTTHUMG00000159627 |  |
|  | ANTXRL |  |
|  | C17orf50 |  |
|  | SORCS1 |  |
|  | OTTHUMG00000154835 |  |
|  | OTTHUMG00000086642 |  |
|  | LGALS4 |  |
|  | ADAMTS13 |  |
|  | OTTHUMG00000162152 |  |
|  | SERPINB5 |  |
|  | CCL17 |  |
|  | KCNH2 |  |
|  | CGNL1 |  |
|  | PRKAR2A-AS1 |  |
|  | OTTHUMG00000152676 |  |
|  | GOLGA6D |  |
|  | OTTHUMG00000160824 |  |
|  | OTTHUMG00000160142 |  |
|  | CDHR4 |  |
|  | SULT1E1 |  |
|  | MIR4522 |  |
|  | LOC100130264 |  |
|  | PPP1R3C |  |
|  | VWA8-AS1 |  |
|  | OTTHUMG00000162821 |  |
|  | OTTHUMG00000035526 |  |
|  | MTNR1B |  |
|  | ATP13A4 |  |
|  | OTTHUMG00000015227 |  |
|  | OR2K2 |  |
|  | MIRLET7DHG |  |
|  | C19orf26 |  |
|  | GSTT1 |  |
|  | OTTHUMG00000161964 |  |
|  | LINC00658 |  |
|  | RNA5SP102 |  |
|  | TRIM51GP |  |
|  | RLN3 |  |
|  | IL20RB-AS1 |  |
|  | OR7D1P |  |
|  | LOC91450 |  |
|  | SNORD113-7 |  |
|  | OTTHUMG00000158384 |  |
|  | SYCN |  |
|  | CETP |  |
|  | OTTHUMG00000153125 |  |
|  | ANKRD24 |  |
|  | OTTHUMG00000037230 |  |
|  | SLC39A5 |  |
|  | OTTHUMG00000164073 |  |
|  | LINC00492 |  |
|  | LOC100507447 |  |
|  | PADI1 |  |
|  | MIR548F1 |  |
|  | ZG16 |  |
|  | THRB-AS1 |  |
|  | GRM6 |  |
|  | RNF157-AS1 |  |
|  | NALCN |  |
|  | OTTHUMG00000155816 |  |
|  | EFR3B |  |
|  | CIDEC |  |
|  | OTTHUMG00000014842 |  |
|  | LAMB4 |  |
|  | OTTHUMG00000153231 |  |
|  | MIR150 |  |
|  | SLC8A3 |  |
|  | MYH1 |  |
|  | OTTHUMG00000155554 |  |
|  | GAMT |  |
|  | TRIM49C |  |
|  | MURC |  |
|  | C19orf57 |  |
|  | C6orf223 |  |
|  | CTAGE11P |  |
|  | KRT16P2 |  |
|  | LRP11 |  |
|  | OTTHUMG00000015618 |  |
|  | WFDC2 |  |
|  | OTTHUMG00000022550 |  |
|  | MROH5 |  |
|  | OR5B12 |  |
|  | SPESP1 |  |
|  | OTTHUMG00000031991 |  |
|  | SPSB4 |  |
|  | KNCN |  |
|  | CPS1 |  |
|  | GLI3 |  |
|  | LOC100133251 |  |
|  | OTTHUMG00000132736 |  |
|  | LOC650226 |  |
|  | PDZK1P1 |  |
|  | G6PC |  |
|  | LINC00202-2 |  |
|  | KCNN3 |  |
|  | SGIP1 |  |
|  | CRHR2 |  |
|  | KIF7 |  |
|  | CLUL1 |  |
|  | ACSBG1 |  |
|  | OTTHUMG00000161349 |  |
|  | PRICKLE2 |  |
|  | OTTHUMG00000161311 |  |
|  | OTTHUMG00000151395 |  |
|  | SLIT2 |  |
|  | OTTHUMG00000017482 |  |
|  | SDC1 |  |
|  | DKFZP434K028 |  |
|  | OTTHUMG00000016839 |  |
|  | PDX1-AS1 |  |
|  | KRT15 |  |
|  | AQP7 |  |
|  | LOC100505550 |  |
|  | COL4A1 |  |
|  | MUC19 |  |
|  | PODNL1 |  |
|  | OTTHUMG00000164826 |  |
|  | SLC22A10 |  |
|  | SYCE2 |  |
|  | CCDC24 |  |
|  | CCDC78 |  |
|  | SAMD12-AS1 |  |
|  | OTTHUMG00000033066 |  |
|  | WNK2 |  |
|  | PLA2G5 |  |
|  | OTTHUMG00000016052 |  |
|  | OTTHUMG00000163319 |  |
|  | TTYH1 |  |
|  | OTTHUMG00000144164 |  |
|  | OTTHUMG00000150948 |  |
|  | RNA5SP133 |  |
|  | MESP2 |  |
|  | C2CD4A |  |
|  | TRAM1L1 |  |
|  | OTTHUMG00000008243 |  |
|  | LOC339788 |  |
|  | OTTHUMG00000164253 |  |
|  | OR6C74 |  |
|  | LTBP2 |  |
|  | OTTHUMG00000163740 |  |
|  | OTTHUMG00000162185 |  |
|  | MYOZ2 |  |
|  | RNA5SP255 |  |
|  | PRSS3 |  |
|  | LOC283914 |  |
|  | PCSK9 |  |
|  | SMO |  |
|  | ECM1 |  |
|  | OTTHUMG00000037355 |  |
|  | CCT8L2 |  |
|  | AK8 |  |
|  | KRT8P11 |  |
|  | OTTHUMG00000001766 |  |
|  | OTTHUMG00000162107 |  |
|  | LOC100130964 |  |
|  | UGT1A5 |  |
|  | DSCR8 |  |
|  | SLC26A9 |  |
|  | MIR373 |  |
|  | SLC22A23 |  |
|  | GRIK5 |  |
|  | LOC101060082 |  |
|  | LCT |  |
|  | LOC440905 |  |
|  | DNAH3 |  |
|  | RNA5SP20 |  |
|  | TACC2 |  |
|  | LOC283575 |  |
|  | RNA5SP480 |  |
|  | SNORD115-7 |  |
|  | MASP2 |  |
|  | SLC6A1 |  |
|  | LOC101060483 |  |
|  | CCDC155 |  |
|  | OTTHUMG00000153765 |  |
|  | TLE6 |  |
|  | MAMSTR |  |
|  | KIF25-AS1 |  |
|  | HNF1B |  |
|  | TAGLN3 |  |
|  | OTTHUMG00000158859 |  |
|  | DCDC5 |  |
|  | PCDH1 |  |
|  | PLXNB3 |  |
|  | LRFN5 |  |
|  | RNA5SP499 |  |
|  | OTTHUMG00000161757 |  |
|  | MMP8 |  |
|  | FKBP9L |  |
|  | MMRN2 |  |
|  | CCKAR |  |
|  | MOXD2P |  |
|  | SSC5D |  |
|  | RASGRF1 |  |
|  | ALDH1L1 |  |
|  | NRCAM |  |
|  | LOC100506446 |  |
|  | SEZ6L2 |  |
|  | OTTHUMG00000019095 |  |
|  | OTTHUMG00000032760 |  |
|  | TERT |  |
|  | ANGPTL2 |  |
|  | C7 |  |
|  | MAGEA2B |  |
|  | OTTHUMG00000161479 |  |
|  | OTTHUMG00000164192 |  |
|  | SPACA7 |  |
|  | LOC100288846 |  |
|  | LRRN4 |  |
|  | FLJ35282 |  |
|  | OTTHUMG00000019566 |  |
|  | OTTHUMG00000155309 |  |
|  | ZNF541 |  |
|  | KIR2DL4 |  |
|  | PRSS53 |  |
|  | C21orf49 |  |
|  | OTTHUMG00000160229 |  |
|  | PDGFRA |  |
|  | PEX5L-AS1 |  |
|  | CNTFR-AS1 |  |
|  | MYH8 |  |
|  | TMPRSS5 |  |
|  | LOC100506272 |  |
|  | FBXO2 |  |
|  | FAT2 |  |
|  | OTTHUMG00000162382 |  |
|  | ARSF |  |
|  | LINC00886 |  |
|  | LOC729224 |  |
|  | MUC5AC |  |
|  | CRCT1 |  |
|  | ASIC4 |  |
|  | KCND3 |  |
|  | OTTHUMG00000014661 |  |
|  | MYOCD |  |
|  | OTTHUMG00000015617 |  |
|  | LOC349160 |  |
|  | GCNT7 |  |
|  | LINC00444 |  |
|  | GLYATL1 |  |
|  | SLC28A2 |  |
|  | KRT24 |  |
|  | LINC00676 |  |
|  | NPNT |  |
|  | OTTHUMG00000153514 |  |
|  | OTTHUMG00000032669 |  |
|  | OTTHUMG00000155346 |  |
|  | KIF26A |  |
|  | OTTHUMG00000151130 |  |
|  | FBN3 |  |
|  | OTTHUMG00000150375 |  |
|  | GALNT15 |  |
|  | FAM163A |  |
|  | TNNI2 |  |
|  | OTTHUMG00000163123 |  |
|  | SLC28A1 |  |
|  | FRAS1 |  |
|  | TMPRSS11E |  |
|  | CLDN10 |  |
|  | GPR6 |  |
|  | SLC34A3 |  |
|  | KIAA1239 |  |
|  | RNA5SP194 |  |
|  | OTTHUMG00000150320 |  |
|  | LOC100996876 |  |
|  | KIF12 |  |
|  | CLDN4 |  |
|  | SH2D4B |  |
|  | OTTHUMG00000164480 |  |
|  | CASP16 |  |
|  | OTTHUMG00000160585 |  |
|  | OTTHUMG00000003927 |  |
|  | LDLRAD2 |  |
|  | OTTHUMG00000014234 |  |
|  | OTTHUMG00000161336 |  |
|  | PITRM1-AS1 |  |
|  | OTTHUMG00000000565 |  |
|  | VWA5B2 |  |
|  | KRT35 |  |
|  | CGB8 |  |
|  | OTTHUMG00000161780 |  |
|  | MTUS1 |  |
|  | CACNA1S |  |
|  | TEAD1 |  |
|  | ST7-AS2 |  |
|  | GCGR |  |
|  | PTOV1-AS1 |  |
|  | OTTHUMG00000039495 |  |
|  | OTTHUMG00000002202 |  |
|  | OTTHUMG00000151378 |  |
|  | GJA1 |  |
|  | STMN3 |  |
|  | OR10J1 |  |
|  | GSTA7P |  |
|  | MYC |  |
|  | LOC100506175 |  |
|  | C3orf65 |  |
|  | C2orf27A |  |
|  | RMST |  |
|  | HIP1R |  |
|  | HNRNPCP5 |  |
|  | OTTHUMG00000022377 |  |
|  | TMEM26 |  |
|  | WNT10B |  |
|  | TSNAXIP1 |  |
|  | SSPO |  |
|  | OTTHUMG00000163220 |  |
|  | ILDR1 |  |
|  | MIR4776-1 |  |
|  | OTTHUMG00000000613 |  |
|  | ALDH1A3 |  |
|  | PTN |  |
|  | MAGEA3 |  |
|  | GPR142 |  |
|  | OTTHUMG00000014256 |  |
|  | TFAP2D |  |
|  | PRSS41 |  |
|  | OR52B4 |  |
|  | KRT16 |  |
|  | OTTHUMG00000009742 |  |
|  | EXTL1 |  |
|  | CYP4X1 |  |
|  | LOC284600 |  |
|  | OTTHUMG00000163132 |  |
|  | NRG1-IT3 |  |
|  | NPSR1 |  |
|  | GABRA1 |  |
|  | IGKV6D-41 |  |
|  | PRDM7 |  |
|  | CYP21A2 |  |
|  | LOC100505609 |  |
|  | OTTHUMG00000162969 |  |
|  | OTTHUMG00000163990 |  |
|  | FAM171A1 |  |
|  | SLC22A2 |  |
|  | PEBP4 |  |
|  | CYP1A2 |  |
|  | OTTHUMG00000151357 |  |
|  | OTTHUMG00000155749 |  |
|  | C10orf120 |  |
|  | HSD17B2 |  |
|  | CPB2-AS1 |  |
|  | OTTHUMG00000008402 |  |
|  | OTTHUMG00000020614 |  |
|  | LINC00035 |  |
|  | IL17A |  |
|  | LOC100506004 |  |
|  | OTTHUMG00000040635 |  |
|  | C6 |  |
|  | HMGCLL1 |  |
|  | EGFR |  |
|  | LOC100507461 |  |
|  | TMEM132D |  |
|  | PDCD4-AS1 |  |
|  | SPDEF |  |
|  | CDT1 |  |
|  | OTTHUMG00000164139 |  |
|  | LOC339593 |  |
|  | OTTHUMG00000150565 |  |
|  | FAM155A |  |
|  | RPS6KL1 |  |
|  | OTTHUMG00000158687 |  |
|  | SLC12A8 |  |
|  | HNF4G |  |
|  | ACTRT2 |  |
|  | MTMR7 |  |
|  | OTTHUMG00000018829 |  |
|  | GSTO2 |  |
|  | CHAC1 |  |
|  | OTTHUMG00000002710 |  |
|  | FSTL4 |  |
|  | LINC00371 |  |
|  | CAP2 |  |
|  | C17orf51 |  |
|  | PRR23C |  |
|  | LOC100506216 |  |
|  | LOC100499183 |  |
|  | SLC16A12 |  |
|  | SULT2A1 |  |
|  | OTTHUMG00000159327 |  |
|  | OTTHUMG00000161216 |  |
|  | LRRC71 |  |
|  | MIR604 |  |
|  | CES3 |  |
|  | OTTHUMG00000014792 |  |
|  | OTTHUMG00000155365 |  |
|  | FAM83C |  |
|  | FAM197Y9 |  |
|  | VGLL1 |  |
|  | OCM2 |  |
|  | LOC100507511 |  |
|  | MFSD2B |  |
|  | CLEC18C |  |
|  | LOC100505964 |  |
|  | CPLX3 |  |
|  | OTTHUMG00000155853 |  |
|  | OTTHUMG00000153173 |  |
|  | OTTHUMG00000152490 |  |
|  | OR8G5 |  |
|  | OTTHUMG00000155145 |  |
|  | IGHV3-43 |  |
|  | KCNK16 |  |
|  | TDH |  |
|  | OTTHUMG00000017877 |  |
|  | OTTHUMG00000059638 |  |
|  | MBD3L5 |  |
|  | MIR4435-1 |  |
|  | LINC00521 |  |
|  | FAM132A |  |
|  | ADORA2A-AS1 |  |
|  | SHROOM2 |  |
|  | ZNF229 |  |
|  | C1orf87 |  |
|  | LRRC3-AS1 |  |
|  | TMEM54 |  |
|  | OTTHUMG00000163567 |  |
|  | OTTHUMG00000155196 |  |
|  | STMN4 |  |
|  | DSCR9 |  |
|  | NYAP2 |  |
|  | KRTAP22-1 |  |
|  | SPATS1 |  |
|  | SFTPC |  |
|  | OR13C4 |  |
|  | ARSH |  |
|  | FBXO40 |  |
|  | FXYD7 |  |
|  | OTTHUMG00000160206 |  |
|  | LINC00633 |  |
|  | TRPV5 |  |
|  | GUCY2EP |  |
|  | ANKRD66 |  |
|  | GRAMD2 |  |
|  | MAB21L3 |  |
|  | CABYR |  |
|  | OTTHUMG00000162186 |  |
|  | OTTHUMG00000150321 |  |
|  | OTTHUMG00000017565 |  |
|  | OTTHUMG00000159698 |  |
|  | LRRN2 |  |
|  | OTTHUMG00000045405 |  |
|  | CYP11B1 |  |
|  | OTTHUMG00000156608 |  |
|  | OTTHUMG00000153975 |  |
|  | AZI1 |  |
|  | STYK1 |  |
|  | OTTHUMG00000157300 |  |
|  | C6orf52 |  |
|  | DKFZP434A062 |  |
|  | NEU2 |  |
|  | ALK |  |
|  | SLC35G6 |  |
|  | OTTHUMG00000163153 |  |
|  | CLDN12 |  |
|  | OTTHUMG00000162785 |  |
|  | BAIAP3 |  |
|  | GPR125 |  |
|  | OTTHUMG00000156955 |  |
|  | OTTHUMG00000014191 |  |
|  | LTF |  |
|  | SOX30 |  |
|  | PTH2R |  |
|  | KLK9 |  |
|  | MIR508 |  |
|  | ARHGEF38-IT1 |  |
|  | TACR1 |  |
|  | OTTHUMG00000151726 |  |
|  | OTTHUMG00000003064 |  |
|  | MEGF10 |  |
|  | HRH1 |  |
|  | GJB7 |  |
|  | OTTHUMG00000161604 |  |
|  | RGS8 |  |
|  | FRMD6 |  |
|  | MS4A3 |  |
|  | APBB2 |  |
|  | OTTHUMG00000155983 |  |
|  | KRT42P |  |
|  | OTTHUMG00000160396 |  |
|  | CCDC153 |  |
|  | MPDZ |  |
|  | OR4C16 |  |
|  | LOC100507431 |  |
|  | OTTHUMG00000017921 |  |
|  | OTTHUMG00000014341 |  |
|  | SNORD114-24 |  |
|  | OTTHUMG00000152910 |  |
|  | OTTHUMG00000163136 |  |
|  | MAGI2-AS1 |  |
|  | CACNA1B |  |
|  | ADCY8 |  |
|  | TEX26-AS1 |  |
|  | SYT6 |  |
|  | OTTHUMG00000164320 |  |
|  | LINC00853 |  |
|  | LOC400620 |  |
|  | GRIA1 |  |
|  | TENM2 |  |
|  | P2RX3 |  |
|  | ARMC12 |  |
|  | TRIM71 |  |
|  | OTTHUMG00000017631 |  |
|  | NPHS2 |  |
|  | GIPC3 |  |
|  | OTTHUMG00000156385 |  |
|  | TMEM25 |  |
|  | PRRT1 |  |
|  | ZRANB2-AS2 |  |
|  | DDR2 |  |
|  | OTTHUMG00000007703 |  |
|  | WNK3 |  |
|  | MAFG-AS1 |  |
|  | DRD3 |  |
|  | WNT3 |  |
|  | OTTHUMG00000159723 |  |
|  | RNU6-76P |  |
|  | GPRASP2 |  |
|  | STK32A |  |
|  | ZP4 |  |
|  | ADAMTS3 |  |
|  | DCAF8L1 |  |
|  | TTTY21 |  |
|  | TTTY21B |  |
|  | TTLL13 |  |
|  | OTTHUMG00000020285 |  |
|  | C10orf53 |  |
|  | OTTHUMG00000149890 |  |
|  | GRB7 |  |
|  | OTOA |  |
|  | IFNB1 |  |
|  | OTTHUMG00000048120 |  |
|  | OTTHUMG00000150953 |  |
|  | OTTHUMG00000163849 |  |
|  | C10orf113 |  |
|  | RNA5SP477 |  |
|  | MUC4 |  |
|  | RNA5SP22 |  |
|  | LINC00475 |  |
|  | SLURP1 |  |
|  | CYP4Z1 |  |
|  | DUOXA1 |  |
|  | DDI1 |  |
|  | C1QTNF5 |  |
|  | MIR205 |  |
|  | PLA2G3 |  |
|  | CELA2B |  |
|  | HRC |  |
|  | LOC149134 |  |
|  | OTTHUMG00000161471 |  |
|  | OR52D1 |  |
|  | RNF144A-AS1 |  |
|  | PAPPA |  |
|  | MAT1A |  |
|  | OTTHUMG00000155426 |  |
|  | SLCO2B1 |  |
|  | KANK2 |  |
|  | OTTHUMG00000161622 |  |
|  | SLC5A8 |  |
|  | HPYR1 |  |
|  | MYLK-AS1 |  |
|  | CASP14 |  |
|  | PLCXD3 |  |
|  | OR6V1 |  |
|  | FAM71F1 |  |
|  | CYP7A1 |  |
|  | IGHA2 |  |
|  | LOC100996474 |  |
|  | SYT14L |  |
|  | WDR16 |  |
|  | PYGO1 |  |
|  | LOC344967 |  |
|  | LOC101059935 |  |
|  | KLK10 |  |
|  | KCNIP1 |  |
|  | ZNF658 |  |
|  | LINC00473 |  |
|  | OTTHUMG00000160654 |  |
|  | OTTHUMG00000036359 |  |
|  | TMC7 |  |
|  | TRIM64 |  |
|  | SLC30A2 |  |
|  | CSRP2 |  |
|  | C7orf62 |  |
|  | OTTHUMG00000160119 |  |
|  | KRT8 |  |
|  | LCE2D |  |
|  | MYADML |  |
|  | GSTA1 |  |
|  | MTRNR2L6 |  |
|  | OTTHUMG00000040015 |  |
|  | CSMD1 |  |
|  | BCAR3 |  |
|  | OTTHUMG00000162543 |  |
|  | MCF2L |  |
|  | PGCP1 |  |
|  | FAM197Y3 |  |
|  | AGBL5-IT1 |  |
|  | HEXIM2 |  |
|  | TSGA10IP |  |
|  | AMBP |  |
|  | ALAS2 |  |
|  | MIR675 |  |
|  | LY6D |  |
|  | OTTHUMG00000037511 |  |
|  | KIR3DL2 |  |
|  | OTTHUMG00000164046 |  |
|  | OTTHUMG00000017971 |  |
|  | CDK15 |  |
|  | HPN |  |
|  | AGBL4 |  |
|  | RNF144A |  |
|  | ATP6V1B1 |  |
|  | FER1L6 |  |
|  | OTTHUMG00000154100 |  |
|  | OTTHUMG00000161880 |  |
|  | OTTHUMG00000162122 |  |
|  | OTTHUMG00000151806 |  |
|  | PARK2 |  |
|  | MS4A8 |  |
|  | OTTHUMG00000032047 |  |
|  | CDH5 |  |
|  | TPO |  |
|  | MIR3160-2 |  |
|  | OTTHUMG00000002292 |  |
|  | LINC00705 |  |
|  | OTTHUMG00000035545 |  |
|  | OTTHUMG00000163128 |  |
|  | OTTHUMG00000018783 |  |
|  | FRMD7 |  |
|  | RNA5SP171 |  |
|  | RD3L |  |
|  | OTTHUMG00000018280 |  |
|  | OTTHUMG00000017697 |  |
|  | MAGEB17 |  |
|  | C8G |  |
|  | SNORD114-4 |  |
|  | FAM43B |  |
|  | FAM71E1 |  |
|  | LINC00594 |  |
|  | OBP2B |  |
|  | OTTHUMG00000155236 |  |
|  | SLC13A2 |  |
|  | KRTAP27-1 |  |
|  | OTTHUMG00000157729 |  |
|  | OTTHUMG00000022612 |  |
|  | OTTHUMG00000011701 |  |
|  | FGF9 |  |
|  | LOC642423 |  |
|  | OTTHUMG00000163476 |  |
|  | OTTHUMG00000162331 |  |
|  | OTTHUMG00000148948 |  |
|  | STXBP1 |  |
|  | OTTHUMG00000160896 |  |
|  | OTTHUMG00000163993 |  |
|  | LRRC16B |  |
|  | INSC |  |
|  | OTTHUMG00000161643 |  |
|  | OR5AS1 |  |
|  | SYP-AS1 |  |
|  | OTTHUMG00000161801 |  |
|  | FGF1 |  |
|  | SLC17A6 |  |
|  | CCKBR |  |
|  | OTTHUMG00000019968 |  |
|  | OCA2 |  |
|  | HCAR1 |  |
|  | KCNH1 |  |
|  | OTTHUMG00000153616 |  |
|  | OTTHUMG00000020400 |  |
|  | GNRH2 |  |
|  | GLI2 |  |
|  | OTTHUMG00000156119 |  |
|  | OTTHUMG00000008768 |  |
|  | OTTHUMG00000155685 |  |
|  | OTTHUMG00000058152 |  |
|  | LINC00334 |  |
|  | FLJ44881 |  |
|  | OTTHUMG00000134300 |  |
|  | SGCG |  |
|  | CADM2 |  |
|  | C1orf116 |  |
|  | DSCAM |  |
|  | OTTHUMG00000002047 |  |
|  | ACSM4 |  |
|  | TSPAN7 |  |
|  | SENP8 |  |
|  | SPATA31E1 |  |
|  | FOLH1B |  |
|  | SERPINA3 |  |
|  | C3orf79 |  |
|  | MLANA |  |
|  | OTTHUMG00000019752 |  |
|  | PDZK1 |  |
|  | OTTHUMG00000090768 |  |
|  | LINC00856 |  |
|  | SERTM1 |  |
|  | PROM2 |  |
|  | OTTHUMG00000020611 |  |
|  | FLJ26245 |  |
|  | ATG9B |  |
|  | DAW1 |  |
|  | OTTHUMG00000160300 |  |
|  | OTTHUMG00000163480 |  |
|  | DDAH1 |  |
|  | KRT4 |  |
|  | NRK |  |
|  | BTNL8 |  |
|  | PDILT |  |
|  | MIR4288 |  |
|  | ZNF582-AS1 |  |
|  | SYNDIG1 |  |
|  | ATP6V1G2 |  |
|  | PDE6G |  |
|  | HMP19 |  |
|  | DLGAP1-AS3 |  |
|  | OTTHUMG00000163393 |  |
|  | RNA5SP406 |  |
|  | PRSS46 |  |
|  | OTTHUMG00000036405 |  |
|  | COL4A3 |  |
|  | LOC100652782 |  |
|  | VRTN |  |
|  | MACC1-AS1 |  |
|  | OTTHUMG00000017769 |  |
|  | OTTHUMG00000154683 |  |
|  | C20orf181 |  |
|  | OTTHUMG00000155764 |  |
|  | ZSCAN5B |  |
|  | FSHR |  |
|  | OTTHUMG00000015538 |  |
|  | CDX4 |  |
|  | TFAP2B |  |
|  | B4GALNT1 |  |
|  | UNQ6975 |  |
|  | TEKT2 |  |
|  | LINC00277 |  |
|  | OTTHUMG00000132235 |  |
|  | COL20A1 |  |
|  | OTTHUMG00000151734 |  |
|  | ANKRD20A8P |  |
|  | SMPDL3B |  |
|  | MIR548H4 |  |
|  | LINC00345 |  |
|  | MIR4514 |  |
|  | LOC728763 |  |
|  | CCL27 |  |
|  | HUNK |  |
|  | KCNK2 |  |
|  | SHANK2 |  |
|  | S100A16 |  |
|  | C9orf135-AS1 |  |
|  | GRM5 |  |
|  | TMCO2 |  |
|  | MIMT1 |  |
|  | LOC100268168 |  |
|  | PLA2G1B |  |
|  | OTTHUMG00000163764 |  |
|  | PRL |  |
|  | RFPL2 |  |
|  | OTTHUMG00000032989 |  |
|  | SLC9A4 |  |
|  | TNS4 |  |
|  | AGRP |  |
|  | FAM110B |  |
|  | MYOC |  |
|  | OTOP1 |  |
|  | TUSC5 |  |
|  | OTTHUMG00000160651 |  |
|  | LINC00423 |  |
|  | OR4K5 |  |
|  | MIR1247 |  |
|  | OTTHUMG00000019246 |  |
|  | KRT81 |  |
|  | OTTHUMG00000161599 |  |
|  | SCNN1G |  |
|  | ACSM5 |  |
|  | OTTHUMG00000156951 |  |
|  | OTTHUMG00000066679 |  |
|  | CGB5 |  |
|  | OTTHUMG00000155312 |  |
|  | PRG4 |  |
|  | OTTHUMG00000160040 |  |
|  | PRTG |  |
|  | RGS4 |  |
|  | WISP3 |  |
|  | PDGFRL |  |
|  | CRYM |  |
|  | DACT3-AS1 |  |
|  | R3HDML |  |
|  | VTCN1 |  |
|  | FRMPD2 |  |
|  | OTTHUMG00000020126 |  |
|  | BBOX1 |  |
|  | ADAMTS14 |  |
|  | LOC340357 |  |
|  | NLRP8 |  |
|  | FLJ31356 |  |
|  | GABRA6 |  |
|  | KRTAP19-8 |  |
|  | OTTHUMG00000159404 |  |
|  | OTTHUMG00000163868 |  |
|  | CPB1 |  |
|  | MIR138-2 |  |
|  | LOC401164 |  |
|  | OTTHUMG00000162113 |  |
|  | UCMA |  |
|  | STMND1 |  |
|  | OTTHUMG00000065062 |  |
|  | RNA5SP45 |  |
|  | OTTHUMG00000017736 |  |
|  | CNNM1 |  |
|  | MIR520G |  |
|  | OTTHUMG00000151427 |  |
|  | NBEA |  |
|  | KNG1 |  |
|  | OTTHUMG00000037843 |  |
|  | ASPHD1 |  |
|  | COL4A4 |  |
|  | SLC9A5 |  |
|  | LOC644070 |  |
|  | MACROD2-IT1 |  |
|  | SLC24A2 |  |
|  | OTTHUMG00000066439 |  |
|  | KRT8P41 |  |
|  | OTTHUMG00000162954 |  |
|  | PAX3 |  |
|  | LIX1 |  |
|  | FXYD1 |  |
|  | OTTHUMG00000163055 |  |
|  | OTTHUMG00000031529 |  |
|  | OTTHUMG00000154871 |  |
|  | LOC116437 |  |
|  | FAM86HP |  |
|  | IGDCC3 |  |
|  | LINC00704 |  |
|  | C11orf93 |  |
|  | LOC402779 |  |
|  | C9orf24 |  |
|  | LOC100287177 |  |
|  | TRIM64DP |  |
|  | MIR3936 |  |
|  | OTTHUMG00000150049 |  |
|  | LAMA3 |  |
|  | LOC100507464 |  |
|  | CIB3 |  |
|  | EPS8L3 |  |
|  | OTTHUMG00000164015 |  |
|  | MIR3591 |  |
|  | TBX10 |  |
|  | DCHS1 |  |
|  | MIR557 |  |
|  | OTTHUMG00000151748 |  |
|  | OTTHUMG00000164870 |  |
|  | FABP6 |  |
|  | XKRX |  |
|  | OTTHUMG00000013955 |  |
|  | GAL3ST1 |  |
|  | ADAMTS12 |  |
|  | HTR2C-IT1 |  |
|  | FLJ33581 |  |
|  | TMEM92 |  |
|  | BPIFB2 |  |
|  | CCL1 |  |
|  | PTCHD3 |  |
|  | C1orf51 |  |
|  | MYRIP |  |
|  | OTTHUMG00000156244 |  |
|  | PITX2 |  |
|  | WDR87 |  |
|  | CLDN5 |  |
|  | OTTHUMG00000163982 |  |
|  | SLC25A30-AS1 |  |
|  | OTTHUMG00000020901 |  |
|  | GLYAT |  |
|  | PAPPA-AS1 |  |
|  | IDO2 |  |
|  | ACSM2A |  |
|  | UNC93A |  |
|  | LOC101060019 |  |
|  | CTSG |  |
|  | OR7E5P |  |
|  | RELN |  |
|  | LOC285419 |  |
|  | OTTHUMG00000013293 |  |
|  | OTTHUMG00000163015 |  |
|  | GPR1 |  |
|  | OTTHUMG00000151464 |  |
|  | TMPRSS12 |  |
|  | PRICKLE2-AS2 |  |
|  | OTTHUMG00000158130 |  |
|  | MIR181D |  |
|  | PALMD |  |
|  | BIRC7 |  |
|  | MIR30C2 |  |
|  | C9orf131 |  |
|  | KCNJ11 |  |
|  | CDIPT-AS1 |  |
|  | OTTHUMG00000164945 |  |
|  | AGBL1-AS1 |  |
|  | LOC100507652 |  |
|  | OTTHUMG00000157379 |  |
|  | OTTHUMG00000159112 |  |
|  | SPP2 |  |
|  | LRRTM2 |  |
|  | LRP2 |  |
|  | CPT1C |  |
|  | CYorf17 |  |
|  | NOBOX |  |
|  | OTTHUMG00000163707 |  |
|  | LCN1 |  |
|  | OTTHUMG00000164833 |  |
|  | MEGF11 |  |
|  | OTTHUMG00000012022 |  |
|  | PGLYRP4 |  |
|  | FAM211B |  |
|  | OTTHUMG00000162459 |  |
|  | LINC00635 |  |
|  | LDLRAD4-AS1 |  |
|  | OTTHUMG00000149883 |  |
|  | DUSP13 |  |
|  | ARHGEF7-AS2 |  |
|  | COL4A5 |  |
|  | OTTHUMG00000032765 |  |
|  | OTTHUMG00000156135 |  |
|  | OTTHUMG00000015322 |  |
|  | CELA3B |  |
|  | C12orf77 |  |
|  | LOC100996291 |  |
|  | C4orf6 |  |
|  | OTTHUMG00000037290 |  |
|  | GAL3ST2 |  |
|  | CXCL6 |  |
|  | SMIM2 |  |
|  | CACNG5 |  |
|  | OTTHUMG00000036746 |  |
|  | OR10D3 |  |
|  | OTTHUMG00000021054 |  |
|  | OTTHUMG00000031891 |  |
|  | CYP2G1P |  |
|  | RNA5SP415 |  |
|  | OTTHUMG00000133761 |  |
|  | OTTHUMG00000032808 |  |
|  | OTTHUMG00000015347 |  |
|  | RNA5SP240 |  |
|  | GALNT16 |  |
|  | FLJ11235 |  |
|  | ZAN |  |
|  | LOC101060277 |  |
|  | WFIKKN2 |  |
|  | AKR1E2 |  |
|  | OTTHUMG00000074100 |  |
|  | FAM3D |  |
|  | HID1 |  |
|  | TRPC4 |  |
|  | OTTHUMG00000012147 |  |
|  | IGHV3-73 |  |
|  | OTTHUMG00000162289 |  |
|  | RNA5SP346 |  |
|  | C6orf141 |  |
|  | TRMT2B-AS1 |  |
|  | CAMK2A |  |
|  | GNG12 |  |
|  | FLJ14082 |  |
|  | OTTHUMG00000037789 |  |
|  | MAGEA11 |  |
|  | OTTHUMG00000164855 |  |
|  | OTTHUMG00000158357 |  |
|  | LOC100506229 |  |
|  | OSBPL10 |  |
|  | COL16A1 |  |
|  | IGLV5-48 |  |
|  | TREML4 |  |
|  | OTTHUMG00000163964 |  |
|  | DNAH9 |  |
|  | OTTHUMG00000162055 |  |
|  | OTTHUMG00000164089 |  |
|  | BPIFA1 |  |
|  | APOOP5 |  |
|  | CCDC13-AS1 |  |
|  | OTTHUMG00000164986 |  |
|  | CLDN24 |  |
|  | DKKL1 |  |
|  | OTTHUMG00000162282 |  |
|  | OTTHUMG00000004419 |  |
|  | RIMKLA |  |
|  | NOVA1-AS1 |  |
|  | OTTHUMG00000151815 |  |
|  | CRYBA4 |  |
|  | LOC100506393 |  |
|  | TLCD2 |  |
|  | LINC00229 |  |
|  | GLYATL3 |  |
|  | C5orf46 |  |
|  | NBPF5P |  |
|  | ACOX2 |  |
|  | OR6B1 |  |
|  | SPATA8 |  |
|  | MIR3150A |  |
|  | AACSP1 |  |
|  | PTPRR |  |
|  | OTTHUMG00000015300 |  |
|  | FCRL5 |  |
|  | HAS1 |  |
|  | OTTHUMG00000163719 |  |
|  | SNTG2 |  |
|  | OTTHUMG00000016605 |  |
|  | MIR4489 |  |
|  | BPIFB4 |  |
|  | DEFB131 |  |
|  | OTTHUMG00000024091 |  |
|  | HOGA1 |  |
|  | TPTE2P3 |  |
|  | SPINK6 |  |
|  | CRYAB |  |
|  | OR10V1 |  |
|  | NOV |  |
|  | RNU7-2P |  |
|  | OTTHUMG00000162741 |  |
|  | OBSCN |  |
|  | NEURL3 |  |
|  | OTTHUMG00000010701 |  |
|  | CHIT1 |  |
|  | LOC100131635 |  |
|  | CHRM3 |  |
|  | OTTHUMG00000159345 |  |
|  | MIR4476 |  |
|  | MIR4663 |  |
|  | CSPG4P5 |  |
|  | OTTHUMG00000164252 |  |
|  | PAPPA2 |  |
|  | PLAT |  |
|  | OTTHUMG00000163848 |  |
|  | GLDN |  |
|  | OTTHUMG00000154464 |  |
|  | PRAMEF11 |  |
|  | OTTHUMG00000160313 |  |
|  | BMPR1B |  |
|  | OTTHUMG00000163585 |  |
|  | ENTHD1 |  |
|  | OTTHUMG00000151725 |  |
|  | PATE2 |  |
|  | DEFB1 |  |
|  | OTTHUMG00000164243 |  |
|  | SPINT4 |  |
|  | OTTHUMG00000162592 |  |
|  | PLA2G4E |  |
|  | OTTHUMG00000151361 |  |
|  | PRKAG3 |  |
|  | SARDH |  |
|  | AGTR2 |  |
|  | RAI2 |  |
|  | PRSS45 |  |
|  | SELE |  |
|  | FOLR4 |  |
|  | LOC389023 |  |
|  | OTTHUMG00000163662 |  |
|  | CNGB1 |  |
|  | OTTHUMG00000032014 |  |
|  | PKD1L2 |  |
|  | OTTHUMG00000152640 |  |
|  | RNA5SP69 |  |
|  | APOC3 |  |
|  | OTTHUMG00000162358 |  |
|  | EMX2 |  |
|  | SH2D7 |  |
|  | KCNC4 |  |
|  | OTTHUMG00000086917 |  |
|  | MIR1255B1 |  |
|  | NBPF22P |  |
|  | ANGPT4 |  |
|  | OTTHUMG00000021098 |  |
|  | KIAA1549 |  |
|  | C8orf87 |  |
|  | LAMB2P1 |  |
|  | OTTHUMG00000020083 |  |
|  | OTTHUMG00000157651 |  |
|  | FAM166B |  |
|  | IGKV1D-43 |  |
|  | TMEM72 |  |
|  | OTTHUMG00000164075 |  |
|  | OTTHUMG00000035229 |  |
|  | OTTHUMG00000066033 |  |
|  | ASTN1 |  |
|  | OTOL1 |  |
|  | GSDMC |  |
|  | OTTHUMG00000032526 |  |
|  | KRT86 |  |
|  | SYT5 |  |
|  | OTTHUMG00000020566 |  |
|  | C1orf65 |  |
|  | SEPP1 |  |
|  | MIR345 |  |
|  | HCN1 |  |
|  | GPR97 |  |
|  | SNORD114-2 |  |
|  | MIR4325 |  |
|  | TRIM51 |  |
|  | DIRC3 |  |
|  | HSD3B2 |  |
|  | OTTHUMG00000156607 |  |
|  | LOC100128770 |  |
|  | TMEM215 |  |
|  | PRLH |  |
|  | OTTHUMG00000151939 |  |
|  | FGF10 |  |
|  | CRB3 |  |
|  | OTTHUMG00000017654 |  |
|  | IGHV3-38 |  |
|  | LIMD1-AS1 |  |
|  | GALNT5 |  |
|  | OTTHUMG00000041452 |  |
|  | COL4A2 |  |
|  | OTTHUMG00000162465 |  |
|  | WIF1 |  |
|  | NRSN2 |  |
|  | TRPV3 |  |
|  | OTTHUMG00000161645 |  |
|  | SLC38A3 |  |
|  | PMCHL2 |  |
|  | CIDEA |  |
|  | CPO |  |
|  | CLSTN2 |  |
|  | LOC100128682 |  |
|  | FAM188B2 |  |
|  | LOC100506050 |  |
|  | TNNT3 |  |
|  | KIR3DS1 |  |
|  | PNLIP |  |
|  | TACR3 |  |
|  | C20orf166-AS1 |  |
|  | C1orf110 |  |
|  | OTTHUMG00000159635 |  |
|  | LOC100506310 |  |
|  | RAB17 |  |
|  | CAV3 |  |
|  | MYL4 |  |
|  | OTTHUMG00000017566 |  |
|  | OTTHUMG00000032217 |  |
|  | LOC100506795 |  |
|  | GAL3ST4 |  |
|  | TRBV7-1 |  |
|  | OTTHUMG00000164859 |  |
|  | GRTP1-AS1 |  |
|  | OTTHUMG00000018169 |  |
|  | BTNL2 |  |
|  | OTTHUMG00000155093 |  |
|  | CA9 |  |
|  | OTTHUMG00000010702 |  |
|  | OTTHUMG00000162926 |  |
|  | LINC00620 |  |
|  | DPYSL5 |  |
|  | LEPREL1-AS1 |  |
|  | LOC100506948 |  |
|  | OTTHUMG00000163661 |  |
|  | TBX5 |  |
|  | OTTHUMG00000019446 |  |
|  | OTTHUMG00000161298 |  |
|  | LOC283038 |  |
|  | DPEP1 |  |
|  | PRDM13 |  |
|  | OTTHUMG00000162881 |  |
|  | REG4 |  |
|  | OTTHUMG00000132937 |  |
|  | OTTHUMG00000149316 |  |
|  | SCN10A |  |
|  | SALL4 |  |
|  | OTTHUMG00000021342 |  |
|  | PKP1 |  |
|  | OTTHUMG00000156122 |  |
|  | LOC100129973 |  |
|  | NLRP6 |  |
|  | CCDC129 |  |
|  | MGC27382 |  |
|  | OTTHUMG00000020564 |  |
|  | SDIM1 |  |
|  | CNPY1 |  |
|  | SYT8 |  |
|  | GRIK3 |  |
|  | OTTHUMG00000018809 |  |
|  | OTTHUMG00000161303 |  |
|  | OTTHUMG00000162438 |  |
|  | GLS2 |  |
|  | OTTHUMG00000161797 |  |
|  | FERD3L |  |
|  | LYZL6 |  |
|  | OTTHUMG00000155049 |  |
|  | OTTHUMG00000163913 |  |
|  | PPAPDC3 |  |
|  | LOC100506810 |  |
|  | DAZL |  |
|  | GNA14 |  |
|  | OTTHUMG00000165050 |  |
|  | OTTHUMG00000164204 |  |
|  | OTTHUMG00000008300 |  |
|  | CD300LG |  |
|  | DLGAP2 |  |
|  | GMNC |  |
|  | FAM78B |  |
|  | LINC00348 |  |
|  | VCX2 |  |
|  | ADAM29 |  |
|  | HCG21 |  |
|  | PRR26 |  |
|  | TMPRSS7 |  |
|  | LOC154872 |  |
|  | COL6A6 |  |
|  | OTTHUMG00000066362 |  |
|  | OTTHUMG00000132737 |  |
|  | LSAMP-AS2 |  |
|  | OTTHUMG00000159353 |  |
|  | OTTHUMG00000019251 |  |
|  | OTTHUMG00000152010 |  |
|  | LOC101060300 |  |
|  | LINC00967 |  |
|  | OTTHUMG00000163916 |  |
|  | OTTHUMG00000160219 |  |
|  | UGDH-AS1 |  |
|  | MCF2L-AS1 |  |
|  | ALG1L13P |  |
|  | LHFPL3 |  |
|  | LOC644135 |  |
|  | OTTHUMG00000163405 |  |
|  | ADORA1 |  |
|  | FSHB |  |
|  | TRIM45 |  |
|  | MC2R |  |
|  | EDN2 |  |
|  | FIGF |  |
|  | OTTHUMG00000150952 |  |
|  | GRID1-AS1 |  |
|  | HTR5BP |  |
|  | OTTHUMG00000154935 |  |
|  | SHC4 |  |
|  | ZNF705B |  |
|  | MIR598 |  |
|  | KANK4 |  |
|  | LOC100131320 |  |
|  | OTTHUMG00000160051 |  |
|  | OTTHUMG00000161668 |  |
|  | CDH3 |  |
|  | IGLV4-69 |  |
|  | C20orf141 |  |
|  | ABCG5 |  |
|  | OTTHUMG00000161291 |  |
|  | PDZD2 |  |
|  | OTTHUMG00000154842 |  |
|  | LENEP |  |
|  | LRRC29 |  |
|  | C2orf73 |  |
|  | SPANXE |  |
|  | OTTHUMG00000041348 |  |
|  | ADAMTS17 |  |
|  | OTTHUMG00000161490 |  |
|  | IGFN1 |  |
|  | PGLYRP2 |  |
|  | OTTHUMG00000021187 |  |
|  | OR4F29 |  |
|  | IMMP2L-IT1 |  |
|  | S100A7L2 |  |
|  | SNAR-F |  |
|  | LOC100506990 |  |
|  | OTTHUMG00000031932 |  |
|  | MIR588 |  |
|  | OTTHUMG00000015756 |  |
|  | FRMPD2P1 |  |
|  | OTTHUMG00000150220 |  |
|  | LOC100506489 |  |
|  | UG0898H09 |  |
|  | GDPD2 |  |
|  | OTTHUMG00000017530 |  |
|  | TEX13B |  |
|  | CCDC41-AS1 |  |
|  | OTTHUMG00000086699 |  |
|  | DLX3 |  |
|  | CCL11 |  |
|  | OTTHUMG00000155340 |  |
|  | IGHV6-1 |  |
|  | OTTHUMG00000032045 |  |
|  | ATP10B |  |
|  | CSN2 |  |
|  | SLC5A1 |  |
|  | OTTHUMG00000161899 |  |
|  | ZBTB32 |  |
|  | SHC3 |  |
|  | HABP2 |  |
|  | MSMP |  |
|  | OTTHUMG00000155195 |  |
|  | KIAA1755 |  |
|  | OTTHUMG00000161580 |  |
|  | OTTHUMG00000019024 |  |
|  | LOC338963 |  |
|  | FMO3 |  |
|  | DKFZP434L187 |  |
|  | OPALIN |  |
|  | OTTHUMG00000163114 |  |
|  | CYP2C8 |  |
|  | OTTHUMG00000164004 |  |
|  | OTTHUMG00000018061 |  |
|  | LOC100505495 |  |
|  | OTTHUMG00000008373 |  |
|  | FAM153C |  |
|  | ADRA1A |  |
|  | C2orf57 |  |
|  | NBPF4 |  |
|  | ADAMTSL3 |  |
|  | ASXL3 |  |
|  | SCARA3 |  |
|  | OTTHUMG00000162951 |  |
|  | OTTHUMG00000037123 |  |
|  | OTTHUMG00000040845 |  |
|  | KRT85 |  |
|  | KCNMB2 |  |
|  | OTTHUMG00000011992 |  |
|  | KISS1 |  |
|  | PCSK1 |  |
|  | OTTHUMG00000161634 |  |
|  | MIR513A1 |  |
|  | FAM86C2P |  |
|  | OTTHUMG00000160168 |  |
|  | GUCA2B |  |
|  | LINC00959 |  |
|  | PSCA |  |
|  | KIR3DL3 |  |
|  | NPAS3 |  |
|  | OTTHUMG00000160754 |  |
|  | SULT1C2 |  |
|  | TSRM |  |
|  | NAA11 |  |
|  | IGHV3-35 |  |
|  | APOD |  |
|  | LINC00839 |  |
|  | RNU7-19P |  |
|  | ZSCAN23 |  |
|  | CCDC70 |  |
|  | OR5AR1 |  |
|  | LCE1C |  |
|  | LINC00518 |  |
|  | OTTHUMG00000163846 |  |
|  | FAM153A |  |
|  | OTTHUMG00000037403 |  |
|  | IFNL1 |  |
|  | MYADML2 |  |
|  | NRG3-AS1 |  |
|  | OTTHUMG00000134297 |  |
|  | OTTHUMG00000152384 |  |
|  | OTTHUMG00000164978 |  |
|  | LINC00614 |  |
|  | IGFBP6 |  |
|  | SNORD115-23 |  |
|  | CT64 |  |
|  | AA06 |  |
|  | FABP3 |  |
|  | FANK1-AS1 |  |
|  | OR11A1 |  |
|  | SPINK13 |  |
|  | OPRD1 |  |
|  | OTTHUMG00000162456 |  |
|  | LCN10 |  |
|  | OTTHUMG00000162006 |  |
|  | IQCF1 |  |
|  | OTTHUMG00000155986 |  |
|  | LOC100507657 |  |
|  | MIR3186 |  |
|  | CACNA2D3-AS1 |  |
|  | ARHGEF15 |  |
|  | C3orf70 |  |
|  | FOLH1 |  |
|  | TUBBP5 |  |
|  | PROZ |  |
|  | MUC7 |  |
|  | MAMDC4 |  |
|  | LDB3 |  |
|  | FAM151A |  |
|  | OTTHUMG00000003326 |  |
|  | OTTHUMG00000152511 |  |
|  | LOC340508 |  |
|  | LOC645355 |  |
|  | AOC3 |  |
|  | HHIPL2 |  |
|  | OTTHUMG00000041157 |  |
|  | COL22A1 |  |
|  | BEAN1 |  |
|  | OTTHUMG00000018146 |  |
|  | OTTHUMG00000015845 |  |
|  | OTTHUMG00000163483 |  |
|  | OR2B2 |  |
|  | PLA2G2E |  |
|  | GABRB3 |  |
|  | F9 |  |
|  | OTTHUMG00000163404 |  |
|  | OTTHUMG00000154735 |  |
|  | TTTY6B |  |
|  | ALMS1P |  |
|  | OTTHUMG00000156393 |  |
|  | KRTAP20-4 |  |
|  | FAM153B |  |
|  | AMIGO1 |  |
|  | LINC00670 |  |
|  | LINC00159 |  |
|  | EBLN1 |  |
|  | HTR1A |  |
|  | NAP1L3 |  |
|  | PCDH18 |  |
|  | MYLK2 |  |
|  | OTTHUMG00000015055 |  |
|  | OTTHUMG00000163796 |  |
|  | CYP4F12 |  |
|  | LRFN2 |  |
|  | HBBP1 |  |
|  | RNA5SP241 |  |
|  | OTTHUMG00000160485 |  |
|  | EYA1 |  |
|  | TRBV6-8 |  |
|  | OTTHUMG00000024076 |  |
|  | LOC285889 |  |
|  | P2RX6P |  |
|  | OTTHUMG00000163760 |  |
|  | A1CF |  |
|  | OTTHUMG00000000721 |  |
|  | C2orf54 |  |
|  | ZNF385C |  |
|  | MAGI1 |  |
|  | FOXL1 |  |
|  | FAM19A3 |  |
|  | C1orf56 |  |
|  | OTTHUMG00000011637 |  |
|  | OR9G4 |  |
|  | GCNT3 |  |
|  | ASH1L-IT1 |  |
|  | OTTHUMG00000065063 |  |
|  | DHRS2 |  |
|  | MIR129-1 |  |
|  | WTAPP1 |  |
|  | OTTHUMG00000156018 |  |
|  | LOC388942 |  |
|  | RNF112 |  |
|  | OTTHUMG00000041453 |  |
|  | ACTRT1 |  |
|  | TMEM45A |  |
|  | LRIT3 |  |
|  | OTTHUMG00000031855 |  |
|  | IGHV1-8 |  |
|  | OTTHUMG00000163891 |  |
|  | LCN8 |  |
|  | FCRL2 |  |
|  | LRRC4C |  |
|  | NLRP4 |  |
|  | OTTHUMG00000160645 |  |
|  | DCLK1 |  |
|  | EHHADH-AS1 |  |
|  | PSG11 |  |
|  | FIGN |  |
|  | SLC23A1 |  |
|  | OTTHUMG00000159391 |  |
|  | DNAJB8 |  |
|  | OTTHUMG00000152865 |  |
|  | CDC20B |  |
|  | RASSF9 |  |
|  | OTTHUMG00000019130 |  |
|  | OTTHUMG00000163471 |  |
|  | PAGE5 |  |
|  | LINC00483 |  |
|  | OTTHUMG00000163750 |  |
|  | OTTHUMG00000020681 |  |
|  | LOC100509303 |  |
|  | UGT2B7 |  |
|  | OTTHUMG00000150731 |  |
|  | OTTHUMG00000043709 |  |
|  | LOC643542 |  |
|  | MUM1L1 |  |
|  | HPCAL4 |  |
|  | VWA5B1 |  |
|  | OTTHUMG00000015214 |  |
|  | RBMS3-AS1 |  |
|  | OTTHUMG00000151787 |  |
|  | PCDHB13 |  |
|  | OTTHUMG00000150931 |  |
|  | GGT5 |  |
|  | OTTHUMG00000034556 |  |
|  | LOC100996635 |  |
|  | GRIA3 |  |
|  | LOC100507651 |  |
|  | CRISP2 |  |
|  | OTTHUMG00000017760 |  |
|  | LOC100287225 |  |
|  | OTTHUMG00000153152 |  |
|  | FGF21 |  |
|  | CLLU1OS |  |
|  | LINC00664 |  |
|  | OTTHUMG00000015221 |  |
|  | TMEM31 |  |
|  | AADAC |  |
|  | CLCN1 |  |
|  | REM1 |  |
|  | FAM66B |  |
|  | WNK4 |  |
|  | LINC00589 |  |
|  | STEAP1 |  |
|  | OTTHUMG00000150919 |  |
|  | OTTHUMG00000153043 |  |
|  | CADPS |  |
|  | SUSD2 |  |
|  | MIR3196 |  |
|  | OTTHUMG00000015772 |  |
|  | ACSM1 |  |
|  | GLIS3 |  |
|  | RNA5SP361 |  |
|  | MIR4708 |  |
|  | OTTHUMG00000162795 |  |
|  | LOC100506368 |  |
|  | OTTHUMG00000160256 |  |
|  | OR5M11 |  |
|  | LOC93463 |  |
|  | TREML3P |  |
|  | IGLV7-43 |  |
|  | SMIM21 |  |
|  | FLJ38723 |  |
|  | KRT37 |  |
|  | SLC12A1 |  |
|  | EPB42 |  |
|  | OTTHUMG00000162405 |  |
|  | DNAJB3 |  |
|  | LOC644215 |  |
|  | OTTHUMG00000151710 |  |
|  | OTTHUMG00000004135 |  |
|  | OTTHUMG00000016859 |  |
|  | OTTHUMG00000161041 |  |
|  | TAS1R3 |  |
|  | LINC00303 |  |
|  | OR10J5 |  |
|  | LNX1-AS1 |  |
|  | MIR4776-2 |  |
|  | OTTHUMG00000160625 |  |
|  | NAP1L6 |  |
|  | C21orf54 |  |
|  | OTTHUMG00000078707 |  |
|  | SLC4A9 |  |
|  | UGT3A1 |  |
|  | LOC84931 |  |
|  | FLJ45872 |  |
|  | OTTHUMG00000154837 |  |
|  | C17orf99 |  |
|  | OTTHUMG00000158284 |  |
|  | EME1 |  |
|  | NRIP2 |  |
|  | HSD17B14 |  |
|  | OR5J2 |  |
|  | MIR4327 |  |
|  | OTTHUMG00000163950 |  |
|  | LOC100169752 |  |
|  | PRB2 |  |
|  | KLKP1 |  |
|  | ANHX |  |
|  | LINC00535 |  |
|  | LOC100144597 |  |
|  | OTTHUMG00000162621 |  |
|  | OR6M1 |  |
|  | OTTHUMG00000153545 |  |
|  | ZNF295-AS1 |  |
|  | TMCO5A |  |
|  | MIR499A |  |
|  | OTTHUMG00000037980 |  |
|  | OTTHUMG00000151622 |  |
|  | GH2 |  |
|  | PDE6A |  |
|  | OTTHUMG00000078275 |  |
|  | LOC100506421 |  |
|  | LOC440173 |  |
|  | OTTHUMG00000164807 |  |
|  | MIR4690 |  |
|  | STRC |  |
|  | FREM2 |  |
|  | OTTHUMG00000151707 |  |
|  | OTTHUMG00000015771 |  |
|  | PRKCDBP |  |
|  | FOXN1 |  |
|  | KRT76 |  |
|  | SBSN |  |
|  | OTTHUMG00000164883 |  |
|  | SEC14L4 |  |
|  | ANKRD20A19P |  |
|  | OTTHUMG00000018026 |  |
|  | LOC100287728 |  |
|  | OTTHUMG00000041087 |  |
|  | OTTHUMG00000162824 |  |
|  | OTTHUMG00000162106 |  |
|  | LOC100507538 |  |
|  | COL19A1 |  |
|  | UBQLN3 |  |
|  | OTTHUMG00000137478 |  |
|  | OTTHUMG00000149045 |  |
|  | OTTHUMG00000149554 |  |
|  | OTTHUMG00000137482 |  |
|  | DCC |  |
|  | LOC100287879 |  |
|  | ANKRD20A11P |  |
|  | IGHEP1 |  |
|  | OTTHUMG00000133695 |  |
|  | MIR548Y |  |
|  | MIR770 |  |
|  | RSPO4 |  |
|  | OTTHUMG00000036070 |  |
|  | TAAR6 |  |
|  | EDDM3B |  |
|  | VAT1L |  |
|  | OTTHUMG00000161299 |  |
|  | OTTHUMG00000161702 |  |
|  | OTTHUMG00000161278 |  |
|  | OTTHUMG00000020815 |  |
|  | OTTHUMG00000008422 |  |
|  | LOC100505776 |  |
|  | NOVA1 |  |
|  | LINC00387 |  |
|  | OTTHUMG00000018243 |  |
|  | OTTHUMG00000032154 |  |
|  | OTTHUMG00000160774 |  |
|  | OTTHUMG00000151592 |  |
|  | HTR5A |  |
|  | FN1 |  |
|  | OTTHUMG00000156551 |  |
|  | ABP1 |  |
|  | GOLGA6L6 |  |
|  | TAAR5 |  |
|  | OTTHUMG00000031478 |  |
|  | PLEKHB1 |  |
|  | OR5B21 |  |
|  | OTTHUMG00000161140 |  |
|  | MIR4654 |  |
|  | OTTHUMG00000066367 |  |
|  | SPTSSB |  |
|  | GJB4 |  |
|  | FMO6P |  |
|  | LOC100505545 |  |
|  | MAP1LC3C |  |
|  | MIR527 |  |
|  | OTTHUMG00000163822 |  |
|  | DEFB115 |  |
|  | NUDT11 |  |
|  | KRBOX1 |  |
|  | RTN4RL2 |  |
|  | FRG2C |  |
|  | RBP2 |  |
|  | OTTHUMG00000035527 |  |
|  | OTTHUMG00000037610 |  |
|  | OTTHUMG00000149451 |  |
|  | LOC728175 |  |
|  | LOC101060615 |  |
|  | C2orf16 |  |
|  | OTTHUMG00000015659 |  |
|  | OTTHUMG00000159842 |  |
|  | OTTHUMG00000165008 |  |
|  | AKR1B15 |  |
|  | CHODL |  |
|  | MBL2 |  |
|  | OR2A25 |  |
|  | OR5T1 |  |
|  | OTTHUMG00000022543 |  |
|  | LINC00840 |  |
|  | C15orf32 |  |
|  | LINC00504 |  |
|  | OTTHUMG00000018570 |  |
|  | CLDN2 |  |
|  | RNA5SP362 |  |
|  | TMEM163 |  |
|  | IGFL2 |  |
|  | MIR877 |  |
|  | SERPINA11 |  |
|  | IGHV1-45 |  |
|  | GPHA2 |  |
|  | RGL4 |  |
|  | RNA5SP296 |  |
|  | FFAR3 |  |
|  | MIR432 |  |
|  | CECR2 |  |
|  | BANF2 |  |
|  | RPS12P23 |  |
|  | OTTHUMG00000162491 |  |
|  | RERG |  |
|  | KIAA1024L |  |
|  | CHI3L2 |  |
|  | OTTHUMG00000152469 |  |
|  | ARMS2 |  |
|  | OTTHUMG00000164908 |  |
|  | CORO2B |  |
|  | NOSIP |  |
|  | WISP2 |  |
|  | OTTHUMG00000153164 |  |
|  | SERPINC1 |  |
|  | LOC441666 |  |
|  | TMPRSS4 |  |
|  | FAM83C-AS1 |  |
|  | NXF5 |  |
|  | ELN |  |
|  | IGHV3-16 |  |
|  | SERPINA12 |  |
|  | SHISA6 |  |
|  | FOLR1 |  |
|  | ATP13A4-AS1 |  |
|  | REG3G |  |
|  | OTTHUMG00000017094 |  |
|  | OTTHUMG00000162864 |  |
|  | TDGF1 |  |
|  | PEG10 |  |
|  | LOC100128164 |  |
|  | TMEM212 |  |
|  | TNR |  |
|  | CASR |  |
|  | LINC00907 |  |
|  | OTTHUMG00000159720 |  |
|  | LINC00263 |  |
|  | OTTHUMG00000008126 |  |
|  | CRHR1 |  |
|  | F2 |  |
|  | OTTHUMG00000015852 |  |
|  | F10 |  |
|  | POU5F1P4 |  |
|  | OTTHUMG00000064910 |  |
|  | GPR176 |  |
|  | HEPHL1 |  |
|  | LOC286442 |  |
|  | OTTHUMG00000152719 |  |
|  | MSLNL |  |
|  | C19orf33 |  |
|  | ZNF705D |  |
|  | LOC646736 |  |
|  | OTTHUMG00000059036 |  |
|  | DYDC2 |  |
|  | GML |  |
|  | CD164L2 |  |
|  | IGHV1OR21-1 |  |
|  | OTTHUMG00000020563 |  |
|  | OTTHUMG00000018679 |  |
|  | TRIM29 |  |
|  | GPC5-AS1 |  |
|  | RNA5SP420 |  |
|  | SULT1C2P1 |  |
|  | LIM2 |  |
|  | MAGEA10 |  |
|  | LOC100507073 |  |
|  | OTTHUMG00000017459 |  |
|  | ZNF774 |  |
|  | TEX35 |  |
|  | CCL8 |  |
|  | LOC388882 |  |
|  | CHIA |  |
|  | OTTHUMG00000159908 |  |
|  | RNA5SP32 |  |
|  | CHRNA6 |  |
|  | KIF5A |  |
|  | SRGAP2-AS1 |  |
|  | OTTHUMG00000031850 |  |
|  | OTTHUMG00000151479 |  |
|  | SVEP1 |  |
|  | COL5A2 |  |
|  | OTTHUMG00000009453 |  |
|  | OTTHUMG00000018055 |  |
|  | CLMP |  |
|  | ONECUT1 |  |
|  | NLRP11 |  |
|  | OTTHUMG00000162822 |  |
|  | WEE2 |  |
|  | KRTAP7-1 |  |
|  | OTTHUMG00000008245 |  |
|  | OTTHUMG00000161051 |  |
|  | OTTHUMG00000163513 |  |
|  | LOC100506098 |  |
|  | OTTHUMG00000150859 |  |
|  | BPIFB1 |  |
|  | OTTHUMG00000154045 |  |
|  | OTTHUMG00000155664 |  |
|  | PIEZO2 |  |
|  | UMODL1 |  |
|  | IL31 |  |
|  | MAOB |  |
|  | OR8I2 |  |
|  | PTH2 |  |
|  | OTTHUMG00000154628 |  |
|  | OTTHUMG00000157319 |  |
|  | OTTHUMG00000151429 |  |
|  | IGFBP5 |  |
|  | KRT33A |  |
|  | IGLV5-37 |  |
|  | RNA5SP360 |  |
|  | RNA5SP56 |  |
|  | ASB4 |  |
|  | PTPN14 |  |
|  | TRBV11-1 |  |
|  | OTTHUMG00000020097 |  |
|  | KRT82 |  |
|  | OTTHUMG00000163155 |  |
|  | MYRFL |  |
|  | LOC728586 |  |
|  | LOC692247 |  |
|  | OTTHUMG00000153283 |  |
|  | OTTHUMG00000152841 |  |
|  | OTTHUMG00000032593 |  |
|  | LINC00964 |  |
|  | MUC13 |  |
|  | UNQ6494 |  |
|  | ABCA12 |  |
|  | MIR3978 |  |
|  | PDE10A |  |
|  | OTTHUMG00000162352 |  |
|  | TP53TG5 |  |
|  | PCP4L1 |  |
|  | OTTHUMG00000164175 |  |
|  | OTTHUMG00000155818 |  |
|  | MTUS2-AS1 |  |
|  | OTTHUMG00000161732 |  |
|  | GABRR1 |  |
|  | OTTHUMG00000164722 |  |
|  | IGLV3-9 |  |
|  | MIR4746 |  |
|  | OTTHUMG00000003683 |  |
|  | KRTAP6-2 |  |
|  | FOXJ1 |  |
|  | OTTHUMG00000019554 |  |
|  | RNA5SP410 |  |
|  | LOC730811 |  |
|  | GJA5 |  |
|  | OTTHUMG00000160257 |  |
|  | SDR42E2 |  |
|  | DBIL5P2 |  |
|  | OTTHUMG00000156285 |  |
|  | FCN3 |  |
|  | CYP2C9 |  |
|  | OTTHUMG00000156463 |  |
|  | MTMR9LP |  |
|  | SCGB2A2 |  |
|  | OTTHUMG00000159760 |  |
|  | OTTHUMG00000153025 |  |
|  | SHOX2 |  |
|  | CSH2 |  |
|  | MIR192 |  |
|  | COL6A4P2 |  |
|  | LOC729970 |  |
|  | IRGM |  |
|  | LMOD1 |  |
|  | OPCML-IT2 |  |
|  | SYNE4 |  |
|  | CES5A |  |
|  | LPPR4 |  |
|  | OCSTAMP |  |
|  | P2RX6 |  |
|  | COL14A1 |  |
|  | ZNF135 |  |
|  | MMP2 |  |
|  | LOC100505716 |  |
|  | TMEM252 |  |
|  | S100A1 |  |
|  | DEFB107A |  |
|  | OTTHUMG00000019057 |  |
|  | OTTHUMG00000013091 |  |
|  | FHOD3 |  |
|  | OTTHUMG00000017184 |  |
|  | LOC100505795 |  |
|  | OTTHUMG00000017550 |  |
|  | TGM4 |  |
|  | THY1 |  |
|  | OTTHUMG00000005710 |  |
|  | OTTHUMG00000007622 |  |
|  | OTTHUMG00000162900 |  |
|  | SNORD114-7 |  |
|  | OTTHUMG00000165019 |  |
|  | AQP8 |  |
|  | HMGN2P46 |  |
|  | RNA5SP275 |  |
|  | OTTHUMG00000065294 |  |
|  | OTTHUMG00000012023 |  |
|  | COX4I2 |  |
|  | LOC100996884 |  |
|  | OTTHUMG00000156511 |  |
|  | APBB1 |  |
|  | PON1 |  |
|  | CYLC2 |  |
|  | OTTHUMG00000021298 |  |
|  | KLK15 |  |
|  | NANOS3 |  |
|  | POU5F1B |  |
|  | KRT223P |  |
|  | LOC100506288 |  |
|  | RNA5SP34 |  |
|  | OTTHUMG00000162779 |  |
|  | OTTHUMG00000155115 |  |
|  | OTTHUMG00000153425 |  |
|  | KIRREL-IT1 |  |
|  | DEFB124 |  |
|  | RAB25 |  |
|  | KLHL6-AS1 |  |
|  | OTTHUMG00000160692 |  |
|  | LOC285847 |  |
|  | TLR8-AS1 |  |
|  | OTTHUMG00000155997 |  |
|  | OTTHUMG00000141312 |  |
|  | LINC00648 |  |
|  | OTTHUMG00000160471 |  |
|  | LOC100506329 |  |
|  | OTTHUMG00000151447 |  |
|  | LOC643441 |  |
|  | OTTHUMG00000163146 |  |
|  | OTTHUMG00000184091 |  |
|  | RNY3P4 |  |
|  | OTTHUMG00000034988 |  |
|  | LRRC3B |  |
|  | C22orf42 |  |
|  | EFNB2 |  |
|  | UBQLNL |  |
|  | OTTHUMG00000150954 |  |
|  | CAMP |  |
|  | ATP2B2-IT1 |  |
|  | OTTHUMG00000152843 |  |
|  | PLEKHS1 |  |
|  | OTTHUMG00000020423 |  |
|  | GADL1 |  |
|  | OTTHUMG00000037131 |  |
|  | LINC00445 |  |
|  | PCP4 |  |
|  | NT5DC4 |  |
|  | S100B |  |
|  | APLNR |  |
|  | OTTHUMG00000152956 |  |
|  | OTTHUMG00000153330 |  |
|  | OTTHUMG00000163148 |  |
|  | ALPI |  |
|  | OTTHUMG00000018325 |  |
|  | GPR137C |  |
|  | PNLIPRP2 |  |
|  | GRM1 |  |
|  | SPHKAP |  |
|  | VSNL1 |  |
|  | SLC38A8 |  |
|  | OTTHUMG00000165042 |  |
|  | LAMC2 |  |
|  | OTTHUMG00000032767 |  |
|  | KIAA2022 |  |
|  | NTN5 |  |
|  | TMEM119 |  |
|  | LOC100270679 |  |
|  | OTTHUMG00000159952 |  |
|  | SPATA31A2 |  |
|  | CTSE |  |
|  | CR2 |  |
|  | SLC47A2 |  |
|  | OTTHUMG00000015897 |  |
|  | OTTHUMG00000160863 |  |
|  | OTTHUMG00000160867 |  |
|  | XAGE1A |  |
|  | OTTHUMG00000163768 |  |
|  | OTTHUMG00000037985 |  |
|  | OTTHUMG00000153513 |  |
|  | MOG |  |
|  | OTTHUMG00000161545 |  |
|  | AADACL2 |  |
|  | MAGEA9 |  |
|  | RNA5SP441 |  |
|  | OR7A10 |  |
|  | MIR4526 |  |
|  | LINC00314 |  |
|  | MUSK |  |
|  | MTUS2 |  |
|  | OTTHUMG00000017561 |  |
|  | OTTHUMG00000086837 |  |
|  | MIR587 |  |
|  | LOC255654 |  |
|  | LINC00692 |  |
|  | OTTHUMG00000163972 |  |
|  | OTTHUMG00000014922 |  |
|  | LINC00494 |  |
|  | MIR31HG |  |
|  | SPATA12 |  |
|  | LINC00524 |  |
|  | OTTHUMG00000162918 |  |
|  | MIR489 |  |
|  | LOC100129427 |  |
|  | OTTHUMG00000161247 |  |
|  | GLP2R |  |
|  | EMCN-IT3 |  |
|  | OR4D5 |  |
|  | MIR4280 |  |
|  | OR52N2 |  |
|  | LOC100506013 |  |
|  | RFPL1 |  |
|  | LOC100507584 |  |
|  | LINC00354 |  |
|  | OTTHUMG00000013154 |  |
|  | PLA2G4D |  |
|  | DMRTC2 |  |
|  | OTTHUMG00000151898 |  |
|  | OTTHUMG00000152531 |  |
|  | OTTHUMG00000161956 |  |
|  | OTTHUMG00000157233 |  |
|  | EFNA1 |  |
|  | LINC00595 |  |
|  | ATP11A-AS1 |  |
|  | MIR622 |  |
|  | OTTHUMG00000161149 |  |
|  | OTTHUMG00000149420 |  |
|  | OTTHUMG00000018904 |  |
|  | PRSS2 |  |
|  | OTTHUMG00000161707 |  |
|  | OTTHUMG00000017053 |  |
|  | TSPEAR-AS1 |  |
|  | C16orf95 |  |
|  | OTTHUMG00000000421 |  |
|  | OTTHUMG00000178492 |  |
|  | KCNE1L |  |
|  | RN7SKP8 |  |
|  | OTTHUMG00000153783 |  |
|  | TRIM51HP |  |
|  | OTTHUMG00000015782 |  |
|  | OTTHUMG00000032525 |  |
|  | OTTHUMG00000002703 |  |
|  | RNF152 |  |
|  | OTTHUMG00000162153 |  |
|  | AKR7A2P1 |  |
|  | QRFP |  |
|  | CDRT15L2 |  |
|  | OTTHUMG00000150925 |  |
|  | ANKRD26P3 |  |
|  | SLC22A7 |  |
|  | OTTHUMG00000007952 |  |
|  | SAPCD1-AS1 |  |
|  | OTTHUMG00000148797 |  |
|  | OTTHUMG00000137424 |  |
|  | OTTHUMG00000149032 |  |
|  | OTTHUMG00000149545 |  |
|  | HOXC-AS2 |  |
|  | PRODH2 |  |
|  | OTTHUMG00000152559 |  |
|  | SLC9B1P1 |  |
|  | OTTHUMG00000015607 |  |
|  | DSCR4-IT1 |  |
|  | KCNB2 |  |
|  | LOC100507384 |  |
|  | SPERT |  |
|  | OTTHUMG00000151724 |  |
|  | LINC00844 |  |
|  | HHATL |  |
|  | AKAP14 |  |
|  | OTTHUMG00000000405 |  |
|  | GAST |  |
|  | LOC101060004 |  |
|  | OTTHUMG00000133666 |  |
|  | FAM178B |  |
|  | OTTHUMG00000020662 |  |
|  | LOC728095 |  |
|  | OTTHUMG00000032051 |  |
|  | OTTHUMG00000159740 |  |
|  | COL4A6 |  |
|  | CCL21 |  |
|  | NEXN-AS1 |  |
|  | OTTHUMG00000156060 |  |
|  | FETUB |  |
|  | SOWAHB |  |
|  | VPREB1 |  |
|  | MRGPRD |  |
|  | MIR548I4 |  |
|  | CRYBA1 |  |
|  | OR14A16 |  |
|  | POU1F1 |  |
|  | OTTHUMG00000078660 |  |
|  | IQCF3 |  |
|  | OTTHUMG00000046129 |  |
|  | OTTHUMG00000031781 |  |
|  | UGT2B15 |  |
|  | OTTHUMG00000164869 |  |
|  | OTTHUMG00000164124 |  |
|  | OTTHUMG00000161242 |  |
|  | TCEAL5 |  |
|  | MIR648 |  |
|  | OTTHUMG00000153500 |  |
|  | TSPYL6 |  |
|  | CST8 |  |
|  | UGT2A2 |  |
|  | HNF1A |  |
|  | FAM87A |  |
|  | SFTPA2 |  |
|  | ATCAY |  |
|  | TENM1 |  |
|  | OTTHUMG00000161928 |  |
|  | OTTHUMG00000164157 |  |
|  | MIR337 |  |
|  | LYPD4 |  |
|  | OTTHUMG00000085305 |  |
|  | LOC146481 |  |
|  | OTTHUMG00000162332 |  |
|  | OTOS |  |
|  | MIR758 |  |
|  | STAB2 |  |
|  | SMARCA1 |  |
|  | OTTHUMG00000163714 |  |
|  | MYH13 |  |
|  | MAGEB16 |  |
|  | OTTHUMG00000163985 |  |
|  | OTTHUMG00000165030 |  |
|  | LOC440337 |  |
|  | OTTHUMG00000156038 |  |
|  | MYL3 |  |
|  | DNALI1 |  |
|  | CELA2A |  |
|  | OTTHUMG00000162801 |  |
|  | LINC00239 |  |
|  | KRTAP4-1 |  |
|  | CYP2A6 |  |
|  | OTTHUMG00000037983 |  |
|  | F7 |  |
|  | CYP2B7P1 |  |
|  | OTTHUMG00000163595 |  |
|  | OTTHUMG00000154065 |  |
|  | OTTHUMG00000160738 |  |
|  | LINC00911 |  |
|  | OTTHUMG00000012388 |  |
|  | TAS2R38 |  |
|  | CTXN2 |  |
|  | RDH12 |  |
|  | NLRP14 |  |
|  | CXCL17 |  |
|  | OTTHUMG00000014222 |  |
|  | OTTHUMG00000154189 |  |
|  | LOC100507554 |  |
|  | OTTHUMG00000161598 |  |
|  | OTTHUMG00000014117 |  |
|  | OTTHUMG00000151194 |  |
|  | CSHL1 |  |
|  | EPHA1 |  |
|  | SLC22A8 |  |
|  | ADARB2-AS1 |  |
|  | OTTHUMG00000086303 |  |
|  | LOC100127888 |  |
|  | ANK2 |  |
|  | TRBV11-2 |  |
|  | KRT32 |  |
|  | TCAM1P |  |
|  | OTTHUMG00000015276 |  |
|  | LINC00572 |  |
|  | GLDC |  |
|  | OTTHUMG00000150750 |  |
|  | OTTHUMG00000011006 |  |
|  | SLAMF9 |  |
|  | OTTHUMG00000161494 |  |
|  | ZMYM4-AS1 |  |
|  | OR10X1 |  |
|  | OTTHUMG00000165031 |  |
|  | OTTHUMG00000151483 |  |
|  | LINC00478 |  |
|  | OTTHUMG00000151425 |  |
|  | OTTHUMG00000163812 |  |
|  | LOC286370 |  |
|  | RNA5SP200 |  |
|  | SNORD114-1 |  |
|  | MSX2P1 |  |
|  | OTTHUMG00000155731 |  |
|  | CLDN16 |  |
|  | OTTHUMG00000160726 |  |
|  | OR6C2 |  |
|  | OTTHUMG00000009217 |  |
|  | LRTM1 |  |
|  | LOC100129518 |  |
|  | USP26 |  |
|  | OTTHUMG00000140119 |  |
|  | OTTHUMG00000140117 |  |
|  | C22orf15 |  |
|  | TRIM48 |  |
|  | OTTHUMG00000164793 |  |
|  | DEFB119 |  |
|  | LOC339240 |  |
|  | MIR4511 |  |
|  | LOC100506059 |  |
|  | OTTHUMG00000162696 |  |
|  | COL3A1 |  |
|  | OR5M9 |  |
|  | LINC00603 |  |
|  | LINC00671 |  |
|  | SST |  |
|  | SERPINA4 |  |
|  | GAB4 |  |
|  | KRT77 |  |
|  | MB |  |
|  | GLYCAM1 |  |
|  | UPK1A |  |
|  | TRPC5OS |  |
|  | HPSE2 |  |
|  | SIAH3 |  |
|  | MAP2 |  |
|  | CELF3 |  |
|  | OTTHUMG00000149475 |  |
|  | SLC22A13 |  |
|  | PAX5 |  |
|  | OTTHUMG00000158980 |  |
|  | RNU7-26P |  |
|  | RNA5SP391 |  |
|  | OTTHUMG00000153722 |  |
|  | OTTHUMG00000036478 |  |
|  | OTTHUMG00000164982 |  |
|  | OTTHUMG00000015496 |  |
|  | LINC00552 |  |
|  | LSAMP |  |
|  | OTTHUMG00000153589 |  |
|  | LINC00618 |  |
|  | FXYD3 |  |
|  | OTTHUMG00000161693 |  |
|  | OTTHUMG00000074207 |  |
|  | TMEM210 |  |
|  | INPP5J |  |
|  | OTTHUMG00000154227 |  |
|  | LOC100507439 |  |
|  | IL22RA1 |  |
|  | OTTHUMG00000154204 |  |
|  | VPREB3 |  |
|  | KRT78 |  |
|  | OTTHUMG00000163630 |  |
|  | EML1 |  |
|  | LOC388948 |  |
|  | SLC34A1 |  |
|  | LOC100507150 |  |
|  | OTTHUMG00000156131 |  |
|  | OTTHUMG00000156317 |  |
|  | DDR1-AS1 |  |
|  | OTTHUMG00000148935 |  |
|  | OTTHUMG00000149215 |  |
|  | PKNOX2 |  |
|  | ARSE |  |
|  | OTTHUMG00000164290 |  |
|  | OTTHUMG00000014657 |  |
|  | MTRNR2L7 |  |
|  | OSBPL6 |  |
|  | JRKL-AS1 |  |
|  | LINC00922 |  |
|  | DQX1 |  |
|  | MACROD2-AS1 |  |
|  | ODF3 |  |
|  | SLC36A3 |  |
|  | LINC00703 |  |
|  | NCMAP |  |
|  | LINC00570 |  |
|  | SNORD88A |  |
|  | OTTHUMG00000018152 |  |
|  | SORBS2 |  |
|  | OTTHUMG00000155418 |  |
|  | OTTHUMG00000153416 |  |
|  | CHRNB4 |  |
|  | OTTHUMG00000134311 |  |
|  | FOXF2 |  |
|  | POTEKP |  |
|  | FOXR2 |  |
|  | SRGAP3-AS2 |  |
|  | PLEKHH2 |  |
|  | OTTHUMG00000154719 |  |
|  | OTTHUMG00000016834 |  |
|  | ADAMTSL1 |  |
|  | CYP17A1 |  |
|  | OTTHUMG00000150909 |  |
|  | DEFA5 |  |
|  | TIMD4 |  |
|  | MIR4330 |  |
|  | OTTHUMG00000161068 |  |
|  | CLVS1 |  |
|  | DARC |  |
|  | SV2B |  |
|  | TEKT3 |  |
|  | OXER1 |  |
|  | MIR107 |  |
|  | MIR610 |  |
|  | OTTHUMG00000009024 |  |
|  | KLK6 |  |
|  | OTTHUMG00000160894 |  |
|  | FLJ42842 |  |
|  | LIPM |  |
|  | ERN2 |  |
|  | OTTHUMG00000161181 |  |
|  | HCG22 |  |
|  | MIR4540 |  |
|  | GRID2 |  |
|  | LOC100507065 |  |
|  | OTTHUMG00000164161 |  |
|  | LOC100129316 |  |
|  | NAALADL2-AS1 |  |
|  | LINC00707 |  |
|  | EML5 |  |
|  | AGXT |  |
|  | MIR4725 |  |
|  | MAGEA8 |  |
|  | RFPL3 |  |
|  | RAG1 |  |
|  | GYG2 |  |
|  | OTTHUMG00000060108 |  |
|  | OR2AT4 |  |
|  | OR51E2 |  |
|  | OTTHUMG00000160720 |  |
|  | LOC100128993 |  |
|  | OTTHUMG00000065234 |  |
|  | MIR3976 |  |
|  | OTTHUMG00000160140 |  |
|  | RAET1G |  |
|  | TLX1NB |  |
|  | OTTHUMG00000132247 |  |
|  | LINC00111 |  |
|  | OTTHUMG00000040631 |  |
|  | DOCK9-AS1 |  |
|  | OTTHUMG00000150106 |  |
|  | LOC100506105 |  |
|  | SRRM2-AS1 |  |
|  | TRHR |  |
|  | MIR891A |  |
|  | C7orf33 |  |
|  | OTTHUMG00000162990 |  |
|  | ITIH6 |  |
|  | OTTHUMG00000035627 |  |
|  | UBD |  |
|  | OTTHUMG00000164410 |  |
|  | CKMT1B |  |
|  | MCCD1 |  |
|  | BTBD17 |  |
|  | LOC100507288 |  |
|  | FGF7 |  |
|  | PLA2G4F |  |
|  | OTTHUMG00000060638 |  |
|  | DEFB106B |  |
|  | FAM74A3 |  |
|  | LOC283194 |  |
|  | HTR3D |  |
|  | LOC401437 |  |
|  | RNA5SP136 |  |
|  | SLC15A5 |  |
|  | OTTHUMG00000153745 |  |
|  | FLJ36777 |  |
|  | SLC13A5 |  |
|  | PRSS44 |  |
|  | TPTE2P6 |  |
|  | LINC00317 |  |
|  | OR4B1 |  |
|  | DUPD1 |  |
|  | OTTHUMG00000018021 |  |
|  | OTTHUMG00000159421 |  |
|  | OTTHUMG00000031966 |  |
|  | RAG2 |  |
|  | OTTHUMG00000017896 |  |
|  | OTTHUMG00000001769 |  |
|  | MC4R |  |
|  | TTLL2 |  |
|  | OTTHUMG00000156168 |  |
|  | OTTHUMG00000060826 |  |
|  | OTTHUMG00000160372 |  |
|  | MOGAT2 |  |
|  | OTTHUMG00000017568 |  |
|  | MGC10955 |  |
|  | ANKRD20A9P |  |
|  | PAGE2 |  |
|  | OTTHUMG00000164861 |  |
|  | OTTHUMG00000086723 |  |
|  | TBC1D29 |  |
|  | RBP5 |  |
|  | TMEM211 |  |
|  | OTTHUMG00000016756 |  |
|  | OR1N2 |  |
|  | OR6S1 |  |
|  | OTTHUMG00000009978 |  |
|  | OTTHUMG00000015720 |  |
|  | ERVMER61-1 |  |
|  | OTTHUMG00000000413 |  |
|  | LOC100996357 |  |
|  | OTTHUMG00000035685 |  |
|  | CYP2AB1P |  |
|  | NAT2 |  |
|  | OTTHUMG00000020201 |  |
|  | OTTHUMG00000014090 |  |
|  | C14orf144 |  |
|  | OTTHUMG00000015926 |  |
|  | LOC285441 |  |
|  | OTTHUMG00000154003 |  |
|  | OR3A3 |  |
|  | LINC00918 |  |
|  | OTTHUMG00000151749 |  |
|  | LINC00479 |  |
|  | GPR111 |  |
|  | IFNA7 |  |
|  | NTRK3 |  |
|  | COL11A1 |  |
|  | CYP2C19 |  |
|  | OTTHUMG00000151721 |  |
|  | MIR4290 |  |
|  | MIR3189 |  |
|  | C1orf180 |  |
|  | CYP17A1-AS1 |  |
|  | OR6Q1 |  |
|  | SUCLA2-AS1 |  |
|  | OTTHUMG00000078253 |  |
|  | SKINTL |  |
|  | CXCR5 |  |
|  | CREB3L1 |  |
|  | OTTHUMG00000066049 |  |
|  | OTTHUMG00000163521 |  |
|  | OTTHUMG00000151698 |  |
|  | OTTHUMG00000152851 |  |
|  | PSKH2 |  |
|  | OTTHUMG00000162355 |  |
|  | PDZD3 |  |
|  | OTTHUMG00000163325 |  |
|  | MIR617 |  |
|  | LIN28A |  |
|  | LINC00668 |  |
|  | IGLV4-60 |  |
|  | TEKT1 |  |
|  | OTTHUMG00000017992 |  |
|  | OTTHUMG00000015770 |  |
|  | OTTHUMG00000086865 |  |
|  | LRTM2 |  |
|  | OTTHUMG00000160427 |  |
|  | MIR204 |  |
|  | OTTHUMG00000150507 |  |
|  | KRTDAP |  |
|  | LOC100507144 |  |
|  | OTTHUMG00000035181 |  |
|  | KCNQ5-AS2 |  |
|  | PRAMEF1 |  |
|  | ABCG8 |  |
|  | OTTHUMG00000078324 |  |
|  | C8orf86 |  |
|  | MIR4710 |  |
|  | LOC154449 |  |
|  | SLC5A11 |  |
|  | TMC3 |  |
|  | MUC2 |  |
|  | OTTHUMG00000014610 |  |
|  | MMP20 |  |
|  | OTTHUMG00000163168 |  |
|  | MYPN |  |
|  | OTTHUMG00000152959 |  |
|  | OTTHUMG00000164364 |  |
|  | OTTHUMG00000159518 |  |
|  | OTTHUMG00000066363 |  |
|  | OTTHUMG00000161971 |  |
|  | GPR85 |  |
|  | POM121L10P |  |
|  | OTTHUMG00000031374 |  |
|  | OTTHUMG00000160650 |  |
|  | SYNGR4 |  |
|  | OTTHUMG00000159455 |  |
|  | SYNPR-AS1 |  |
|  | SAA4 |  |
|  | MIR9-1 |  |
|  | OTTHUMG00000164358 |  |
|  | OTTHUMG00000163152 |  |
|  | OTTHUMG00000039533 |  |
|  | OTTHUMG00000163069 |  |
|  | GPRC6A |  |
|  | SPINK7 |  |
|  | LOC100506373 |  |
|  | GIPC2 |  |
|  | RARRES2 |  |
|  | LOC400743 |  |
|  | GOLT1A |  |
|  | TFF1 |  |
|  | PRND |  |
|  | PGC |  |
|  | OTTHUMG00000015252 |  |
|  | OTTHUMG00000021018 |  |
|  | LOC100190940 |  |
|  | PPY2 |  |
|  | OTTHUMG00000156067 |  |
|  | OTTHUMG00000165084 |  |
|  | GLIS2 |  |
|  | FAM86B1 |  |
|  | THSD7B |  |
|  | OTTHUMG00000156171 |  |
|  | OTTHUMG00000066355 |  |
|  | CPA2 |  |
|  | LOC400654 |  |
|  | KRTAP19-7 |  |
|  | OBP2A |  |
|  | LOC100505841 |  |
|  | IGSF11-AS1 |  |
|  | KRT33B |  |
|  | OTTHUMG00000018930 |  |
|  | DEFB130 |  |
|  | OTTHUMG00000161764 |  |
|  | OTTHUMG00000004857 |  |
|  | SLC2A2 |  |
|  | LINC00663 |  |
|  | NXPE1 |  |
|  | LYG2 |  |
|  | RNA5SP449 |  |
|  | OTTHUMG00000150139 |  |
|  | OTTHUMG00000015943 |  |
|  | ROBO4 |  |
|  | HSD52 |  |
|  | LINC00601 |  |
|  | OTTHUMG00000133725 |  |
|  | MIR550B2 |  |
|  | TRPC2 |  |
|  | RNA5SP328 |  |
|  | ECSCR |  |
|  | CDR2L |  |
|  | LINC00207 |  |
|  | LINC00687 |  |
|  | OTTHUMG00000152562 |  |
|  | MAGEC3 |  |
|  | LOC100131347 |  |
|  | PRDM9 |  |
|  | ROBO2 |  |
|  | RGAG1 |  |
|  | ZFHX4 |  |
|  | IGHV1-46 |  |
|  | OTTHUMG00000019287 |  |
|  | OTTHUMG00000133704 |  |
|  | OTTHUMG00000010769 |  |
|  | LOC339760 |  |
|  | OR1B1 |  |
|  | WFDC5 |  |
|  | RNA5SP304 |  |
|  | OTTHUMG00000058269 |  |
|  | LINC00028 |  |
|  | HTR1B |  |
|  | OTTHUMG00000164909 |  |
|  | PLA2G2A |  |
|  | FGF12-AS1 |  |
|  | KIAA0087 |  |
|  | OTTHUMG00000020824 |  |
|  | SAMD7 |  |
|  | OTTHUMG00000014879 |  |
|  | LCE3A |  |
|  | IGHV3-53 |  |
|  | MIR3176 |  |
|  | PIGR |  |
|  | C5orf60 |  |
|  | OTTHUMG00000152373 |  |
|  | SNAR-D |  |
|  | TDGF1P3 |  |
|  | OTTHUMG00000159096 |  |
|  | OTTHUMG00000035577 |  |
|  | LOC100505822 |  |
|  | FOXE3 |  |
|  | C20orf173 |  |
|  | VAC14-AS1 |  |
|  | OTTHUMG00000161635 |  |
|  | OTTHUMG00000013290 |  |
|  | MIR4638 |  |
|  | BPIFA2 |  |
|  | TRIM64B |  |
|  | OR2B11 |  |
|  | OTTHUMG00000041229 |  |
|  | LOC100506183 |  |
|  | C7orf69 |  |
|  | OTTHUMG00000153849 |  |
|  | SACS-AS1 |  |
|  | OTTHUMG00000156509 |  |
|  | OTTHUMG00000154381 |  |
|  | TNC |  |
|  | OOEP |  |
|  | OTTHUMG00000157216 |  |
|  | LINC00883 |  |
|  | MIR1910 |  |
|  | KRT16P3 |  |
|  | KIAA1644 |  |
|  | OC90 |  |
|  | OTTHUMG00000162516 |  |
|  | OTTHUMG00000007307 |  |
|  | CDC20P1 |  |
|  | OTTHUMG00000153059 |  |
|  | OTTHUMG00000018646 |  |
|  | IL34 |  |
|  | NACAD |  |
|  | OTTHUMG00000156278 |  |
|  | KRT6B |  |
|  | DAOA-AS1 |  |
|  | TMEM213 |  |
|  | OTTHUMG00000163672 |  |
|  | LINC00411 |  |
|  | OTTHUMG00000020591 |  |
|  | COL1A2 |  |
|  | OTTHUMG00000014287 |  |
|  | OTTHUMG00000162103 |  |
|  | SPPL2C |  |
|  | OTTHUMG00000021092 |  |
|  | DENND5B-AS1 |  |
|  | AGAP1-IT1 |  |
|  | GIF |  |
|  | SFTPA1 |  |
|  | OTTHUMG00000164086 |  |
|  | HTR3E |  |
|  | OTTHUMG00000021379 |  |
|  | LOC100128714 |  |
|  | SFTA3 |  |
|  | ERCC6L |  |
|  | LOC286178 |  |
|  | LOC284751 |  |
|  | OTTHUMG00000151360 |  |
|  | OTTHUMG00000162869 |  |
|  | LINC00489 |  |
|  | SMR3B |  |
|  | SELENBP1 |  |
|  | TMEM212-AS1 |  |
|  | C2orf53 |  |
|  | LOC644838 |  |
|  | OPN5 |  |
|  | OTTHUMG00000035808 |  |
|  | DEFB126 |  |
|  | FLNB-AS1 |  |
|  | OTTHUMG00000159002 |  |
|  | MTRNR2L4 |  |
|  | OTTHUMG00000017201 |  |
|  | NXNL2 |  |
|  | SLC7A3 |  |
|  | BDKRB1 |  |
|  | TGM3 |  |
|  | LOC157273 |  |
|  | OTTHUMG00000017574 |  |
|  | OTTHUMG00000160740 |  |
|  | LOC643623 |  |
|  | OTTHUMG00000066035 |  |
|  | OTTHUMG00000041633 |  |
|  | LINC00920 |  |
|  | LOC100270746 |  |
|  | LOC100506102 |  |
|  | OTTHUMG00000015403 |  |
|  | NR3C2 |  |
|  | TMEM52B |  |
|  | OR52E8 |  |
|  | OTTHUMG00000017843 |  |
|  | MIR193A |  |
|  | IL36B |  |
|  | LOC100507199 |  |
|  | OTTHUMG00000151675 |  |
|  | CAPN13 |  |
|  | ABI3BP |  |
|  | OTTHUMG00000133715 |  |
|  | OTTHUMG00000148962 |  |
|  | OTTHUMG00000162767 |  |
|  | ANXA8L1 |  |
|  | C1orf191 |  |
|  | AANAT |  |
|  | MAGEB10 |  |
|  | NRG1-IT2 |  |
|  | KCNJ4 |  |
|  | LINC00905 |  |
|  | OTTHUMG00000160291 |  |
|  | OTTHUMG00000020443 |  |
|  | OTTHUMG00000153961 |  |
|  | SLC22A12 |  |
|  | LOC441242 |  |
|  | GSTA2 |  |
|  | KLHL1 |  |
|  | DEFA4 |  |
|  | PDPN |  |
|  | CEACAM16 |  |
|  | MGC4294 |  |
|  | LOC100506869 |  |
|  | OTTHUMG00000164081 |  |
|  | HYDIN |  |
|  | OTTHUMG00000153049 |  |
|  | TMEM95 |  |
|  | OTTHUMG00000151375 |  |
|  | OTTHUMG00000015860 |  |
|  | ANXA8L2 |  |
|  | PHGR1 |  |
|  | MIR921 |  |
|  | GALR3 |  |
|  | OTTHUMG00000018384 |  |
|  | RNASE7 |  |
|  | OTTHUMG00000163558 |  |
|  | LOC100505978 |  |
|  | DHRS3 |  |
|  | OTTHUMG00000162626 |  |
|  | OTTHUMG00000159199 |  |
|  | C10orf40 |  |
|  | OTTHUMG00000018885 |  |
|  | OTTHUMG00000008098 |  |
|  | GRM3 |  |
|  | TRBV7-4 |  |
|  | KCNMB2-IT1 |  |
|  | SLC9B1P3 |  |
|  | OTTHUMG00000058671 |  |
|  | OTTHUMG00000037896 |  |
|  | MIR548D2 |  |
|  | OTTHUMG00000037611 |  |
|  | OTTHUMG00000164644 |  |
|  | OTTHUMG00000037423 |  |
|  | LOC653712 |  |
|  | TAB3-AS1 |  |
|  | LOC100505940 |  |
|  | LINC00898 |  |
|  | FLJ45139 |  |
|  | SPATA31A1 |  |
|  | OTTHUMG00000163751 |  |
|  | RNA5SP332 |  |
|  | MIR892A |  |
|  | LOC148709 |  |
|  | FAM25E |  |
|  | FAM25D |  |
|  | SNORD115-13 |  |
|  | SLC6A15 |  |
|  | OTTHUMG00000013472 |  |
|  | SGCA |  |
|  | OTTHUMG00000014831 |  |
|  | OTTHUMG00000022403 |  |
|  | LINC00305 |  |
|  | OTTHUMG00000151833 |  |
|  | LOC100506534 |  |
|  | OTTHUMG00000012031 |  |
|  | C10orf129 |  |
|  | MIR4429 |  |
|  | GRM5-AS1 |  |
|  | OTTHUMG00000021383 |  |
|  | OTTHUMG00000161091 |  |
|  | OTTHUMG00000160367 |  |
|  | CAMTA1-IT1 |  |
|  | MIR455 |  |
|  | OTTHUMG00000015715 |  |
|  | CD7 |  |
|  | RNA5SP76 |  |
|  | OTTHUMG00000161823 |  |
|  | OTTHUMG00000153822 |  |
|  | OTTHUMG00000017681 |  |
|  | OTTHUMG00000159344 |  |
|  | LOC100507363 |  |
|  | AZGP1P1 |  |
|  | TEX29 |  |
|  | RNA5SP521 |  |
|  | RNA5SP522 |  |
|  | LOC100130700 |  |
|  | BMP3 |  |
|  | LCE1F |  |
|  | OTTHUMG00000021671 |  |
|  | OTTHUMG00000162131 |  |
|  | LEMD1-AS1 |  |
|  | KCNJ16 |  |
|  | PRY2 |  |
|  | OTTHUMG00000015831 |  |
|  | OTTHUMG00000153708 |  |
|  | OTTHUMG00000032887 |  |
|  | WWC2-AS1 |  |
|  | LINC00958 |  |
|  | MYL2 |  |
|  | OTTHUMG00000160753 |  |
|  | IGKV1-8 |  |
|  | DNMBP-AS1 |  |
|  | FITM1 |  |
|  | OTTHUMG00000160141 |  |
|  | LOC101060595 |  |
|  | OTTHUMG00000160614 |  |
|  | OTTHUMG00000056664 |  |
|  | LOC100506851 |  |
|  | PDE9A |  |
|  | SCP2D1 |  |
|  | GPX6 |  |
|  | OTTHUMG00000164388 |  |
|  | OTTHUMG00000158020 |  |
|  | LOC100996490 |  |
|  | OTTHUMG00000153205 |  |
|  | OR7D4 |  |
|  | LOC100505817 |  |
|  | KRTAP19-4 |  |
|  | RHOXF2B |  |
|  | OTTHUMG00000161647 |  |
|  | OTTHUMG00000155274 |  |
|  | TM4SF1-AS1 |  |
|  | OTTHUMG00000048096 |  |
|  | LOC285556 |  |
|  | EMX2OS |  |
|  | OTTHUMG00000059450 |  |
|  | LOC100128505 |  |
|  | VCX3A |  |
|  | GHRHR |  |
|  | MRGPRE |  |
|  | OTTHUMG00000162638 |  |
|  | LOC284950 |  |
|  | OR51F1 |  |
|  | KIR3DX1 |  |
|  | CLIC6 |  |
|  | TCP10 |  |
|  | MRO |  |
|  | SLC7A8 |  |
|  | OTTHUMG00000035263 |  |
|  | RNU5F-3P |  |
|  | OTTHUMG00000163994 |  |
|  | OTTHUMG00000066440 |  |
|  | OTTHUMG00000019281 |  |
|  | OTTHUMG00000017731 |  |
|  | LOC339666 |  |
|  | SLC39A2 |  |
|  | RTP2 |  |
|  | LOC283693 |  |
|  | GDPD4 |  |
|  | KIAA1244 |  |
|  | S100A14 |  |
|  | OTTHUMG00000162286 |  |
|  | BMPER |  |
|  | GPR139 |  |
|  | OTTHUMG00000161755 |  |
|  | IGKV1D-17 |  |
|  | OTTHUMG00000153390 |  |
|  | C4orf17 |  |
|  | OTTHUMG00000161724 |  |
|  | NUDT10 |  |
|  | CYP4F43P |  |
|  | KRT74 |  |
|  | SLC17A4 |  |
|  | ZBTB8B |  |
|  | OTTHUMG00000014768 |  |
|  | MLIP-IT1 |  |
|  | EGFR-AS1 |  |
|  | CSAG1 |  |
|  | ATG10-IT1 |  |
|  | MIR4756 |  |
|  | LINC00533 |  |
|  | LOC100130880 |  |
|  | OTTHUMG00000164229 |  |
|  | MIR885 |  |
|  | OTTHUMG00000162000 |  |
|  | OTTHUMG00000015255 |  |
|  | OTTHUMG00000163616 |  |
|  | OTTHUMG00000001239 |  |
|  | IVL |  |
|  | OTTHUMG00000162054 |  |
|  | OR1Q1 |  |
|  | KRTAP24-1 |  |
|  | LOC100505912 |  |
|  | LINC00350 |  |
|  | NLGN4Y-AS1 |  |
|  | IGLV3-16 |  |
|  | OTTHUMG00000037977 |  |
|  | OTTHUMG00000159201 |  |
|  | GPR156 |  |
|  | PRR23A |  |
|  | OTTHUMG00000035891 |  |
|  | LOC100996694 |  |
|  | OTTHUMG00000035304 |  |
|  | LGALS13 |  |
|  | LOC151009 |  |
|  | B3GALT1 |  |
|  | MYBPHL |  |
|  | ZFYVE9 |  |
|  | OTTHUMG00000163523 |  |
|  | OTTHUMG00000017483 |  |
|  | SCGB1D4 |  |
|  | C9orf57 |  |
|  | OTTHUMG00000035182 |  |
|  | OTTHUMG00000017792 |  |
|  | LOC100507325 |  |
|  | OTTHUMG00000036117 |  |
|  | OR4D6 |  |
|  | OTTHUMG00000161957 |  |
|  | PLA2G2D |  |
|  | OTTHUMG00000163790 |  |
|  | OTTHUMG00000074318 |  |
|  | SPATA6L |  |
|  | LOC100506530 |  |
|  | OTTHUMG00000018228 |  |
|  | OTTHUMG00000032826 |  |
|  | UBOX5-AS1 |  |
|  | C2orf50 |  |
|  | OTTHUMG00000161799 |  |
|  | OTTHUMG00000020594 |  |
|  | SLC51B |  |
|  | OTTHUMG00000151750 |  |
|  | OTTHUMG00000151319 |  |
|  | MIR1243 |  |
|  | OTTHUMG00000078247 |  |
|  | OTTHUMG00000154542 |  |
|  | OTTHUMG00000017527 |  |
|  | AMBN |  |
|  | OTTHUMG00000161589 |  |
|  | LINC00639 |  |
|  | OTTHUMG00000019148 |  |
|  | INSL5 |  |
|  | LOC100652770 |  |
|  | MIR4262 |  |
|  | OTTHUMG00000001777 |  |
|  | GSG1 |  |
|  | RNA5SP47 |  |
|  | OTTHUMG00000019555 |  |
|  | TISP43 |  |
|  | OTTHUMG00000008189 |  |
|  | OTTHUMG00000017397 |  |
|  | CDH7 |  |
|  | OTTHUMG00000161893 |  |
|  | OTTHUMG00000154248 |  |
|  | KRT38 |  |
|  | MIR1238 |  |
|  | LOC100133957 |  |
|  | ZC4H2 |  |
|  | LOC100287413 |  |
|  | OTTHUMG00000156460 |  |
|  | OTTHUMG00000059376 |  |
|  | OTTHUMG00000162810 |  |
|  | OTTHUMG00000152614 |  |
|  | MYL7 |  |
|  | OTTHUMG00000009770 |  |
|  | IGKV3D-20 |  |
|  | OTTHUMG00000016062 |  |
|  | OTTHUMG00000159132 |  |
|  | OTTHUMG00000161282 |  |
|  | OTTHUMG00000032784 |  |
|  | OTTHUMG00000164576 |  |
|  | CCBP2 |  |
|  | OTTHUMG00000152058 |  |
|  | UGT2B4 |  |
|  | LRIT1 |  |
|  | ZSWIM5 |  |
|  | LOC388849 |  |
|  | OTTHUMG00000010108 |  |
|  | SNORD119 |  |
|  | OTTHUMG00000165017 |  |
|  | RNA5SP496 |  |
|  | TTR |  |
|  | OTTHUMG00000020645 |  |
|  | ZP1 |  |
|  | OTTHUMG00000017965 |  |
|  | OTTHUMG00000160613 |  |
|  | OTTHUMG00000017707 |  |
|  | KC6 |  |
|  | LOC100507462 |  |
|  | LINC00261 |  |
|  | OTTHUMG00000021486 |  |
|  | CRISP1 |  |
|  | LOC340515 |  |
|  | OTTHUMG00000154103 |  |
|  | GYPB |  |
|  | OTTHUMG00000078661 |  |
|  | MUC5B |  |
|  | OTTHUMG00000156464 |  |
|  | F10-AS1 |  |
|  | OTTHUMG00000153618 |  |
|  | MIR449B |  |
|  | MYL1 |  |
|  | OTTHUMG00000078124 |  |
|  | SYN3 |  |
|  | MROH2B |  |
|  | OTTHUMG00000020432 |  |
|  | LOC645188 |  |
|  | OTTHUMG00000160964 |  |
|  | ENTPD8 |  |
|  | MIR4682 |  |
|  | IGKV1-17 |  |
|  | TNFRSF19 |  |
|  | OTTHUMG00000034550 |  |
|  | KRTAP12-3 |  |
|  | OTTHUMG00000086462 |  |
|  | OR2L1P |  |
|  | OTTHUMG00000016716 |  |
|  | OTTHUMG00000020884 |  |
|  | OTTHUMG00000164017 |  |
|  | MIR3120 |  |
|  | IGLV3-22 |  |
|  | IGHV2-70 |  |
|  | OTTHUMG00000067148 |  |
|  | LOC100509646 |  |
|  | TREH |  |
|  | OTTHUMG00000150550 |  |
|  | OTTHUMG00000040653 |  |
|  | MGC32805 |  |
|  | TPD52L3 |  |
|  | KRTAP12-4 |  |
|  | OTTHUMG00000163754 |  |
|  | OTTHUMG00000163385 |  |
|  | OTTHUMG00000161934 |  |
|  | LOC285000 |  |
|  | CTAG1B |  |
|  | RNA5SP80 |  |
|  | CDRT7 |  |
|  | OTTHUMG00000130762 |  |
|  | RNA5SP54 |  |
|  | LOC100996679 |  |
|  | 1-Dec |  |
|  | SNORD109B |  |
|  | OTTHUMG00000007457 |  |
|  | OTTHUMG00000163287 |  |
|  | HOXC13 |  |
|  | OTTHUMG00000163084 |  |
|  | OTTHUMG00000031897 |  |
|  | OTTHUMG00000160925 |  |
|  | NT5E |  |
|  | IL22RA2 |  |
|  | CYP2J2 |  |
|  | DEFB109P1 |  |
|  | LINC00871 |  |
|  | SERPINA5 |  |
|  | OTTHUMG00000156394 |  |
|  | ART1 |  |
|  | LINC00502 |  |
|  | OTTHUMG00000153867 |  |
|  | OTTHUMG00000010779 |  |
|  | G6PC2 |  |
|  | OTTHUMG00000067927 |  |
|  | DYTN |  |
|  | OTTHUMG00000039465 |  |
|  | C7orf10 |  |
|  | FLJ45974 |  |
|  | FAM57B |  |
|  | PLCL2-AS1 |  |
|  | SNORD113-8 |  |
|  | LOC100291105 |  |
|  | LOC100270804 |  |
|  | LMLN-AS1 |  |
|  | OTTHUMG00000152857 |  |
|  | MIR585 |  |
|  | SLC22A6 |  |
|  | OTTHUMG00000163744 |  |
|  | SPACA3 |  |
|  | OTTHUMG00000153881 |  |
|  | OTTHUMG00000155474 |  |
|  | OTTHUMG00000163718 |  |
|  | OTTHUMG00000035578 |  |
|  | OR4C3 |  |
|  | BAGE4 |  |
|  | CCAT1 |  |
|  | SLC6A13 |  |
|  | MASP1 |  |
|  | IGLV4-3 |  |
|  | SOX14 |  |
|  | LCE6A |  |
|  | OTTHUMG00000163283 |  |
|  | INHBA-AS1 |  |
|  | CNTNAP5 |  |
|  | COL25A1 |  |
|  | OTTHUMG00000158586 |  |
|  | OTTHUMG00000017237 |  |
|  | OTTHUMG00000157305 |  |
|  | AGBL1 |  |
|  | DYNAP |  |
|  | GPR158 |  |
|  | LOC100505986 |  |
|  | OTTHUMG00000036132 |  |
|  | OTTHUMG00000163062 |  |
|  | FAM86B2 |  |
|  | OTTHUMG00000020434 |  |
|  | SMAD5-AS1 |  |
|  | SLC35F1 |  |
|  | OTTHUMG00000015958 |  |
|  | HRCT1 |  |
|  | KIR2DL1 |  |
|  | DLG3-AS1 |  |
|  | LOC100506457 |  |
|  | SPRNP1 |  |
|  | TOP1P2 |  |
|  | OTTHUMG00000032492 |  |
|  | LOC100507166 |  |
|  | NEK2 |  |
|  | OTTHUMG00000039859 |  |
|  | UGT2B10 |  |
|  | OTTHUMG00000155746 |  |
|  | TEX37 |  |
|  | MIR1265 |  |
|  | STC1 |  |
|  | OTTHUMG00000155476 |  |
|  | OTTHUMG00000009894 |  |
|  | PAGE1 |  |
|  | ASB12 |  |
|  | OTTHUMG00000161199 |  |
|  | MIR422A |  |
|  | OTTHUMG00000018395 |  |
|  | PRSS8 |  |
|  | OTTHUMG00000161714 |  |
|  | FA2H |  |
|  | CXorf61 |  |
|  | OTTHUMG00000017366 |  |
|  | OTTHUMG00000059642 |  |
|  | OR9Q1 |  |
|  | OTTHUMG00000144163 |  |
|  | OTTHUMG00000058268 |  |
|  | LOC100505515 |  |
|  | OTTHUMG00000014329 |  |
|  | OSTN-AS1 |  |
|  | OTTHUMG00000164708 |  |
|  | SAMSN1-AS1 |  |
|  | OTTHUMG00000133345 |  |
|  | OTTHUMG00000154523 |  |
|  | IGKV6D-21 |  |
|  | OTTHUMG00000037758 |  |
|  | LINC00934 |  |
|  | OTTHUMG00000032968 |  |
|  | OTTHUMG00000153019 |  |
|  | LOC392364 |  |
|  | CNTN4-AS2 |  |
|  | MIR4681 |  |
|  | OTTHUMG00000019692 |  |
|  | OTTHUMG00000020274 |  |
|  | OTTHUMG00000162927 |  |
|  | OTTHUMG00000020784 |  |
|  | OTTHUMG00000014182 |  |
|  | OPA1-AS1 |  |
|  | PRAMEF24P |  |
|  | OTTHUMG00000008195 |  |
|  | LINC00453 |  |
|  | NPFFR1 |  |
|  | OTTHUMG00000164319 |  |
|  | AMHR2 |  |
|  | ARHGEF3-AS1 |  |
|  | OTTHUMG00000153099 |  |
|  | MRAP |  |
|  | LINC00442 |  |
|  | OTTHUMG00000037718 |  |
|  | OR52L1 |  |
|  | OTTHUMG00000163105 |  |
|  | OTTHUMG00000161925 |  |
|  | OTTHUMG00000018238 |  |
|  | OTTHUMG00000019540 |  |
|  | MXRA5P1 |  |
|  | FAM90A27P |  |
|  | LINC00210 |  |
|  | FAM216B |  |
|  | RNA5SP439 |  |
|  | LINC00160 |  |
|  | CRYGEP |  |
|  | OTTHUMG00000067150 |  |
|  | LOC340094 |  |
|  | OTTHUMG00000163904 |  |
|  | IGKV1D-42 |  |
|  | FMOD |  |
|  | SLC7A14 |  |
|  | RNA5SP278 |  |
|  | IGLV3-1 |  |
|  | OTTHUMG00000008497 |  |
|  | OTTHUMG00000021958 |  |
|  | SNORD114-9 |  |
|  | OTTHUMG00000161058 |  |
|  | LOC100507160 |  |
|  | OTTHUMG00000021363 |  |
|  | CYP4F35P |  |
|  | ORM1 |  |
|  | GOLGA6L2 |  |
|  | NMNAT2 |  |
|  | RBMS3-AS2 |  |
|  | OTTHUMG00000160074 |  |
|  | SLC5A12 |  |
|  | OTTHUMG00000086827 |  |
|  | OR13G1 |  |
|  | OTTHUMG00000021532 |  |
|  | CHRNA1 |  |
|  | OTTHUMG00000162427 |  |
|  | ULBP1 |  |
|  | MIR1275 |  |
|  | RNA5SP451 |  |
|  | OTTHUMG00000155767 |  |
|  | MIR3065 |  |
|  | OTTHUMG00000159120 |  |
|  | OTTHUMG00000065346 |  |
|  | LOC100506895 |  |
|  | FAM74A6 |  |
|  | OTTHUMG00000024096 |  |
|  | OTTHUMG00000164236 |  |
|  | SNORD114-22 |  |
|  | OTTHUMG00000150318 |  |
|  | KRTAP5-10 |  |
|  | LOC100505799 |  |
|  | OTTHUMG00000017940 |  |
|  | RERG-IT1 |  |
|  | OTTHUMG00000039911 |  |
|  | FLJ45743 |  |
|  | CPA1 |  |
|  | LOC729305 |  |
|  | OTTHUMG00000018105 |  |
|  | OTTHUMG00000086581 |  |
|  | OTTHUMG00000148711 |  |
|  | OTTHUMG00000086849 |  |
|  | OTTHUMG00000148880 |  |
|  | OTTHUMG00000149160 |  |
|  | OTTHUMG00000149412 |  |
|  | OTTHUMG00000004780 |  |
|  | OTTHUMG00000008928 |  |
|  | CD8BP |  |
|  | OTTHUMG00000020245 |  |
|  | RNA5SP90 |  |
|  | MYBPH |  |
|  | RNASE8 |  |
|  | RNA5SP300 |  |
|  | LOC100507254 |  |
|  | GABRR3 |  |
|  | CA6 |  |
|  | OTTHUMG00000132777 |  |
|  | OTTHUMG00000158757 |  |
|  | OTTHUMG00000037661 |  |
|  | OTTHUMG00000013086 |  |
|  | OTTHUMG00000164398 |  |
|  | OTTHUMG00000078125 |  |
|  | LINC00320 |  |
|  | OTTHUMG00000037959 |  |
|  | C11orf44 |  |
|  | OTTHUMG00000161885 |  |
|  | OTTHUMG00000012473 |  |
|  | OTTHUMG00000161742 |  |
|  | HIGD2B |  |
|  | OTTHUMG00000158688 |  |
|  | CRYAA |  |
|  | IFNA13 |  |
|  | MKNK1-AS1 |  |
|  | CEACAM8 |  |
|  | OTTHUMG00000161384 |  |
|  | GYG2P1 |  |
|  | OR51T1 |  |
|  | FSCN3 |  |
|  | OTTHUMG00000078773 |  |
|  | TXNDC2 |  |
|  | CEACAM20 |  |
|  | OTTHUMG00000019234 |  |
|  | LINC00941 |  |
|  | PCDH9-AS4 |  |
|  | THEG5 |  |
|  | OTTHUMG00000002945 |  |
|  | MIR2113 |  |
|  | LOC400768 |  |
|  | CT45A5 |  |
|  | LINC00637 |  |
|  | MIR634 |  |
|  | GPA33 |  |
|  | OTTHUMG00000154540 |  |
|  | OR2J1 |  |
|  | OTTHUMG00000151813 |  |
|  | LINC00485 |  |
|  | OTTHUMG00000021872 |  |
|  | OTTHUMG00000150221 |  |
|  | OTTHUMG00000032921 |  |
|  | OOSP1 |  |
|  | TMEM45B |  |
|  | LOC100506691 |  |
|  | OTTHUMG00000158236 |  |
|  | TCP10L2 |  |
|  | OTTHUMG00000009204 |  |
|  | OTTHUMG00000160984 |  |
|  | OTTHUMG00000038016 |  |
|  | CST5 |  |
|  | RNU3P3 |  |
|  | OTTHUMG00000152992 |  |
|  | LOC100129216 |  |
|  | FLJ46284 |  |
|  | OR10G9 |  |
|  | LOC729177 |  |
|  | OTTHUMG00000159892 |  |
|  | PRAMEF4 |  |
|  | OTTHUMG00000164862 |  |
|  | OR1A1 |  |
|  | SNURFL |  |
|  | CXorf48 |  |
|  | LINC00857 |  |
|  | DAND5 |  |
|  | OTTHUMG00000164609 |  |
|  | OTTHUMG00000158658 |  |
|  | OTTHUMG00000152727 |  |
|  | LOC100507477 |  |
|  | LOC283585 |  |
|  | SNORD114-19 |  |
|  | OTTHUMG00000000451 |  |
|  | OTTHUMG00000016019 |  |
|  | OTTHUMG00000022592 |  |
|  | OTTHUMG00000162551 |  |
|  | IGHV1-18 |  |
|  | OTTHUMG00000035786 |  |
|  | LINC00578 |  |
|  | OR2C1 |  |
|  | OTTHUMG00000151706 |  |
|  | OTTHUMG00000151745 |  |
|  | LOC401296 |  |
|  | OTTHUMG00000161756 |  |
|  | LOC729506 |  |
|  | RNU7-52P |  |
|  | DPT |  |
|  | CGB |  |
|  | OTTHUMG00000150930 |  |
|  | OTTHUMG00000153504 |  |
|  | OTTHUMG00000016885 |  |
|  | LINC00686 |  |
|  | OTTHUMG00000156628 |  |
|  | RNU7-62P |  |
|  | MIR4326 |  |
|  | FAM170B |  |
|  | HNF1A-AS1 |  |
|  | OTTHUMG00000159446 |  |
|  | OTTHUMG00000153027 |  |
|  | CCL19 |  |
|  | OR6N1 |  |
|  | MIR1912 |  |
|  | OTTHUMG00000159191 |  |
|  | FAM66A |  |
|  | OTTHUMG00000164327 |  |
|  | OR51G1 |  |
|  | OTTHUMG00000164255 |  |
|  | KRTAP4-11 |  |
|  | GUCY2GP |  |
|  | KRTAP1-5 |  |
|  | OTTHUMG00000161562 |  |
|  | IGHV4-31 |  |
|  | OTTHUMG00000157590 |  |
|  | OTTHUMG00000156990 |  |
|  | GPR88 |  |
|  | LCE5A |  |
|  | OTTHUMG00000156583 |  |
|  | OTTHUMG00000020101 |  |
|  | GUSBP5 |  |
|  | SRGAP3-AS4 |  |
|  | LOC150568 |  |
|  | LINC00366 |  |
|  | OTTHUMG00000155710 |  |
|  | OR52I2 |  |
|  | CYP3A4 |  |
|  | ANKFN1 |  |
|  | FLJ22763 |  |
|  | MIR548O |  |
|  | SERPIND1 |  |
|  | OTTHUMG00000162705 |  |
|  | LOC100507616 |  |
|  | GCM1 |  |
|  | OR5AU1 |  |
|  | MIR4314 |  |
|  | GNG12-AS1 |  |
|  | OTTHUMG00000161151 |  |
|  | OTTHUMG00000150928 |  |
|  | OTTHUMG00000058813 |  |
|  | SAGE1 |  |
|  | LOC339505 |  |
|  | OTTHUMG00000157643 |  |
|  | LGR5 |  |
|  | SERPINB12 |  |
|  | OTTHUMG00000031936 |  |
|  | OTTHUMG00000018929 |  |
|  | OTTHUMG00000020899 |  |
|  | OTTHUMG00000035686 |  |
|  | PRSS33 |  |
|  | OTTHUMG00000160278 |  |
|  | OTTHUMG00000032495 |  |
|  | LINC00607 |  |
|  | OTTHUMG00000035544 |  |
|  | LOC400891 |  |
|  | OTTHUMG00000014348 |  |
|  | OTTHUMG00000162112 |  |
|  | MIR4323 |  |
|  | OTTHUMG00000010913 |  |
|  | OTTHUMG00000007844 |  |
|  | OTTHUMG00000164675 |  |
|  | OTTHUMG00000017923 |  |
|  | LRRC52 |  |
|  | SLC36A2 |  |
|  | LOC645434 |  |
|  | OTTHUMG00000031898 |  |
|  | SNORD114-5 |  |
|  | HTR3C |  |
|  | OTTHUMG00000155125 |  |
|  | MIR4786 |  |
|  | MAGEA1 |  |
|  | UGT2B11 |  |
|  | CT60 |  |
|  | OTTHUMG00000163917 |  |
|  | OTTHUMG00000151900 |  |
|  | OTTHUMG00000042020 |  |
|  | MRGPRX3 |  |
|  | POU6F2-AS2 |  |
|  | LOC442028 |  |
|  | OTTHUMG00000162399 |  |
|  | OTTHUMG00000160549 |  |
|  | SFTA1P |  |
|  | OR4K14 |  |
|  | OTTHUMG00000132218 |  |
|  | SSTR5 |  |
|  | OR4N4 |  |
|  | OTTHUMG00000086643 |  |
|  | OTTHUMG00000153755 |  |
|  | OTTHUMG00000007956 |  |
|  | OTTHUMG00000012009 |  |
|  | HCG4 |  |
|  | OTTHUMG00000164866 |  |
|  | CYP4Z2P |  |
|  | OTTHUMG00000157317 |  |
|  | FAM86B3P |  |
|  | DSCR4 |  |
|  | OTTHUMG00000164340 |  |
|  | LY6G6E |  |
|  | BCL9 |  |
|  | OTTHUMG00000152017 |  |
|  | OTTHUMG00000015535 |  |
|  | OTTHUMG00000020960 |  |
|  | KRTAP1-1 |  |
|  | OTTHUMG00000020728 |  |
|  | OTTHUMG00000020648 |  |
|  | OOEP-AS1 |  |
|  | RFX4 |  |
|  | OTTHUMG00000151830 |  |
|  | IGLV11-55 |  |
|  | UGT2A3 |  |
|  | XIRP1 |  |
|  | OTTHUMG00000132354 |  |
|  | SLC13A4 |  |
|  | OTTHUMG00000157094 |  |
|  | MIR593 |  |
|  | MIR4298 |  |
|  | C2orf70 |  |
|  | OTTHUMG00000031835 |  |
|  | OTTHUMG00000162965 |  |
|  | MDH1B |  |
|  | LOC100507346 |  |
|  | FGFBP1 |  |
|  | OTTHUMG00000014269 |  |
|  | FAM26D |  |
|  | EIF5AL1 |  |
|  | OTTHUMG00000015885 |  |
|  | SNORD114-16 |  |
|  | MIR2682 |  |
|  | EPPK1 |  |
|  | OTTHUMG00000157255 |  |
|  | PDYN |  |
|  | CTXN3 |  |
|  | OR6C6 |  |
|  | OTTHUMG00000161004 |  |
|  | C10orf126 |  |
|  | NAV2-AS3 |  |
|  | SERPINA9 |  |
|  | OTTHUMG00000041089 |  |
|  | CHRM2 |  |
|  | OTTHUMG00000133700 |  |
|  | LOC100507443 |  |
|  | MIR4731 |  |
|  | POTEE |  |
|  | SMCR2 |  |
|  | AQP2 |  |
|  | OTTHUMG00000020612 |  |
|  | OTTHUMG00000160762 |  |
|  | LINC00460 |  |
|  | KRTAP19-3 |  |
|  | MIR297 |  |
|  | SDC4P |  |
|  | OTTHUMG00000017936 |  |
|  | DCX |  |
|  | OTTHUMG00000037911 |  |
|  | MIR30A |  |
|  | LOC644662 |  |
|  | OTTHUMG00000074550 |  |
|  | MIR4294 |  |
|  | OTTHUMG00000164932 |  |
|  | OTTHUMG00000164281 |  |
|  | SERPINA6 |  |
|  | CNR1 |  |
|  | OR8K5 |  |
|  | CCDC60 |  |
|  | OTTHUMG00000018553 |  |
|  | OTTHUMG00000034431 |  |
|  | OTTHUMG00000032220 |  |
|  | OTTHUMG00000164266 |  |
|  | GSTA5 |  |
|  | CTAGE10P |  |
|  | LOC100505596 |  |
|  | C1orf137 |  |
|  | CHRM1 |  |
|  | IL1F10 |  |
|  | OTTHUMG00000165055 |  |
|  | OTTHUMG00000078482 |  |
|  | OTTHUMG00000045125 |  |
|  | OTTHUMG00000163395 |  |
|  | WBP11P1 |  |
|  | DAPL1 |  |
|  | OTTHUMG00000153209 |  |
|  | OTTHUMG00000151147 |  |
|  | KRTAP10-9 |  |
|  | PAGE2B |  |
|  | OTTHUMG00000155205 |  |
|  | OTTHUMG00000019433 |  |
|  | OTTHUMG00000154047 |  |
|  | OTTHUMG00000018729 |  |
|  | OTTHUMG00000161739 |  |
|  | OTTHUMG00000163704 |  |
|  | OTTHUMG00000158937 |  |
|  | RDH8 |  |
|  | IQCF5 |  |
|  | OTTHUMG00000159155 |  |
|  | OTTHUMG00000132217 |  |
|  | MCCC1-AS1 |  |
|  | OTTHUMG00000008375 |  |
|  | HTR4 |  |
|  | OTTHUMG00000021409 |  |
|  | LOC100507091 |  |
|  | OTTHUMG00000036120 |  |
|  | C2orf71 |  |
|  | LOC440233 |  |
|  | OTTHUMG00000033126 |  |
|  | GPX5 |  |
|  | C8B |  |
|  | OTTHUMG00000163970 |  |
|  | MIR2392 |  |
|  | OTTHUMG00000010648 |  |
|  | SLC22A25 |  |
|  | OTTHUMG00000163315 |  |
|  | NPS |  |
|  | TRIM77 |  |
|  | LINC00917 |  |
|  | OTTHUMG00000032002 |  |
|  | OTTHUMG00000162001 |  |
|  | LCE2B |  |
|  | LOC339166 |  |
|  | PCDHB6 |  |
|  | OTTHUMG00000160414 |  |
|  | OTTHUMG00000019258 |  |
|  | MIR656 |  |
|  | LOC100505540 |  |
|  | OR10G4 |  |
|  | OR5H1 |  |
|  | OTTHUMG00000041543 |  |
|  | CLEC19A |  |
|  | LOC100506371 |  |
|  | OTTHUMG00000163869 |  |
|  | LNX1-AS2 |  |
|  | TAAR9 |  |
|  | OTTHUMG00000152486 |  |
|  | VWC2L |  |
|  | DLGAP1-AS5 |  |
|  | OTTHUMG00000162967 |  |
|  | MUCL1 |  |
|  | RNA5SP261 |  |
|  | KIAA1549L |  |
|  | PCDHB11 |  |
|  | RNA5SP61 |  |
|  | DMD-AS1 |  |
|  | OTTHUMG00000015806 |  |
|  | OTTHUMG00000007882 |  |
|  | PRAMEF16 |  |
|  | OTTHUMG00000015478 |  |
|  | MIR30C1 |  |
|  | OTTHUMG00000164048 |  |
|  | OTTHUMG00000018730 |  |
|  | MORC1-AS1 |  |
|  | POTEH |  |
|  | OTTHUMG00000151090 |  |
|  | MS4A10 |  |
|  | LOC646268 |  |
|  | MIR548H1 |  |
|  | AMELY |  |
|  | OTTHUMG00000161960 |  |
|  | OTTHUMG00000163248 |  |
|  | LOC100506899 |  |
|  | OR1N1 |  |
|  | OTTHUMG00000016055 |  |
|  | DPH6-AS1 |  |
|  | GAP43 |  |
|  | PLCXD2-AS1 |  |
|  | SUN5 |  |
|  | ANKRD30A |  |
|  | DUSP21 |  |
|  | FGF23 |  |
|  | NEGR1-IT1 |  |
|  | DEFB117 |  |
|  | CEACAM18 |  |
|  | GABRG3 |  |
|  | MIR103B1 |  |
|  | OTTHUMG00000152267 |  |
|  | MIR548AC |  |
|  | OTTHUMG00000018310 |  |
|  | OTTHUMG00000042022 |  |
|  | OTTHUMG00000074330 |  |
|  | OTTHUMG00000078333 |  |
|  | DKFZp779M0652 |  |
|  | DISP2 |  |
|  | OTTHUMG00000003412 |  |
|  | RNA5SP486 |  |
|  | OTTHUMG00000153939 |  |
|  | TMEM89 |  |
|  | TAS2R42 |  |
|  | FAM27L |  |
|  | C10orf82 |  |
|  | OTTHUMG00000155590 |  |
|  | UGT2B28 |  |
|  | OTTHUMG00000020015 |  |
|  | OTTHUMG00000150328 |  |
|  | RNA5SP384 |  |
|  | OTTHUMG00000151728 |  |
|  | OTTHUMG00000163524 |  |
|  | OTTHUMG00000039886 |  |
|  | IFNL3 |  |
|  | SLN |  |
|  | LPO |  |
|  | OR4C6 |  |
|  | TRIM40 |  |
|  | OTTHUMG00000153978 |  |
|  | NPAS2 |  |
|  | ZNF285 |  |
|  | OTTHUMG00000154378 |  |
|  | OTTHUMG00000057525 |  |
|  | OTTHUMG00000013252 |  |
|  | OTTHUMG00000153229 |  |
|  | OTTHUMG00000155310 |  |
|  | NALCN-AS1 |  |
|  | OTTHUMG00000150017 |  |
|  | KRTAP3-1 |  |
|  | MIR3622A |  |
|  | C12orf68 |  |
|  | MTRNR2L10 |  |
|  | LOC255187 |  |
|  | MIR769 |  |
|  | OTTHUMG00000161777 |  |
|  | LINC00651 |  |
|  | OTTHUMG00000160519 |  |
|  | LINC00582 |  |
|  | OR10G3 |  |
|  | LOC100129636 |  |
|  | KRTAP8-1 |  |
|  | RNU7-70P |  |
|  | MIR4686 |  |
|  | OTTHUMG00000018539 |  |
|  | OTTHUMG00000019257 |  |
|  | LOC100996589 |  |
|  | NBPF6 |  |
|  | CST9LP1 |  |
|  | OTTHUMG00000019301 |  |
|  | KRTAP9-3 |  |
|  | OTTHUMG00000159638 |  |
|  | RNA5SP35 |  |
|  | LOC100507489 |  |
|  | TSIX |  |
|  | SOX5 |  |
|  | OTTHUMG00000163664 |  |
|  | OTTHUMG00000017871 |  |
|  | IL19 |  |
|  | OR8B3 |  |
|  | OTTHUMG00000040658 |  |
|  | CCL22 |  |
|  | OTTHUMG00000154428 |  |
|  | LSMEM2 |  |
|  | TNR-IT1 |  |
|  | ANKRD30BP2 |  |
|  | GPR26 |  |
|  | OTTHUMG00000164280 |  |
|  | OTTHUMG00000035227 |  |
|  | OTTHUMG00000163511 |  |
|  | GPR63 |  |
|  | FHIT |  |
|  | OTTHUMG00000162923 |  |
|  | LOC400752 |  |
|  | ADAMTS9-AS2 |  |
|  | LOC100129027 |  |
|  | OTTHUMG00000019106 |  |
|  | OTTHUMG00000162198 |  |
|  | FLJ27255 |  |
|  | MIR646 |  |
|  | OTTHUMG00000159583 |  |
|  | CYP2F1 |  |
|  | ASPDH |  |
|  | SPINK4 |  |
|  | IGF1R |  |
|  | TGM7 |  |
|  | C16orf90 |  |
|  | OR4E2 |  |
|  | METTL21C |  |
|  | OTTHUMG00000066051 |  |
|  | LOC100192426 |  |
|  | LOC100130673 |  |
|  | OTTHUMG00000151737 |  |
|  | DEFB118 |  |
|  | LOC100505827 |  |
|  | SMC5-AS1 |  |
|  | OTTHUMG00000151487 |  |
|  | OR4S1 |  |
|  | OTTHUMG00000154909 |  |
|  | OTTHUMG00000158383 |  |
|  | DMRTC1B |  |
|  | RNA5SP398 |  |
|  | PART1 |  |
|  | OTTHUMG00000155047 |  |
|  | STMN2 |  |
|  | IGHV3-30 |  |
|  | OTTHUMG00000015922 |  |
|  | LOC100505664 |  |
|  | MIR2117 |  |
|  | GJA10 |  |
|  | PROP1 |  |
|  | OTTHUMG00000153158 |  |
|  | TTC3-AS1 |  |
|  | CACNA1C-AS2 |  |
|  | OTTHUMG00000161063 |  |
|  | MIR346 |  |
|  | NOS2P2 |  |
|  | ZPLD1 |  |
|  | OTTHUMG00000161360 |  |
|  | OTTHUMG00000033102 |  |
|  | DEFB4B |  |
|  | SLC10A2 |  |
|  | RNA5SP87 |  |
|  | LOC100996524 |  |
|  | OTTHUMG00000161438 |  |
|  | OTTHUMG00000020937 |  |
|  | S100A7A |  |
|  | OTTHUMG00000161390 |  |
|  | NCR2 |  |
|  | RNF133 |  |
|  | OTTHUMG00000151472 |  |
|  | OTTHUMG00000161875 |  |
|  | IGKV2-29 |  |
|  | OTTHUMG00000163874 |  |
|  | SERPINE3 |  |
|  | XAGE-4 |  |
|  | DEFA10P |  |
|  | MIR1250 |  |
|  | SATB1 |  |
|  | PSG4 |  |
|  | OTTHUMG00000037143 |  |
|  | RNA5SP446 |  |
|  | OTTHUMG00000163854 |  |
|  | OTTHUMG00000009218 |  |
|  | MPRIP-AS1 |  |
|  | MIR767 |  |
|  | SMCR9 |  |
|  | KRTAP12-2 |  |
|  | OTTHUMG00000015653 |  |
|  | OTTHUMG00000163746 |  |
|  | OTTHUMG00000059736 |  |
|  | OTTHUMG00000016063 |  |
|  | OTTHUMG00000161457 |  |
|  | OTTHUMG00000059635 |  |
|  | OTTHUMG00000158345 |  |
|  | OTTHUMG00000031892 |  |
|  | TRAV8-7 |  |
|  | OTTHUMG00000018052 |  |
|  | KRT75 |  |
|  | C15orf65 |  |
|  | OTTHUMG00000150069 |  |
|  | OTTHUMG00000163999 |  |
|  | OTTHUMG00000161703 |  |
|  | RNA5SP57 |  |
|  | RNA5SP58 |  |
|  | OTTHUMG00000162017 |  |
|  | OTTHUMG00000153495 |  |
|  | OTTHUMG00000151821 |  |
|  | LOC642426 |  |
|  | IGHV5-51 |  |
|  | RNA5SP89 |  |
|  | CDH6 |  |
|  | CDH23-AS1 |  |
|  | LOC442132 |  |
|  | NHEG1 |  |
|  | LINC00269 |  |
|  | OR5M10 |  |
|  | OTTHUMG00000032780 |  |
|  | GLIS3-AS1 |  |
|  | OTTHUMG00000164158 |  |
|  | RGR |  |
|  | ZNF705G |  |
|  | OTTHUMG00000014232 |  |
|  | OTTHUMG00000013630 |  |
|  | ERC2-IT1 |  |
|  | OTTHUMG00000019216 |  |
|  | IGHV3-9 |  |
|  | OTTHUMG00000153177 |  |
|  | OTTHUMG00000018480 |  |
|  | GUCA2A |  |
|  | MIR4313 |  |
|  | OTTHUMG00000042245 |  |
|  | OTTHUMG00000015101 |  |
|  | LOC100509541 |  |
|  | OTTHUMG00000150015 |  |
|  | OTTHUMG00000018301 |  |
|  | OR1L1 |  |
|  | PANX3 |  |
|  | OTTHUMG00000164912 |  |
|  | OTTHUMG00000015822 |  |
|  | LOC339568 |  |
|  | CDH12 |  |
|  | LINC00675 |  |
|  | AWAT1 |  |
|  | CSTF3-AS1 |  |
|  | OTTHUMG00000151842 |  |
|  | DYNLL1-AS1 |  |
|  | RNA5SP341 |  |
|  | AVPR2 |  |
|  | CHEK2P2 |  |
|  | OR6C68 |  |
|  | LOC146513 |  |
|  | ZNF705E |  |
|  | IGFL3 |  |
|  | OTTHUMG00000059261 |  |
|  | OTTHUMG00000015689 |  |
|  | OTTHUMG00000041463 |  |
|  | OTTHUMG00000165018 |  |
|  | LOC729911 |  |
|  | C9orf141 |  |
|  | MIR516A1 |  |
|  | OTTHUMG00000162673 |  |
|  | OTTHUMG00000018556 |  |
|  | OTTHUMG00000151992 |  |
|  | CD5L |  |
|  | CH25H |  |
|  | OTTHUMG00000158954 |  |
|  | OTTHUMG00000161648 |  |
|  | OTTHUMG00000016828 |  |
|  | OTTHUMG00000019201 |  |
|  | OTTHUMG00000022173 |  |
|  | OTTHUMG00000160203 |  |
|  | OTTHUMG00000015619 |  |
|  | CDRT15P2 |  |
|  | HPN-AS1 |  |
|  | XKRY2 |  |
|  | OTTHUMG00000014652 |  |
|  | OTTHUMG00000150907 |  |
|  | ADIG |  |
|  | GDF9 |  |
|  | OTTHUMG00000128937 |  |
|  | OTTHUMG00000150950 |  |
|  | OTTHUMG00000163201 |  |
|  | OTTHUMG00000046113 |  |
|  | LOC100506025 |  |
|  | OTTHUMG00000034840 |  |
|  | CTSL1P2 |  |
|  | OTTHUMG00000151352 |  |
|  | MSMB |  |
|  | MIR3925 |  |
|  | OTTHUMG00000164617 |  |
|  | OTTHUMG00000015310 |  |
|  | OTTHUMG00000046223 |  |
|  | OTTHUMG00000037425 |  |
|  | OTTHUMG00000161339 |  |
|  | RNA5SP497 |  |
|  | IGKV6-21 |  |
|  | OTTHUMG00000155653 |  |
|  | FAM25C |  |
|  | OTTHUMG00000008154 |  |
|  | OTTHUMG00000152489 |  |
|  | TBC1D4-AS1 |  |
|  | GOLGA6L1 |  |
|  | MIR134 |  |
|  | LOC100505478 |  |
|  | OTTHUMG00000032810 |  |
|  | OTTHUMG00000034588 |  |
|  | POU6F2 |  |
|  | OR51J1 |  |
|  | LOC284801 |  |
|  | LINC00645 |  |
|  | ANKRD35 |  |
|  | OTTHUMG00000162401 |  |
|  | OTTHUMG00000153378 |  |
|  | ZNF204P |  |
|  | DEFB128 |  |
|  | OTTHUMG00000161060 |  |
|  | OTTHUMG00000156107 |  |
|  | OTTHUMG00000151895 |  |
|  | OTTHUMG00000014288 |  |
|  | OR1K1 |  |
|  | LOC100506647 |  |
|  | RBM11 |  |
|  | OTTHUMG00000151855 |  |
|  | INHA |  |
|  | OTTHUMG00000160388 |  |
|  | OTTHUMG00000031877 |  |
|  | OTTHUMG00000018120 |  |
|  | ISM1 |  |
|  | LOC100505718 |  |
|  | OTTHUMG00000163878 |  |
|  | LOC100506172 |  |
|  | OTTHUMG00000032718 |  |
|  | PCCA-AS1 |  |
|  | OTTHUMG00000020686 |  |
|  | OTTHUMG00000160432 |  |
|  | OTTHUMG00000151708 |  |
|  | OTTHUMG00000157236 |  |
|  | GRM7-AS1 |  |
|  | LOC100506414 |  |
|  | LINC00939 |  |
|  | OTTHUMG00000162067 |  |
|  | OTTHUMG00000014933 |  |
|  | LOC100505685 |  |
|  | C9orf152 |  |
|  | OTTHUMG00000162244 |  |
|  | OTTHUMG00000162012 |  |
|  | LOC100652856 |  |
|  | OTTHUMG00000156952 |  |
|  | LOC100653243 |  |
|  | RPS11P6 |  |
|  | OR4C5 |  |
|  | RNA5SP28 |  |
|  | TMPRSS11GP |  |
|  | OTTHUMG00000151492 |  |
|  | OTTHUMG00000004936 |  |
|  | MIR4524A |  |
|  | MAGEA12 |  |
|  | NR1I3 |  |
|  | RIC3 |  |
|  | OTTHUMG00000015979 |  |
|  | PRSS55 |  |
|  | C16orf89 |  |
|  | OTTHUMG00000150377 |  |
|  | LCE1A |  |
|  | OTTHUMG00000163170 |  |
|  | TRBV10-2 |  |
|  | OTTHUMG00000014082 |  |
|  | OTTHUMG00000015884 |  |
|  | FAM27E3 |  |
|  | OTTHUMG00000011632 |  |
|  | OTTHUMG00000074727 |  |
|  | SNORD114-11 |  |
|  | OTTHUMG00000015719 |  |
|  | OR5P3 |  |
|  | HS6ST3 |  |
|  | OTTHUMG00000163057 |  |
|  | OTTHUMG00000154647 |  |
|  | LOC150577 |  |
|  | OTTHUMG00000020567 |  |
|  | TRIM31-AS1 |  |
|  | OTTHUMG00000131063 |  |
|  | OTTHUMG00000148917 |  |
|  | OTTHUMG00000149197 |  |
|  | OTTHUMG00000149445 |  |
|  | OTTHUMG00000004813 |  |
|  | OTTHUMG00000131239 |  |
|  | TPTE2P1 |  |
|  | OTTHUMG00000153875 |  |
|  | MIR3689F |  |
|  | OR51I1 |  |
|  | OTTHUMG00000156495 |  |
|  | OTTHUMG00000155553 |  |
|  | RNA5SP96 |  |
|  | OTTHUMG00000156146 |  |
|  | RNA5SP213 |  |
|  | OTTHUMG00000162997 |  |
|  | OTTHUMG00000008075 |  |
|  | MIR3138 |  |
|  | OTTHUMG00000157272 |  |
|  | OR14A2 |  |
|  | LINC00615 |  |
|  | IL21-AS1 |  |
|  | OTTHUMG00000002515 |  |
|  | OTTHUMG00000157034 |  |
|  | MMP13 |  |
|  | MIR3155B |  |
|  | LCE4A |  |
|  | PCDH10 |  |
|  | WBP2P1 |  |
|  | LOC400558 |  |
|  | CPS1-IT1 |  |
|  | OTTHUMG00000002880 |  |
|  | MIR548AL |  |
|  | OTTHUMG00000055982 |  |
|  | C21orf62 |  |
|  | FLJ38668 |  |
|  | OTTHUMG00000012285 |  |
|  | REG1P |  |
|  | SLC14A2 |  |
|  | OTTHUMG00000150677 |  |
|  | LOC100507388 |  |
|  | OTTHUMG00000017626 |  |
|  | OTTHUMG00000161644 |  |
|  | BTNL10 |  |
|  | OTTHUMG00000015667 |  |
|  | OTTHUMG00000158223 |  |
|  | DEFB114 |  |
|  | OTTHUMG00000002408 |  |
|  | OTTHUMG00000165079 |  |
|  | OTTHUMG00000153696 |  |
|  | UPK2 |  |
|  | C21orf128 |  |
|  | OTTHUMG00000161824 |  |
|  | MIR4732 |  |
|  | OTTHUMG00000163188 |  |
|  | OTTHUMG00000162884 |  |
|  | LINC00619 |  |
|  | OTTHUMG00000164937 |  |
|  | FLJ41278 |  |
|  | OTTHUMG00000154970 |  |
|  | OTTHUMG00000002525 |  |
|  | WFDC11 |  |
|  | OR3A4P |  |
|  | OTTHUMG00000019888 |  |
|  | ARGFX |  |
|  | HTR3E-AS1 |  |
|  | OTTHUMG00000154090 |  |
|  | OTTHUMG00000074543 |  |
|  | MIR3909 |  |
|  | LOC348761 |  |
|  | RNU1-23P |  |
|  | PIP |  |
|  | OTTHUMG00000151764 |  |
|  | LOC100130452 |  |
|  | OTTHUMG00000160354 |  |
|  | OTTHUMG00000151772 |  |
|  | AQP7P1 |  |
|  | LOC100506937 |  |
|  | LGALS17A |  |
|  | OR5L2 |  |
|  | RNA5SP53 |  |
|  | OTTHUMG00000162830 |  |
|  | OTTHUMG00000156487 |  |
|  | OR4K2 |  |
|  | MIR4533 |  |
|  | FOXI1 |  |
|  | OTTHUMG00000033140 |  |
|  | IFNA16 |  |
|  | DNTT |  |
|  | SORCS3-AS1 |  |
|  | RPRM |  |
|  | OTTHUMG00000162519 |  |
|  | OTTHUMG00000017562 |  |
|  | SLC5A7 |  |
|  | IGHV3-7 |  |
|  | LINC00454 |  |
|  | OR10AD1 |  |
|  | IGHD2-21 |  |
|  | OTTHUMG00000163621 |  |
|  | OTTHUMG00000022271 |  |
|  | OTTHUMG00000161646 |  |
|  | OTTHUMG00000151428 |  |
|  | OTTHUMG00000156144 |  |
|  | OTTHUMG00000009889 |  |
|  | COL4A2-AS2 |  |
|  | OTTHUMG00000164211 |  |
|  | OTTHUMG00000163861 |  |
|  | LGALS14 |  |
|  | TMEM233 |  |
|  | OTTHUMG00000151100 |  |
|  | OTTHUMG00000153942 |  |
|  | OMD |  |
|  | OTTHUMG00000161630 |  |
|  | OTTHUMG00000150957 |  |
|  | CACNG3 |  |
|  | LINC00200 |  |
|  | OTTHUMG00000161289 |  |
|  | RNA5SP31 |  |
|  | KRTAP1-4 |  |
|  | OTTHUMG00000018648 |  |
|  | OTTHUMG00000129094 |  |
|  | OTTHUMG00000154332 |  |
|  | RNA5SP364 |  |
|  | OTTHUMG00000021313 |  |
|  | CDR1 |  |
|  | OTTHUMG00000163871 |  |
|  | OTTHUMG00000155113 |  |
|  | OTTHUMG00000164213 |  |
|  | ADH1A |  |
|  | CRYGC |  |
|  | OTTHUMG00000155199 |  |
|  | OR5K3 |  |
|  | OTTHUMG00000035112 |  |
|  | OTTHUMG00000153091 |  |
|  | IGHV2-5 |  |
|  | OTTHUMG00000032148 |  |
|  | C11orf86 |  |
|  | LOC100506643 |  |
|  | OTTHUMG00000152809 |  |
|  | OTTHUMG00000041137 |  |
|  | MIR139 |  |
|  | OTTHUMG00000152001 |  |
|  | CPN2 |  |
|  | LOC100506101 |  |
|  | ADIPOQ-AS1 |  |
|  | OTTHUMG00000014664 |  |
|  | OTTHUMG00000151796 |  |
|  | IFNA14 |  |
|  | OTTHUMG00000152056 |  |
|  | OTTHUMG00000020009 |  |
|  | OTTHUMG00000153754 |  |
|  | SPATA31D3 |  |
|  | ZNF157 |  |
|  | OTTHUMG00000161768 |  |
|  | MIR2276 |  |
|  | OTTHUMG00000078706 |  |
|  | OTTHUMG00000155092 |  |
|  | KCNQ1DN |  |
|  | OTTHUMG00000156272 |  |
|  | OTTHUMG00000014261 |  |
|  | OTTHUMG00000034877 |  |
|  | ADH1C |  |
|  | OTTHUMG00000009400 |  |
|  | OTTHUMG00000150955 |  |
|  | MIR4283-1 |  |
|  | OTTHUMG00000161894 |  |
|  | OTTHUMG00000020457 |  |
|  | OTTHUMG00000032811 |  |
|  | OTTHUMG00000021161 |  |
|  | OTTHUMG00000160773 |  |
|  | C14orf132 |  |
|  | OTTHUMG00000134296 |  |
|  | TAC4 |  |
|  | OTTHUMG00000040077 |  |
|  | PBOV1 |  |
|  | RNA5SP40 |  |
|  | MIR676 |  |
|  | KRTAP19-2 |  |
|  | OTTHUMG00000024097 |  |
|  | OR8B12 |  |
|  | ZP2 |  |
|  | OTTHUMG00000141319 |  |
|  | OTTHUMG00000156041 |  |
|  | PRAMEF21 |  |
|  | OR10G7 |  |
|  | OTTHUMG00000153551 |  |
|  | OTTHUMG00000032534 |  |
|  | OIT3 |  |
|  | OTTHUMG00000162772 |  |
|  | LINC00162 |  |
|  | LOC388780 |  |
|  | MIR3622B |  |
|  | TNNI1 |  |
|  | SPRR2A |  |
|  | LOC100996448 |  |
|  | EGFLAM-AS2 |  |
|  | MIR4757 |  |
|  | EGFLAM-AS4 |  |
|  | TOX3 |  |
|  | OTTHUMG00000153144 |  |
|  | FAM41AY1 |  |
|  | FAM41AY2 |  |
|  | FIBIN |  |
|  | MIR1229 |  |
|  | RNA5SP447 |  |
|  | GPR31 |  |
|  | TRBV20OR9-2 |  |
|  | RAET1L |  |
|  | OTTHUMG00000022722 |  |
|  | OTTHUMG00000149338 |  |
|  | OTTHUMG00000140118 |  |
|  | IGKV1-27 |  |
|  | SMR3A |  |
|  | OTTHUMG00000031842 |  |
|  | ERBB4 |  |
|  | MIR378F |  |
|  | OTTHUMG00000161032 |  |
|  | LOC285629 |  |
|  | OTTHUMG00000046204 |  |
|  | OTTHUMG00000151209 |  |
|  | LOC100506128 |  |
|  | OR3A2 |  |
|  | LOC441025 |  |
|  | OTTHUMG00000156967 |  |
|  | OR8H3 |  |
|  | MIR2467 |  |
|  | ERVW-1 |  |
|  | OTTHUMG00000041441 |  |
|  | KRTAP10-7 |  |
|  | LRIT2 |  |
|  | SPRR2E |  |
|  | RIPPLY1 |  |
|  | OTTHUMG00000000506 |  |
|  | KRTAP9-7 |  |
|  | IRG1 |  |
|  | LOC339535 |  |
|  | OTTHUMG00000066714 |  |
|  | OTTHUMG00000153102 |  |
|  | LINC00505 |  |
|  | TAS2R41 |  |
|  | CNGA2 |  |
|  | SNORD114-17 |  |
|  | OTTHUMG00000157724 |  |
|  | OTTHUMG00000022051 |  |
|  | OTTHUMG00000160663 |  |
|  | OTTHUMG00000164763 |  |
|  | MIR1539 |  |
|  | OTTHUMG00000010986 |  |
|  | CXorf28 |  |
|  | TTTY16 |  |
|  | OTTHUMG00000161692 |  |
|  | LOC100505633 |  |
|  | RNA5SP453 |  |
|  | IGHV2-26 |  |
|  | RNA5SP408 |  |
|  | EPHX2 |  |
|  | ARHGEF7-AS1 |  |
|  | OTTHUMG00000152532 |  |
|  | IGKV1D-33 |  |
|  | PSG9 |  |
|  | MIR4672 |  |
|  | MIR105-2 |  |
|  | KRTAP23-1 |  |
|  | MIR4689 |  |
|  | OTTHUMG00000156053 |  |
|  | GHSR |  |
|  | GABRA3 |  |
|  | LOC340073 |  |
|  | OTTHUMG00000133703 |  |
|  | OR2A5 |  |
|  | PSG3 |  |
|  | OTTHUMG00000031808 |  |
|  | LINC00381 |  |
|  | ERVH48-1 |  |
|  | SNORD91B |  |
|  | OTTHUMG00000133420 |  |
|  | OTTHUMG00000156711 |  |
|  | PRAMEF20 |  |
|  | OTTHUMG00000155987 |  |
|  | OTTHUMG00000156655 |  |
|  | OTTHUMG00000017710 |  |
|  | OTTHUMG00000162658 |  |
|  | OR6C1 |  |
|  | LOC401134 |  |
|  | MIR603 |  |
|  | LYPD8 |  |
|  | OTTHUMG00000163335 |  |
|  | OTTHUMG00000156065 |  |
|  | ACVR1C |  |
|  | OTTHUMG00000154786 |  |
|  | OTTHUMG00000016904 |  |
|  | OR2AP1 |  |
|  | CTAGE1 |  |
|  | SNRPD2P2 |  |
|  | OTTHUMG00000010691 |  |
|  | MIR526B |  |
|  | DEFB123 |  |
|  | POTEJ |  |
|  | OTTHUMG00000160328 |  |
|  | OTTHUMG00000161631 |  |
|  | OTTHUMG00000152589 |  |
|  | PRPH |  |
|  | TRBV5-3 |  |
|  | PRAMEF9 |  |
|  | MIR3169 |  |
|  | LINC00864 |  |
|  | MRGPRX2 |  |
|  | FOXB1 |  |
|  | OTTHUMG00000161793 |  |
|  | OTTHUMG00000067215 |  |
|  | OTTHUMG00000152846 |  |
|  | OTTHUMG00000163518 |  |
|  | OTTHUMG00000015510 |  |
|  | OTTHUMG00000014196 |  |
|  | MSGN1 |  |
|  | LOC339622 |  |
|  | LOC728228 |  |
|  | RNA5SP139 |  |
|  | OTTHUMG00000161962 |  |
|  | OTTHUMG00000154592 |  |
|  | RNA5SP70 |  |
|  | OTTHUMG00000160711 |  |
|  | KRTAP20-2 |  |
|  | LINC00508 |  |
|  | MIR4717 |  |
|  | OTTHUMG00000161243 |  |
|  | PTGER4P2-CDK2AP2P2 |  |
|  | PLXDC1 |  |
|  | MIR105-1 |  |
|  | LINC00484 |  |
|  | LOC648691 |  |
|  | CCL18 |  |
|  | ULBP2 |  |
|  | BCAR4 |  |
|  | WBSCR28 |  |
|  | IGKV1D-8 |  |
|  | OTTHUMG00000163519 |  |
|  | OTTHUMG00000163061 |  |
|  | OTTHUMG00000018903 |  |
|  | MIR548AJ2 |  |
|  | OTTHUMG00000164494 |  |
|  | OTTHUMG00000152641 |  |
|  | LOC285692 |  |
|  | OTTHUMG00000161720 |  |
|  | OTTHUMG00000156611 |  |
|  | OTTHUMG00000152941 |  |
|  | OTTHUMG00000162058 |  |
|  | SSX1 |  |
|  | IGKV1D-13 |  |
|  | OTTHUMG00000150992 |  |
|  | SNAR-A12 |  |
|  | OTTHUMG00000020447 |  |
|  | SLIT2-IT1 |  |
|  | OTTHUMG00000163279 |  |
|  | OTTHUMG00000021356 |  |
|  | OTTHUMG00000032809 |  |
|  | MIR548AA1 |  |
|  | OTTHUMG00000156290 |  |
|  | OTTHUMG00000037897 |  |
|  | OTTHUMG00000160325 |  |
|  | MIR3165 |  |
|  | RNA5SP288 |  |
|  | OTTHUMG00000018511 |  |
|  | OTTHUMG00000014342 |  |
|  | LOC339298 |  |
|  | KRTAP5-4 |  |
|  | MIR584 |  |
|  | LOC100996624 |  |
|  | SSTR3 |  |
|  | OTTHUMG00000020550 |  |
|  | IFNA17 |  |
|  | ZSCAN12P1 |  |
|  | SCGB1A1 |  |
|  | OTTHUMG00000156604 |  |
|  | SPTLC3 |  |
|  | MIR4437 |  |
|  | KRT20 |  |
|  | OTTHUMG00000163574 |  |
|  | OTTHUMG00000132154 |  |
|  | LOC730159 |  |
|  | POTEF |  |
|  | TRBV6-5 |  |
|  | OTTHUMG00000032498 |  |
|  | OTTHUMG00000157591 |  |
|  | PSG2 |  |
|  | OR2A2 |  |
|  | OTTHUMG00000153202 |  |
|  | OTTHUMG00000163056 |  |
|  | OR2J2 |  |
|  | OTTHUMG00000160761 |  |
|  | PSG7 |  |
|  | OTTHUMG00000008925 |  |
|  | C1orf147 |  |
|  | OTTHUMG00000161359 |  |
|  | POTEI |  |
|  | C2orf27B |  |
|  | COLEC10 |  |
|  | CYP3A7 |  |
|  | OTTHUMG00000036265 |  |
|  | OTTHUMG00000151712 |  |
|  | DEFB136 |  |
|  | MIR3692 |  |
|  | OTTHUMG00000014145 |  |
|  | LOC151484 |  |
|  | OTTHUMG00000162886 |  |
|  | OTTHUMG00000154823 |  |
|  | RNU7-34P |  |
|  | RNY3P10 |  |
|  | PIK3IP1 |  |
|  | OTTHUMG00000151781 |  |
|  | MIR4656 |  |
|  | OTTHUMG00000008935 |  |
|  | ADAMTS9-AS1 |  |
|  | LOC730081 |  |
|  | OTTHUMG00000018510 |  |
|  | OR7E14P |  |
|  | C12orf36 |  |
|  | OTTHUMG00000164306 |  |
|  | OTTHUMG00000032716 |  |
|  | IGHV7-81 |  |
|  | FAM106CP |  |
|  | DCUN1D2-AS2 |  |
|  | LINC00488 |  |
|  | MIR3154 |  |
|  | OR2Y1 |  |
|  | OTTHUMG00000152671 |  |
|  | OTTHUMG00000163659 |  |
|  | RP1L1 |  |
|  | HLTF-AS1 |  |
|  | LOC100507194 |  |
|  | OTTHUMG00000021819 |  |
|  | OTTHUMG00000161691 |  |
|  | OTTHUMG00000164828 |  |
|  | OTTHUMG00000002332 |  |
|  | IGSF5 |  |
|  | LOC100133286 |  |
|  | PRAMEF10 |  |
|  | OTTHUMG00000040080 |  |
|  | OTTHUMG00000152753 |  |
|  | RNA5SP120 |  |
|  | KRTAP9-6 |  |
|  | OTTHUMG00000158944 |  |
|  | OTTHUMG00000014828 |  |
|  | LOC285627 |  |
|  | OTTHUMG00000036103 |  |
|  | OTTHUMG00000163534 |  |
|  | OTTHUMG00000014238 |  |
|  | OTTHUMG00000015187 |  |
|  | LINC00184 |  |
|  | LGALS16 |  |
|  | CXorf67 |  |
|  | IGLV3-25 |  |
|  | MIR3650 |  |
|  | AMELX |  |
|  | TRBV6-6 |  |
|  | OR9A4 |  |
|  | MAGEA5 |  |
|  | NTM-IT2 |  |
|  | OTTHUMG00000008263 |  |
|  | SCGB1B2P |  |
|  | RNA5SP417 |  |
|  | OTTHUMG00000162702 |  |
|  | OTTHUMG00000015908 |  |
|  | OTTHUMG00000164347 |  |
|  | OTTHUMG00000015949 |  |
|  | WFDC13 |  |
|  | OTTHUMG00000152394 |  |
|  | OTTHUMG00000036254 |  |
|  | FLJ43315 |  |
|  | SNORD88C |  |
|  | TRIM43B |  |
|  | ST8SIA6-AS1 |  |
|  | OTTHUMG00000040863 |  |
|  | OTTHUMG00000160363 |  |
|  | OTTHUMG00000035513 |  |
|  | OTTHUMG00000164263 |  |
|  | PLCE1-AS1 |  |
|  | REG1B |  |
|  | MIR4698 |  |
|  | OTTHUMG00000162847 |  |
|  | TAAR1 |  |
|  | TGFA-IT1 |  |
|  | OTTHUMG00000161813 |  |
|  | OTTHUMG00000156193 |  |
|  | OTTHUMG00000013379 |  |
|  | OTTHUMG00000017554 |  |
|  | MAGEE2 |  |
|  | FLJ46361 |  |
|  | IL36A |  |
|  | OTTHUMG00000032678 |  |
|  | OTTHUMG00000041395 |  |
|  | OTTHUMG00000160737 |  |
|  | SULT1C3 |  |
|  | OTTHUMG00000159799 |  |
|  | GP5 |  |
|  | LOC100133077 |  |
|  | LOC728084 |  |
|  | OTTHUMG00000156217 |  |
|  | OTTHUMG00000037527 |  |
|  | LINC00940 |  |
|  | MIR197 |  |
|  | OTTHUMG00000160727 |  |
|  | POTEM |  |
|  | KRT25 |  |
|  | LOC100506384 |  |
|  | OTTHUMG00000155424 |  |
|  | LOC645949 |  |
|  | MIR4516 |  |
|  | OTTHUMG00000040690 |  |
|  | OTTHUMG00000161607 |  |
|  | OR8S1 |  |
|  | POTED |  |
|  | C16orf47 |  |
|  | LALBA |  |
|  | OTTHUMG00000151775 |  |
|  | PRSS37 |  |
|  | OTTHUMG00000021679 |  |
|  | OTTHUMG00000086761 |  |
|  | OTTHUMG00000164537 |  |
|  | LOC643401 |  |
|  | MIR211 |  |
|  | CXADR |  |
|  | SPATA31A6 |  |
|  | OTTHUMG00000160976 |  |
|  | LINC00355 |  |
|  | MAB21L2 |  |
|  | OTTHUMG00000013244 |  |
|  | LINC00052 |  |
|  | MIR514B |  |
|  | C16orf97 |  |
|  | OTTHUMG00000151740 |  |
|  | OTTHUMG00000158945 |  |
|  | OTTHUMG00000164182 |  |
|  | OTTHUMG00000021111 |  |
|  | OTTHUMG00000155481 |  |
|  | TPRXL |  |
|  | OTTHUMG00000159595 |  |
|  | GPRASP1 |  |
|  | OTTHUMG00000017942 |  |
|  | OTTHUMG00000163757 |  |
|  | OTTHUMG00000160918 |  |
|  | OTTHUMG00000019205 |  |
|  | REG3A |  |
|  | OTTHUMG00000164458 |  |
|  | OTTHUMG00000161606 |  |
|  | RNA5SP430 |  |
|  | CNKSR2 |  |
|  | OTTHUMG00000150002 |  |
|  | SCGB1D1 |  |
|  | OTTHUMG00000162876 |  |
|  | LINC00587 |  |
|  | LOC100996663 |  |
|  | OTTHUMG00000034433 |  |
|  | OTTHUMG00000009067 |  |
|  | OTTHUMG00000164166 |  |
|  | ZNF385D-AS1 |  |
|  | OTTHUMG00000015993 |  |
|  | OTTHUMG00000032101 |  |
|  | RNA5SP427 |  |
|  | SSX5 |  |
|  | OTTHUMG00000161722 |  |
|  | OTTHUMG00000015204 |  |
|  | MIR4291 |  |
|  | OTTHUMG00000163989 |  |
|  | OR2T12 |  |
|  | OTTHUMG00000078243 |  |
|  | KRTAP21-2 |  |
|  | OTTHUMG00000002621 |  |
|  | OTTHUMG00000130102 |  |
|  | OTTHUMG00000161907 |  |
|  | TRPC7-AS2 |  |
|  | OTTHUMG00000164250 |  |
|  | OTTHUMG00000017549 |  |
|  | OR2AG1 |  |
|  | OTTHUMG00000158832 |  |
|  | OTTHUMG00000086815 |  |
|  | OTTHUMG00000018041 |  |
|  | RNA5SP326 |  |
|  | LINC00458 |  |
|  | OR6T1 |  |
|  | LINC00632 |  |
|  | OTTHUMG00000161882 |  |
|  | S100A2 |  |
|  | IGHV3-23 |  |
|  | TRAV9-1 |  |
|  | OTTHUMG00000045406 |  |
|  | LOC100652994 |  |
|  | OTTHUMG00000160309 |  |
|  | WASIR1 |  |
|  | OTTHUMG00000163017 |  |
|  | FAM205B |  |
|  | OTTHUMG00000019909 |  |
|  | OTTHUMG00000008359 |  |
|  | OR3A1 |  |
|  | SNORD114-28 |  |
|  | OTTHUMG00000164044 |  |
|  | OTTHUMG00000154152 |  |
|  | NTM-IT1 |  |
|  | OTTHUMG00000156392 |  |
|  | OTTHUMG00000163915 |  |
|  | OTTHUMG00000142828 |  |
|  | EXPH5 |  |
|  | OTTHUMG00000017117 |  |
|  | SORCS3 |  |
|  | OTTHUMG00000162917 |  |
|  | LINC00499 |  |
|  | LOC728040 |  |
|  | MIR3935 |  |
|  | OTTHUMG00000164852 |  |
|  | OTTHUMG00000153710 |  |
|  | MUC16 |  |
|  | OTTHUMG00000013242 |  |
|  | KRTAP19-1 |  |
|  | OTTHUMG00000020086 |  |
|  | IGKV4-1 |  |
|  | OTTHUMG00000162899 |  |
|  | IGHV3-33 |  |
|  | OR1A2 |  |
|  | OTTHUMG00000014898 |  |
|  | LOC339975 |  |
|  | OR2G6 |  |
|  | OTTHUMG00000152839 |  |
|  | OTTHUMG00000078257 |  |
|  | OTTHUMG00000013470 |  |
|  | CCL7 |  |
|  | OTTHUMG00000151543 |  |
|  | RNY1P4 |  |
|  | OTTHUMG00000154310 |  |
|  | OTTHUMG00000153313 |  |
|  | OTTHUMG00000017531 |  |
|  | OTTHUMG00000019177 |  |
|  | MIR4268 |  |
|  | STEAP1B |  |
|  | LOC100506688 |  |
|  | OTTHUMG00000014892 |  |
|  | OTTHUMG00000159649 |  |
|  | CACHD1 |  |
|  | OTTHUMG00000032886 |  |
|  | OTTHUMG00000153690 |  |
|  | OTTHUMG00000161731 |  |
|  | OTTHUMG00000159203 |  |
|  | OR13J1 |  |
|  | KRTAP6-3 |  |
|  | OTTHUMG00000163716 |  |
|  | LOC100130954 |  |
|  | OTTHUMG00000161626 |  |
|  | OTTHUMG00000164760 |  |
|  | OTTHUMG00000018545 |  |
|  | OTTHUMG00000160917 |  |
|  | DEFB105A |  |
|  | LINC00927 |  |
|  | OTTHUMG00000164278 |  |
|  | OTTHUMG00000017564 |  |
|  | OTTHUMG00000160310 |  |
|  | OTTHUMG00000164880 |  |
|  | OTTHUMG00000153108 |  |
|  | OTTHUMG00000041183 |  |
|  | MIR4644 |  |
|  | SPRR4 |  |
|  | C6orf7 |  |
|  | AGT |  |
|  | OTTHUMG00000162904 |  |
|  | PRSS48 |  |
|  | LOC101101776 |  |
|  | DCAF4L2 |  |
|  | LRRC18 |  |
|  | PRG1 |  |
|  | PLAG1 |  |
|  | OTTHUMG00000016088 |  |
|  | OR11H6 |  |
|  | MIR548T |  |
|  | OTTHUMG00000159113 |  |
|  | OR8D1 |  |
|  | TLE1P1 |  |
|  | OTTHUMG00000015778 |  |
|  | GAL3ST3 |  |
|  | OTTHUMG00000154940 |  |
|  | OTTHUMG00000161685 |  |
|  | LINC00626 |  |
|  | OTTHUMG00000155313 |  |
|  | SNORD116-11 |  |
|  | OTTHUMG00000019589 |  |
|  | OTTHUMG00000015106 |  |
|  | THRSP |  |
|  | HCCAT5 |  |
|  | OTTHUMG00000161136 |  |
|  | TCF23 |  |
|  | DEFB129 |  |
|  | FAM25A |  |
|  | LINC00866 |  |
|  | MIR711 |  |
|  | IGKV1-9 |  |
|  | OTTHUMG00000161767 |  |
|  | OTTHUMG00000163723 |  |
|  | LINC00575 |  |
|  | OTTHUMG00000153861 |  |
|  | KRBOX1-AS1 |  |
|  | OTTHUMG00000151377 |  |
|  | C1orf105 |  |
|  | OTTHUMG00000016476 |  |
|  | OTTHUMG00000164291 |  |
|  | MIR548H3 |  |
|  | PRAMEF8 |  |
|  | OTTHUMG00000031890 |  |
|  | OTTHUMG00000151260 |  |
|  | KRTAP10-11 |  |
|  | OTTHUMG00000090442 |  |
|  | KRTAP4-16P |  |
|  | PSG10P |  |
|  | OTTHUMG00000018017 |  |
|  | OTTHUMG00000161902 |  |
|  | OTTHUMG00000153758 |  |
|  | OR9A2 |  |
|  | OTTHUMG00000032688 |  |
|  | SNTN |  |
|  | LDLRAP1 |  |
|  | OTTHUMG00000020352 |  |
|  | ATG10-AS1 |  |
|  | OTTHUMG00000022540 |  |
|  | OTTHUMG00000022462 |  |
|  | TTTY8B |  |
|  | TTTY8 |  |
|  | OTTHUMG00000035458 |  |
|  | OTTHUMG00000157217 |  |
|  | OR10P1 |  |
|  | HSD3B1 |  |
|  | OTTHUMG00000152144 |  |
|  | OTTHUMG00000153366 |  |
|  | LINC00701 |  |
|  | MAPT-AS1 |  |
|  | PSG6 |  |
|  | LINC00410 |  |
|  | OTTHUMG00000162230 |  |
|  | OR13F1 |  |
|  | XAGE3 |  |
|  | OTTHUMG00000034319 |  |
|  | LOC100131107 |  |
|  | OTTHUMG00000163825 |  |
|  | OTTHUMG00000018255 |  |
|  | OTTHUMG00000154505 |  |
|  | OR5AP2 |  |
|  | MIR1255B2 |  |
|  | OR9A3P |  |
|  | ASCL3 |  |
|  | OTTHUMG00000015375 |  |
|  | MIR548Q |  |
|  | MIR516B1 |  |
|  | KRTAP4-7 |  |
|  | OTTHUMG00000156006 |  |
|  | OTTHUMG00000151376 |  |
|  | OTTHUMG00000086763 |  |
|  | OTTHUMG00000043021 |  |
|  | OTTHUMG00000003881 |  |
|  | OTTHUMG00000008128 |  |
|  | LINC00500 |  |
|  | C9orf38 |  |
|  | KRT6C |  |
|  | MIR3911 |  |
|  | OTTHUMG00000164860 |  |
|  | IGLJ4 |  |
|  | OTTHUMG00000063970 |  |
|  | MIR1231 |  |
|  | OTTHUMG00000162359 |  |
|  | OTTHUMG00000164715 |  |
|  | MIR185 |  |
|  | OTTHUMG00000153424 |  |
|  | LINC00487 |  |
|  | RAET1K |  |
|  | OTTHUMG00000154265 |  |
|  | MIR4252 |  |
|  | LOC100506912 |  |
|  | OTTHUMG00000162799 |  |
|  | RNA5SP180 |  |
|  | MIR1249 |  |
|  | SMCP |  |
|  | OTTHUMG00000020024 |  |
|  | OTTHUMG00000161749 |  |
|  | OTTHUMG00000058964 |  |
|  | OTTHUMG00000151346 |  |
|  | OTTHUMG00000086778 |  |
|  | OTTHUMG00000152375 |  |
|  | OTTHUMG00000152029 |  |
|  | OTTHUMG00000017405 |  |
|  | OTTHUMG00000021149 |  |
|  | PRAMEF17 |  |
|  | MIR193B |  |
|  | IGHV3-15 |  |
|  | OTTHUMG00000150675 |  |
|  | OTTHUMG00000015064 |  |
|  | OTTHUMG00000153006 |  |
|  | OTTHUMG00000078325 |  |
|  | CYP4A11 |  |
|  | OTTHUMG00000160893 |  |
|  | HILS1 |  |
|  | OTTHUMG00000156378 |  |
|  | OTTHUMG00000161900 |  |
|  | RNA5SP24 |  |
|  | LOC440704 |  |
|  | OTTHUMG00000164076 |  |
|  | OR10T2 |  |
|  | OR2G2 |  |
|  | OTTHUMG00000151705 |  |
|  | LCE3B |  |
|  | OTTHUMG00000163392 |  |
|  | HCG24 |  |
|  | OTTHUMG00000035655 |  |
|  | OTTHUMG00000018993 |  |
|  | OR8B4 |  |
|  | OTTHUMG00000002437 |  |
|  | MIR2278 |  |
|  | OTTHUMG00000161627 |  |
|  | KRTAP5-5 |  |
|  | OR10C1 |  |
|  | KRTAP2-2 |  |
|  | MIR4320 |  |
|  | OTTHUMG00000084883 |  |
|  | OTTHUMG00000148688 |  |
|  | OTTHUMG00000085278 |  |
|  | OTTHUMG00000148852 |  |
|  | OTTHUMG00000149131 |  |
|  | OTTHUMG00000149387 |  |
|  | OTTHUMG00000004760 |  |
|  | OTTHUMG00000085910 |  |
|  | OTTHUMG00000078079 |  |
|  | OR7A5 |  |
|  | LDHAL6CP |  |
|  | OTTHUMG00000032885 |  |
|  | SPRR3 |  |
|  | LOC100287704 |  |
|  | TMPRSS4-AS1 |  |
|  | LOC100129935 |  |
|  | IGKV1-37 |  |
|  | KIAA0196-AS1 |  |
|  | OTTHUMG00000163011 |  |
|  | MIR548A1 |  |
|  | OTTHUMG00000162167 |  |
|  | CXADRP2 |  |
|  | MAGEB6 |  |
|  | OR51V1 |  |
|  | OTTHUMG00000161522 |  |
|  | OTTHUMG00000158359 |  |
|  | OTTHUMG00000154037 |  |
|  | SPATA31D1 |  |
|  | MRGPRX1 |  |
|  | OTTHUMG00000159320 |  |
|  | OTTHUMG00000020108 |  |
|  | OTTHUMG00000164156 |  |
|  | OTTHUMG00000152019 |  |
|  | OTTHUMG00000067122 |  |
|  | MIR215 |  |
|  | IGLV7-46 |  |
|  | OTTHUMG00000156616 |  |
|  | TRIM49B |  |
|  | OTTHUMG00000133689 |  |
|  | OR4F6 |  |
|  | LOC729739 |  |
|  | IGKV3-7 |  |
|  | OTTHUMG00000153667 |  |
|  | PSG8 |  |
|  | MID2 |  |
|  | AHSG |  |
|  | PRAMEF6 |  |
|  | OTTHUMG00000153145 |  |
|  | OTTHUMG00000014592 |  |
|  | SLC22A17 |  |
|  | OR5H2 |  |
|  | OTTHUMG00000153709 |  |
|  | OTTHUMG00000020483 |  |
|  | OTTHUMG00000162028 |  |
|  | KGFLP2 |  |
|  | OTTHUMG00000017570 |  |
|  | PGLYRP3 |  |
|  | MIR4718 |  |
|  | LOC647264 |  |
|  | FAM170A |  |
|  | C11orf53 |  |
|  | OTTHUMG00000150956 |  |
|  | IQCF5-AS1 |  |
|  | OTTHUMG00000161054 |  |
|  | KRT6A |  |
|  | MIR3677 |  |
|  | LINC00362 |  |
|  | OR5AC2 |  |
|  | OTTHUMG00000013471 |  |
|  | NUPR1 |  |
|  | SPRR2D |  |
|  | MIR4730 |  |
|  | LOC100509195 |  |
|  | OTTHUMG00000161903 |  |
|  | OTTHUMG00000163185 |  |
|  | MIR4254 |  |
|  | HMHB1 |  |
|  | LOC100131496 |  |
|  | LOC150935 |  |
|  | PARD6G-AS1 |  |
|  | FAM183B |  |
|  | IGKV3D-15 |  |
|  | OTTHUMG00000184165 |  |
|  | KRTAP9-9 |  |
|  | OTTHUMG00000010575 |  |
|  | OTTHUMG00000018992 |  |
|  | OTTHUMG00000164977 |  |
|  | OTTHUMG00000163348 |  |
|  | OTTHUMG00000017359 |  |
|  | OTTHUMG00000013382 |  |
|  | OTTHUMG00000014257 |  |
|  | OTTHUMG00000153893 |  |
|  | LOC100506406 |  |
|  | GSTM3 |  |
|  | SOSTDC1 |  |
|  | OTTHUMG00000056663 |  |
|  | TRIM53AP |  |
|  | RFPL4A |  |
|  | OTTHUMG00000153435 |  |
|  | OTTHUMG00000158734 |  |
|  | MIR4640 |  |
|  | DEFB4A |  |
|  | CCL13 |  |
|  | SPATA31D4 |  |
|  | FLJ45256 |  |
|  | RNY1P8 |  |
|  | OR52N4 |  |
|  | LOC340113 |  |
|  | IGLV9-49 |  |
|  | OTTHUMG00000160041 |  |
|  | MIR764 |  |
|  | DEFB134 |  |
|  | FLG |  |
|  | OTTHUMG00000035542 |  |
|  | SLC17A2 |  |
|  | OTTHUMG00000030075 |  |
|  | LOC100505840 |  |
|  | HAVCR1P1 |  |
|  | OTTHUMG00000024194 |  |
|  | FREM2-AS1 |  |
|  | KRTAP13-3 |  |
|  | INSL4 |  |
|  | OR8G1 |  |
|  | OTTHUMG00000164487 |  |
|  | APOBEC3B-AS1 |  |
|  | LINC00901 |  |
|  | OTTHUMG00000157001 |  |
|  | KRTAP10-3 |  |
|  | OTTHUMG00000014290 |  |
|  | LOC100507222 |  |
|  | OR1J1 |  |
|  | GPR182 |  |
|  | OTTHUMG00000013093 |  |
|  | OTTHUMG00000067142 |  |
|  | NR0B2 |  |
|  | KRT39 |  |
|  | OTTHUMG00000161436 |  |
|  | OTTHUMG00000152555 |  |
|  | OR4A5 |  |
|  | OR6W1P |  |
|  | PEX5L-AS2 |  |
|  | PRB3 |  |
|  | OTTHUMG00000162302 |  |
|  | LOC283299 |  |
|  | OTTHUMG00000151384 |  |
|  | OR8H2 |  |
|  | OTTHUMG00000163530 |  |
|  | C5orf27 |  |
|  | F2RL2 |  |
|  | OTTHUMG00000164397 |  |
|  | OTTHUMG00000020726 |  |
|  | LOC400655 |  |
|  | CYP4A22 |  |
|  | OTTHUMG00000162941 |  |
|  | LINC00283 |  |
|  | RPL29P30 |  |
|  | OTTHUMG00000151809 |  |
|  | OTTHUMG00000164540 |  |
|  | LCE1B |  |
|  | C20orf78 |  |
|  | OTTHUMG00000157467 |  |
|  | MIR548M |  |
|  | OTTHUMG00000163900 |  |
|  | SPATA31C2 |  |
|  | OTTHUMG00000015218 |  |
|  | C11orf34 |  |
|  | OTTHUMG00000155022 |  |
|  | LINC00960 |  |
|  | OTTHUMG00000153281 |  |
|  | ZNF733P |  |
|  | ERVFRD-1 |  |
|  | MIR4267 |  |
|  | OTTHUMG00000160246 |  |
|  | KRTAP2-1 |  |
|  | KCNJ5 |  |
|  | OR5F1 |  |
|  | OTTHUMG00000144166 |  |
|  | OTTHUMG00000150678 |  |
|  | REG1A |  |
|  | OTTHUMG00000019207 |  |
|  | OTTHUMG00000160163 |  |
|  | OTTHUMG00000154113 |  |
|  | OTTHUMG00000034834 |  |
|  | OTTHUMG00000150168 |  |
|  | ENOX1-AS1 |  |
|  | OTTHUMG00000016843 |  |
|  | OTTHUMG00000163410 |  |
|  | MIR1976 |  |
|  | OTTHUMG00000041139 |  |
|  | MIR4289 |  |
|  | OTTHUMG00000163748 |  |
|  | OTTHUMG00000154238 |  |
|  | OTTHUMG00000078249 |  |
|  | RNA5SP293 |  |
|  | OTTHUMG00000158064 |  |
|  | RNA5SP26 |  |
|  | LINC00330 |  |
|  | OR1L4 |  |
|  | LOC158434 |  |
|  | OTTHUMG00000163927 |  |
|  | LOC392621 |  |
|  | OTTHUMG00000018174 |  |
|  | OTTHUMG00000066730 |  |
|  | OR10Z1 |  |
|  | OTTHUMG00000153044 |  |
|  | OTTHUMG00000017095 |  |
|  | PLCH1-AS1 |  |
|  | OTTHUMG00000162582 |  |
|  | DPPA3P2 |  |
|  | POTEG |  |
|  | OTTHUMG00000067507 |  |
|  | PLLP |  |
|  | OTTHUMG00000014989 |  |
|  | OR5T2 |  |
|  | OTTHUMG00000041309 |  |
|  | RNA5SP285 |  |
|  | OTTHUMG00000157083 |  |
|  | SNAR-I |  |
|  | OR5A2 |  |
|  | OR8B8 |  |
|  | OTTHUMG00000159111 |  |
|  | OTTHUMG00000153468 |  |
|  | OTTHUMG00000036381 |  |
|  | RNA5SP112 |  |
|  | OTTHUMG00000151756 |  |
|  | OR4C15 |  |
|  | OTTHUMG00000015769 |  |
|  | OTTHUMG00000159241 |  |
|  | MIR504 |  |
|  | OTTHUMG00000036214 |  |
|  | IGHV3-48 |  |
|  | OTTHUMG00000163935 |  |
|  | IGKV1D-12 |  |
|  | OR4K13 |  |
|  | OTTHUMG00000032097 |  |
|  | OTTHUMG00000153642 |  |
|  | OTTHUMG00000163446 |  |
|  | LOC100996601 |  |
|  | OTTHUMG00000058802 |  |
|  | OTTHUMG00000150172 |  |
|  | OTTHUMG00000152143 |  |
|  | MIR1293 |  |
|  | MIR382 |  |
|  | OR4N5 |  |
|  | OTTHUMG00000036266 |  |
|  | KRTAP3-3 |  |
|  | MIR1260B |  |
|  | SSX6 |  |
|  | IGKV3D-11 |  |
|  | LOC101060787 |  |
|  | LOC100507562 |  |
|  | FAM74A4 |  |
|  | OTTHUMG00000160708 |  |
|  | OTTHUMG00000150949 |  |
|  | OTTHUMG00000161085 |  |
|  | OTTHUMG00000162363 |  |
|  | OTTHUMG00000163770 |  |
|  | SUPT20HL2 |  |
|  | OTTHUMG00000157089 |  |
|  | OTTHUMG00000164390 |  |
|  | PACRG |  |
|  | MIR3663 |  |
|  | PRAMEF5 |  |
|  | LOC286189 |  |
|  | OTTHUMG00000163613 |  |
|  | MIR4493 |  |
|  | OTTHUMG00000151751 |  |
|  | LINC-ROR |  |
|  | SLC25A21-AS1 |  |
|  | OTTHUMG00000153999 |  |
|  | OTTHUMG00000144189 |  |
|  | HOOK1 |  |
|  | OTTHUMG00000161704 |  |
|  | LOC100506154 |  |
|  | LOC100507403 |  |
|  | OTTHUMG00000151403 |  |
|  | OTTHUMG00000008421 |  |
|  | TMIGD2 |  |
|  | OR10K1 |  |
|  | MUC21 |  |
|  | IGHJ1 |  |
|  | OTTHUMG00000148794 |  |
|  | OTTHUMG00000132955 |  |
|  | OTTHUMG00000149022 |  |
|  | OTTHUMG00000004916 |  |
|  | OTTHUMG00000133032 |  |
|  | MIR541 |  |
|  | RNA5SP247 |  |
|  | MIR1280 |  |
|  | OTTHUMG00000002076 |  |
|  | RNA5SP272 |  |
|  | LOC284344 |  |
|  | LEF1-AS1 |  |
|  | LY6G6C |  |
|  | WASIR2 |  |
|  | OTTHUMG00000132706 |  |
|  | OTTHUMG00000037760 |  |
|  | OTTHUMG00000162955 |  |
|  | DPCR1 |  |
|  | RNU1-24P |  |
|  | LINC00538 |  |
|  | RMDN2-AS1 |  |
|  | OTTHUMG00000150496 |  |
|  | OTTHUMG00000160129 |  |
|  | OR56A5 |  |
|  | DEFB125 |  |
|  | FLG2 |  |
|  | OTTHUMG00000001219 |  |
|  | FAM167B |  |
|  | HPVC1 |  |
|  | OTTHUMG00000037739 |  |
|  | NTM-IT3 |  |
|  | OTTHUMG00000160373 |  |
|  | OTTHUMG00000031921 |  |
|  | OTTHUMG00000161690 |  |
|  | LINGO2 |  |
|  | OTTHUMG00000020831 |  |
|  | LOC100507484 |  |
|  | KRTAP4-6 |  |
|  | CT49 |  |
|  | OTTHUMG00000150429 |  |
|  | NDP-AS1 |  |
|  | MIR1266 |  |
|  | OTTHUMG00000161241 |  |
|  | OTTHUMG00000163857 |  |
|  | SPRR1B |  |
|  | ANKRD55 |  |
|  | KRTAP20-3 |  |
|  | PWRN1 |  |
|  | WFDC12 |  |
|  | OTTHUMG00000155651 |  |
|  | OR7C2 |  |
|  | FAM47B |  |
|  | OTTHUMG00000164757 |  |
|  | OTTHUMG00000161791 |  |
|  | LOC100996902 |  |
|  | OTTHUMG00000037740 |  |
|  | OTTHUMG00000164181 |  |
|  | OTTHUMG00000021115 |  |
|  | OTTHUMG00000016460 |  |
|  | UBL4B |  |
|  | GAGE10 |  |
|  | OTTHUMG00000034949 |  |
|  | OTTHUMG00000164180 |  |
|  | OTTHUMG00000019140 |  |
|  | MC3R |  |
|  | SNORD63 |  |
|  | SPATA31A7 |  |
|  | OTTHUMG00000161137 |  |
|  | OTTHUMG00000159250 |  |
|  | OTTHUMG00000160886 |  |
|  | OTTHUMG00000154832 |  |
|  | LMO7 |  |
|  | LOC644669 |  |
|  | OTTHUMG00000164955 |  |
|  | DNASE2B |  |
|  | MT4 |  |
|  | OTTHUMG00000020654 |  |
|  | MIR4417 |  |
|  | OR7E12P |  |
|  | KRTAP6-1 |  |
|  | OTTHUMG00000155064 |  |
|  | OTTHUMG00000157195 |  |
|  | SNORD114-13 |  |
|  | OTTHUMG00000154509 |  |
|  | HIST1H2AH |  |
|  | OTTHUMG00000165033 |  |
|  | OTTHUMG00000020189 |  |
|  | LINC00400 |  |
|  | SSX9 |  |
|  | OTTHUMG00000018647 |  |
|  | OTTHUMG00000013262 |  |
|  | FLJ12825 |  |
|  | MIR544A |  |
|  | OR52R1 |  |
|  | APBA2 |  |
|  | LOC100505768 |  |
|  | LINC00302 |  |
|  | OTTHUMG00000161379 |  |
|  | OTTHUMG00000043647 |  |
|  | OTTHUMG00000151279 |  |
|  | IGLJ5 |  |
|  | LINC00681 |  |
|  | TRBV21OR9-2 |  |
|  | OTTHUMG00000156257 |  |
|  | EMCN-IT2 |  |
|  | OR6Y1 |  |
|  | SMLR1 |  |
|  | OTTHUMG00000022248 |  |
|  | MIR483 |  |
|  | OTTHUMG00000002711 |  |
|  | OR2L3 |  |
|  | ADTRP |  |
|  | ST6GAL2-IT1 |  |
|  | OTTHUMG00000162867 |  |
|  | OTTHUMG00000158362 |  |
|  | OTTHUMG00000164676 |  |
|  | IGBP1-AS2 |  |
|  | OTTHUMG00000160584 |  |
|  | OTTHUMG00000032152 |  |
|  | POTEC |  |
|  | OTTHUMG00000160821 |  |
|  | OTTHUMG00000078370 |  |
|  | KRTAP10-2 |  |
|  | IGHV3-66 |  |
|  | PFN1P2 |  |
|  | LINC00415 |  |
|  | IGFL1 |  |
|  | OTTHUMG00000162804 |  |
|  | HSD3BP4 |  |
|  | SNORD114-30 |  |
|  | OTTHUMG00000160992 |  |
|  | MIR4454 |  |
|  | OTTHUMG00000078483 |  |
|  | OTTHUMG00000160577 |  |
|  | OTTHUMG00000019602 |  |
|  | OTTHUMG00000155977 |  |
|  | OR2T1 |  |
|  | OTTHUMG00000018710 |  |
|  | OTTHUMG00000015224 |  |
|  | MIR1269A |  |
|  | SSX7 |  |
|  | OTTHUMG00000067120 |  |
|  | OTTHUMG00000058669 |  |
|  | OR2W5 |  |
|  | OR2A42 |  |
|  | LOC100507537 |  |
|  | OTTHUMG00000037755 |  |
|  | OTTHUMG00000152391 |  |
|  | RNA5SP111 |  |
|  | OTTHUMG00000020570 |  |
|  | OTTHUMG00000032494 |  |
|  | MIR500B |  |
|  | KRTAP5-1 |  |
|  | OTTHUMG00000066734 |  |
|  | OTTHUMG00000164033 |  |
|  | LOC100188947 |  |
|  | OTTHUMG00000011166 |  |
|  | OTTHUMG00000160440 |  |
|  | OTTHUMG00000010261 |  |
|  | OR13C8 |  |
|  | LOC283856 |  |
|  | OTTHUMG00000041435 |  |
|  | OTTHUMG00000165046 |  |
|  | OTTHUMG00000041136 |  |
|  | OR4D9 |  |
|  | OTTHUMG00000085915 |  |
|  | OTTHUMG00000085771 |  |
|  | OTTHUMG00000149115 |  |
|  | OTTHUMG00000149371 |  |
|  | OTTHUMG00000005007 |  |
|  | OTTHUMG00000085951 |  |
|  | FLJ45831 |  |
|  | OTTHUMG00000161747 |  |
|  | OTTHUMG00000041431 |  |
|  | OTTHUMG00000161939 |  |
|  | OTTHUMG00000151435 |  |
|  | OTTHUMG00000020254 |  |
|  | OTTHUMG00000156289 |  |
|  | OTTHUMG00000010851 |  |
|  | KRTAP21-3 |  |
|  | MIR4450 |  |
|  | LCE1D |  |
|  | OR51G2 |  |
|  | NSAP11 |  |
|  | OTTHUMG00000165064 |  |
|  | IGKV1-5 |  |
|  | OR5V1 |  |
|  | OTTHUMG00000132773 |  |
|  | OTTHUMG00000149304 |  |
|  | OTTHUMG00000149535 |  |
|  | OR4X1 |  |
|  | OTTHUMG00000155439 |  |
|  | MRGPRX4 |  |
|  | MIR548H2 |  |
|  | OTTHUMG00000003323 |  |
|  | MIR1299 |  |
|  | OTTHUMG00000009712 |  |
|  | OTTHUMG00000155014 |  |
|  | OR5M3 |  |
|  | OTTHUMG00000018618 |  |
|  | OR52E2 |  |
|  | OTTHUMG00000058154 |  |
|  | OTTHUMG00000161238 |  |
|  | OTTHUMG00000043773 |  |
|  | OTTHUMG00000158714 |  |
|  | OTTHUMG00000002044 |  |
|  | SATL1 |  |
|  | PCDHB18 |  |
|  | OTTHUMG00000022458 |  |
|  | DIRC1 |  |
|  | LINC00083 |  |
|  | TTLL7-IT1 |  |
|  | SPATA31C1 |  |
|  | IGKV3D-7 |  |
|  | OR5M8 |  |
|  | FMO1 |  |
|  | OTTHUMG00000162372 |  |
|  | OTTHUMG00000152567 |  |
|  | OTTHUMG00000150598 |  |
|  | OTTHUMG00000140353 |  |
|  | OTTHUMG00000155126 |  |
|  | USP17L4 |  |
|  | USP17L1P |  |
|  | OR11H12 |  |
|  | MIR1289-2 |  |
|  | OR2H2 |  |
|  | OTTHUMG00000163517 |  |
|  | MIR1226 |  |
|  | OTTHUMG00000016072 |  |
|  | OTTHUMG00000000392 |  |
|  | POTEB |  |
|  | SLC9A9-AS2 |  |
|  | OTTHUMG00000152670 |  |
|  | OTTHUMG00000153574 |  |
|  | LOC100996671 |  |
|  | DPPA3 |  |
|  | PSG1 |  |
|  | FLJ41200 |  |
|  | TPRG1-AS2 |  |
|  | OTTHUMG00000009845 |  |
|  | OR4C46 |  |
|  | MIR4736 |  |
|  | PRB1 |  |
|  | OTTHUMG00000153548 |  |
|  | OR2A14 |  |
|  | OTTHUMG00000015988 |  |
|  | MIR575 |  |
|  | OTTHUMG00000150317 |  |
|  | OTTHUMG00000164567 |  |
|  | RNU7-58P |  |
|  | SNORD116-12 |  |
|  | OTTHUMG00000148843 |  |
|  | OTTHUMG00000161708 |  |
|  | IGKV2D-26 |  |
|  | VTRNA2-1 |  |
|  | OTTHUMG00000032491 |  |
|  | NPBWR2 |  |
|  | OTTHUMG00000162672 |  |
|  | IGKV1-6 |  |
|  | FAM47A |  |
|  | C2orf78 |  |
|  | LPA |  |
|  | OTTHUMG00000014659 |  |
|  | MIR1260A |  |
|  | OTTHUMG00000155548 |  |
|  | OTTHUMG00000162005 |  |
|  | OTTHUMG00000013243 |  |
|  | MIR338 |  |
|  | MIR4739 |  |
|  | AFAP1-AS1 |  |
|  | OTTHUMG00000151012 |  |
|  | OTTHUMG00000017662 |  |
|  | IGKV2-28 |  |
|  | OTTHUMG00000041027 |  |
|  | OTTHUMG00000015621 |  |
|  | OR9Q2 |  |
|  | SNORD42A |  |
|  | WFDC6 |  |
|  | OTTHUMG00000163261 |  |
|  | KRTAP25-1 |  |
|  | LRRC10 |  |
|  | OTTHUMG00000018680 |  |
|  | OR11H4 |  |
|  | OTTHUMG00000020435 |  |
|  | KRTAP4-8 |  |
|  | OTTHUMG00000164759 |  |
|  | OR7E91P |  |
|  | KCNAB1-AS2 |  |
|  | OTTHUMG00000018185 |  |
|  | IGLJ7 |  |
|  | OTTHUMG00000161141 |  |
|  | MIR383 |  |
|  | OTTHUMG00000154730 |  |
|  | OTTHUMG00000164738 |  |
|  | OTTHUMG00000013297 |  |
|  | RNA5SP146 |  |
|  | OTTHUMG00000017159 |  |
|  | KRTAP10-8 |  |
|  | OTTHUMG00000019124 |  |
|  | LINC00520 |  |
|  | OTTHUMG00000046176 |  |
|  | OTTHUMG00000162919 |  |
|  | OTTHUMG00000163632 |  |
|  | OTTHUMG00000018194 |  |
|  | OTTHUMG00000067216 |  |
|  | OTTHUMG00000020320 |  |
|  | MIR4316 |  |
|  | IGLV3-12 |  |
|  | OR6P1 |  |
|  | OTTHUMG00000162995 |  |
|  | MYO16-AS1 |  |
|  | OTTHUMG00000032099 |  |
|  | OTTHUMG00000021757 |  |
|  | OTTHUMG00000151345 |  |
|  | OTTHUMG00000162327 |  |
|  | DEFB108B |  |
|  | PATE4 |  |
|  | OTTHUMG00000020222 |  |
|  | OTTHUMG00000020155 |  |
|  | OTTHUMG00000017545 |  |
|  | OR4F21 |  |
|  | OTTHUMG00000015698 |  |
|  | KRTAP4-2 |  |
|  | SPRR2C |  |
|  | MIR509-1 |  |
|  | OTTHUMG00000163670 |  |
|  | TRBV10-1 |  |
|  | OR5B3 |  |
|  | OTTHUMG00000162208 |  |
|  | OTTHUMG00000003078 |  |
|  | LINC00353 |  |
|  | ESRG |  |
|  | ACTR3BP6 |  |
|  | C11orf40 |  |
|  | OTTHUMG00000160767 |  |
|  | CARM1P1 |  |
|  | OTTHUMG00000039875 |  |
|  | KPRP |  |
|  | IGHV3-20 |  |
|  | OTTHUMG00000024161 |  |
|  | OTTHUMG00000162204 |  |
|  | DEFB104A |  |
|  | OR51L1 |  |
|  | OR4D10 |  |
|  | LOC100130451 |  |
|  | OTTHUMG00000020074 |  |
|  | OTTHUMG00000019590 |  |
|  | OR5W2 |  |
|  | OTTHUMG00000018267 |  |
|  | OTTHUMG00000013469 |  |
|  | OTTHUMG00000015982 |  |
|  | OTTHUMG00000015384 |  |
|  | LOC284632 |  |
|  | OTTHUMG00000162318 |  |
|  | OTTHUMG00000132850 |  |
|  | OTTHUMG00000160106 |  |
|  | OTTHUMG00000017704 |  |
|  | OTTHUMG00000161716 |  |
|  | OTTHUMG00000017569 |  |
|  | OTTHUMG00000152476 |  |
|  | OTTHUMG00000156011 |  |
|  | NPY6R |  |
|  | KCNIP4-IT1 |  |
|  | OTTHUMG00000132305 |  |
|  | MIR548AA2 |  |
|  | OTTHUMG00000040769 |  |
|  | OTTHUMG00000153947 |  |
|  | OTTHUMG00000059605 |  |
|  | OTTHUMG00000163302 |  |
|  | OTTHUMG00000035479 |  |
|  | OTTHUMG00000086771 |  |
|  | OTTHUMG00000151792 |  |
|  | RFPL4AL1 |  |
|  | MIR548A3 |  |
|  | CT45A6 |  |
|  | RNA5SP309 |  |
|  | MIR4669 |  |
|  | OTTHUMG00000017341 |  |
|  | OTTHUMG00000156934 |  |
|  | MIR574 |  |
|  | OTTHUMG00000012112 |  |
|  | SPRR2B |  |
|  | OTTHUMG00000015419 |  |
|  | MIR1271 |  |
|  | MIR3922 |  |
|  | OTTHUMG00000162165 |  |
|  | OTTHUMG00000018931 |  |
|  | OTTHUMG00000010651 |  |
|  | OTTHUMG00000164160 |  |
|  | PSG5 |  |
|  | OTTHUMG00000017628 |  |
|  | MAS1L |  |
|  | OTTHUMG00000159640 |  |
|  | ALDH1L1-AS1 |  |
|  | LINC00268 |  |
|  | PCAT4 |  |
|  | OTTHUMG00000162820 |  |
|  | C1orf140 |  |
|  | OTTHUMG00000161694 |  |
|  | OTTHUMG00000040900 |  |
|  | OR11H2 |  |
|  | TRNAI2 |  |
|  | BTBD9-AS1 |  |
|  | OTTHUMG00000014917 |  |
|  | OR2L5 |  |
|  | OTTHUMG00000074319 |  |
|  | DEFB116 |  |
|  | MIR3666 |  |
|  | MIR548O2 |  |
|  | KRTAP4-5 |  |
|  | OTTHUMG00000008374 |  |
|  | FAM170B-AS1 |  |
|  | OTTHUMG00000161561 |  |
|  | OTTHUMG00000001770 |  |
|  | OTTHUMG00000153650 |  |
|  | LOC100506827 |  |
|  | OTTHUMG00000153588 |  |
|  | IGKV2D-24 |  |
|  | OR2T27 |  |
|  | OTTHUMG00000153703 |  |
|  | SNORD116-10 |  |
|  | OTTHUMG00000155471 |  |
|  | IGHA1 |  |
|  | OTTHUMG00000041465 |  |
|  | OTTHUMG00000004228 |  |
|  | OTTHUMG00000017551 |  |
|  | PRSS3P3 |  |
|  | MAL |  |
|  | OTTHUMG00000163671 |  |
|  | OTTHUMG00000016750 |  |
|  | TAAR8 |  |
|  | OR2T2 |  |
|  | OTTHUMG00000005714 |  |
|  | CD27 |  |
|  | LRP6 |  |
|  | LOC728724 |  |
|  | SPRR1A |  |
|  | OR10H2 |  |
|  | IGKV2D-29 |  |
|  | LOC100126582 |  |
|  | RNASE3 |  |
|  | OTTHUMG00000161808 |  |
|  | OTTHUMG00000037424 |  |
|  | LCE1E |  |
|  | RNA5SP181 |  |
|  | IGKV2-24 |  |
|  | OTTHUMG00000003037 |  |
|  | OR51A2 |  |
|  | OTTHUMG00000163244 |  |
|  | OTTHUMG00000020437 |  |
|  | OR52A5 |  |
|  | OTTHUMG00000164558 |  |
|  | SPATA31A3 |  |
|  | OR5D14 |  |
|  | MIR4480 |  |
|  | LOC645359 |  |
|  | OTTHUMG00000016049 |  |
|  | MIR4446 |  |
|  | OTTHUMG00000037849 |  |
|  | MIR1256 |  |
|  | OTTHUMG00000015399 |  |
|  | OTTHUMG00000078699 |  |
|  | TAS2R39 |  |
|  | MIR4711 |  |
|  | OR2M5 |  |
|  | LOC650293 |  |
|  | TP53TG3D |  |
|  | OTTHUMG00000032468 |  |
|  | OTTHUMG00000022584 |  |
|  | OR52B6 |  |
|  | OTTHUMG00000014573 |  |
|  | LINC00393 |  |
|  | OTTHUMG00000018926 |  |
|  | OTTHUMG00000017146 |  |
|  | KRTAP29-1 |  |
|  | OTTHUMG00000158448 |  |
|  | OTTHUMG00000019703 |  |
|  | RASGRF2 |  |
|  | OTTHUMG00000015705 |  |
|  | AQP7P3 |  |
|  | SNORD116-25 |  |
|  | OTTHUMG00000017352 |  |
|  | OTTHUMG00000161116 |  |
|  | OTTHUMG00000153759 |  |
|  | ACTR3BP2 |  |
|  | LOC100506474 |  |
|  | OTTHUMG00000033267 |  |
|  | OTTHUMG00000074167 |  |
|  | OTTHUMG00000155562 |  |
|  | OTTHUMG00000133141 |  |
|  | OTTHUMG00000164545 |  |
|  | ATP13A5-AS1 |  |
|  | OTTHUMG00000158995 |  |
|  | OTTHUMG00000013475 |  |
|  | MIR4747 |  |
|  | FAM205A |  |
|  | NBEAP1 |  |
|  | TCEA3 |  |
|  | BACH2 |  |
|  | OTTHUMG00000016983 |  |
|  | OTTHUMG00000164897 |  |
|  | C21ORF116 |  |
|  | LOC392196 |  |
|  | OTTHUMG00000163174 |  |
|  | OTTHUMG00000021533 |  |
|  | OTTHUMG00000152140 |  |
|  | OR10G8 |  |
|  | OTTHUMG00000153374 |  |
|  | OTTHUMG00000163339 |  |
|  | OTTHUMG00000152863 |  |
|  | CT45A3 |  |
|  | OTTHUMG00000162457 |  |
|  | OTTHUMG00000164191 |  |
|  | CERS6-AS1 |  |
|  | MIR1301 |  |
|  | OR10K2 |  |
|  | OTTHUMG00000059072 |  |
|  | OTTHUMG00000016533 |  |
|  | OTTHUMG00000160299 |  |
|  | OTTHUMG00000160828 |  |
|  | LINC00517 |  |
|  | OTTHUMG00000161729 |  |
|  | OR2M7 |  |
|  | OTTHUMG00000162042 |  |
|  | OTTHUMG00000017544 |  |
|  | SSX2B |  |
|  | DEFB112 |  |
|  | OTTHUMG00000012411 |  |
|  | CT45A1 |  |
|  | OTTHUMG00000157151 |  |
|  | CSN3 |  |
|  | CT45A2 |  |
|  | OTTHUMG00000040386 |  |
|  | OTTHUMG00000164525 |  |
|  | DISC1-IT1 |  |
|  | OR4N2 |  |
|  | KRTAP1-3 |  |
|  | RNU7-88P |  |
|  | OR2AK2 |  |
|  | OTTHUMG00000152344 |  |
|  | RNA5SP98 |  |
|  | OTTHUMG00000162216 |  |
|  | OTTHUMG00000078245 |  |
|  | MIR548AN |  |
|  | AKR1B1P6 |  |
|  | OTTHUMG00000160990 |  |
|  | OTTHUMG00000162780 |  |
|  | OTTHUMG00000133661 |  |
|  | OTTHUMG00000055854 |  |
|  | LELP1 |  |
|  | OTTHUMG00000014881 |  |
|  | OTTHUMG00000011899 |  |
|  | LCE2C |  |
|  | OTTHUMG00000161909 |  |
|  | OTTHUMG00000161459 |  |
|  | MLLT10P1 |  |
|  | OTTHUMG00000034467 |  |
|  | RNA5SP321 |  |
|  | IGKV3-11 |  |
|  | OTTHUMG00000165024 |  |
|  | ABCC5-AS1 |  |
|  | OTTHUMG00000042242 |  |
|  | LINC00519 |  |
|  | IGKV2D-28 |  |
|  | OTTHUMG00000158839 |  |
|  | OTTHUMG00000161740 |  |
|  | OR2B6 |  |
|  | LCE3E |  |
|  | OTTHUMG00000151371 |  |
|  | PISRT1 |  |
|  | OTTHUMG00000132246 |  |
|  | OR51F2 |  |
|  | OR10H3 |  |
|  | CT45A4 |  |
|  | OR2T6 |  |
|  | OTTHUMG00000078329 |  |
|  | IFNA1 |  |
|  | OTTHUMG00000163612 |  |
|  | FER1L6-AS1 |  |
|  | BACH1-AS1 |  |
|  | OTTHUMG00000161769 |  |
|  | OTTHUMG00000059147 |  |
|  | OTTHUMG00000164841 |  |
|  | MIR488 |  |
|  | NMUR2 |  |
|  | LOC100507521 |  |
|  | OTTHUMG00000020444 |  |
|  | UCA1 |  |
|  | OR4D2 |  |
|  | OTTHUMG00000014802 |  |
|  | OTTHUMG00000074606 |  |
|  | MIR654 |  |
|  | OR10A2 |  |
|  | KRTAP20-1 |  |
|  | RPTN |  |
|  | HAO2-IT1 |  |
|  | KRTAP26-1 |  |
|  | OTTHUMG00000155405 |  |
|  | OR52M1 |  |
|  | OR10G2 |  |
|  | CHL1-AS1 |  |
|  | OTTHUMG00000154841 |  |
|  | OTTHUMG00000078332 |  |
|  | OTTHUMG00000164436 |  |
|  | OR5M1 |  |
|  | MIR138-1 |  |
|  | MIR802 |  |
|  | KRTAP4-3 |  |
|  | OTTHUMG00000022472 |  |
|  | CMA1 |  |
|  | PCA3 |  |
|  | MIR4273 |  |
|  | IGKV1D-16 |  |
|  | OTTHUMG00000161215 |  |
|  | OTTHUMG00000065530 |  |
|  | OTTHUMG00000162202 |  |
|  | KIRREL3-AS2 |  |
|  | OTTHUMG00000154972 |  |
|  | OTTHUMG00000155708 |  |
|  | OTTHUMG00000035230 |  |
|  | AK5 |  |
|  | OTTHUMG00000157320 |  |
|  | OTTHUMG00000013300 |  |
|  | OTTHUMG00000163343 |  |
|  | OTTHUMG00000017337 |  |
|  | OTTHUMG00000162882 |  |
|  | OTTHUMG00000164478 |  |
|  | LOC100133746 |  |
|  | OTTHUMG00000039868 |  |
|  | OR9I1 |  |
|  | OR51Q1 |  |
|  | LCE2A |  |
|  | OTTHUMG00000164831 |  |
|  | OR5AK4P |  |
|  | OR4C12 |  |
|  | LINC00525 |  |
|  | OTTHUMG00000154316 |  |
|  | RNA5SP471 |  |
|  | OTTHUMG00000058150 |  |
|  | OR2T8 |  |
|  | OTTHUMG00000014379 |  |
|  | OTTHUMG00000008156 |  |
|  | OR5B2 |  |
|  | OR2T35 |  |
|  | GPR119 |  |
|  | MIR4279 |  |
|  | OTTHUMG00000161198 |  |
|  | S100A7 |  |
|  | LOC340074 |  |
|  | MIR370 |  |
|  | OTTHUMG00000086813 |  |
|  | PHEX-AS1 |  |
|  | OTTHUMG00000015687 |  |
|  | OTTHUMG00000016092 |  |
|  | MIR216A |  |
|  | OR5D16 |  |
|  | OTTHUMG00000154784 |  |
|  | OR51H1P |  |
|  | OR4K15 |  |
|  | OTTHUMG00000163422 |  |
|  | OTTHUMG00000150222 |  |
|  | OR14J1 |  |
|  | P4HA2-AS1 |  |
|  | LOC286297 |  |
|  | OR1S2 |  |
|  | SCEL-AS1 |  |
|  | DDX18P6 |  |
|  | RNA5SP274 |  |
|  | OTTHUMG00000015558 |  |
|  | OR6C75 |  |
|  | RNU7-9P |  |
|  | OTTHUMG00000019015 |  |
|  | SNORD114-31 |  |
|  | OR7C1 |  |
|  | IGKV1D-39 |  |
|  | OR4Q3 |  |
|  | SNORD116-14 |  |
|  | EDAR |  |
|  | OR7E37P |  |
|  | PRO0611 |  |
|  | OTTHUMG00000162193 |  |
|  | OR52N5 |  |
|  | OTTHUMG00000000399 |  |
|  | OTTHUMG00000153603 |  |
|  | OTTHUMG00000160276 |  |
|  | FAM71C |  |
|  | OTTHUMG00000036134 |  |
|  | OTTHUMG00000008401 |  |
|  | OTTHUMG00000074544 |  |
|  | C13orf45 |  |
|  | OTTHUMG00000040701 |  |
|  | PTCSC3 |  |
|  | FABP3P2 |  |
|  | OTTHUMG00000151323 |  |
|  | OTTHUMG00000164452 |  |
|  | OTTHUMG00000010849 |  |
|  | RNU7-75P |  |
|  | TSHZ2 |  |
|  | LOC100996643 |  |
|  | OTTHUMG00000039775 |  |
|  | OTTHUMG00000161213 |  |
|  | OTTHUMG00000021088 |  |
|  | OR6K6 |  |
|  | OR7E2P |  |
|  | OTTHUMG00000160455 |  |
|  | ACTR3BP5 |  |
|  | TP53TG3B |  |
|  | OTTHUMG00000150009 |  |
|  | KRTAP4-4 |  |
|  | OR4C45 |  |
|  | OTTHUMG00000009567 |  |
|  | LINC00361 |  |
|  | OR2T34 |  |
|  | KRTAP4-12 |  |
|  | OR1D2 |  |
|  | LOC100133920 |  |
|  | OR2L8 |  |
|  | OTTHUMG00000158943 |  |
|  | MIR4531 |  |
|  | CACNA1C-AS4 |  |
|  | KRTAP9-8 |  |
|  | OR5D13 |  |
|  | OR10A4 |  |
|  | KRTAP9-4 |  |
|  | OR1L3 |  |
|  | OTTHUMG00000018806 |  |
|  | KU-MEL-3 |  |
|  | OTTHUMG00000037619 |  |
|  | SNORD116-2 |  |
|  | OTTHUMG00000016041 |  |
|  | OR2L2 |  |
|  | OTTHUMG00000001215 |  |
|  | OR1J4 |  |
|  | OR56A1 |  |
|  | OR6N2 |  |
|  | SCGB1D2 |  |
|  | OR10J3 |  |
|  | KRTAP15-1 |  |
|  | OTTHUMG00000018038 |  |
|  | OR14K1 |  |
|  | OTTHUMG00000066715 |  |
|  | OTTHUMG00000017177 |  |
|  | OTTHUMG00000163008 |  |
|  | IGKV1D-37 |  |
|  | PRR21 |  |
|  | IGKV5-2 |  |
|  | USP17L2 |  |
|  | OTTHUMG00000163984 |  |
|  | OR4Q2 |  |
|  | OR2T4 |  |
|  | OTTHUMG00000016436 |  |
|  | OR10Q1 |  |
|  | MIR3151 |  |
|  | OTTHUMG00000018077 |  |
|  | PATE3 |  |
|  | OTTHUMG00000016470 |  |
|  | OTTHUMG00000066368 |  |
|  | TESPA1 |  |
|  | RNA5SP223 |  |
|  | OTTHUMG00000015761 |  |
|  | OTTHUMG00000153626 |  |
|  | OTTHUMG00000163798 |  |
|  | SPRR2F |  |
|  | GTF2IP3 |  |
|  | MAGI1-AS1 |  |
|  | SNORD116-26 |  |
|  | OR10S1 |  |
|  | RNA5SP501 |  |
|  | OTTHUMG00000162183 |  |
|  | OTTHUMG00000015645 |  |
|  | LCE3D |  |
|  | FLJ46066 |  |
|  | OTTHUMG00000161802 |  |
|  | LINC00297 |  |
|  | OTTHUMG00000163826 |  |
|  | LOC153910 |  |
|  | KRTAP9-2 |  |
|  | JAKMIP2-AS1 |  |
|  | MIR4770 |  |
|  | OTTHUMG00000043432 |  |
|  | OTTHUMG00000162648 |  |
|  | LOC100507030 |  |
|  | OTTHUMG00000152744 |  |
|  | OTTHUMG00000150901 |  |
|  | PWRN2 |  |
|  | OTTHUMG00000156552 |  |
|  | OR8G2 |  |
|  | TAAR3 |  |
|  | OTTHUMG00000090613 |  |
|  | MIR222 |  |
|  | OTTHUMG00000017650 |  |
|  | FLJ16171 |  |
|  | TXK |  |
|  | MIR548A2 |  |
|  | OTTHUMG00000018119 |  |
|  | KERA |  |
|  | SNORD114-25 |  |
|  | OTTHUMG00000008097 |  |
|  | PPEF1-AS1 |  |
|  | EPYC |  |
|  | OTTHUMG00000162901 |  |
|  | OTTHUMG00000018659 |  |
|  | KRTAP9-1 |  |
|  | OTTHUMG00000008038 |  |
|  | OTTHUMG00000163830 |  |
|  | MIR708 |  |
|  | LOC100996920 |  |
|  | OR2A4 |  |
|  | MIR548W |  |
|  | EGFLAM-AS1 |  |
|  | KRTAP11-1 |  |
|  | SNORD116-29 |  |
|  | OTTHUMG00000160365 |  |
|  | MIR509-2 |  |
|  | OTTHUMG00000015798 |  |
|  | MIR1261 |  |
|  | OTTHUMG00000013474 |  |
|  | NHS-AS1 |  |
|  | LOC100509638 |  |
|  | OTTHUMG00000015256 |  |
|  | OR4F15 |  |
|  | OTTHUMG00000161792 |  |
|  | OTTHUMG00000161773 |  |
|  | OTTHUMG00000160243 |  |
|  | OTTHUMG00000009500 |  |
|  | PRR9 |  |
|  | OR5P2 |  |
|  | OR5A1 |  |
|  | OTTHUMG00000014787 |  |
|  | OTTHUMG00000008426 |  |
|  | LOC642929 |  |
|  | LOC100506053 |  |
|  | OTTHUMG00000163252 |  |
|  | LINC00434 |  |
|  | OTTHUMG00000019951 |  |
|  | KRT72 |  |
|  | LOC100508046 |  |
|  | OTTHUMG00000163654 |  |
|  | OTTHUMG00000086615 |  |
|  | OR51A4 |  |
|  | MIR4472-2 |  |
|  | LINC00700 |  |
|  | OTTHUMG00000163814 |  |
|  | LINC00440 |  |
|  | OR4A15 |  |
|  | OR51C1P |  |
|  | OR56B4 |  |
|  | OR6K2 |  |
|  | OTTHUMG00000058148 |  |
|  | MLIP-AS1 |  |
|  | OR1L8 |  |
|  | OR8H1 |  |
|  | OTTHUMG00000067237 |  |
|  | OTTHUMG00000151387 |  |
|  | CHRM3-AS2 |  |
|  | OTTHUMG00000086700 |  |
|  | OR14C36 |  |
|  | MIR548X |  |
|  | OTTHUMG00000161340 |  |
|  | MIR147A |  |
|  | OTTHUMG00000009087 |  |
|  | OTTHUMG00000154884 |  |
|  | IGKV1D-27 |  |
|  | MIR103B2 |  |
|  | MIR1911 |  |
|  | OR4M2 |  |
|  | OTTHUMG00000015768 |  |
|  | MIR548U |  |
|  | OTTHUMG00000018630 |  |
|  | OTTHUMG00000152596 |  |
|  | IGKV2D-30 |  |
|  | OTTHUMG00000151714 |  |
|  | OR4X2 |  |
|  | OTTHUMG00000013631 |  |
|  | OR1G1 |  |
|  | OR5R1 |  |
|  | VWC2L-IT1 |  |
|  | OR52N1 |  |
|  | OTTHUMG00000155735 |  |
|  | LOC643648 |  |
|  | OR6C3 |  |
|  | OTTHUMG00000162849 |  |
|  | OR9G1 |  |
|  | OR1C1 |  |
|  | OR4D11 |  |
|  | NELL2 |  |
|  | OR2T3 |  |
|  | OR51B6 |  |
|  | OTTHUMG00000150319 |  |
|  | OR2M3 |  |
|  | LINC00439 |  |
|  | OTTHUMG00000067240 |  |
|  | OR5D18 |  |
|  | OTTHUMG00000013378 |  |
|  | OR2M2 |  |
|  | LOC100996339 |  |
|  | OR5E1P |  |
|  | SCGB2A1 |  |
|  | MIR4461 |  |
|  | OR4F17 |  |
|  | IGHD2-15 |  |
|  | OTTHUMG00000032141 |  |
|  | OTTHUMG00000155472 |  |
|  | OTTHUMG00000020640 |  |
|  | OR2M4 |  |
|  | OTTHUMG00000157228 |  |
|  | OR11H1 |  |
|  | OR10A7 |  |
|  | OR5B17 |  |
|  | OTTHUMG00000018018 |  |
|  | MIR4500 |  |
|  | MIR548I2 |  |
|  | MIR548I1 |  |
|  | MIR4328 |  |
|  | OR10A3 |  |
|  | OR4C13 |  |
|  | OTTHUMG00000037874 |  |
|  | DEFB108P2 |  |
|  | ATP2B2-IT2 |  |
|  | OTTHUMG00000013632 |  |
|  | OTTHUMG00000041043 |  |
|  | OTTHUMG00000153411 |  |
|  | OR52J3 |  |
|  | MIR548I3 |  |
|  | OR7E24 |  |
|  | OTTHUMG00000013089 |  |
|  | MIR605 |  |
|  | OTTHUMG00000151816 |  |
|  | OR52E6 |  |
|  | OR4F2P |  |
|  | RNU6-65 |  |
|  | OTTHUMG00000085417 |  |
|  | OR1D4 |  |
|  | LINC00402 |  |
|  | PLCH1-AS2 |  |
|  | DSC1 |  |
|  | LRRN3 |  |
|  | OR4F4 |  |
|  | OR4F5 |  |
|  | RNA5SP222 |  |
|  | RNA5SP311 |  |
|  | RNA5SP314 |  |
|  | RNA5SP317 |  |
|  | OR5I1 |  |
|  | RNA5SP310 |  |
|  | RNA5SP316 |  |
|  | KRT73 |  |
|  | RNA5SP320 |  |
|  | OR1D5 |  |
|  | RNA5SP312 |  |
|  | RNA5SP315 |  |
|  | RNA5SP313 |  |
|  | OR9A1P |  |
|  | OTTHUMG00000037063 |  |
|  | OTTHUMG00000013272 |  |
|  | MIR216B |  |
|  | NOG |  |
|  | CCR7 |  |
| pDCs | IFI44L | up |
|  | IFIT1 |  |
|  | XAF1 |  |
|  | CMPK2 |  |
|  | IFI44 |  |
|  | IFI6 |  |
|  | USP18 |  |
|  | RSAD2 |  |
|  | HESX1 |  |
|  | OAS3 |  |
|  | EPSTI1 |  |
|  | DDX60 |  |
|  | SLFN5 |  |
|  | IFI27 |  |
|  | IFITM1 |  |
|  | SAMD9L |  |
|  | OAS2 |  |
|  | IFIT3 |  |
|  | HERC6 |  |
|  | IFITM3 |  |
|  | OAS1 |  |
|  | MX2 |  |
|  | MX1 |  |
|  | SIGLEC1 |  |
|  | TNFSF10 |  |
|  | IFI35 |  |
|  | PARP12 |  |
|  | ISG20 |  |
|  | PARP9 |  |
|  | PARP14 |  |
|  | DTX3L |  |
|  | TRIM69 |  |
|  | MSR1 |  |
|  | EIF2AK2 |  |
|  | CXCL10 |  |
|  | GMPR |  |
|  | FIG4 |  |
|  | C1QC |  |
|  | PLSCR1 |  |
|  | IFIH1 |  |
|  | DDX60L |  |
|  | STAP1 |  |
|  | C1QB |  |
|  | CKS2 |  |
|  | BTN3A3 |  |
|  | ISG15 |  |
|  | BST2 |  |
|  | C19orf66 |  |
|  | HERC5 |  |
|  | IFITM2 |  |
|  | CTSL |  |
|  | RP11-395B7.7 |  |
|  | CD38 |  |
|  | TOR1B |  |
|  | GBP1 |  |
|  | NT5C3A |  |
|  | SAMD9 |  |
|  | COBL |  |
|  | UBE2L6 |  |
|  | SLC23A3 |  |
|  | CCL2 |  |
|  | STAT1 |  |
|  | SP110 |  |
|  | TRANK1 |  |
|  | ALDH8A1 |  |
|  | PSMB9 |  |
|  | GIMAP7 |  |
|  | OASL |  |
|  | LAP3 |  |
|  | TDRD7 |  |
|  | SAG |  |
|  | SLFN12 |  |
|  | NMI |  |
|  | BTN3A2 |  |
|  | SHISA5 |  |
|  | LOC100507535 |  |
|  | TNFSF13B |  |
|  | IFI16 |  |
|  | S100A11 |  |
|  | SECTM1 |  |
|  | TNS1 |  |
|  | TRIM5 |  |
|  | SMAD1 |  |
|  | PHF11 |  |
|  | CHMP5 |  |
|  | RP4-781L3.1 |  |
|  | DCBLD1 |  |
|  | TMEM86A |  |
|  | IGSF6 |  |
|  | PFKP |  |
|  | JAK2 |  |
|  | BLVRA |  |
|  | ELMO1 |  |
|  | C17orf62 |  |
|  | LY6E |  |
|  | LGALS9 |  |
|  | MERTK |  |
|  | TUBBP5 |  |
|  | SPATS2L |  |
|  | GPD2 |  |
|  | SMC6 |  |
|  | HEG1 |  |
|  | TMCC3 |  |
|  | RP11-687F6.1 |  |
|  | CD180 |  |
|  | LOC283745 |  |
|  | C2orf43 |  |
|  | NR1H3 |  |
|  | AF090939 |  |
|  | CHST12 |  |
|  | HCG26 |  |
|  | DDX58 |  |
|  | C21orf58 |  |
|  | IL1RN |  |
|  | NAPA |  |
|  | HGH1 |  |
|  | NR2E1 |  |
|  | AGPAT4-IT1 |  |
|  | OK/SW-CL.58 |  |
|  | AGBL2 |  |
|  | ADCK1 |  |
|  | MR1 |  |
|  | NUF2 |  |
|  | C1QA |  |
|  | DNAJC17 |  |
|  | FYB |  |
|  | CCR7 |  |
|  | RIPK3 |  |
|  | GNS |  |
|  | PDIA4 |  |
|  | TNFAIP8L1 |  |
|  | LOC642757 |  |
|  | RTP4 |  |
|  | HLA-F |  |
|  | TBCE |  |
|  | ADAR |  |
|  | IPO11 |  |
|  | GPR15 |  |
|  | RP11-45M22.3 |  |
|  | TRIM14 |  |
|  | RNF213 |  |
|  | VIPAS39 |  |
|  | LINC00607 |  |
|  | TM9SF1 |  |
|  | SCD |  |
|  | BRCA2 |  |
|  | IRF7 |  |
|  | PTPN22 |  |
|  | CASP4 |  |
|  | SLC9A9 |  |
|  | AC007362.3 |  |
|  | RP3-497J21.1 |  |
|  | LOC101928865 |  |
|  | LRP5 |  |
|  | KIAA0040 |  |
|  | LOC101929460 |  |
|  | RNASE1 |  |
|  | TAGLN |  |
|  | MYD88 |  |
|  | RAB29 |  |
|  | PSEN2 |  |
|  | ACVRL1 |  |
|  | AAAS |  |
|  | C19orf24 |  |
|  | PSMB8 |  |
|  | FAM129C |  |
|  | NOL6 |  |
|  | TRIM25 |  |
|  | BBX |  |
|  | LINC01260 |  |
|  | COX5A |  |
|  | LOC283194 |  |
|  | CISH |  |
|  | BYSL |  |
|  | HIRIP3 |  |
|  | MRPS18A |  |
|  | IL12RB2 |  |
|  | ELP6 |  |
|  | TRIM22 |  |
|  | LOC401176 |  |
|  | MACC1 |  |
|  | ANGPTL2 |  |
|  | S100A6 |  |
|  | TYMP |  |
|  | CARS |  |
|  | TOR4A |  |
|  | ELK1 |  |
|  | PANK2 |  |
|  | IGSF11 |  |
|  | ANKIB1 |  |
|  | REC8 |  |
|  | MVP |  |
|  | ZNF784 |  |
|  | LOC284632 |  |
|  | TINF2 |  |
|  | ARHGAP22 |  |
|  | COG4 |  |
|  | MITF |  |
|  | ACAT2 |  |
|  | C6orf62 |  |
|  | LINC01432 |  |
|  | BPHL |  |
|  | AP1M1 |  |
|  | FOXF2 |  |
|  | KCNK9 |  |
|  | FCHSD2 |  |
|  | DNTT |  |
|  | F11R |  |
|  | CCL27 |  |
|  | DDX26B |  |
|  | FOXP3 |  |
|  | TMEM27 |  |
|  | WNT8A |  |
|  | RP11-471G13.5 |  |
|  | TNFRSF14 |  |
|  | LOC101927257 |  |
|  | TRAPPC5 |  |
|  | TXN |  |
|  | IQCF5-AS1 |  |
|  | SP100 |  |
|  | SP2 |  |
|  | CCT8L2 |  |
|  | KNTC1 |  |
|  | LOC283435 |  |
|  | CTC-425F1.4 |  |
|  | ZMAT5 |  |
|  | AKR1A1 |  |
|  | VPREB1 |  |
|  | SDC3 |  |
|  | GALNT8 |  |
|  | NOX3 |  |
|  | MYL4 |  |
|  | WFDC6 |  |
|  | PDE10A |  |
|  | KDELC1 |  |
|  | FZD9 |  |
|  | CALR |  |
|  | SPEF2 |  |
|  | DDAH1 |  |
|  | RPS6KL1 |  |
|  | MPDU1 |  |
|  | FERMT3 |  |
|  | DHX58 |  |
|  | THTPA |  |
|  | MYCL |  |
|  | TMEM234 |  |
|  | FARSA |  |
|  | APOL1 |  |
|  | FAM46D |  |
|  | TMEM37 |  |
|  | DENND5A |  |
|  | LINC00656 |  |
|  | LENEP |  |
|  | LOC284865 |  |
|  | SAPCD2 |  |
|  | NAPSB |  |
|  | IRX4 |  |
|  | KIF4A |  |
|  | BTN3A1 |  |
|  | TMEM206 |  |
|  | GRIA2 |  |
|  | TMEM110 |  |
|  | KRT72 |  |
|  | FLJ31306 | down |
|  | LSMEM1 |  |
|  | AP3S1 |  |
|  | PRDM10 |  |
|  | TTC29 |  |
|  | TYW1 |  |
|  | PIGC |  |
|  | CLINT1 |  |
|  | ABT1 |  |
|  | MED4 |  |
|  | CBX5 |  |
|  | NPSR1-AS1 |  |
|  | CAPRIN1 |  |
|  | C2orf49 |  |
|  | LOC101929114 |  |
|  | LSM14A |  |
|  | COPS3 |  |
|  | KRR1 |  |
|  | DEPDC1 |  |
|  | ADAM32 |  |
|  | WBP4 |  |
|  | TOMM20 |  |
|  | IST1 |  |
|  | MAP7D1 |  |
|  | TNFSF18 |  |
|  | TUBAL3 |  |
|  | WDR48 |  |
|  | KLF13 |  |
|  | ZFP91 |  |
|  | LOC100128281 |  |
|  | USP26 |  |
|  | SMAD5 |  |
|  | REPS2 |  |
|  | SELT |  |
|  | GPCPD1 |  |
|  | MPHOSPH10 |  |
|  | PAQR9 |  |
|  | THUMPD3 |  |
|  | MORF4L1 |  |
|  | SLC4A7 |  |
|  | CTD-2540F13.2 |  |
|  | PPP2CA |  |
|  | PIK3CG |  |
|  | DIDO1 |  |
|  | EIF1 |  |
|  | PRPF18 |  |
|  | PGRMC2 |  |
|  | PLEKHA1 |  |
|  | TBC1D25 |  |
|  | KIAA0232 |  |
|  | COMMD3 |  |
|  | ZBTB1 |  |
|  | WNT2B |  |
|  | LYPLAL1 |  |
|  | DYNC1LI2 |  |
|  | AC018755.17 |  |
|  | NMD3 |  |
|  | SLC2A11 |  |
|  | TOE1 |  |
|  | DNAJB8-AS1 |  |
|  | CAMTA2 |  |
|  | CHMP2B |  |
|  | ACTR5 |  |
|  | FAM161A |  |
|  | ATP11A |  |
|  | FAM122B |  |
|  | RB1CC1 |  |
|  | GAREML |  |
|  | ERAL1 |  |
|  | RP1-263J7.2 |  |
|  | SRP72 |  |
|  | RP11-73M18.8 |  |
|  | ABCC10 |  |
|  | PSD3 |  |
|  | FEM1B |  |
|  | AQP3 |  |
|  | EYA1 |  |
|  | C18orf21 |  |
|  | SIK1 |  |
|  | ZNF395 |  |
|  | LMBR1L |  |
|  | PSMD6 |  |
|  | RMND5A |  |
|  | BRD1 |  |
|  | CA1 |  |
|  | EIF1AX |  |
|  | CPSF6 |  |
|  | ACVR1B |  |
|  | TMEM216 |  |
|  | TAOK2 |  |
|  | NDNL2 |  |
|  | PET117 |  |
|  | RP11-471M2.3 |  |
|  | SLC30A1 |  |
|  | UBL3 |  |
|  | GUK1 |  |
|  | PLCG2 |  |
|  | PIGH |  |
|  | BRAP |  |
|  | DLL1 |  |
|  | LUZP1 |  |
|  | SNAI1 |  |
|  | LOC100129973 |  |
|  | H2AFJ |  |
|  | STARD5 |  |
|  | FBXL5 |  |
|  | CEP85L |  |
|  | CTPS1 |  |
|  | SIAH1 |  |
|  | CECR3 |  |
|  | NAE1 |  |
|  | TLR6 |  |
|  | ZNF292 |  |
|  | DNTTIP2 |  |
|  | CYP2U1 |  |
|  | 8-Mar |  |
|  | MRPS22 |  |
|  | LINC00667 |  |
|  | USP7 |  |
|  | ZNF227 |  |
|  | CYSLTR1 |  |
|  | CD44 |  |
|  | YY1AP1 |  |
|  | DDX20 |  |
|  | MST4 |  |
|  | SLC25A36 |  |
|  | COIL |  |
|  | LINC01206 |  |
|  | SENP2 |  |
|  | PRKRIR |  |
|  | CCDC93 |  |
|  | RFK |  |
|  | MCMDC2 |  |
|  | PLEKHF2 |  |
|  | ZDHHC20 |  |
|  | RC3H1 |  |
|  | CLK1 |  |
|  | RBM27 |  |
|  | DCBLD2 |  |
|  | NLGN3 |  |
|  | DSTN |  |
|  | PRDM2 |  |
|  | ZNF74 |  |
|  | USP16 |  |
|  | C8orf58 |  |
|  | ABAT |  |
|  | DANCR |  |
|  | MEPCE |  |
|  | ATG14 |  |
|  | POLG2 |  |
|  | CCDC147 |  |
|  | DCTN4 |  |
|  | TTLL11 |  |
|  | HHEX |  |
|  | ZXDB |  |
|  | ANKS6 |  |
|  | ZNF417 |  |
|  | DUSP11 |  |
|  | RP9 |  |
|  | PRPF4B |  |
|  | YPEL3 |  |
|  | MPP6 |  |
|  | HIST1H1T |  |
|  | ZNF354B |  |
|  | ING1 |  |
|  | CD19 |  |
|  | HIST1H4J |  |
|  | RNF168 |  |
|  | ZHX1 |  |
|  | ZC3H15 |  |
|  | PRKRA |  |
|  | NFYB |  |
|  | TLE4 |  |
|  | RCOR1 |  |
|  | MAP4K3 |  |
|  | POLG |  |
|  | ATMIN |  |
|  | KIDINS220 |  |
|  | ENTPD4 |  |
|  | SCAF8 |  |
|  | RP11-473I1.9 |  |
|  | APBA1 |  |
|  | SFXN1 |  |
|  | SYAP1 |  |
|  | ARNTL |  |
|  | FUT11 |  |
|  | YTHDF3 |  |
|  | EI24 |  |
|  | TMEM17 |  |
|  | CDK2AP1 |  |
|  | LOC100507634 |  |
|  | STIM2 |  |
|  | NANP |  |
|  | C8orf44 |  |
|  | HIST1H4L |  |
|  | POLE2 |  |
|  | COL10A1 |  |
|  | KIAA1432 |  |
|  | LAMA5 |  |
|  | ADCY3 |  |
|  | R3HCC1L |  |
|  | CEBPZOS |  |
|  | SPATA7 |  |
|  | DONSON |  |
|  | ING2 |  |
|  | SENP5 |  |
|  | CYB5D1 |  |
|  | RP3-525N10.2 |  |
|  | KIF11 |  |
|  | ZNF484 |  |
|  | LANCL2 |  |
|  | RFWD3 |  |
|  | LRRC8B |  |
|  | ABHD14B |  |
|  | PNPLA8 |  |
|  | MPC1 |  |
|  | TJP2 |  |
|  | DDX59 |  |
|  | RAE1 |  |
|  | ITGAE |  |
|  | ICOS |  |
|  | HIST1H3C |  |
|  | RNF139 |  |
|  | HIST1H2BH |  |
|  | ZBTB11 |  |
|  | TGFBRAP1 |  |
|  | FYTTD1 |  |
|  | JOSD1 |  |
|  | TBPL1 |  |
|  | UBE2G1 |  |
|  | SFT2D3 |  |
|  | B3GALTL |  |
|  | SGK494 |  |
|  | UBE2E3 |  |
|  | RPF1 |  |
|  | RP5-1074L1.4 |  |
|  | MTURN |  |
|  | KAT6B |  |
|  | FAM126B |  |
|  | PLXDC1 |  |
|  | AMFR |  |
|  | NFYA |  |
|  | LOC102723918 |  |
|  | PDZK1 |  |
|  | BEND3 |  |
|  | EIF2AK3 |  |
|  | EPB42 |  |
|  | LOXL2 |  |
|  | METAP2 |  |
|  | MTPAP |  |
|  | NR1D2 |  |
|  | EIF5A2 |  |
|  | C1orf52 |  |
|  | GNMT |  |
|  | LOC101927211 |  |
|  | CGRRF1 |  |
|  | SEPT7P2 |  |
|  | MIS12 |  |
|  | PEX2 |  |
|  | USP45 |  |
|  | RCOR3 |  |
|  | RAB11FIP1 |  |
|  | CHST10 |  |
|  | ZC3H12C |  |
|  | GDE1 |  |
|  | FAR1 |  |
|  | SELP |  |
|  | PPP1R17 |  |
|  | TGFBR1 |  |
|  | N4BP3 |  |
|  | ZNF814 |  |
|  | TFDP2 |  |
|  | SUN1 |  |
|  | TRIM59 |  |
|  | FAM217A |  |
|  | TADA1 |  |
|  | CMTM4 |  |
|  | LOC100505716 |  |
|  | CPD |  |
|  | ZNF776 |  |
|  | GJA1 |  |
|  | BMF |  |
|  | CAAP1 |  |
|  | NFE2L3 |  |
|  | H2AFV |  |
|  | ZNF667 |  |
|  | TPM3 |  |
|  | PTAR1 |  |
|  | MAD2L1BP |  |
|  | NECAP1 |  |
|  | DCAF16 |  |
|  | ZNF329 |  |
|  | XK |  |
|  | RASGRP1 |  |
|  | RP11-421E14.2 |  |
|  | FAM101B |  |
|  | DNAJC1 |  |
|  | ARMC8 |  |
|  | ZNF326 |  |
|  | ZNF677 |  |
|  | COG3 |  |
|  | UIMC1 |  |
|  | CPQ |  |
|  | IFT57 |  |
|  | BCOR |  |
|  | SLC35D1 |  |
|  | CCNY |  |
|  | PIGA |  |
|  | GPR83 |  |
|  | LINC00565 |  |
|  | IFRD1 |  |
|  | AC139100.3 |  |
|  | RP11-650K20.3 |  |
|  | HSF2 |  |
|  | RBM17 |  |
|  | RP1-265C24.8 |  |
|  | NOL11 |  |
|  | UGT8 |  |
|  | UBXN7 |  |
|  | RAB33B |  |
|  | TOB1 |  |
|  | BAIAP2L1 |  |
|  | TMX4 |  |
|  | PLK4 |  |
|  | ARL4A |  |
|  | MYNN |  |
|  | HMGB3 |  |
|  | NXT1 |  |
|  | GNG2 |  |
|  | C12orf29 |  |
|  | HIST1H4E |  |
|  | KIAA0753 |  |
|  | MTR |  |
|  | PHF1 |  |
|  | CA8 |  |
|  | RIMS3 |  |
|  | NUBPL |  |
|  | CFL2 |  |
|  | LOC101928371 |  |
|  | KAT7 |  |
|  | CTNNB1 |  |
|  | CDS2 |  |
|  | CD109 |  |
|  | PRNP |  |
|  | BC041363 |  |
|  | BUB1B |  |
|  | EFCAB7 |  |
|  | CENPV |  |
|  | TGFBR3 |  |
|  | SMAD7 |  |
|  | ECI1 |  |
|  | DCHS1 |  |
|  | LINC00997 |  |
|  | FCRL3 |  |
|  | GPRASP1 |  |
|  | COCH |  |
|  | TCF7L1 |  |
|  | IQCC |  |
|  | U2SURP |  |
|  | MEX3C |  |
|  | THUMPD2 |  |
|  | CLMN |  |
|  | SH3RF3 |  |
|  | FBXL3 |  |
|  | SLITRK5 |  |
|  | ING3 |  |
|  | GLB1L |  |
|  | MSL2 |  |
|  | ZNF836 |  |
|  | DDX52 |  |
|  | ISG20L2 |  |
|  | TOR1AIP2 |  |
|  | MXI1 |  |
|  | HM13 |  |
|  | CHPT1 |  |
|  | HSPC081 |  |
|  | RP11-348N5.7 |  |
|  | LINC00969 |  |
|  | SLC19A2 |  |
|  | EPB41L1 |  |
|  | IRS2 |  |
|  | RP11-158G18.1 |  |
|  | CENPC |  |
|  | C16orf87 |  |
|  | UBXN2A |  |
|  | C12orf61 |  |
|  | GAFA3 |  |
|  | NAP1L3 |  |
|  | ITIH2 |  |
|  | EXTL2 |  |
|  | LOC153577 |  |
|  | BVES |  |
|  | FAM46C |  |
|  | RSBN1 |  |
|  | SLC18A2 |  |
|  | NAA16 |  |
|  | RP5-1065J22.8 |  |
|  | KLHL20 |  |
|  | TTC25 |  |
|  | BCL2L11 |  |
|  | CHEK1 |  |
|  | SERAC1 |  |
|  | ZNF711 |  |
|  | GADD45A |  |
|  | ZNF432 |  |
| PBMC | IFI44L | up |
|  | RSAD2 |  |
|  | HESX1 |  |
|  | IFIT1 |  |
|  | OTOF |  |
|  | IFIT3 |  |
|  | C1orf173 |  |
|  | ISG15 |  |
|  | CMPK2 |  |
|  | IFI44 |  |
|  | HES4 |  |
|  | HERC5 |  |
|  | STATH |  |
|  | IFIT2 |  |
|  | FAM70A |  |
|  | ID2B |  |
|  | DXS542 |  |
|  | LY6E |  |
|  | LOC255167 |  |
|  | FLJ10489 |  |
|  | LAMP3 |  |
|  | IFI6 |  |
|  | PKN3 |  |
|  | LOC340239 |  |
|  | HCG4 |  |
|  | KLKB1 |  |
|  | DKK1 |  |
|  | C9orf152 |  |
|  | ATP1B4 |  |
|  | PRG4 |  |
|  | HERC6 |  |
|  | MATN3 |  |
|  | FMO9P |  |
|  | LCE2B |  |
|  | SLC7A13 |  |
|  | HTR1F |  |
|  | CXCL10 |  |
|  | LOC391020 |  |
|  | LRAT |  |
|  | SCTR |  |
|  | LOC100128922 |  |
|  | CXCL11 |  |
|  | IGFBP1 |  |
|  | TAS2R38 |  |
|  | EFHD1 |  |
|  | PDGFRB |  |
|  | MEIG1 |  |
|  | LOC157740 |  |
|  | SCN1A |  |
|  | GJA10 |  |
|  | LOC100128590 |  |
|  | KCNK9 |  |
|  | ZCCHC12 |  |
|  | RAI14 |  |
|  | LOC151878 |  |
|  | KCNF1 |  |
|  | LOC644135 |  |
|  | IP6K3 |  |
|  | MAS1L |  |
|  | LOC100128164 |  |
|  | EFNA1 |  |
|  | FGF12 |  |
|  | ENSG00000204706 |  |
|  | TIE1 |  |
|  | EIF2AK2 |  |
|  | AMPD1 |  |
|  | SYDE2 |  |
|  | C6orf142 |  |
|  | CPN2 |  |
|  | DDX60 |  |
|  | HYAL4 |  |
|  | C10orf68 |  |
|  | IL9 |  |
|  | RAET1E |  |
|  | FLJ11235 |  |
|  | C10orf108 |  |
|  | CXorf64 |  |
|  | MTBP |  |
|  | TTTY14 |  |
|  | LOC644090 |  |
|  | LOC340090 |  |
|  | C8orf56 |  |
|  | ADCY10 |  |
|  | CENPE |  |
|  | LOC284561 |  |
|  | RASGEF1B |  |
|  | MEOX2 |  |
|  | LOC26010 |  |
|  | SLC12A8 |  |
|  | THBS4 |  |
|  | TLR3 |  |
|  | IFIT5 |  |
|  | SAMD9L |  |
|  | SDC1 |  |
|  | SIX3 |  |
|  | AGRN |  |
|  | TMEM72 |  |
|  | PHKA1 |  |
|  | PPFIA4 |  |
|  | PHOX2B |  |
|  | MELK |  |
|  | BAALC |  |
|  | RXRG |  |
|  | SCIN |  |
|  | NPBWR1 |  |
|  | C8orf54 |  |
|  | POU3F3 |  |
|  | DUSP9 |  |
|  | AHDC1 |  |
|  | MAB21L2 |  |
|  | FAM83H |  |
|  | PARP9 |  |
|  | SRPK3 |  |
|  | S100A16 |  |
|  | SAMD9 |  |
|  | MYL3 |  |
|  | RTP4 |  |
|  | TTLL2 |  |
|  | HTR5A |  |
|  | FLJ38379 |  |
|  | EPHB2 |  |
|  | SNTG1 |  |
|  | IFIH1 |  |
|  | LOC100132618 |  |
|  | MYO6 |  |
|  | STMN2 |  |
|  | SRPX2 |  |
|  | DENND1B |  |
|  | DDX58 |  |
|  | PLSCR1 |  |
|  | LOC285370 |  |
|  | PLXNA3 |  |
|  | NFKBIL2 |  |
|  | RGS12 |  |
|  | ZSCAN12L1 |  |
|  | LOC642031 |  |
|  | PCDHB14 |  |
|  | PPM1L |  |
|  | CDX1 |  |
|  | FGF8 |  |
|  | ZNF713 |  |
|  | GLI2 |  |
|  | KIF6 |  |
|  | DDX60L |  |
|  | OR1Q1 |  |
|  | DES |  |
|  | PARP12 |  |
|  | CXorf48 |  |
|  | CXorf57 |  |
|  | TTC21A |  |
|  | LOC727944 |  |
|  | CGN |  |
|  | RGL1 |  |
|  | PCTK3 |  |
|  | EPHA7 |  |
|  | LAP3 |  |
|  | CAPSL |  |
|  | PNPT1 |  |
|  | IMPG1 |  |
|  | SDK1 |  |
|  | PLEKHG5 |  |
|  | CFC1 |  |
|  | KIAA1958 |  |
|  | THPO |  |
|  | MFSD7 |  |
|  | SLC38A5 |  |
|  | SCN11A |  |
|  | C1orf201 |  |
|  | ENSG00000217005 |  |
|  | COX6A1P |  |
|  | DHCR24 |  |
|  | CCNB3 |  |
|  | CD38 |  |
|  | PTX3 |  |
|  | SPATA16 |  |
|  | AKR1C3 |  |
|  | FKBP14 |  |
|  | ZNF251 |  |
|  | GPR82 |  |
|  | LOC100128003 |  |
|  | PPM1K |  |
|  | HR |  |
|  | PRF1 |  |
|  | PGAP1 |  |
|  | DTX3L |  |
|  | EHHADH |  |
|  | SPON2 |  |
|  | FGF18 |  |
|  | TOR1B |  |
|  | SYN2 |  |
|  | SLC45A2 |  |
|  | CISH |  |
|  | ZNF684 |  |
|  | NEXN |  |
|  | CSRNP1 |  |
|  | REEP2 |  |
|  | CHMP5 |  |
|  | GFI1 |  |
|  | TNFSF10 |  |
|  | PI4K2B |  |
|  | ENPP5 |  |
|  | MARCKS |  |
|  | RXFP3 |  |
|  | MOV10 |  |
|  | SMPDL3B |  |
|  | VIT |  |
|  | TDRD7 |  |
|  | CCR1 |  |
|  | GPIHBP1 |  |
|  | BLVRA |  |
|  | CTSL1 |  |
|  | IFI16 |  |
|  | ZBTB37 |  |
|  | MASTL |  |
|  | FEV |  |
|  | TXK |  |
|  | MERTK |  |
|  | SLC22A14 |  |
|  | FANCL |  |
|  | CLDN23 |  |
|  | SH2D2A |  |
|  | STAP1 |  |
|  | SP110 |  |
|  | IFT74 |  |
|  | FAM46A |  |
|  | GPR146 |  |
|  | CCDC136 |  |
|  | FASLG |  |
|  | FTSJD2 |  |
|  | NMI |  |
|  | PLAC8 |  |
|  | SP100 |  |
|  | ABCA11P |  |
|  | DTX2 |  |
|  | TUBE1 |  |
|  | PM20D2 |  |
|  | CYP51A1 |  |
|  | TRAT1 |  |
|  | HEG1 |  |
|  | ACTL6B |  |
|  | ZFP37 |  |
|  | STAG3 |  |
|  | CHST10 |  |
|  | ARRDC3 |  |
|  | CUZD1 |  |
|  | ODF2L |  |
|  | CHST12 |  |
|  | ADAR |  |
|  | AMIGO1 |  |
|  | C2orf34 |  |
|  | ACSL6 |  |
|  | C7orf46 |  |
|  | TGFBR3 |  |
|  | ID2 |  |
|  | ELOVL6 |  |
|  | C4orf29 |  |
|  | ZNRF2 |  |
|  | CPEB3 |  |
|  | SHISA5 |  |
|  | SLCO4C1 |  |
|  | OTUD6B |  |
|  | TREX1 |  |
|  | PNLDC1 |  |
|  | TAF1B |  |
|  | LOC150759 |  |
|  | PTPN4 |  |
|  | CENPC1 |  |
|  | USP45 |  |
|  | NME7 |  |
|  | GALE |  |
|  | PYHIN1 |  |
|  | AP3M2 |  |
|  | ZNF273 |  |
|  | DENND4C |  |
|  | PARP10 |  |
|  | MSH3 |  |
|  | CD247 |  |
|  | TSEPA |  |
|  | IGFBP7 |  |
|  | MRPL19 |  |
|  | ARTN |  |
|  | ENPP4 |  |
|  | BLZF1 |  |
|  | SQLE |  |
|  | YIPF4 |  |
|  | LGI3 |  |
|  | USP24 |  |
|  | C1QTNF3 |  |
|  | LBA1 |  |
|  | MYBL1 |  |
|  | C2orf47 |  |
|  | ITK |  |
|  | FAM169A |  |
|  | MAGEE1 |  |
|  | C1GALT1 |  |
|  | TBCCD1 |  |
|  | TMEM170B | down |
|  | DNAJC8 |  |
|  | PABPC4 |  |
|  | CETN2 |  |
|  | CSF2RA |  |
|  | EAF2 |  |
|  | CLCN5 |  |
|  | ENTPD1 |  |
|  | TSC22D2 |  |
|  | PANK3 |  |
|  | COQ2 |  |
|  | FRS3 |  |
|  | FAM135A |  |
|  | RNF11 |  |
|  | PNPLA4 |  |
|  | BLK |  |
|  | TKT |  |
|  | FAM63A |  |
|  | UBE4B |  |
|  | RAB4A |  |
|  | ARHGAP18 |  |
|  | C9orf119 |  |
|  | RBP7 |  |
|  | CDC14B |  |
|  | FAM128B |  |
|  | SETD7 |  |
|  | ARHGAP24 |  |
|  | SLC39A4 |  |
|  | SUMF1 |  |
|  | C9orf16 |  |
|  | SMARCD3 |  |
|  | ZBED3 |  |
|  | CSF3R |  |
|  | F13A1 |  |
|  | hCG_1806964 |  |
|  | CLIP2 |  |
|  | SGMS2 |  |
|  | SEMA3C |  |
|  | IL1R1 |  |
|  | S100Z |  |
|  | FKBP1B |  |
|  | CRTAP |  |
|  | CYB5R1 |  |
|  | LOC541471 |  |
|  | PTCRA |  |
|  | MPP1 |  |
|  | PIK3CB |  |
|  | C7orf53 |  |
|  | CCDC90A |  |
|  | GSN |  |
|  | ST7 |  |
|  | IGF2R |  |
|  | CLCN3 |  |
|  | ODC1 |  |
|  | MBOAT2 |  |
|  | BICD2 |  |
|  | DNM3 |  |
|  | WNT10A |  |
|  | CPVL |  |
|  | C5 |  |
|  | PLA2G2D |  |
|  | PID1 |  |
|  | IL13RA1 |  |
|  | KLHL29 |  |
|  | TBC1D12 |  |
|  | MAPK10 |  |
|  | PLA2G12A |  |
|  | CCR2 |  |
|  | MST150 |  |
|  | TMEM40 |  |
|  | CLIC4 |  |
|  | EPB49 |  |
|  | TUBB2C |  |
|  | PDE5A |  |
|  | CTNNBIP1 |  |
|  | ST6GALNAC3 |  |
|  | LOC730139 |  |
|  | NMNAT3 |  |
|  | PDLIM1 |  |
|  | ARHGAP6 |  |
|  | TGFA |  |
|  | PTAFR |  |
|  | TNFSF4 |  |
|  | FMO5 |  |
|  | SCFD2 |  |
|  | C1orf198 |  |
|  | PBX1 |  |
|  | TMEM163 |  |
|  | CDA |  |
|  | LMNA |  |
|  | VNN2 |  |
|  | PGRMC1 |  |
|  | PGD |  |
|  | QARS |  |
|  | TBC1D9 |  |
|  | DTWD2 |  |
|  | TAGLN2 |  |
|  | RUNX2 |  |
|  | ENSG00000185389 |  |
|  | GNG11 |  |
|  | BASP1 |  |
|  | CLDN18 |  |
|  | CYBRD1 |  |
|  | ZNF185 |  |
|  | KIAA0114 |  |
|  | HADHA |  |
|  | LOC153469 |  |
|  | COL6A3 |  |
|  | FGF13 |  |
|  | NAT8B |  |
|  | SH3BP4 |  |
|  | FBXO24 |  |
|  | KIAA1211 |  |
|  | CCNJL |  |
|  | GPR177 |  |
|  | LEPR |  |
|  | HSPC159 |  |
|  | EHD3 |  |
|  | ITGB5 |  |
|  | GUCY1B3 |  |
|  | TSPAN33 |  |
|  | C7orf41 |  |
|  | PRTFDC1 |  |
|  | SLC35D3 |  |
|  | C5orf4 |  |
|  | HCG11 |  |
|  | SDPR |  |
|  | AGBL5 |  |
|  | SELP |  |
|  | FAM167A |  |
|  | PRKAR2B |  |
|  | SPR |  |
|  | REPS2 |  |
|  | HECW2 |  |
|  | PADI4 |  |
|  | CALD1 |  |
|  | SOD2 |  |
|  | LEFTY1 |  |
|  | ENKUR |  |
|  | GPX3 |  |
|  | TFPI |  |
|  | SCRG1 |  |
|  | SLC46A2 |  |
|  | C8orf42 |  |
|  | ZBTB8A |  |
|  | ASAP2 |  |
|  | FSTL1 |  |
|  | PTGS1 |  |
|  | C3orf54 |  |
|  | ALDH1A1 |  |
|  | FILIP1L |  |
|  | AQP1 |  |
|  | GFI1B |  |
|  | SPARC |  |
|  | EPB41L4B |  |
|  | ELOVL7 |  |
|  | ABLIM3 |  |
|  | C3orf27 |  |
|  | PNMA2 |  |
|  | SLC9A3 |  |
|  | FHL1 |  |
|  | FGFBP1 |  |
|  | MFAP3L |  |
|  | HRASLS |  |
|  | CXCL5 |  |
|  | SNCA |  |
|  | MYO3A |  |
|  | LOC151438 |  |
|  | OTC |  |
|  | CD1C |  |
|  | NRP2 |  |
|  | PLAU |  |
|  | CLU |  |
|  | CPNE5 |  |
|  | GP9 |  |
|  | SGK196 |  |
|  | VEPH1 |  |
|  | EGF |  |
|  | FAM151A |  |
|  | C4BPA |  |
|  | USP49 |  |
|  | LTBP1 |  |
|  | CTDSPL |  |
|  | BEND2 |  |
|  | LIFR |  |
|  | MPL |  |
|  | C7orf16 |  |
|  | CD1E |  |
|  | AQP10 |  |
|  | NEUROD1 |  |
|  | CLEC2L |  |
|  | PAPSS2 |  |
|  | RFTN2 |  |
|  | AGXT |  |
|  | NR2F1 |  |
|  | PHYHIPL |  |
|  | FAM171B |  |
|  | SVEP1 |  |
|  | HHIPL2 |  |
|  | GGTA1 |  |
|  | ALPL |  |
|  | SYTL4 |  |
|  | WDR38 |  |
|  | VSIG4 |  |
|  | CHSY3 |  |
|  | CACNA1D |  |
|  | FNDC5 |  |
|  | RLBP1L1 |  |
|  | GPR144 |  |
|  | NPFFR1 |  |
|  | TREML1 |  |
|  | ILDR1 |  |
|  | KIAA0485 |  |
|  | KCNE4 |  |
|  | LENEP |  |
|  | LOC157627 |  |
|  | PDPN |  |
|  | CXorf31 |  |
|  | MST1R |  |
|  | THBS2 |  |
|  | MGAM |  |
|  | HEY1 |  |
|  | OLAH |  |
|  | SH3BGRL2 |  |
|  | GC |  |
|  | FLJ31713 |  |
|  | BAT5 |  |
|  | CYLC1 |  |
|  | LOC339751 |  |
|  | INHBB |  |
|  | C2orf83 |  |
|  | PROS1 |  |
|  | C3orf48 |  |
|  | TRBV27 |  |
|  | CDR1 |  |
|  | NSUN7 |  |
|  | IL1F9 |  |
|  | PLA1A |  |
|  | PIGR |  |
|  | FLJ23834 |  |
|  | RYR2 |  |
|  | TEX15 |  |
|  | PCDHB4 |  |
|  | AWAT1 |  |
|  | NR0B2 |  |
|  | RSPO3 |  |
|  | SMR3A |  |
|  | C9orf53 |  |
|  | PLXNB3 |  |
|  | FAM26E |  |
|  | COL21A1 |  |
|  | ZNF462 |  |
|  | EYA1 |  |
|  | S100A5 |  |
|  | ZNF804B |  |
|  | STK32B |  |
|  | LOC645591 |  |
|  | ZNF704 |  |
|  | SPINK4 |  |
|  | RBP4 |  |
|  | F13B |  |
|  | SERPINI2 |  |
|  | C1orf98 |  |
|  | ST18 |  |
|  | FTHL17 |  |
|  | ACOT12 |  |
|  | OPALIN |  |
|  | CLRN3 |  |
|  | OR2S2 |  |
|  | SMYD1 |  |
|  | CNBD1 |  |
|  | LVRN |  |
|  | MAGEC1 |  |
|  | CDC25C |  |
|  | LEFTY2 |  |
|  | KIAA1751 |  |
|  | GABRB1 |  |
|  | hCG_1645220 |  |
|  | S100A14 |  |
|  | LOC200772 |  |
|  | FMR1NB |  |
|  | ADAM32 |  |
|  | MYPN |  |
|  | USP51 |  |
|  | NPHS2 |  |
|  | RNF186 |  |
|  | BEYLA |  |
|  | LOC401097 |  |
|  | ZNF732 |  |
|  | LMX1A |  |
|  | LOC158434 |  |
|  | LOC284578 |  |
|  | SPOCD1 |  |
|  | C10orf141 |  |
|  | FRK |  |
|  | CTTNBP2 |  |
|  | DIRC1 |  |
