# Supplementary material for: Immune cells transcriptome-based drug repositioning for multiple sclerosis
Source: Front Immunol. 2022 Oct 20;13:1020721. doi: 10.3389/fimmu.2022.1020721 (PMC9630342; doi:10.3389/fimmu.2022.1020721)
Supplement: Supplementary Table 3 — Detailed target genes obtained from MS patients after the application of Fingolimod or IFN-β according to the type of CD19+ B cells, CD4+ T cells, pDCs and PBMC. [file Table_3.docx]

| Sample | up-regulated target gene | down-regulated target gene |
| --- | --- | --- |
| CD19^+^ B cells |  | TOX |
|  |  | POLR3G |
| CD4^+^ T cells | TMEM45B | GNAI3 |
|  | SUSD4 | IPO7 |
|  | APBA2 | SMC1A |
|  | ACER1 | SEC23A |
|  | TSHZ2 | CAPN2 |
|  | CCR7 | RAB10 |
|  |  | SERP1 |
|  |  | ITM2B |
|  |  | HEXIM1 |
|  |  | IQGAP1 |
|  |  | ARFGEF2 |
|  |  | TMEM43 |
|  |  | GALNT1 |
|  |  | MYH9 |
|  |  | GMPS |
|  |  | ZMPSTE24 |
|  |  | YWHAQ |
|  |  | UBXN4 |
|  |  | PPP2R5A |
|  |  | ITGA5 |
|  |  | ITSN2 |
|  |  | AUP1 |
|  |  | SCYL2 |
|  |  | STXBP3 |
|  |  | PRKX |
|  |  | NUS1 |
|  |  | APOL6 |
|  |  | ARF6 |
|  |  | CHMP4B |
|  |  | AFTPH |
|  |  | SGPP1 |
|  |  | ABHD13 |
|  |  | RAP1B |
|  |  | SRP72 |
|  |  | RAB2A |
|  |  | WDFY1 |
|  |  | ZBTB33 |
|  |  | ACOT13 |
|  |  | LCP2 |
|  |  | UBP1 |
|  |  | ZFC3H1 |
|  |  | SLC7A5P2 |
|  |  | USP34 |
|  |  | KIF5B |
|  |  | FUCA2 |
|  |  | PDP1 |
|  |  | NADSYN1 |
|  |  | NFE2L3 |
|  |  | SRSF4 |
|  |  | SPTY2D1 |
|  |  | GNPAT |
|  |  | CIAO1 |
|  |  | TXNDC15 |
|  |  | SPEN |
|  |  | LMBRD1 |
|  |  | SLAIN2 |
|  |  | PSEN1 |
|  |  | NPEPPS |
|  |  | RAB18 |
|  |  | DHX36 |
|  |  | TLN1 |
|  |  | LYSMD3 |
|  |  | MAP1LC3B |
|  |  | MAT2A |
|  |  | APPL1 |
|  |  | ITGA4 |
|  |  | DCTN2 |
|  |  | ATP1B3 |
|  |  | DNM2 |
|  |  | VCL |
|  |  | CPD |
|  |  | CCDC6 |
|  |  | KPNA4 |
|  |  | SPCS3 |
|  |  | ACTR2 |
|  |  | EFCAB14 |
|  |  | SGK3 |
|  |  | SPPL2A |
|  |  | PRDM4 |
|  |  | PPM1D |
|  |  | GOLPH3 |
|  |  | TRIM25 |
|  |  | FAM91A1 |
|  |  | DHX15 |
|  |  | HSP90B1 |
|  |  | EXOC5 |
|  |  | MAP3K2 |
|  |  | DIAPH2 |
|  |  | EPM2AIP1 |
|  |  | PPTC7 |
|  |  | GNL3L |
|  |  | PTEN |
|  |  | CCSAP |
|  |  | ZNRF2 |
|  |  | PHIP |
|  |  | VPS13D |
|  |  | STX12 |
|  |  | NUFIP2 |
|  |  | ATP6V1C1 |
|  |  | IGF2R |
|  |  | SLK |
|  |  | CDC73 |
|  |  | RAB22A |
|  |  | STRN |
|  |  | RRN3P2 |
|  |  | RAB6A |
|  |  | MED21 |
|  |  | STK10 |
|  |  | SLC31A1 |
|  |  | KPNA6 |
|  |  | IFNAR1 |
|  |  | RNF103 |
|  |  | TMEM30A |
|  |  | TYMP |
|  |  | B4GALT5 |
|  |  | LIMS1 |
|  |  | COG5 |
|  |  | PRKCB |
|  |  | TRIQK |
|  |  | RYBP |
|  |  | GCLC |
|  |  | SMIM14 |
|  |  | TMEM167A |
|  |  | SSR1 |
|  |  | KRCC1 |
|  |  | C16orf72 |
|  |  | SFT2D2 |
|  |  | RAB11A |
|  |  | AP1G1 |
|  |  | YY1 |
|  |  | SNX3 |
|  |  | YWHAG |
|  |  | KPNA3 |
|  |  | CD164 |
|  |  | STX5 |
|  |  | PURB |
|  |  | VCPIP1 |
|  |  | RAP2A |
|  |  | KIF1B |
|  |  | AGTPBP1 |
|  |  | HOOK3 |
|  |  | LATS2 |
|  |  | CHSY1 |
|  |  | CACUL1 |
|  |  | SNX18 |
|  |  | TTL |
|  |  | ABHD2 |
|  |  | ERP44 |
|  |  | BNIP2 |
|  |  | LYST |
|  |  | ERN1 |
|  |  | LAMP2 |
|  |  | KPNB1 |
|  |  | SRCAP |
|  |  | SCAF8 |
|  |  | MANEA |
|  |  | SCARB2 |
|  |  | SP100 |
|  |  | LRRC58 |
|  |  | DLST |
|  |  | UBE2K |
|  |  | NCEH1 |
|  |  | SMG1P1 |
|  |  | RRAGC |
|  |  | PREX1 |
|  |  | SP1 |
|  |  | RAB12 |
|  |  | NHLRC3 |
|  |  | STRAP |
|  |  | PLEKHA3 |
|  |  | DSTN |
|  |  | RAB11FIP2 |
|  |  | PAPOLA |
|  |  | DOCK7 |
|  |  | MOSPD2 |
|  |  | IRF1 |
|  |  | RBBP4 |
|  |  | MBD4 |
|  |  | FAM102B |
|  |  | MYO9B |
|  |  | DUSP10 |
|  |  | KLHL18 |
|  |  | TAOK1 |
|  |  | NIPBL |
|  |  | NCOA1 |
|  |  | PRDM1 |
|  |  | SLC25A40 |
|  |  | SLC30A1 |
|  |  | GOLT1B |
|  |  | ATP6V1A |
|  |  | ITGAM |
|  |  | CA2 |
|  |  | CCNT2 |
|  |  | MAP3K1 |
|  |  | TMX3 |
|  |  | PDIA3 |
|  |  | ABHD15 |
|  |  | SCAMP1 |
|  |  | FGL2 |
|  |  | TET2 |
|  |  | YTHDF1 |
|  |  | CHML |
|  |  | ATP2A2 |
|  |  | FBXO30 |
|  |  | LYZ |
|  |  | SPTSSA |
|  |  | REEP3 |
|  |  | RASA1 |
|  |  | EIF4EBP2 |
|  |  | FAR1 |
|  |  | NCOA3 |
|  |  | ATL3 |
|  |  | OGDH |
|  |  | DKFZP586I1420 |
|  |  | NSUN2 |
|  |  | ZNF326 |
|  |  | NHLRC2 |
|  |  | ATP2B4 |
|  |  | MED13L |
|  |  | DOCK2 |
|  |  | NUP62 |
|  |  | RPS6KA1 |
|  |  | ZSWIM6 |
|  |  | MSH3 |
|  |  | TRAF7 |
|  |  | XYLT1 |
|  |  | PPID |
|  |  | TUG1 |
|  |  | MCOLN1 |
|  |  | AHNAK |
|  |  | GALNT10 |
|  |  | GPBP1L1 |
|  |  | INPPL1 |
|  |  | POLR2A |
|  |  | BNIP3L |
|  |  | NBPF10 |
|  |  | WDR44 |
|  |  | RDX |
|  |  | CPPED1 |
|  |  | NFATC2IP |
|  |  | VPS8 |
|  |  | LOC653653 |
|  |  | TMEM65 |
|  |  | RIPK3 |
|  |  | POLH |
|  |  | UBXN11 |
|  |  | SLC30A9 |
|  |  | HM13 |
|  |  | RAB14 |
|  |  | FBXL20 |
|  |  | PLXDC2 |
|  |  | DIAPH1 |
|  |  | MTMR9 |
|  |  | LTB4R |
|  |  | UBXN2B |
|  |  | DCUN1D2 |
|  |  | LOC202181 |
|  |  | LUZP6 |
|  |  | FTH1 |
|  |  | IAH1 |
|  |  | AGO2 |
|  |  | ARL8B |
|  |  | VPS41 |
|  |  | TACC1 |
|  |  | FOXN3 |
|  |  | INPP5D |
|  |  | CSF2RB |
|  |  | LIMD1 |
|  |  | AGFG1 |
|  |  | STX7 |
|  |  | POLR2E |
|  |  | SUZ12P1 |
|  |  | NCOR1 |
|  |  | FBXO34 |
|  |  | MKLN1 |
|  |  | TYW5 |
|  |  | SLC35A5 |
|  |  | RFFL |
|  |  | CALM2 |
|  |  | HNRNPR |
|  |  | TRIP12 |
|  |  | BICD2 |
|  |  | SMAP2 |
|  |  | GPR65 |
|  |  | BMPR2 |
|  |  | WASL |
|  |  | NAA50 |
|  |  | FAM120A |
|  |  | GNB4 |
|  |  | ATAD2B |
|  |  | CTDNEP1 |
|  |  | MBOAT1 |
|  |  | ZFP36L2 |
|  |  | C9orf72 |
|  |  | TPM4 |
|  |  | IPMK |
|  |  | HNRNPA2B1 |
|  |  | UBE2B |
|  |  | BCL10 |
|  |  | KIAA0754 |
|  |  | PIGF |
|  |  | ATXN1 |
|  |  | CPT1A |
|  |  | G2E3 |
|  |  | PCGF3 |
|  |  | ACIN1 |
|  |  | GMFB |
|  |  | SEC23IP |
|  |  | NPIPB3 |
|  |  | SMNDC1 |
|  |  | UTRN |
|  |  | SOS2 |
|  |  | PDE8A |
|  |  | MIS18BP1 |
|  |  | MRPS25 |
|  |  | UBASH3B |
|  |  | RAB7A |
|  |  | ETV6 |
|  |  | JAZF1 |
|  |  | ZNFX1 |
|  |  | PIEZO1 |
|  |  | PTP4A2 |
|  |  | FASTKD1 |
|  |  | TMEM138 |
|  |  | PIK3CG |
|  |  | BAG4 |
|  |  | CAPRIN1 |
|  |  | EIF4G2 |
|  |  | TRIM37 |
|  |  | TM9SF2 |
|  |  | TTLL3 |
|  |  | SRSF1 |
|  |  | BLOC1S6 |
|  |  | ARID1A |
|  |  | SLC35F6 |
|  |  | UBE3A |
|  |  | SLC35A3 |
|  |  | CCNK |
|  |  | ANKRD50 |
|  |  | EFHD2 |
|  |  | WASH3P |
|  |  | ARHGDIA |
|  |  | WDR33 |
|  |  | DCAF7 |
|  |  | KCNRG |
|  |  | MEGF9 |
|  |  | TRIM24 |
|  |  | UBE2A |
|  |  | WDFY2 |
|  |  | ST8SIA4 |
|  |  | ZFYVE16 |
|  |  | EIF3M |
|  |  | KSR1 |
|  |  | GOLGA1 |
|  |  | RB1 |
|  |  | MARCKS |
|  |  | CDK8 |
|  |  | YWHAB |
|  |  | RPS6KA3 |
|  |  | SMIM7 |
|  |  | BTN2A1 |
|  |  | HNRNPD |
|  |  | TMEM87A |
|  |  | LPXN |
|  |  | WSB1 |
|  |  | INO80 |
|  |  | LOC286437 |
|  |  | AGPS |
|  |  | PPP3R1 |
|  |  | GPR52 |
|  |  | MYCBP2 |
|  |  | MBNL2 |
|  |  | SLC9A9 |
|  |  | DARS2 |
|  |  | RNF11 |
|  |  | KCTD12 |
|  |  | ORMDL1 |
|  |  | UBE2D1 |
|  |  | MEF2C |
|  |  | DNAJC16 |
|  |  | FGR |
|  |  | ZFX |
|  |  | FLT3 |
|  |  | ATG16L2 |
|  |  | EXOC1 |
|  |  | NHSL2 |
|  |  | MTMR14 |
|  |  | PFDN1 |
|  |  | PHACTR4 |
|  |  | MXD1 |
|  |  | VCAN |
|  |  | KYNU |
|  |  | TNPO1 |
|  |  | ARF1 |
|  |  | PRR11 |
|  |  | ARAP1 |
|  |  | PARP12 |
|  |  | CD33 |
|  |  | CHP1 |
|  |  | CAMK2D |
|  |  | ETV3 |
|  |  | LOC652276 |
|  |  | CSK |
|  |  | TMEM168 |
|  |  | AGAP5 |
|  |  | RBM12B |
|  |  | CUL5 |
|  |  | FBXO9 |
|  |  | THOC2 |
|  |  | HERC4 |
|  |  | ARHGEF2 |
|  |  | KHDRBS1 |
|  |  | SNX29 |
|  |  | FCHO2 |
|  |  | LZIC |
|  |  | AUH |
|  |  | FGD6 |
|  |  | PRKACA |
|  |  | ZC2HC1A |
|  |  | QKI |
|  |  | DSN1 |
|  |  | TAS2R19 |
|  |  | USP38 |
|  |  | KANSL1L |
|  |  | IQCB1 |
|  |  | CD74 |
|  |  | LOC100190986 |
|  |  | LTA4H |
|  |  | UBE2R2 |
|  |  | MAP4K5 |
|  |  | NRP1 |
|  |  | GPR137B |
|  |  | CAPZA1 |
|  |  | BCL6 |
|  |  | SLC35F5 |
|  |  | ARHGAP31 |
|  |  | OTUD4 |
|  |  | PHTF2 |
|  |  | ELF4 |
|  |  | TGFB1 |
|  |  | TVP23C |
|  |  | TMEM170B |
|  |  | FRY |
|  |  | PIGB |
|  |  | PA2G4P4 |
|  |  | ZNF706 |
|  |  | ATG7 |
|  |  | SH2B3 |
|  |  | RHOQ |
|  |  | MAP2K5 |
|  |  | ITPK1 |
|  |  | PAK2 |
|  |  | YWHAH |
|  |  | SSH2 |
|  |  | ACSL1 |
|  |  | NPIPB5 |
|  |  | ADNP |
|  |  | LRCH1 |
|  |  | CPNE8 |
|  |  | CTNNA1 |
|  |  | PPM1M |
|  |  | DENND6A |
|  |  | MAP4K3 |
|  |  | SCNM1 |
|  |  | PLEKHM1 |
|  |  | PRDM2 |
|  |  | DEDD |
|  |  | CPOX |
|  |  | CLIP4 |
|  |  | FMR1 |
|  |  | PID1 |
|  |  | DDX60L |
|  |  | PHF12 |
|  |  | MICAL2 |
|  |  | SACM1L |
|  |  | MTMR10 |
|  |  | SLC39A11 |
|  |  | CORO1C |
|  |  | MEF2A |
|  |  | ARPC2 |
|  |  | RNF13 |
|  |  | DNM1L |
|  |  | LRRK2 |
|  |  | RAPGEF1 |
|  |  | UBE2E2 |
|  |  | TMEM164 |
|  |  | NPIPB11 |
|  |  | MIR3140 |
|  |  | CLTC |
|  |  | TNKS2 |
|  |  | FIG4 |
|  |  | COX15 |
|  |  | CMIP |
|  |  | KMO |
|  |  | SKAP2 |
|  |  | PARP14 |
|  |  | MIR548C |
|  |  | ARSB |
|  |  | ARHGAP26 |
|  |  | MIR4742 |
|  |  | ROCK1P1 |
|  |  | SPATA6 |
|  |  | CHD1 |
|  |  | GNLY |
|  |  | MIR4802 |
|  |  | GUSB |
|  |  | CREB1 |
|  |  | MIR624 |
|  |  | NOTCH2 |
|  |  | CCDC18 |
|  |  | SERPINB8 |
|  |  | MIR142 |
|  |  | PLAA |
|  |  | FMNL1 |
|  |  | LACTB |
|  |  | LCORL |
|  |  | ARF3 |
|  |  | TBCE |
|  |  | TBC1D2 |
|  |  | MIR186 |
|  |  | SNORD56B |
|  |  | TTC7A |
|  |  | PXN |
|  |  | EML4 |
|  |  | RHOG |
|  |  | UNC93B1 |
|  |  | ADCY7 |
|  |  | CKAP2 |
|  |  | TP53 |
|  |  | SLC43A2 |
|  |  | PPP2R5E |
|  |  | SLC15A2 |
|  |  | LINC00294 |
|  |  | PCCA |
|  |  | DEPDC5 |
|  |  | PAK1 |
|  |  | EVI2B |
|  |  | GPR34 |
|  |  | MARK3 |
|  |  | SLCO3A1 |
|  |  | MAP2K4 |
|  |  | PTGDS |
|  |  | SIN3B |
|  |  | MIR4420 |
|  |  | LPCAT2 |
|  |  | LPCAT1 |
|  |  | PI4KA |
|  |  | TMUB2 |
|  |  | PTPN22 |
|  |  | TMEM154 |
|  |  | ACER3 |
|  |  | RAP1GAP2 |
|  |  | TLK2 |
|  |  | NOMO2 |
|  |  | EIF4G3 |
|  |  | SIGLEC14 |
|  |  | GTF2A1 |
|  |  | RMDN1 |
|  |  | STAU1 |
|  |  | MYO1F |
|  |  | SEC24D |
|  |  | CTNND1 |
|  |  | KMT2D |
|  |  | CDK19 |
|  |  | CMC1 |
|  |  | AP3D1 |
|  |  | CSPP1 |
|  |  | ZSCAN9 |
|  |  | SMARCC1 |
|  |  | SMN1 |
|  |  | DPRXP4 |
|  |  | GNAI2 |
|  |  | BRD2 |
|  |  | NAPB |
|  |  | FAM135A |
|  |  | SMAD5 |
|  |  | CDCA7 |
|  |  | ZBTB34 |
|  |  | GTF2H2B |
|  |  | NBPF9 |
| pDCs | ZNF711 | TMEM27 |
|  | C16orf87 | CARS |
|  | BCL2L11 | THTPA |
|  | HIST1H4E | DHX58 |
|  | KLHL20 | KIAA0040 |
|  | CHEK1 | F11R |
|  | GADD45A | ADCK1 |
|  | GLB1L | MYD88 |
|  | HSPC081 | COX5A |
|  | NFE2L3 | FAM46D |
|  | IRS2 | DCBLD1 |
|  | GPR83 | MYCL |
|  | POLG | TXN |
|  | IQCC | TMEM110 |
|  | RP5-1065J22.8 | AKR1A1 |
|  | HMGB3 | LINC01432 |
|  | RAB11FIP1 | TNFAIP8L1 |
|  | CHPT1 | PHF11 |
|  | PIGA | C19orf24 |
|  | BC041363 | IFI16 |
|  | RAB33B | DDX26B |
|  | ISG20L2 | FARSA |
|  | TPM3 | CHST12 |
|  | FYTTD1 | GNS |
|  | LOC101927211 | AGPAT4-IT1 |
|  | SNAI1 | ANKIB1 |
|  | CCNY | COG4 |
|  | UBXN2A | HERC6 |
|  | TADA1 | TMEM86A |
|  | HIST1H2BH | HIRIP3 |
|  | NXT1 | CD38 |
|  | RP9 | TM9SF1 |
|  | NUBPL | IRF7 |
|  | LOC101928371 | TNFSF13B |
|  | EPB42 | TINF2 |
|  | MEX3C | RAB29 |
|  | LINC00565 | HGH1 |
|  | C1orf52 | LOC100507535 |
|  | RNF139 | TDRD7 |
|  | PRNP | TOR1B |
|  | FAM217A | CD180 |
|  | TLE4 | IPO11 |
|  | PRDM2 | CHMP5 |
|  | AC139100.3 | LOC283745 |
|  | MTPAP | SECTM1 |
|  | HIST1H4J | TRIM14 |
|  | PHF1 | ELMO1 |
|  | MXI1 | MX2 |
|  | BMF | PARP12 |
|  | TOE1 | USP18 |
|  | MAD2L1BP | FIG4 |
|  | KIAA1432 | TRIM69 |
|  | SENP5 | C17orf62 |
|  | ABAT | BTN3A3 |
|  | SFT2D3 | DDX60 |
|  | EIF1 | IFI44L |
|  | HIST1H1T | CKS2 |
|  | ARL4A |  |
|  | CPSF6 |  |
|  | HIST1H3C |  |
|  | DNAJB8-AS1 |  |
|  | FEM1B |  |
|  | MAP4K3 |  |
|  | DNAJC1 |  |
|  | DLL1 |  |
|  | DDX59 |  |
|  | PPP1R17 |  |
|  | IFT57 |  |
|  | RP11-473I1.9 |  |
|  | CMTM4 |  |
|  | ZNF814 |  |
|  | SIK1 |  |
|  | ACVR1B |  |
|  | FBXL5 |  |
|  | RC3H1 |  |
|  | SIAH1 |  |
|  | TFDP2 |  |
|  | RNF168 |  |
|  | RP1-263J7.2 |  |
|  | ZC3H15 |  |
|  | JOSD1 |  |
|  | KAT6B |  |
|  | NFYA |  |
|  | SLC35D1 |  |
|  | COG3 |  |
|  | CAPRIN1 |  |
|  | DCTN4 |  |
|  | SELT |  |
|  | LOC102723918 |  |
|  | ATP11A |  |
|  | ZNF292 |  |
|  | MORF4L1 |  |
|  | FAM161A |  |
|  | TAOK2 |  |
|  | SLC25A36 |  |
|  | LOC101929114 |  |
|  | WBP4 |  |
| PBMC | CYBRD1 | CHST10 |
|  | SH3BGRL2 | LAMP3 |
|  | CTDSPL | EFNA1 |
|  | TREML1 | SYN2 |
|  | SELP | CLDN23 |
|  | F13A1 | ZNF713 |
|  | PRKAR2B | SNTG1 |
|  | PTGS1 | IGFBP1 |
|  | GNG11 | SCIN |
|  | GUCY1B3 | DUSP9 |
|  | LTBP1 | ACTL6B |
|  | BEND2 | LCE2B |
|  | CDA |  |
|  | SPARC |  |
|  | CLU |  |
|  | FHL1 |  |
|  | MGAM |  |
|  | PDE5A |  |
|  | PGD |  |
|  | REPS2 |  |
|  | PADI4 |  |
|  | PAPSS2 |  |
|  | C1orf198 |  |
|  | EHD3 |  |
|  | PGRMC1 |  |
|  | EGF |  |
|  | MPP1 |  |
|  | MFAP3L |  |
|  | VEPH1 |  |
|  | TMEM40 |  |
|  | TNFSF4 |  |
|  | ELOVL7 |  |
|  | MBOAT2 |  |
|  | PLA2G12A |  |
|  | CCNJL |  |
|  | ITGB5 |  |
|  | AQP10 |  |
|  | ASAP2 |  |
|  | TSPAN33 |  |
|  | CDC14B |  |
|  | DNAJC8 |  |
|  | TFPI |  |
|  | PIK3CB |  |
|  | FSTL1 |  |
|  | CLCN3 |  |
|  | SDPR |  |
|  | ABLIM3 |  |
|  | ARHGAP18 |  |
|  | ENTPD1 |  |
|  | PDLIM1 |  |
|  | SCFD2 |  |
|  | ODC1 |  |
|  | LEPR |  |
|  | GSN |  |
|  | CSF2RA |  |
|  | TKT |  |
|  | HADHA |  |
|  | ST7 |  |
|  | BICD2 |  |
|  | FMO5 |  |
|  | AGBL5 |  |
