# Supplementary material for: Immune cells transcriptome-based drug repositioning for multiple sclerosis
Source: Front Immunol. 2022 Oct 20;13:1020721. doi: 10.3389/fimmu.2022.1020721 (PMC9630342; doi:10.3389/fimmu.2022.1020721)
Supplement: Supplementary Table 4 — Detailed pathways obtained from MS patients without treatment according to the type of CD19+ B cells, CD4+ T cells, pDCs and PBMC. [file Table_4.docx]

| Sample | Pathway ID | Pathway Name | P value |
| --- | --- | --- | --- |
| CD19^+^ B cells | hsa04659 | Th17 cell differentiation | 9.16E-05 |
|  | hsa04062 | Chemokine signaling pathway | 0.000221798 |
|  | hsa05321 | Inflammatory bowel disease (IBD) | 0.000227808 |
|  | hsa04640 | Hematopoietic cell lineage | 0.000338987 |
|  | hsa04725 | Cholinergic synapse | 0.000760817 |
|  | hsa04630 | JAK-STAT signaling pathway | 0.001505052 |
|  | hsa00380 | Tryptophan metabolism | 0.002369894 |
|  | hsa04917 | Prolactin signaling pathway | 0.002464609 |
|  | hsa05152 | Tuberculosis | 0.002817287 |
|  | hsa04066 | HIF-1 signaling pathway | 0.003462951 |
|  | hsa05167 | Kaposi sarcoma-associated herpesvirus infection | 0.003565389 |
|  | hsa05169 | Epstein-Barr virus infection | 0.005683169 |
|  | hsa05235 | PD-L1 expression and PD-1 checkpoint pathway in cancer | 0.006910268 |
|  | hsa05170 | Human immunodeficiency virus 1 infection | 0.007766152 |
|  | hsa04658 | Th1 and Th2 cell differentiation | 0.007931557 |
|  | hsa05164 | Influenza A | 0.007986402 |
|  | hsa04650 | Natural killer cell mediated cytotoxicity | 0.008427861 |
|  | hsa04713 | Circadian entrainment | 0.009859734 |
|  | hsa05163 | Human cytomegalovirus infection | 0.010918157 |
|  | hsa04061 | Viral protein interaction with cytokine and cytokine receptor | 0.011159698 |
|  | hsa04750 | Inflammatory mediator regulation of TRP channels | 0.011159698 |
|  | hsa04620 | Toll-like receptor signaling pathway | 0.013068923 |
|  | hsa04625 | C-type lectin receptor signaling pathway | 0.013068923 |
|  | hsa04932 | Non-alcoholic fatty liver disease (NAFLD) | 0.015252073 |
|  | hsa04668 | TNF signaling pathway | 0.017526571 |
|  | hsa05160 | Hepatitis C | 0.01819676 |
|  | hsa04971 | Gastric acid secretion | 0.018349949 |
|  | hsa05205 | Proteoglycans in cancer | 0.020180605 |
|  | hsa01521 | EGFR tyrosine kinase inhibitor resistance | 0.021779435 |
|  | hsa04217 | Necroptosis | 0.022100556 |
|  | hsa04935 | Growth hormone synthesis, secretion and action | 0.022171241 |
|  | hsa05132 | Salmonella infection | 0.022694017 |
|  | hsa05135 | Yersinia infection | 0.022894066 |
|  | hsa03013 | RNA transport | 0.023934875 |
|  | hsa04662 | B cell receptor signaling pathway | 0.024592777 |
|  | hsa04742 | Taste transduction | 0.025577191 |
|  | hsa04012 | ErbB signaling pathway | 0.02761662 |
|  | hsa04380 | Osteoclast differentiation | 0.029230325 |
|  | hsa04728 | Dopaminergic synapse | 0.031866075 |
|  | hsa04120 | Ubiquitin mediated proteolysis | 0.036581805 |
|  | hsa04912 | GnRH signaling pathway | 0.03672764 |
|  | hsa04657 | IL-17 signaling pathway | 0.037974732 |
|  | hsa05162 | Measles | 0.038582515 |
|  | hsa05134 | Legionellosis | 0.039592317 |
|  | hsa00563 | Glycosylphosphatidylinositol (GPI)-anchor biosynthesis | 0.044283235 |
|  | hsa04060 | Cytokine-cytokine receptor interaction | 0.044865217 |
|  | hsa04933 | AGE-RAGE signaling pathway in diabetic complications | 0.045965182 |
|  | hsa04020 | Calcium signaling pathway | 0.04610737 |
|  | hsa05142 | Chagas disease (American trypanosomiasis) | 0.048822094 |
|  | hsa00310 | Lysine degradation | 0.049004779 |
| CD4^+^ T cells | hsa03010 | Ribosome | 2.20E-23 |
|  | hsa04722 | Neurotrophin signaling pathway | 0.00021808 |
|  | hsa04120 | Ubiquitin mediated proteolysis | 0.000603435 |
|  | hsa04114 | Oocyte meiosis | 0.000627995 |
|  | hsa04912 | GnRH signaling pathway | 0.001258332 |
|  | hsa04713 | Circadian entrainment | 0.002027243 |
|  | hsa04971 | Gastric acid secretion | 0.002688323 |
|  | hsa04540 | Gap junction | 0.004772326 |
|  | hsa04070 | Phosphatidylinositol signaling system | 0.006184094 |
|  | hsa04921 | Oxytocin signaling pathway | 0.006359927 |
|  | hsa04931 | Insulin resistance | 0.00636377 |
|  | hsa04750 | Inflammatory mediator regulation of TRP channels | 0.006822177 |
|  | hsa04934 | Cushing syndrome | 0.007409375 |
|  | hsa04720 | Long-term potentiation | 0.007423102 |
|  | hsa04371 | Apelin signaling pathway | 0.007651948 |
|  | hsa04010 | MAPK signaling pathway | 0.009444539 |
|  | hsa04728 | Dopaminergic synapse | 0.009842432 |
|  | hsa05161 | Hepatitis B | 0.012249526 |
|  | hsa04927 | Cortisol synthesis and secretion | 0.01536883 |
|  | hsa04919 | Thyroid hormone signaling pathway | 0.01628937 |
|  | hsa04935 | Growth hormone synthesis, secretion and action | 0.01628937 |
|  | hsa05135 | Yersinia infection | 0.017589929 |
|  | hsa04670 | Leukocyte transendothelial migration | 0.01940555 |
|  | hsa04144 | Endocytosis | 0.019856005 |
|  | hsa05211 | Renal cell carcinoma | 0.023313806 |
|  | hsa04611 | Platelet activation | 0.023608725 |
|  | hsa04962 | Vasopressin-regulated water reabsorption | 0.024401294 |
|  | hsa05120 | Epithelial cell signaling in Helicobacter pylori infection | 0.025698148 |
|  | hsa04330 | Notch signaling pathway | 0.026482242 |
|  | hsa04961 | Endocrine and other factor-regulated calcium reabsorption | 0.026482242 |
|  | hsa04914 | Progesterone-mediated oocyte maturation | 0.029867478 |
|  | hsa03015 | mRNA surveillance pathway | 0.03304494 |
|  | hsa04261 | Adrenergic signaling in cardiomyocytes | 0.034210896 |
|  | hsa04062 | Chemokine signaling pathway | 0.034494989 |
|  | hsa04152 | AMPK signaling pathway | 0.034624144 |
|  | hsa03060 | Protein export | 0.035184412 |
|  | hsa00562 | Inositol phosphate metabolism | 0.037011621 |
|  | hsa04725 | Cholinergic synapse | 0.038598501 |
|  | hsa05170 | Human immunodeficiency virus 1 infection | 0.039475468 |
|  | hsa04151 | PI3K-Akt signaling pathway | 0.048767547 |
| pDCs | hsa05323 | Rheumatoid arthritis | 2.63E-05 |
|  | hsa05134 | Legionellosis | 7.62E-05 |
|  | hsa05161 | Hepatitis B | 0.000136272 |
|  | hsa04064 | NF-kappa B signaling pathway | 0.000312213 |
|  | hsa04657 | IL-17 signaling pathway | 0.000313386 |
|  | hsa04210 | Apoptosis | 0.000337654 |
|  | hsa04140 | Autophagy - animal | 0.000376578 |
|  | hsa04620 | Toll-like receptor signaling pathway | 0.000403797 |
|  | hsa05203 | Viral carcinogenesis | 0.000951157 |
|  | hsa04928 | Parathyroid hormone synthesis, secretion and action | 0.001382813 |
|  | hsa05163 | Human cytomegalovirus infection | 0.001396041 |
|  | hsa04962 | Vasopressin-regulated water reabsorption | 0.001771075 |
|  | hsa04621 | NOD-like receptor signaling pathway | 0.001825414 |
|  | hsa03022 | Basal transcription factors | 0.002120553 |
|  | hsa05142 | Chagas disease (American trypanosomiasis) | 0.002287095 |
|  | hsa04668 | TNF signaling pathway | 0.002610734 |
|  | hsa05167 | Kaposi sarcoma-associated herpesvirus infection | 0.002691928 |
|  | hsa04068 | FoxO signaling pathway | 0.00290038 |
|  | hsa05132 | Salmonella infection | 0.003428232 |
|  | hsa05144 | Malaria | 0.00411082 |
|  | hsa05222 | Small cell lung cancer | 0.00500765 |
|  | hsa05152 | Tuberculosis | 0.006549266 |
|  | hsa05164 | Influenza A | 0.006891662 |
|  | hsa05216 | Thyroid cancer | 0.00690808 |
|  | hsa00020 | Citrate cycle (TCA cycle) | 0.007327484 |
|  | hsa05202 | Transcriptional misregulation in cancer | 0.010428251 |
|  | hsa04144 | Endocytosis | 0.011768687 |
|  | hsa05223 | Non-small cell lung cancer | 0.012913337 |
|  | hsa05219 | Bladder cancer | 0.012964284 |
|  | hsa05133 | Pertussis | 0.014622741 |
|  | hsa05140 | Leishmaniasis | 0.014622741 |
|  | hsa05210 | Colorectal cancer | 0.016010104 |
|  | hsa04120 | Ubiquitin mediated proteolysis | 0.01934592 |
|  | hsa03018 | RNA degradation | 0.019496584 |
|  | hsa04610 | Complement and coagulation cascades | 0.019496584 |
|  | hsa05120 | Epithelial cell signaling in Helicobacter pylori infection | 0.019601359 |
|  | hsa04061 | Viral protein interaction with cytokine and cytokine receptor | 0.023706049 |
|  | hsa04115 | p53 signaling pathway | 0.023773807 |
|  | hsa00900 | Terpenoid backbone biosynthesis | 0.025161674 |
|  | hsa04145 | Phagosome | 0.028469416 |
|  | hsa01523 | Antifolate resistance | 0.030995576 |
|  | hsa04110 | Cell cycle | 0.032622071 |
|  | hsa04672 | Intestinal immune network for IgA production | 0.035346302 |
|  | hsa05213 | Endometrial cancer | 0.035522693 |
|  | hsa05215 | Prostate cancer | 0.039130357 |
|  | hsa04062 | Chemokine signaling pathway | 0.039558223 |
|  | hsa05162 | Measles | 0.041550473 |
|  | hsa05211 | Renal cell carcinoma | 0.041740395 |
|  | hsa04380 | Osteoclast differentiation | 0.042011691 |
|  | hsa05169 | Epstein-Barr virus infection | 0.042431721 |
|  | hsa00860 | Porphyrin and chlorophyll metabolism | 0.043082212 |
|  | hsa05170 | Human immunodeficiency virus 1 infection | 0.043144873 |
|  | hsa01210 | 2-Oxocarboxylic acid metabolism | 0.047490552 |
|  | hsa05135 | Yersinia infection | 0.047919856 |
| Peripheral blood leukocytes | hsa00600 | Sphingolipid metabolism | 5.39E-05 |
|  | hsa00052 | Galactose metabolism | 0.000160357 |
|  | hsa05134 | Legionellosis | 0.000326601 |
|  | hsa04071 | Sphingolipid signaling pathway | 0.000427746 |
|  | hsa04611 | Platelet activation | 0.000723508 |
|  | hsa04640 | Hematopoietic cell lineage | 0.000930857 |
|  | hsa05135 | Yersinia infection | 0.001273627 |
|  | hsa04621 | NOD-like receptor signaling pathway | 0.001309943 |
|  | hsa04810 | Regulation of actin cytoskeleton | 0.001335665 |
|  | hsa05142 | Chagas disease (American trypanosomiasis) | 0.001444198 |
|  | hsa04664 | Fc epsilon RI signaling pathway | 0.002006639 |
|  | hsa05230 | Central carbon metabolism in cancer | 0.002281911 |
|  | hsa05418 | Fluid shear stress and atherosclerosis | 0.0028572 |
|  | hsa00524 | Neomycin, kanamycin and gentamicin biosynthesis | 0.002881802 |
|  | hsa05145 | Toxoplasmosis | 0.003840852 |
|  | hsa04620 | Toll-like receptor signaling pathway | 0.004603772 |
|  | hsa04625 | C-type lectin receptor signaling pathway | 0.004603772 |
|  | hsa00010 | Glycolysis / Gluconeogenesis | 0.006072074 |
|  | hsa04670 | Leukocyte transendothelial migration | 0.009151757 |
|  | hsa04510 | Focal adhesion | 0.009166113 |
|  | hsa00500 | Starch and sucrose metabolism | 0.009965859 |
|  | hsa05321 | Inflammatory bowel disease (IBD) | 0.012322242 |
|  | hsa00030 | Pentose phosphate pathway | 0.014542112 |
|  | hsa05221 | Acute myeloid leukemia | 0.015110051 |
|  | hsa04145 | Phagosome | 0.015729057 |
|  | hsa05231 | Choline metabolism in cancer | 0.015819954 |
|  | hsa04066 | HIF-1 signaling pathway | 0.016368479 |
|  | hsa05144 | Malaria | 0.017178093 |
|  | hsa04610 | Complement and coagulation cascades | 0.018186307 |
|  | hsa04062 | Chemokine signaling pathway | 0.018756602 |
|  | hsa05219 | Bladder cancer | 0.019987376 |
|  | hsa05120 | Epithelial cell signaling in Helicobacter pylori infection | 0.020137101 |
|  | hsa05152 | Tuberculosis | 0.020332873 |
|  | hsa05323 | Rheumatoid arthritis | 0.02433842 |
|  | hsa04660 | T cell receptor signaling pathway | 0.024829725 |
|  | hsa05100 | Bacterial invasion of epithelial cells | 0.02628888 |
|  | hsa00920 | Sulfur metabolism | 0.026703199 |
|  | hsa00590 | Arachidonic acid metabolism | 0.026758686 |
|  | hsa04010 | MAPK signaling pathway | 0.030448683 |
|  | hsa00511 | Other glycan degradation | 0.030874869 |
|  | hsa00564 | Glycerophospholipid metabolism | 0.032575292 |
|  | hsa05215 | Prostate cancer | 0.032575292 |
|  | hsa03320 | PPAR signaling pathway | 0.033681353 |
|  | hsa05140 | Leishmaniasis | 0.033681353 |
|  | hsa05212 | Pancreatic cancer | 0.033681353 |
|  | hsa04144 | Endocytosis | 0.034134781 |
|  | hsa05163 | Human cytomegalovirus infection | 0.034377186 |
|  | hsa04930 | Type II diabetes mellitus | 0.035510575 |
|  | hsa00480 | Glutathione metabolism | 0.035697449 |
|  | hsa05160 | Hepatitis C | 0.035983886 |
|  | hsa01212 | Fatty acid metabolism | 0.039142023 |
|  | hsa05205 | Proteoglycans in cancer | 0.039300366 |
|  | hsa05235 | PD-L1 expression and PD-1 checkpoint pathway in cancer | 0.039916099 |
|  | hsa04360 | Axon guidance | 0.040874365 |
|  | hsa04142 | Lysosome | 0.041295081 |
|  | hsa04668 | TNF signaling pathway | 0.042103105 |
|  | hsa01521 | EGFR tyrosine kinase inhibitor resistance | 0.04241793 |
|  | hsa00520 | Amino sugar and nucleotide sugar metabolism | 0.043478255 |
