# Supplementary material for: Immune cells transcriptome-based drug repositioning for multiple sclerosis
Source: Front Immunol. 2022 Oct 20;13:1020721. doi: 10.3389/fimmu.2022.1020721 (PMC9630342; doi:10.3389/fimmu.2022.1020721)
Supplement: Supplementary Table 5 — Detailed pathways obtained from MS patients before and after the application of Fingolimod and IFN-β according to the type of CD19+ B cells, CD4+ T cells, pDCs and PBMC. [file Table_5.docx]

| Sample | Pathway ID | Pathway Name | P value |
| --- | --- | --- | --- |
| CD19^+^ B cells | hsa04740 | Olfactory transduction | 4.21E-17 |
|  | hsa05034 | Alcoholism | 8.75E-05 |
|  | hsa05322 | Systemic lupus erythematosus | 0.000125885 |
|  | hsa05203 | Viral carcinogenesis | 0.000245449 |
|  | hsa05135 | Yersinia infection | 0.000720982 |
|  | hsa04010 | MAPK signaling pathway | 0.002381373 |
|  | hsa04144 | Endocytosis | 0.002462489 |
|  | hsa04810 | Regulation of actin cytoskeleton | 0.003697803 |
|  | hsa04114 | Oocyte meiosis | 0.004092419 |
|  | hsa04360 | Axon guidance | 0.006546173 |
|  | hsa04722 | Neurotrophin signaling pathway | 0.007804129 |
|  | hsa05131 | Shigellosis | 0.013039804 |
|  | hsa04520 | Adherens junction | 0.017009285 |
|  | hsa04120 | Ubiquitin mediated proteolysis | 0.017695513 |
|  | hsa04216 | Ferroptosis | 0.028296241 |
|  | hsa05163 | Human cytomegalovirus infection | 0.031520909 |
|  | hsa04660 | T cell receptor signaling pathway | 0.031873249 |
|  | hsa05130 | Pathogenic Escherichia coli infection | 0.032879493 |
|  | hsa04962 | Vasopressin-regulated water reabsorption | 0.038439013 |
|  | hsa04310 | Wnt signaling pathway | 0.044033743 |
|  | hsa04720 | Long-term potentiation | 0.045174279 |
|  | hsa00600 | Sphingolipid metabolism | 0.047244912 |
|  | hsa05031 | Amphetamine addiction | 0.047651285 |
|  | hsa04062 | Chemokine signaling pathway | 0.049424268 |
| CD4^+^ T cells | hsa04740 | Olfactory transduction | 5.69E-32 |
|  | hsa04145 | Phagosome | 2.48E-10 |
|  | hsa04142 | Lysosome | 4.16E-10 |
|  | hsa05152 | Tuberculosis | 1.91E-09 |
|  | hsa04621 | NOD-like receptor signaling pathway | 6.40E-08 |
|  | hsa05150 | Staphylococcus aureus infection | 2.06E-07 |
|  | hsa05164 | Influenza A | 9.65E-06 |
|  | hsa05169 | Epstein-Barr virus infection | 1.74E-05 |
|  | hsa03050 | Proteasome | 3.33E-05 |
|  | hsa05130 | Pathogenic Escherichia coli infection | 0.000180514 |
|  | hsa04144 | Endocytosis | 0.00032481 |
|  | hsa04141 | Protein processing in endoplasmic reticulum | 0.000449513 |
|  | hsa05131 | Shigellosis | 0.000528364 |
|  | hsa04972 | Pancreatic secretion | 0.000648872 |
|  | hsa05110 | Vibrio cholerae infection | 0.000706344 |
|  | hsa04915 | Estrogen signaling pathway | 0.000731198 |
|  | hsa05120 | Epithelial cell signaling in Helicobacter pylori infection | 0.000811912 |
|  | hsa04510 | Focal adhesion | 0.000886799 |
|  | hsa05145 | Toxoplasmosis | 0.001106492 |
|  | hsa04614 | Renin-angiotensin system | 0.001260987 |
|  | hsa05132 | Salmonella infection | 0.001662934 |
|  | hsa04966 | Collecting duct acid secretion | 0.001706181 |
|  | hsa05140 | Leishmaniasis | 0.001724659 |
|  | hsa04392 | Hippo signaling pathway - multiple species | 0.001903387 |
|  | hsa04974 | Protein digestion and absorption | 0.001957223 |
|  | hsa05323 | Rheumatoid arthritis | 0.002012764 |
|  | hsa04926 | Relaxin signaling pathway | 0.002039467 |
|  | hsa04062 | Chemokine signaling pathway | 0.002790771 |
|  | hsa05160 | Hepatitis C | 0.003501482 |
|  | hsa04670 | Leukocyte transendothelial migration | 0.00369443 |
|  | hsa04666 | Fc gamma R-mediated phagocytosis | 0.003824202 |
|  | hsa04216 | Ferroptosis | 0.004266636 |
|  | hsa04935 | Growth hormone synthesis, secretion and action | 0.004278335 |
|  | hsa05146 | Amoebiasis | 0.004429076 |
|  | hsa04380 | Osteoclast differentiation | 0.004682371 |
|  | hsa05163 | Human cytomegalovirus infection | 0.004695109 |
|  | hsa05135 | Yersinia infection | 0.005484866 |
|  | hsa04512 | ECM-receptor interaction | 0.005658872 |
|  | hsa04927 | Cortisol synthesis and secretion | 0.00574181 |
|  | hsa05161 | Hepatitis B | 0.006123467 |
|  | hsa04662 | B cell receptor signaling pathway | 0.006257864 |
|  | hsa04530 | Tight junction | 0.006602769 |
|  | hsa04612 | Antigen processing and presentation | 0.006679027 |
|  | hsa04071 | Sphingolipid signaling pathway | 0.007257829 |
|  | hsa05416 | Viral myocarditis | 0.008700527 |
|  | hsa05322 | Systemic lupus erythematosus | 0.008907131 |
|  | hsa04650 | Natural killer cell mediated cytotoxicity | 0.009286753 |
|  | hsa04217 | Necroptosis | 0.009494621 |
|  | hsa04722 | Neurotrophin signaling pathway | 0.011936581 |
|  | hsa00531 | Glycosaminoglycan degradation | 0.013444255 |
|  | hsa04611 | Platelet activation | 0.013608821 |
|  | hsa05014 | Amyotrophic lateral sclerosis (ALS) | 0.013937127 |
|  | hsa04728 | Dopaminergic synapse | 0.014726777 |
|  | hsa05100 | Bacterial invasion of epithelial cells | 0.018188392 |
|  | hsa05167 | Kaposi sarcoma-associated herpesvirus infection | 0.018710847 |
|  | hsa04140 | Autophagy - animal | 0.01996403 |
|  | hsa04979 | Cholesterol metabolism | 0.020082204 |
|  | hsa04721 | Synaptic vesicle cycle | 0.021773979 |
|  | hsa04962 | Vasopressin-regulated water reabsorption | 0.0219153 |
|  | hsa04918 | Thyroid hormone synthesis | 0.023643451 |
|  | hsa04120 | Ubiquitin mediated proteolysis | 0.025066132 |
|  | hsa05320 | Autoimmune thyroid disease | 0.026585617 |
|  | hsa04151 | PI3K-Akt signaling pathway | 0.02658985 |
|  | hsa04610 | Complement and coagulation cascades | 0.027822528 |
|  | hsa00140 | Steroid hormone biosynthesis | 0.031492233 |
|  | hsa00480 | Glutathione metabolism | 0.034150879 |
|  | hsa05134 | Legionellosis | 0.034150879 |
|  | hsa04810 | Regulation of actin cytoskeleton | 0.034938842 |
|  | hsa04720 | Long-term potentiation | 0.03598929 |
|  | hsa05310 | Asthma | 0.037670862 |
|  | hsa00591 | Linoleic acid metabolism | 0.038506764 |
|  | hsa04724 | Glutamatergic synapse | 0.040435051 |
|  | hsa04080 | Neuroactive ligand-receptor interaction | 0.043366866 |
|  | hsa04664 | Fc epsilon RI signaling pathway | 0.04579397 |
|  | hsa05033 | Nicotine addiction | 0.046779262 |
| pDCs | hsa05164 | Influenza A | 1.58E-06 |
|  | hsa05169 | Epstein-Barr virus infection | 1.87E-05 |
|  | hsa05160 | Hepatitis C | 0.000110196 |
|  | hsa05162 | Measles | 0.001384927 |
|  | hsa05168 | Herpes simplex virus 1 infection | 0.003419491 |
|  | hsa05020 | Prion diseases | 0.004567841 |
|  | hsa05142 | Chagas disease (American trypanosomiasis) | 0.004892712 |
|  | hsa04622 | RIG-I-like receptor signaling pathway | 0.006441629 |
|  | hsa05322 | Systemic lupus erythematosus | 0.009440687 |
|  | hsa04621 | NOD-like receptor signaling pathway | 0.012252603 |
|  | hsa05217 | Basal cell carcinoma | 0.014301247 |
|  | hsa05216 | Thyroid cancer | 0.028602999 |
|  | hsa04550 | Signaling pathways regulating pluripotency of stem cells | 0.033443988 |
|  | hsa00240 | Pyrimidine metabolism | 0.033677932 |
|  | hsa05225 | Hepatocellular carcinoma | 0.04055479 |
|  | hsa04916 | Melanogenesis | 0.040771262 |
|  | hsa00563 | Glycosylphosphatidylinositol (GPI)-anchor biosynthesis | 0.043199217 |
|  | hsa05224 | Breast cancer | 0.043554829 |
|  | hsa04623 | Cytosolic DNA-sensing pathway | 0.048631823 |
|  | hsa04962 | Vasopressin-regulated water reabsorption | 0.049605426 |
| PBMC | hsa04610 | Complement and coagulation cascades | 8.37E-05 |
|  | hsa04630 | JAK-STAT signaling pathway | 0.000799652 |
|  | hsa04060 | Cytokine-cytokine receptor interaction | 0.002085241 |
|  | hsa05160 | Hepatitis C | 0.005172761 |
|  | hsa04014 | Ras signaling pathway | 0.007239109 |
|  | hsa04640 | Hematopoietic cell lineage | 0.007253188 |
|  | hsa04512 | ECM-receptor interaction | 0.01342268 |
|  | hsa00100 | Steroid biosynthesis | 0.016390229 |
|  | hsa04550 | Signaling pathways regulating pluripotency of stem cells | 0.019939556 |
|  | hsa00480 | Glutathione metabolism | 0.022285506 |
|  | hsa01212 | Fatty acid metabolism | 0.023872576 |
|  | hsa05164 | Influenza A | 0.025343271 |
|  | hsa04964 | Proximal tubule bicarbonate reclamation | 0.027532911 |
|  | hsa00590 | Arachidonic acid metabolism | 0.034920174 |
|  | hsa04068 | FoxO signaling pathway | 0.03572655 |
|  | hsa00790 | Folate biosynthesis | 0.037956468 |
|  | hsa00062 | Fatty acid elongation | 0.041817704 |
|  | hsa05410 | Hypertrophic cardiomyopathy (HCM) | 0.045516745 |
|  | hsa04973 | Carbohydrate digestion and absorption | 0.046008579 |
