# Supplementary material for: Immune cells transcriptome-based drug repositioning for multiple sclerosis
Source: Front Immunol. 2022 Oct 20;13:1020721. doi: 10.3389/fimmu.2022.1020721 (PMC9630342; doi:10.3389/fimmu.2022.1020721)
Supplement: Supplementary Table 6 — Detailed candidate drugs for targeting target pathways obtained from the KEGG database according to the types of CD19+ B cells, CD4+ T cells, pDCs and PBMC. [file Table_6.docx]

| Sample | Pathway ID | Drug |
| --- | --- | --- |
| CD19^+^ B cells | hsa04062 | Bindarit |
|  | hsa04062 | Ancriviroc |
|  | hsa04062 | Tolimidone |
|  | hsa04062 | Vicriviroc maleate |
|  | hsa04062 | Aplaviroc hydrochloride |
|  | hsa04062 | Maraviroc |
|  | hsa04062 | Plerixafor |
|  | hsa04062 | Reparixin |
|  | hsa04062 | Saracatinib |
|  | hsa04062 | Mogamulizumab |
|  | hsa04062 | Carlumab |
|  | hsa04062 | Cenicriviroc |
|  | hsa04062 | Ruxolitinib |
|  | hsa04062 | Tofacitinib |
|  | hsa04062 | Navarixin |
|  | hsa04062 | Baricitinib |
|  | hsa04062 | Elubrixin |
|  | hsa04062 | Vercirnon |
|  | hsa04062 | Danirixin |
|  | hsa04062 | Defactinib |
|  | hsa04062 | Plozalizumab |
|  | hsa04062 | Delgocitinib |
|  | hsa04062 | Nemiralisib |
|  | hsa04062 | Umbralisib |
|  | hsa04062 | Leronlimab |
|  | hsa04062 | Quetmolimab |
|  | hsa04062 | Mavorixafor |
|  | hsa04062 | Lazucirnon |
|  | hsa04062 | Belumosudil |
|  | hsa04062 | Ritlecitinib tosilate |
|  | hsa04062 | Ilacirnon |
|  | hsa04062 | Balixafortide |
|  | hsa04062 | Laduviglusib |
| CD4^+^ T cells | hsa04722 | Begacestat |
|  | hsa04722 | Tarenflurbil |
|  | hsa04722 | Semagacestat |
|  | hsa04722 | Avagacestat |
|  | hsa04722 | Fulranumab |
|  | hsa04722 | Rebastinib |
|  | hsa04722 | Cenegermin |
|  | hsa04722 | Larotrectinib |
|  | hsa04722 | Repotrectinib |
|  | hsa04722 | Pegcantratinib |
|  | hsa04722 | Vodobatinib |
|  | hsa04722 | Edasalonexent |
|  | hsa04728 | Levodopa |
|  | hsa04728 | Cocaine |
|  | hsa04728 | Haloperidol |
|  | hsa04728 | Phloroglucinol |
|  | hsa04728 | Reserpine |
|  | hsa04728 | Ajmaline |
|  | hsa04728 | Carbamazepine |
|  | hsa04728 | Chlorpromazine |
|  | hsa04728 | Clozapine |
|  | hsa04728 | Disopyramide |
|  | hsa04728 | Droperidol |
|  | hsa04728 | Lamotrigine |
|  | hsa04728 | Lidocaine |
|  | hsa04728 | Mazindol |
|  | hsa04728 | Thioridazine |
|  | hsa04728 | Thiothixene |
|  | hsa04728 | Mephenytoin |
|  | hsa04728 | Triflupromazine |
|  | hsa04728 | Methotrimeprazine |
|  | hsa04728 | Risperidone |
|  | hsa04728 | Olanzapine |
|  | hsa04728 | Prochlorperazine |
|  | hsa04728 | Perphenazine |
|  | hsa04728 | Phenytoin |
|  | hsa04728 | Oxcarbazepine |
|  | hsa04728 | Zonisamide |
|  | hsa04728 | Tetracaine |
|  | hsa04728 | Benzocaine |
|  | hsa04728 | Prilocaine |
|  | hsa04728 | Carbidopa |
|  | hsa04728 | Pimozide |
|  | hsa04728 | Sertindole |
|  | hsa04728 | Metoclopramide |
|  | hsa04728 | Dibucaine |
|  | hsa04728 | Pramoxine hydrochloride |
|  | hsa04728 | Riluzole |
|  | hsa04728 | Entacapone |
|  | hsa04728 | Tolcapone |
|  | hsa04728 | Acetophenazine maleate |
|  | hsa04728 | Chlorprothixene |
|  | hsa04728 | Cabergoline |
|  | hsa04728 | Sparteine |
|  | hsa04728 | Flupentixol |
|  | hsa04728 | Spiperone |
|  | hsa04728 | Bromperidol |
|  | hsa04728 | Oxethazaine |
|  | hsa04728 | Aripiprazole |
|  | hsa04728 | Blonanserin |
|  | hsa04728 | Sulpiride |
|  | hsa04728 | Flopropione |
|  | hsa04728 | Zotepine |
|  | hsa04728 | Aprindine hydrochloride |
|  | hsa04728 | Terguride |
|  | hsa04728 | Perazine |
|  | hsa04728 | Nemonapride |
|  | hsa04728 | Propericiazine |
|  | hsa04728 | Levomepromazine |
|  | hsa04728 | Domperidone |
|  | hsa04728 | Benoxinate hydrochloride |
|  | hsa04728 | Safrazine hydrochloride |
|  | hsa04728 | Docarpamine |
|  | hsa04728 | Amphetamine |
|  | hsa04728 | Methamphetamine hydrochloride |
|  | hsa04728 | Loxapine |
|  | hsa04728 | Thiethylperazine |
|  | hsa04728 | Toloxatone |
|  | hsa04728 | Brofaromine |
|  | hsa04728 | Moclobemide |
|  | hsa04728 | Befloxatone |
|  | hsa04728 | Iproniazid |
|  | hsa04728 | Isocarboxazid |
|  | hsa04728 | Cimoxatone |
|  | hsa04728 | Clopenthixol |
|  | hsa04728 | Pipamperone |
|  | hsa04728 | Trifluperidol |
|  | hsa04728 | Benperidol |
|  | hsa04728 | Alentemol hydrobromide |
|  | hsa04728 | Carphenazine maleate |
|  | hsa04728 | Fananserin |
|  | hsa04728 | Iloperidone |
|  | hsa04728 | Mesoridazine |
|  | hsa04728 | Ocaperidone |
|  | hsa04728 | Oxiperomide |
|  | hsa04728 | Tiospirone hydrochloride |
|  | hsa04728 | Talampanel |
|  | hsa04728 | Actisomide |
|  | hsa04728 | Adrogolide hydrochloride |
|  | hsa04728 | Benserazide |
|  | hsa04728 | Brasofensine maleate |
|  | hsa04728 | Bromocriptine |
|  | hsa04728 | Aplindore fumarate |
|  | hsa04728 | Armodafinil |
|  | hsa04728 | Ladostigil tartrate |
|  | hsa04728 | Nitecapone |
|  | hsa04728 | Clorgiline |
|  | hsa04728 | Caroxazone |
|  | hsa04728 | Cifenline |
|  | hsa04728 | Ciladopa hydrochloride |
|  | hsa04728 | Clebopride |
|  | hsa04728 | Zuclopenthixol |
|  | hsa04728 | Selegiline |
|  | hsa04728 | Mofegiline hydrochloride |
|  | hsa04728 | Dextroamphetamine |
|  | hsa04728 | Diethylpropion hydrochloride |
|  | hsa04728 | Dizocilpine maleate |
|  | hsa04728 | Dopexamine |
|  | hsa04728 | Ecopipam hydrochloride |
|  | hsa04728 | Etryptamine acetate |
|  | hsa04728 | Etidocaine |
|  | hsa04728 | Etilevodopa |
|  | hsa04728 | Farampator |
|  | hsa04728 | Gavestinel |
|  | hsa04728 | Ibopamine |
|  | hsa04728 | Indecainide hydrochloride |
|  | hsa04728 | Lazabemide |
|  | hsa04728 | Lergotrile |
|  | hsa04728 | Licostinel |
|  | hsa04728 | Lorajmine hydrochloride |
|  | hsa04728 | Lurasidone hydrochloride |
|  | hsa04728 | Methyldopate hydrochloride |
|  | hsa04728 | Methylphenidate |
|  | hsa04728 | Metopimazine |
|  | hsa04728 | Milacemide hydrochloride |
|  | hsa04728 | Minaprine |
|  | hsa04728 | Napamezole hydrochloride |
|  | hsa04728 | Naxagolide hydrochloride |
|  | hsa04728 | Paliperidone |
|  | hsa04728 | Phenmetrazine hydrochloride |
|  | hsa04728 | Phentermine |
|  | hsa04728 | Pramipexole |
|  | hsa04728 | Pyrovalerone hydrochloride |
|  | hsa04728 | Quinelorane hydrochloride |
|  | hsa04728 | Quinpirole hydrochloride |
|  | hsa04728 | Radafaxine hydrochloride |
|  | hsa04728 | Rotigotine |
|  | hsa04728 | Rufinamide |
|  | hsa04728 | Sarizotan hydrochloride |
|  | hsa04728 | Sibenadet hydrochloride |
|  | hsa04728 | Sonepiprazole mesylate |
|  | hsa04728 | Tocainide |
|  | hsa04728 | Transcainide |
|  | hsa04728 | Traxoprodil mesylate |
|  | hsa04728 | Bifeprunox |
|  | hsa04728 | Tezampanel |
|  | hsa04728 | Acamprosate |
|  | hsa04728 | Acepromazine |
|  | hsa04728 | Bromopride |
|  | hsa04728 | Alizapride |
|  | hsa04728 | Quinagolide |
|  | hsa04728 | Piribedil |
|  | hsa04728 | Budipine |
|  | hsa04728 | Melperone |
|  | hsa04728 | Amisulpride |
|  | hsa04728 | Levosulpiride |
|  | hsa04728 | Captodiame |
|  | hsa04728 | Nialamide |
|  | hsa04728 | Iproclozide |
|  | hsa04728 | Amantadine |
|  | hsa04728 | Amfepramone |
|  | hsa04728 | Amfetamine |
|  | hsa04728 | Apomorphine |
|  | hsa04728 | Articaine |
|  | hsa04728 | Benzfetamine |
|  | hsa04728 | Bupivacaine |
|  | hsa04728 | Bupropion |
|  | hsa04728 | Buspirone |
|  | hsa04728 | Chloroprocaine |
|  | hsa04728 | Dexmethylphenidate |
|  | hsa04728 | Dihydroergocryptine |
|  | hsa04728 | Dopamine |
|  | hsa04728 | Encainide |
|  | hsa04728 | Fenoldopam |
|  | hsa04728 | Flecainide |
|  | hsa04728 | Fluphenazine |
|  | hsa04728 | Fosphenytoin |
|  | hsa04728 | Itopride |
|  | hsa04728 | Levobupivacaine |
|  | hsa04728 | Levomethadone |
|  | hsa04728 | Lisdexamfetamine |
|  | hsa04728 | Lisuride |
|  | hsa04728 | Mepivacaine |
|  | hsa04728 | Metamfetamine |
|  | hsa04728 | Methadone |
|  | hsa04728 | Mexiletine |
|  | hsa04728 | Molindone |
|  | hsa04728 | Mosapramine |
|  | hsa04728 | Opipramol |
|  | hsa04728 | Oxetacaine hydrochloride |
|  | hsa04728 | Oxybuprocaine |
|  | hsa04728 | Pergolide |
|  | hsa04728 | Phenelzine |
|  | hsa04728 | Polyphloroglucinol phosphate |
|  | hsa04728 | Pipotiazine |
|  | hsa04728 | Pirlindole |
|  | hsa04728 | Pirmenol |
|  | hsa04728 | Pramocaine |
|  | hsa04728 | Procainamide |
|  | hsa04728 | Procaine |
|  | hsa04728 | Promazine |
|  | hsa04728 | Propafenone |
|  | hsa04728 | Pargyline |
|  | hsa04728 | Quetiapine |
|  | hsa04728 | Quinidine |
|  | hsa04728 | Rasagiline |
|  | hsa04728 | Ropinirole |
|  | hsa04728 | Ropivacaine |
|  | hsa04728 | Sibutramine |
|  | hsa04728 | Sultopride |
|  | hsa04728 | Talipexole |
|  | hsa04728 | Tetrabenazine |
|  | hsa04728 | Thioproperazine |
|  | hsa04728 | Tiapride |
|  | hsa04728 | Tranylcypromine |
|  | hsa04728 | Trifluoperazine |
|  | hsa04728 | Ziprasidone |
|  | hsa04728 | Perampanel |
|  | hsa04728 | Zonampanel |
|  | hsa04728 | Eslicarbazepine |
|  | hsa04728 | Pardoprunox |
|  | hsa04728 | Mibampator |
|  | hsa04728 | Pridopidine |
|  | hsa04728 | Cariprazine |
|  | hsa04728 | Ioflupane I 123 |
|  | hsa04728 | Amitifadine |
|  | hsa04728 | Dasolampanel |
|  | hsa04728 | Safinamide |
|  | hsa04728 | Brexpiprazole |
|  | hsa04728 | Valbenazine |
|  | hsa04728 | Centanafadine |
|  | hsa04728 | Dasotraline |
|  | hsa04728 | Deutetrabenazine |
|  | hsa04728 | Opicapone |
|  | hsa04728 | Lumateperone |
|  | hsa04728 | Midomafetamine |
|  | hsa04728 | Sembragiline |
|  | hsa04728 | Solriamfetol |
|  | hsa04728 | Asenapine |
|  | hsa04728 | Mevidalen |
|  | hsa04728 | Foslevodopa |
|  | hsa04728 | Trazpiroben |
|  | hsa04728 | Laduviglusib |
|  | hsa05161 | Peginterferon alfa-2a |
|  | hsa05161 | Interferon alfa (NAMALWA) |
|  | hsa04927 | Corticotropin |
|  | hsa04927 | Cosyntropin |
|  | hsa04927 | Tetracosactide acetate |
|  | hsa04927 | Seractide acetate |
|  | hsa04927 | Corticotropin, repository |
|  | hsa04927 | Alsactide |
|  | hsa04927 | Giractide |
|  | hsa04935 | Edotreotide gallium Ga-68 |
|  | hsa04935 | Copper Cu 64 dotatate |
|  | hsa04670 | Alicaforsen sodium |
|  | hsa04670 | Batimastat |
|  | hsa04670 | Ilomastat |
|  | hsa04670 | Marimastat |
|  | hsa04670 | Prinomastat |
|  | hsa04670 | Rebimastat |
|  | hsa04670 | Tanomastat |
|  | hsa04670 | Enlimomab |
|  | hsa04670 | Erlizumab |
|  | hsa04670 | Midostaurin |
|  | hsa04670 | Natalizumab |
|  | hsa04670 | Valategrast hydrochloride |
|  | hsa04670 | Lifitegrast |
|  | hsa04670 | Ripasudil hydrochloride hydrate |
|  | hsa04670 | Defactinib |
|  | hsa04144 | Basiliximab |
|  | hsa04144 | Ancriviroc |
|  | hsa04144 | Daclizumab |
|  | hsa04144 | Denileukin diftitox |
|  | hsa04144 | Vicriviroc maleate |
|  | hsa04144 | Aplaviroc hydrochloride |
|  | hsa04144 | Maraviroc |
|  | hsa04144 | Cenicriviroc |
|  | hsa04144 | Vintafolide |
|  | hsa04144 | Leronlimab |
|  | hsa04144 | Pabinafusp alfa (genetical recombination) |
|  | hsa04144 | Farletuzumab ecteribulin |
|  | hsa04611 | Epoprostenol |
|  | hsa04611 | Pimilprost |
|  | hsa04611 | Trapidil |
|  | hsa04611 | Cilostazol |
|  | hsa04611 | Clinprost |
|  | hsa04611 | Beraprost |
|  | hsa04611 | Iloprost |
|  | hsa04611 | Abciximab |
|  | hsa04611 | Cangrelor |
|  | hsa04611 | Ciprostene calcium |
|  | hsa04611 | Daltroban |
|  | hsa04611 | Dazmegrel |
|  | hsa04611 | Dazoxiben hydrochloride |
|  | hsa04611 | Elarofiban |
|  | hsa04611 | Furegrelate sodium |
|  | hsa04611 | Ifetroban |
|  | hsa04611 | Lamifiban |
|  | hsa04611 | Lotrafiban hydrochloride |
|  | hsa04611 | Orbofiban acetate |
|  | hsa04611 | Pirmagrel |
|  | hsa04611 | Prasugrel hydrochloride |
|  | hsa04611 | Ridogrel |
|  | hsa04611 | Roxifiban acetate |
|  | hsa04611 | Sibrafiban |
|  | hsa04611 | Sulotroban |
|  | hsa04611 | Terbogrel |
|  | hsa04611 | Treprostinil |
|  | hsa04611 | Vapiprost hydrochloride |
|  | hsa04611 | Xemilofiban hydrochloride |
|  | hsa04611 | Giripladib |
|  | hsa04611 | Tadocizumab |
|  | hsa04611 | Eptifibatide |
|  | hsa04611 | Picotamide |
|  | hsa04611 | Indobufen |
|  | hsa04611 | Cinaciguat |
|  | hsa04611 | Ozagrel |
|  | hsa04611 | Ticlopidine |
|  | hsa04611 | Tirofiban |
|  | hsa04611 | Regrelor disodium |
|  | hsa04611 | Ticagrelor |
|  | hsa04611 | Fostamatinib disodium |
|  | hsa04611 | Riociguat |
|  | hsa04611 | Vorapaxar |
|  | hsa04611 | Selexipag |
|  | hsa04611 | Turoctocog alfa pegol (genetical recombination) |
|  | hsa04611 | Acalabrutinib |
|  | hsa04611 | Vericiguat |
|  | hsa04611 | Tirabrutinib hydrochloride |
|  | hsa04611 | Caplacizumab |
|  | hsa04611 | Entospletinib |
|  | hsa04611 | Praliciguat |
|  | hsa04611 | Vecabrutinib |
|  | hsa04611 | Fenebrutinib |
|  | hsa04611 | Olinciguat |
|  | hsa04611 | Branebrutinib |
|  | hsa04611 | Mivavotinib |
|  | hsa04611 | Pirtobrutinib |
|  | hsa04611 | Zalunfiban |
|  | hsa04611 | Luxeptinib |
|  | hsa04611 | Orelabrutinib |
|  | hsa04962 | Vasopressin |
|  | hsa04962 | Desmopressin |
|  | hsa04962 | Lypressin |
|  | hsa04962 | Tolvaptan |
|  | hsa04962 | Mozavaptane hydrochloride |
|  | hsa04962 | Argipressin tannate |
|  | hsa04962 | Lixivaptan |
|  | hsa04962 | Terlipressin |
|  | hsa04962 | Ornipressin |
|  | hsa04962 | Conivaptan |
|  | hsa04062 | Bindarit |
|  | hsa04062 | Ancriviroc |
|  | hsa04062 | Tolimidone |
|  | hsa04062 | Vicriviroc maleate |
|  | hsa04062 | Aplaviroc hydrochloride |
|  | hsa04062 | Maraviroc |
|  | hsa04062 | Plerixafor |
|  | hsa04062 | Reparixin |
|  | hsa04062 | Saracatinib |
|  | hsa04062 | Mogamulizumab |
|  | hsa04062 | Carlumab |
|  | hsa04062 | Cenicriviroc |
|  | hsa04062 | Ruxolitinib |
|  | hsa04062 | Tofacitinib |
|  | hsa04062 | Navarixin |
|  | hsa04062 | Baricitinib |
|  | hsa04062 | Elubrixin |
|  | hsa04062 | Vercirnon |
|  | hsa04062 | Danirixin |
|  | hsa04062 | Defactinib |
|  | hsa04062 | Plozalizumab |
|  | hsa04062 | Delgocitinib |
|  | hsa04062 | Nemiralisib |
|  | hsa04062 | Umbralisib |
|  | hsa04062 | Leronlimab |
|  | hsa04062 | Quetmolimab |
|  | hsa04062 | Mavorixafor |
|  | hsa04062 | Lazucirnon |
|  | hsa04062 | Belumosudil |
|  | hsa04062 | Ritlecitinib tosilate |
|  | hsa04062 | Ilacirnon |
|  | hsa04062 | Balixafortide |
|  | hsa04062 | Laduviglusib |
|  | hsa04151 | Ancestim |
|  | hsa04151 | Binetrakin |
|  | hsa04151 | Filgrastim |
|  | hsa04151 | Lenograstim |
|  | hsa04151 | Mecasermin |
|  | hsa04151 | Fostriecin sodium |
|  | hsa04151 | Lestaurtinib |
|  | hsa04151 | Midostaurin |
|  | hsa04151 | Milodistim |
|  | hsa04151 | Pegvisomant |
|  | hsa04151 | Tandutinib |
|  | hsa04151 | Triciribine phosphate |
|  | hsa04151 | Vatalanib |
|  | hsa04151 | Alvespimycin hydrochloride |
|  | hsa04151 | Tanespimycin |
|  | hsa04151 | Motesanib |
|  | hsa04151 | Pegfilgrastim |
|  | hsa04151 | Tozasertib |
|  | hsa04151 | Toceranib |
|  | hsa04151 | Dovitinib lactate |
|  | hsa04151 | Omacetaxine mepesuccinate |
|  | hsa04151 | Retaspimycin |
|  | hsa04151 | Brivanib |
|  | hsa04151 | Linifanib |
|  | hsa04151 | Cabozantinib |
|  | hsa04151 | Ganetespib |
|  | hsa04151 | Pictilisib |
|  | hsa04151 | Tivozanib hydrochloride |
|  | hsa04151 | Golvatinib |
|  | hsa04151 | Lipegfilgrastim |
|  | hsa04151 | Balugrastim |
|  | hsa04151 | Dupilumab |
|  | hsa04151 | Gandotinib |
|  | hsa04151 | Palbociclib |
|  | hsa04151 | Afuresertib |
|  | hsa04151 | Nesvacumab |
|  | hsa04151 | Apitolisib |
|  | hsa04151 | Dactolisib |
|  | hsa04151 | Duvelisib |
|  | hsa04151 | Idelalisib |
|  | hsa04151 | Buparlisib |
|  | hsa04151 | Gedatolisib |
|  | hsa04151 | Ipatasertib |
|  | hsa04151 | Luminespib |
|  | hsa04151 | Abemaciclib |
|  | hsa04151 | Capmatinib |
|  | hsa04151 | Omipalisib |
|  | hsa04151 | Onalespib |
|  | hsa04151 | Copanlisib |
|  | hsa04151 | Ribociclib |
|  | hsa04151 | Dezapelisib |
|  | hsa04151 | Entrectinib |
|  | hsa04151 | Erdafitinib |
|  | hsa04151 | Istiratumab |
|  | hsa04151 | Itacitinib |
|  | hsa04151 | Naquotinib |
|  | hsa04151 | Alpelisib |
|  | hsa04151 | Pegteograstim |
|  | hsa04151 | Modotuximab |
|  | hsa04151 | Navicixizumab |
|  | hsa04151 | Trilaciclib |
|  | hsa04151 | Sitravatinib |
|  | hsa04151 | Tucatinib |
|  | hsa04151 | Axicabtagene ciloleucel |
|  | hsa04151 | Leniolisib |
|  | hsa04151 | Somavaratan |
|  | hsa04151 | Somapacitan |
|  | hsa04151 | Entospletinib |
|  | hsa04151 | Nemiralisib |
|  | hsa04151 | Pexidartinib |
|  | hsa04151 | Rivoceranib |
|  | hsa04151 | Umbralisib |
|  | hsa04151 | Loncastuximab |
|  | hsa04151 | Ripretinib |
|  | hsa04151 | Capivasertib |
|  | hsa04151 | Miransertib |
|  | hsa04151 | Pemigatinib |
|  | hsa04151 | Parsaclisib |
|  | hsa04151 | Bimiralisib |
|  | hsa04151 | Lerociclib |
|  | hsa04151 | Derazantinib hydrochloride |
|  | hsa04151 | Lonapegsomatropin |
|  | hsa04151 | Samotolisib |
|  | hsa04151 | Razuprotafib |
|  | hsa04151 | Zotiraciclib |
|  | hsa04151 | Tafasitamab |
|  | hsa04151 | Dilpacimab |
|  | hsa04151 | Gusacitinib |
|  | hsa04151 | Tepotinib |
|  | hsa04151 | Voxtalisib |
|  | hsa04151 | Duligotuzumab |
|  | hsa04151 | Inebilizumab |
|  | hsa04151 | Lucitanib |
|  | hsa04151 | Pacritinib |
|  | hsa04151 | Tesevatinib |
|  | hsa04151 | Taselisib |
|  | hsa04151 | Vixarelimab |
|  | hsa04151 | Paxalisib |
|  | hsa04151 | Brexucabtagene autoleucel |
|  | hsa04151 | Surufatinib |
|  | hsa04151 | Amivantamab |
|  | hsa04151 | Eganelisib |
|  | hsa04151 | Pimitespib |
|  | hsa04151 | Inavolisib |
|  | hsa04151 | Murizatoclax |
|  | hsa04151 | Tapotoclax |
|  | hsa04151 | Fruquintinib |
|  | hsa04151 | Efbemalenograstim alfa |
|  | hsa04151 | Lisocabtagene maraleucel |
|  | hsa04151 | Zenocutuzumab |
|  | hsa04151 | Zandelisib |
|  | hsa04151 | Sotorasib |
|  | hsa04151 | Patritumab deruxtecan |
| pDCs | hsa04962 | Vasopressin |
|  | hsa04962 | Desmopressin |
|  | hsa04962 | Lypressin |
|  | hsa04962 | Tolvaptan |
|  | hsa04962 | Mozavaptane hydrochloride |
|  | hsa04962 | Argipressin tannate |
|  | hsa04962 | Lixivaptan |
|  | hsa04962 | Terlipressin |
|  | hsa04962 | Ornipressin |
|  | hsa04962 | Conivaptan |
|  | hsa04621 | Cinacalcet |
|  | hsa04621 | Mifamurtide |
|  | hsa04621 | Pralnacasan |
|  | hsa04621 | Canakinumab |
|  | hsa04621 | Gevokizumab |
|  | hsa04621 | Belnacasan |
|  | hsa04621 | Etelcalcetide |
|  | hsa04621 | Evocalcet |
|  | hsa04621 | Inarigivir soproxil |
|  | hsa04621 | Upacicalcet sodium hydrate |
|  | hsa04621 | Deucravacitinib |
|  | hsa05216 | Vandetanib |
|  | hsa05216 | Sorafenib |
|  | hsa05216 | Lenvatinib |
|  | hsa05216 | Selpercatinib |
|  | hsa05162 | Interferon alfa (NAMALWA) |
| PBMC | hsa04610 | Aminocaproic acid |
|  | hsa04610 | Argatroban |
|  | hsa04610 | Desmopressin |
|  | hsa04610 | Tranexamic acid |
|  | hsa04610 | Fondaparinux sodium |
|  | hsa04610 | Ximelagatran |
|  | hsa04610 | Heparin |
|  | hsa04610 | Anticoagulant heparin |
|  | hsa04610 | Bivalirudin |
|  | hsa04610 | Apixaban |
|  | hsa04610 | Kallidinogenase |
|  | hsa04610 | Reviparin sodium |
|  | hsa04610 | Dalteparin sodium |
|  | hsa04610 | Danaparoid sodium |
|  | hsa04610 | Deligoparin sodium |
|  | hsa04610 | Enoxaparin sodium |
|  | hsa04610 | Deltibant |
|  | hsa04610 | Desirudin |
|  | hsa04610 | Efegatran sulfate |
|  | hsa04610 | Napsagatran |
|  | hsa04610 | Diaplasinin |
|  | hsa04610 | Ecallantide |
|  | hsa04610 | Eculizumab |
|  | hsa04610 | Razaxaban hydrochloride |
|  | hsa04610 | Fidexaban |
|  | hsa04610 | Icatibant acetate |
|  | hsa04610 | Parnaparin sodium |
|  | hsa04610 | Tifacogin |
|  | hsa04610 | Tinzaparin sodium |
|  | hsa04610 | Thrombomodulin alfa |
|  | hsa04610 | Lepirudin |
|  | hsa04610 | Rivaroxaban |
|  | hsa04610 | Melagatran |
|  | hsa04610 | Adomiparin |
|  | hsa04610 | Aminomethylbenzoic acid |
|  | hsa04610 | Gabexate |
|  | hsa04610 | Sulodexide |
|  | hsa04610 | Aleplasinin |
|  | hsa04610 | Betrixaban |
|  | hsa04610 | Eribaxaban |
|  | hsa04610 | Dabigatran |
|  | hsa04610 | Edoxaban |
|  | hsa04610 | Vorapaxar |
|  | hsa04610 | Darexaban maleate |
|  | hsa04610 | Atopaxar |
|  | hsa04610 | Letaxaban |
|  | hsa04610 | Delparantag pentahydrochloride |
|  | hsa04610 | Idraparinux sodium |
|  | hsa04610 | Pegnivacogin |
|  | hsa04610 | Turoctocog alfa pegol (genetical recombination) |
|  | hsa04610 | Emicizumab |
|  | hsa04610 | Conestat alfa |
|  | hsa04610 | Andexanet alfa |
|  | hsa04610 | Ravulizumab |
|  | hsa04610 | Avacopan |
|  | hsa04610 | Lanadelumab |
|  | hsa04610 | Caplacizumab |
|  | hsa04610 | Marstacimab |
|  | hsa04610 | Olendalizumab |
|  | hsa04610 | Nomacopan |
|  | hsa04610 | Pozelimab |
|  | hsa04610 | Sutimlimab |
|  | hsa04610 | Narsoplimab |
|  | hsa04610 | Pegcetacoplan |
|  | hsa04610 | Danicopan |
|  | hsa04610 | Berotralstat |
|  | hsa04610 | Crovalimab |
|  | hsa04610 | Avacincaptad pegol sodium |
|  | hsa04610 | Milvexian |
|  | hsa04610 | Fitusiran sodium |
|  | hsa04610 | Tisotumab |
|  | hsa04610 | Vilobelimab |
|  | hsa04610 | Concizumab |
|  | hsa04610 | alpha 1-Antitrypsin |
|  | hsa04610 | Plasminogen |
|  | hsa05160 | Interferon alfa-2b |
|  | hsa05160 | Peginterferon alfa-2a |
|  | hsa05160 | Peginterferon alfa-2b |
|  | hsa05160 | Interferon beta |
|  | hsa05160 | Interferon alfa (NAMALWA) |
|  | hsa00590 | Aspirin |
|  | hsa00590 | Naproxen |
|  | hsa00590 | Sulindac |
|  | hsa00590 | Ibuprofen |
|  | hsa00590 | Piroxicam |
|  | hsa00590 | Diflunisal |
|  | hsa00590 | Ketoprofen |
|  | hsa00590 | Indomethacin |
|  | hsa00590 | Mefenamic acid |
|  | hsa00590 | Meclofenamate sodium |
|  | hsa00590 | Acetaminophen |
|  | hsa00590 | Etodolac |
|  | hsa00590 | Flurbiprofen |
|  | hsa00590 | Zileuton |
|  | hsa00590 | Nabumetone |
|  | hsa00590 | Salsalate |
|  | hsa00590 | Oxaprozin |
|  | hsa00590 | Phenylbutazone |
|  | hsa00590 | Sodium salicylate |
|  | hsa00590 | Celecoxib |
|  | hsa00590 | Rofecoxib |
|  | hsa00590 | Choline salicylate |
|  | hsa00590 | Magnesium salicylate |
|  | hsa00590 | Meloxicam |
|  | hsa00590 | Nimesulide |
|  | hsa00590 | Tolfenamic acid |
|  | hsa00590 | Alclofenac |
|  | hsa00590 | Bufexamac |
|  | hsa00590 | Tiaprofenic acid |
|  | hsa00590 | Fenbufen |
|  | hsa00590 | Ampiroxicam |
|  | hsa00590 | Alminoprofen |
|  | hsa00590 | Epidihydrocholesterin |
|  | hsa00590 | Aceclofenac |
|  | hsa00590 | Zaltoprofen |
|  | hsa00590 | Glycol salicylate |
|  | hsa00590 | Indometacin farnesil |
|  | hsa00590 | Pranoprofen |
|  | hsa00590 | Flufenamic acid |
|  | hsa00590 | Acemetacin |
|  | hsa00590 | Felbinac |
|  | hsa00590 | Mofezolac |
|  | hsa00590 | Tenoxicam |
|  | hsa00590 | Oxatomide |
|  | hsa00590 | Salicylamide |
|  | hsa00590 | Ufenamate |
|  | hsa00590 | Lornoxicam |
|  | hsa00590 | Tilmacoxib |
|  | hsa00590 | Meclofenamic acid |
|  | hsa00590 | Fenoprofen |
|  | hsa00590 | Tolmetin |
|  | hsa00590 | Valdecoxib |
|  | hsa00590 | Acivicin |
|  | hsa00590 | Atreleuton |
|  | hsa00590 | Benoxaprofen |
|  | hsa00590 | Flufenamate aluminum |
|  | hsa00590 | Darbufelone mesylate |
|  | hsa00590 | Dazmegrel |
|  | hsa00590 | Dazoxiben hydrochloride |
|  | hsa00590 | Deracoxib |
|  | hsa00590 | Etoricoxib |
|  | hsa00590 | Firocoxib |
|  | hsa00590 | Lumiracoxib |
|  | hsa00590 | Dexibuprofen |
|  | hsa00590 | Parecoxib |
|  | hsa00590 | Docebenone |
|  | hsa00590 | Ecopladib |
|  | hsa00590 | Enazadrem phosphate |
|  | hsa00590 | Etofenamate |
|  | hsa00590 | Fenleuton |
|  | hsa00590 | Furegrelate sodium |
|  | hsa00590 | Lonapalene |
|  | hsa00590 | Mavacoxib |
|  | hsa00590 | Nepafenac |
|  | hsa00590 | Orpanoxin |
|  | hsa00590 | Phenbutazone sodium glycerate |
|  | hsa00590 | Piriprost |
|  | hsa00590 | Pirmagrel |
|  | hsa00590 | Pravadoline maleate |
|  | hsa00590 | Ridogrel |
|  | hsa00590 | Salicylate meglumine |
|  | hsa00590 | Tebufelone |
|  | hsa00590 | Tenidap |
|  | hsa00590 | Tepoxalin |
|  | hsa00590 | Zidometacin |
|  | hsa00590 | Giripladib |
|  | hsa00590 | Efipladib |
|  | hsa00590 | Indobufen |
|  | hsa00590 | Triflusal |
|  | hsa00590 | Droxicam |
|  | hsa00590 | Ibuproxam |
|  | hsa00590 | Dexketoprofen |
|  | hsa00590 | Propacetamol |
|  | hsa00590 | Aloxiprin |
|  | hsa00590 | Amfenac |
|  | hsa00590 | Amtolmetin guacil |
|  | hsa00590 | Benzydamine |
|  | hsa00590 | Bromfenac |
|  | hsa00590 | Diclofenac |
|  | hsa00590 | Eltenac |
|  | hsa00590 | Ketorolac |
|  | hsa00590 | Loxoprofen |
|  | hsa00590 | Niflumic acid |
|  | hsa00590 | Oxyphenbutazone |
|  | hsa00590 | Ozagrel |
|  | hsa00590 | Piketoprofen |
|  | hsa00590 | Proglumetacin |
|  | hsa00590 | Trolamine salicylate |
|  | hsa00590 | Apricoxib |
|  | hsa00590 | Naproxcinod |
|  | hsa00590 | Setileuton |
|  | hsa00590 | Esflurbiprofen |
|  | hsa00590 | Polmacoxib |
|  | hsa04640 | Thalidomide |
|  | hsa04640 | Pirfenidone |
|  | hsa04640 | Tocilizumab |
|  | hsa04640 | Mirimostim |
|  | hsa04640 | Abciximab |
|  | hsa04640 | Ancestim |
|  | hsa04640 | Rituximab |
|  | hsa04640 | Basiliximab |
|  | hsa04640 | Epoetin alfa |
|  | hsa04640 | Epoetin beta |
|  | hsa04640 | Filgrastim |
|  | hsa04640 | Lenograstim |
|  | hsa04640 | Gemtuzumab ozogamicin |
|  | hsa04640 | Cilengitide |
|  | hsa04640 | Daclizumab |
|  | hsa04640 | Daniplestim |
|  | hsa04640 | Darbepoetin alfa |
|  | hsa04640 | Elarofiban |
|  | hsa04640 | Epoetin delta |
|  | hsa04640 | Epratuzumab |
|  | hsa04640 | Ibritumomab tiuxetan |
|  | hsa04640 | Lamifiban |
|  | hsa04640 | Lenalidomide |
|  | hsa04640 | Leridistim |
|  | hsa04640 | Lotrafiban hydrochloride |
|  | hsa04640 | Mepolizumab |
|  | hsa04640 | Milodistim |
|  | hsa04640 | Molgramostim |
|  | hsa04640 | Muplestim |
|  | hsa04640 | Ocrelizumab |
|  | hsa04640 | Oprelvekin |
|  | hsa04640 | Orbofiban acetate |
|  | hsa04640 | Roxifiban acetate |
|  | hsa04640 | Sargramostim |
|  | hsa04640 | Sibrafiban |
|  | hsa04640 | Siplizumab |
|  | hsa04640 | Xemilofiban hydrochloride |
|  | hsa04640 | Yttrium Y 90 epratuzumab |
|  | hsa04640 | Yttrium Y 90 epratuzumab tetraxetan |
|  | hsa04640 | Zolimomab aritox |
|  | hsa04640 | Lumiliximab |
|  | hsa04640 | Tadocizumab |
|  | hsa04640 | Eptifibatide |
|  | hsa04640 | Pegfilgrastim |
|  | hsa04640 | Tirofiban |
|  | hsa04640 | Tositumomab |
|  | hsa04640 | Inotuzumab ozogamicin |
|  | hsa04640 | Pomalidomide |
|  | hsa04640 | Veltuzumab |
|  | hsa04640 | Anti-human thymocyte immunoglobulin, rabbit |
|  | hsa04640 | Catumaxomab |
|  | hsa04640 | Ofatumumab |
|  | hsa04640 | Obinutuzumab |
|  | hsa04640 | Moxetumomab pasudotox |
|  | hsa04640 | Quizartinib |
|  | hsa04640 | Epoetin epsilon |
|  | hsa04640 | Tosedostat |
|  | hsa04640 | Valategrast hydrochloride |
|  | hsa04640 | Sacubitril |
|  | hsa04640 | Lipegfilgrastim |
|  | hsa04640 | Balugrastim |
|  | hsa04640 | Dupilumab |
|  | hsa04640 | Daratumumab |
|  | hsa04640 | Gilteritinib fumarate |
|  | hsa04640 | Epoetin theta |
|  | hsa04640 | Epoetin zeta |
|  | hsa04640 | Tucidinostat |
|  | hsa04640 | Pegteograstim |
|  | hsa04640 | Isatuximab |
|  | hsa04640 | Axicabtagene ciloleucel |
|  | hsa04640 | Denintuzumab |
|  | hsa04640 | Lenzilumab |
|  | hsa04640 | Ublituximab |
|  | hsa04640 | Vadastuximab |
|  | hsa04640 | Flotetuzumab |
|  | hsa04640 | Camidanlumab |
|  | hsa04640 | Loncastuximab |
|  | hsa04640 | Tagraxofusp |
|  | hsa04640 | Duvortuxizumab |
|  | hsa04640 | Tisagenlecleucel-T |
|  | hsa04640 | Mosunetuzumab |
|  | hsa04640 | Cibisatamab |
|  | hsa04640 | Odronextamab |
|  | hsa04640 | Zotiraciclib |
|  | hsa04640 | Tafasitamab |
|  | hsa04640 | Plamotamab |
|  | hsa04640 | Inebilizumab |
|  | hsa04640 | Pabinafusp alfa (genetical recombination) |
|  | hsa04640 | Glofitamab |
|  | hsa04640 | Vibecotamab |
|  | hsa04640 | Brexucabtagene autotemcel |
|  | hsa04640 | Betibeglogene autotemcel |
|  | hsa04640 | Mezagitamab |
|  | hsa04640 | Efbemalenograstim alfa |
|  | hsa04640 | Lisocabtagene maraleucel |
|  | hsa04640 | Pavurutamab |
|  | hsa04640 | Zalunfiban |
|  | hsa04640 | Elranatamab |
|  | hsa04640 | Lilotomab |
|  | hsa04640 | Lutetium (177Lu) lilotomab satetraxetan |
|  | hsa04640 | Emerfetamab |
|  | hsa04640 | Eluvixtamab |
|  | hsa04640 | Teclistamab |
|  | hsa04640 | Talquetamab |
|  | hsa00480 | Hydroxyurea |
|  | hsa00480 | Acivicin |
|  | hsa00480 | Eflornithine |
|  | hsa00480 | Ezatiostat hydrochloride |
|  | hsa01212 | Oxfenicine |
|  | hsa01212 | Olumacostat glasaretil |
|  | hsa01212 | Firsocostat |
|  | hsa01212 | Clesacostat |
