# Supplementary material for: Immune cells transcriptome-based drug repositioning for multiple sclerosis
Source: Front Immunol. 2022 Oct 20;13:1020721. doi: 10.3389/fimmu.2022.1020721 (PMC9630342; doi:10.3389/fimmu.2022.1020721)
Supplement: Supplementary Table 7 — Detailed candidate drugs for targeting 2 target pathways obtained from the KEGG database according to the types of CD19+ B cells, CD4+ T cells, pDCs and PBMC. [file Table_7.docx]

| Drug | Target pathway | Effect | Sample |
| --- | --- | --- | --- |
| Laduviglusib | Chemokine signaling pathway, Dopaminergic synapse | Glycogen synthase kinase 3 inhibitor | CD19^+^ B cells, CD4^+^ T cells |
| Defactinib | Chemokine signaling pathway, Leukocyte transendothelial migration | Antineoplastic, Focal adhesion kinase inhibitor | CD19^+^ B cells, CD4^+^ T cells |
| Ancriviroc | Chemokine signaling pathway, Endocytosis | Antiviral, CCR5 antagonist | CD19^+^ B cells, CD4^+^ T cells |
| Vicriviroc maleate | Chemokine signaling pathway, Endocytosis | Antiviral, CCR5 antagonist | CD19^+^ B cells, CD4^+^ T cells |
| Aplaviroc hydrochloride | Chemokine signaling pathway, Endocytosis | Antiviral, CCR5 antagonist | CD19^+^ B cells, CD4^+^ T cells |
| Maraviroc | Chemokine signaling pathway, Endocytosis | Antiviral, CCR5 antagonist | CD19^+^ B cells, CD4^+^ T cells |
| Cenicriviroc | Chemokine signaling pathway, Endocytosis | Antiviral, C-C chemokine receptor (CCR) antagonist | CD19^+^ B cells, CD4^+^ T cells |
| Leronlimab | Chemokine signaling pathway, Endocytosis | Antiviral, Anti-CCR5 antibody | CD19^+^ B cells, CD4^+^ T cells |
| Nemiralisib | Chemokine signaling pathway, PI3K-Akt signaling pathway | Antiasthmatic, Anti-inflammatory, Phosphatidylinositol 3-kinase inhibitor | CD19^+^ B cells, CD4^+^ T cells |
| Umbralisib | Chemokine signaling pathway, PI3K-Akt signaling pathway | Antineoplastic | CD19^+^ B cells, CD4^+^ T cells |
| Basiliximab | Endocytosis, Hematopoietic cell lineage | Immunosuppressant, Anti-CD25 antibody | CD4^+^T cells, PBMC |
| Daclizumab | Endocytosis, Hematopoietic cell lineage | Immunosuppressant, Anti-CD25 antibody | CD4^+^T cells, PBMC |
| Abciximab | Platelet activation, Hematopoietic cell lineage | Platelet aggregation inhibitor, Glycoprotein IIb/IIIa receptor antagonist | CD4^+^T cells, PBMC |
| Elarofiban | Platelet activation, Hematopoietic cell lineage | Platelet aggregation inhibitor, Glycoprotein IIb/IIIa receptor antagonist | CD4^+^T cells, PBMC |
| Xemilofiban hydrochloride | Platelet activation, Hematopoietic cell lineage | Platelet aggregation inhibitor, Glycoprotein IIb/IIIa receptor antagonist | CD4^+^T cells, PBMC |
| Eptifibatide | Platelet activation, Hematopoietic cell lineage | Platelet aggregation inhibitor, Glycoprotein IIb/IIIa receptor antagonist | CD4^+^T cells, PBMC |
| Tirofiban | Platelet activation, Hematopoietic cell lineage | Platelet aggregation inhibitor, Glycoprotein IIb/IIIa receptor antagonist | CD4^+^T cells, PBMC |
| Zalunfiban | Platelet activation, Hematopoietic cell lineage | Platelet aggregation inhibitor, Glycoprotein IIb/IIIa receptor antagonist | CD4^+^T cells, PBMC |
| Lamifiban | Platelet activation, Hematopoietic cell lineage | Antithrombotic, Platelet aggregation inhibitor, Glycoprotein IIb/IIIa receptor antagonist | CD4^+^T cells, PBMC |
| Lotrafiban hydrochloride | Platelet activation, Hematopoietic cell lineage | Antithrombotic, Platelet aggregation inhibitor, Glycoprotein IIb/IIIa receptor antagonist | CD4^+^T cells, PBMC |
| Orbofiban acetate | Platelet activation, Hematopoietic cell lineage | Antithrombotic, Platelet aggregation inhibitor, Glycoprotein IIb/IIIa receptor antagonist | CD4^+^T cells, PBMC |
| Roxifiban acetate | Platelet activation, Hematopoietic cell lineage | Antithrombotic, Platelet aggregation inhibitor, Glycoprotein IIb/IIIa receptor antagonist | CD4^+^T cells, PBMC |
| Sibrafiban | Platelet activation, Hematopoietic cell lineage | Antithrombotic, Platelet aggregation inhibitor, Glycoprotein IIb/IIIa receptor antagonist | CD4^+^T cells, PBMC |
| Tadocizumab | Platelet activation, Hematopoietic cell lineage | Platelet aggregation inhibitor, Anti-glycoprotein IIb/IIIa antibody | CD4^+^T cells, PBMC |
| Dazoxiben hydrochloride | Platelet activation, Arachidonic acid metabolism | Antithrombotic, Thromboxane A2 (TXA2) synthase inhibitor | CD4^+^T cells, PBMC |
| Furegrelate sodium | Platelet activation, Arachidonic acid metabolism | Platelet aggregation inhibitor, Thromboxane A2 (TXA2) synthase inhibitor | CD4^+^T cells, PBMC |
| Dazmegrel | Platelet activation, Arachidonic acid metabolism | Platelet aggregation inhibitor, Thromboxane A2 (TXA2) synthase inhibitor | CD4^+^T cells, PBMC |
| Pirmagrel | Platelet activation, Arachidonic acid metabolism | Platelet aggregation inhibitor, Thromboxane A2 (TXA2) synthase inhibitor | CD4^+^T cells, PBMC |
| Ozagrel | Platelet activation, Arachidonic acid metabolism | Anticoagulant, Platelet aggregation inhibitor, Thromboxane A2 (TXA2) synthase inhibitor | CD4^+^T cells, PBMC |
| Ridogrel | Platelet activation, Arachidonic acid metabolism | Antithrombotic, Thromboxane A2 (TXA2) receptor antagonist, Thromboxane synthetase inhibitor | CD4^+^T cells, PBMC |
| Giripladib | Platelet activation, Arachidonic acid metabolism | Analgesic, Anti-inflammatory, Cytosolic phospholipase A2-alpha inhibitor | CD4^+^T cells, PBMC |
| Indobufen | Platelet activation, Arachidonic acid metabolism | Anticoagulant, Anti-inflammatory, Platelet aggregation inhibitor, COX inhibitor | CD4^+^T cells, PBMC |
| Vorapaxar | Platelet activation, Complement and coagulation cascades | Platelet aggregation inhibitor, Coagulation factor II receptor (PAR1) antagonist | CD4^+^T cells, PBMC |
| Caplacizumab | Platelet activation, Complement and coagulation cascades | Platelet aggregation inhibitor, Anti-vWF antibody | CD4^+^T cells, PBMC |
| Entospletinib | Platelet activation, PI3K-Akt signaling pathway | Antineoplastic, Tyrosine kinase inhibitor | CD4^+^T cells, |
| Desmopressin | Vasopressin-regulated water reabsorption, Complement and coagulation cascades | Antidiuretic, Arginine vasopressin receptor agonist | CD4^+^T cells, PBMC |
| Ancestim | PI3K-Akt signaling pathway, Hematopoietic cell lineage | Hematopoietic adjuvant (stem cell factor) | CD4^+^T cells, PBMC |
| Filgrastim | PI3K-Akt signaling pathway, Hematopoietic cell lineage | Antineutropenic, Hematopoietic stimulant, Antineoplastic (enhancer), Granulocyte colony stimulating factor (G-CSF) | CD4^+^T cells, PBMC |
| Lenograstim | PI3K-Akt signaling pathway, Hematopoietic cell lineage | Antineutropenic, Hematopoietic stimulant, Granulocyte colony stimulating factor (G-CSF) | CD4^+^T cells, PBMC |
| Pegfilgrastim | PI3K-Akt signaling pathway, Hematopoietic cell lineage | Antineutropenic, Hematopoietic stimulant, Granulocyte colony stimulating factor (G-CSF) | CD4^+^T cells, PBMC |
| Lipegfilgrastim | PI3K-Akt signaling pathway, Hematopoietic cell lineage | Antineutropenic, Hematopoietic stimulant, Granulocyte colony stimulating factor (G-CSF) | CD4^+^T cells, PBMC |
| Milodistim | PI3K-Akt signaling pathway, Hematopoietic cell lineage | Antineutropenic, Hematopoietic stimulant | CD4^+^T cells, PBMC |
| Balugrastim | PI3K-Akt signaling pathway, Hematopoietic cell lineage | Antineutropenic, Granulocyte colony stimulating factor (G-CSF) | CD4^+^T cells, PBMC |
| Pegteograstim | PI3K-Akt signaling pathway, Hematopoietic cell lineage | Antineutropenic, Granulocyte colony stimulating factor (G-CSF) | CD4^+^T cells, PBMC |
| Efbemalenograstim alfa | PI3K-Akt signaling pathway, Hematopoietic cell lineage | Antineutropenic, Granulocyte colony stimulating factor (G-CSF) | CD4^+^T cells, PBMC |
| Axicabtagene ciloleucel | PI3K-Akt signaling pathway, Hematopoietic cell lineage | Antineoplastic, Anti-CD19 CAR-T cell | CD4^+^T cells, PBMC |
| Brexucabtagene autoleucel | PI3K-Akt signaling pathway, Hematopoietic cell lineage | Antineoplastic, Anti-CD19 CAR-T cell | CD4^+^T cells, PBMC |
| Lisocabtagene maraleucel | PI3K-Akt signaling pathway, Hematopoietic cell lineage | Antineoplastic, Anti-CD19 CAR-T cell | CD4^+^T cells, PBMC |
| Zotiraciclib | PI3K-Akt signaling pathway, Hematopoietic cell lineage | Antineoplastic | CD4^+^T cells, PBMC |
| Tafasitamab | PI3K-Akt signaling pathway, Hematopoietic cell lineage | Antineoplastic, Anti-CD19 antibody | CD4^+^T cells, PBMC |
| Inebilizumab | PI3K-Akt signaling pathway, Hematopoietic cell lineage | Antineoplastic, Anti-CD19 antibody | CD4^+^T cells, PBMC |
| Loncastuximab | PI3K-Akt signaling pathway, Hematopoietic cell lineage | Antineoplastic, Anti-CD19 antibody | CD4^+^T cells, PBMC |
| Dupilumab | PI3K-Akt signaling pathway, Hematopoietic cell lineage | Anti-inflammatory, Anti-IL-4/IL-13 antibody | CD4^+^T cells, PBMC |
| Peginterferon alfa-2a | Hepatitis B, Hepatitis C | Antiviral, Biological response modifier | CD4^+^T cells, PBMC |
| Interferon alfa (NAMALWA) | Hepatitis B, Hepatitis C | Antineoplastic, Antiviral, Biological response modifier | CD4^+^T cells, PBMC |
| Valategrast hydrochloride | Leukocyte transendothelial migration, Hematopoietic cell lineage | Antiasthmatic | CD4^+^T cells, PBMC |
| Midostaurin | Leukocyte transendothelial migration, PI3K-Akt signaling pathway | Antineoplastic, Receptor tyrosine kinase inhibitor | CD4^+^ T cells |
| Acivicin | Arachidonic acid metabolism, Glutathione metabolism | Antineoplastic, Antimetabolite | PBMC |
