# Supplementary material for: Immune cells transcriptome-based drug repositioning for multiple sclerosis
Source: Front Immunol. 2022 Oct 20;13:1020721. doi: 10.3389/fimmu.2022.1020721 (PMC9630342; doi:10.3389/fimmu.2022.1020721)
Supplement: Supplementary Table 8 — The genes differentially expressed in CD4+ T cells of MS patients without treatment at both mRNA and protein levels. [file Table_8.docx]

| Sample | gene |
| --- | --- |
| CD4^+^ T cells | TES |
|  | GABPA |
|  | ARF6 |
|  | VCL |
|  | TYMP |
|  | LIMS1 |
|  | YWHAG |
|  | RAD21 |
|  | EDC4 |
|  | ATP6V1A |
|  | SPN |
|  | PDIA3 |
|  | ATP2A2 |
|  | NFKB2 |
|  | TRIM28 |
|  | AAK1 |
|  | TPM4 |
|  | BCKDHB |
|  | CAMK2D |
|  | BCL11B |
|  | FAM114A2 |
|  | LTA4H |
|  | CLTC |
|  | AP2A2 |
