# Supplementary material for: Immune cells transcriptome-based drug repositioning for multiple sclerosis
Source: Front Immunol. 2022 Oct 20;13:1020721. doi: 10.3389/fimmu.2022.1020721 (PMC9630342; doi:10.3389/fimmu.2022.1020721)
Supplement: Supplementary Table 9 — \The pathways that were enriched by genes differentially expressed in CD4+ T cells of MS patients without treatment at both mRNA and protein levels. [file Table_9.docx]

| Sample | Pathway ID | Pathway Name | P value |
| --- | --- | --- | --- |
| CD4^+^ T cells | hsa04721 | Synaptic vesicle cycle | 0.000617229 |
|  | hsa04261 | Adrenergic signaling in cardiomyocytes | 0.004049892 |
|  | hsa04961 | Endocrine and other factor-regulated calcium reabsorption | 0.005918386 |
|  | hsa05100 | Bacterial invasion of epithelial cells | 0.01218045 |
|  | hsa04260 | Cardiac muscle contraction | 0.015372064 |
|  | hsa05410 | Hypertrophic cardiomyopathy | 0.016392851 |
|  | hsa04144 | Endocytosis | 0.016642259 |
|  | hsa05414 | Dilated cardiomyopathy | 0.018519664 |
|  | hsa04110 | Cell cycle | 0.030762757 |
|  | hsa04114 | Oocyte meiosis | 0.033048205 |
