# Supplementary material for: Immune cells transcriptome-based drug repositioning for multiple sclerosis
Source: Front Immunol. 2022 Oct 20;13:1020721. doi: 10.3389/fimmu.2022.1020721 (PMC9630342; doi:10.3389/fimmu.2022.1020721)
Supplement: Supplementary Table 10 — The DEGs of MS patients and healthy individuals (FC > 2). [file Table_10.docx]

| ID | Disease | Platform ID | Case/ control | Sample | Publish time | DEG | |
| --- | --- | --- | --- | --- | --- | --- | --- |
|  |  |  |  |  |  | Up-regulated DEG | Down-regulated DEG |
| GSE117935 | RRMS | GPL5175 | 10/10 | CD19^+^ B cells | 2018 | 1 | 1 |
| GSE172009 | RRMS | GPL20301  GPL24676 | 4/4 | CD4^+^ T cells | 2021 | 91 | 1561 |
| GSE37750 | RRMS | GPL570 | 9/8 | pDCs | 2015 | 35 | 30 |
| GSE41890 | RRMS | GPL6244 | 8/4 | Peripheral blood leukocytes | 2013 | 34 | 2 |
