# Supplementary material for: Immune cells transcriptome-based drug repositioning for multiple sclerosis
Source: Front Immunol. 2022 Oct 20;13:1020721. doi: 10.3389/fimmu.2022.1020721 (PMC9630342; doi:10.3389/fimmu.2022.1020721)
Supplement: Supplementary Table 11 — The DEGs of MS patients before and after the application of IFN-β or Fingolimod (FC > 2). [file Table_11.docx]

| ID | Disease | Platform ID | Drug | Case/ control | Sample | Publish time | DEG | |
| --- | --- | --- | --- | --- | --- | --- | --- | --- |
|  |  |  |  |  |  |  | Up-regulated DEG | Down-regulated DEG |
| GSE81604 | RRMS | GPL17586 | Fingolimod | 5/5 | CD19^+^ B cells | 2016 | 11 | 0 |
| GSE73079 | RRMS | GPL17586 | Fingolimod | 5/5 | CD4^+^ T cells | 2015 | 367 | 25 |
| GSE37750 | RRMS | GPL570 | IFN-β | 9/9 | pDCs | 2015 | 43 | 2 |
| GSE33464 | RRMS | GPL14837 | IFN-β | 12/12 | PBMC | 2011 | 70 | 48 |
