# Supplementary material for: Immune cells transcriptome-based drug repositioning for multiple sclerosis
Source: Front Immunol. 2022 Oct 20;13:1020721. doi: 10.3389/fimmu.2022.1020721 (PMC9630342; doi:10.3389/fimmu.2022.1020721)
Supplement: Supplementary Table 12 — The target pathways of Fingolimod and IFN-β for MS treatment (FC > 2). [file Table_12.docx]

| Sample | Pathway ID | Pathway Name |
| --- | --- | --- |
| CD4^+^ T cells | hsa05202 | Transcriptional misregulation in cancer |
|  | hsa05322 | Systemic lupus erythematosus |
|  | hsa04613 | Neutrophil extracellular trap formation |
|  | hsa05120 | Epithelial cell signaling in Helicobacter pylori infection |
|  | hsa05135 | Yersinia infection |
|  | hsa05166 | Human T-cell leukemia virus 1 infection |
|  | hsa04625 | C-type lectin receptor signaling pathway |
| pDCs | hsa05142 | Chagas disease |
|  | hsa05171 | Coronavirus disease - COVID-19 |
|  | hsa05168 | Herpes simplex virus 1 infection |
|  | hsa05162 | Measles |
|  | hsa05133 | Pertussis |
|  | hsa05164 | Influenza A |
|  | hsa05150 | Staphylococcus aureus infection |
|  | hsa04622 | RIG-I-like receptor signaling pathway |
|  | hsa04621 | NOD-like receptor signaling pathway |
|  | hsa05169 | Epstein-Barr virus infection |
|  | hsa04610 | Complement and coagulation cascades |
